# Essential Genes in the Core Genome of the Human Pathogen *Streptococcus pyogenes*

**DOI:** 10.1038/srep09838

**Published:** 2015-05-21

**Authors:** Yoann Le Breton, Ashton T. Belew, Kayla M. Valdes, Emrul Islam, Patrick Curry, Hervé Tettelin, Mark E. Shirtliff, Najib M. El-Sayed, Kevin S. McIver

**Affiliations:** 1 Department of Cell Biology & Molecular Genetics and Maryland Pathogen Research Institute, University of Maryland, College Park, MD USA; 2 Center for Bioinformatics and Computation Biology, University of Maryland, College Park, MD USA; 3 Institute for Genome Sciences, University of Maryland School of Medicine, Baltimore, MD USA; 4 Department of Microbiology & Immunology, University of Maryland School of Medicine, Baltimore, MD USA; 5 Department of Microbial Pathogenesis, School of Dentistry, University of Maryland School of Medicine, Baltimore, MD USA

## Abstract

*Streptococcus pyogenes* (Group A Streptococcus, GAS) remains a major public health burden worldwide, infecting over 750 million people leading to over 500,000 deaths annually. GAS pathogenesis is complex, involving genetically distinct GAS strains and multiple infection sites. To overcome fastidious genetic manipulations and accelerate pathogenesis investigations in GAS, we developed a *mariner*-based system (*Krmit*) for *en masse* monitoring of complex mutant pools by transposon sequencing (Tn-seq). Highly saturated transposant libraries (*Krmit* insertions in *ca*. every 25 nucleotides) were generated in two distinct GAS clinical isolates, a serotype M1T1 invasive strain 5448 and a nephritogenic serotype M49 strain NZ131, and analyzed using a Bayesian statistical model to predict GAS essential genes, identifying sets of 227 and 241 of those genes in 5448 and NZ131, respectively. A large proportion of GAS essential genes corresponded to key cellular processes and metabolic pathways, and 177 were found conserved within the GAS core genome established from 20 available GAS genomes. Selected essential genes were validated using conditional-expression mutants. Finally, comparison to previous essentiality analyses in *S. sanguinis* and *S. pneumoniae* revealed significant overlaps, providing valuable insights for the development of new antimicrobials to treat infections by GAS and other pathogenic streptococci.

In 2002, *Streptococcus pyogenes* (Group A Streptococcus, GAS) was reported by the World Health Organization among the top 10 causes of morbidity and mortality due to bacterial infections worldwide; and it currently remains a major public health problem^1^. GAS is responsible for a wide range of diseases, most commonly presenting as self-limiting infections of the skin (impetigo) and throat (pharyngitis)^2^,^3^,^4^,^5^. GAS can also enter normally sterile sites and cause severe life-threatening invasive infections (*e.g.* necrotizing fasciitis and toxic shock syndrome), leading to over 150,000 deaths worldwide each year^2^,^4^. Invasive GAS infections typically spread rapidly causing extended tissue damage that require drastic surgical intervention^6^. Moreover, immune-mediated complications following GAS infections (*e.g.*, glomerulonephritis and acute rheumatic fever, ARF), affect children and young adults in developing countries, resulting in over 350,000 deaths each year^2^,^4^. Although GAS is generally treatable with antibiotics and remains one of the few pathogens susceptible to beta-lactams (*e.g.,* penicillin), treatment failures can occur and antibiotic treatment does not guarantee prevention of immune sequelae^7^,^8^,^9^. Low availability of antibiotics in developing countries and the emergence of antibiotic-resistant GAS strains emphasizes the need for novel interventions to address GAS diseases^4^. To help guide the development of new treatment strategies, a better understanding of GAS physiology and pathogenesis during human infection is key.

Essential genes, defined as those necessary for growth and survival under a given condition, represent attractive targets for the discovery of new therapeutics against bacterial pathogens^10^. Identification of these genes on a genomic scale has been of particular interest as it provides candidates for guiding subsequent drug development^11^,^12^. A gene is considered essential when one is unable to generate a viable knockout mutation in that gene under the tested conditions. Gene-by-gene knockout strategies have been successfully implemented to screen the genomes of a few bacteria for essential genes, such as *Escherichia coli*^13^, *Bacillus subtilis*^14^ and *Streptococcus sanguinis*^15^; while, in *S.aureus*, Forsyth *et al*.^16^ successfully used a shotgun antisense RNA method towards this goal. However, these approaches require organisms for which extensive and efficient genetic tools are available (*e.g.*, natural competence). To circumvent the labor and technical difficulties inherent to these approaches, transposon mutagenesis has become a method of choice for genome-scale gene essentiality studies^17^,^18^. Genome-wide identification of mutable genes, and therefore of the non-mutable essential genes, has been carried out using conventional DNA sequencing^19^,^20^, genetic footprinting^21^, and microarray hybridization (transposon site hybridization or TraSH)^22^. More recently, transposon sequencing (Tn-seq) using parallel sequencing of transposon-adjacent chromosome has allowed for the identification of insertions at nucleotide resolution in complex mutant libraries^23^,^24^,^25^,^26^.

Essentiality screens have been successfully carried out in two pathogenic streptococci, the oral pathogen *S. sanguinis* using directed mutagenesis^15^ and the respiratory pathogen *S. pneumoniae* both by TraSH^27^ and Tn-seq^26^; however, these analyses have not yet been undertaken for GAS. Towards this goal, we recently developed a *mariner* transposon (*Oskar*) for stable random mutagenesis in GAS^28^ and used a complex mutant library for TraSH to identify genes that are important for GAS fitness in an *ex vivo* model of human blood infection^29^. Though successful, our study revealed technical bottlenecks of TraSH such as the incomplete representation of the GAS genome on the microarray and the inability to accurately map transposon mutations^29^.

Here, we present the development and application of a modified *mariner*-based transposon (*Krmit*) for the application of Tn-seq in GAS to allow significantly increased mutant monitoring through next-generation sequencing. High saturation mutagenesis was achieved in two divergent GAS strains that are representative of strains with tropism for the throat alone (serotype M1T1 strain 5448) and both the throat and skin (serotype M49 strain NZ131), respectively. Tn-seq was combined with Bayesian analyses integrated over multiple *in vitro* passages to identify highly conserved genes in the GAS core genome that are essential for growth in rich media under optimal conditions. This work establishes a baseline for future Tn-seq studies of GAS growing in disease-relevant environments and provides invaluable information for the discovery of new targets for antimicrobial drugs as well as the study of GAS pathogenesis on a genome-scale.

## Results

### Development of *Krmit:* a transposon for Tn-seq in GAS

Identification of essential genes by Tn-seq requires the production of saturated mutant libraries in a given bacterial genome by transposition^30^. To accomplish this, we modified the *mariner* transposon (*Oskar*) developed for GAS by our group^28^,^29^ for use in Tn-seq. The pKRMIT *in vivo* *mariner* delivery plasmid was produced through PCR-mediated modification of the pOSKAR plasmid to include *Mme*I restriction sites in *Oskar* mini-transposon ITR sequences, creating the new transposon *Krmit* (for Kanamycin-resistant transposon for massive identification of transposants) **(Fig. S1AB)**. The presence of *Mme*I sites allows for digestion of genomic DNA containing *Krmit* transposon insertion sites (TIS), producing uniform 20-nt regions of adjacent chromosomal DNA (insertion tags) for next-generation sequencing and Tn-seq screens^31^. Initial tests for *in vivo* transposition using pKRMIT in a GAS serotype M1T1 strain revealed that *Krmit* transposed comparably to *Oskar*^28^,^29^, exhibiting an average transposition frequency of 4 × 10^−3^ with insertions occurring exclusively within the dinucleotide TA (**Fig. S1C**). *Krmit* transposition in different GAS serotypes (*e.g*. M1T1, M3, M6, M49) showed similar results (data not shown), demonstrating that introduction of the two *Mme*I sites did not affect *in vivo* transposon delivery in GAS. Since pKRMIT relies on the pWV01 replicon, it has the potential to be used in other closely related Gram-positive genera (*e.g.,* *Streptococcus*, *Enterococcus*, and *Lactococcus*).

### Selection of M1T1 GAS *Krmit* libraries under *in vitro* growth

*Krmit* transposition was performed in the GAS strain 5448, a representative of the invasive M1T1 serotype circulating worldwide that is associated with a throat tropism (Pattern A, Class I *emm* gene)^32^. For *in vivo* *Krmit* transposition in M1T1 5448, the protocol developed for *Oskar* was used^28^,^29^ as detailed in Materials and Methods. Following transformation with pKRMIT, a set of individual colonies (*ca*. 120) was subjected to phenotype screening to verify the presence of the intact plasmid and to select appropriate mutant libraries (*ca*. 24) for further genetic analyses. A low percentage of spectinomycin-resistance (Sp^r^) among *Krmit* transposants (< 5%) was preferred to limit the frequency of the pKRMIT plasmid within the mutant pools; an unwanted phenomenon previously observed^29^. A total of 15 libraries with a plasmid integration frequency ranging from 0.05% to 5% (av. 2.6%) were selected for arbitrary-primed PCR (AP-PCR) analyses to identify the *Krmit* transposon insertion site (TIS) in at least 20 randomly picked mutants per library. Transposon insertion randomness was found to vary widely between the tested mutant pools (35% to 95%).

To determine the set of genes essential for M1T1 5448 growth *in vitro* in "rich" Todd-Hewitt Yeast (THY) medium under typical laboratory conditions (37°C, 5% CO_2_), four independent *Krmit* mutant libraries with optimal randomness were selected and subjected to three additional 24-hour passages under these conditions. This approach allowed for *en masse* mutant selection over continued *in vitro* growth in THY (T_0_ to T_3_), with mutants in essential genes predicted to be lost from the library in the early passages (T_0_, T_1_) and those with reduced fitness being lost only during the final passages (T_2 _to T_3_). The screen resulted in a total of 16 different passaged libraries (4 libraries at 4 time points) for the Tn-seq analyses.

### Tn-seq analyses of *in vitro*-grown M1T1 5448

The complexity of the *Krmit* mutant libraries in M1T1 5448 was examined by Tn-seq^26^,^31^ with modifications for its use in GAS with *Krmit*. Briefly, genomic DNA was isolated from the different mutant pools (T_0_ to T_3_), subjected to complete digestion by *Mme*I followed by ligation to *Mme*I adapters, and PCR amplification to produce DNA-seq libraries consisting of 176-bp *Krmit*-specific insertion tags containing adjacent genomic sequence, Illumina-specific sequences and one of 8 distinct barcodes for multiplexing. DNA-seq reads containing both the barcode sequence (specific for *Krmit* mutant libraries) and the *Krmit* ITR sequence were assigned into 16 distinct Tn-seq datasets, each containing, on average, a total of *ca*. 13.7 millions reads (Table S1).

For genome alignment of the reads, the 20-nt sequence corresponding to the *Krmit* TIS was retained and the dinucleotide TA used as a means to orient the transposon on the chromosome. The complete annotated genome of M1T1 5448 is not publically available; however, the draft genome sequence was found to be almost identical to the reference genome of M1T1 MGAS5005^33^,^34^ and the latter was used for mapping. Sequencing reads that aligned to more than one position on the MGAS5005 chromosome (*ca*. 5% of the total read counts, Table S1) were kept in the datasets and randomly mapped to one candidate position on the genome. The orientation of *Krmit* insertions showed an equal distribution of TIS on the forward and reverse strand of the chromosome (data not shown).

Over 90% of the reads matched perfectly to the reference GAS genome in the different datasets, with the exception of the T_0_ datasets where this proportion varied from 69% to 6% (Table S1). The reads that did not align to the GAS genome were found to correspond to sequences on pKRMIT, with the vast majority aligning to the region adjacent to *Krmit* on the plasmid (data not shown). This indicated that pKRMIT was still present during the initial passage of the mutant libraries (T_0_); however, it was lost during further passages in THY (T_1_, T_2_ and T_3_) (Table S1).

**Figure 1:**
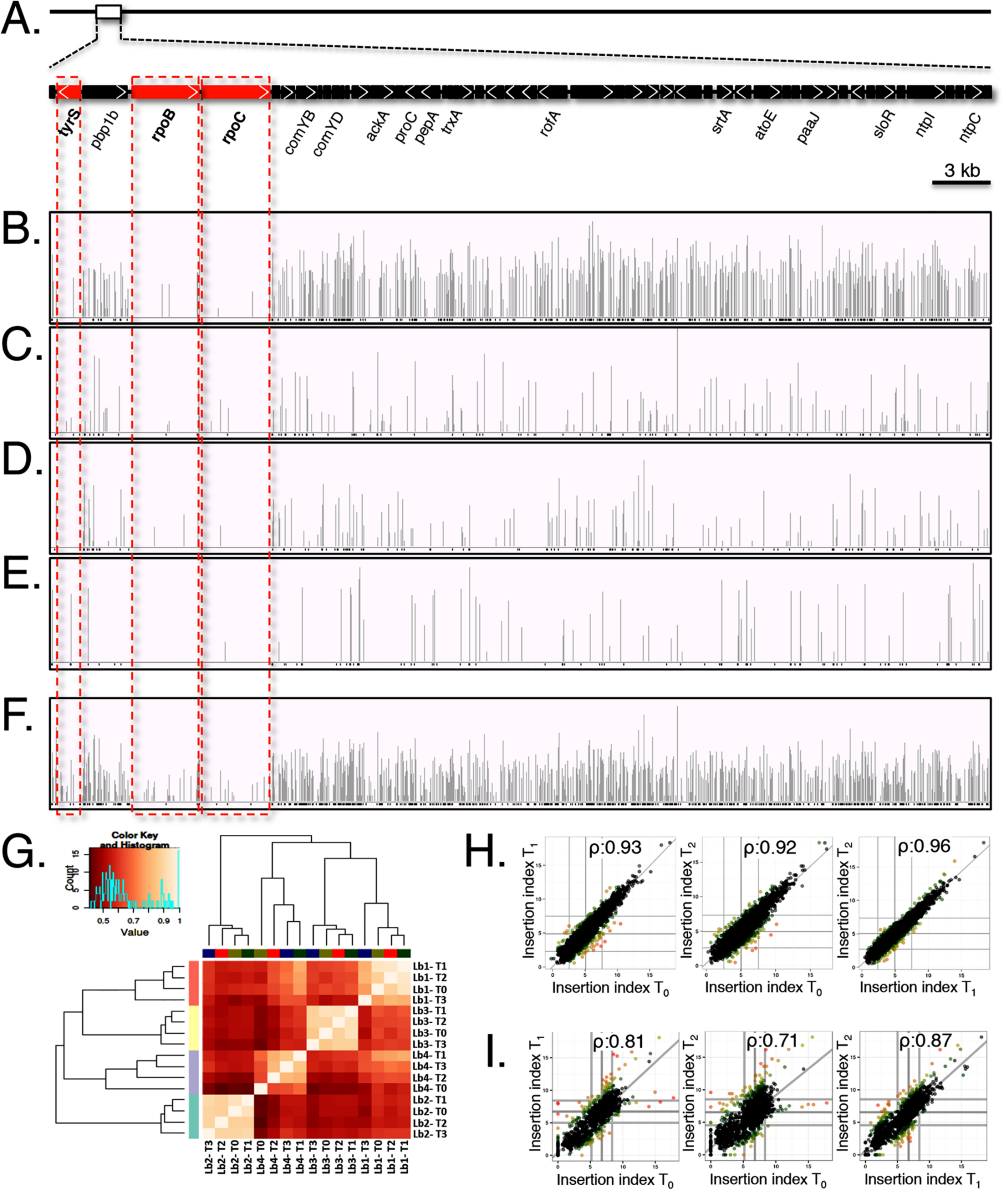
Comprehensive *Krmit* mutant libraries in GAS M1T1 5448 and M49 NZ131. (A) Schematic of GAS M1T1 5448 chromosomal region from *tyrS* to *ntpC*. Genes that possess limited *Krmit* transposon insertion sites (TIS) determined by Tn-seq and Bayesian analysis are shown in red. Scale bar is indicated at right. (B to E) Location (horizontal axis) and depth (vertical axis) of all *Krmit* TIS identified within the 5448 genomic region in four independent mutant libraries as determined by Tn-seq at time point T_0_ presented using IGV. (F) Location and density of *Krmit* TIS in the “Master” M1T1 5448 library generated by merging the 4 independent libraries B-E. (G) Heat map summarizing the pairwise comparison of the four independent M1T1 5448 *Krmit* mutant libraries as determined by Tn-seq at each time point (T_0_, T_1_, T_2_ and T_3_; 16 total libraries) using Spearman’s rank correlation coefficients. Scatter plot summarizing the pairwise comparison of the *Krmit* TIS in the M1T1 5448 “Master” library (H) and the M49 NZ131 library (I) at different time points using Spearman’s rank correlation coefficients.

Visualization of the read alignments using IGV^35^,^36^ revealed that the initial libraries (T_0_) contained distinct *Krmit* insertion patterns as shown for the genomic region from the *tyrS* to *ntpC* genes (Fig. 1A) with TIS density being highest in library 1 (Fig. 1B) compared to libraries 2 to 4 (Fig. 1C-E). Determination of the unique *Krmit* TIS and the percentage of TAs containing a transposon correlated with this result (Table S1). Library 1 TIS density (*ca*. 45% of TAs targeted) was significantly higher than the TIS density in the other libraries (20% or less). Our results also showed that TIS density typically increased between T_0_ and T_1 _(Table S1), likely due to continued transposition in T_0_ libraries that still contained pKRMIT.

Pairwise comparison analyses, including Principal Component Analysis (**Fig. S2A**), Euclidean distance (**Fig. S2B**), and Spearman’s rank correlation (Fig. 1G), were performed to determine the degree of similarity between the 16 different mutant pools. The results revealed that the members of each *Krmit* mutant library (i.e., same library at different time points) were much more similar to one another than to the other libraries. Thus, although the individual libraries were random, they were not saturated enough individually to permit optimal library comparison and gene essentiality predictions. Therefore, data from the four independent *Krmit* libraries (Lb1, Lb2, Lb3 and Lb4) were combined to generate a single high-density *Krmit* insertion dataset for each of the four passages (T_0_ to T_3_). This “Master” library in M1T1 5448 was found to contain on average 45.8 million aligned reads corresponding to an average of over 85,000 unique TIS in all four passages (Table S1), representing *Krmit* integration in over 64% of the available TAs on the M1T1 5448 genome or one *Krmit* TIS for every 22 nucleotides. Read alignments revealed the increased complexity of the library (Fig. 1F**, data not shown**), showing high numbers of *Krmit* insertions in most genes. Spearman’s correlation rank analyses found that the master libraries at the 4 time points were highly comparable with ρ values over 0.92 (Fig. 1H).

### Bayesian prediction of GAS 5448 essential genes

A recently developed Bayesian statistical model^37^ was used to analyze the Tn-seq data generated from M1T1 5448 to rigorously predict the essentiality of individual genes. Essential genes are typically defined as those that do not tolerate disruption and should be identified in our datasets as loci that lack *Krmit* insertions. However, recent Tn-seq analyses in other bacteria have shown that in addition to genes completely lacking transposon insertions, essential genes may also have insertions in the extreme 5’ and 3’ ends of the open reading frame or in sequences encoding non-essential domains (Fig. 1F; *tyrS*, *rpoB* and *rpoC*)^17^,^38^. To account for this, the DeJesus Bayesian method^37^ makes rigorous predictions of statistically significant stretches of TA sites within a given gene lacking transposon (*i.e*., *Krmit*) insertions regardless of the presence of insertions in other regions in the ORF. For stringency, TA sites with just one insertion were discarded from our datasets as these could represent sequencing errors. This filter resulted in a significant coverage reduction for the Tn-seq dataset generated at time point T_3_ (Table S2) and consequently this dataset was excluded from all further analyses.

**Figure 2:**
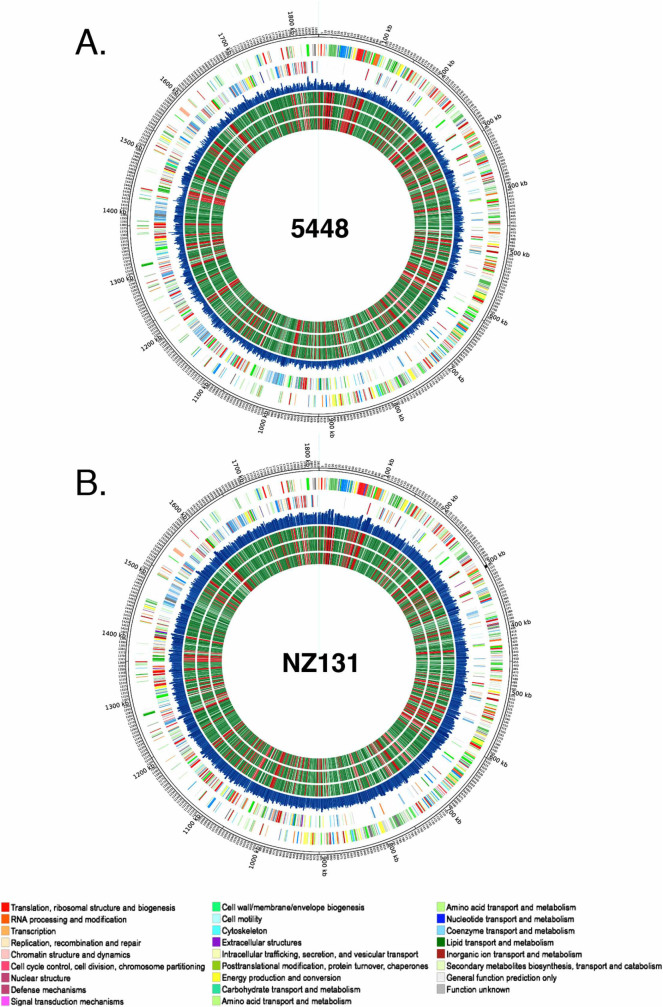
Bayesian prediction of gene essentiality in GAS M1T1 5448 and M49 NZ131 genomes at multiple time points. Circos atlas representation of M1T1 5448 (A) and M49 NZ131 (B) genomes are shown with base pair (bp) ruler on outer ring. Next two outer circles represent GAS open reading frames on the (+) and (−) strands, respectively, with colors depicting COG categories (legend at bottom). The next circle (blue) indicates the frequency of *Krmit* TIS in each genome at T_0_. The inner three circles present the results of Bayesian analysis of GAS gene essentiality at time points T_0_, T_1_ and T_2_ in order towards center; with essential genes (E^Bay^) in red, non-essential genes (NE^Bay^) in green, and excluded genes in black.

The Bayesian analysis generated a posterior probability of essentiality *Z_i_* score for every M1T1 5448 gene in each remaining dataset (T_0_, T_1_ and T_2_), assigning each gene into 4 distinct fitness categories: non-essential gene (NE*^Bay^*) (0.0 < *Z_i_* < 0.05), essential gene (E*^Bay^*) (*Z_i_*> 0.995), non-conclusive data (scarcity of insertions) (0.05 < *Z_i_* < 0.995), and gene too small for analysis (*Z_i_* = −1)^37^. The resulting Bayesian analysis for M1T1 5448 is illustrated in Fig. 2A and detailed in **Table S3**.

To integrate the results of the Bayesian analysis at all three time points (T_0_, T_1_ and T_2_), we established the following four penultimate categories: a gene was “Essential” when found to be E*^Bay^* at all 3 time points (T_0_, T_1_ and T_2_) or the latter 2 time points (T_1_ and T_2_); a gene was “Critical” for fitness when found to be E*^Bay^* only at the final T_2_ time point; and a gene was “Non Essential” if found NE*^Bay^* at all 3 time points. Those genes that did not meet any of these three criteria were categorized as “Non-conclusive”. Using those stringent criteria on the 1841 annotated genes in the M1T1 5448 genome, our analyses found that 227 (~12%) were “Essential”, 71 (~4%) were “Critical”, 1337 genes (~73%) were “Non-Essential”, and 206 genes (~11%) were “Non-Conclusive” for *in vitro* growth (Table S4).

### Genes “Essential” for *in vitro* growth of GAS M49 strain NZ131

To compare essential genes between two divergent GAS strains, *Krmit* mutant libraries were produced in the GAS serotype M49 isolate NZ131, a nephritogenic representative of “generalist” GAS strains associated with both throat and skin infections (Pattern E, Class II *emm* gene)^32^. NZ131 has a fully annotated genome sequence that is publically available^39^. *Krmit* transposition proved more efficient in the highly transformable NZ131, facilitating the selection of complex *Krmit* mutant libraries (data not shown). Tn-seq analysis was performed as described above on a selected mutant library passaged 4 times in THY broth. Even though read alignments showed that the M49 NZ131 *Krmit* mutant library did not have the depth (18 millions aligned reads on average) of the master library in M1T1 5448 (45.8 millions aligned reads on average), the TIS saturation of NZ131 was reasonably similar to that observed for 5448 with a *Krmit* insertion every 27 nucleotides compared to every 22 nucleotides, respectively (Table S1). Furthermore, a Spearman’s correlation rank analyses showed that the passaged libraries were very similar (Fig. 1I).

The Tn-seq data from the M49 NZ131 *Krmit* libraries at each time point was subjected to Bayesian analysis as described for M1T1 5448 (Fig. 2B and **Table S5),** followed by integration of time points into our defined gene categories (Table S6). As with 5448, the T_3_ time point was removed from the analysis due to low *Krmit* coverage. Out of the 1698 annotated genes in the NZ131 genome, 241 genes (~14%) were “Essential”, 45 genes (~3%) were “Critical”, 1177 genes (~69%) were considered “Non-Essential”, and 235 genes (~14%) were found “Non-Conclusive” (Table S6).

### Conserved "Essential" genes in the core GAS genome

**Figure 3:**
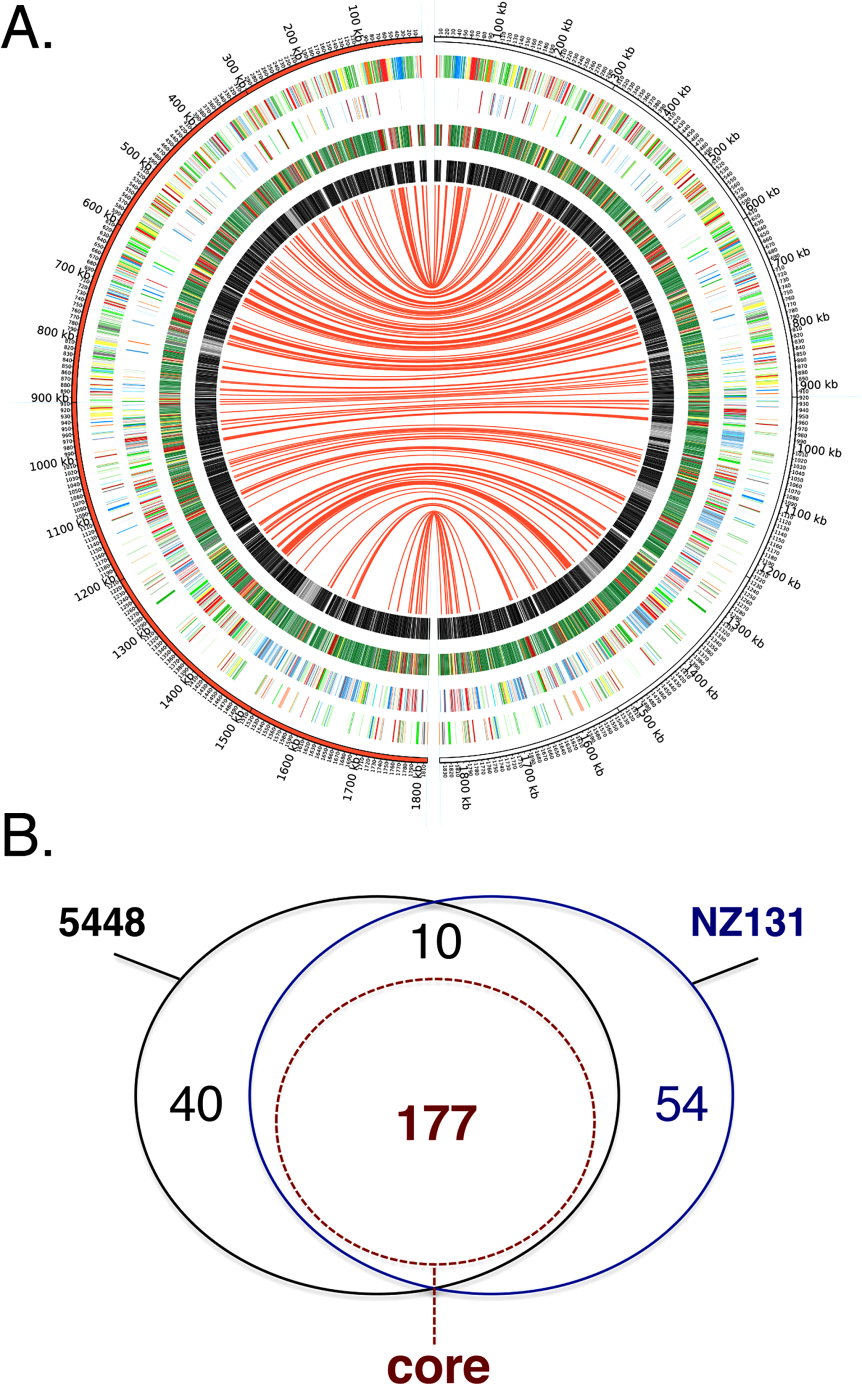
Conserved GAS gene essentiality based on integration of Bayesian analysis at all time points. Bayesian prediction datasets at the time points T_0_, T_1_ and T_2_ were integrated for both M1T1 5448 and M49 NZ131 into the categories "essential", "critical", "non-essential", or "non-conclusive". (A) Circos atlas representation of M1T1 5448 (left, red semicircle) and M49 NZ131 (right, white semicircle) genomes are shown with base pair (bp) ruler on outer ring. Next two outer circles represent GAS open reading frames on the (+) and (−) strands, respectively, with colors depicting COG categories (see Fig. 2). The next circle presents the integrated analysis of GAS gene essentiality at all time points; with "essential" (red bars), "critical" (yellow bars), "non-essential" (green bars), and "non-conclusive" (black bars) genes indicated. Inner circle represents a genomic comparison between the M1T1 5448 and M49 NZ131 with homology (black) and non-homology (grey) shown. Red centerlines connect conserved "essential" genes shared between the two GAS genomes. (B) Venn diagram representing a comparison between the integrated "essential" genes found in GAS M1T1 5448 (227 genes, black) and M49 NZ131 (234 genes, blue) genomes. Shared "essential" genes also found within the GAS "core" genome (177 genes, see Fig. 4) are shown (dashed red line). See Table S7 for detailed list of genes.

**Figure 4:**
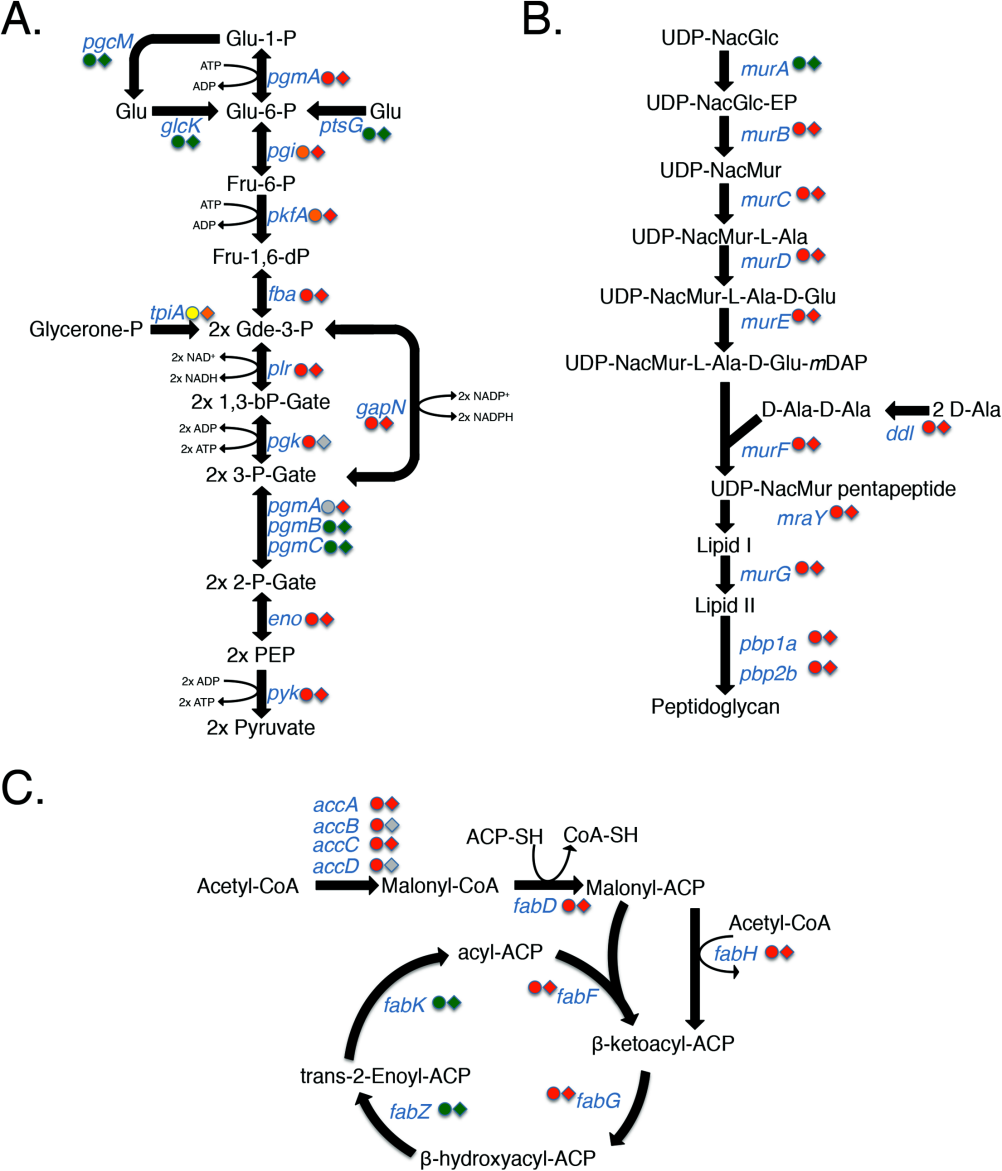
GAS essential genes correlate to key metabolic pathways and cellular functions. Schematics of the pathways for (A) Glycolysis, (B) Peptidoglycan biosynthesis, and (C) Fatty acid biosynthesis are shown, including reactants/products (black font) and genes encoding key enzymes (blue font). Integrated Bayesian analysis results for key enzymes are shown for GAS 5448 (circles) and NZ131 (diamonds) with "essential" genes in red; "critical" genes in yellow; "non-essential" genes in green; and "non conclusive" genes in grey.

The integrated essentiality results from M1T1 5448 and M49 NZ131 were compared (Fig. 3 and Table S7). Of the genes found "Essential" in 5448 (227) and NZ131 (241), 187 were found to overlap in both strains and most likely represent essential genes shared by many GAS strains (Fig. 3A and Table S7). Comparison of the GAS 5448 and NZ131 datasets revealed the importance of conserved essential genes in key metabolic pathways such as glycolysis (Fig. 4A), peptidoglycan biosynthesis (Fig. 4B) and fatty acid synthesis (Fig. 4C).

Of the non-overlapping "Essential" genes (*i.e*. 40 genes for 5448; 54 genes for NZ131), only a few (*i.e*., 1 in 5448 and 2 in NZ131) were located in prophages found in one GAS strain, but not the other **(**Fig. 3B). The remaining genes were found in both strains, but classified in different categories: "Essential" in 1 strain, but either "Critical", "Non-essential", or "Non-conclusive" in the other. Overall, the comparison revealed a set of 187 conserved "essential" genes found in both 5448 and NZ131 (Fig. 3B and Table S7).

A "core genome" is defined as the genes shared by all strains of a given species as determined by comparative genomics of multiple sequenced genomes^40^. There are currently 20 annotated GAS genome sequences publically available representing *emm* serotypes correlated with tissue tropism^32^, including pattern A-C associated with throat infections (**M1**, M3, M5, M6, M12, M14, and M18), pattern D associated with skin infections (M53), and pattern E (generalists) that can colonize both sites (M2, M4, M28, **M49**, and M59). The GAS core genome was determined using these sequenced genomes (see Materials and Methods), identifying a total of 1224 orthologous genes common to all of the GAS strains (Fig. S3 and Table S7). The vast majority of the genes categorized as either "Essential" or "Critical" in M1T1 5448 (271/298 or 91%) and M49 NZ131 (267/286 or 93%) were part of the GAS core genome (Table S7). Furthermore, of the 187 "Essential" genes shared by both 5448 and NZ131, 177 were present in the GAS core genome (Fig. 3B, Fig. S4 and Table S7) and likely to be essential for all GAS strains.

### Validation of selected GAS "Essential" genes

**Figure 5:**
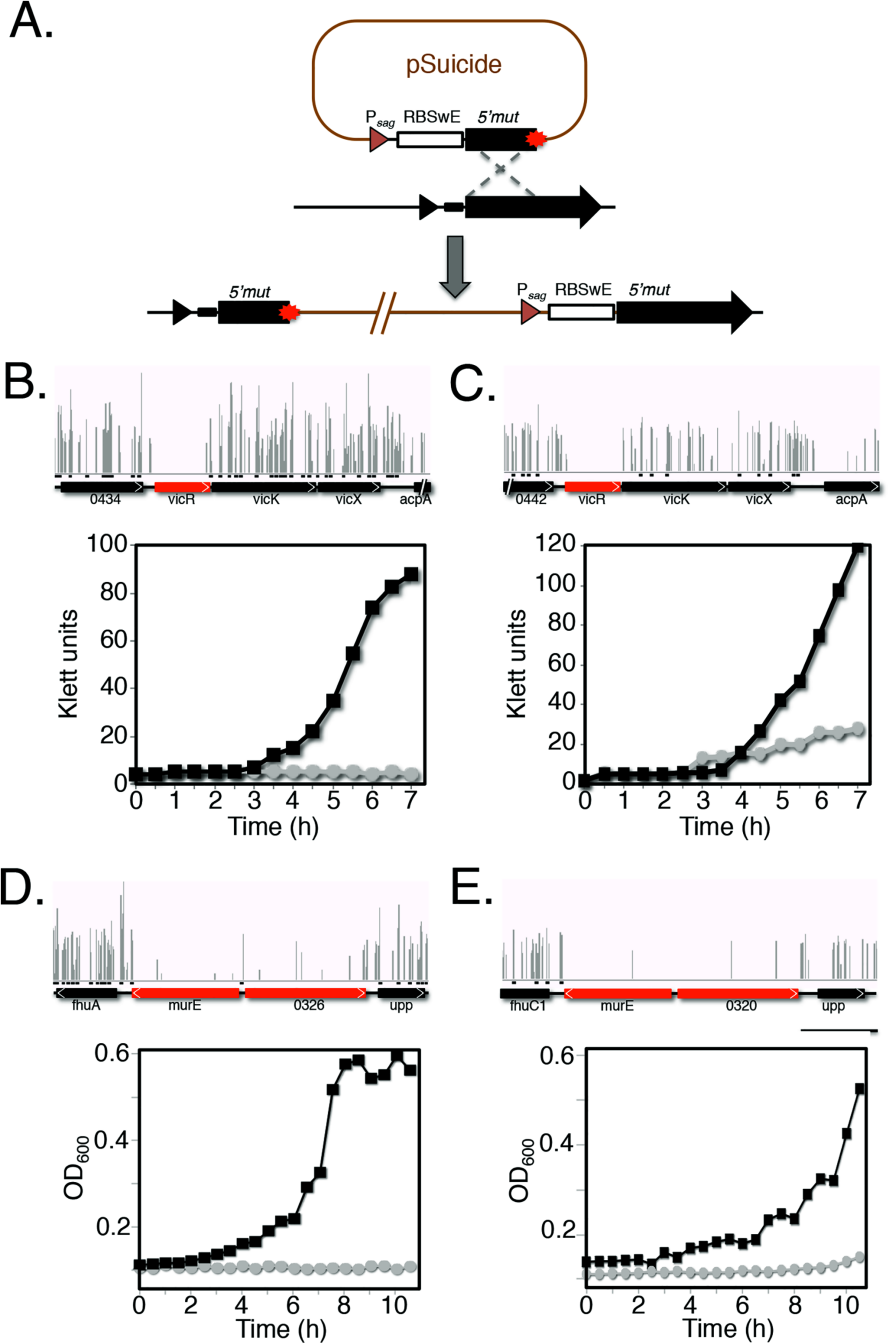
Experimental validation of selected essential genes. (A) Schematic showing construction of GAS conditional expression mutants controlled by riboswitch E. The 5’-end of the selected gene was cloned along with the P*_sag_* promoter and a theophylline-inducible riboswitch into the new pSinS vector for stable chromosomal integration in GAS. Merodiploid integrants have target gene expression under the control of the riboswitch, while the wild-type promoter controls the expression of a truncated target gene. (B to C) Validation of *vicR* (B to C) and *murE* (D to E) as an essential gene in GAS 5448 (B and D) and NZ131 (C and D). The top section of each panel represents the distribution of *Krmit* TIS in the each locus. The bottom section shows the growth of an inducible mutant in the presence (black squares) or absence (grey circles) of theophylline.

To confirm that conserved genes identified in our screen are required for *in vitro* growth of GAS, we utilized a conditionally lethal approach that took advantage of a theophylline-sensitive synthetic riboswitch functional in GAS^41^. A suicide/helper system pSin-pHlp was created for GAS to allow for stable insertional gene inactivation (see Materials and Methods). Stable plasmid integration was generated in the GAS chromosome resulting in a merodiploid strain containing the heterologous P*_sag_* promoter followed by the theophylline-inducible riboswitch E controlling expression of the full length targeted gene, while the wild type promoter of the targeted gene transcribed a truncated non-functional allele (Fig. 5A).

Two conserved essential genes were selected to validate our essentiality findings in both serotypes; *murE,* encoding a UDP-N-acetylmuramoylalanyl-D-glutamate-L-lysine ligase involved in peptidoglycan synthesis, and *vicR,* encoding a two-component system response regulator shown to be essential in various Gram-positive bacteria, including *B. subtilis*^42^, *E. faecalis*^43^, *S. aureus*^44^, *S. pneumoniae*^26^ and *S. sanguinis*^15^. Both 5448 and NZ131 *vicR*-inducible strains were viable when theophylline was provided into the growth medium, yet showed significantly compromised growth in the absence of theophylline (Fig. 5BC). Similarly, the *murE*-inducible mutants were not viable in the absence of theophylline yet grew normally in the presence of the inducer (**Fig. 5DE**). Together, these data validate that *murE* and *vicR* do represent genes essential for *in vitro* growth of both M1T1 5448 and M49 NZ131 as indicated by our analysis.

### Essential genes conserved amongst pathogenic streptococci

**Figure 6:**
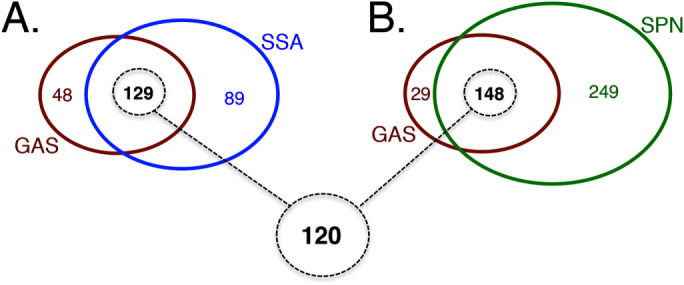
Essential gene set conserved between pathogenic streptococci. Venn diagram showing the comparison of the 177 essential genes identified in the GAS core genome to found in (A) *Streptococcus sanguinis* (blue circle) and (B) *Streptococcus pneumoniae* (green circle). Overlap of essential genes conserved between all three streptococcal pathogens is shown at bottom (dashed circle).

In order to identify essential genes conserved across the genus *Streptococcus*, we compared the conserved 177 genes found "Essential" in the GAS core genome to the results obtained from published essentiality studies performed in two other pathogenic streptococci. Xu *et al*.^15^ identified 218 genes essential for *S. sanguinis* growth *in vitro* in THY using a gene-by-gene inactivation strategy, while van Opijnen *et al*.^26^ used Tn-seq to report 397 genes in *S. pneumoniae* that were either essential or likely essential for growth under comparable *in vitro* conditions. Orthologs of the GAS core "Essential" genes were determined within the *S. sanguinis* and *S. pneumoniae* genomes using the COG database and then compared to the published essential gene lists. Of the 177 genes identified as essential in GAS, 129 and 148 genes were shared in the essential gene sets of *S. sanguinis* and *S. pneumoniae*, respectively (Fig. 6); and a total of 120 genes (Table S7) were found to be "Essential" in all three *Streptococcus* species. We also identified a total of 8 genes "Essential" for GAS, but unambiguously dispensable for *S. sanguinis* and *S. pneumoniae*, including those encoding the heat shock protein gene *grpE*, the cobalt ABC transporter gene *cbiO*, group A carbohydrate biosynthesis genes *(gac)*, and 3 genes encoding poorly characterized proteins. Overall, the 120 conserved streptococcal "Essential" genes identified here represent attractive targets for the potential development of antimicrobials against multiple streptococcal human pathogens.

## Discussion

GAS remains a considerable disease burden worldwide, resulting in over half a million deaths each year^1^. Despite considerable efforts, progress in designing new vaccines against this significant pathogen has been slow in comparison to other infectious diseases^45^. GAS pathogenesis is complex as it involves multiple infection sites and genetically divergent GAS clinical isolates. Systematic genome-wide forward-genetic approaches should provide invaluable resources for a better, large-scale understanding of GAS physiology and virulence^29^,^46^,^47^.

In this work, we modified our recently developed *mariner* system for whole-genome TraSH screens in GAS^28^,^29^ to create *Krmit*, a transposon system for Tn-seq analyses. This work significantly improves high-resolution whole-genome mutant screens in GAS, as we are now able to track complex mutant pools at nucleotide resolution. As a first application of the new *Krmit* system, we identified a set of 177 genes essential for growth in rich medium *in vitro* and conserved in the GAS core genome, as well as identified those essential genes conserved in the genomes of multiple pathogenic streptococci and representing valuable targets for vaccine and drug development.

### *Krmit* and the production of *Krmit* libraries for Tn-seq in GAS

Tn-seq^26^ allows for *en masse* identification of mutants in highly complex pools through the production of specific TIS tags. To allow for both qualitative (TIS tag location) and quantitative (gene fitness index) analyses of the mutant pool composition, a *mariner* transposon with modified *Mme*I-containing ITRs is used to produce TIS tags of identical size. However, the ITR modification must have no deleterious effect on the *mariner* transposition. We were able to show that the ITR modification of *Krmit* did not significantly impact *in vivo* *mariner* delivery in different GAS genetic backgrounds (**Fig. S1**) compared to the published parental *Oskar* *mariner* system.

The M1T1 strain 5448 represents a model for the study of GAS invasive pathogenesis as it produces strong virulence phenotypes in different *in vivo* and *ex vivo* infections models. However, genetic manipulation of 5448 has proven fastidious and impacts *mariner* transposition efficiency^28^,^29^. Thus, phenotype screens and AP-PCR analyses were necessary on 120 independent 5448 *Krmit* libraries in order to select for the most desirable libraries prior to the production and sequencing of *Krmit* TIS tags. Tn-seq analyses of four of these 5448 *Krmit* libraries emphasized the heterogeneity in the *mariner* mutagenesis randomness and the necessity to pool together different libraries to achieve near-saturation mutagenesis in GAS 5448 (Fig. 1). In contrast, *Krmit* library production in the highly transformable GAS NZ131 was found to be more straightforward, needing analysis of far fewer independent libraries and not necessarily requiring pooling (Fig. 1). Thus, as previously observed^28^, ease of *in vivo* *Krmit mariner* delivery in GAS appears to strongly correlate with the transformation efficiency of the strain and this fact should be kept in mind during future Tn-seq studies using *Krmit* in GAS.

Tn-seq revealed the presence of the pKRMIT delivery plasmid in the first passage of *Krmit* libraries (T_0_) (Table S1). Originally seen as an inconvenient contamination of the mutant libraries that could affect library stability^28^,^29^, our Tn-seq data showed that the plasmid presence in T_0_ leads to significantly increased library TIS in the subsequent passage (T_1_) combined with plasmid dilution and disappearance. This observation shows that maximum coverage and near saturation mutagenesis is achieved in poorly transformable GAS strains only after two consecutive overnight passages *in vitro*. Furthermore, these T_1_ libraries should represent the "input" libraries for comparison to different *ex vivo* and *in vivo* GAS growth environments.

Tn-seq also revealed high mutagenesis saturation at T_1_; with *Krmit* transposons found *ca*. every 25 nucleotides in both the GAS 5448 and NZ131 genomes (Fig. 2). To our knowledge, this work provides the most comprehensive mutant libraries yet produced in GAS and represents an invaluable resource for the study of GAS physiology and pathogenesis. As Tn-seq provides the means to track the libraries’ complexity at a nucleotide scale, this is a significant improvement over our recent TraSH screens where, due to the limitations of microarrays, we could only assess *ca*. 60% of the GAS genome^29^. Moving forward, Tn-seq’s resolution has the potential to monitor qualitatively (saturation index) and quantitatively (fitness index) libraries’ composition. We are currently using Tn-seq with the 5448 and NZ131 *Krmit* libraries to investigate GAS physiology and pathogenesis. The level of *Krmit* saturation achieved in GAS 5448 and NZ131 chromosomes should provide the means to determine the contribution of both protein-coding genes and non-coding regions.

### Prediction of essential genes for GAS 5448 and NZ131

As a first application of our new resources, we chose to identify GAS essential genes as these provide valuable targets for the development of novel therapeutic interventions and identify genes to be removed from future Tn-seq analyses of GAS fitness under *ex vivo* and *in vivo* growth conditions. Tn-seq data analyses present some challenges for gene essentiality prediction and different methods are emerging for computational analyses. One approach is to determine the frequency of a given mutant in the population (read depth) and average the results to generate a fitness value (fitness index) for each gene^23^,^24^,^25^,^26^,^31^,^48^,^49^,^50^,^51^. Initial attempts revealed that this approach was not suited for essential gene prediction using our datasets (data not shown), likely due to the protocol used to produce mutant libraries. Since transposition events were not synchronized during our *in vivo mariner* delivery procedure, mutants that appeared early during the procedure had the potential to become overrepresented within the mutant pool, thus affecting the read depth for the corresponding location. Read mapping analyses confirmed this hypothesis showing overrepresented clones (data not shown).

An alternative approach for gene essentiality prediction consists of searching for genes with significant gaps of transposon insertions (coverage) regardless of read depth^37^,^52^,^53^,^54^,^55^. Here we used the computational pipeline developed by DeJesus *et al*.^37^ to make prediction of essential genes by means of a Bayesian statistical model that scanned the GAS genome to determine the distance between *Krmit* insertions within the GAS genomes and identify abnormal insertion gaps as a call for gene essentiality (Fig. 2; **Tables S3 and S5**). We then integrated the analyses obtained from 3 consecutive *in vitro* passages of the *Krmit* libraries (T_0_, T_1_ and T_2_) into 4 categories: essential, critical, non-essential and non-conclusive (**Tables S4 and S6**). Using this approach, we identified a set of 227 and 241 essential genes in GAS 5448 and NZ131, respectively. Perhaps not surprisingly, a large proportion of the genes found essential in both GAS strains corresponded to genes involved in basic cell functions such as DNA replication, RNA transcription, aminoacyl-tRNA synthesis, ribosomal proteins, central carbon metabolism, generation of proton motive force, peptidoglycan biosynthesis, and fatty acid synthesis. The paucity of known virulence factors^56^ in the list of genes essential for GAS growth *in vitro* likely reflects the host-specific requirement for these genes *in vivo*. There was a striking overlap with our data and a "minimal" essential gene set^57^ developed through the integration of data collected from different Gram-negative and Gram-positive bacterial species using various experimental approaches (mutagenesis, bioinformatics) (data not shown). This provides a strong validation of the integrated Bayesian approach used to define GAS essential genes.

When directly comparing the results obtained in 5448 and NZ131, we found a total of 187 essential genes common to the two diverse GAS strains (Fig. 3). Therefore, our analysis also identified 94 genes essential exclusively in one GAS strain, but not in the other. There are several explanations that could account for these findings.

First, some of these essential genes were identified in strain-specific phages, where they typically encode for phage repressor proteins from the cI/Cro-family, including the 5448 PhiRamid phage^58^ (M5005_Spy_1464) or the ΦNZ131.1 phage^39^ (Spy49_0369). Additional phage cI/Cro repressor-encoding genes were also found to be critical (Table S4, S6 and S7). The suggestion would be that loss of the repressor leads to induction of the lytic phage and loss of the mutant from the population.

Second, the conservative nature of our essentiality call could exclude a gene that was deemed non-conclusive in the other strain leading to an underestimation of the shared GAS essential genes. We found that analysis of the data within metabolic pathways using the KEGG database (*e.g*., central carbon metabolism, peptidoglycan and fatty acid biosynthesis) (Fig. 4) and/or comparison of visualized read alignments for data in both GAS strains (Fig. 1** and **5) helped to reveal the critical or essential roles of non-conclusive genes in vital cell processes.

Third, GAS strain-specific essential genes may also reflect loci that are more related to fitness (*e.g.*, environment stress response) than being truly essential, where the competitive environment of the *en masse* mutant selection led to an underrepresentation or disappearance of mutants that were less fit in one of the GAS strains. The distinction between "gene essentiality" and "fitness disadvantage" can be quite subtle in Tn-seq^26^,^59^,^60^,^61^. Recently, Valentino *et al*.^51^ found, as they were attempting to experimentally validate genes identified by Tn-seq as essential in *S. aureus*, that 50% of the conditional mutants tested presented delayed growth. We found a number of genes linked to stress responses that were called essential in one of the strains but not in the other. For example, *codY* and *ccpA* were found essential in NZ131, but critical and non-conclusive, respectively, in 5448. These two genes encode transcriptional regulators involved in stress responses to nutrient limitation that are likely to be experienced by the GAS cells in our experimental setting. Previous analyses have shown that both *codY*^62^ and *ccpA*^63^,^64^,^65^ were mutable in various serotypes of GAS. Similarly, we found *covS*, encoding the histidine-kinase of the CovRS two-component system in GAS, to be essential in NZ131 but not in 5448. Although *covS* has been mutated by many groups^66^,^67^,^68^,^69^, Dalton and Scott showed that a *covS* mutant was sensitive to physicochemical stresses^67^.

Another explanation for GAS strain-specific essential genes might be that the mechanisms involved in the environmental stress response during *in vitro* library selection could be different in the two GAS strains.

### Conserved essential genes in the "Core" GAS genome and other streptococci

Molecular epidemiology has identified a GAS population genetic structure with 3 discrete subpopulations based on tissue tropism^32^, including skin specialists (Pattern D), throat specialists (Pattern A-C) and generalists (Pattern E). Importantly, any effective therapeutic should target all 3 distinct subpopulations. By comparing the available genome sequences of 20 different GAS strains reflecting these subpopulations, we were able to identify a set of 1224 genes common to all GAS strains (GAS core genome). Of these, 177 genes were unambiguously identified as essential in our Tn-seq analysis of GAS 5448 and NZ131. Furthermore, 120 of these core GAS essential genes were also found to be essential in *S. sanguinis* and *S. pneumoniae* (Fig. 6). These two sets of essential genes represent interesting candidates for the development of drugs against GAS and multiple streptococcal pathogens, respectively. Currently, the most successful antibiotics exploit a small number of targets, *i.e*. the ribosome, protein synthesis, cell wall synthesis, folic acid metabolism, RNA polymerase, DNA gyrase and DNA topoisomerase^70^. Our data identifies additional essential genes and diverse pathways that could be the targets for novel antimicrobial approaches (**Table S7**).

By identifying genes that are non-essential, essential or critical for *in vitro* GAS fitness, our data also provides invaluable information for the investigation of GAS pathogenesis as it provides a roadmap for GAS genetic manipulation. Whereas genes shown as non-essential in both GAS 5448 and NZ131 are likely to be easy targets for null mutations, essential or critical genes might potentially require more effort. GAS genetic manipulations of these genes that require extensive strain passaging could give rise to compensatory (suppressor) mutations masking the true phenotype of the gene initially targeted. One example from our data is *vicR*, which has previously been shown to be mutable albeit with considerable effort^71^, but here is clearly found to be an essential gene both by Tn-seq and through conditional gene expression **(**Fig. 6** and Table S7)**.

## Concluding remarks

The development of *Krmit* and its use for Tn-seq in GAS now allows for unparalleled *en masse* monitoring of mutants to assess gene essentiality and fitness on a genome-wide scale in this important human pathogen. In addition to the invasive M1T1 5448 and the nephritogenic M49 NZ131 strains, we have established complex libraries in many other GAS serotypes as a resource for the GAS pathogenesis community. Using Tn-seq in conjunction with RNA-seq, we can now explore the functional implications of genetic elements (coding and non-coding genes) at an unprecedented level of resolution in GAS disease-relevant environments.

## Methods

### Bacterial strains and media

Bacterial strains used in this study are shown in **Table S8**. 5448^72^ is a GAS clinical isolate representative of the globally disseminated invasive serotype M1T1 clone. NZ131^73^ is a GAS strain isolated from a patient with Acute Post-Streptococcal Glomerulonephritis (APSGN). GAS strains were routinely cultured in Todd-Hewitt medium (Alpha Biosciences) supplemented with 0.2% yeast extract (THY) as described elsewhere^74^. *Escherichia coli* strains DH5α^75^ and C43[DE3]^76^ were used as hosts for plasmid construction and preparation and were cultured in Luria-Bertani (LB) medium (EMD Chemicals). Antibiotics (Fischer Scientific; Gold Biotechnology) were used at the following concentrations: Ampicillin (Ap) at 100 μg/ml for *E. coli*, Spectinomycin (Sp) at 100 μg/ml for both *E. coli* and GAS, and Kanamycin (Km) at 50 μg/ml for *E. coli* and 300 μg/ml for GAS.

### Molecular genetics

Oligonucleotides used in this study were synthesized by Integrated DNA Technologies, Inc. and are listed in **Table S9**. Plasmids used in this study are shown in **Table S8**. Plasmids were isolated using the Wizard Plus SV Minipreps kit (Promega) or the QIAGEN Plasmid Purification Midi Kit (QIAGEN). Restriction enzymes, Antarctic Phosphatase and T4 DNA ligase (New England Biolabs) were used according to the manufacturer’s instructions. PCR was performed using either *Taq* DNA polymerase (New England Biolabs) or High-Fidelity AccuPrime *Pfx* DNA polymerase (Life Technologies) with 1 μg of DNA template and 10 pmol of the appropriate primers (**Table S9**). When necessary, PCR products were purified using Wizard SV Gel and PCR Clean-Up System kit (Promega). Transformations were performed with the Gene Pulser Xcell System apparatus (Bio-Rad) as recommended by the manufacturer, using electrocompetent cells of *E. coli* or GAS prepared as described by Ausubel *et al*.^77^ or Le Breton and McIver^28^, respectively. Genomic DNA (gDNA) from GAS was purified using the MasterPure Complete DNA Purification kit (Epicentre Biotechnologies). Genewiz, Inc performed the Sanger DNA sequencing.

### Generation of the *Krmit* *mariner* transposon

The pOSKAR *mariner* delivery system^29^ was modified for Tn-seq analyses as follows: an *Mme*I restriction site was introduced into both inverted terminal repeat (ITR) sequences of *Oskar* by PCR using pOSKAR (**Table S8**) and the primer oKrmit1 (**Table S9**) to generate the *Krmit* transposon (**Fig. S1A**). The resulting *Krmit* PCR product was digested with *Pst*I and used to replace *Oskar* in *Pst*I-digested pOSKAR and generate the *mariner* delivery plasmid pKRMIT (**Fig. S1B, Table S8**). Like pOSKAR^28^,^29^, pKRMIT is unstable in *E. coli* and the use of C43[DE3]^76^ was required for efficient amplification.

### Generation of *Krmit* mutant libraries in GAS

*In vivo* transposition of *Krmit* for random mutagenesis in GAS was accomplished as described for pOSKAR^28^,^29^ with some modifications. GAS 5448 and NZ131 were transformed with 300 μg and 50 μg pKRMIT, respectively, and allowed to outgrow in THY broth at 30°C for 4 h. Transformants were plated on THY agar containing Km and Sp, and then incubated at 30°C for 48 h. Naturally occurring kanamycin resistance has not been observed in GAS 5448 or NZ131. The presence of intact pKRMIT was tested as previously described^28^ and proper GAS transformants were stored at −80°C. For *Krmit* transposition, an individual GAS (pKRMIT) freezer stock was used to inoculate 250 ml THY containing Km for overnight growth at 37°C (T_0_). The quality of *Krmit* transposition was tested as previously described^28^. The complexity or randomness of *Krmit* mutant libraries was assessed by amplifying the insertion sites of random mutants by AP-PCR^28^,^29^, Sanger DNA sequencing, and mapping to their appropriate GAS genome. Percent randomness was determined by defining a ratio of unique insertions (by AP-PCR sequencing) among a tested population.

### Tn-seq analyses of GAS following growth in THY

A 10 ml aliquot of *Krmit* mutant libraries in either GAS 5448 or NZ131 (T_0_) was grown overnight in 250 ml THY containing Km at 37°C (T_1_). 10 ml of the resulting T_1 _culture was inoculated in 250 ml THY containing Km and incubated overnight at 37°C a second (T_2_) and third (T_3_) time. At each time point, 10 ml aliquots were collected to harvest cells by centrifugation for subsequent gDNA isolation.

Tn-seq was performed as originally described by van Opijnen *et al*.^31^ with some modifications: GAS gDNA was isolated from *Krmit* mutant pools and 5 µg was subjected to complete digestion with *Mme*I, treated with Antarctic Phosphatase (NEB), and purified by phenol/chloroform extraction. The *Mme*I-digested gDNA fragments were then ligated to an *Mme*I adapter. Eight different *Mme*I adapters (Adapter-501 to Adapter-508) were generated by annealing oligonucleotide pairs (**Table S9**) containing Illumina barcode sequences (501 to 508) to allow sample multiplexing during massively parallel sequencing. The ligation mixture was then used as a template for a 20-cycle PCR with the primers oKrmit-Tnseq2 and oAdapterPCR (**Table S9**), resulting in the production of 176-bp *Krmit* insertion tags (**Fig. S2**) that were purified from a 2% agarose gel. Quality and yield of the resulting tags was assessed using both a NanoDrop 8000 spectrophotometer (Thermo Scientific) and a Bioanalyzer (Agilent).

Libraries of *Krmit* insertion tags were analyzed by massively parallel sequencing (50-nt single end reads) on an Illumina HiSeq 1500 platform in the Institute for Bioscience and Biotechnology Research (IBBR) Sequencing Facility located at the University of Maryland, College Park. The detailed procedures for analysis of the Tn-seq data are presented in **Text S1**. Succinctly, the quality of read datasets (Sanger FastQ format) was determined using FastQC^78^, data filtered and trimmed using Biopieces (biopieces.org) to select for reads containing the Tn-seq barcodes and *Krmit* ITR end. Reads were then de-multiplexed and count tables generated using SamTools^79^ and HTseq^80^. Reads were mapped to the GAS MGAS5005 (for 5448) or NZ131 genome using Bowtie^81^ and data visualized using the Integrative Genomics Viewer (IGV) browser (broadinstitute.org/igv/home)^35^,^36^. Gene essentiality was determined using a Bayesian statistical model based on the Metropolis-Hastings algorithm using the Python script (saclab.tamu.edu/essentiality) developed by DeJesus *et al*.^37^.

### Determination of the GAS "core" genome

Twenty GAS whole genome sequences publicly available at the time of this study (*i.e*. MGAS5005, NZ131, SF370, MGAS8232, MGAS315, MGAS10394, MGAS6180, MGAS9429, MGAS10270, MGAS2096, MGAS10750, MGAS15252, MGAS1882, Alab49, A20, M1-476, HSC5, HKU, Manfredo and SSI-1; **Table S10**) were subjected to whole genome multiple sequence alignment using the Mugsy software^82^ with default parameters. Clusters of syntenic orthologs were generated using the Mugsy-Annotator software^83^ based on the annotated genes from each genome and using synteny information derived from the Mugsy alignment. Core clusters were then selected by requiring that each cluster contain at least one gene from each of the 20 genomes analyzed.

### Validation of gene essentiality by riboswitch-inducible expression

Mutants in two selected genes identified as essential in the Tn-seq screen (*murE, vicR*) were constructed in order to have their mRNA translation under control of a synthetic theophylline-dependent riboswitch developed by Topp *et al*.^84^ as follows. First, a new mutagenesis system pSin/pHlp was created for stable plasmid integration into the GAS chromosome comprised of: (i) a pSinS suicide plasmid unable to independently propagate in GAS derived by PCR from the pCRS plasmid backbone^29^ using the primers RepAminus1 and RepAminus2 to delete a portion of the *repA*^ts^ gene, (ii) a pHlpK helper plasmid possessing a thermosensitive pWV01 replicon and constructed by PCR from the pCRK backbone^29^ using the primers KmR1NotI and OTS2 to delete pCRK’s multiple cloning site and its ColE1 origin of replication. The mutagenic plasmid was obtained by cloning a DNA fragment into the pSinS vector produced by SOE-PCR to fuse the P*_sag_* promoter along with the synthetic riboswitch E contained on pEU7742-E to the 5’-end (*ca*. 600 bp) of the target gene (Fig. 5A, Tables S8 and S9). The construct was then introduced into pHlpK-containing GAS cells and transformants selected in the presence of Km and Sp at permissive temperature (30°C) to allow replication of the two plasmids. Chromosomal integration of the mutagenic plasmid was achieved by culturing the clone at non-permissive temperature (37°C) in the presence of 2 mM theophylline. A merodiploid GAS mutant was produced in which the wild type gene had its expression controlled by the theophylline-inducible riboswitch, while the wild-type promoter of the targeted gene controlled the expression of a truncated allele (Fig. 5A). The growth of the integrative mutant in THY in the presence or absence of theophylline was monitored by measuring the culture turbidity using a Klett-Summerson colorimeter equipped with an A filter (Klett Units) or an automated BMG FLUOstar Omega microplate spectrophotometer (OD_600_ nm).

### Accession number for public deposition of Tn-seq data

Illumnia sequencing reads from the Tn-seq analyses were deposited in the NCBI Sequence Read Archive (SRA) under the accession number (PRJNA280537).

## Author Contributions

Y.L.B. and K.S.M. conceived and designed the research plan, supervised the project, analyzed the data and interpreted the results. Y.L.B. performed most of the experiments with technical contributions from K.M.V. (pKRMIT design), E.I. (construction of *Krmit* libraries, pSin/pHlp system and inducible mutants) and P.C. (AP-PCRs for NZ131 *Krmit* mutants). A.T.B. and N.M.E. performed the bioinformatics analyses. H.T. determined the GAS core genome. M.E.S. provided intellectual input and guidance. Y.L.B., A.T.B., H.T., N.M.E. and K.S.M. wrote the manuscript. All authors read and approved the final manuscript.

## Additional information

**Supplementary information** accompanies this paper at http://www.nature.com/scientificreports

**How to cite this article:** Le Breton, Y. *et al*. Essential Genes in the Core Genome of the Human Pathogen *Streptococcus pyogenes. Sci. Rep.* 5, 9838; DOI:10.1038/srep09838 (2015).

## Supplementary Material

Supplementary Information Supplementary Information
