# Supplementary material for: Essential Genes in the Core Genome of the Human Pathogen Streptococcus pyogenes
Source: Sci Rep. 2015 May 21;5:9838. doi: 10.1038/srep09838 (PMC4440532; doi:10.1038/srep09838)
Supplement: Supplementary Information [file srep09838-s1.pdf]

Supplemental Materials for:

Essential Genes in the Core Genome of the Human Pathogen *Streptococcus pyogenes*

Yoann Le Breton[1], Ashton T. Belew[1], Kayla M. Valdes[1], Emrul Islam[1], Patrick Curry[1], Hervé Tettelin[3, 4], Mark. E. Shirtliff [4, 5], Najib M. El-Sayed[1, 2], and Kevin S. McIver[1]

[a]Department of Cell Biology & Molecular Genetics and Maryland Pathogen Research Institute, [b]Center for Bioinformatics and Computation Biology, University of Maryland, College Park, MD 20742; [3]Institute for Genome Sciences, [4]Department of Microbiology & Immunology, [5]Department of Microbial Pathogenesis, School of Dentistry, University of Maryland School of Medicine, Baltimore, MD USA

Supplemental Materials Contents:

Supplemental Methods. Methodology of all computation analyses performed in the manuscript, including scripts used, genome alignments, library matrices and statistical analyses.

Supplemental Figures. Following four (4) figures are included:

Figure S1. pKRMIT, a Tn-seq compatible *in vivo mariner* transposition system for GAS.

Figure S2. Additional statistical analyses for pairwise comparisons of the initial libraries produced in GAS 5448.

Figure S3. The GAS core genome.

Figure S4. Conserved essential genes within the GAS core genome.

Supplemental Tables. Following ten (10) tables are included:

Table S1. Complete analysis of Tn-seq read counts and alignments.

Table S2. Selection of reads for the Bayesian analysis.

Table S3. Bayesian analysis of Tn-seq datasets for GAS M1T1 5448 grown *in vitro*.

Table S4. Integrated gene essentiality determination for GAS M1T1 5448 for all time points.

Table S5. Bayesian analysis of Tn-seq datasets for GAS M49 NZ131 grown *in vitro*.

Table S6. Integrated gene essentiality determination for GAS M49 NZ131 for all time points.

Table S7. Conserved GAS essential genes identified in both M1T1 5448 and M49 NZ131 compared to the predicted GAS core genome.

Table S8. Bacterial strains and plasmids.

Table S9. Primers used in this study.

Table S10. Summary of 10 publically available GAS genomes.

Supplemental Methods

Scripts used

Italicized directory names (ending in /) refer to directories within the git repository for this project, italicized scripts are provided in the *scripts/* directory, and italicized data files reside in the *data/* directory. The git repository may be downloaded by visiting *https://gitlab.umiacs.umd.edu/abelew/tnseq_gas* or with the command:

git clone https://gitlab.umiacs.umd.edu/abelew/tnseq_gas.git .

Annotation Collection.

GenBank genomes including annotation information were downloaded for each of the GAS strains used in this study: CP000017 is the reference MGAS 5005 genome [1] used for MGAS 5448 alignments as it is the most similar annotated genome available; NC_011375 is the reference genome for NZ131 [2]. A genbank file for the plasmid was also parsed (*reference/genbank/pOSKAR.gb*) and used to define how many reads did not integrate into the genome properly. For each of the genbank annotations collected, the perl script *gb2gff.pl* was used to split the genbank file into the following: a nucleotide fasta file containing a single genomic sequence, a nucleotide multi-fasta of all annotated coding sequences, a peptide multi-fasta of annotated coding sequences, a gff file of all annotations, a gff file of only the coding sequence annotations, and a gff file describing the inter-cds regions. These may be found in *reference/genbank/*. The resulting fasta and gff files were used as the reference for most of the operations that follow; conversely, gene ontology information was downloaded from microbesonline.org for strains 5448

and NZ131 and cross referenced against these annotations when necessary. Copies are located in *reference/microbesonline/*.

Genome Alignments.

The reads generated from Tn-seq data produced in GAS 5448 and NZ131 were queried for quality with FastQC [3] before demultiplexing against the local indexing adapters with Bio::SeqIO [4] and again queried for quality. The *mariner* ITR leading sequences were removed with a combination of Biopieces (biopieces.org), Cutadapt [5], and Trimmomatic [6], reads lacking the ITR sequence were discarded. This process left libraries of approximately 15,000,000 reads (**Table S1)** of lengths primarily 12<=x<=15 nucleotides. In the cases where are large number of reads did not align to the genome, the primary culprit was invariably the plasmid (**Table S1).**

These libraries were aligned against the appropriate GAS genomes with Bowtie [7,8] using options to limit the seed sequence to 10 nucleotides (-l 10), with no mismatch (-v 0), and randomly assign multi-matched reads to one of the possible matching positions (-M 1); these are summarized in **Table S1.** This was later repeated with 1 mismatch. The resulting alignments were converted to sorted/compressed binary alignments using SAMtools [9] and counted [10] against the reference genome CDS and intergenic regions. These operations were performed with the *process_reads.sh* script. The resulting count tables are in *data/count_tables/*; organized by strain, library, and time point; they are referenced in *all_samples.csv* for all operations in R. When R functions are noted, they are italicized and have following parentheses. These functions will be found in *R/myr.R* or rarely in *tnseq.rmd*. All operations performed in R are recorded in *tnseq.rmd*

(tnseqv0.rmd for no-mismatches, and tnseqv1.rmd for 1 mismatch analyses) and knitr (http://yihui.name/knitr/) was used to generate *tnseq.pdf* which provides a (longer than anyone will ever read) report of the operations performed.

Library Metrics

Before using the libraries to quantify the essentiality/fitness of coding sequences in the genome, it was necessary to compare and contrast the libraries and quantify their relative coverage, similarity, and saturation with respect to available TA insertion points. These comparisons in turn require some attention to the normalization strategies employed.

Library Saturation by TA insertion points. The GAS genome is AT rich, providing a relatively large number of possible *mariner* insertion points: there are 1,838,562 and 1,815,783 TA sites found on the genome of MGAS5005 and NZ131, respectively. Therefore, the bam alignments were read into *tnseq_count_hitsv2.pl* along with the fasta genome; this script created a simple array of the genome and filled in the number of observed reads, which started (forward strand) or ended (reverse strand) at every TA in the genome. The bowtie parameters used allowed no mismatch, therefore this script excluded any read which did not match exactly the TA insertion point. The resulting text files contain one line for each TA of the genome, include a column for the position of each TA, the number of observed reads on the forward strand, reverse strand, and missed reads. These may be found in *data/count_tas/*; there is one file for the concatenated 5448 libraries as well as each separate library for all strains, in each case the file ends in '*tas_only.txt*'. Some reads were observed (again due to bowtie mismatches)

to hit near but not on the TA insertion points. These were also collected in the *tnseqcount.out* files, but were discarded. These same outputs were used to feed the essentiality package [11].

These files were read into the R function *plot_saturation()* and used to visualize the lifespan of each library over time. This was done by taking the $\log_2$(hits + 1) for each position and plotting them as a set of histograms.

Normalization and visualization strategies. The most expedient method of comparing the libraries was to treat them as if they were components of an RNA sequencing experiment and assuming similar normalization strategies [12] apply. This strategy is very similar to that taken by the essentials software package [13], but uses voom/limma instead of EdgeR. Therefore the alignments were counted by reference genome coding sequence and quantile normalized without removing the low-count genes. These normalized counts were adjusted by the size of the library and length of each coding sequence (rpkm implemented in edgeR [14] with assistance from DESeq [15] and $\log_2$ transformed. Conversely, the quantile normalized counts were adjusted by library size (cpm), then adjusted by the ratio of TAs observed in the coding sequence divided by the median number of TAs in all coding sequences (all normalizations were performed by *my_norm* and *divide_seq*). The resulting data sets were used to quantify the median coverage of the genome. Pairwise Euclidean distances (Fig. S2B), Spearman correlation coefficients (Fig. 1G, H, I) and principle component analyses (Fig. S2A) were then used to visualize the similarities/differences between normalized libraries.

Pairwise sample comparisons. In order to further explore the similarities and difference between the libraries, they were plotted against one another as a series of

scatter plots (generated by *my_linear_scatter*) and histograms. These provided a visual metric of the similarities and differences among the libraries and time points between libraries. In the case of the 5448 strain, voom [16] and limma [17] were used to combine the samples, correct for the batch effect among libraries, and calculate the merged changes between time points (performed by *simple_comparison*), and include significance with respect to the variance in the data.

Essentiality. The essentiality software package [11] provides an opportunity to query statistically significant stretches of TAs which have no observed insertions to further inform its metric of essentiality. The insertion data was therefore converted into its expected format (*tnseq_count_hitsv2.pl*) and passed to the version 1.21 of the implementation python script. The resulting table provided a count of the number of insertions observed in each ORF, the number of observed TAs, the maximum length of non-observed sequence, the nucleotide span of this region, a call on whether each ORF is essential, and the posterior probability for each call. A few different parameters were tested with each strain and settled on a minimum hit parameter of 2 for both strains.

Circos. R was used to massage various data structures into the format expected by circos [18].

SUPPLEMENTAL REFERENCES

1 Sumby, P. *et al.* Evolutionary origin and emergence of a highly successful clone of serotype M1 group A streptococcus involved multiple horizontal gene transfer events. *J. Infect. Dis.* **192**, 771-782 (2005).

2 McShan, W. M. *et al.* Genome sequence of a nephritogenic and highly transformable M49 strain of *Streptococcus pyogenes*. *J. Bacteriol.* **190**, 7773-7785, doi:10.1128/JB.00672-08 (2008).

3 Patel, R. K. & Jain, M. NGS QC Toolkit: A Toolkit for Quality Control of Next Generation Sequencing Data. *PloS One* **7**, e30619 (2012).

4 Stajich, J. E. *et al.* The Bioperl toolkit: Perl modules for the life sciences. *Genome Res.* **12**, 1611-1618 (2002).

5 Martin, M. Cutadapt removes adapter sequences from high-throughput sequencing reads. *EMBnet J* (2011).

6 Bolger, A. M., Lohse, M. & Usadel, B. Trimmomatic: a flexible trimmer for Illumina sequence data. *Bioinformatics* **30**, 2114-2120 (2014).

7 Langmead, B. Aligning short sequencing reads with Bowtie. *Current Protocols in Bioinformatics* **11** (2010).

8 Langmead, B., Trapnell, C., Pop, M. & Salzberg, S. L. Ultrafast and memory-efficient alignment of short DNA sequences to the human genome. *Genome Biology* **10**, R25 (2009).

9 Li, H. *et al.* The Sequence Alignment/Map format and SAMtools. *Bioinformatics* **25**, 2078-2079 (2009).

10 Anders, S. & Huber, W. Differential expression analysis for sequence count data. *Genome Biology* **11**, R106 (2010).

11 DeJesus, M. A. *et al.* Bayesian analysis of gene essentiality based on sequencing of transposon insertion libraries. *Bioinformatics* **29**, 695-703 (2013).

12 Dillies, M. A. *et al.* A comprehensive evaluation of normalization methods for Illumina high-throughput RNA sequencing data analysis. *Briefings in Bioinformatics* **14**, 671-683, doi:10.1093/bib/bbs046 (2013).

13 Zomer, A., Burghout, P., Bootsma, H. J., Hermans, P. W. & van Hijum, S. A. ESSENTIALS: software for rapid analysis of high throughput transposon insertion sequencing data. *PloS One* **7**, e43012, doi:10.1371/journal.pone.0043012 (2012).

14 McCarthy, D. J., Chen, Y. & Smyth, G. K. Differential expression analysis of multifactor RNA-Seq experiments with respect to biological variation. *Nucleic Acids Res.* **40**, 4288-4297, doi:10.1093/nar/gks042 (2012).

15 Love, M. I., Huber, W. & Anders, S. Moderated estimation of fold change and dispersion for RNA-Seq data with DESeq2. *bioRxiv*, doi:doi:10.1101/002832 (2014).

16 Law, C. W., Chen, Y., Shi, W. & Smyth, G. K. voom: Precision weights unlock linear model analysis tools for RNA-seq read counts. *Genome Biology* **15**, R29, doi:10.1186/gb-2014-15-2-r29 (2014).

17 Qin, H., Feng, T., Harding, S. A., Tsai, C. J. & Zhang, S. An efficient method to identify differentially expressed genes in microarray experiments. *Bioinformatics* **24**, 1583-1589, doi:10.1093/bioinformatics/btn215 (2008).

18 Krzywinski, M. *et al.* Circos: an information aesthetic for comparative genomics. *Genome Res.* **19**, 1639-1645, doi:10.1101/gr.092759.109 (2009).

Supplemental Figures

Supplemental Figure S1. pKRMIT, a Tn-seq compatible *in vivo mariner* transposition system for GAS. The *Krmit mariner* transposable element (A) contains a Kanamycin-resistance (Km^r) cassette surrounded by inverse terminal repeats (ITR) targeted by the *Himar1 mariner* transposase. *Mme*I restriction sites were introduced in the ITR for Tn-seq insertion tag production as indicated. Promoter elements and RBS are also shown. (B) The pKRMIT *E. coli*/GAS shuttle vector possesses a hyperactive allele of the *Himar1 mariner* transposase (*tnpC9*) driven from the *L. lactis* P_{23} promoter, a ColE1 origin of replication functional in *E. coli*, a pWV01-derived thermosensitive origin of replication (oriRts) functional in GAS, and a spectinomycin resistance element (Sp^R). (C) Web logo consensus analysis of *Krmit* transposon insertion sites (TIS) in 120 independent transposants produced in GAS 5448. Nucleotide sequences at the site of *Krmit* insertion are displayed along the horizontal axis. Positions 31 and 32 correspond to the "TA" dinucleotide targeted by *Krmit*. The height and order of each letter in the stack of letters at each position correspond to the relative frequency of the nucleotides at that position.

Supplemental Figure S2. Additional statistical analyses for pairwise comparisons of the initial libraries produced in GAS 5448. Libraries 1 to 4 are presented by their original names in the study (Library 9, 11, 12 and 34, respectively) and compared using two additional methods. (A) Principal Component Analysis (PCA) was performed and

the results are presented graphically with PC1 (x axis) and PC2 (y axis). Libraries are color coded based on time points as follows; T_0 (yellow), T_1 (green), T_2 (red), and T_3 (blue). (B) Euclidean Distance statistical analysis is presented as a heat map.

Supplemental Figure S3. The GAS core genome. Artemis ACT [83] graphical representation of the whole genome multiple sequence alignment of 20 publicly available GAS strains using core clusters of syntenic orthologs (pink lines) as the linking information. Genome order from top to bottom: MGAS5005, NZ131, SF370, MGAS8232, MGAS315, MGAS10394, MGAS6180, MGAS9429, MGAS10270, MGAS2096, MGAS10750. MGAS15252, MGAS1882, Alab49, A20, M1-476, HSC5, HKU, Manfredo and SSI-1.

Supplemental Figure S4. Conserved essential genes within the GAS core genome. Artemis ACT [83] graphical representation of the whole genome multiple sequence alignment of 20 publicly available GAS strains using clusters of essential syntenic orthologs (red lines) as the linking information. Genome order from top to bottom: MGAS5005, NZ131, SF370, MGAS8232, MGAS315, MGAS10394, MGAS6180, MGAS9429, MGAS10270, MGAS2096, MGAS10750. MGAS15252, MGAS1882, Alab49, A20, M1-476, HSC5, HKU, Manfredo and SSI-1.

Figure S1

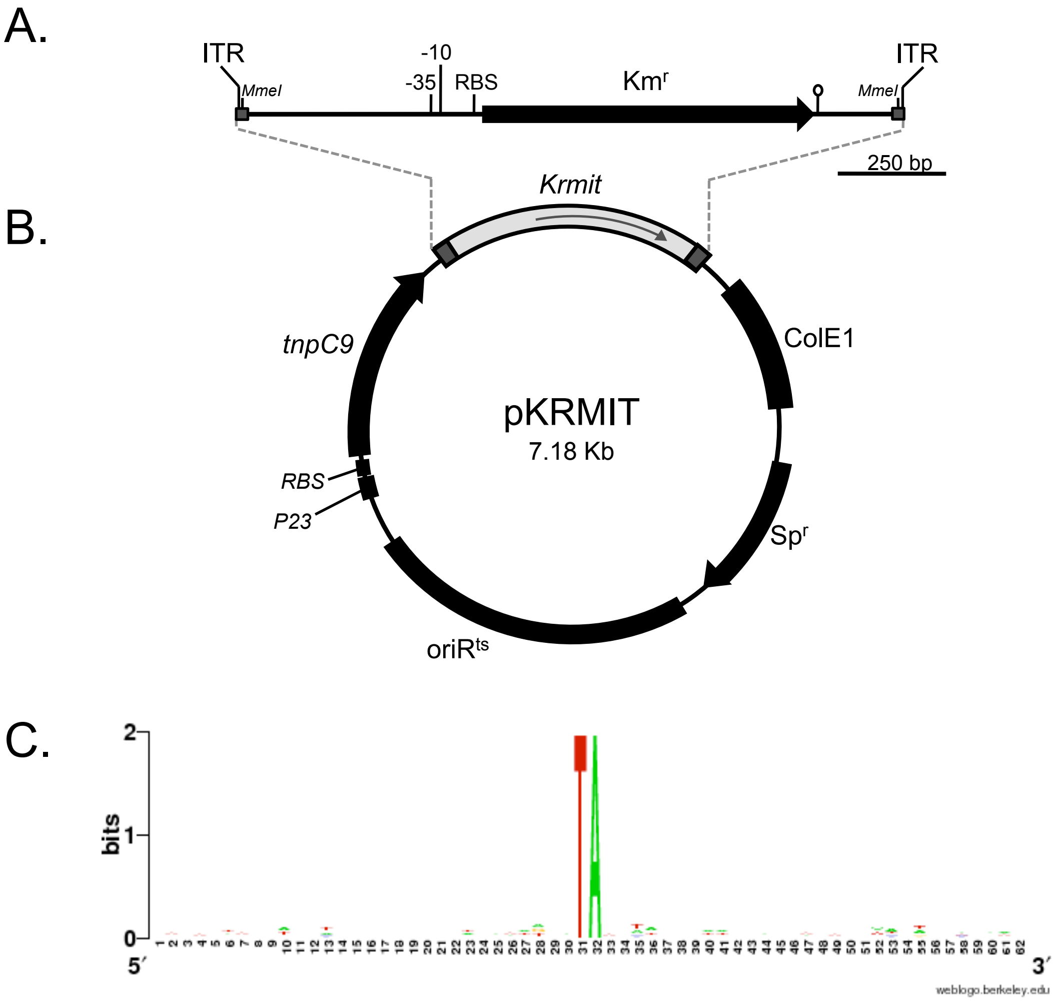

A.
ITR
MmeI
-10
-35
RBS
Km^r
ITR
MmeI
250 bp
B.
Krmit
tnpC9
ColE1
pKRMIT
7.18 Kb
RBS
P23
Sp^r
oriR^ts
C.
2
bits
1
0
5′
3′
weblogo.berkeley.edu

Figure S2

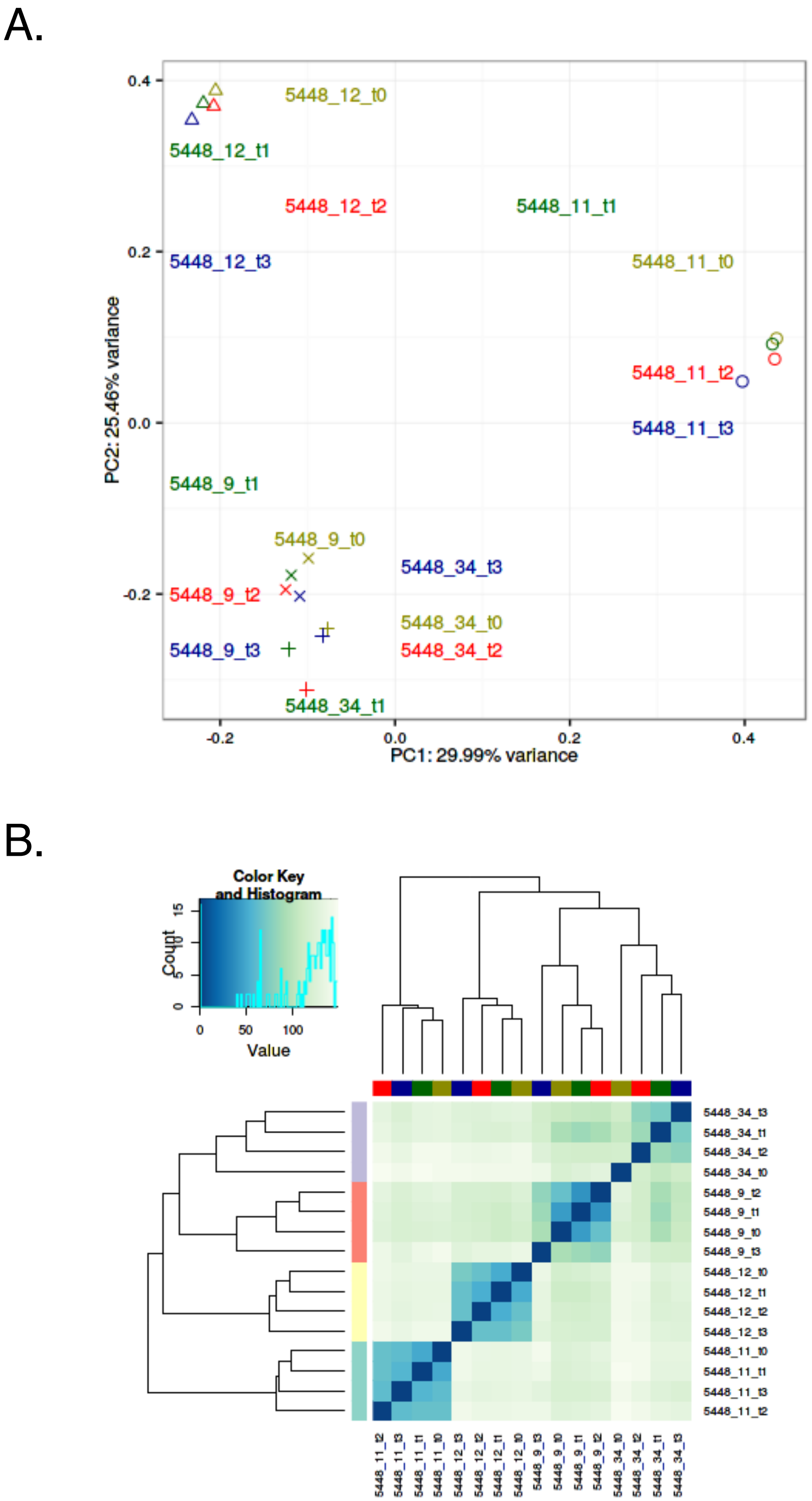

A.
PC2: 25.46% variance
PC1: 29.99% variance
0.4
0.2
0.0
-0.2
5448_12_t0
5448_12_t1
5448_12_t2
5448_12_t3
5448_11_t1
5448_11_t0
5448_11_t2
5448_11_t3
5448_9_t1
5448_9_t0
5448_9_t2
5448_9_t3
5448_34_t3
5448_34_t0
5448_34_t2
5448_34_t1
B.
Color Key and Histogram
Count
0
5
10
15
0
50
100
Value
5448_34_t3
5448_34_t1
5448_34_t2
5448_34_t0
5448_9_t2
5448_9_t1
5448_9_t0
5448_9_t3
5448_12_t0
5448_12_t1
5448_12_t2
5448_12_t3
5448_11_t0
5448_11_t1
5448_11_t3
5448_11_t2

Figure S3

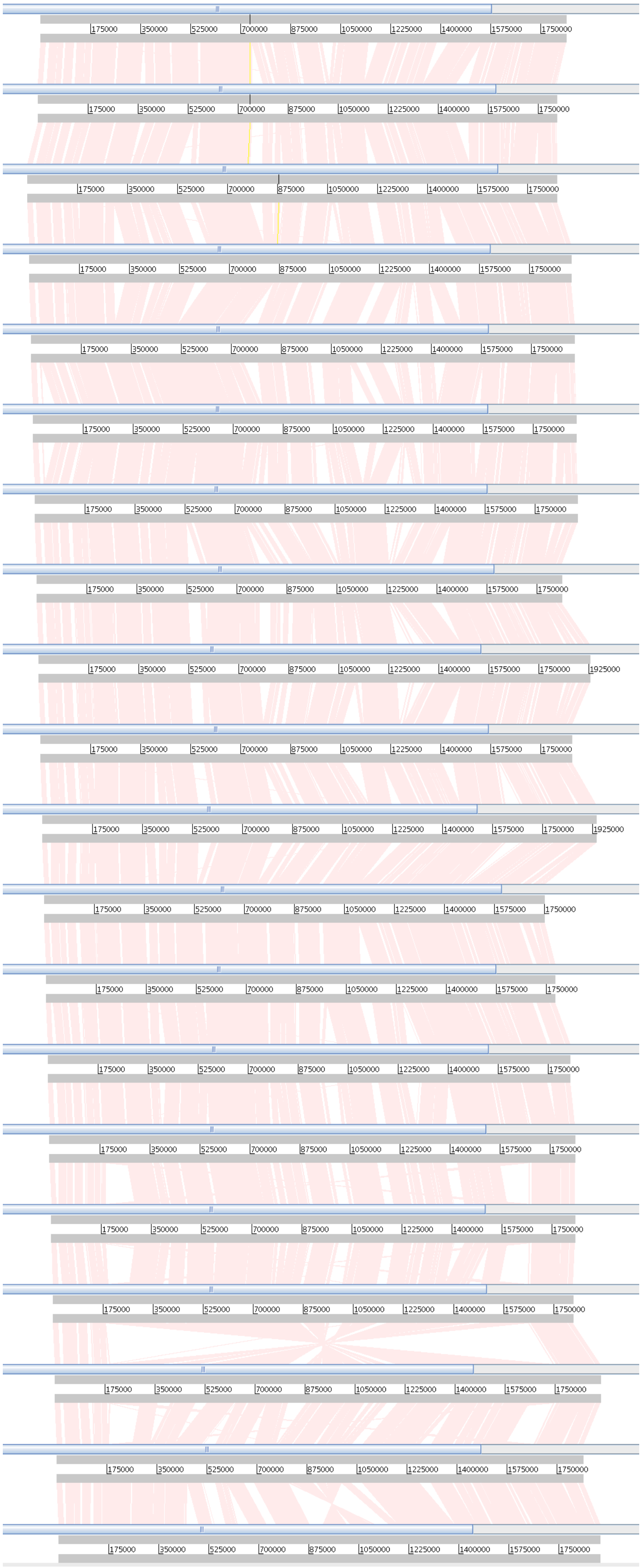

Figure S4

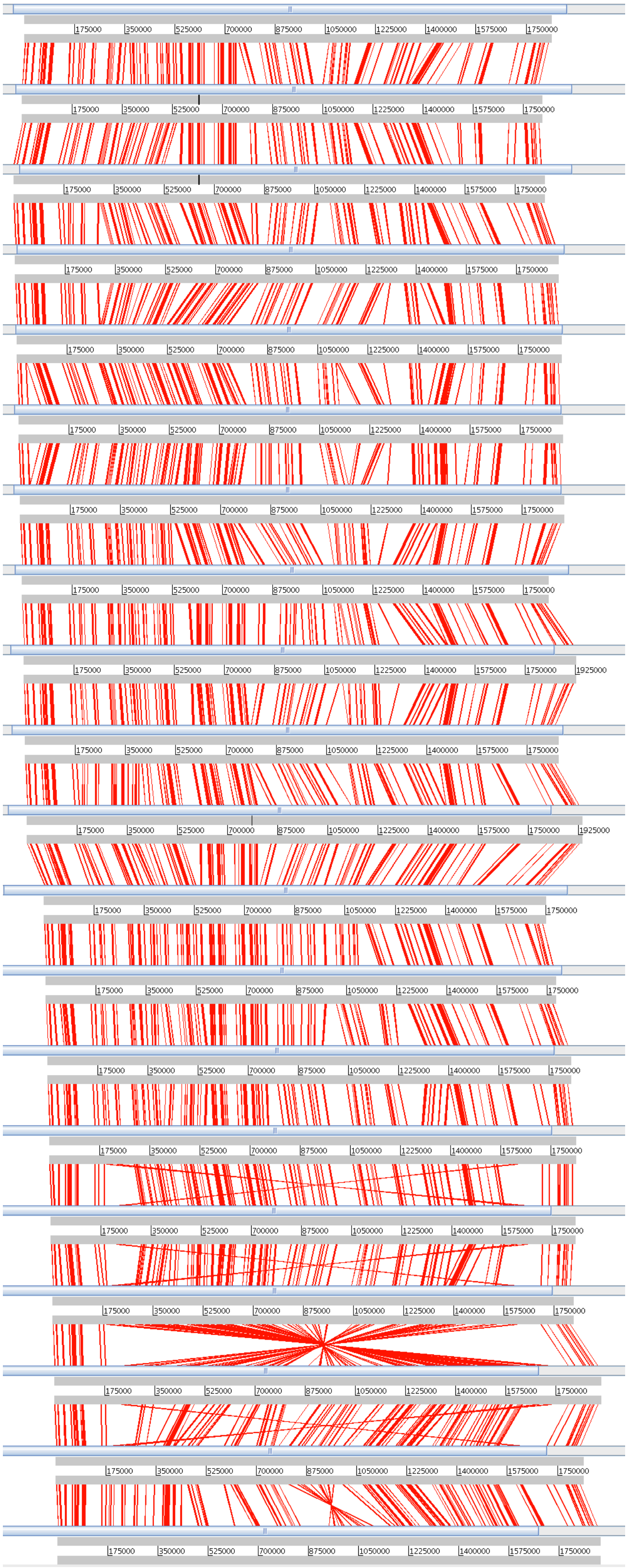
175000
350000
525000
700000
875000
1050000
1225000
1400000
1575000
1750000
1925000

Supplemental Tables

Following nine (9) tables are presented:

**Table S1.**	Complete analysis of Tn-seq read counts and alignments.
**Table S2.**	Selection of reads for the Bayesian analysis.
**Table S3.**	Bayesian analysis of Tn-seq datasets for GAS M1T1 5448 grown *in vitro*.
**Table S4.**	Integrated gene essentiality determination for GAS M1T1 5448 for all time points.
**Table S5.**	Bayesian analysis of Tn-seq datasets for GAS M49 NZ131 grown *in vitro*.
**Table S6.**	Integrated gene essentiality determination for GAS M49 NZ131 for all time points.
**Table S7.**	Conserved GAS essential genes identified in both M1T1 5448 and M49 NZ131 compared to the predicted GAS core genome.
**Table S8.**	Bacterial strains and plasmids.
**Table S9.**	Primers used in this study.
**Table S10.**	Summary of the 20 publically available GAS genome sequences.

Table S1. Complete analysis of Tn-seq read counts and alignments.

Sample(a)	Total reads	Strict aligned	Randomly placed(b)	Total aligned (%)(c)	Failed	Plasmid hits (%)(d)	Unique insert sites (TIS)	Saturation index(e)	Av. Distance (f)
5448 Lb1 T0	12,326,943	3,366,045	127,359	3,493,404 (28%)	8,833,539	8,597,058 (97%)	54,757	41	34
5448 Lb1 T1	15,691,922	13,870,884	499,569	14,370,453 (92%)	1,321,469	631,064 (48%)	90,292	68	20
5448 Lb1 T2	14,173,628	11,449,398	1,338,258	12,787,656 (90%)	1,385,972	871,696 (63%)	72,114	54	25
5448 Lb1 T3	12,692,504	11,471,765	632,320	12,104,085 (95%)	588,419	239,739 (41%)	20,132	15	91
5448 Lb2 T0	14,893,811	10,081,697	238,856	10,320,553 (69%)	4,573,258	4,101,079 (90%)	28,705	22	64
5448 Lb2 T1	12,603,471	11,272,221	267,163	11,539,384 (92%)	1,064,087	687,446 (65%)	24,123	18	76
5448 Lb2 T2	10,583,515	9,904,226	198,478	10,102,704 (95%)	480,811	152,719 (32%)	22,297	17	82
5448 Lb2 T3	15,500,886	13,903,253	598,603	14,501,856 (94%)	999,030	437,253 (44%)	33,710	25	55
5448 Lb3 T0	13,394,016	9,867,412	1,192,267	11,059,679 (83%)	2,334,337	2,037,516 (87%)	23,428	18	78
5448 Lb3 T1	15,131,244	13,023,394	1,053,742	14,077,136 (93%)	1,054,108	551,455 (52%)	32,406	24	57
5448 Lb3 T2	13,337,250	11,576,167	1,101,352	12,677,519 (95%)	659,731	402,269 (61%)	24,694	19	74
5448 Lb3 T3	17,623,505	13,938,708	3,080,613	17,019,321 (97%)	604,184	64,548 (11%)	15,840	12	116
5448 Lb4 T0	11,679,250	679,170	32,819	711,989 (6%)	10,967,261	10,879,152 (99%)	8,645	6	213
5448 Lb4 T1	15,856,339	14,747,260	255,938	15,003,198 (95%)	853,141	446,419 (52%)	42,032	32	44
5448 Lb4 T2	10,873,096	10,490,140	58,750	10,548,890 (97%)	324,206	119,664 (37%)	15,401	12	119
5448 Lb4 T3	13,664,074	12,976,990	53,330	13,030,320 (5%)	633,754	401,089 (63%)	26,038	20	71
Master 5448 T0	52,294,020	23,994,324	1,591,301	25,585,625 (49%)	26,708,395	25,614,805 (96%)	79,622	60	23
Master 5448 T1	59,282,976	52,913,759	2,076,412	54,990,171 (93%)	4,292,805	2,316,384 (54%)	107,059	80	17
Master 5448 T2	48,967,489	43,419,931	2,696,838	46,116,769 (94%)	2,850,720	1,546,348 (54%)	90,768	68	20
Master 5448 T3	59,480,969	52,290,716	4,364,866	56,655,582 (95%)	2,825,387	1,142,629 (40%)	65,183	49	28
NZ131 T0	3,460,214	1,652,565	88,035	1,740,600 (50%)	1,719,614	1,579,596 (92%)	52,464	40	35
NZ131 T1	31,730,825	20,195,930	1,717,501	21,913,431 (69%)	9,817,394	7,636,932 (78%)	111,001	84	16
NZ131 T2	17,433,089	10,882,091	220,199	11,102,290 (64%)	6,330,799	5,390,105 (85%)	67,449	51	27
NZ131 T3	44,784,994	36,885,896	307,314	37,193,210 (83%)	7,591,784	4,164,076 (55%)	57,377	44	32

(a) Sample name describes the GAS strain (i.e GAS 5448 or NZ131) used to produce the Krmit library. For 5448, 4 independent libraries (Lb1 to Lb4) were analyzed separately.
The different libraries produced (T0) were subjected to 3 additional passages in THY (T1 to T3). Master 5448 is the combinaison of the reads from the 4 initial libraries in 5448.
(b) Reads aligning to more than one position on the chromosome were randomly placed onto the GAS chromosome.
(c) Percentage in parenthesis represents the proportion of the initial reads (sum of strictly aligned and randomly placed) that align to the GAS genome.
(d) Percentage in parenthesis coresponds to the proportion of reads among the reads that failed to align to the GAS genome that were aligned to the pKRMIT plasmid sequence.
(e) The saturation index depicts the proportion of unique TA sites found on the GAS chromosome hit at least once by Krmit.
(f) Calculated distance separating two adjacent TA sites where Krmit inserted.

Table S2. Selection of reads for the Bayesian analysis.

	All TIS(a)		TIS with at least 2 Krmit copies(b)	
**Library**	**Unique TIS**	**Saturation index(c)**	**Unique TIS**	**Saturation index(c)**
5448 (T0)	79,622	60	48,250	36
5448 (T1)	107,059	80	74,428	56
5448 (T2)	90,768	68	56,967	43
5448 (T3)	65,183	49	24,716	19
NZ131 (T0)	52,464	40	39,254	30
NZ131 (T1)	111,001	84	79,307	60
NZ131 (T2)	67,449	51	35,787	27
NZ131 (T3)	57,377	44	11,487	9

(a) Recapitulation of the total number of unique transposon insertion sites (TIS) and saturation indexes for the *Krmit* libraries produced in GAS 5448 and NZ131.

(b) The Bayesian analysis for gene essentiality prediction called for eliminating TIS with one *Krmit* insertion. The remaining reads have at least two *Krmit* counts.

(c) The saturation index depicts the proportion of unique TA sites found on the GAS chromosome hit at least once by *Krmit*.

Table S3. Bayesian analysis of Tn-seq datasets for GAS M1T1 5448 grown *in vitro*.

(a) Spy numbers from MGAS5005 genome.

(b) When available, gene name is provided.

					Bayesian for T_0		Bayesian for T_1		Bayesian for T_2	
**Locus Tag(a)**	**Gene Name(b)**	**Start**	**Stop**	**Length**	**Z score**	**Call**	**Z score**	**Call**	**Z score**	**Call**
M5005_Spy_0001	dnaA	202	1557	451	1	E	1	E	1	E
M5005_Spy_0002	dnaN	1712	2848	378	1	E	1	E	1	E
M5005_Spy_0003	-	2923	3120	65	0	NE	0	NE	0	NE
M5005_Spy_0004	ychF	3450	4565	371	0	NE	0	NE	0	NE
M5005_Spy_0005	pth	4635	5204	189	0.99875	E	0.99975	E	0.98	U
M5005_Spy_0006	trcF	5207	8710	1167	0.00025	NE	0	NE	0	NE
M5005_Spy_0007	-	8872	9144	90	0	NE	0	NE	0	NE
M5005_Spy_0008	divIC	9131	9502	123	0.00025	NE	0.0015	NE	0.001	NE
M5005_Spy_0009	-	9499	9624	41	-1	S	-1	S	-1	S
M5005_Spy_0010	-	9637	10923	428	0	NE	0	NE	0	NE
M5005_Spy_0011	tilS/mesJ	10920	12206	428	1	E	1	E	1	E
M5005_Spy_0012	hpt	12211	12753	180	0.999	E	0.00025	NE	1	E
M5005_Spy_0013	ftsH	12775	14754	659	0.999	E	1	E	1	E
M5005_Spy_0014	-	15079	16470	463	0	NE	0	NE	0	NE
M5005_Spy_0015c	-	16811	16945	44	-1	S	-1	S	-1	S
M5005_Spy_0017	sibA	31134	32330	398	1	E	1	E	1	E
M5005_Spy_0018	prsA.2	32583	33545	320	0.98775	U	1	E	1	E
M5005_Spy_0019	recO	33731	34486	251	0	NE	0	NE	0	NE
M5005_Spy_0020	plsX	34589	35596	335	0.99875	E	1	E	1	E
M5005_Spy_0021	acpP.2	35589	35831	80	0.00025	NE	0.9235	U	0.222	U
M5005_Spy_0022	-	35952	36686	244	0.107	U	0	NE	0	NE
M5005_Spy_0023	-	36762	40535	1257	0.00025	NE	0	NE	0	NE
M5005_Spy_0024	purF	40696	42150	484	0	NE	0	NE	0	NE
M5005_Spy_0025	purM	42178	43200	340	0.00025	NE	0	NE	0	NE
M5005_Spy_0026	purN	43368	43922	184	0	NE	0	NE	0	NE
M5005_Spy_0027	purH	44106	45653	515	0	NE	0	NE	0	NE
M5005_Spy_0028c	-	45711	46835	374	0	NE	0	NE	0	NE
M5005_Spy_0029	purD	46998	48353	451	0	NE	0	NE	0	NE
M5005_Spy_0030	purE	48511	49122	203	0	NE	0	NE	0	NE
M5005_Spy_0031	purK	49106	50182	358	0.00175	NE	0	NE	0	NE
M5005_Spy_0032	-	50209	51852	547	0	NE	0	NE	0	NE
M5005_Spy_0033	purB	51870	53162	430	0.00025	NE	0	NE	0	NE
M5005_Spy_0034	-	53294	54205	303	0.00025	NE	0	NE	0	NE
M5005_Spy_0035	ruvB	54431	55429	332	0	NE	0	NE	0.002	NE
M5005_Spy_0036	-	55567	56004	145	0	NE	0	NE	0	NE
M5005_Spy_0037	-	56027	56428	133	0.00025	NE	1	E	0.002	NE
M5005_Spy_0038	-	56425	58200	591	0.40175	U	1	E	1	E
M5005_Spy_0039	adh2/adhE	58509	61151	880	0	NE	0	NE	0	NE
M5005_Spy_0040	adhA/adhP	61403	62419	338	0	NE	0	NE	0	NE
M5005_Spy_0041	norM	62807	63778	323	0	NE	0	NE	0	NE
M5005_Spy_0043	rpsJ	64301	64609	102	0.99	U	1	E	0.999	E
M5005_Spy_0044	rplC	64825	65451	208	0.9915	U	1	E	1	E
M5005_Spy_0045	rplD	65475	66098	207	1	E	1	E	1	E
M5005_Spy_0046	rplW	66098	66394	98	0.002	NE	0	NE	0	NE
M5005_Spy_0047	rplB	66412	67245	277	0.99875	E	1	E	1	E
M5005_Spy_0048	rpsS	67384	67662	92	0.992	U	0.00575	NE	0.999	E
M5005_Spy_0049	rplV	67678	68022	114	0.99825	E	1	E	1	E
M5005_Spy_0050	rpsC	68035	68688	217	0.993	U	1	E	1	E
M5005_Spy_0051	rplP	68692	69105	137	0.99925	E	1	E	1	E
M5005_Spy_0052	rpmC	69115	69321	68	0.90175	U	0	NE	0	NE
M5005_Spy_0053	rpsQ	69347	69607	86	0.99925	E	1	E	0.009	NE
M5005_Spy_0054	rplN	69632	70000	122	0.00025	NE	0.01675	NE	1	E
M5005_Spy_0055	rplX	70079	70384	101	0.001	NE	0.78675	U	0.024	NE
M5005_Spy_0056	rplE	70408	70950	180	1	E	0.70775	U	0.987	U
M5005_Spy_0057	rpsN	70966	71151	61	0.00975	NE	0.999	E	0.99	U
M5005_Spy_0058	rpsH	71302	71700	132	0.99875	E	0.02525	NE	0.001	NE
M5005_Spy_0059	rplF	71903	72439	178	1	E	1	E	1	E
M5005_Spy_0060	rplR	72544	72900	118	0.88275	U	1	E	0.991	U

M5005_Spy_0061	rpsE	72919	73413	164	0.9995	E	1	E	1	E
M5005_Spy_0062	rpmD	73428	73610	60	0.00025	NE	1	E	1	E
M5005_Spy_0063	rplO	73824	74264	146	0.00025	NE	0.00025	NE	0.001	NE
M5005_Spy_0064	secY	74281	75585	434	0.99975	E	1	E	1	E
M5005_Spy_0065	adk	75735	76373	212	0.00025	NE	1	E	0.08	U
M5005_Spy_0066	infA	76491	76709	72	0.99125	U	1	E	0.999	E
M5005_Spy_0067	rpmJ	76735	76851	38	-1	S	-1	S	-1	S
M5005_Spy_0068	rpsM	76869	77234	121	0.999	E	0.9995	E	0.999	E
M5005_Spy_0069	rpsK	77252	77635	127	0.001	NE	1	E	1	E
M5005_Spy_0070	rpoA	77681	78619	312	1	E	1	E	1	E
M5005_Spy_0071	rplQ	78634	79020	128	0.00025	NE	1	E	1	E
M5005_Spy_0072c	-	79618	79752	44	-1	S	-1	S	-1	S
M5005_Spy_0073	-	86264	86449	61	0	NE	0	NE	0.004	NE
M5005_Spy_0074	-	87087	87224	45	-1	S	-1	S	-1	S
M5005_Spy_0075	-	87235	87393	52	-1	S	-1	S	-1	S
M5005_Spy_0076	-	87499	87660	53	-1	S	-1	S	-1	S
M5005_Spy_0077	adcR	87770	88213	147	0	NE	0	NE	0	NE
M5005_Spy_0078	adcC	88217	88936	239	0	NE	0	NE	0	NE
M5005_Spy_0079	adcB	88929	89744	271	0	NE	0	NE	0	NE
M5005_Spy_0080c	-	89784	90167	127	0	NE	0	NE	0	NE
M5005_Spy_0081c	tyrS	90218	91474	418	1	E	0.99975	E	1	E
M5005_Spy_0082	pbp1b	91566	93878	770	0	NE	0	NE	0	NE
M5005_Spy_0083	rpoB	94142	97708	1188	1	E	1	E	1	E
M5005_Spy_0084	rpoC	97799	101440	1213	1	E	1	E	1	E
M5005_Spy_0085	-	101592	101957	121	0	NE	0	NE	0	NE
M5005_Spy_0086	comYA	102050	102988	312	0	NE	0	NE	0	NE
M5005_Spy_0087	comYB	102867	103958	363	0.00025	NE	0	NE	0	NE
M5005_Spy_0088	comYC	103960	104286	108	0	NE	0	NE	0	NE
M5005_Spy_0089	-	104360	104689	109	0	NE	0	NE	0	NE
M5005_Spy_0090	-	104646	104930	94	0	NE	0	NE	0	NE
M5005_Spy_0091	comYD	104923	105357	144	0	NE	0	NE	0	NE
M5005_Spy_0092	-	105341	105667	108	0	NE	0	NE	0	NE
M5005_Spy_0093	-	105765	106718	317	0	NE	0	NE	0	NE
M5005_Spy_0094	ackA	106777	107973	398	0.00025	NE	0	NE	0.001	NE
M5005_Spy_0095	-	108160	108468	102	0	NE	0	NE	0	NE
M5005_Spy_0096c	proC	108551	109321	256	0	NE	0	NE	0	NE
M5005_Spy_0097c	pepA	109369	110436	355	0	NE	0	NE	0	NE
M5005_Spy_0098	-	110546	110710	54	-1	S	-1	S	-1	S
M5005_Spy_0099	-	110892	111185	97	0	NE	0	NE	0	NE
M5005_Spy_0100	trx.1	111182	111499	105	0	NE	0	NE	0	NE
M5005_Spy_0101	-	111517	112143	208	0	NE	0	NE	0	NE
M5005_Spy_0102	ssb	112295	112690	131	0	NE	0	NE	0	NE
M5005_Spy_0103c	-	112950	113591	213	0	NE	0	NE	0	NE
M5005_Spy_0104c	-	113611	114588	325	0	NE	0	NE	0	NE
M5005_Spy_0105c	hslO	114575	115447	290	0	NE	0	NE	0	NE
M5005_Spy_0106c	rofA/nra	115594	117087	497	0	NE	0	NE	0	NE
M5005_Spy_0107	cbp	117335	119623	762	0	NE	0	NE	0	NE
M5005_Spy_0108	lepA-1	119616	120173	185	0	NE	0	NE	0	NE
M5005_Spy_0109	prtF	120206	121228	340	0	NE	0	NE	0	NE
M5005_Spy_0110	eftLSL.B	121238	121951	237	0	NE	0	NE	0	NE
M5005_Spy_0111	-	121973	122620	215	0	NE	0	NE	0	NE
M5005_Spy_0112c	-	122807	124129	440	0	NE	0	NE	0	NE
M5005_Spy_0113c	-	124370	124720	116	0	NE	0	NE	0	NE
M5005_Spy_0114	-	125024	125779	251	0	NE	0	NE	0	NE
M5005_Spy_0115c	-	125973	126638	221	0	NE	0	NE	0	NE
M5005_Spy_0116	atoE	126987	128393	468	0	NE	0	NE	0	NE
M5005_Spy_0117c	-	128461	128856	131	0	NE	0	NE	0	NE
M5005_Spy_0118c	-	128952	129371	139	0	NE	0	NE	0	NE
M5005_Spy_0119	-	129492	130676	394	0	NE	0	NE	0	NE
M5005_Spy_0120	atoD.2	130688	131347	219	0	NE	0	NE	0	NE
M5005_Spy_0121	-	131350	131997	215	0	NE	0	NE	0	NE
M5005_Spy_0122c	-	132119	132799	226	0	NE	0	NE	0	NE
M5005_Spy_0123	-	132973	133338	121	0	NE	0	NE	0	NE

M5005_Spy_0124	sloR	133375	134394	339	0	NE	0	NE	0	NE
M5005_Spy_0125	-	134849	135169	106	0	NE	0	NE	0	NE
M5005_Spy_0126	ntpI	135159	137180	673	0	NE	0	NE	0	NE
M5005_Spy_0127	ntpK	137182	137661	159	0.00025	NE	0	NE	0	NE
M5005_Spy_0128	ntpE	137729	138313	194	0	NE	0	NE	0	NE
M5005_Spy_0129	ntpC	138329	139327	332	0	NE	0	NE	0	NE
M5005_Spy_0130	ntpF	139324	139644	106	0	NE	0	NE	0	NE
M5005_Spy_0131	ntpA	139845	141620	591	0.00025	NE	0	NE	0	NE
M5005_Spy_0132	ntpB	141621	143036	471	0	NE	0	NE	0	NE
M5005_Spy_0133	ntpD	143081	143707	208	0	NE	0	NE	0	NE
M5005_Spy_0134c	-	143827	145089	420	0	NE	0	NE	0	NE
M5005_Spy_0135c	-	145102	145980	292	0	NE	0	NE	0	NE
M5005_Spy_0136	purA	146418	147710	430	0	NE	0	NE	0	NE
M5005_Spy_0137	-	148037	149080	347	0	NE	0	NE	0	NE
M5005_Spy_0138	nusG	149253	149792	179	0.79375	U	0.47925	U	0.999	E
M5005_Spy_0139	nga	150154	151518	454	0	NE	0	NE	0	NE
M5005_Spy_0140	-	151523	152008	161	0	NE	0	NE	0	NE
M5005_Spy_0141	slo	152032	153747	571	0	NE	0	NE	0	NE
M5005_Spy_0142	-	154002	154433	143	0	NE	0	NE	0	NE
M5005_Spy_0143c	-	154619	154855	78	0	NE	0	NE	0	NE
M5005_Spy_0144c	-	155265	155432	55	-1	S	-1	S	-1	S
M5005_Spy_0145c	-	155607	155894	95	0	NE	0	NE	0	NE
M5005_Spy_0146	metB	156335	157525	396	0	NE	0	NE	0	NE
M5005_Spy_0147	leuS	157736	160237	833	1	E	1	E	1	E
M5005_Spy_0148	ulaA	160544	161977	477	0	NE	0	NE	0.28	U
M5005_Spy_0149	-	162048	162326	92	0	NE	0	NE	0	NE
M5005_Spy_0150	-	162449	162934	161	0	NE	0	NE	0	NE
M5005_Spy_0151	ulaD	163025	163687	220	0.00025	NE	0	NE	0	NE
M5005_Spy_0152	-	163692	164555	287	0	NE	0	NE	0	NE
M5005_Spy_0153	araD	164557	165261	234	0	NE	0	NE	0	NE
M5005_Spy_0154	-	165319	165465	48	-1	S	-1	S	-1	S
M5005_Spy_0155	-	165586	167232	548	0	NE	0	NE	0	NE
M5005_Spy_0156	-	167485	168576	363	0	NE	0	NE	0	NE
M5005_Spy_0157	opuAA	169064	170260	398	0	NE	0	NE	0	NE
M5005_Spy_0158	opuABC	170276	172003	575	0	NE	0	NE	0.001	NE
M5005_Spy_0159	polA	172134	174776	880	0.0005	NE	0.999	E	1	E
M5005_Spy_0160	-	174963	175418	151	0	NE	0	NE	0	NE
M5005_Spy_0161	perR	175470	175937	155	0	NE	0	NE	0	NE
M5005_Spy_0162	vlg	176094	176393	99	0	NE	0	NE	0	NE
M5005_Spy_0163	-	176615	177949	444	0.00025	NE	0	NE	0	NE
M5005_Spy_0164	-	177942	178472	176	0	NE	0	NE	0	NE
M5005_Spy_0165c	-	178519	178761	80	0	NE	0	NE	0	NE
M5005_Spy_0166c	-	178801	179016	71	0	NE	0	NE	0	NE
M5005_Spy_0167c	-	179056	179625	189	0	NE	0	NE	0	NE
M5005_Spy_0168c	-	179658	179873	71	0	NE	0	NE	0	NE
M5005_Spy_0169c	-	180029	181339	436	0	NE	0	NE	0	NE
M5005_Spy_0170	nadC	181563	182435	290	0	NE	0	NE	0	NE
M5005_Spy_0171c	-	182735	183541	268	0	NE	0	NE	0	NE
M5005_Spy_0172c	-	183562	184077	171	0	NE	0	NE	0	NE
M5005_Spy_0173c	-	184163	185026	287	0	NE	0	NE	0	NE
M5005_Spy_0174	-	185063	185221	52	-1	S	-1	S	-1	S
M5005_Spy_0175	tgt	185245	186387	380	0	NE	0	NE	0	NE
M5005_Spy_0176	-	186604	186915	103	0.00025	NE	0	NE	0	NE
M5005_Spy_0177	bioY	186919	187458	179	0	NE	0	NE	0	NE
M5005_Spy_0178	-	187598	188377	259	0	NE	0	NE	0	NE
M5005_Spy_0179	-	188377	188892	171	0	NE	0	NE	0.001	NE
M5005_Spy_0180c	-	189506	190726	406	0	NE	0	NE	0	NE
M5005_Spy_0181	-	190780	190887	35	-1	S	-1	S	-1	S
M5005_Spy_0182	speG	191138	191842	234	0	NE	0	NE	0	NE
M5005_Spy_0183	-	191927	192046	39	-1	S	-1	S	-1	S
M5005_Spy_0184	-	192082	192201	39	-1	S	-1	S	-1	S
M5005_Spy_0185	pgi	192298	193647	449	1	E	1	E	1	E
M5005_Spy_0186c	ralp4	193996	195504	502	0	NE	0	NE	0	NE

M5005_Spy_0187	-	196059	196763	234	0	NE	0	NE	0	NE
M5005_Spy_0188	-	196666	197181	171	0	NE	0	NE	0	NE
M5005_Spy_0189	-	197165	197290	41	-1	S	-1	S	-1	S
M5005_Spy_0190	-	197371	197478	35	-1	S	-1	S	-1	S
M5005_Spy_0191	-	197506	198177	223	0	NE	0	NE	0	NE
M5005_Spy_0192c	hasC.2	198276	199175	299	0.00025	NE	1	E	0.002	NE
M5005_Spy_0194c	gpsA	199408	200223	271	1	E	1	E	1	E
M5005_Spy_0195	-	200521	200970	149	0	NE	0	NE	0	NE
M5005_Spy_0196	-	200963	202669	568	0	NE	0	NE	0.001	NE
M5005_Spy_0197	-	202672	204456	594	0.00025	NE	0	NE	0	NE
M5005_Spy_0198	-	204574	205341	255	0	NE	0	NE	0	NE
M5005_Spy_0199	dut	205451	205897	148	0.00025	NE	0	NE	0.001	NE
M5005_Spy_0200	radA	205978	207339	453	0.00025	NE	0	NE	0	NE
M5005_Spy_0201	-	207528	208025	165	0	NE	0	NE	1	E
M5005_Spy_0202	-	208156	208866	236	0	NE	0	NE	0	NE
M5005_Spy_0203	gltX	209048	210493	481	0.98775	U	1	E	1	E
M5005_Spy_0204	fasB	210888	212234	448	0	NE	0	NE	0	NE
M5005_Spy_0205	fasC	212231	213514	427	0.00025	NE	0	NE	0	NE
M5005_Spy_0206	fasA	213518	214258	246	0	NE	0	NE	0	NE
M5005_Spy_0207	rnpA	214798	215157	119	0.15675	U	0.99	U	1	E
M5005_Spy_0208	-	215141	215950	269	0	NE	0	NE	0	NE
M5005_Spy_0209	-	215962	216876	304	0	NE	0	NE	0	NE
M5005_Spy_0210	-	216961	217083	40	-1	S	-1	S	-1	S
M5005_Spy_0211	rpmH	217191	217325	44	-1	S	-1	S	-1	S
M5005_Spy_0212	-	217599	218303	234	0	NE	0	NE	0	NE
M5005_Spy_0213	-	218352	219671	439	0	NE	0	NE	0	NE
M5005_Spy_0214	-	219774	220661	295	0	NE	0	NE	0	NE
M5005_Spy_0215	-	220674	221504	276	0	NE	0	NE	0	NE
M5005_Spy_0216	-	221661	222323	220	0	NE	0	NE	0	NE
M5005_Spy_0217	nanH	222335	223249	304	0	NE	0	NE	0	NE
M5005_Spy_0218	-	223271	224209	312	0	NE	0	NE	0	NE
M5005_Spy_0219c	-	224320	225150	276	0	NE	0	NE	0	NE
M5005_Spy_0220	tatD	225402	226226	274	0	NE	0	NE	0	NE
M5005_Spy_0221	-	226198	226788	196	0	NE	0	NE	0	NE
M5005_Spy_0222	ksgA	226902	227774	290	0.0025	NE	0	NE	0	NE
M5005_Spy_0223	engC	228198	229070	290	0.639	U	0.99975	E	1	E
M5005_Spy_0224	rpe	229080	229742	220	0.00025	NE	0	NE	1	E
M5005_Spy_0225	-	229735	230367	210	0	NE	0	NE	0.001	NE
M5005_Spy_0226	-	230369	231640	423	0	NE	0	NE	0	NE
M5005_Spy_0227	cbf	231630	232568	312	0	NE	0	NE	0	NE
M5005_Spy_0228	purR	232835	233674	279	0	NE	0	NE	0	NE
M5005_Spy_0229	prgA	233665	236286	873	0.00025	NE	0	NE	0	NE
M5005_Spy_0230	rpsL	236494	236907	137	0.99225	U	0.99975	E	0.992	E
M5005_Spy_0231	rpsG	236928	237398	156	0.964	U	1	E	0.998	E
M5005_Spy_0232	fus	237765	239843	692	1	E	1	E	1	E
M5005_Spy_0233	plr	240191	241201	336	1	E	1	E	1	E
M5005_Spy_0234c	-	241427	241543	38	-1	S	-1	S	-1	S
M5005_Spy_0235c	-	241685	242425	246	1	E	1	E	1	E
M5005_Spy_0236c	-	242418	243986	522	0.67175	U	1	E	1	E
M5005_Spy_0237	-	244184	246082	632	0	NE	0	NE	0	NE
M5005_Spy_0238	uppP/bacA	246148	246987	279	0	NE	0	NE	0	NE
M5005_Spy_0239	mecA	247133	247894	253	0	NE	0	NE	0	NE
M5005_Spy_0240	rgpG	248045	249070	341	1	E	1	E	1	E
M5005_Spy_0241	rgpG	249040	249165	41	-1	S	-1	S	-1	S
M5005_Spy_0242	-	249192	249962	256	0	NE	0	NE	0.998	E
M5005_Spy_0243	-	250057	251319	420	0	NE	0	NE	1	E
M5005_Spy_0244	nifS3	251350	252576	408	0	NE	0.00025	NE	1	E
M5005_Spy_0245	nifU	252563	253042	159	0.00025	NE	0	NE	1	E
M5005_Spy_0246	-	253035	254453	472	0.0005	NE	0	NE	1	E
M5005_Spy_0247c	pbp7	254605	255786	393	0	NE	0	NE	0	NE
M5005_Spy_0248c	dacA2	255954	257186	410	0	NE	0	NE	0	NE
M5005_Spy_0249	oppA	257517	259487	656	0	NE	0	NE	0	NE
M5005_Spy_0250	oppB	259540	261054	504	0	NE	0	NE	0	NE

M5005_Spy_0251	oppC	261054	261980	308	0	NE	0	NE	0	NE
M5005_Spy_0252	oppD	261989	263059	356	0	NE	0	NE	0	NE
M5005_Spy_0253	oppF	263052	263975	307	0	NE	0	NE	0	NE
M5005_Spy_0254c	-	264216	264362	48	-1	S	-1	S	-1	S
M5005_Spy_0255c	-	264737	264871	44	-1	S	-1	S	-1	S
M5005_Spy_0256	comX1.1	270293	270844	183	0	NE	0	NE	0	NE
M5005_Spy_0257	-	271455	272159	234	0	NE	0	NE	0	NE
M5005_Spy_0258	-	272062	272577	171	0	NE	0	NE	0	NE
M5005_Spy_0259	-	272561	272665	34	-1	S	-1	S	-1	S
M5005_Spy_0260	-	272683	273267	194	0.99975	E	0.99975	E	1	E
M5005_Spy_0261	-	273267	274385	372	0.86825	U	0.91825	U	1	E
M5005_Spy_0262	-	274410	274718	102	0	NE	0	NE	0	NE
M5005_Spy_0263	nadD	274787	275419	210	0.4555	U	0.99875	E	1	E
M5005_Spy_0264	-	275416	276009	197	0	NE	0	NE	0.001	NE
M5005_Spy_0265	-	276009	276386	125	0.00025	NE	0	NE	0	NE
M5005_Spy_0266	-	276428	277177	249	0	NE	0	NE	0	NE
M5005_Spy_0267	-	277431	278537	368	0	NE	0	NE	0	NE
M5005_Spy_0268	-	278874	279029	51	-1	S	-1	S	-1	S
M5005_Spy_0269	-	279019	279735	238	0	NE	0	NE	0	NE
M5005_Spy_0270	atmA	279938	280780	280	0.00025	NE	0	NE	0	NE
M5005_Spy_0271	atmB	281109	281954	281	0	NE	0	NE	0	NE
M5005_Spy_0272	atmD	282204	283268	354	0	NE	0	NE	0	NE
M5005_Spy_0273	atmE	283269	283961	230	0	NE	0	NE	0	NE
M5005_Spy_0274c	braB	284015	285385	456	0	NE	0	NE	0	NE
M5005_Spy_0275	-	285619	286833	404	0.00025	NE	0	NE	0.001	NE
M5005_Spy_0276c	-	286888	287562	224	0	NE	0	NE	0.997	E
M5005_Spy_0277c	-	287572	288963	463	0	NE	0	NE	0.001	NE
M5005_Spy_0278c	gidB	289033	289746	237	0.00025	NE	0	NE	0	NE
M5005_Spy_0279	lemA	289896	290453	185	0	NE	0	NE	0	NE
M5005_Spy_0280	htpX	290500	291396	298	0	NE	0	NE	0	NE
M5005_Spy_0281	-	291630	292163	177	0	NE	0	NE	0	NE
M5005_Spy_0282	covR	292430	293116	228	0	NE	0	NE	0	NE
M5005_Spy_0283	covS	293259	294623	454	0	NE	0	NE	0	NE
M5005_Spy_0284	nrdR	294838	295332	164	0	NE	0	NE	0.001	NE
M5005_Spy_0285	dnaB	295316	296491	391	0.00075	NE	1	E	1	E
M5005_Spy_0286	dnaI	296492	297394	300	1	E	1	E	1	E
M5005_Spy_0287	engA	297457	298767	436	1	E	1	E	1	E
M5005_Spy_0288	snf	298974	302072	1032	0.00025	NE	0	NE	0.002	NE
M5005_Spy_0289	-	302315	302917	200	0	NE	0	NE	0	NE
M5005_Spy_0290	murC	302957	304285	442	0.0165	NE	1	E	1	E
M5005_Spy_0291	-	304331	304813	160	0	NE	0	NE	0	NE
M5005_Spy_0292	-	304931	306499	522	0.0005	NE	0	NE	1	E
M5005_Spy_0293	greA	306524	307054	176	0	NE	0	NE	0	NE
M5005_Spy_0294c	-	307269	307412	47	-1	S	-1	S	-1	S
M5005_Spy_0295c	oxaA	307677	308600	307	0.262	U	1	E	1	E
M5005_Spy_0296c	-	308682	308960	92	0	NE	0	NE	0	NE
M5005_Spy_0297c	-	309115	309630	171	0	NE	0	NE	0	NE
M5005_Spy_0298c	-	309533	310237	234	0	NE	0	NE	0	NE
M5005_Spy_0299	-	310535	311272	245	0	NE	0	NE	0	NE
M5005_Spy_0300	-	311311	311811	166	0	NE	0	NE	0	NE
M5005_Spy_0301	-	311826	312515	229	0	NE	0	NE	0	NE
M5005_Spy_0302	-	312693	312935	80	0.001	NE	0.97375	U	0.999	E
M5005_Spy_0303	glr	313113	313907	264	0.62175	U	1	E	1	E
M5005_Spy_0304	-	313904	314890	328	0	NE	0	NE	0	NE
M5005_Spy_0305	-	314869	315390	173	0.00025	NE	0	NE	0	NE
M5005_Spy_0306	-	315387	315848	153	0	NE	0	NE	0	NE
M5005_Spy_0307	xerD	315845	316591	248	0.00025	NE	0	NE	0	NE
M5005_Spy_0308	scpA	316591	317292	233	0	NE	0	NE	0	NE
M5005_Spy_0309	scpB	317289	317840	183	0.00025	NE	0	NE	0.001	NE
M5005_Spy_0310	rluB	317939	318685	248	0.00025	NE	0	NE	0	NE
M5005_Spy_0311	-	318682	318945	87	0	NE	0	NE	0	NE
M5005_Spy_0312	-	319123	319671	182	0.00025	NE	0	NE	0	NE
M5005_Spy_0313	-	319982	320545	187	0.00025	NE	0.00025	NE	1	E

M5005_Spy_0314	-	320547	321200	217	0	NE	0	NE	0	NE
M5005_Spy_0315	-	321493	322413	306	0	NE	0	NE	0	NE
M5005_Spy_0316	-	322452	323006	184	0	NE	0	NE	0	NE
M5005_Spy_0317	hlyX	323139	324473	444	0	NE	0	NE	0	NE
M5005_Spy_0318	pflC	324479	325342	287	0	NE	0	NE	0	NE
M5005_Spy_0319	ppaC	325473	326408	311	0.99825	E	0.0255	NE	0.986	U
M5005_Spy_0320	-	326484	327137	217	0	NE	0	NE	0	NE
M5005_Spy_0321c	fhuG	327182	328108	308	0	NE	0	NE	0	NE
M5005_Spy_0322c	fhuB	328180	329232	350	0	NE	0	NE	0	NE
M5005_Spy_0323c	fhuD	329222	330154	310	0	NE	0	NE	0	NE
M5005_Spy_0324c	fhuA	330180	330962	260	0	NE	0	NE	0	NE
M5005_Spy_0325c	murE	331208	332653	481	1	E	1	E	1	E
M5005_Spy_0326	-	332741	334375	544	0.98475	U	1	E	1	E
M5005_Spy_0327	upp	334543	335172	209	0	NE	0	NE	0.001	NE
M5005_Spy_0328	clpP	335396	335986	196	0.00025	NE	0.00175	NE	1	E
M5005_Spy_0329	-	336478	336753	91	0	NE	0	NE	0	NE
M5005_Spy_0330	tmk	337002	337637	211	0.998	E	1	E	1	E
M5005_Spy_0331	dnaX	337655	338530	291	0.99725	E	1	E	1	E
M5005_Spy_0332	-	338549	338788	79	0.00025	NE	0	NE	0	NE
M5005_Spy_0333	-	338877	339038	53	-1	S	-1	S	-1	S
M5005_Spy_0334	-	339193	339516	107	0.00625	NE	0	NE	0.05	U
M5005_Spy_0335	-	339521	340384	287	0	NE	0	NE	0.001	NE
M5005_Spy_0336	-	340411	340803	130	0.00025	NE	0	NE	0.001	NE
M5005_Spy_0337c	cutC	340850	341479	209	0	NE	0	NE	0	NE
M5005_Spy_0338	-	341778	342134	118	0.00025	NE	0	NE	0	NE
M5005_Spy_0339c	exoA	342208	343119	303	0.05625	U	0	NE	0	NE
M5005_Spy_0340	lctO	343269	344450	393	0	NE	0	NE	0	NE
M5005_Spy_0341	prtS	344713	349656	1647	0	NE	0	NE	0	NE
M5005_Spy_0343	-	350145	350324	59	0.01475	NE	0.00025	NE	0.017	NE
M5005_Spy_0344	-	350427	351134	235	0	NE	0	NE	0	NE
M5005_Spy_0345	metG	351377	353374	665	1	E	1	E	1	E
M5005_Spy_0346	-	353705	353872	55	-1	S	-1	S	-1	S
M5005_Spy_0347	nrdF	353869	354882	337	0	NE	0	NE	0	NE
M5005_Spy_0348	nrdI	354886	355374	162	0	NE	0	NE	0	NE
M5005_Spy_0349	nrdE.1	355341	357521	726	0	NE	0	NE	0	NE
M5005_Spy_0350	-	357500	357718	72	-1	S	-1	S	-1	S
M5005_Spy_0351c	spyA	357724	358476	250	0	NE	0	NE	0	NE
M5005_Spy_0352	-	358935	359471	178	0	NE	0	NE	0	NE
M5005_Spy_0353	-	359613	359996	127	0.00025	NE	0	NE	0	NE
M5005_Spy_0354c	-	360090	360722	210	0	NE	0	NE	0	NE
M5005_Spy_0355	-	361401	361721	106	0	NE	0	NE	0	NE
M5005_Spy_0356c	speJ	362034	362732	232	0	NE	0	NE	0	NE
M5005_Spy_0357c	-	362980	363594	204	0.00025	NE	0	NE	0	NE
M5005_Spy_0358	-	363955	364191	78	0	NE	0	NE	0	NE
M5005_Spy_0359	fabG	364175	364882	235	0	NE	0	NE	0	NE
M5005_Spy_0360	-	364928	365917	329	0	NE	0	NE	0	NE
M5005_Spy_0361	glpT	366250	367587	445	0	NE	0	NE	0	NE
M5005_Spy_0362	glmU	367760	369142	460	1	E	1	E	1	E
M5005_Spy_0363	-	369173	369727	184	0.00025	NE	0	NE	0	NE
M5005_Spy_0364	-	369727	369978	83	0.07725	U	0	NE	0	NE
M5005_Spy_0365	pfs	369998	370693	231	0.0005	NE	1	E	1	E
M5005_Spy_0366	-	370844	371185	113	0	NE	0	NE	0	NE
M5005_Spy_0367c	mtsR	371286	371933	215	0	NE	0	NE	0.235	U
M5005_Spy_0368	mtsA	372091	373011	306	0	NE	0	NE	0	NE
M5005_Spy_0369	mtsB	373075	373800	241	0	NE	0	NE	0	NE
M5005_Spy_0370	mtsC	373801	374655	284	0	NE	0	NE	0	NE
M5005_Spy_0371c	cypB	374803	375609	268	0	NE	0	NE	0	NE
M5005_Spy_0372	ftsK	375826	378231	801	1	E	1	E	1	E
M5005_Spy_0373c	-	378301	378654	117	0	NE	0	NE	0	NE
M5005_Spy_0374	rplK	378898	379323	141	0.9995	E	1	E	0.926	U
M5005_Spy_0375	rplA	379429	380118	229	0.9995	E	1	E	1	E
M5005_Spy_0376	-	380469	381602	377	0	NE	0	NE	0	NE
M5005_Spy_0377	pyrH	381767	381937	56	1	E	1	E	1	E

M5005_Spy_0380	frr	382524	383081	185	0.9895	U	1	E	0.997	E
M5005_Spy_0381	-	383190	384047	285	0.00025	NE	0	NE	0	NE
M5005_Spy_0382	msrA.2	384120	384629	169	0	NE	0	NE	0	NE
M5005_Spy_0383	-	384626	384841	71	0	NE	0	NE	0	NE
M5005_Spy_0384	-	384997	386166	389	0	NE	0	NE	0	NE
M5005_Spy_0385	-	386441	388252	603	0	NE	0	NE	0	NE
M5005_Spy_0386	phoH	388411	389463	350	0	NE	0	NE	0	NE
M5005_Spy_0387	-	389509	390084	191	0	NE	0	NE	0	NE
M5005_Spy_0388	-	390192	390740	182	0.00025	NE	0.999	E	1	E
M5005_Spy_0389	dgk	390721	391128	135	0.0265	NE	1	E	1	E
M5005_Spy_0390	era	391248	392144	298	0.6295	U	0.469	U	1	E
M5005_Spy_0391	-	392128	392640	170	0	NE	0	NE	0	NE
M5005_Spy_0392c	-	392945	393199	84	0	NE	0	NE	0	NE
M5005_Spy_0393	-	393597	393842	81	0	NE	0	NE	0	NE
M5005_Spy_0394	-	393857	394039	60	0	NE	0	NE	0	NE
M5005_Spy_0395c	-	394230	394409	59	0	NE	0	NE	0	NE
M5005_Spy_0396c	-	394348	394497	49	-1	S	-1	S	-1	S
M5005_Spy_0397c	-	394494	394916	140	0	NE	0	NE	0	NE
M5005_Spy_0398	-	395189	395398	69	0	NE	0	NE	0	NE
M5005_Spy_0399	-	395463	395663	66	0	NE	0	NE	0	NE
M5005_Spy_0400	silD	395957	396151	64	0	NE	0	NE	0	NE
M5005_Spy_0401c	-	396344	396460	38	-1	S	-1	S	-1	S
M5005_Spy_0402	-	396644	397639	331	0	NE	0	NE	0	NE
M5005_Spy_0403	-	397684	398130	148	0	NE	0	NE	0	NE
M5005_Spy_0404c	-	398478	398747	89	0	NE	0	NE	0	NE
M5005_Spy_0405	-	398948	399055	35	-1	S	-1	S	-1	S
M5005_Spy_0406c	-	399269	399511	80	0	NE	0	NE	0	NE
M5005_Spy_0407	mutR	399818	400684	288	0	NE	0	NE	0	NE
M5005_Spy_0408	fpg	400856	401683	275	0	NE	0	NE	0	NE
M5005_Spy_0409	coaE	401593	402273	226	1	E	1	E	1	E
M5005_Spy_0410	-	402463	403965	500	0	NE	0	NE	0	NE
M5005_Spy_0411	-	404087	405280	397	0	NE	0	NE	0	NE
M5005_Spy_0412	rpmG	405277	405423	48	-1	S	-1	S	-1	S
M5005_Spy_0413	secG	405469	405705	78	0.9975	E	1	E	1	E
M5005_Spy_0414	-	405802	408132	776	0	NE	0	NE	0	NE
M5005_Spy_0415	smpB	408135	408602	155	0.00025	NE	0	NE	0.262	U
M5005_Spy_0416	-	408617	409327	236	0	NE	0	NE	0	NE
M5005_Spy_0417c	pcp	409443	410090	215	0	NE	0	NE	0	NE
M5005_Spy_0418c	-	410140	411066	308	0	NE	0	NE	0	NE
M5005_Spy_0419c	-	411066	411749	227	0.00025	NE	0	NE	0.001	NE
M5005_Spy_0420c	-	411960	412886	308	0	NE	0	NE	0	NE
M5005_Spy_0421c	gloA	413031	413408	125	0.00025	NE	0	NE	0	NE
M5005_Spy_0422c	-	413419	414084	221	0	NE	0	NE	0	NE
M5005_Spy_0423c	pepQ	414133	415218	361	0.00225	NE	0	NE	0	NE
M5005_Spy_0424	ccpA	415392	416393	333	0.99075	U	0.00025	NE	0.075	U
M5005_Spy_0425	-	416524	417522	332	0.00025	NE	0.5615	U	1	E
M5005_Spy_0426	-	417524	418858	444	0.98575	U	1	E	1	E
M5005_Spy_0427	thrS	419280	421223	647	1	E	1	E	1	E
M5005_Spy_0428	drrA/tagH	421364	422356	330	0	NE	0	NE	0	NE
M5005_Spy_0429	-	422358	423176	272	0	NE	0	NE	0	NE
M5005_Spy_0430	-	423178	423963	261	0	NE	0	NE	0	NE
M5005_Spy_0431	-	424164	424313	49	-1	S	-1	S	-1	S
M5005_Spy_0432	-	424708	425856	382	0	NE	0	NE	0	NE
M5005_Spy_0433	-	425813	427060	415	0	NE	0	NE	0	NE
M5005_Spy_0434	-	427116	428150	344	0	NE	0	NE	0	NE
M5005_Spy_0435	vicR	428312	429022	236	1	E	0.99975	E	1	E
M5005_Spy_0436	vicK	429015	430367	450	0.00025	NE	0	NE	0.001	NE
M5005_Spy_0437	vicX	430371	431180	269	0	NE	0	NE	0	NE
M5005_Spy_0438	rnc	431624	432316	230	0.00025	NE	0	NE	1	E
M5005_Spy_0439	smc	432317	435856	1179	0	NE	0	NE	0	NE
M5005_Spy_0440c	-	436109	436960	283	0	NE	0	NE	0	NE
M5005_Spy_0441	aroE	437234	438106	290	0	NE	0	NE	0	NE
M5005_Spy_0442	-	438205	438939	244	0	NE	0	NE	0.001	NE

M5005_Spy_0443	-	438941	439675	244	0	NE	0	NE	0	NE
M5005_Spy_0444	-	439668	440654	328	0	NE	0	NE	0	NE
M5005_Spy_0445	metK1	440664	441863	399	0	NE	0	NE	0	NE
M5005_Spy_0446	-	441847	442815	322	0.00025	NE	0	NE	0	NE
M5005_Spy_0447	-	442870	443859	329	0	NE	0	NE	0	NE
M5005_Spy_0448	-	444385	444528	47	-1	S	-1	S	-1	S
M5005_Spy_0449	hasB.2	444552	445709	385	0.00025	NE	0	NE	0	NE
M5005_Spy_0450	mefE	445794	447002	402	0	NE	0	NE	0	NE
M5005_Spy_0451c	-	447105	447314	69	0	NE	0	NE	0	NE
M5005_Spy_0452c	-	447304	448149	281	0	NE	0	NE	0	NE
M5005_Spy_0453c	-	448234	448962	242	0	NE	0	NE	0	NE
M5005_Spy_0454c	-	448953	449177	74	0.00025	NE	0	NE	0	NE
M5005_Spy_0455c	-	449177	450271	364	0	NE	0	NE	0	NE
M5005_Spy_0456	-	450317	450619	100	0.07525	U	0.00025	NE	0.606	U
M5005_Spy_0457	-	450619	450924	101	0	NE	0	NE	0	NE
M5005_Spy_0458	-	450943	451215	90	0	NE	0	NE	0	NE
M5005_Spy_0459	-	451320	451772	150	0.00025	NE	0	NE	0	NE
M5005_Spy_0460	-	451838	452152	104	0	NE	0	NE	0	NE
M5005_Spy_0461	-	452569	452907	112	0.00025	NE	0	NE	0	NE
M5005_Spy_0462	-	452931	453755	274	0	NE	0	NE	0	NE
M5005_Spy_0463	-	453769	455016	415	0	NE	0	NE	0	NE
M5005_Spy_0464	mccF	455244	455960	238	0	NE	0	NE	0	NE
M5005_Spy_0465	-	455973	456173	66	0.017	NE	0	NE	0	NE
M5005_Spy_0466	-	456293	457192	299	0	NE	0	NE	0	NE
M5005_Spy_0467c	-	457645	458358	237	0	NE	0	NE	0	NE
M5005_Spy_0468c	-	458406	458681	91	0.00825	NE	0	NE	0	NE
M5005_Spy_0469	-	458701	458919	72	0.00025	NE	0	NE	0	NE
M5005_Spy_0470	-	458986	459783	265	0	NE	0	NE	0	NE
M5005_Spy_0471	-	459787	460611	274	0	NE	0	NE	0	NE
M5005_Spy_0472	ftsY	460611	462161	516	0.99775	E	1	E	1	E
M5005_Spy_0473c	-	462215	463582	455	0	NE	0	NE	0	NE
M5005_Spy_0474	licT/bglG	463910	464752	280	0	NE	0	NE	0	NE
M5005_Spy_0475	-	464916	466616	566	0	NE	0	NE	0	NE
M5005_Spy_0476	bglA	466635	468059	474	0	NE	0	NE	0	NE
M5005_Spy_0477c	-	468158	468973	271	0	NE	0	NE	0	NE
M5005_Spy_0478c	-	468973	469875	300	0	NE	0	NE	0	NE
M5005_Spy_0479	-	470009	470206	65	0	NE	0	NE	0	NE
M5005_Spy_0480	-	470304	470432	42	0	NE	0	NE	0.001	NE
M5005_Spy_0481c	-	470412	472544	710	-1	S	-1	S	-1	S
M5005_Spy_0482	-	472486	472968	160	0	NE	0	NE	0	NE
M5005_Spy_0483	-	473032	473304	90	0	NE	0	NE	0	NE
M5005_Spy_0484	ptsK	473609	474601	330	0.012	NE	1	E	1	E
M5005_Spy_0485	lgt	474598	475377	259	0.00025	NE	0	NE	0	NE
M5005_Spy_0486	-	475399	475806	135	0	NE	0	NE	0	NE
M5005_Spy_0487	-	475799	476227	142	0	NE	0	NE	0	NE
M5005_Spy_0488c	-	476386	476670	94	0.00025	NE	0	NE	0.001	NE
M5005_Spy_0489	-	476869	477237	122	0	NE	0	NE	0	NE
M5005_Spy_0491	-	477887	479173	428	0	NE	0	NE	0	NE
M5005_Spy_0492c	-	479186	479314	42	-1	S	-1	S	-1	S
M5005_Spy_0493	-	479383	479595	70	0	NE	0	NE	0	NE
M5005_Spy_0494c	-	479692	479832	46	-1	S	-1	S	-1	S
M5005_Spy_0495c	lysS	479968	481461	497	1	E	1	E	1	E
M5005_Spy_0496	-	481635	482537	300	0.00025	NE	0	NE	0	NE
M5005_Spy_0497c	-	482645	483268	207	0	NE	0	NE	0	NE
M5005_Spy_0498c	-	483609	484088	159	0	NE	0	NE	0	NE
M5005_Spy_0499c	-	484179	484742	187	0	NE	0	NE	0	NE
M5005_Spy_0500c	-	485011	485859	282	0.00025	NE	0	NE	1	E
M5005_Spy_0501	-	486184	486687	167	0	NE	0	NE	0	NE
M5005_Spy_0502	-	486671	487057	128	0.00025	NE	0	NE	0	NE
M5005_Spy_0503c	-	487103	487582	159	0	NE	0	NE	0	NE
M5005_Spy_0504c	pepF	487575	489374	599	0	NE	0	NE	0	NE
M5005_Spy_0505	ppc	489518	492331	937	0	NE	0	NE	0	NE
M5005_Spy_0506	ftsW	492501	493775	424	1	E	1	E	1	E

M5005_Spy_0507c	-	493792	493932	46	-1	S	-1	S	-1	S
M5005_Spy_0508	tuf	494129	495325	398	1	E	1	E	1	E
M5005_Spy_0509	tpiA	495566	496324	252	0.97425	U	0.98925	U	1	E
M5005_Spy_0510c	murN	496423	497658	411	1	E	1	E	1	E
M5005_Spy_0511c	murM	497645	498871	408	1	E	1	E	1	E
M5005_Spy_0512c	-	498871	499680	269	0	NE	0	NE	0	NE
M5005_Spy_0513c	-	499831	500064	77	0	NE	0.84975	U	0.584	U
M5005_Spy_0514c	-	500136	501437	433	0	NE	0	NE	0	NE
M5005_Spy_0515	-	501519	501905	128	0	NE	0	NE	0	NE
M5005_Spy_0516	pacL	502136	504817	893	1	E	1	E	1	E
M5005_Spy_0517c	regR	504901	505896	331	0	NE	0	NE	0	NE
M5005_Spy_0518c	-	505960	507867	635	0	NE	0	NE	0	NE
M5005_Spy_0519c	agaD	507954	508775	273	0	NE	0	NE	0	NE
M5005_Spy_0520c	agaW	508762	509544	260	0	NE	0	NE	0	NE
M5005_Spy_0521c	agaV	509563	510051	162	0.00025	NE	0	NE	0	NE
M5005_Spy_0522c	ugl	510087	511286	399	0.00025	NE	0	NE	0.001	NE
M5005_Spy_0523	-	511353	511883	176	0	NE	0	NE	0	NE
M5005_Spy_0524	idnO	512059	512853	264	0	NE	0	NE	0	NE
M5005_Spy_0525	-	512878	513519	213	0	NE	0	NE	0	NE
M5005_Spy_0526	kdgK	513548	514549	333	0	NE	0	NE	0	NE
M5005_Spy_0527	kgdA	514554	515189	211	0	NE	0	NE	0	NE
M5005_Spy_0528	-	515485	516135	216	0	NE	0	NE	0.001	NE
M5005_Spy_0529	-	516776	517951	391	0.00025	NE	0	NE	0	NE
M5005_Spy_0530	prfB	518105	519118	337	0.02825	NE	1	E	1	E
M5005_Spy_0531	ftsE	519137	519829	230	0.00025	NE	0.974	U	0.091	U
M5005_Spy_0532	ftsX	519822	520751	309	0.0005	NE	0.985	U	1	E
M5005_Spy_0533c	-	521061	521696	211	0.00025	NE	0	NE	0	NE
M5005_Spy_0534	-	521927	522541	204	0	NE	0	NE	0	NE
M5005_Spy_0535	-	522545	522691	48	-1	S	-1	S	-1	S
M5005_Spy_0536	dinG	522841	525300	819	0	NE	0	NE	0	NE
M5005_Spy_0537	aspC	525635	526828	397	0.00025	NE	0	NE	0	NE
M5005_Spy_0538	asnC	526849	528195	448	1	E	1	E	1	E
M5005_Spy_0539	-	528609	529499	296	0.00025	NE	0	NE	0.001	NE
M5005_Spy_0540	-	529496	530473	325	0	NE	0	NE	0	NE
M5005_Spy_0541	-	530470	531381	303	0.00025	NE	0	NE	0.01	NE
M5005_Spy_0542	pepD	531514	532911	465	0	NE	0	NE	0	NE
M5005_Spy_0543	adcA	533063	534610	515	0	NE	0	NE	0	NE
M5005_Spy_0544	-	534758	535480	240	0	NE	0	NE	0	NE
M5005_Spy_0545	agaS	535499	536698	399	0	NE	0	NE	0	NE
M5005_Spy_0546c	rpmE2	536795	537055	86	0.9685	U	0	NE	0.998	E
M5005_Spy_0547c	-	537170	538111	313	0.00025	NE	0.00225	NE	0.757	U
M5005_Spy_0548	flaV	538505	538954	149	0.0005	NE	0	NE	1	E
M5005_Spy_0549	-	539130	539414	94	0	NE	0	NE	0	NE
M5005_Spy_0550	-	539407	540669	420	0	NE	0	NE	0.001	NE
M5005_Spy_0551	rplS	540784	541131	115	0.822	U	1	E	0.985	U
M5005_Spy_0552	-	542134	542703	189	0.002	NE	0	NE	0.001	NE
M5005_Spy_0553	gyrB	542704	544656	650	1	E	1	E	1	E
M5005_Spy_0554	ezrA	545024	546748	574	0.9785	U	1	E	1	E
M5005_Spy_0555c	-	546880	547338	152	0	NE	0	NE	0	NE
M5005_Spy_0556	eno	547565	548872	435	1	E	1	E	1	E
M5005_Spy_0557c	-	549398	549988	196	0	NE	0	NE	0	NE
M5005_Spy_0558c	-	550037	550288	83	0	NE	0	NE	0	NE
M5005_Spy_0559c	-	550650	551306	218	0	NE	0	NE	0	NE
M5005_Spy_0560c	-	551303	552190	295	0.00025	NE	0	NE	0	NE
M5005_Spy_0561	epf	552662	558841	2059	0	NE	0	NE	0	NE
M5005_Spy_0562	sagA	559706	559867	53	0	NE	0	NE	0	NE
M5005_Spy_0563	sagB	560089	561039	316	0	NE	0	NE	0	NE
M5005_Spy_0564	sagC	561036	562094	352	0	NE	0	NE	0	NE
M5005_Spy_0565	sagD	562114	563472	452	0	NE	0	NE	0	NE
M5005_Spy_0566	sagE	563447	564118	223	0	NE	0	NE	0	NE
M5005_Spy_0567	sagF	564115	564798	227	0	NE	0	NE	0	NE
M5005_Spy_0568	sagG	564821	565744	307	0.00025	NE	0	NE	0.003	NE
M5005_Spy_0569	sagH	565753	566880	375	0.00025	NE	0	NE	0.001	NE

M5005_Spy_0570	sagI	566877	567995	372	0	NE	0	NE	0.001	NE
M5005_Spy_0571	-	568566	571298	910	0.00025	NE	0	NE	0	NE
M5005_Spy_0572	-	571576	572076	166	0	NE	0	NE	0	NE
M5005_Spy_0573	ligA	572270	574228	652	1	E	1	E	1	E
M5005_Spy_0574	-	574242	575264	340	1	E	1	E	1	E
M5005_Spy_0575	atpE	575657	575854	65	0.88075	U	0.02675	NE	0.989	U
M5005_Spy_0576	atpB	575889	576605	238	1	E	1	E	0.007	NE
M5005_Spy_0577	atpF	576623	577117	164	0.108	U	1	E	1	E
M5005_Spy_0578	atpH	577117	577653	178	1	E	1	E	1	E
M5005_Spy_0579	atpA	577669	579177	502	1	E	1	E	1	E
M5005_Spy_0580	atpG	579193	580068	291	0.9815	U	1	E	1	E
M5005_Spy_0581	atpD	580230	581636	468	1	E	1	E	1	E
M5005_Spy_0582	atpC	581649	582065	138	0.4415	U	0.80875	U	0.991	U
M5005_Spy_0583	-	582331	582588	85	0	NE	0	NE	0	NE
M5005_Spy_0584	murA	582815	583924	369	0	NE	0	NE	0	NE
M5005_Spy_0585	epuA	583928	584116	62	0	NE	0	NE	0	NE
M5005_Spy_0586	endA	584152	584691	179	0	NE	0	NE	0	NE
M5005_Spy_0587	pheS	584974	586017	347	1	E	1	E	1	E
M5005_Spy_0588	pheT	586212	588632	806	1	E	1	E	1	E
M5005_Spy_0589	-	588742	589119	125	0	NE	0	NE	0	NE
M5005_Spy_0590	-	589112	589498	128	0	NE	0	NE	0	NE
M5005_Spy_0591	-	589571	590647	358	0	NE	0	NE	0	NE
M5005_Spy_0592	-	590657	591325	222	0	NE	0	NE	0	NE
M5005_Spy_0593c	-	591427	592344	305	0	NE	0	NE	0	NE
M5005_Spy_0594	rexB	592495	595710	1071	0.82275	U	0	NE	0.818	U
M5005_Spy_0595	rexA	595671	599339	1222	0.00025	NE	0	NE	1	E
M5005_Spy_0596	-	599479	600291	270	0	NE	0	NE	0	NE
M5005_Spy_0597	rpsU	600432	600608	58	0.99425	U	1	E	1	E
M5005_Spy_0598c	mscL	600736	601098	120	0	NE	0	NE	0	NE
M5005_Spy_0599	dnaG	601229	603043	604	1	E	1	E	1	E
M5005_Spy_0600	rpoD	603052	604161	369	1	E	1	E	1	E
M5005_Spy_0601	-	604397	604735	112	0	NE	0	NE	0	NE
M5005_Spy_0602	rmlD	604873	605727	284	1	E	1	E	0.999	E
M5005_Spy_0603	rgpAc	605846	607000	384	0.0005	NE	1	E	1	E
M5005_Spy_0604	rgpBc	606990	607922	310	0.942	U	1	E	1	E
M5005_Spy_0605	rgpCc	607925	608728	267	0.99925	E	1	E	1	E
M5005_Spy_0606	rgpDc	608728	609933	401	0.9975	E	0.999	E	1	E
M5005_Spy_0607	rgpEc	609958	610965	335	1	E	1	E	1	E
M5005_Spy_0608	rgpFc	610962	612707	581	1	E	1	E	1	E
M5005_Spy_0609	-	612704	615178	824	0.00025	NE	0	NE	0	NE
M5005_Spy_0610	-	615357	616052	231	0	NE	0	NE	0	NE
M5005_Spy_0611	-	616054	616395	113	0	NE	0	NE	0	NE
M5005_Spy_0612	amrA	616388	617674	428	0.00025	NE	0.00025	NE	1	E
M5005_Spy_0613	-	617655	619151	498	0.00025	NE	1	E	1	E
M5005_Spy_0614	pepT	619245	620468	407	0	NE	0	NE	0	NE
M5005_Spy_0615	ebsA	620509	620997	162	0	NE	0	NE	0	NE
M5005_Spy_0616c	-	620984	621181	65	0	NE	0	NE	0	NE
M5005_Spy_0617	-	621230	621706	158	0.00025	NE	0	NE	0.302	U
M5005_Spy_0618	cmk	621721	622401	226	0.0005	NE	0.98775	U	0.997	E
M5005_Spy_0619	infC	622563	623093	176	0.995	U	0.99175	U	1	E
M5005_Spy_0620	rpmI	623135	623332	65	0.0065	NE	0.00925	NE	0.995	E
M5005_Spy_0621	rplT	623391	623750	119	0.00025	NE	0.99975	E	0.008	NE
M5005_Spy_0622c	-	624041	626212	723	1	E	1	E	1	E
M5005_Spy_0623	-	626359	627522	387	0	NE	0	NE	0	NE
M5005_Spy_0624	aroD	627519	628205	228	0	NE	0	NE	0	NE
M5005_Spy_0625	aroF	628299	629465	388	0	NE	0	NE	0	NE
M5005_Spy_0626	-	629526	629867	113	0.00025	NE	0	NE	0	NE
M5005_Spy_0627	gor	630088	631440	450	0	NE	0	NE	0	NE
M5005_Spy_0628c	folC.2	631528	632796	422	0	NE	0	NE	0	NE
M5005_Spy_0629c	-	632826	633266	146	0	NE	0	NE	0	NE
M5005_Spy_0630	nifS1	633501	634643	380	0	NE	0	NE	0	NE
M5005_Spy_0631	thiI	634655	635869	404	0	NE	0	NE	0	NE
M5005_Spy_0632	capA	635907	637199	430	0	NE	0	NE	0	NE

M5005_Spy_0633	rplU	637413	637727	104	0.00025	NE	0.62425	U	0.081	U
M5005_Spy_0634	-	637739	638065	108	0.58675	U	0.0325	NE	0.002	NE
M5005_Spy_0635	rpmA	638093	638386	97	0.99825	E	0.029	NE	0.003	NE
M5005_Spy_0636	-	638734	639648	304	0.00025	NE	0	NE	0	NE
M5005_Spy_0637	lsp	639645	640103	152	0	NE	0	NE	0	NE
M5005_Spy_0638	-	640093	640983	296	0	NE	0	NE	0	NE
M5005_Spy_0639	pyrR	641379	641900	173	0.00025	NE	0	NE	0	NE
M5005_Spy_0640	pyrP	641916	643175	419	0.00025	NE	0	NE	0	NE
M5005_Spy_0641	pyrB	643236	644171	311	0.00275	NE	0	NE	0	NE
M5005_Spy_0642	carA	644215	645297	360	0	NE	0	NE	0	NE
M5005_Spy_0643	carB	645523	648699	1058	0.00025	NE	0	NE	0	NE
M5005_Spy_0644	-	648999	650177	392	0	NE	0	NE	0	NE
M5005_Spy_0645	-	650177	650887	236	0	NE	0	NE	0	NE
M5005_Spy_0646	-	650899	652119	406	0	NE	0	NE	0	NE
M5005_Spy_0647	-	652373	654106	577	0	NE	0	NE	0	NE
M5005_Spy_0648	rpsP	654233	654505	90	0.00025	NE	0.00025	NE	0.001	NE
M5005_Spy_0649	-	654515	654754	79	0	NE	0	NE	0	NE
M5005_Spy_0650	-	654763	654870	35	-1	S	-1	S	-1	S
M5005_Spy_0651	-	655665	658691	1008	0	NE	0	NE	0	NE
M5005_Spy_0652	-	658711	659112	133	0	NE	0	NE	0	NE
M5005_Spy_0653c	czcD	659292	660167	291	0.00025	NE	0	NE	0	NE
M5005_Spy_0654	-	660303	660824	173	0	NE	0	NE	0	NE
M5005_Spy_0655	rimM	661039	661557	172	0	NE	0.00225	NE	0.027	NE
M5005_Spy_0656	trmD	661547	662278	243	0.9995	E	1	E	1	E
M5005_Spy_0657	trxB	662278	663270	330	0.00025	NE	0	NE	0	NE
M5005_Spy_0658	-	663448	664506	352	0	NE	0	NE	0	NE
M5005_Spy_0659	apbA	664519	665442	307	0	NE	0	NE	0	NE
M5005_Spy_0660	fruR	665698	666411	237	0	NE	0	NE	0	NE
M5005_Spy_0661	fruB	666408	667319	303	0	NE	0	NE	0.001	NE
M5005_Spy_0662	fruA	667316	669262	648	0.00025	NE	0	NE	0	NE
M5005_Spy_0663	mur1.1	669361	669954	197	0	NE	0	NE	0.001	NE
M5005_Spy_0664	mur1.2	670106	670813	235	0	NE	0	NE	0	NE
M5005_Spy_0665c	-	670870	671088	72	0	NE	0	NE	0	NE
M5005_Spy_0666c	-	671291	671410	39	-1	S	-1	S	-1	S
M5005_Spy_0667c	-	671385	671594	69	-1	S	-1	S	-1	S
M5005_Spy_0668c	mac	671769	672788	339	0	NE	0	NE	0	NE
M5005_Spy_0669	-	672944	673063	39	-1	S	-1	S	-1	S
M5005_Spy_0670	-	673287	673463	58	0	NE	0	NE	0	NE
M5005_Spy_0671	-	673691	674071	126	0	NE	0	NE	0.001	NE
M5005_Spy_0672	-	674071	674919	282	0	NE	0	NE	0	NE
M5005_Spy_0673	papS	675044	676252	402	1	E	1	E	1	E
M5005_Spy_0674	-	676249	678126	625	0.00975	NE	0	NE	0	NE
M5005_Spy_0675	-	678383	678535	50	-1	S	-1	S	-1	S
M5005_Spy_0676	-	678544	678762	72	0	NE	0	NE	0	NE
M5005_Spy_0677	fms	679018	679428	136	0	NE	0	NE	0	NE
M5005_Spy_0678c	-	679510	681522	670	0	NE	0	NE	0	NE
M5005_Spy_0679	-	681742	682392	216	0.00025	NE	0	NE	0.001	NE
M5005_Spy_0680	-	682395	683063	222	0	NE	0	NE	0	NE
M5005_Spy_0681	-	683072	684304	410	0	NE	0	NE	0	NE
M5005_Spy_0682	mvaK1	684583	685476	297	0.9995	E	1	E	0.999	E
M5005_Spy_0683	mvaD	685458	686402	314	0.77975	U	1	E	1	E
M5005_Spy_0684	mvaK2	686395	687402	335	0.99975	E	0.97825	U	1	E
M5005_Spy_0685	-	687395	688384	329	0.99975	E	1	E	1	E
M5005_Spy_0686c	-	688614	689891	425	0.07925	U	0.001	NE	1	E
M5005_Spy_0687c	mvaS.1	689878	691053	391	0.998	E	1	E	1	E
M5005_Spy_0688	thyA	691262	692101	279	0	NE	0.99925	E	0.8	U
M5005_Spy_0689	dyr	692181	692678	165	0.99975	E	1	E	1	E
M5005_Spy_0690	-	692698	692868	56	0	NE	0	NE	0	NE
M5005_Spy_0691	clpX	692998	694227	409	0.00025	NE	0	NE	0.001	NE
M5005_Spy_0692	engB	694237	694836	199	0.97325	U	1	E	1	E
M5005_Spy_0693	-	694984	695727	247	0	NE	0	NE	0	NE
M5005_Spy_0694c	clpL	695785	697884	699	0	NE	0	NE	0.001	NE
M5005_Spy_0695	rpiA	698263	698946	227	0.00025	NE	0.00025	NE	1	E

M5005_Spy_0696	deoB	699023	700234	403	0	NE	0	NE	0	NE
M5005_Spy_0697	arsC	700253	700693	146	0	NE	0	NE	0	NE
M5005_Spy_0698	punA	700677	701486	269	0	NE	0	NE	0.069	U
M5005_Spy_0699	deoD	702149	702862	237	0	NE	0.004	NE	0.012	NE
M5005_Spy_0700	cpsX	702855	703643	262	0	NE	0	NE	0	NE
M5005_Spy_0701c	cpsY	703722	704576	284	0	NE	0	NE	0	NE
M5005_Spy_0702	-	704849	705385	178	0	NE	0	NE	0.001	NE
M5005_Spy_0703	pyrF	705662	706354	230	0	NE	0	NE	0	NE
M5005_Spy_0704	pyrE	706412	707041	209	0	NE	0	NE	0	NE
M5005_Spy_0705	amiC	707238	708692	484	0	NE	0	NE	0	NE
M5005_Spy_0706	-	708810	709673	287	0	NE	0	NE	0	NE
M5005_Spy_0707	-	709702	710352	216	0	NE	0	NE	0	NE
M5005_Spy_0708	ung	710485	711138	217	0	NE	0	NE	0	NE
M5005_Spy_0709	pyrC	711270	712538	422	0.0005	NE	0	NE	0	NE
M5005_Spy_0710c	-	712596	713237	213	0.0725	U	1	E	1	E
M5005_Spy_0711	parE	713372	715321	649	0.981	U	1	E	1	E
M5005_Spy_0712	parC	715412	717871	819	1	E	1	E	1	E
M5005_Spy_0713	bcaT	717994	719016	340	0	NE	0	NE	0	NE
M5005_Spy_0714	-	719080	719310	76	0	NE	0	NE	0.077	U
M5005_Spy_0715	rpsA	719701	720906	401	0.838	U	1	E	1	E
M5005_Spy_0716c	-	721453	721755	100	0	NE	0	NE	0	NE
M5005_Spy_0717	-	721954	722940	328	0	NE	0	NE	0	NE
M5005_Spy_0718	-	723011	723202	63	0.0015	NE	0	NE	0	NE
M5005_Spy_0719	-	723251	723670	139	0	NE	0	NE	0	NE
M5005_Spy_0720	-	723907	724815	302	0	NE	0	NE	0	NE
M5005_Spy_0721c	-	724933	725109	58	0.91575	U	0.97825	U	0.971	U
M5005_Spy_0722	miaA	725243	726142	299	0	NE	0	NE	0	NE
M5005_Spy_0723	hflX	726215	727453	412	0.00025	NE	0	NE	0	NE
M5005_Spy_0724	-	727446	728081	211	0	NE	0	NE	0	NE
M5005_Spy_0725	elaC	728096	729025	309	0.98775	U	1	E	0.999	E
M5005_Spy_0726	-	729025	729789	254	0	NE	0	NE	0	NE
M5005_Spy_0727	recJ	729786	731996	736	0	NE	0	NE	0	NE
M5005_Spy_0728	apt	732147	732665	172	0	NE	0	NE	0	NE
M5005_Spy_0729	dnaD	732746	733429	227	0.98725	U	0.41925	U	0.001	NE
M5005_Spy_0730	nth	733426	734082	218	0	NE	0	NE	0	NE
M5005_Spy_0731	-	734154	734738	194	0.9245	U	1	E	1	E
M5005_Spy_0732	-	734829	735617	262	0	NE	0	NE	0	NE
M5005_Spy_0733	-	735657	736763	368	0	NE	0	NE	0	NE
M5005_Spy_0734	cpsFO/rmlA	736821	737690	289	0.22075	U	1	E	1	E
M5005_Spy_0735	cpsFP/rmlC	737690	738283	197	0.95275	U	1	E	0.001	NE
M5005_Spy_0736	cpsFQ/rmlB	738527	739567	346	0.9995	E	1	E	1	E
M5005_Spy_0737	mutX	739788	740264	158	0	NE	0	NE	0	NE
M5005_Spy_0738	-	740322	741503	393	0	NE	0	NE	0	NE
M5005_Spy_0739	-	741493	742740	415	0	NE	0	NE	0	NE
M5005_Spy_0740c	fbp	742799	744085	428	0	NE	0	NE	0.001	NE
M5005_Spy_0742	-	744497	744622	41	-1	S	-1	S	-1	S
M5005_Spy_0743	-	744804	745802	332	0.00025	NE	0	NE	0	NE
M5005_Spy_0744	-	745792	745971	59	-1	S	-1	S	-1	S
M5005_Spy_0745	-	746148	747017	289	0	NE	0	NE	0	NE
M5005_Spy_0746	-	747014	747772	252	0	NE	0	NE	0	NE
M5005_Spy_0747	-	747976	749637	553	0.011	NE	1	E	1	E
M5005_Spy_0748	estA	749770	750555	261	0	NE	0	NE	0	NE
M5005_Spy_0749	-	750585	750911	108	0	NE	0	NE	0	NE
M5005_Spy_0750	-	750957	752864	635	0	NE	0	NE	0	NE
M5005_Spy_0751	acoA	753149	754117	322	0	NE	0	NE	0	NE
M5005_Spy_0752	acoB	754173	755174	333	0	NE	0	NE	0	NE
M5005_Spy_0753	acoC	755359	756768	469	0	NE	0	NE	0	NE
M5005_Spy_0754c	-	756823	757062	79	0.0005	NE	0	NE	0	NE
M5005_Spy_0755	acoL	757095	758858	587	0.0005	NE	0	NE	0	NE
M5005_Spy_0756c	-	758874	759086	70	0	NE	0	NE	0	NE
M5005_Spy_0757c	hylA	759532	761949	805	0.00025	NE	0	NE	0	NE
M5005_Spy_0758	lplB	762182	763171	329	0	NE	0	NE	0	NE
M5005_Spy_0759c	cobQ	763280	764071	263	0.95425	U	1	E	1	E

M5005_Spy_0760c	murC2	764071	765414	447	0.999	E	1	E	1	E
M5005_Spy_0761	-	765521	766372	283	1	E	1	E	1	E
M5005_Spy_0762	-	766369	767325	318	0	NE	0	NE	0	NE
M5005_Spy_0763	glmM	767379	768734	451	0.99775	E	1	E	1	E
M5005_Spy_0764	-	768868	769509	213	0	NE	0	NE	0	NE
M5005_Spy_0765	hemN	769506	770702	398	0	NE	0	NE	0	NE
M5005_Spy_0766	-	770712	771464	250	0	NE	0	NE	1	E
M5005_Spy_0767	-	771464	772228	254	0	NE	0	NE	0	NE
M5005_Spy_0768	-	772228	772860	210	0	NE	0	NE	0	NE
M5005_Spy_0769	cas9	773340	777446	1368	0.00025	NE	0	NE	0	NE
M5005_Spy_0770	cas1	777446	778315	289	0	NE	0	NE	0	NE
M5005_Spy_0771	cas2	778312	778653	113	0	NE	0	NE	0	NE
M5005_Spy_0772	csn2	778643	779305	220	0	NE	0	NE	0	NE
M5005_Spy_0773c	-	779352	779639	95	0	NE	0	NE	0	NE
M5005_Spy_0774	-	779772	779951	59	0	NE	0	NE	0	NE
M5005_Spy_0775	-	780015	780161	48	-1	S	-1	S	-1	S
M5005_Spy_0776	lepA	780285	782117	610	0	NE	0	NE	0	NE
M5005_Spy_0777	sclB	782375	783256	293	0	NE	0	NE	0	NE
M5005_Spy_0778	msrB/crsA	783442	783879	145	0	NE	0	NE	0.001	NE
M5005_Spy_0779	-	783994	785013	339	0	NE	0	NE	0	NE
M5005_Spy_0780	-	785220	785645	141	0.00025	NE	0	NE	0	NE
M5005_Spy_0781	ptsB	785664	786155	163	0	NE	0	NE	0	NE
M5005_Spy_0782	ptsC	786172	786981	269	0	NE	0	NE	0	NE
M5005_Spy_0783	ptsD	786978	787805	275	0	NE	0	NE	0	NE
M5005_Spy_0784	-	787941	789590	549	0	NE	0	NE	0	NE
M5005_Spy_0785	-	789594	790382	262	0	NE	0	NE	0	NE
M5005_Spy_0786	-	790376	791422	348	0	NE	0	NE	0	NE
M5005_Spy_0787	-	791519	791866	115	0	NE	0	NE	0	NE
M5005_Spy_0788	-	792201	792767	188	0	NE	0	NE	0	NE
M5005_Spy_0789	-	792783	793472	229	0	NE	0	NE	0	NE
M5005_Spy_0790	gabD	793567	794964	465	0	NE	0	NE	0	NE
M5005_Spy_0791	uvrC	795066	796862	598	0.00025	NE	0	NE	0	NE
M5005_Spy_0792	-	797047	797649	200	0	NE	0	NE	0	NE
M5005_Spy_0793	-	797774	799183	469	0	NE	0	NE	0	NE
M5005_Spy_0794c	trmE	799251	800627	458	0.3345	U	0	NE	1	E
M5005_Spy_0795	rplJ	800960	801460	166	0.0025	NE	0.00025	NE	0.001	NE
M5005_Spy_0796	rplL	801525	801890	121	0.00175	NE	0.00025	NE	0.001	NE
M5005_Spy_0797	-	802286	802426	46	-1	S	-1	S	-1	S
M5005_Spy_0798	-	802420	802788	122	0	NE	0	NE	0	NE
M5005_Spy_0799	-	802767	802904	45	-1	S	-1	S	-1	S
M5005_Spy_0800	-	802963	803286	107	0	NE	0	NE	0	NE
M5005_Spy_0801	-	803344	803466	40	-1	S	-1	S	-1	S
M5005_Spy_0802	-	803599	803811	70	0	NE	0	NE	0	NE
M5005_Spy_0803	srtI	804076	804771	231	0	NE	0	NE	0	NE
M5005_Spy_0804	srtR	804922	805608	228	0	NE	0	NE	0	NE
M5005_Spy_0805	srtK	805601	806947	448	0.00025	NE	0	NE	0	NE
M5005_Spy_0806	srtA	807095	807235	46	-1	S	-1	S	-1	S
M5005_Spy_0807	srtT	807350	807967	205	0	NE	0	NE	0	NE
M5005_Spy_0808	srtF	808049	808738	229	0	NE	0	NE	0	NE
M5005_Spy_0809	srtE	808744	809493	249	0.00025	NE	0	NE	0	NE
M5005_Spy_0810	srtG	809496	810218	240	0	NE	0	NE	0	NE
M5005_Spy_0811	-	810410	810628	72	0	NE	0	NE	0	NE
M5005_Spy_0812c	-	810737	810952	71	0	NE	0	NE	0	NE
M5005_Spy_0817	dacA1	812371	813699	442	0.00025	NE	0	NE	0.001	NE
M5005_Spy_0818c	-	813816	814778	320	0	NE	0	NE	0	NE
M5005_Spy_0819	-	814866	815021	51	-1	S	-1	S	-1	S
M5005_Spy_0820	folC.1	815110	816387	425	0.99325	U	1	E	1	E
M5005_Spy_0821	folE	816434	817000	188	0	NE	0	NE	0	NE
M5005_Spy_0822	folP	817009	817809	266	0.00025	NE	0.0005	NE	1	E
M5005_Spy_0823	folQ	817816	818175	119	0.00025	NE	0	NE	0.001	NE
M5005_Spy_0824	folK	818172	818672	166	0	NE	0	NE	0.696	U
M5005_Spy_0825	murB	818822	819709	295	1	E	1	E	1	E
M5005_Spy_0826	potA	819755	820909	384	0	NE	0	NE	0.001	NE

M5005_Spy_0827	potB	820893	821687	264	0	NE	0	NE	0	NE
M5005_Spy_0828	potC	821684	822460	258	0	NE	0	NE	0	NE
M5005_Spy_0829	potD	822453	823526	357	0	NE	0	NE	0	NE
M5005_Spy_0830c	dpiA	823581	824246	221	0	NE	0	NE	0	NE
M5005_Spy_0831c	dpiB	824227	825768	513	0	NE	0	NE	0	NE
M5005_Spy_0832	malP	825929	827260	443	0.00025	NE	0	NE	0	NE
M5005_Spy_0833	-	827291	828457	388	0.00025	NE	0	NE	0	NE
M5005_Spy_0834c	-	828540	829631	363	0	NE	0	NE	0	NE
M5005_Spy_0835	aphA	829825	830187	120	0	NE	0	NE	0	NE
M5005_Spy_0837	-	830733	832265	510	0	NE	0	NE	0	NE
M5005_Spy_0838	-	832428	833042	204	0	NE	0	NE	0	NE
M5005_Spy_0839	-	833222	834349	375	0	NE	0	NE	0	NE
M5005_Spy_0840	radC	834398	835078	226	0.00025	NE	0	NE	0	NE
M5005_Spy_0841c	-	835080	835775	231	0	NE	0	NE	0	NE
M5005_Spy_0842c	-	835785	836429	214	0.00025	NE	0	NE	0.99	U
M5005_Spy_0843c	-	836681	837028	115	0	NE	0	NE	0	NE
M5005_Spy_0844c	nifS2	837018	838145	375	0.8785	U	1	E	1	E
M5005_Spy_0845c	prs	838142	839122	326	0	NE	0	NE	0.001	NE
M5005_Spy_0846c	-	839262	839840	192	0	NE	0	NE	0	NE
M5005_Spy_0847	-	839928	840599	223	0.00025	NE	0	NE	0.001	NE
M5005_Spy_0848	ppnK	840574	841410	278	1	E	1	E	1	E
M5005_Spy_0849	rluD	841407	842312	301	0	NE	0	NE	0	NE
M5005_Spy_0851	pta/eutD	842489	843310	273	0	NE	0	NE	0	NE
M5005_Spy_0852	-	843437	844054	205	0	NE	0	NE	0	NE
M5005_Spy_0853	-	844051	844209	52	-1	S	-1	S	-1	S
M5005_Spy_0854c	-	844403	845092	229	0	NE	0	NE	0	NE
M5005_Spy_0855	proV	845512	846240	242	0	NE	0	NE	0	NE
M5005_Spy_0856	proX	846233	847759	508	0	NE	0	NE	0	NE
M5005_Spy_0857	guaC	848037	849020	327	0	NE	0	NE	0	NE
M5005_Spy_0858	xpt	849325	849906	193	0	NE	0	NE	0	NE
M5005_Spy_0859	-	849906	851189	427	0.00025	NE	0	NE	0	NE
M5005_Spy_0860c	apbE	851253	852191	312	0	NE	0	NE	0	NE
M5005_Spy_0861c	-	852244	852429	61	0	NE	0	NE	0	NE
M5005_Spy_0862	tdk2	852567	853136	189	0	NE	0	NE	0	NE
M5005_Spy_0863	prfA	853171	854250	359	1	E	1	E	1	E
M5005_Spy_0864	hemK	854250	855089	279	0	NE	0	NE	0	NE
M5005_Spy_0865	-	855073	855663	196	0.00475	NE	0.00475	NE	0.665	U
M5005_Spy_0866	-	855681	856133	150	0	NE	0	NE	0	NE
M5005_Spy_0867	glyA	856123	857379	418	0	NE	0	NE	0	NE
M5005_Spy_0868	-	857386	858363	325	0.00025	NE	0	NE	0.001	NE
M5005_Spy_0869	-	858364	858963	199	0	NE	0	NE	0	NE
M5005_Spy_0870	-	858973	860697	574	0	NE	0	NE	0	NE
M5005_Spy_0871	-	860694	862421	575	0.00025	NE	0	NE	0	NE
M5005_Spy_0872	nox	862962	864032	356	0	NE	0	NE	0	NE
M5005_Spy_0873c	ldh	864191	865174	327	0.00025	NE	0	NE	0.002	NE
M5005_Spy_0874	gyrA	865365	867851	828	1	E	1	E	1	E
M5005_Spy_0875	-	867871	868620	249	0.9985	E	1	E	1	E
M5005_Spy_0876	-	868700	869116	138	0	NE	0	NE	0	NE
M5005_Spy_0877	-	869603	869722	39	-1	S	-1	S	-1	S
M5005_Spy_0878	-	869730	870098	122	0.0005	NE	0	NE	0.001	NE
M5005_Spy_0879c	-	870148	871059	303	0	NE	0	NE	0	NE
M5005_Spy_0880c	hlyIII	871176	871826	216	0.00025	NE	0.00025	NE	1	E
M5005_Spy_0881c	-	871823	872263	146	0.00025	NE	0	NE	0.001	NE
M5005_Spy_0882	rbgA	872482	873330	282	1	E	1	E	1	E
M5005_Spy_0883	rnhB	873320	874111	263	0.00025	NE	0	NE	0	NE
M5005_Spy_0884	smf	874176	875012	278	0.00025	NE	0	NE	0	NE
M5005_Spy_0885	topA	875119	877248	709	0	NE	0.069	U	0.995	E
M5005_Spy_0886c	-	877323	877805	160	0	NE	0	NE	0	NE
M5005_Spy_0887c	-	877812	877934	40	-1	S	-1	S	-1	S
M5005_Spy_0888c	-	878080	878205	41	-1	S	-1	S	-1	S
M5005_Spy_0889	-	878218	878763	181	0	NE	0	NE	0	NE
M5005_Spy_0890	ddh	878778	879770	330	0	NE	0	NE	0	NE
M5005_Spy_0891	satD	879875	880540	221	1	E	0.99975	E	1	E

M5005_Spy_0892	satE	880533	881249	238	0	NE	0	NE	0	NE
M5005_Spy_0893	gid	881400	882746	448	0.00025	NE	0	NE	0.389	U
M5005_Spy_0894	oadA2	882899	884305	468	0	NE	0	NE	0	NE
M5005_Spy_0895	-	884348	884662	104	0.00025	NE	0	NE	0.001	NE
M5005_Spy_0896	-	884662	885012	116	0	NE	0	NE	0	NE
M5005_Spy_0897	-	885022	886152	376	0	NE	0	NE	0	NE
M5005_Spy_0898c	-	886352	887236	294	0	NE	0	NE	0	NE
M5005_Spy_0899c	citG	887229	887924	231	0	NE	0	NE	0	NE
M5005_Spy_0900c	-	888082	889488	468	0	NE	0	NE	0	NE
M5005_Spy_0901	-	889865	890188	107	0	NE	0	NE	0	NE
M5005_Spy_0902	-	890242	890640	132	0	NE	0	NE	0	NE
M5005_Spy_0903	oadB	890685	891806	373	0	NE	0	NE	0	NE
M5005_Spy_0904	-	891824	891958	44	-1	S	-1	S	-1	S
M5005_Spy_0905	citD	891994	892302	102	0	NE	0	NE	0	NE
M5005_Spy_0906	citE	892320	893177	285	0	NE	0	NE	0	NE
M5005_Spy_0907	citF	893180	894712	510	0.00025	NE	0	NE	0.836	U
M5005_Spy_0908	citX	894642	895283	213	0	NE	0	NE	0	NE
M5005_Spy_0909	oadA1	895298	896692	464	0.00025	NE	0.0005	NE	0.001	NE
M5005_Spy_0910c	citC	896802	897854	350	0	NE	0	NE	0	NE
M5005_Spy_0911c	-	897943	898425	160	0	NE	0	NE	0	NE
M5005_Spy_0912	-	898910	899014	34	-1	S	-1	S	-1	S
M5005_Spy_0913c	xerS	899125	900039	304	0.8115	U	0.61075	U	1	E
M5005_Spy_0914	-	900733	901362	209	0.00025	NE	0	NE	0.001	NE
M5005_Spy_0915c	ffh	901479	903041	520	0.98975	U	1	E	0.999	E
M5005_Spy_0916c	ylxM	903056	903397	113	0.00025	NE	0.00075	NE	1	E
M5005_Spy_0917c	-	903486	904184	232	0.00025	NE	0.00025	NE	1	E
M5005_Spy_0918c	-	904267	905700	477	0	NE	0	NE	0	NE
M5005_Spy_0919	guaA	905867	907429	520	0.49925	U	0.001	NE	1	E
M5005_Spy_0920c	murM2	907471	908694	407	0	NE	0	NE	0	NE
M5005_Spy_0921c	-	909059	910603	514	0.00025	NE	0	NE	0	NE
M5005_Spy_0922c	pdxK	910737	911294	185	0	NE	0	NE	0	NE
M5005_Spy_0923c	-	911272	912138	288	0	NE	0	NE	0	NE
M5005_Spy_0924	-	912228	913496	422	0	NE	0	NE	0	NE
M5005_Spy_0925c	rnhB	913894	914250	118	0	NE	0	NE	0	NE
M5005_Spy_0926c	-	914572	916149	525	0.00025	NE	0	NE	0.148	U
M5005_Spy_0927c	fhs.1	916234	917904	556	1	E	1	E	1	E
M5005_Spy_0928c	lplA	918032	919051	339	0	NE	0	NE	0	NE
M5005_Spy_0929c	-	919098	919979	293	0	NE	0	NE	0	NE
M5005_Spy_0930c	-	919972	920784	270	0	NE	0	NE	0	NE
M5005_Spy_0931c	-	920777	921109	110	0	NE	0	NE	0	NE
M5005_Spy_0932c	-	921151	922149	332	0	NE	0	NE	0	NE
M5005_Spy_0933c	-	922146	923345	399	0.00025	NE	0	NE	0	NE
M5005_Spy_0934c	-	923342	924178	278	0	NE	0	NE	0	NE
M5005_Spy_0935	dpfB	924374	925066	230	0	NE	0.00025	NE	1	E
M5005_Spy_0936	dfp	925059	925604	181	0.00025	NE	0.996	E	1	E
M5005_Spy_0937	-	925662	926231	189	0.00025	NE	0	NE	0.001	NE
M5005_Spy_0938	pgmA	926407	928125	572	1	E	1	E	1	E
M5005_Spy_0939c	-	928338	929294	318	0	NE	0	NE	0	NE
M5005_Spy_0940c	-	929296	930360	354	0.00025	NE	0	NE	0	NE
M5005_Spy_0941c	-	930353	931903	516	0	NE	0	NE	0	NE
M5005_Spy_0942c	-	932024	933076	350	0	NE	0	NE	0	NE
M5005_Spy_0943c	cdd	933170	933559	129	0	NE	0	NE	0	NE
M5005_Spy_0944c	-	934218	934805	195	0	NE	0	NE	0	NE
M5005_Spy_0945	coaA	935073	935993	306	1	E	1	E	1	E
M5005_Spy_0946	rpsT	936062	936295	77	0.0005	NE	0	NE	0	NE
M5005_Spy_0947c	ciaH	936420	937730	436	0.0005	NE	0	NE	0.001	NE
M5005_Spy_0948c	ciaR	937723	938397	224	0	NE	0	NE	0.004	NE
M5005_Spy_0949c	pepN	938743	941280	845	0	NE	0	NE	0	NE
M5005_Spy_0950c	phoU	941485	942138	217	0.00025	NE	0	NE	0.086	U
M5005_Spy_0951c	pstB	942206	942964	252	0.00025	NE	0	NE	0	NE
M5005_Spy_0952c	pstB2	942977	943780	267	0.00025	NE	0	NE	0	NE
M5005_Spy_0953c	pstA	943796	944683	295	0.00025	NE	0	NE	0	NE
M5005_Spy_0954c	pstC	944673	945491	272	0	NE	0.0625	U	0.001	NE

M5005_Spy_0955c	pstS	945618	946484	288	0.00025	NE	0	NE	0	NE
M5005_Spy_0956c	-	946623	947933	436	0.00025	NE	1	E	1	E
M5005_Spy_0957c	-	947936	948724	262	0.04525	NE	0	NE	0.001	NE
M5005_Spy_0958c	-	948714	948992	92	0	NE	0	NE	0	NE
M5005_Spy_0959c	spxA	948994	949398	134	0	NE	0	NE	0	NE
M5005_Spy_0960c	mreA	949441	950373	310	1	E	1	E	1	E
M5005_Spy_0961c	truB	950402	951286	294	0.00025	NE	0	NE	0	NE
M5005_Spy_0962c	-	951402	952742	446	0	NE	0	NE	0	NE
M5005_Spy_0963c	-	952839	953795	318	0	NE	0	NE	0	NE
M5005_Spy_0964c	-	953806	954402	198	0	NE	0	NE	0	NE
M5005_Spy_0965c	-	954494	955669	391	0	NE	0	NE	0	NE
M5005_Spy_0967c	-	957146	957847	233	0	NE	0	NE	0	NE
M5005_Spy_0968	-	957965	958507	180	0	NE	0	NE	0	NE
M5005_Spy_0969c	-	958504	958647	47	-1	S	-1	S	-1	S
M5005_Spy_0970c	-	958650	959291	213	0	NE	0	NE	0	NE
M5005_Spy_0971c	-	959454	959942	162	0.00025	NE	0	NE	0.001	NE
M5005_Spy_0972c	-	959953	960153	66	0	NE	0	NE	0	NE
M5005_Spy_0973c	-	960194	960733	179	0	NE	0	NE	0	NE
M5005_Spy_0974c	-	960746	960934	62	0.148	U	0	NE	0.003	NE
M5005_Spy_0975c	-	960945	961532	195	0	NE	0	NE	0	NE
M5005_Spy_0976c	-	961593	961799	68	0	NE	0	NE	0.001	NE
M5005_Spy_0977c	pcrA	962205	964523	772	0.0245	NE	0.006	NE	1	E
M5005_Spy_0978	-	964891	965061	56	0.00025	NE	0.0415	NE	1	E
M5005_Spy_0979c	-	965052	966374	440	-1	S	-1	S	-1	S
M5005_Spy_0980	-	966494	967729	411	0.00025	NE	0	NE	0	NE
M5005_Spy_0981c	cfa	968098	968871	257	0	NE	0	NE	0	NE
M5005_Spy_0982c	-	969241	970077	278	0	NE	0	NE	0	NE
M5005_Spy_0983c	-	970093	970722	209	0.06425	U	0	NE	0	NE
M5005_Spy_0984c	-	970732	971373	213	0	NE	0	NE	0	NE
M5005_Spy_0985c	-	971479	971814	111	0	NE	0	NE	0	NE
M5005_Spy_0986c	glmS	972010	973824	604	1	E	1	E	1	E
M5005_Spy_0987c	sipC	974000	974557	185	0	NE	0	NE	0	NE
M5005_Spy_0988c	pyk	974775	976277	500	0.999	E	1	E	1	E
M5005_Spy_0989c	pfkA	976340	977353	337	0.99325	U	1	E	1	E
M5005_Spy_0990c	dnaE	977433	980543	1036	1	E	1	E	1	E
M5005_Spy_0991	-	980728	981099	123	0.00025	NE	0.00075	NE	0.747	U
M5005_Spy_0992	-	981099	981797	232	0	NE	0	NE	0	NE
M5005_Spy_0993	-	981807	982592	261	0	NE	0	NE	0	NE
M5005_Spy_0994c	-	982719	983333	204	0.00025	NE	0.00025	NE	1	E
M5005_Spy_0995c	-	983924	984112	62	0	NE	0	NE	0	NE
M5005_Spy_0996	speA2	984332	985087	251	0	NE	0	NE	0	NE
M5005_Spy_0997c	-	985209	985868	219	0	NE	0	NE	0	NE
M5005_Spy_0998c	-	985868	986089	73	0	NE	0	NE	0	NE
M5005_Spy_0999c	-	986099	986872	257	0.00025	NE	0	NE	0	NE
M5005_Spy_1000c	-	986883	987485	200	0	NE	0	NE	0	NE
M5005_Spy_1001c	-	987497	988261	254	0	NE	0	NE	0	NE
M5005_Spy_1002c	-	988263	988595	110	0	NE	0	NE	0	NE
M5005_Spy_1003c	-	988595	988963	122	0	NE	0	NE	0	NE
M5005_Spy_1004c	-	988932	989096	54	-1	S	-1	S	-1	S
M5005_Spy_1005c	-	989068	989415	115	0	NE	0	NE	0	NE
M5005_Spy_1006c	-	989426	991177	583	0	NE	0	NE	0	NE
M5005_Spy_1007c	-	991293	994733	1146	0.00025	NE	0	NE	0	NE
M5005_Spy_1008c	-	994734	996218	494	0	NE	0	NE	0	NE
M5005_Spy_1009c	-	996219	998024	601	0	NE	0	NE	0	NE
M5005_Spy_1010c	-	998017	998475	152	0	NE	0	NE	0.833	U
M5005_Spy_1011c	-	998448	998765	105	0.00025	NE	0	NE	0	NE
M5005_Spy_1012c	-	998778	999284	168	0	NE	0	NE	0	NE
M5005_Spy_1013c	-	999296	999706	136	0.00025	NE	0	NE	0	NE
M5005_Spy_1014c	-	999708	1000103	131	0	NE	0	NE	0	NE
M5005_Spy_1015c	-	1000100	1000411	103	0.00025	NE	0	NE	0.001	NE
M5005_Spy_1016c	-	1000408	1000752	114	0.00025	NE	0	NE	0	NE
M5005_Spy_1017c	-	1000766	1001059	97	0	NE	0	NE	0	NE
M5005_Spy_1018c	-	1001072	1001962	296	0	NE	0	NE	0	NE

M5005_Spy_1019c	-	1001981	1002550	189	0.00025	NE	0	NE	0	NE
M5005_Spy_1020c	-	1002795	1003064	89	0	NE	0	NE	0	NE
M5005_Spy_1021c	-	1003071	1003979	302	0	NE	0	NE	0	NE
M5005_Spy_1022c	-	1003948	1005273	441	0	NE	0	NE	0	NE
M5005_Spy_1023c	-	1005273	1006547	424	0	NE	0	NE	0	NE
M5005_Spy_1024c	-	1006537	1006917	126	0.00025	NE	0	NE	0	NE
M5005_Spy_1025c	-	1007527	1007961	144	0	NE	0	NE	0	NE
M5005_Spy_1026c	-	1008247	1008513	88	0	NE	0	NE	0	NE
M5005_Spy_1027c	-	1008510	1009034	174	0	NE	0	NE	0	NE
M5005_Spy_1028c	-	1009037	1009669	210	0	NE	0	NE	0	NE
M5005_Spy_1029c	-	1009671	1009955	94	0	NE	0	NE	0	NE
M5005_Spy_1030c	-	1009952	1010146	64	0	NE	0	NE	0	NE
M5005_Spy_1031c	-	1010119	1010358	79	0	NE	0	NE	0	NE
M5005_Spy_1032c	-	1010355	1010600	81	0.00025	NE	0	NE	0	NE
M5005_Spy_1033c	-	1010597	1010953	118	0	NE	0	NE	0	NE
M5005_Spy_1034c	-	1010950	1011390	146	0	NE	0	NE	0	NE
M5005_Spy_1035c	-	1011390	1011593	67	0	NE	0	NE	0	NE
M5005_Spy_1036c	ssb2	1011599	1012024	141	0	NE	0	NE	0	NE
M5005_Spy_1037c	ssb1	1012017	1012691	224	0.00025	NE	0	NE	0.001	NE
M5005_Spy_1038c	-	1012692	1013174	160	0	NE	0	NE	0	NE
M5005_Spy_1039c	-	1013196	1013450	84	0	NE	0	NE	0	NE
M5005_Spy_1040c	-	1013431	1013784	117	0	NE	0	NE	0	NE
M5005_Spy_1041c	-	1013797	1013934	45	-1	S	-1	S	-1	S
M5005_Spy_1042c	-	1013925	1014707	260	0.91875	U	0	NE	0.333	U
M5005_Spy_1043c	-	1014694	1015524	276	0	NE	0	NE	0	NE
M5005_Spy_1044	-	1015738	1016199	153	0.00025	NE	0	NE	0.001	NE
M5005_Spy_1045c	-	1016649	1016849	66	-1	S	-1	S	-1	S
M5005_Spy_1046	-	1016923	1017309	128	0.00025	NE	0	NE	0	NE
M5005_Spy_1047c	-	1017298	1017507	69	0	NE	0	NE	0	NE
M5005_Spy_1048	-	1017561	1018160	199	0.00025	NE	0	NE	0	NE
M5005_Spy_1049c	-	1018190	1018348	52	-1	S	-1	S	-1	S
M5005_Spy_1050	-	1018705	1019529	274	0.0005	NE	0.0005	NE	1	E
M5005_Spy_1051	-	1019565	1020458	297	0	NE	0	NE	0	NE
M5005_Spy_1052	int.1	1020579	1021667	362	0	NE	0	NE	0	NE
M5005_Spy_1053	-	1021799	1021912	37	-1	S	-1	S	-1	S
M5005_Spy_1054	-	1022030	1022650	206	0	NE	0	NE	0	NE
M5005_Spy_1055c	glgP	1022907	1025171	754	0	NE	0	NE	0	NE
M5005_Spy_1056c	malM	1025206	1026699	497	0	NE	0	NE	0	NE
M5005_Spy_1057c	malR	1026814	1027833	339	0	NE	0	NE	0	NE
M5005_Spy_1058	malE	1027874	1029325	483	0	NE	0	NE	0	NE
M5005_Spy_1059	malF	1029830	1030960	376	0.00025	NE	0	NE	0.001	NE
M5005_Spy_1060	malG	1030960	1031796	278	0	NE	0	NE	0	NE
M5005_Spy_1061	-	1031951	1032751	266	0.00025	NE	0	NE	0	NE
M5005_Spy_1062c	malA	1032867	1033682	271	0	NE	0	NE	0	NE
M5005_Spy_1063c	malD	1033704	1034564	286	0.00025	NE	0	NE	0	NE
M5005_Spy_1064c	malC	1034561	1035868	435	0	NE	0	NE	0	NE
M5005_Spy_1065c	amyA	1035943	1038078	711	0	NE	0	NE	0	NE
M5005_Spy_1066c	amyB	1038104	1039807	567	0.00025	NE	0	NE	0	NE
M5005_Spy_1067c	malX	1039973	1041232	419	0	NE	0	NE	0	NE
M5005_Spy_1068	-	1041558	1041782	74	0	NE	0	NE	0	NE
M5005_Spy_1069c	-	1041847	1042830	327	0	NE	0	NE	0	NE
M5005_Spy_1070c	dltD	1042861	1044111	416	0	NE	0	NE	0.001	NE
M5005_Spy_1071c	dltC	1044104	1044343	79	0	NE	0	NE	0	NE
M5005_Spy_1072c	dltB	1044361	1045617	418	0	NE	0	NE	0	NE
M5005_Spy_1073c	dltA	1045614	1047152	512	0	NE	0	NE	0	NE
M5005_Spy_1074c	-	1047164	1047307	47	-1	S	-1	S	-1	S
M5005_Spy_1075c	uvrB	1047571	1049562	663	0	NE	0	NE	0	NE
M5005_Spy_1076	glnH	1049755	1051929	724	1	E	1	E	1	E
M5005_Spy_1077	glnQ.2	1051929	1052669	246	0.98675	U	0.97275	U	0.285	U
M5005_Spy_1078c	-	1052817	1052969	50	-1	S	-1	S	-1	S
M5005_Spy_1079c	-	1052966	1054345	459	0	NE	0	NE	0	NE
M5005_Spy_1080c	-	1054523	1054972	149	0	NE	0	NE	0	NE
M5005_Spy_1081c	-	1054969	1055304	111	0	NE	0	NE	0	NE

M5005_Spy_1082c	-	1055307	1055618	103	0	NE	0	NE	0	NE
M5005_Spy_1083c	-	1055641	1057635	664	0.014	NE	0	NE	0.197	U
M5005_Spy_1084c	-	1057741	1058835	364	0	NE	0	NE	0	NE
M5005_Spy_1085c	bglA.2	1058844	1060244	466	0	NE	0	NE	0	NE
M5005_Spy_1086	-	1060469	1061164	231	0	NE	0	NE	0	NE
M5005_Spy_1087c	-	1061169	1061327	52	-1	S	-1	S	-1	S
M5005_Spy_1088c	obgE	1061396	1062703	435	1	E	1	E	1	E
M5005_Spy_1089c	-	1062766	1062894	42	-1	S	-1	S	-1	S
M5005_Spy_1090	-	1063136	1063651	171	0	NE	0	NE	0	NE
M5005_Spy_1091	-	1063824	1064480	218	0	NE	0	NE	0	NE
M5005_Spy_1092c	rsuA	1064529	1065263	244	0	NE	0	NE	0	NE
M5005_Spy_1093	-	1065375	1065740	121	0	NE	0	NE	0	NE
M5005_Spy_1094c	-	1065860	1067080	406	0	NE	0	NE	0	NE
M5005_Spy_1095	-	1067382	1068938	518	0	NE	0	NE	0	NE
M5005_Spy_1096c	-	1069045	1069446	133	0	NE	0	NE	0	NE
M5005_Spy_1097c	-	1069531	1070043	170	0	NE	0	NE	0	NE
M5005_Spy_1098c	-	1070348	1071703	451	0	NE	0	NE	0	NE
M5005_Spy_1099c	psr	1071785	1073236	483	0	NE	0	NE	0	NE
M5005_Spy_1100c	aroK	1073444	1073935	163	0	NE	0	NE	0	NE
M5005_Spy_1101c	aroA1	1073928	1075220	430	0	NE	0	NE	0	NE
M5005_Spy_1102c	-	1075322	1076287	321	0.00025	NE	0	NE	0.001	NE
M5005_Spy_1103c	map	1076289	1077149	286	0.00025	NE	1	E	1	E
M5005_Spy_1104c	-	1077165	1078448	427	0	NE	0	NE	0.001	NE
M5005_Spy_1105c	-	1078457	1078999	180	0	NE	0	NE	0	NE
M5005_Spy_1106c	grab	1079237	1079890	217	0	NE	0	NE	0	NE
M5005_Spy_1107c	murZ	1080248	1081507	419	0	NE	0	NE	0.001	NE
M5005_Spy_1108c	metK2	1081681	1082877	398	0.98925	U	1	E	1	E
M5005_Spy_1109c	inlA	1083414	1085792	792	0.00025	NE	0	NE	0	NE
M5005_Spy_1110	birA	1085996	1086937	313	0	NE	1	E	1	E
M5005_Spy_1111c	-	1086912	1087220	102	0	NE	0	NE	0	NE
M5005_Spy_1112c	dnaX	1087210	1088880	556	1	E	1	E	1	E
M5005_Spy_1113c	-	1088880	1089377	165	0	NE	0	NE	0	NE
M5005_Spy_1114	-	1089522	1090331	269	0	NE	0	NE	0	NE
M5005_Spy_1115c	-	1090384	1090647	87	0	NE	0	NE	0	NE
M5005_Spy_1116c	udk	1090727	1091353	208	0	NE	0	NE	0	NE
M5005_Spy_1117	deaD2	1091451	1092536	361	0	NE	0	NE	0	NE
M5005_Spy_1118c	-	1092700	1093920	406	0.00025	NE	0	NE	0	NE
M5005_Spy_1119c	gapN	1094015	1095442	475	1	E	1	E	1	E
M5005_Spy_1120c	pstI	1095627	1097360	577	0	NE	0.9685	U	1	E
M5005_Spy_1121c	ptsH	1097365	1097628	87	0.97675	U	0	NE	0.998	E
M5005_Spy_1122	nrdH	1098021	1098239	72	0	NE	0	NE	0.001	NE
M5005_Spy_1123	nrdE.2	1098259	1100418	719	0	NE	0	NE	0	NE
M5005_Spy_1124	nrdF	1100751	1101710	319	0	NE	0	NE	0	NE
M5005_Spy_1125	-	1101685	1102998	437	0	NE	0	NE	0	NE
M5005_Spy_1129c	-	1104384	1105079	231	0	NE	0	NE	0	NE
M5005_Spy_1130c	-	1105098	1105859	253	0	NE	0	NE	0	NE
M5005_Spy_1131c	-	1105856	1106077	73	0.064	U	0	NE	0	NE
M5005_Spy_1132c	alaS	1106432	1109050	872	1	E	1	E	1	E
M5005_Spy_1133c	prsA	1109437	1110492	351	0	NE	0	NE	0	NE
M5005_Spy_1134c	-	1110555	1111262	235	0	NE	0	NE	0	NE
M5005_Spy_1135c	-	1111328	1112524	398	0	NE	0	NE	0	NE
M5005_Spy_1136c	pepB	1112900	1114705	601	0	NE	0	NE	0	NE
M5005_Spy_1137c	-	1114718	1115680	320	0	NE	0	NE	0	NE
M5005_Spy_1138c	-	1115975	1116691	238	0.00025	NE	0	NE	0	NE
M5005_Spy_1139c	nagB	1116810	1117514	234	0	NE	0	NE	0	NE
M5005_Spy_1140	queA	1117716	1118744	342	0	NE	0	NE	0.001	NE
M5005_Spy_1141	-	1118751	1119971	406	0.00025	NE	0	NE	0	NE
M5005_Spy_1142c	-	1120085	1120675	196	0	NE	0	NE	0	NE
M5005_Spy_1143c	-	1120672	1120920	82	0	NE	0	NE	0	NE
M5005_Spy_1144c	-	1120905	1121132	75	0	NE	0	NE	0	NE
M5005_Spy_1145c	sodA	1121299	1121904	201	0	NE	0	NE	0	NE
M5005_Spy_1146c	holA	1122001	1123041	346	1	E	1	E	1	E
M5005_Spy_1147c	comEC	1123112	1125355	747	0.00025	NE	0	NE	0	NE

M5005_Spy_1148c	comE	1125336	1125998	220	0.00025	NE	0	NE	0	NE
M5005_Spy_1149c	-	1126198	1126938	246	0.99725	E	1	E	1	E
M5005_Spy_1150	-	1127056	1127832	258	0	NE	0	NE	0	NE
M5005_Spy_1151	-	1127822	1128100	92	0	NE	0	NE	0	NE
M5005_Spy_1152c	kup	1128124	1129107	327	0	NE	0	NE	0	NE
M5005_Spy_1154c	deaD	1130251	1131870	539	0	NE	0	NE	0	NE
M5005_Spy_1155c	prfC	1132177	1133721	514	0.00025	NE	0	NE	0	NE
M5005_Spy_1156c	-	1133969	1134664	231	0	NE	0	NE	0	NE
M5005_Spy_1157c	murF	1134744	1136135	463	1	E	1	E	1	E
M5005_Spy_1158c	ddl	1136326	1137372	348	0.6055	U	1	E	1	E
M5005_Spy_1159c	recR	1137473	1138069	198	0.00025	NE	0	NE	0.001	NE
M5005_Spy_1160c	-	1138116	1138307	63	0	NE	0	NE	0	NE
M5005_Spy_1161c	fdhC	1138868	1139647	259	0	NE	0	NE	0	NE
M5005_Spy_1162c	-	1139771	1140313	180	0	NE	0	NE	0	NE
M5005_Spy_1163c	-	1140474	1140995	173	0	NE	0	NE	0	NE
M5005_Spy_1164c	gpmA	1141102	1141797	231	0.99975	E	1	E	0.481	U
M5005_Spy_1165	pyrD	1142036	1142971	311	0.00025	NE	0	NE	0	NE
M5005_Spy_1166c	-	1143026	1143199	57	-1	S	-1	S	-1	S
M5005_Spy_1167c	-	1143271	1145133	620	0	NE	0	NE	0	NE
M5005_Spy_1168c	-	1145664	1145846	60	-1	S	-1	S	-1	S
M5005_Spy_1169	spd3	1146085	1146885	266	0	NE	0	NE	0	NE
M5005_Spy_1170	-	1147096	1147590	164	0	NE	0	NE	0	NE
M5005_Spy_1171c	-	1147660	1148865	401	0	NE	0	NE	0	NE
M5005_Spy_1172c	-	1148981	1149208	75	0	NE	0	NE	0	NE
M5005_Spy_1173c	-	1149205	1149480	91	0	NE	0	NE	0	NE
M5005_Spy_1174c	-	1149490	1150107	205	0	NE	0	NE	0	NE
M5005_Spy_1175c	-	1150104	1150541	145	0	NE	0	NE	0	NE
M5005_Spy_1176c	-	1150553	1152421	622	0	NE	0	NE	0	NE
M5005_Spy_1177c	-	1152418	1153113	231	0	NE	0	NE	0	NE
M5005_Spy_1178c	-	1153110	1155467	785	0	NE	0	NE	0	NE
M5005_Spy_1179c	-	1155467	1155838	123	0	NE	0	NE	0	NE
M5005_Spy_1180c	-	1155853	1156116	87	0	NE	0	NE	0	NE
M5005_Spy_1181c	-	1156127	1156720	197	0	NE	0	NE	0	NE
M5005_Spy_1182c	-	1156732	1157067	111	0	NE	0	NE	0	NE
M5005_Spy_1183c	-	1157068	1157304	78	0	NE	0	NE	0	NE
M5005_Spy_1184c	-	1157297	1157635	112	0.00025	NE	0	NE	0	NE
M5005_Spy_1185c	-	1157595	1158017	140	0	NE	0	NE	0	NE
M5005_Spy_1186c	-	1158027	1158227	66	0	NE	0	NE	0	NE
M5005_Spy_1187c	-	1158227	1159138	303	0.00025	NE	0	NE	0	NE
M5005_Spy_1188c	-	1159163	1159624	153	0	NE	0	NE	0	NE
M5005_Spy_1189c	-	1159705	1161120	471	0.00025	NE	0	NE	0	NE
M5005_Spy_1190c	-	1161230	1161496	88	0	NE	0	NE	0	NE
M5005_Spy_1191c	-	1161535	1161750	71	0	NE	0	NE	0	NE
M5005_Spy_1192c	-	1161719	1161952	77	0	NE	0	NE	0	NE
M5005_Spy_1193c	-	1161949	1163442	497	0	NE	0	NE	0	NE
M5005_Spy_1194c	-	1163435	1164703	422	0.00025	NE	0	NE	0	NE
M5005_Spy_1195c	-	1164700	1165056	118	0.00025	NE	0	NE	0	NE
M5005_Spy_1196c	-	1165205	1165549	114	0	NE	0	NE	0	NE
M5005_Spy_1197c	-	1165658	1166077	139	0	NE	0	NE	0	NE
M5005_Spy_1198c	-	1166345	1166980	211	0	NE	0	NE	0	NE
M5005_Spy_1199c	-	1166982	1167251	89	0	NE	0	NE	0	NE
M5005_Spy_1200c	-	1167335	1167847	170	0.00025	NE	0	NE	0	NE
M5005_Spy_1201c	-	1167844	1168257	137	0	NE	0	NE	0	NE
M5005_Spy_1202c	-	1168363	1168530	55	-1	S	-1	S	-1	S
M5005_Spy_1203c	-	1168540	1169337	265	0	NE	0	NE	0	NE
M5005_Spy_1204c	-	1169334	1170263	309	0.00025	NE	0	NE	0.001	NE
M5005_Spy_1205c	-	1170266	1170595	109	0	NE	0	NE	0	NE
M5005_Spy_1206c	-	1170651	1170857	68	0	NE	0	NE	0	NE
M5005_Spy_1207c	-	1170866	1171006	46	-1	S	-1	S	-1	S
M5005_Spy_1208c	-	1171003	1171236	77	0	NE	0	NE	0	NE
M5005_Spy_1209c	-	1171217	1171606	129	0.00025	NE	0	NE	0	NE
M5005_Spy_1210c	-	1171751	1171990	79	0	NE	0	NE	0.001	NE
M5005_Spy_1211c	-	1172090	1172275	61	-1	S	-1	S	-1	S

M5005_Spy_1212c	xis	1172277	1172588	103	0.00025	NE	0	NE	0	NE
M5005_Spy_1213c	-	1172666	1172851	61	0.1975	U	0	NE	0	NE
M5005_Spy_1214	-	1173018	1173257	79	0	NE	0	NE	0	NE
M5005_Spy_1215	-	1173399	1174205	268	0.00025	NE	0	NE	0	NE
M5005_Spy_1216c	-	1174140	1174406	88	0	NE	0	NE	0	NE
M5005_Spy_1217c	-	1174438	1175154	238	0.00475	NE	0	NE	0.001	NE
M5005_Spy_1218c	-	1175166	1175357	63	0	NE	0	NE	0	NE
M5005_Spy_1219	-	1176511	1176858	115	0.99975	E	1	E	1	E
M5005_Spy_1220	-	1176862	1177242	126	0	NE	0	NE	0	NE
M5005_Spy_1221	-	1177254	1177520	88	0	NE	0	NE	0	NE
M5005_Spy_1222	int.2	1177644	1178786	380	0	NE	0	NE	0	NE
M5005_Spy_1223c	-	1178876	1179151	91	0.984	U	1	E	0.98	U
M5005_Spy_1224c	-	1179250	1179837	195	0	NE	0	NE	0	NE
M5005_Spy_1225c	-	1179815	1180657	280	0	NE	0	NE	0	NE
M5005_Spy_1226c	-	1180650	1181501	283	0	NE	0	NE	0	NE
M5005_Spy_1227c	-	1181717	1182658	313	0	NE	0	NE	0	NE
M5005_Spy_1228c	recN	1182830	1184491	553	0	NE	0	NE	0	NE
M5005_Spy_1229c	argR1	1184513	1184983	156	0	NE	0	NE	0	NE
M5005_Spy_1230c	-	1184970	1185797	275	0	NE	0	NE	0	NE
M5005_Spy_1231c	fps	1185790	1186662	290	0	NE	0	NE	0	NE
M5005_Spy_1232c	xseB	1186662	1186877	71	0.782	U	0	NE	0	NE
M5005_Spy_1233c	xseA	1186855	1188195	446	0.00025	NE	0	NE	0	NE
M5005_Spy_1234c	folD	1188348	1189202	284	0	NE	0.00025	NE	1	E
M5005_Spy_1235c	-	1189410	1191113	567	0	NE	0	NE	0	NE
M5005_Spy_1236c	phr	1191282	1192691	469	0	NE	0	NE	0	NE
M5005_Spy_1237c	artP/gtr	1192840	1193574	244	0.04	NE	0	NE	0	NE
M5005_Spy_1238c	artQ	1193574	1194260	228	0	NE	0	NE	0	NE
M5005_Spy_1239c	-	1194387	1194617	76	0.45	U	0.00425	NE	0.954	U
M5005_Spy_1240	clpE	1194915	1197197	760	0	NE	0	NE	0	NE
M5005_Spy_1241	mutT	1197325	1197780	151	0	NE	0	NE	0	NE
M5005_Spy_1242	-	1197831	1198133	100	0	NE	0	NE	0	NE
M5005_Spy_1243c	ileS	1198398	1201199	933	0.99775	E	1	E	1	E
M5005_Spy_1244c	divIVAS	1201472	1202230	252	0.00025	NE	1	E	1	E
M5005_Spy_1245c	-	1202240	1203031	263	0	NE	0	NE	0	NE
M5005_Spy_1246c	-	1203031	1203285	84	0.00025	NE	0	NE	0.161	U
M5005_Spy_1247c	-	1203290	1203958	222	0.00025	NE	0	NE	0.001	NE
M5005_Spy_1248c	-	1203958	1204629	223	0	NE	0.00025	NE	0.001	NE
M5005_Spy_1249c	ftsZ	1204632	1205951	439	0.9745	U	1	E	1	E
M5005_Spy_1250c	ftsA	1205975	1207339	454	1	E	1	E	1	E
M5005_Spy_1251c	divIB/ftsQ	1207551	1208699	382	0.00075	NE	0.7875	U	0.002	NE
M5005_Spy_1252c	murG	1208700	1209803	367	1	E	1	E	1	E
M5005_Spy_1253c	murD	1209782	1211140	452	1	E	1	E	1	E
M5005_Spy_1254c	-	1211510	1211761	83	0	NE	0	NE	0	NE
M5005_Spy_1255c	typA	1211883	1213724	613	0.00025	NE	0	NE	0.303	U
M5005_Spy_1256c	-	1213907	1214296	129	0	NE	0	NE	0	NE
M5005_Spy_1257c	glcK	1214306	1215277	323	0.00025	NE	0	NE	0	NE
M5005_Spy_1258c	-	1215282	1215485	67	0	NE	0	NE	0	NE
M5005_Spy_1259c	dpr	1215627	1216154	175	0	NE	0	NE	0	NE
M5005_Spy_1260	-	1216382	1217008	208	0	NE	0	NE	0	NE
M5005_Spy_1261c	-	1217090	1218169	359	0	NE	0	NE	0	NE
M5005_Spy_1262c	-	1218173	1218793	206	0	NE	0	NE	0	NE
M5005_Spy_1263c	-	1219088	1219207	39	-1	S	-1	S	-1	S
M5005_Spy_1264c	-	1219241	1219942	233	0	NE	0	NE	0	NE
M5005_Spy_1265c	-	1220035	1220265	76	0.00025	NE	0	NE	0	NE
M5005_Spy_1266c	-	1220631	1221668	345	0	NE	0	NE	0	NE
M5005_Spy_1267c	coaD	1221655	1222146	163	0.338	U	1	E	1	E
M5005_Spy_1268c	-	1222136	1222675	179	0	NE	0	NE	0	NE
M5005_Spy_1269c	asnA	1222798	1223790	330	0	NE	0	NE	0	NE
M5005_Spy_1270c	arcC	1224103	1225053	316	0.0005	NE	0	NE	0.001	NE
M5005_Spy_1271c	-	1225073	1226404	443	0	NE	0	NE	0.001	NE
M5005_Spy_1272c	-	1226421	1227914	497	0	NE	0	NE	0	NE
M5005_Spy_1273c	arcB	1228084	1229097	337	0	NE	0	NE	0	NE
M5005_Spy_1274c	-	1229137	1229565	142	0	NE	0	NE	0	NE

M5005_Spy_1275c	arcA	1229665	1230900	411	0	NE	0	NE	0	NE
M5005_Spy_1276c	-	1231174	1231854	226	0	NE	0	NE	0.001	NE
M5005_Spy_1277	ahrC.2/argR	1231996	1232469	157	0	NE	0	NE	0	NE
M5005_Spy_1278c	-	1232635	1233351	238	0	NE	0	NE	0	NE
M5005_Spy_1279c	-	1233365	1234444	359	0	NE	0	NE	0	NE
M5005_Spy_1280c	yesM	1234517	1236250	577	0.535	U	0	NE	0	NE
M5005_Spy_1281c	yesN	1236247	1236987	246	0	NE	0	NE	0.001	NE
M5005_Spy_1282c	msrA	1237075	1238181	368	0.00025	NE	0	NE	0.001	NE
M5005_Spy_1283c	tlpA	1238224	1238847	207	0	NE	0	NE	0	NE
M5005_Spy_1284c	ccdA	1238860	1239570	236	0	NE	0	NE	0	NE
M5005_Spy_1285c	-	1240173	1240466	97	0	NE	0	NE	0	NE
M5005_Spy_1286c	-	1240477	1241502	341	0	NE	0	NE	0	NE
M5005_Spy_1287c	-	1241499	1242173	224	0	NE	0	NE	0	NE
M5005_Spy_1288c	-	1242175	1243023	282	0	NE	0	NE	0	NE
M5005_Spy_1289c	-	1243028	1244923	631	0.00025	NE	0	NE	0	NE
M5005_Spy_1290c	-	1244923	1245651	242	0	NE	0	NE	0	NE
M5005_Spy_1291c	-	1245784	1248186	800	0	NE	0	NE	0	NE
M5005_Spy_1292c	valS	1248346	1250751	801	1	E	1	E	1	E
M5005_Spy_1293c	-	1250995	1251558	187	0	NE	0	NE	0	NE
M5005_Spy_1294c	-	1251555	1251734	59	-1	S	-1	S	-1	S
M5005_Spy_1295c	-	1252159	1252554	131	0	NE	0	NE	0	NE
M5005_Spy_1296c	-	1252572	1252826	84	0	NE	0	NE	0	NE
M5005_Spy_1297	aroA2	1253305	1254057	250	0.00025	NE	0	NE	0	NE
M5005_Spy_1298	aroB	1254113	1255186	357	0	NE	0	NE	0	NE
M5005_Spy_1299c	-	1255455	1255613	52	0	NE	0	NE	0	NE
M5005_Spy_1300c	-	1255621	1255926	101	0	NE	0	NE	0	NE
M5005_Spy_1301c	-	1255928	1256266	112	0	NE	0	NE	0	NE
M5005_Spy_1302c	-	1256319	1257074	251	0	NE	0	NE	0	NE
M5005_Spy_1303c	aroE	1257309	1258187	292	0	NE	0	NE	0	NE
M5005_Spy_1304c	lacZ	1258325	1261741	1138	0	NE	0	NE	0	NE
M5005_Spy_1305c	trxR	1261761	1263245	494	0	NE	0	NE	0.001	NE
M5005_Spy_1306c	trxS	1263245	1264969	574	0	NE	0	NE	0	NE
M5005_Spy_1307c	trxT	1264959	1265564	201	0	NE	0	NE	0	NE
M5005_Spy_1308c	-	1265870	1267315	481	0	NE	0	NE	0	NE
M5005_Spy_1309c	-	1267396	1268322	308	0	NE	0	NE	0	NE
M5005_Spy_1310c	-	1268332	1269282	316	0	NE	0	NE	0	NE
M5005_Spy_1311	-	1269478	1270356	292	0	NE	0	NE	0	NE
M5005_Spy_1312	-	1270500	1270622	40	-1	S	-1	S	-1	S
M5005_Spy_1313c	-	1270968	1272410	480	0	NE	0	NE	0	NE
M5005_Spy_1314c	hyl	1272434	1274128	564	0	NE	0	NE	0.001	NE
M5005_Spy_1315c	-	1274179	1275219	346	0.00025	NE	0	NE	0	NE
M5005_Spy_1316	-	1275352	1276638	428	0.002	NE	0	NE	0	NE
M5005_Spy_1317	-	1276653	1279358	901	0.001	NE	0	NE	0	NE
M5005_Spy_1318c	rocA	1279459	1280814	451	0	NE	0	NE	0	NE
M5005_Spy_1319c	-	1281479	1282834	451	0	NE	0	NE	0.001	NE
M5005_Spy_1320	recX	1282949	1283725	258	0	NE	0	NE	0	NE
M5005_Spy_1321	-	1283805	1284338	177	0.00025	NE	0	NE	1	E
M5005_Spy_1322c	-	1284437	1284586	49	-1	S	-1	S	-1	S
M5005_Spy_1323	-	1284708	1284986	92	0	NE	0	NE	0	NE
M5005_Spy_1324	-	1291239	1291373	44	-1	S	-1	S	-1	S
M5005_Spy_1325c	-	1292030	1292578	182	0	NE	0	NE	0	NE
M5005_Spy_1326c	comFC	1292658	1293323	221	0.00025	NE	0	NE	0	NE
M5005_Spy_1327c	comFA	1293295	1294509	404	0	NE	0	NE	0	NE
M5005_Spy_1328	-	1294676	1295308	210	0.00025	NE	0	NE	0	NE
M5005_Spy_1329	cysM	1295436	1296377	313	0.00025	NE	0	NE	0	NE
M5005_Spy_1330c	-	1296395	1296772	125	0.00025	NE	0	NE	1	E
M5005_Spy_1331c	-	1296772	1298172	466	0	NE	0	NE	0	NE
M5005_Spy_1332c	yvqC	1298209	1298850	213	0	NE	0	NE	0.901	U
M5005_Spy_1333c	yvqE	1298843	1299847	334	0	NE	0	NE	0	NE
M5005_Spy_1334c	yvqF	1299844	1300536	230	0.00025	NE	0	NE	0	NE
M5005_Spy_1335c	-	1300659	1302557	632	1	E	1	E	1	E
M5005_Spy_1336c	pppL	1302554	1303294	246	0.003	NE	0.9995	E	1	E
M5005_Spy_1337c	sunL	1303332	1304654	440	0	NE	0	NE	0	NE

M5005_Spy_1338c	fmt	1304644	1305579	311	0.87975	U	1	E	1	E
M5005_Spy_1339c	priA	1305641	1308025	794	0.00025	NE	0	NE	0.997	E
M5005_Spy_1340c	-	1308090	1308407	105	0	NE	0	NE	0	NE
M5005_Spy_1341c	gmk	1308423	1309058	211	0.08825	U	1	E	0.998	E
M5005_Spy_1342c	-	1309168	1310775	535	0.10175	U	0	NE	0	NE
M5005_Spy_1343c	-	1310905	1311801	298	0	NE	0	NE	0	NE
M5005_Spy_1344	atoB	1312003	1313190	395	0	NE	0	NE	0	NE
M5005_Spy_1345	atoD.1	1313214	1313864	216	0	NE	0	NE	0	NE
M5005_Spy_1346	atoA	1313866	1314525	219	0.00025	NE	0	NE	0.001	NE
M5005_Spy_1347	-	1314558	1315337	259	0	NE	0	NE	0	NE
M5005_Spy_1348	-	1315408	1316739	443	0.00025	NE	0	NE	0	NE
M5005_Spy_1349	luxS	1316723	1317295	190	0.00025	NE	0	NE	0	NE
M5005_Spy_1350c	-	1317440	1318909	489	0	NE	0	NE	0	NE
M5005_Spy_1351c	-	1318923	1320077	384	0	NE	0	NE	0	NE
M5005_Spy_1352c	-	1320522	1320848	108	0.8855	U	0.808	U	0.066	U
M5005_Spy_1353c	-	1320970	1321485	171	0.00025	NE	0	NE	0	NE
M5005_Spy_1354	recU	1321566	1322165	199	0.00025	NE	0	NE	0.001	NE
M5005_Spy_1355	pbp1A	1322152	1324317	721	1	E	1	E	1	E
M5005_Spy_1356c	pepC	1324784	1326121	445	0	NE	0	NE	0	NE
M5005_Spy_1357c	nadE	1326306	1327130	274	0.948	U	1	E	1	E
M5005_Spy_1358c	nadE	1327132	1328586	484	0.999	E	1	E	1	E
M5005_Spy_1359c	-	1328757	1330136	459	0	NE	0	NE	0	NE
M5005_Spy_1360c	-	1330305	1331222	305	0.00025	NE	0	NE	1	E
M5005_Spy_1361c	aapA	1331286	1331510	74	0.00025	NE	1	E	1	E
M5005_Spy_1362c	-	1331614	1332360	248	0.00025	NE	0	NE	0	NE
M5005_Spy_1363c	-	1332357	1333160	267	0	NE	0	NE	0	NE
M5005_Spy_1364c	-	1333355	1334698	447	1	E	1	E	0.999	E
M5005_Spy_1365c	mraY	1334856	1335866	336	1	E	1	E	1	E
M5005_Spy_1366c	ftsI	1335868	1338123	751	0.98025	U	1	E	1	E
M5005_Spy_1367c	ftsL	1338127	1338450	107	0.00025	NE	1	E	1	E
M5005_Spy_1368c	mraW	1338455	1339468	337	0	NE	0	NE	1	E
M5005_Spy_1369c	-	1339492	1339599	35	-1	S	-1	S	-1	S
M5005_Spy_1370c	proA	1339942	1341192	416	0	NE	0	NE	0	NE
M5005_Spy_1371c	proB	1341185	1342006	273	0	NE	0	NE	0	NE
M5005_Spy_1372c	proB	1342071	1343699	542	0	NE	0	NE	0	NE
M5005_Spy_1373c	-	1343704	1344438	244	0	NE	0	NE	0	NE
M5005_Spy_1374c	-	1344472	1344738	88	0	NE	0	NE	0	NE
M5005_Spy_1375c	tkt	1344931	1346916	661	0	NE	0	NE	0.004	NE
M5005_Spy_1376c	tal	1347134	1347778	214	0	NE	0	NE	0	NE
M5005_Spy_1377c	-	1347904	1349403	499	0	NE	0	NE	0	NE
M5005_Spy_1378c	npx	1349393	1350739	448	0	NE	0	NE	0	NE
M5005_Spy_1379c	glpF	1350848	1351549	233	0	NE	0	NE	0	NE
M5005_Spy_1380c	glpO	1351551	1353389	612	0.00025	NE	0	NE	0	NE
M5005_Spy_1381c	glpK	1353405	1354931	508	0	NE	0	NE	0	NE
M5005_Spy_1382c	-	1355292	1355684	130	0	NE	0	NE	0	NE
M5005_Spy_1383c	-	1355811	1356068	85	0	NE	0	NE	0.306	U
M5005_Spy_1384c	glyS	1356222	1358261	679	0.998	E	1	E	1	E
M5005_Spy_1385c	glyQ	1358639	1359556	305	0.9735	U	1	E	1	E
M5005_Spy_1386c	-	1359928	1360461	177	0	NE	0	NE	0	NE
M5005_Spy_1387c	-	1360593	1361432	279	0	NE	0	NE	0	NE
M5005_Spy_1388c	nagA	1361554	1362702	382	0	NE	0	NE	0	NE
M5005_Spy_1389c	-	1362820	1364469	549	0	NE	0	NE	0	NE
M5005_Spy_1390	-	1364653	1365375	240	0	NE	0	NE	0	NE
M5005_Spy_1391c	-	1365504	1366346	280	0	NE	0	NE	0	NE
M5005_Spy_1392	-	1366639	1367196	185	0.00025	NE	0	NE	0	NE
M5005_Spy_1393c	-	1367232	1368056	274	0	NE	0	NE	0	NE
M5005_Spy_1394c	-	1368058	1368675	205	0	NE	0	NE	0	NE
M5005_Spy_1395c	lacD.1	1368872	1369849	325	0	NE	0	NE	0	NE
M5005_Spy_1396c	lacC1	1369999	1370349	116	0	NE	0	NE	0	NE
M5005_Spy_1397c	lacB.1	1370359	1370874	171	0	NE	0	NE	0	NE
M5005_Spy_1398c	lacA.1	1370889	1371314	141	0	NE	0	NE	0	NE
M5005_Spy_1399c	-	1371554	1373002	482	0	NE	0	NE	0	NE
M5005_Spy_1400c	-	1373031	1373336	101	0	NE	0	NE	0	NE

M5005_Spy_1401c	-	1373329	1373802	157	0	NE	0	NE	0	NE
M5005_Spy_1402	lacR.1	1374039	1374809	256	0	NE	0	NE	0	NE
M5005_Spy_1403c	-	1374836	1375006	56	0.00025	NE	0.0005	NE	0	NE
M5005_Spy_1404c	copZ	1375013	1375216	67	0	NE	0	NE	0	NE
M5005_Spy_1405c	copA	1375230	1377461	743	0	NE	0	NE	0	NE
M5005_Spy_1406c	copY	1377461	1377895	144	0	NE	0	NE	0	NE
M5005_Spy_1407	-	1378067	1379053	328	0	NE	0	NE	0	NE
M5005_Spy_1408c	rbfA	1379187	1379543	118	0.00025	NE	0	NE	0.992	E
M5005_Spy_1409c	infB	1379742	1382603	953	1	E	1	E	1	E
M5005_Spy_1410c	-	1382623	1382925	100	0.00025	NE	0.0005	NE	0.999	E
M5005_Spy_1411c	-	1382918	1383214	98	0.00225	NE	0.00125	NE	1	E
M5005_Spy_1412c	nusA	1383230	1384387	385	1	E	1	E	1	E
M5005_Spy_1413c	-	1384562	1385098	178	0	NE	0	NE	0.001	NE
M5005_Spy_1414c	-	1385343	1385522	59	-1	S	-1	S	-1	S
M5005_Spy_1415c	sdaD2	1385761	1386933	390	0	NE	0	NE	0	NE
M5005_Spy_1416c	-	1387049	1388245	398	0	NE	0	NE	0	NE
M5005_Spy_1417c	-	1388356	1388541	61	0	NE	0	NE	0	NE
M5005_Spy_1418c	-	1388538	1388837	99	0	NE	0	NE	0	NE
M5005_Spy_1419c	-	1388848	1389468	206	0	NE	0	NE	0	NE
M5005_Spy_1420c	-	1389471	1389632	53	-1	S	-1	S	-1	S
M5005_Spy_1421c	-	1389641	1391548	635	0	NE	0	NE	0.001	NE
M5005_Spy_1422c	-	1391559	1392194	211	0	NE	0	NE	0	NE
M5005_Spy_1423c	-	1392194	1393249	351	0	NE	0	NE	0	NE
M5005_Spy_1424c	-	1393246	1395228	660	0	NE	0	NE	0	NE
M5005_Spy_1425c	-	1395238	1396080	280	0	NE	0	NE	0	NE
M5005_Spy_1426c	-	1396092	1400474	1460	0	NE	0	NE	0	NE
M5005_Spy_1427c	-	1400489	1400722	77	0	NE	0	NE	0	NE
M5005_Spy_1428c	-	1400797	1401252	151	0	NE	0	NE	0	NE
M5005_Spy_1429c	-	1401306	1401905	199	0	NE	0	NE	0	NE
M5005_Spy_1430c	-	1401917	1402276	119	0.00025	NE	0	NE	0	NE
M5005_Spy_1431c	-	1402280	1402606	108	0	NE	0	NE	0	NE
M5005_Spy_1432c	-	1402621	1402899	92	0	NE	0	NE	0	NE
M5005_Spy_1433c	-	1402910	1403266	118	0.00025	NE	0	NE	0	NE
M5005_Spy_1434c	-	1403278	1404165	295	0	NE	0	NE	0	NE
M5005_Spy_1435c	-	1404178	1404747	189	0	NE	0	NE	0	NE
M5005_Spy_1436c	-	1404903	1405169	88	0	NE	0	NE	0	NE
M5005_Spy_1437c	-	1405172	1405360	62	0	NE	0	NE	0	NE
M5005_Spy_1438c	-	1405391	1406830	479	0	NE	0	NE	0	NE
M5005_Spy_1439c	-	1406796	1408328	510	0	NE	0	NE	0	NE
M5005_Spy_1440c	-	1408344	1409621	425	0	NE	0	NE	0	NE
M5005_Spy_1441c	-	1409611	1410105	164	0	NE	0	NE	0	NE
M5005_Spy_1442c	-	1410153	1410569	138	0	NE	0	NE	0	NE
M5005_Spy_1443c	-	1410566	1410757	63	0	NE	0	NE	0	NE
M5005_Spy_1444c	-	1410747	1411598	283	0.00025	NE	0	NE	0	NE
M5005_Spy_1445c	-	1411607	1411873	88	0	NE	0	NE	0	NE
M5005_Spy_1446c	-	1411870	1412037	55	-1	S	-1	S	-1	S
M5005_Spy_1447c	-	1412038	1413360	440	0	NE	0	NE	0	NE
M5005_Spy_1448c	-	1413357	1413632	91	0	NE	0	NE	0	NE
M5005_Spy_1449c	-	1414019	1416403	794	0	NE	0	NE	0	NE
M5005_Spy_1450c	-	1416408	1418330	640	0	NE	0	NE	0	NE
M5005_Spy_1451c	-	1418373	1418930	185	0	NE	0	NE	0	NE
M5005_Spy_1452c	-	1418941	1419339	132	0.00025	NE	0	NE	0	NE
M5005_Spy_1453c	-	1419343	1420497	384	0	NE	0	NE	0	NE
M5005_Spy_1454c	-	1420497	1420796	99	0.0285	NE	0	NE	0	NE
M5005_Spy_1455c	-	1420884	1421087	67	0.05025	U	0	NE	0	NE
M5005_Spy_1456c	-	1421084	1421236	50	-1	S	-1	S	-1	S
M5005_Spy_1457c	-	1421233	1421619	128	0.00025	NE	0	NE	0.001	NE
M5005_Spy_1458c	-	1421616	1421819	67	0	NE	0	NE	0.127	U
M5005_Spy_1459c	-	1421812	1421982	56	-1	S	-1	S	-1	S
M5005_Spy_1460c	-	1421979	1422254	91	0.424	U	0	NE	0.001	NE
M5005_Spy_1461c	-	1422316	1422531	71	0.001	NE	0.0005	NE	0	NE
M5005_Spy_1462	-	1422579	1422992	137	0.00025	NE	0	NE	0	NE
M5005_Spy_1463c	-	1422973	1423128	51	-1	S	-1	S	-1	S

M5005_Spy_1464	-	1423403	1423804	133	0.99925	E	0.99975	E	1	E
M5005_Spy_1465	-	1423818	1424201	127	0	NE	0	NE	0	NE
M5005_Spy_1466	-	1424212	1424763	183	0.00025	NE	0	NE	0	NE
M5005_Spy_1467	int.3	1424880	1425959	359	0	NE	0	NE	0	NE
M5005_Spy_1468c	trmB	1426157	1426792	211	0	NE	0	NE	0	NE
M5005_Spy_1469c	-	1426792	1427583	263	0.9995	E	1	E	1	E
M5005_Spy_1470c	-	1427647	1428681	344	0	NE	0	NE	0	NE
M5005_Spy_1471c	-	1428684	1429409	241	0	NE	0	NE	0	NE
M5005_Spy_1472	hit	1429481	1429900	139	0	NE	0	NE	0	NE
M5005_Spy_1473	-	1429897	1430253	118	0	NE	0	NE	0	NE
M5005_Spy_1474c	lytR	1430364	1431638	424	0	NE	0	NE	0	NE
M5005_Spy_1475c	-	1431647	1432171	174	0	NE	0	NE	0	NE
M5005_Spy_1476c	-	1432146	1432607	153	0.00025	NE	0	NE	0.924	U
M5005_Spy_1477c	-	1432761	1434221	486	0	NE	0	NE	0	NE
M5005_Spy_1478	-	1434485	1435297	270	0	NE	0	NE	0	NE
M5005_Spy_1479	manL	1435649	1436641	330	0.00025	NE	0	NE	0	NE
M5005_Spy_1480	manM	1436730	1437539	269	0.00025	NE	0	NE	0	NE
M5005_Spy_1481	manN	1437556	1438467	303	0	NE	0	NE	0	NE
M5005_Spy_1482	manO	1438581	1438940	119	0	NE	0	NE	0	NE
M5005_Spy_1483	serS	1439332	1440609	425	0.9995	E	1	E	1	E
M5005_Spy_1484c	accD	1440831	1441601	256	0.032	NE	1	E	1	E
M5005_Spy_1485c	accA	1441598	1442464	288	0.99925	E	1	E	1	E
M5005_Spy_1486c	accC	1442473	1443837	454	0.9925	U	1	E	1	E
M5005_Spy_1487c	fabZ	1443869	1444291	140	0	NE	0.00075	NE	1	E
M5005_Spy_1488c	accB	1444288	1444788	166	0.9815	U	1	E	1	E
M5005_Spy_1489c	fabF	1444790	1446022	410	1	E	1	E	1	E
M5005_Spy_1490c	fabG	1446037	1446771	244	0.00025	NE	1	E	0.994	E
M5005_Spy_1491c	fabD	1446761	1447699	312	0.00025	NE	1	E	1	E
M5005_Spy_1492c	fabK	1447724	1448695	323	0.00325	NE	0.15275	U	0.999	E
M5005_Spy_1493c	acpP	1448897	1449121	74	0.00025	NE	1	E	0.997	E
M5005_Spy_1494c	fabH	1449182	1450156	324	1	E	1	E	1	E
M5005_Spy_1495c	-	1450157	1450591	144	0	NE	0	NE	0.857	U
M5005_Spy_1496c	phaB	1450668	1451459	263	1	E	1	E	1	E
M5005_Spy_1497c	dnaJ	1451679	1452836	385	1	E	1	E	1	E
M5005_Spy_1498c	dnaK	1453117	1454943	608	1	E	1	E	1	E
M5005_Spy_1499c	grpE	1455124	1455657	177	0.99525	E	1	E	1	E
M5005_Spy_1500c	hrcA	1455699	1456733	344	0.00025	NE	0.00025	NE	1	E
M5005_Spy_1501c	-	1456868	1457437	189	0.00025	NE	0	NE	0.001	NE
M5005_Spy_1502c	-	1457434	1458177	247	0	NE	0	NE	0	NE
M5005_Spy_1503c	-	1458167	1458874	235	0	NE	0	NE	0	NE
M5005_Spy_1504c	-	1459265	1459513	82	0	NE	0	NE	0	NE
M5005_Spy_1505c	-	1459723	1459938	71	0	NE	0	NE	0	NE
M5005_Spy_1506c	gatB	1459935	1461374	479	1	E	1	E	1	E
M5005_Spy_1507c	gatA	1461374	1462840	488	1	E	1	E	1	E
M5005_Spy_1508c	gatC	1462840	1463142	100	0.97625	U	1	E	0.997	E
M5005_Spy_1509c	-	1463374	1463583	69	0	NE	0	NE	0	NE
M5005_Spy_1510c	-	1463555	1463671	38	-1	S	-1	S	-1	S
M5005_Spy_1511c	-	1464245	1464691	148	0	NE	0	NE	0	NE
M5005_Spy_1512c	codY	1464838	1465620	260	0.00575	NE	0.0005	NE	1	E
M5005_Spy_1513c	-	1465838	1467052	404	0	NE	0	NE	0	NE
M5005_Spy_1514	-	1467283	1467735	150	0.00025	NE	0	NE	0.001	NE
M5005_Spy_1515c	-	1467858	1469135	425	0.00025	NE	0	NE	0	NE
M5005_Spy_1516	asnB	1469318	1470283	321	0	NE	0	NE	0	NE
M5005_Spy_1517c	-	1470632	1471336	234	0	NE	0	NE	0	NE
M5005_Spy_1518c	-	1471349	1472251	300	0.00025	NE	0	NE	0	NE
M5005_Spy_1519c	recG	1472544	1474559	671	0	NE	0	NE	0	NE
M5005_Spy_1520	-	1474652	1474852	66	0.00275	NE	0	NE	0	NE
M5005_Spy_1521c	-	1474934	1476388	484	0	NE	0	NE	0	NE
M5005_Spy_1522c	-	1476325	1477005	226	0	NE	0	NE	0	NE
M5005_Spy_1523c	-	1477002	1477595	197	0	NE	0	NE	0	NE
M5005_Spy_1524c	cycC	1477592	1479262	556	0	NE	0	NE	0	NE
M5005_Spy_1525c	cycD	1479255	1481018	587	0	NE	0	NE	0	NE
M5005_Spy_1526c	fhuC	1481015	1481851	278	0	NE	0	NE	0	NE

M5005_Spy_1527c	fhuB2	1481848	1482870	340	0	NE	0	NE	0	NE
M5005_Spy_1528c	fhuD2	1482872	1483756	294	0	NE	0	NE	0	NE
M5005_Spy_1529c	shp	1483740	1484615	291	0	NE	0	NE	0	NE
M5005_Spy_1530c	-	1484812	1488639	1275	0	NE	0	NE	0	NE
M5005_Spy_1531c	isp2	1489128	1490639	503	0	NE	0	NE	0	NE
M5005_Spy_1532c	alr	1490726	1491826	366	0.99575	E	0.9555	U	1	E
M5005_Spy_1533c	acpS	1491823	1492179	118	0.999	E	0.05675	NE	1	E
M5005_Spy_1534c	secA	1492295	1494814	839	1	E	1	E	1	E
M5005_Spy_1535c	-	1494892	1494996	34	-1	S	-1	S	-1	S
M5005_Spy_1536c	-	1494980	1495495	171	0	NE	0	NE	0	NE
M5005_Spy_1537c	-	1495398	1496102	234	0	NE	0	NE	0	NE
M5005_Spy_1538c	pmi	1496264	1497217	317	0	NE	0	NE	0	NE
M5005_Spy_1539c	scrK	1497312	1498268	318	0	NE	0	NE	0	NE
M5005_Spy_1540c	endoS	1498460	1501447	995	0	NE	0	NE	0	NE
M5005_Spy_1541c	-	1501471	1501635	54	-1	S	-1	S	-1	S
M5005_Spy_1542c	scrA	1501678	1503561	627	0.00025	NE	0	NE	0	NE
M5005_Spy_1543	scrB	1503803	1505242	479	0.00025	NE	0.00025	NE	0.001	NE
M5005_Spy_1544	scrR	1505247	1506212	321	0	NE	0	NE	0	NE
M5005_Spy_1545c	nusB	1506353	1506805	150	0.00025	NE	0	NE	1	E
M5005_Spy_1546c	-	1506798	1507187	129	0	NE	0	NE	0.896	U
M5005_Spy_1547c	efp	1507233	1507790	185	0	NE	0	NE	0	NE
M5005_Spy_1548c	comEB	1507886	1508347	153	0	NE	0	NE	0	NE
M5005_Spy_1549c	pepP	1508382	1509455	357	0	NE	0	NE	0	NE
M5005_Spy_1550c	uvrA	1509570	1512428	952	0.00025	NE	0	NE	0	NE
M5005_Spy_1551	corA	1512601	1513545	314	0.00075	NE	1	E	1	E
M5005_Spy_1552	-	1513678	1514334	218	0	NE	0	NE	0	NE
M5005_Spy_1553c	rpsR	1514467	1514706	79	0.91925	U	0.997	E	0.99	U
M5005_Spy_1554c	ssb3	1514871	1515362	163	0.00025	NE	0	NE	0.295	U
M5005_Spy_1555c	rpsF	1515384	1515674	96	0.987	U	0	NE	0.172	U
M5005_Spy_1556c	-	1515847	1516140	97	0	NE	0	NE	0	NE
M5005_Spy_1557	mutY	1516461	1517462	333	0	NE	0	NE	0	NE
M5005_Spy_1558	-	1517538	1518125	195	0	NE	0	NE	0	NE
M5005_Spy_1559c	trx	1518177	1518491	104	0.00025	NE	0	NE	0.018	NE
M5005_Spy_1560c	-	1518572	1519075	167	0	NE	0	NE	0	NE
M5005_Spy_1561c	mutS2	1519076	1521415	779	0.0015	NE	0	NE	0	NE
M5005_Spy_1562c	-	1521564	1522109	181	0.00025	NE	0	NE	0	NE
M5005_Spy_1563c	-	1522112	1522420	102	0	NE	0	NE	0	NE
M5005_Spy_1564	-	1522577	1523479	300	0.1885	U	0.00025	NE	1	E
M5005_Spy_1565	spi	1523490	1524083	197	0.75	U	1	E	1	E
M5005_Spy_1566	recD	1524141	1526594	817	0	NE	0	NE	0	NE
M5005_Spy_1567	-	1526685	1527167	160	0.00025	NE	0	NE	0.001	NE
M5005_Spy_1568c	dinP	1527260	1528354	364	0	NE	0	NE	0	NE
M5005_Spy_1569	pfl	1528563	1530890	775	0	NE	0	NE	0	NE
M5005_Spy_1570c	-	1531068	1532018	316	0	NE	0	NE	0	NE
M5005_Spy_1571c	cppA	1532003	1532755	250	0	NE	0	NE	0	NE
M5005_Spy_1572	-	1533053	1533949	298	0	NE	0	NE	0.001	NE
M5005_Spy_1573c	glpF.2	1534285	1535133	282	0.00025	NE	0.00025	NE	1	E
M5005_Spy_1574c	-	1535303	1535449	48	-1	S	-1	S	-1	S
M5005_Spy_1575c	norA	1535635	1536831	398	0.00025	NE	0	NE	0	NE
M5005_Spy_1576	srv	1536937	1537656	239	0	NE	0	NE	0	NE
M5005_Spy_1577	pepXP	1537678	1539960	760	0	NE	0	NE	0	NE
M5005_Spy_1578c	-	1540040	1540261	73	0	NE	0	NE	0	NE
M5005_Spy_1579	-	1540431	1540805	124	0.00025	NE	0	NE	0	NE
M5005_Spy_1580	-	1540998	1541297	99	0	NE	0	NE	0	NE
M5005_Spy_1581c	-	1541354	1542091	245	0	NE	0	NE	0	NE
M5005_Spy_1582c	dnaQ	1542240	1542827	195	0	NE	0	NE	0	NE
M5005_Spy_1583c	-	1542876	1543406	176	0	NE	0	NE	0.001	NE
M5005_Spy_1584	-	1543612	1544781	389	0	NE	0	NE	0	NE
M5005_Spy_1585c	deoC	1544876	1545547	223	0.00025	NE	0	NE	0	NE
M5005_Spy_1586c	nupC	1545577	1546779	400	0	NE	0	NE	0	NE
M5005_Spy_1587c	udp	1546800	1547579	259	0.00025	NE	0	NE	0	NE
M5005_Spy_1588c	-	1547644	1547745	33	-1	S	-1	S	-1	S
M5005_Spy_1589	crgR	1547819	1548562	247	0	NE	0	NE	0	NE

M5005_Spy_1590	rpsN	1548798	1549067	89	0	NE	0	NE	0	NE
M5005_Spy_1591c	gcp	1549238	1550266	342	0.00025	NE	1	E	0.998	E
M5005_Spy_1592c	-	1550256	1550711	151	0	NE	0	NE	0	NE
M5005_Spy_1593c	-	1550683	1551381	232	0.00025	NE	0.6645	U	1	E
M5005_Spy_1594	-	1551666	1551896	76	0.00025	NE	1	E	1	E
M5005_Spy_1595	-	1551898	1553580	560	0.99875	E	1	E	1	E
M5005_Spy_1596c	glnA	1553807	1555153	448	0	NE	0	NE	0.001	NE
M5005_Spy_1597c	glnR	1555191	1555562	123	0	NE	0	NE	0	NE
M5005_Spy_1598c	-	1555629	1556180	183	0.00025	NE	0	NE	0.001	NE
M5005_Spy_1599c	pgk	1556443	1557639	398	0.999	E	1	E	1	E
M5005_Spy_1600c	lppC	1557832	1558686	284	0	NE	0	NE	0	NE
M5005_Spy_1601c	-	1558916	1559806	296	0	NE	0	NE	0	NE
M5005_Spy_1602c	-	1560043	1561707	554	1	E	1	E	1	E
M5005_Spy_1603c	asp	1561707	1562072	121	0	NE	0	NE	0.001	NE
M5005_Spy_1604c	-	1562097	1562234	45	-1	S	-1	S	-1	S
M5005_Spy_1605c	-	1562239	1563372	377	0	NE	0	NE	0	NE
M5005_Spy_1606c	rpmB	1563550	1563738	62	0.00175	NE	0	NE	0	NE
M5005_Spy_1607c	fba	1564120	1565001	293	1	E	1	E	1	E
M5005_Spy_1608c	-	1565347	1566273	308	0	NE	0	NE	0	NE
M5005_Spy_1610c	pyrG	1567892	1568044	50	0	NE	0	NE	0	NE
M5005_Spy_1611c	rpoE	1568301	1568876	191	0	NE	0	NE	0	NE
M5005_Spy_1612c	tig/ropA	1569093	1570376	427	0.00025	NE	0	NE	0	NE
M5005_Spy_1613	-	1570697	1571542	281	0	NE	0	NE	0.001	NE
M5005_Spy_1614c	-	1571607	1572167	186	0	NE	0	NE	0	NE
M5005_Spy_1615c	-	1572181	1572651	156	0	NE	0	NE	0	NE
M5005_Spy_1616c	thiD	1572641	1573405	254	0	NE	0	NE	0	NE
M5005_Spy_1617c	truA	1573395	1574144	249	0	NE	0	NE	0	NE
M5005_Spy_1618c	comX.2	1574329	1574880	183	0	NE	0	NE	0	NE
M5005_Spy_1619	-	1580302	1580436	44	-1	S	-1	S	-1	S
M5005_Spy_1620c	-	1580598	1581740	380	0.00025	NE	0	NE	0	NE
M5005_Spy_1621	hsdR	1582022	1585000	992	0	NE	0	NE	0	NE
M5005_Spy_1622	hsdS	1585013	1586212	399	0	NE	0	NE	0	NE
M5005_Spy_1623	hsdM	1586225	1587805	526	0.00025	NE	0	NE	0	NE
M5005_Spy_1624c	-	1588015	1588206	63	0	NE	0	NE	0	NE
M5005_Spy_1625c	salR	1588358	1588963	201	0	NE	0	NE	0	NE
M5005_Spy_1626c	salK	1588944	1590506	520	0	NE	0	NE	0	NE
M5005_Spy_1627c	salY	1590546	1592453	635	0	NE	0	NE	0	NE
M5005_Spy_1628c	salX	1592455	1593192	245	0	NE	0	NE	0	NE
M5005_Spy_1629c	salX/salT	1593189	1593650	153	0	NE	0	NE	0	NE
M5005_Spy_1630c	salB	1593724	1595349	541	0	NE	0	NE	0	NE
M5005_Spy_1631c	salA	1595432	1595578	48	-1	S	-1	S	-1	S
M5005_Spy_1632c	lacG	1596090	1597496	468	0	NE	0	NE	0	NE
M5005_Spy_1633c	lacE	1597584	1599281	565	0.00075	NE	0.017	NE	0	NE
M5005_Spy_1634c	lacF	1599281	1599598	105	0	NE	0	NE	0	NE
M5005_Spy_1635c	lacD.2	1599622	1600605	327	0	NE	0	NE	0	NE
M5005_Spy_1636c	lacC.2	1600609	1601538	309	0	NE	0	NE	0	NE
M5005_Spy_1637c	lacB.2	1601586	1602101	171	0	NE	0	NE	0	NE
M5005_Spy_1638c	lacA.2	1602136	1602564	142	0	NE	0	NE	0	NE
M5005_Spy_1639	lacR.2	1603011	1603784	257	0	NE	0	NE	0	NE
M5005_Spy_1640	-	1604445	1604732	95	0	NE	0	NE	0	NE
M5005_Spy_1641	-	1604722	1605057	111	0	NE	0	NE	0	NE
M5005_Spy_1642	-	1605209	1605391	60	0	NE	0	NE	0.003	NE
M5005_Spy_1643	-	1605380	1605565	61	-1	S	-1	S	-1	S
M5005_Spy_1644c	-	1605479	1605679	66	-1	S	-1	S	-1	S
M5005_Spy_1645	-	1606013	1606162	49	-1	S	-1	S	-1	S
M5005_Spy_1646c	rpsI	1606282	1606674	130	0.0005	NE	0.0005	NE	0.445	U
M5005_Spy_1647c	rplM	1606695	1607141	148	0.00025	NE	0.9995	E	0.001	NE
M5005_Spy_1648c	-	1607359	1607565	68	0.0215	NE	0	NE	0.074	U
M5005_Spy_1649c	-	1607562	1608068	168	0	NE	0	NE	0	NE
M5005_Spy_1650c	-	1608204	1609064	286	0	NE	0	NE	0.001	NE
M5005_Spy_1651c	-	1609161	1609679	172	0	NE	0	NE	0	NE
M5005_Spy_1652c	-	1609683	1610429	248	0	NE	0	NE	0	NE
M5005_Spy_1653c	-	1610476	1611273	265	0	NE	0	NE	0	NE

M5005_Spy_1654c	-	1611469	1611882	137	0	NE	0	NE	0	NE
M5005_Spy_1655c	cysS	1611875	1613218	447	1	E	1	E	0.992	E
M5005_Spy_1656c	-	1613246	1613476	76	0	NE	0	NE	0	NE
M5005_Spy_1657c	-	1613489	1613761	90	0	NE	0	NE	0	NE
M5005_Spy_1658c	cysE	1613955	1614536	193	0.958	U	0.9995	E	0.791	U
M5005_Spy_1659c	-	1614545	1615297	250	0.00025	NE	0	NE	0.001	NE
M5005_Spy_1660c	pnp	1615290	1617422	710	0	NE	0	NE	0	NE
M5005_Spy_1661c	-	1617703	1618431	242	0	NE	0	NE	0	NE
M5005_Spy_1662c	ulaA	1618587	1619822	411	0	NE	0	NE	0	NE
M5005_Spy_1663c	-	1619849	1620133	94	0.00025	NE	0	NE	0	NE
M5005_Spy_1664c	-	1620126	1622186	686	0	NE	0	NE	0	NE
M5005_Spy_1665c	-	1622411	1622554	47	-1	S	-1	S	-1	S
M5005_Spy_1666c	rpsO	1622538	1622807	89	0.65075	U	0	NE	0.755	U
M5005_Spy_1667c	-	1622965	1623120	51	-1	S	-1	S	-1	S
M5005_Spy_1668	-	1623246	1623515	89	0	NE	0	NE	0	NE
M5005_Spy_1669	def	1623616	1624230	204	0.93575	U	1	E	1	E
M5005_Spy_1670c	-	1624264	1624806	180	0	NE	0	NE	0	NE
M5005_Spy_1671c	-	1624937	1625365	142	0	NE	0	NE	0	NE
M5005_Spy_1672c	polC	1625475	1629872	1465	1	E	1	E	1	E
M5005_Spy_1673c	proS	1630127	1631983	618	1	E	1	E	1	E
M5005_Spy_1674c	eep	1632181	1633440	419	0	NE	0	NE	0	NE
M5005_Spy_1675c	cdsA	1633513	1634307	264	1	E	1	E	1	E
M5005_Spy_1676c	uppS	1634320	1635069	249	0.9755	U	1	E	0.999	E
M5005_Spy_1677c	yajC	1635288	1635653	121	0	NE	0	NE	0	NE
M5005_Spy_1678c	-	1635769	1636116	115	0	NE	0	NE	0	NE
M5005_Spy_1680c	pulA	1638902	1639783	293	0	NE	0	NE	0	NE
M5005_Spy_1681c	dexB	1639954	1641567	537	0	NE	0	NE	0	NE
M5005_Spy_1682c	msmK	1641696	1642829	377	0	NE	0	NE	0	NE
M5005_Spy_1683c	lrp	1643127	1643975	282	0	NE	0	NE	0	NE
M5005_Spy_1684	ska	1644335	1645657	440	0	NE	0	NE	0	NE
M5005_Spy_1685c	dtd	1645755	1646198	147	0	NE	0	NE	0	NE
M5005_Spy_1686c	relA	1646213	1648432	739	0.00125	NE	1	E	1	E
M5005_Spy_1687c	sclA	1648684	1648827	47	0	NE	0	NE	0	NE
M5005_Spy_1690	nrdI2	1650113	1650595	160	0	NE	0	NE	0.001	NE
M5005_Spy_1691c	-	1650989	1651807	272	0	NE	0	NE	0	NE
M5005_Spy_1693c	-	1653782	1654075	97	0	NE	0	NE	0	NE
M5005_Spy_1694c	-	1654433	1655182	249	0	NE	0	NE	0	NE
M5005_Spy_1695c	prmA	1655182	1656135	317	0	NE	0	NE	0	NE
M5005_Spy_1696c	-	1656206	1656676	156	0	NE	0	NE	0	NE
M5005_Spy_1697	papB	1656876	1658633	585	0	NE	0	NE	0	NE
M5005_Spy_1698	trpG	1658666	1659232	188	0	NE	0	NE	0.621	U
M5005_Spy_1699	-	1659265	1660533	422	0	NE	0	NE	0	NE
M5005_Spy_1700	pai1	1661030	1661470	146	0	NE	0	NE	0	NE
M5005_Spy_1701	flaR	1661525	1662031	168	0	NE	0	NE	0	NE
M5005_Spy_1702	smeZ	1662280	1662981	233	0	NE	0	NE	0	NE
M5005_Spy_1703c	-	1663265	1663531	88	0	NE	0	NE	0	NE
M5005_Spy_1704	dppA	1663710	1665338	542	0	NE	0	NE	0	NE
M5005_Spy_1705	dppB	1665451	1666428	325	0	NE	0	NE	0	NE
M5005_Spy_1706	dppC	1666425	1667246	273	0.00025	NE	0	NE	0	NE
M5005_Spy_1707	dppD	1667258	1668061	267	0	NE	0	NE	0	NE
M5005_Spy_1708	dppE	1668045	1668671	208	0	NE	0	NE	0	NE
M5005_Spy_1709c	-	1668752	1668952	66	0	NE	0	NE	0	NE
M5005_Spy_1710c	-	1669121	1671598	825	0.0025	NE	0	NE	0	NE
M5005_Spy_1711c	lmb	1671611	1672531	306	0	NE	0	NE	0	NE
M5005_Spy_1712c	-	1672709	1673842	377	0	NE	0	NE	0	NE
M5005_Spy_1713c	-	1673896	1674048	50	-1	S	-1	S	-1	S
M5005_Spy_1714c	-	1674093	1675160	355	0	NE	0	NE	0	NE
M5005_Spy_1715c	scpA	1675257	1678751	1164	0	NE	0	NE	0	NE
M5005_Spy_1716	-	1679185	1680390	401	0	NE	0	NE	0	NE
M5005_Spy_1717	-	1680369	1680551	60	-1	S	-1	S	-1	S
M5005_Spy_1718c	sic1.01	1680969	1681910	313	0	NE	0	NE	0	NE
M5005_Spy_1719c	emm1.0	1682099	1683553	484	0	NE	0	NE	0	NE
M5005_Spy_1720c	mga	1683738	1685327	529	0	NE	0	NE	0	NE

M5005_Spy_1721	-	1685712	1685897	61	0.9495	U	0	NE	0.003	NE
M5005_Spy_1722c	-	1686003	1686254	83	0	NE	0	NE	0	NE
M5005_Spy_1723c	isp	1686333	1687934	533	0.00025	NE	0	NE	0	NE
M5005_Spy_1724c	ihk	1688036	1689424	462	0	NE	0	NE	0	NE
M5005_Spy_1725c	irr	1689421	1690074	217	0	NE	0	NE	0	NE
M5005_Spy_1726c	-	1690168	1691385	405	0	NE	0	NE	0	NE
M5005_Spy_1727c	-	1691398	1692072	224	0	NE	0	NE	0	NE
M5005_Spy_1728c	-	1692059	1693327	422	0	NE	0	NE	0	NE
M5005_Spy_1729c	-	1693750	1694154	134	0.00175	NE	0	NE	0	NE
M5005_Spy_1730c	-	1694181	1694477	98	0	NE	0	NE	0	NE
M5005_Spy_1731	grm	1694721	1694957	78	0	NE	0	NE	0	NE
M5005_Spy_1732c	prsA	1695233	1696162	309	0.00025	NE	0	NE	1	E
M5005_Spy_1733c	-	1696218	1696535	105	0	NE	0	NE	0	NE
M5005_Spy_1734c	-	1696642	1696947	101	0	NE	0.00025	NE	0.003	NE
M5005_Spy_1735c	speB	1696949	1698145	398	0	NE	0	NE	0	NE
M5005_Spy_1736	-	1698670	1698801	43	-1	S	-1	S	-1	S
M5005_Spy_1737	rgg	1699085	1699927	280	0	NE	0	NE	0	NE
M5005_Spy_1738c	spd	1700168	1700983	271	0	NE	0	NE	0	NE
M5005_Spy_1739	-	1701067	1701195	42	-1	S	-1	S	-1	S
M5005_Spy_1740	-	1701347	1701856	169	0	NE	0	NE	0	NE
M5005_Spy_1741c	gldA	1701938	1703026	362	0	NE	0	NE	0	NE
M5005_Spy_1742c	mipB	1703083	1703751	222	0	NE	0	NE	0	NE
M5005_Spy_1743c	pflD	1703764	1706136	790	0	NE	0	NE	0	NE
M5005_Spy_1744c	-	1706391	1707695	434	0	NE	0	NE	0	NE
M5005_Spy_1745c	-	1707705	1708013	102	0	NE	0	NE	0	NE
M5005_Spy_1746c	-	1708041	1708361	106	0	NE	0	NE	0	NE
M5005_Spy_1747c	-	1708649	1709629	326	0	NE	0	NE	0	NE
M5005_Spy_1748c	-	1709645	1710394	249	0	NE	0	NE	0	NE
M5005_Spy_1749	-	1710517	1711290	257	0	NE	0	NE	0	NE
M5005_Spy_1750	-	1711325	1711525	66	0	NE	0	NE	0	NE
M5005_Spy_1751c	secE	1711527	1711703	58	0.755	U	0.998	E	0.944	U
M5005_Spy_1752c	rpmG	1711717	1711869	50	-1	S	-1	S	-1	S
M5005_Spy_1753c	pbp2A	1711918	1714254	778	0	NE	0	NE	0	NE
M5005_Spy_1754c	-	1714293	1714673	126	0	NE	0	NE	0	NE
M5005_Spy_1755	-	1715164	1715355	63	0	NE	0	NE	0	NE
M5005_Spy_1756	-	1715249	1716250	333	0	NE	0	NE	0	NE
M5005_Spy_1757c	-	1716339	1717979	546	0	NE	0	NE	0	NE
M5005_Spy_1758	-	1718226	1719722	498	0	NE	0	NE	0	NE
M5005_Spy_1759c	-	1720207	1720371	54	0	NE	0	NE	0	NE
M5005_Spy_1760c	-	1720350	1720532	60	0	NE	0	NE	0	NE
M5005_Spy_1761c	groEL	1720925	1722556	543	0.773	U	1	E	1	E
M5005_Spy_1762c	groES	1722592	1722882	96	0.00025	NE	0.9995	E	0.998	E
M5005_Spy_1763c	clpC	1723060	1725504	814	0.00025	NE	0	NE	0.019	NE
M5005_Spy_1764c	ctsR	1725504	1725965	153	0	NE	0	NE	0	NE
M5005_Spy_1765c	csp	1726161	1726370	69	0	NE	0	NE	0.036	NE
M5005_Spy_1766c	-	1726507	1726611	34	-1	S	-1	S	-1	S
M5005_Spy_1767c	-	1726595	1727728	377	0	NE	0	NE	0	NE
M5005_Spy_1768	ahpC	1728685	1729245	186	0	NE	0	NE	0	NE
M5005_Spy_1769	ahpF	1729266	1730798	510	0.00025	NE	0	NE	0	NE
M5005_Spy_1770c	hutI	1730856	1732121	421	0	NE	0	NE	0	NE
M5005_Spy_1771	hutU	1732413	1734443	676	0.00025	NE	0	NE	0	NE
M5005_Spy_1772	-	1734532	1735431	299	0	NE	0	NE	0	NE
M5005_Spy_1773	-	1735442	1736068	208	0	NE	0	NE	0	NE
M5005_Spy_1774	fhs.2	1736086	1737759	557	0	NE	0	NE	0	NE
M5005_Spy_1775	-	1737781	1738377	198	0	NE	0	NE	0	NE
M5005_Spy_1776	-	1738597	1739940	447	0.00025	NE	0	NE	0	NE
M5005_Spy_1777	hutH	1739951	1741492	513	0	NE	0	NE	0	NE
M5005_Spy_1778	hutG	1741678	1742664	328	0	NE	0	NE	0	NE
M5005_Spy_1779c	-	1742695	1745769	1024	0	NE	0	NE	0	NE
M5005_Spy_1780	rpsB	1746073	1746840	255	0.64925	U	1	E	1	E
M5005_Spy_1781	tsf	1746974	1748014	346	0.99575	E	1	E	1	E
M5005_Spy_1782c	pepO	1748180	1750075	631	0	NE	0	NE	0	NE
M5005_Spy_1783c	dexS	1750283	1751911	542	0.00525	NE	0	NE	0.991	U

M5005_Spy_1784c	-	1751978	1754002	674	0	NE	0	NE	0.001	NE
M5005_Spy_1785	-	1754213	1754926	237	0	NE	0	NE	0.001	NE
M5005_Spy_1786c	-	1755205	1755372	55	0.00025	NE	0	NE	0	NE
M5005_Spy_1787	-	1755730	1756587	285	0.00025	NE	0	NE	0	NE
M5005_Spy_1788	yaaA	1756629	1757360	243	0	NE	0	NE	0	NE
M5005_Spy_1789c	nrdG	1757534	1758148	204	0	NE	0	NE	0.001	NE
M5005_Spy_1790c	-	1758148	1758657	169	0.00025	NE	0	NE	0	NE
M5005_Spy_1791c	-	1758666	1759601	311	0.0005	NE	0.5475	U	0.001	NE
M5005_Spy_1792c	-	1759630	1759776	48	-1	S	-1	S	-1	S
M5005_Spy_1793c	nrdD	1759958	1762156	732	0.00025	NE	0.00025	NE	1	E
M5005_Spy_1794c	-	1762253	1763812	519	0	NE	0	NE	0	NE
M5005_Spy_1795c	-	1764225	1764530	101	0	NE	0	NE	0	NE
M5005_Spy_1796c	-	1764542	1764961	139	0.00025	NE	1	E	1	E
M5005_Spy_1797c	-	1764958	1765227	89	0	NE	0	NE	0	NE
M5005_Spy_1798c	spxA	1765340	1765738	132	0.00025	NE	0	NE	1	E
M5005_Spy_1799c	recA	1766029	1767165	378	0.00025	NE	0	NE	0.001	NE
M5005_Spy_1800c	cinA	1767254	1768525	423	0	NE	0	NE	0	NE
M5005_Spy_1801c	tag	1768594	1769154	186	0.842	U	0	NE	0	NE
M5005_Spy_1802c	ruvA	1769164	1769760	198	0.00025	NE	0	NE	0.737	U
M5005_Spy_1803c	lmrP	1769762	1770982	406	0	NE	0	NE	0	NE
M5005_Spy_1804c	mutL	1770993	1772975	660	0.27475	U	0	NE	0.006	NE
M5005_Spy_1805c	mutS	1773104	1775659	851	0.26775	U	0	NE	0	NE
M5005_Spy_1806c	-	1775646	1775852	68	0	NE	0	NE	0	NE
M5005_Spy_1807c	argR2	1775995	1776432	145	0	NE	0	NE	0	NE
M5005_Spy_1808	argS	1776723	1778414	563	0.8705	U	1	E	1	E
M5005_Spy_1809	uviB	1778502	1778810	102	0	NE	0	NE	0	NE
M5005_Spy_1810c	-	1778837	1779709	290	0	NE	0	NE	0	NE
M5005_Spy_1811c	-	1779752	1780687	311	0	NE	0	NE	0	NE
M5005_Spy_1812c	-	1780650	1781591	313	0	NE	0	NE	0.043	NE
M5005_Spy_1813c	aspS	1781584	1783332	582	1	E	1	E	1	E
M5005_Spy_1814c	hisS	1783670	1784950	426	0.99725	E	1	E	1	E
M5005_Spy_1815	rpmF	1785170	1785352	60	0.00025	NE	0	NE	0	NE
M5005_Spy_1816	rpmG	1785368	1785517	49	-1	S	-1	S	-1	S
M5005_Spy_1817	cadD	1785810	1786424	204	0	NE	0	NE	0	NE
M5005_Spy_1818	cadC	1786406	1786774	122	0	NE	0	NE	0	NE
M5005_Spy_1819	-	1786825	1787748	307	0	NE	0	NE	0	NE
M5005_Spy_1820	-	1787814	1788566	250	0	NE	0	NE	0	NE
M5005_Spy_1821	-	1788563	1789165	200	0	NE	0	NE	0	NE
M5005_Spy_1822c	-	1789641	1789925	94	0.00025	NE	0	NE	0	NE
M5005_Spy_1823	-	1790376	1791218	280	0.00025	NE	0	NE	0	NE
M5005_Spy_1824c	-	1791265	1791906	213	0	NE	0	NE	0	NE
M5005_Spy_1825	-	1792113	1792439	108	0	NE	0	NE	0	NE
M5005_Spy_1826	-	1792426	1793013	195	0	NE	0	NE	0.001	NE
M5005_Spy_1827	-	1793010	1794089	359	0.00025	NE	0	NE	0.001	NE
M5005_Spy_1828c	-	1794207	1796075	622	0	NE	0	NE	0	NE
M5005_Spy_1830	-	1796614	1797147	177	0	NE	0	NE	0	NE
M5005_Spy_1831c	rpsD	1797301	1797912	203	1	E	1	E	1	E
M5005_Spy_1832c	-	1798115	1798219	34	-1	S	-1	S	-1	S
M5005_Spy_1833c	-	1798203	1799336	377	0	NE	0	NE	0	NE
M5005_Spy_1834c	-	1799968	1800240	90	0.00025	NE	0	NE	0.002	NE
M5005_Spy_1835c	holB/dnaC	1800257	1801624	455	1	E	1	E	1	E
M5005_Spy_1836c	rplI	1801654	1802106	150	0	NE	0	NE	0	NE
M5005_Spy_1837c	-	1802103	1804079	658	0.00025	NE	0	NE	0.001	NE
M5005_Spy_1838c	gidA	1804170	1806068	632	0.00075	NE	0	NE	1	E
M5005_Spy_1839c	-	1806192	1806509	105	0	NE	0	NE	0	NE
M5005_Spy_1840c	mnmA	1807286	1808407	373	1	E	1	E	1	E
M5005_Spy_1841	sdhB	1808705	1809376	223	0	NE	0	NE	0	NE
M5005_Spy_1842	sdhA	1809388	1810260	290	0.00025	NE	0	NE	0	NE
M5005_Spy_1843c	-	1810673	1811287	204	0	NE	0	NE	0	NE
M5005_Spy_1844c	cbiQ	1811659	1812459	266	0	NE	0.9905	U	1	E
M5005_Spy_1845c	cbiO	1812452	1813294	280	1	E	1	E	1	E
M5005_Spy_1846c	cbiO	1813270	1814160	296	0	NE	1	E	1	E
M5005_Spy_1847c	pgsA	1814111	1814653	180	0.008	NE	1	E	0.731	U

M5005_Spy_1848c	-	1814667	1815692	341	0	NE	0	NE	0.001	NE
M5005_Spy_1849c	-	1815742	1817031	429	0	NE	0	NE	0	NE
M5005_Spy_1850c	-	1817033	1818277	414	0	NE	0	NE	0	NE
M5005_Spy_1851	hasA	1818708	1819967	419	0.00025	NE	0	NE	0	NE
M5005_Spy_1852	hasB	1820003	1821211	402	0	NE	0	NE	0	NE
M5005_Spy_1853	hasC	1821393	1822307	304	0	NE	0	NE	0	NE
M5005_Spy_1854	-	1822615	1823028	137	0	NE	0	NE	0	NE
M5005_Spy_1855	recF	1823030	1824136	368	0	NE	0	NE	0	NE
M5005_Spy_1856c	-	1824191	1825024	277	0.00025	NE	0	NE	0	NE
M5005_Spy_1857c	guaB	1825256	1826737	493	0	NE	0	NE	0	NE
M5005_Spy_1858c	trsA	1827045	1828067	340	1	E	1	E	1	E
M5005_Spy_1859c	-	1828159	1828284	41	-1	S	-1	S	-1	S
M5005_Spy_1860	-	1828486	1829358	290	0	NE	0	NE	0	NE
M5005_Spy_1861	-	1829437	1831056	539	0.00025	NE	0	NE	0	NE
M5005_Spy_1862	-	1831139	1833715	858	0	NE	0	NE	0	NE
M5005_Spy_1863c	-	1834881	1835162	93	0	NE	0	NE	0	NE
M5005_Spy_1864c	-	1835744	1836223	159	0	NE	0	NE	0	NE
M5005_Spy_1865	htrA	1836435	1837658	407	0.00025	NE	0	NE	1	E
M5005_Spy_1866	parB	1837717	1838523	268	0.00025	NE	0	NE	0	NE

Table S4. Integrated gene essentiality determination for GAS M1T1 5448 for all time points.

(a) Spy numbers from MGAS5005 genome.

(b) When available, gene name is provided.

(c) Call integrating data from Bayesian analyses on 3 time points.

(d) Hyperlink to the NCBI Gene website (http://www.ncbi.nlm.nih.gov/gene).

(e) Information on transcription units based on the work of McClure *et al*. (2013).

(f) Asteriks refer to genes found in the GAS core genome.

Locus Tag(a)	Gene Name(b)	Call(c)	Start	Stop	Length	Strand	Gene ID(d)	COG Designation	Protein Function	Transcriptional Unit (e) Strand: + -	Part of GAS Core Genome (f)
M5005_Spy_0001	dnaA	E	202	1557	451	+	3571011	COG0593L	chromosome replication initiator DnaA		*
M5005_Spy_0002	dnaN	E	1712	2848	378	+	3571012	COG0592L	DNA polymerase III subunit beta		*
M5005_Spy_0003	-	NE	2923	3120	65	+	3571013	COG4481S	hypothetical protein M5005_Spy_0003		
M5005_Spy_0004	ychF	NE	3450	4565	371	+	3571014	COG0012J	GTP-dependent nucleic acid-binding protein EngD		*
M5005_Spy_0005	pth	NC	4635	5204	189	+	3571015	COG0193J	peptidyl-tRNA hydrolase		*
M5005_Spy_0006	trcF	NE	5207	8710	1167	+	3571016	COG1197LK	transcription-repair coupling factor		*
M5005_Spy_0007	-	NE	8872	9144	90	+	3572946	COG1188J	heat shock protein 15		*
M5005_Spy_0008	divIC	NE	9131	9502	123	+	3572947	COG2919D	cell division protein		*
M5005_Spy_0009	-	NC	9499	9624	41	+	3572948	-	hypothetical protein M5005_Spy_0009		
M5005_Spy_0010	-	NE	9637	10923	428	+	3572949	COG2367V	beta-lactamase		*
M5005_Spy_0011	tilS/mesJ	E	10920	12206	428	+	3572950	COG0037D	tRNA(Ile)-lysidine synthetase		*
M5005_Spy_0012	hpt	NC	12211	12753	180	+	3572951	COG0634F	hypoxanthine-guanine phosphoribosyltransferase		
M5005_Spy_0013	ftsH	E	12775	14754	659	+	3572952	COG0465O	cell division protein		*
M5005_Spy_0014	-	NE	15079	16470	463	+	3572953	COG0531E	amino acid permease		*
M5005_Spy_0015c	-	NC	16811	16945	44	-	3572954	-	hypothetical protein M5005_Spy_0015		
M5005_Spy_0017	sibA	E	31134	32330	398	+	3572901	COG3883S	secreted protein		*
M5005_Spy_0018	prsA.2	E	32583	33545	320	+	3572902	COG0462FE	ribose-phosphate pyrophosphokinase		*
M5005_Spy_0019	recO	NE	33731	34486	251	+	3572903	COG1381L	DNA repair protein RecO		*
M5005_Spy_0020	plsX	E	34589	35596	335	+	3572904	COG0416I	glycerol-3-phosphate acyltransferase		*
M5005_Spy_0021	acpP.2	NC	35589	35831	80	+	3572905	COG0236IQ	acyl carrier protein		
M5005_Spy_0022	-	NC	35952	36686	244	+	3572906	COG0152F	synthase		
M5005_Spy_0023	-	NE	36762	40535	1257	+	3572907	COG0046F	phosphoribosylformylglycinamidine synthase		*
M5005_Spy_0024	purF	NE	40696	42150	484	+	3572908	COG0034F	amidophosphoribosyltransferase		*
M5005_Spy_0025	purM	NE	42178	43200	340	+	3572909	COG0150F	phosphoribosylaminoimidazole synthetase		*
M5005_Spy_0026	purN	NE	43368	43922	184	+	3572910	COG0299F	phosphoribosylglycinamide formyltransferase		*
M5005_Spy_0027	purH	NE	44106	45653	515	+	3572911	COG0138F	phosphoribosylaminoimidazolecarboxamide		
M5005_Spy_0028c	-	NE	45711	46835	374	-	3572912	COG3942R	autolysin		*
M5005_Spy_0029	purD	NE	46998	48353	451	+	3572913	COG0151F	phosphoribosylamine--glycine ligase		*
M5005_Spy_0030	purE	NE	48511	49122	203	+	3572914	COG0041F	subunit		*
M5005_Spy_0031	purK	NE	49106	50182	358	+	3572915	COG0026F	subunit		*
M5005_Spy_0032	-	NE	50209	51852	547	+	3572916	-	hypothetical protein M5005_Spy_0032		
M5005_Spy_0033	purB	NE	51870	53162	430	+	3572917	COG0015F	adenylosuccinate lyase		*
M5005_Spy_0034	-	NE	53294	54205	303	+	3572880	-	transcriptional regulator		
M5005_Spy_0035	ruvB	NE	54431	55429	332	+	3572881	COG2255L	Holliday junction DNA helicase RuvB		*
M5005_Spy_0036	-	NE	55567	56004	145	+	3572882	COG0394T	protein tyrosine phosphatase		*
M5005_Spy_0037	-	NC	56027	56428	133	+	3572883	COG4642S	hypothetical protein M5005_Spy_0037		*
M5005_Spy_0038	-	E	56425	58200	591	+	3572884	COG1835I	acyltransferase		*
M5005_Spy_0039	adh2/adhE	NE	58509	61151	880	+	3572885	COG1454C	dehydrogenase		
M5005_Spy_0040	adhA/adhP	NE	61403	62419	338	+	3572886	COG1064R	alcohol dehydrogenase		*
M5005_Spy_0041	norM	NE	62807	63778	323	+	3572887	COG0534V	Na+ driven multidrug efflux pump		*
M5005_Spy_0043	rpsJ	E	64301	64609	102	+	3572889	COG0051J	30S ribosomal protein S10		*
M5005_Spy_0044	rplC	E	64825	65451	208	+	3572890	COG0087J	50S ribosomal protein L3		*
M5005_Spy_0045	rplD	E	65475	66098	207	+	3572891	COG0088J	50S ribosomal protein L4		*
M5005_Spy_0046	rplW	NE	66098	66394	98	+	3572892	COG0089J	50S ribosomal protein L23		*
M5005_Spy_0047	rplB	E	66412	67245	277	+	3572893	COG0090J	50S ribosomal protein L2		*
M5005_Spy_0048	rpsS	C	67384	67662	92	+	3572894	COG0185J	30S ribosomal protein S19		*
M5005_Spy_0049	rplV	E	67678	68022	114	+	3572895	COG0091J	50S ribosomal protein L22		*
M5005_Spy_0050	rpsC	E	68035	68688	217	+	3572896	COG0092J	30S ribosomal protein S3		*

M5005_Spy_0051	rplP	E	68692	69105	137	+	3572897	COG0197J	50S ribosomal protein L16	*
M5005_Spy_0052	rpmC	NE	69115	69321	68	+	3572898	COG0255J	50S ribosomal protein L29	
M5005_Spy_0053	rpsQ	NC	69347	69607	86	+	3572899	COG0186J	30S ribosomal protein S17	
M5005_Spy_0054	rplN	C	69632	70000	122	+	3572861	COG0093J	50S ribosomal protein L14	*
M5005_Spy_0055	rplX	NC	70079	70384	101	+	3572862	COG0198J	50S ribosomal protein L24	*
M5005_Spy_0056	rplE	NC	70408	70950	180	+	3572863	COG0094J	50S ribosomal protein L5	*
M5005_Spy_0057	rpsN	NC	70966	71151	61	+	3572864	COG0199J	30S ribosomal protein S14	
M5005_Spy_0058	rpsH	NC	71302	71700	132	+	3572865	COG0096J	30S ribosomal protein S8	*
M5005_Spy_0059	rplF	E	71903	72439	178	+	3572866	COG0097J	50S ribosomal protein L6	*
M5005_Spy_0060	rplR	NC	72544	72900	118	+	3572867	COG0256J	50S ribosomal protein L18	*
M5005_Spy_0061	rpsE	E	72919	73413	164	+	3572868	COG0098J	30S ribosomal protein S5	*
M5005_Spy_0062	rpmD	E	73428	73610	60	+	3572869	COG1841J	50S ribosomal protein L30	
M5005_Spy_0063	rplO	NE	73824	74264	146	+	3572870	COG0200J	50S ribosomal protein L15	*
M5005_Spy_0064	secY	E	74281	75585	434	+	3572871	COG0201U	preprotein translocase subunit SecY	*
M5005_Spy_0065	adk	NC	75735	76373	212	+	3572872	COG0563F	adenylate kinase	*
M5005_Spy_0066	infA	E	76491	76709	72	+	3572873	COG0361J	translation initiation factor IF-1	
M5005_Spy_0067	rpmJ	NC	76735	76851	38	+	3572874	COG0257J	50S ribosomal protein L36	
M5005_Spy_0068	rpsM	E	76869	77234	121	+	3572875	COG0099J	30S ribosomal protein S13	
M5005_Spy_0069	rpsK	E	77252	77635	127	+	3572876	COG0100J	30S ribosomal protein S11	*
M5005_Spy_0070	rpoA	E	77681	78619	312	+	3572877	COG0202K	DNA-directed RNA polymerase subunit alpha	*
M5005_Spy_0071	rplQ	E	78634	79020	128	+	3572878	COG0203J	50S ribosomal protein L17	*
M5005_Spy_0072c	-	NC	79618	79752	44	-	3572879	-	hypothetical protein M5005_Spy_0072	
M5005_Spy_0073	-	NC	86264	86449	61	+	3572845	-	hypothetical protein M5005_Spy_0073	
M5005_Spy_0074	-	NC	87087	87224	45	+	3572846	COG1947I	4-diphosphocytidyl-2-C-methyl-D-erythritol kinase	
M5005_Spy_0075	-	NC	87235	87393	52	+	3572847	COG1947I	partial	
M5005_Spy_0076	-	NC	87499	87660	53	+	3572848	COG1947I	4-diphosphocytidyl-2-C-methyl-D-erythritol kinase	
M5005_Spy_0077	adcR	NE	87770	88213	147	+	3572849	COG1846K	MarR family transcriptional regulator	*
M5005_Spy_0078	adcC	NE	88217	88936	239	+	3572850	COG1121P	high-affinity zinc uptake system ATP-binding protein	*
M5005_Spy_0079	adcB	NE	88929	89744	271	+	3572819	COG1108P	high-affinity zinc uptake system membrane protein	*
M5005_Spy_0080c	-	NE	89784	90167	127	-	3572820	COG0537FGR	bis(5\'-nucleosyl)-tetraphosphatase (asymmetrical)	
M5005_Spy_0081c	tyrS	E	90218	91474	418	-	3572821	COG0162J	tyrosyl-tRNA synthetase	*
M5005_Spy_0082	pbp1b	NE	91566	93878	770	+	3572822	COG0744M	1B	*
M5005_Spy_0083	rpoB	E	94142	97708	1188	+	3572823	COG0085K	DNA-directed RNA polymerase subunit beta	*
M5005_Spy_0084	rpoC	E	97799	101440	1213	+	3572824	COG0086K	DNA-directed RNA polymerase subunit beta\'	*
M5005_Spy_0085	-	NE	101592	101957	121	+	3572825	COG4699S	DNA binding protein	*
M5005_Spy_0086	comYA	NE	102050	102988	312	+	3572826	COG2804NU	competence protein ComG	*
M5005_Spy_0087	comYB	NE	102867	103958	363	+	3572827	COG1459NU	competence protein ComG	*
M5005_Spy_0088	comYC	NE	103960	104286	108	+	3572828	COG4537U	competence protein ComG	*
M5005_Spy_0089	-	NE	104360	104689	109	+	3572829	-	competence protein ComG	*
M5005_Spy_0090	-	NE	104646	104930	94	+	3572830	-	hypothetical protein M5005_Spy_0090	
M5005_Spy_0091	comYD	NE	104923	105357	144	+	3572831	COG4940U	competence protein ComG	*
M5005_Spy_0092	-	NE	105341	105667	108	+	3572832	-	competence protein ComG	*
M5005_Spy_0093	-	NE	105765	106718	317	+	3572833	COG0827L	adenine-specific methyltransferase	*
M5005_Spy_0094	ackA	NE	106777	107973	398	+	3572834	COG0282C	acetate kinase	*
M5005_Spy_0095	-	NE	108160	108468	102	+	3572835	-	hypothetical protein M5005_Spy_0095	
M5005_Spy_0096c	proC	NE	108551	109321	256	-	3572836	COG0345E	pyrroline-5-carboxylate reductase	*
M5005_Spy_0097c	pepA	NE	109369	110436	355	-	3572837	COG1363G	glutamyl aminopeptidase	*
M5005_Spy_0098	-	NC	110546	110710	54	-	3572838	-	hypothetical protein M5005_Spy_0098	
M5005_Spy_0099	-	NE	110892	111185	97	+	3572800	-	hypothetical protein M5005_Spy_0099	*
M5005_Spy_0100	trx.1	NE	111182	111499	105	+	3572801	COG0526OC	thioredoxin	
M5005_Spy_0101	-	NE	111517	112143	208	+	3572802	COG0073R	tRNA-binding domain-containing protein	
M5005_Spy_0102	ssb	NE	112295	112690	131	+	3572803	COG0629L	single-stranded DNA-binding protein	
M5005_Spy_0103c	-	NE	112950	113591	213	-	3572804	COG1428F	deoxyadenosine kinase/deoxyguanosine kinase	*
M5005_Spy_0104c	-	NE	113611	114588	325	-	3572805	COG0042J	tRNA-dihydrouridine synthase	*
M5005_Spy_0105c	hslO	NE	114575	115447	290	-	3572806	COG1281O	heat shock protein 33	*
M5005_Spy_0106c	rofA/nra	NE	115594	117087	497	-	3572807	-	transcriptional regulator	
M5005_Spy_0107	cbp	NE	117335	119623	762	+	3572808	-	fibronectin-binding protein	
M5005_Spy_0108	lepA-1	NE	119616	120173	185	+	3572809	-	signal peptidase I	
M5005_Spy_0109	prtF	NE	120206	121228	340	+	3572810	-	fibronectin-binding protein	

M5005_Spy_0110	eftLSL.B	**NE**	121238	121951	237	+	3572811	COG4509S	hypothetical protein M5005_Spy_0110	
M5005_Spy_0111	-	**NE**	121973	122620	215	+	3572812	-	hypothetical protein M5005_Spy_0111	
M5005_Spy_0112c	-	**NE**	122807	124129	440	-	3572813	COG3436L	transposase	
M5005_Spy_0113c	-	**NE**	124370	124720	116	-	3572814	COG3436L	transposase	
M5005_Spy_0114	-	**NE**	125024	125779	251	+	3572815	COG3764M	sortase	
M5005_Spy_0115c	-	**NE**	125973	126638	221	-	3572816	-	hypothetical protein M5005_Spy_0115	*
M5005_Spy_0116	atoE	**NE**	126987	128393	468	+	3572817	COG2031I	short-chain fatty acids transporter	*
M5005_Spy_0117c	-	**NE**	128461	128856	131	-	3572818	-	LysR family transcriptional regulator	
M5005_Spy_0118c	-	**NE**	128952	129371	139	-	3572780	COG0583K	LysR family transcriptional regulator	
M5005_Spy_0119	-	**NE**	129492	130676	394	+	3572781	COG0183I	acetyl-CoA acetyltransferase	
M5005_Spy_0120	atoD.2	**NE**	130688	131347	219	+	3572782	COG1788I	acetate CoA-transferase subunit alpha	
M5005_Spy_0121	-	**NE**	131350	131997	215	+	3572783	COG2057I	acetyl-CoA:acetoacetyl-CoA transferase subunit beta	*
M5005_Spy_0122c	-	**NE**	132119	132799	226	-	3572784	COG2964S	DNA-binding protein	*
M5005_Spy_0123	-	**NE**	132973	133338	121	+	3572785	COG0251J	translation initiation inhibitor	*
M5005_Spy_0124	sloR	**NE**	133375	134394	339	+	3572786	COG1299G	transcriptional regulator	*
M5005_Spy_0125	-	**NE**	134849	135169	106	+	3572787	-	hypothetical protein M5005_Spy_0125	*
M5005_Spy_0126	ntpI	**NE**	135159	137180	673	+	3572788	COG1269C	V-type ATP synthase subunit I	*
M5005_Spy_0127	ntpK	**NE**	137182	137661	159	+	3572789	COG0636C	V-type ATP synthase subunit K	*
M5005_Spy_0128	ntpE	**NE**	137729	138313	194	+	3572790	-	V-type sodium ATP synthase subunit E	*
M5005_Spy_0129	ntpC	**NE**	138329	139327	332	+	3572791	COG1527C	V-type ATP synthase subunit C	*
M5005_Spy_0130	ntpF	**NE**	139324	139644	106	+	3572792	COG1436C	V-type ATP synthase subunit F	
M5005_Spy_0131	ntpA	**NE**	139845	141620	591	+	3572793	COG1155C	V-type ATP synthase subunit A	*
M5005_Spy_0132	ntpB	**NE**	141621	143036	471	+	3572794	COG1156C	V-type ATP synthase subunit B	*
M5005_Spy_0133	ntpD	**NE**	143081	143707	208	+	3572795	COG1394C	V-type ATP synthase subunit D	*
M5005_Spy_0134c	-	**NE**	143827	145089	420	-	3572796	COG3853P	tellurite resistance protein	*
M5005_Spy_0135c	-	**NE**	145102	145980	292	-	3572797	-	hypothetical protein M5005_Spy_0135	*
M5005_Spy_0136	purA	**NE**	146418	147710	430	+	3572798	COG0104F	adenylosuccinate synthetase	*
M5005_Spy_0137	-	**NE**	148037	149080	347	+	3572799	COG1744R	nucleoside-binding protein	*
M5005_Spy_0138	nusG	**C**	149253	149792	179	+	3572761	COG0250K	transcription antitermination protein NusG	*
M5005_Spy_0139	nga	**NE**	150154	151518	454	+	3572762	-	NAD glycohydrolase	*
M5005_Spy_0140	-	**NE**	151523	152008	161	+	3572763	-	hypothetical protein M5005_Spy_0140	
M5005_Spy_0141	slo	**NE**	152032	153747	571	+	3572764	-	streptolysin O	*
M5005_Spy_0142	-	**NE**	154002	154433	143	+	3572765	-	hypothetical protein M5005_Spy_0142	
M5005_Spy_0143c	-	**NE**	154619	154855	78	-	3572766	-	hypothetical protein M5005_Spy_0143	
M5005_Spy_0144c	-	**NC**	155265	155432	55	-	3572767	-	hypothetical protein M5005_Spy_0144	
M5005_Spy_0145c	-	**NE**	155607	155894	95	-	3572768	-	hypothetical protein M5005_Spy_0145	
M5005_Spy_0146	metB	**NE**	156335	157525	396	+	3572769	COG0626E	cystathionine beta-lyase	
M5005_Spy_0147	leuS	**E**	157736	160237	833	+	3572770	COG0495J	leucyl-tRNA synthetase	*
M5005_Spy_0148	ulaA	**NC**	160544	161977	477	+	3572771	COG3037S	IIC	*
M5005_Spy_0149	-	**NE**	162048	162326	92	+	3572772	COG3414G	subunit IIB	*
M5005_Spy_0150	-	**NE**	162449	162934	161	+	3572773	COG1762GT	subunit IIA	*
M5005_Spy_0151	ulaD	**NE**	163025	163687	220	+	3572774	COG0269G	3-keto-L-gulonate-6-phosphate decarboxylase	*
M5005_Spy_0152	-	**NE**	163692	164555	287	+	3572775	COG3623G	L-xylulose 5-phosphate 3-epimerase	*
M5005_Spy_0153	araD	**NE**	164557	165261	234	+	3572776	COG0235G	L-ribulose-5-phosphate 4-epimerase	*
M5005_Spy_0154	-	**NC**	165319	165465	48	+	3572777	-	hypothetical protein M5005_Spy_0154	
M5005_Spy_0155	-	**NE**	165586	167232	548	+	3572778	COG3711K	BigG family transcription antiterminator	*
M5005_Spy_0156	-	**NE**	167485	168576	363	+	3572779	COG2220R	L-ascorbate 6-phosphate lactonase	*
M5005_Spy_0157	opuAA	**NE**	169064	170260	398	+	3572741	COG4175E	glycine betaine transport ATP-binding protein	*
M5005_Spy_0158	opuABC	**NE**	170276	172003	575	+	3572742	COG4176E	transporter permease	*
M5005_Spy_0159	polA	**E**	172134	174776	880	+	3572743	COG0749L	DNA polymerase I	*
M5005_Spy_0160	-	**NE**	174963	175418	151	+	3572744	COG1832R	CoA binding protein	*
M5005_Spy_0161	perR	**NE**	175470	175937	155	+	3572745	COG0735P	ferric uptake regulation protein	*
M5005_Spy_0162	vlg	**NE**	176094	176393	99	+	3572746	-	trans-acting positive regulator Mry	
M5005_Spy_0163	-	**NE**	176615	177949	444	+	3572747	COG3969R	sulfotransferase	
M5005_Spy_0164	-	**NE**	177942	178472	176	+	3572748	COG1475K	parB-like nuclease	
M5005_Spy_0165c	-	**NE**	178519	178761	80	-	3572749	COG3464L	transposase	
M5005_Spy_0166c	-	**NE**	178801	179016	71	-	3572750	COG2801L	transposase	
M5005_Spy_0167c	-	**NE**	179056	179625	189	-	3572751	COG2801L	transposase	
M5005_Spy_0168c	-	**NE**	179658	179873	71	-	3572752	COG2963L	transposase	

M5005_Spy_0169c	-	NE	180029	181339	436	-	3572753 COG0471P	malonate permease	*
M5005_Spy_0170	nadC	NE	181563	182435	290	+	3572754 COG0157H	nicotinate-nucleotide pyrophosphorylase	
M5005_Spy_0171c	-	NE	182735	183541	268	-	3572755 COG2801L	transposase	
M5005_Spy_0172c	-	NE	183562	184077	171	-	3572756 COG2963L	transposase	
M5005_Spy_0173c	-	NE	184163	185026	287	-	3572757 COG5523S	hypothetical protein M5005_Spy_0173	*
M5005_Spy_0174	-	NC	185063	185221	52	+	3572758 -	hypothetical protein M5005_Spy_0174	
M5005_Spy_0175	tgt	NE	185245	186387	380	+	3572759 COG0343J	queuine tRNA-ribosyltransferase	*
M5005_Spy_0176	-	NE	186604	186915	103	+	3572760 COG4357S	hypothetical protein M5005_Spy_0176	*
M5005_Spy_0177	bioY	NE	186919	187458	179	+	3572722 COG1268R	bioY protein	*
M5005_Spy_0178	-	NE	187598	188377	259	+	3572723 COG1234R	metal-dependent hydrolase	*
M5005_Spy_0179	-	NE	188377	188892	171	+	3572724 COG0590FJ	tRNA-specific adenosine deaminase	
M5005_Spy_0180c	-	NE	189506	190726	406	-	3572725 COG5279D	S-layer protein	*
M5005_Spy_0181	-	NC	190780	190887	35	+	3572726 -	hypothetical protein M5005_Spy_0181	
M5005_Spy_0182	speG	NE	191138	191842	234	+	3572727 -	exotoxin type G	
M5005_Spy_0183	-	NC	191927	192046	39	+	3572728 -	hypothetical protein M5005_Spy_0183	
M5005_Spy_0184	-	NC	192082	192201	39	+	3572729 -	hypothetical protein M5005_Spy_0184	
M5005_Spy_0185	pgi	E	192298	193647	449	+	3572730 COG0166G	glucose-6-phosphate isomerase	*
M5005_Spy_0186c	ralp4	NE	193996	195504	502	-	3572731 -	transcriptional regulator	*
M5005_Spy_0187	-	NE	196059	196763	234	+	3572732 COG5433L	transposase	
M5005_Spy_0188	-	NE	196666	197181	171	+	3572733 -	transposase	
M5005_Spy_0189	-	NC	197165	197290	41	+	3572734 -	hypothetical protein M5005_Spy_0189	
M5005_Spy_0190	-	NC	197371	197478	35	+	3572735 -	hypothetical protein M5005_Spy_0190	
M5005_Spy_0191	-	NE	197506	198177	223	+	3572736 COG0705R	rhomboid family integral membrane protein	*
M5005_Spy_0192c	hasC.2	NC	198276	199175	299	-	3572737 COG1210M	UTP-glucose-1-phosphate uridylyltransferase	*
M5005_Spy_0194c	gpsA	E	199408	200223	271	-	3572739 COG0240C	glycerol-3-phosphate dehydrogenase, partial	*
M5005_Spy_0195	-	NE	200521	200970	149	+	3572740 COG1846K	MarR family transcriptional regulator	*
M5005_Spy_0196	-	NE	200963	202669	568	+	3572702 COG1132V	protein/permease	*
M5005_Spy_0197	-	NE	202672	204456	594	+	3572703 COG1132V	protein/permease	*
M5005_Spy_0198	-	NE	204574	205341	255	+	3572704 COG1636S	hypothetical protein M5005_Spy_0198	*
M5005_Spy_0199	dut	NE	205451	205897	148	+	3572705 COG0756F	deoxyuridine 5\'-triphosphate nucleotidohydrolase	*
M5005_Spy_0200	radA	NE	205978	207339	453	+	3572706 COG1066O	DNA repair protein RadA	*
M5005_Spy_0201	-	C	207528	208025	165	+	3572707 COG0288P	carbonic anhydrase	*
M5005_Spy_0202	-	NE	208156	208866	236	+	3572708 COG2013S	hypothetical protein M5005_Spy_0202	*
M5005_Spy_0203	gltX	E	209048	210493	481	+	3572709 COG0008J	glutamyl-tRNA synthetase	*
M5005_Spy_0204	fasB	NE	210888	212234	448	+	3572710 COG2972T	sensory transduction protein kinase	*
M5005_Spy_0205	fasC	NE	212231	213514	427	+	3572711 COG2972T	sensory transduction protein kinase	
M5005_Spy_0206	fasA	NE	213518	214258	246	+	3572712 COG3279KT	response regulator	*
M5005_Spy_0207	rnpA	C	214798	215157	119	+	3572713 COG0594J	ribonuclease P	*
M5005_Spy_0208	-	NE	215141	215950	269	+	3572714 COG0706U	hypothetical protein M5005_Spy_0208	*
M5005_Spy_0209	-	NE	215962	216876	304	+	3572715 COG1847R	jag protein	*
M5005_Spy_0210	-	NC	216961	217083	40	+	3572716 -	hypothetical protein M5005_Spy_0210	
M5005_Spy_0211	rpmH	NC	217191	217325	44	+	3572717 -	50S ribosomal protein L34	
M5005_Spy_0212	-	NE	217599	218303	234	+	3572718 COG3010G	N-acetylmannosamine-6-phosphate 2-epimerase	*
M5005_Spy_0213	-	NE	218352	219671	439	+	3572719 COG1653G	N-acetylneuraminate-binding protein	*
M5005_Spy_0214	-	NE	219774	220661	295	+	3572720 COG1175G	N-acetylneuraminate transporter permease	*
M5005_Spy_0215	-	NE	220674	221504	276	+	3572721 COG0395G	N-acetylneuraminate transporter permease	*
M5005_Spy_0216	-	NE	221661	222323	220	+	3572683 COG5578S	hypothetical protein M5005_Spy_0216	*
M5005_Spy_0217	nanH	NE	222335	223249	304	+	3572684 COG0329EM	N-acetylneuraminate lyase	*
M5005_Spy_0218	-	NE	223271	224209	312	+	3572685 COG1940KG	N-acetylmannosamine kinase	*
M5005_Spy_0219c	-	NE	224320	225150	276	-	3572686 COG1737K	RpiR family transcriptional regulator	*
M5005_Spy_0220	tatD	NE	225402	226226	274	+	3572687 COG0084L	sec-independent protein translocase	*
M5005_Spy_0221	-	NE	226198	226788	196	+	3572688 COG1658L	ribonuclease M5	*
M5005_Spy_0222	ksgA	NE	226902	227774	290	+	3572689 COG0030J	dimethyladenosine transferase	*
M5005_Spy_0223	engC	E	228198	229070	290	+	3572690 COG1162R	ribosome-associated GTPase	*
M5005_Spy_0224	rpe	C	229080	229742	220	+	3572691 COG0036G	ribulose-phosphate 3-epimerase	*
M5005_Spy_0225	-	NE	229735	230367	210	+	3572692 COG1564H	thiamin pyrophosphokinase	*
M5005_Spy_0226	-	NE	230369	231640	423	+	3572693 COG1322S	rmuC family protein	*
M5005_Spy_0227	cbf	NE	231630	232568	312	+	3572694 COG3481R	CMP-binding factor	*
M5005_Spy_0228	purR	NE	232835	233674	279	+	3572695 COG0503F	pur operon repressor	*

M5005_Spy_0229	prgA	NE	233665	236286	873	+	3572696	COG1196D	surface exclusion protein	*
M5005_Spy_0230	rpsL	E	236494	236907	137	+	3572697	COG0048J	30S ribosomal protein S12	*
M5005_Spy_0231	rpsG	E	236928	237398	156	+	3572698	COG0049J	30S ribosomal protein S7	*
M5005_Spy_0232	fus	E	237765	239843	692	+	3572699	COG0480J	elongation factor G	*
M5005_Spy_0233	plr	E	240191	241201	336	+	3572700	COG0057G	glyceraldehyde-3-phosphate dehydrogenase	*
M5005_Spy_0234c	-	NC	241427	241543	38	-	3572701	-	hypothetical protein M5005_Spy_0234	
M5005_Spy_0235c	-	E	241685	242425	246	-	3572663	COG1126E	amino acid transport ATP-binding protein	*
M5005_Spy_0236c	-	E	242418	243986	522	-	3572664	COG0765E	amino acid ABC transporter permease	*
M5005_Spy_0237	-	NE	244184	246082	632	+	3572665	COG4907S	hypothetical protein M5005_Spy_0237	*
M5005_Spy_0238	uppP/bacA	NE	246148	246987	279	+	3572666	COG1968V	undecaprenyl pyrophosphate phosphatase	*
M5005_Spy_0239	mecA	NE	247133	247894	253	+	3572667	COG4862OTN	adaptor protein	*
M5005_Spy_0240	rgpG	E	248045	249070	341	+	3572668	COG0472M	acetylglucosaminephosphotransferase	*
M5005_Spy_0241	rgpG	NC	249040	249165	41	+	3572669	-	hypothetical protein M5005_Spy_0241	
M5005_Spy_0242	-	C	249192	249962	256	+	3572670	COG0396O	ABC transporter ATP-binding protein	*
M5005_Spy_0243	-	C	250057	251319	420	+	3572671	COG0719O	ABC transporter	*
M5005_Spy_0244	nifS3	C	251350	252576	408	+	3572672	COG0520E	cysteine desulfhydrase	*
M5005_Spy_0245	nifU	C	252563	253042	159	+	3572673	COG0822C	iscU protein	*
M5005_Spy_0246	-	C	253035	254453	472	+	3572674	COG0719O	ABC transporter	*
M5005_Spy_0247c	pbp7	NE	254605	255786	393	-	3572675	COG1686M	D-alanyl-D-alanine carboxypeptidase	
M5005_Spy_0248c	dacA2	NE	255954	257186	410	-	3572676	COG1686M	D-alanyl-D-alanine carboxypeptidase	*
M5005_Spy_0249	oppA	NE	257517	259487	656	+	3572677	COG4166E	oligopeptide-binding protein	
M5005_Spy_0250	oppB	NE	259540	261054	504	+	3572678	COG0601EP	oligopeptide transporter permease	*
M5005_Spy_0251	oppC	NE	261054	261980	308	+	3572679	COG1173EP	oligopeptide transporter permease	*
M5005_Spy_0252	oppD	NE	261989	263059	356	+	3572680	COG0444EP	oligopeptide transport ATP-binding protein	*
M5005_Spy_0253	oppF	NE	263052	263975	307	+	3572681	COG1123R	oligopeptide transport ATP-binding protein	*
M5005_Spy_0254c	-	NC	264216	264362	48	-	3572682	-	transposase	
M5005_Spy_0255c	-	NC	264737	264871	44	-	3572650	-	hypothetical protein M5005_Spy_0255	
M5005_Spy_0256	comX1.1	NE	270293	270844	183	+	3572657	-	competence-specific sigma factor	
M5005_Spy_0257	-	NE	271455	272159	234	+	3572658	COG5433L	transposase, partial	
M5005_Spy_0258	-	NE	272062	272577	171	+	3572659	-	transposase	
M5005_Spy_0259	-	NC	272561	272665	34	+	3572660	-	hypothetical protein M5005_Spy_0259	
M5005_Spy_0260	-	E	272683	273267	194	+	3572661	COG2179R	lipase	*
M5005_Spy_0261	-	C	273267	274385	372	+	3572662	COG1161R	GTP-binding protein YqeH	*
M5005_Spy_0262	-	NE	274410	274718	102	+	3572630	COG1534J	RNA-binding protein	
M5005_Spy_0263	nadD	E	274787	275419	210	+	3572631	COG1057H	nicotinic acid mononucleotide adenylyltransferase	*
M5005_Spy_0264	-	NE	275416	276009	197	+	3572632	COG1713H	HAD superfamily hydrolase	*
M5005_Spy_0265	-	NE	276009	276386	125	+	3572633	COG0799S	iojap superfamily protein	*
M5005_Spy_0266	-	NE	276428	277177	249	+	3572634	COG2226H	methyltransferase	*
M5005_Spy_0267	-	NE	277431	278537	368	+	3572635	COG1323R	hypothetical protein M5005_Spy_0267	*
M5005_Spy_0268	-	NC	278874	279029	51	+	3572636	-	hypothetical protein M5005_Spy_0268	
M5005_Spy_0269	-	NE	279019	279735	238	+	3572637	COG0217S	hypothetical protein M5005_Spy_0269	*
M5005_Spy_0270	atmA	NE	279938	280780	280	+	3572638	COG0834ET	ABC transporter substrate-binding protein	*
M5005_Spy_0271	atmB	NE	281109	281954	281	+	3572639	COG1464P	ABC transporter substrate-binding protein	*
M5005_Spy_0272	atmD	NE	282204	283268	354	+	3572640	COG1135P	ABC transporter ATP-binding protein	*
M5005_Spy_0273	atmE	NE	283269	283961	230	+	3572641	COG2011P	ABC transporter permease	*
M5005_Spy_0274c	braB	NE	284015	285385	456	-	3572642	COG1114E	protein	
M5005_Spy_0275	-	NE	285619	286833	404	+	3572643	COG3633E	serine/threonine transporter SstT	*
M5005_Spy_0276c	-	C	286888	287562	224	-	3572644	COG0569P	potassium uptake protein	*
M5005_Spy_0277c	-	NE	287572	288963	463	-	3572645	COG0168P	potassium uptake protein	*
M5005_Spy_0278c	gidB	NE	289033	289746	237	-	3572646	COG0357M	16S rRNA methyltransferase GidB	*
M5005_Spy_0279	lemA	NE	289896	290453	185	+	3572647	COG1704S	hypothetical protein M5005_Spy_0279	*
M5005_Spy_0280	htpX	NE	290500	291396	298	+	3572648	COG0501O	heat shock protein HtpX	*
M5005_Spy_0281	-	NE	291630	292163	177	+	3572649	COG1399R	hypothetical protein M5005_Spy_0281	*
M5005_Spy_0282	covR	NE	292430	293116	228	+	3572611	COG0745TK	response regulator	
M5005_Spy_0283	covS	NE	293259	294623	454	+	3572612	COG0642T	transmembrane histidine kinase	*
M5005_Spy_0284	nrdR	NE	294838	295332	164	+	3572613	COG1327K	NrdR family transcriptional regulator	*
M5005_Spy_0285	dnaB	E	295316	296491	391	+	3572614	COG3611L	replicative DNA helicase	*
M5005_Spy_0286	dnaI	E	296492	297394	300	+	3572615	COG1484L	primosomal protein DnaI	*
M5005_Spy_0287	engA	E	297457	298767	436	+	3572616	COG1160R	GTP-binding protein EngA	*

M5005_Spy_0288	snf	NE	298974	302072	1032	+	3572617	COG0553KL	SWF/SNF family helicase	*
M5005_Spy_0289	-	NE	302315	302917	200	+	3572618	-	hypothetical protein M5005_Spy_0289	*
M5005_Spy_0290	murC	E	302957	304285	442	+	3572619	COG0773M	UDP-N-acetylmuramate--L-alanine ligase	*
M5005_Spy_0291	-	NE	304331	304813	160	+	3572620	COG0456R	acetyltransferase	*
M5005_Spy_0292	-	C	304931	306499	522	+	3572621	COG1559R	aminodeoxychorismate lyase	*
M5005_Spy_0293	greA	NE	306524	307054	176	+	3572622	COG0782K	transcription elongation factor GreA	*
M5005_Spy_0294c	-	NC	307269	307412	47	-	3572623	-	transposase	
M5005_Spy_0295c	oxaA	E	307677	308600	307	-	3572624	COG0706U	OxaA-like protein precursor	*
M5005_Spy_0296c	-	NE	308682	308960	92	-	3572625	COG1254C	acylphosphatase	*
M5005_Spy_0297c	-	NE	309115	309630	171	-	3572626	-	transposase	
M5005_Spy_0298c	-	NE	309533	310237	234	-	3572627	COG5433L	transposase	
M5005_Spy_0299	-	NE	310535	311272	245	+	3572628	COG0566J	23S rRNA methyltransferase	*
M5005_Spy_0300	-	NE	311311	311811	166	+	3572629	COG1418R	HAD superfamily hydrolase	*
M5005_Spy_0301	-	NE	311826	312515	229	+	3572591	COG0670R	hypothetical protein M5005_Spy_0301	*
M5005_Spy_0302	-	C	312693	312935	80	+	3572592	COG3763S	hypothetical protein M5005_Spy_0302	
M5005_Spy_0303	glr	E	313113	313907	264	+	3572593	COG0796M	glutamate racemase	*
M5005_Spy_0304	-	NE	313904	314890	328	+	3572594	COG0127F	pyrophosphatase/unknown domain fusion protein	*
M5005_Spy_0305	-	NE	314869	315390	173	+	3572595	COG0622R	phosphoesterase	*
M5005_Spy_0306	-	NE	315387	315848	153	+	3572596	COG4109K	hypothetical protein M5005_Spy_0306	*
M5005_Spy_0307	xerD	NE	315845	316591	248	+	3572597	COG4974L	site-specific tyrosine recombinase XerD	*
M5005_Spy_0308	scpA	NE	316591	317292	233	+	3572598	COG1354S	segregation and condensation protein A	*
M5005_Spy_0309	scpB	NE	317289	317840	183	+	3572599	COG1386K	segregation and condensation protein B	*
M5005_Spy_0310	rluB	NE	317939	318685	248	+	3572600	COG1187J	ribosomal large subunit pseudouridine synthase B	*
M5005_Spy_0311	-	NE	318682	318945	87	+	3572601	COG0759S	hypothetical protein M5005_Spy_0311	
M5005_Spy_0312	-	NE	319123	319671	182	+	3572602	COG0219J	23S rRNA methyltransferase	*
M5005_Spy_0313	-	C	319982	320545	187	+	3572603	COG3601S	riboflavin transporter	*
M5005_Spy_0314	-	NE	320547	321200	217	+	3572604	COG0671I	phosphatidylglycerophosphatase B	*
M5005_Spy_0315	-	NE	321493	322413	306	+	3572605	COG1242R	Fe-S oxidoreductase	*
M5005_Spy_0316	-	NE	322452	323006	184	+	3572606	COG0144J	SAM-dependent methyltransferase	*
M5005_Spy_0317	hlyX	NE	323139	324473	444	+	3572607	COG1253R	hemolysin	*
M5005_Spy_0318	pflC	NE	324479	325342	287	+	3572608	COG1180O	pyruvate formate-lyase activating enzyme	*
M5005_Spy_0319	ppaC	NC	325473	326408	311	+	3572609	COG1227C	manganese-dependent inorganic pyrophosphatase	*
M5005_Spy_0320	-	NE	326484	327137	217	+	3572610	-	hypothetical protein M5005_Spy_0320	*
M5005_Spy_0321c	fhuG	NE	327182	328108	308	-	3572572	COG0609P	ferrichrome transporter permease	*
M5005_Spy_0322c	fhuB	NE	328180	329232	350	-	3572573	COG0609P	ferrichrome transporter permease	*
M5005_Spy_0323c	fhuD	NE	329222	330154	310	-	3572574	COG0614P	ferrichrome-binding protein	*
M5005_Spy_0324c	fhuA	NE	330180	330962	260	-	3572575	COG1120PH	ferrichrome ABC transporter ATP-binding protein	*
M5005_Spy_0325c	murE	E	331208	332653	481	-	3572576	COG0769M	ligase	*
M5005_Spy_0326	-	E	332741	334375	544	+	3572577	COG2244R	export protein for polysaccharides and teichoic acids	*
M5005_Spy_0327	upp	NE	334543	335172	209	+	3572578	COG0035F	uracil phosphoribosyltransferase	*
M5005_Spy_0328	clpP	C	335396	335986	196	+	3572579	COG0740OU	ATP-dependent Clp protease proteolytic subunit	*
M5005_Spy_0329	-	NE	336478	336753	91	+	3572580	COG4471S	hypothetical protein M5005_Spy_0329	
M5005_Spy_0330	tmk	E	337002	337637	211	+	3572581	COG0125F	thymidylate kinase	*
M5005_Spy_0331	dnaX	E	337655	338530	291	+	3572582	COG0470L	DNA polymerase III subunit delta\'	*
M5005_Spy_0332	-	NE	338549	338788	79	+	3572583	-	tpl protein	
M5005_Spy_0333	-	NC	338877	339038	53	+	3572584	COG1774S	signal peptidase-like protein	
M5005_Spy_0334	-	NC	339193	339516	107	+	3572585	COG4467S	DNA replication intiation control protein YabA	*
M5005_Spy_0335	-	NE	339521	340384	287	+	3572586	COG0313R	corrin/porphyrin methyltransferase	*
M5005_Spy_0336	-	NE	340411	340803	130	+	3572587	-	hypothetical protein M5005_Spy_0336	*
M5005_Spy_0337c	cutC	NE	340850	341479	209	-	3572588	COG3142P	copper homeostasis protein	*
M5005_Spy_0338	-	NE	341778	342134	118	+	3572589	COG1393P	arsenate reductase	*
M5005_Spy_0339c	exoA	NC	342208	343119	303	-	3572590	COG0708L	exodeoxyribonuclease III	*
M5005_Spy_0340	lctO	NE	343269	344450	393	+	3572552	COG1304C	L-lactate oxidase	*
M5005_Spy_0341	prtS	NE	344713	349656	1647	+	3572553	COG1404O	lactocepin	*
M5005_Spy_0343	-	NE	350145	350324	59	+	3572554	-	hypothetical protein M5005_Spy_0343	
M5005_Spy_0344	-	NE	350427	351134	235	+	3572555	COG3619S	permease	*
M5005_Spy_0345	metG	E	351377	353374	665	+	3572556	COG0143J	methionyl-tRNA synthetase	*
M5005_Spy_0346	-	NC	353705	353872	55	+	3572557	-	hypothetical protein M5005_Spy_0346	
M5005_Spy_0347	nrdF	NE	353869	354882	337	+	3572558	COG0208F	ribonucleotide-diphosphate reductase subunit beta	*

M5005_Spy_0348	nrdI	NE	354886	355374	162	+	3572559 COG1780F	ribonucleotide reductase stimulatory protein	*
M5005_Spy_0349	nrdE.1	NE	355341	357521	726	+	3572560 COG0209F	ribonucleotide-diphosphate reductase subunit alpha	*
M5005_Spy_0350	-	NC	357500	357718	72	+	3572561 -	hypothetical protein M5005_Spy_0350	
M5005_Spy_0351c	spyA	NE	357724	358476	250	-	3572562 COG5585T	C3 family ADP-ribosyltransferase	*
M5005_Spy_0352	-	NE	358935	359471	178	+	3572563 -	hypothetical protein M5005_Spy_0352	
M5005_Spy_0353	-	NE	359613	359996	127	+	3572564 -	hypothetical protein M5005_Spy_0353	
M5005_Spy_0354c	-	NE	360090	360722	210	-	3572565 -	hypothetical protein M5005_Spy_0354	
M5005_Spy_0355	-	NE	361401	361721	106	+	3572566 -	hypothetical protein M5005_Spy_0355	
M5005_Spy_0356c	speJ	NE	362034	362732	232	-	3572567 -	exotoxin type J	
M5005_Spy_0357c	-	NE	362980	363594	204	-	3572568 -	hypothetical protein M5005_Spy_0357	
M5005_Spy_0358	-	NE	363955	364191	78	+	3572569 -	hypothetical protein M5005_Spy_0358	
M5005_Spy_0359	fabG	NE	364175	364882	235	+	3572570 COG1028IQR	3-ketoacyl-ACP reductase	*
M5005_Spy_0360	-	NE	364928	365917	329	+	3572571 COG0673R	NAD-dependent oxidoreductase	*
M5005_Spy_0361	glpT	NE	366250	367587	445	+	3572533 COG2271G	phosphoglycerate transporter protein	*
M5005_Spy_0362	glmU	E	367760	369142	460	+	3572534 COG1207M	uridyltransferase/glucosamine-1-phosphate	*
M5005_Spy_0363	-	NE	369173	369727	184	+	3572535 COG0494LR	phosphohydrolase	*
M5005_Spy_0364	-	NC	369727	369978	83	+	3572536 -	hypothetical protein M5005_Spy_0364	
M5005_Spy_0365	pfs	E	369998	370693	231	+	3572537 COG0775F	nucleosidase	*
M5005_Spy_0366	-	NE	370844	371185	113	+	3572538 -	hypothetical protein M5005_Spy_0366	
M5005_Spy_0367c	mtsR	NC	371286	371933	215	-	3572539 COG1321K	iron-dependent repressor	*
M5005_Spy_0368	mtsA	NE	372091	373011	306	+	3572540 COG0803P	manganese-binding protein	*
M5005_Spy_0369	mtsB	NE	373075	373800	241	+	3572541 COG1121P	manganese transporter ATP-binding protein	*
M5005_Spy_0370	mtsC	NE	373801	374655	284	+	3572542 COG1108P	manganese transporter membrane protein	*
M5005_Spy_0371c	cypB	NE	374803	375609	268	-	3572543 COG0652O	peptidyl-prolyl cis-trans isomerase	*
M5005_Spy_0372	ftsK	E	375826	378231	801	+	3572544 COG1674D	cell division protein	*
M5005_Spy_0373c	-	NE	378301	378654	117	-	3572545 -	hypothetical protein M5005_Spy_0373	
M5005_Spy_0374	rplK	NC	378898	379323	141	+	3572546 COG0080J	50S ribosomal protein L11	*
M5005_Spy_0375	rplA	E	379429	380118	229	+	3572547 COG0081J	50S ribosomal protein L1	*
M5005_Spy_0376	-	NE	380469	381602	377	+	3572548 COG5433L	transposase	
M5005_Spy_0377	pyrH	E	381767	381937	56	+	3572549 COG0528F	uridylate kinase	*
M5005_Spy_0380	frr	E	382524	383081	185	+	3572513 COG0233J	ribosome recycling factor	*
M5005_Spy_0381	-	NE	383190	384047	285	+	3572514 COG2996S	S1 RNA-binding domain-containing protein	*
M5005_Spy_0382	msrA.2	NE	384120	384629	169	+	3572515 COG0225O	methionine sulfoxide reductase A	*
M5005_Spy_0383	-	NE	384626	384841	71	+	3572516 COG4479S	hypothetical protein M5005_Spy_0383	
M5005_Spy_0384	-	NE	384997	386166	389	+	3572517 -	surface antigen	*
M5005_Spy_0385	-	NE	386441	388252	603	+	3572518 COG4716S	hypothetical protein M5005_Spy_0385	
M5005_Spy_0386	phoH	NE	388411	389463	350	+	3572519 COG1702T	phoH protein	*
M5005_Spy_0387	-	NE	389509	390084	191	+	3572520 COG1573L	uracil DNA glycosylase	*
M5005_Spy_0388	-	E	390192	390740	182	+	3572521 COG0319R	metalloprotease	*
M5005_Spy_0389	dgk	E	390721	391128	135	+	3572522 COG0818M	diacylglycerol kinase	*
M5005_Spy_0390	era	C	391248	392144	298	+	3572523 COG1159R	GTP-binding protein Era	*
M5005_Spy_0391	-	NE	392128	392640	170	+	3572524 COG1051F	phosphohydrolase	*
M5005_Spy_0392c	-	NE	392945	393199	84	-	3572525 -	hypothetical protein M5005_Spy_0392	
M5005_Spy_0393	-	NE	393597	393842	81	+	3572526 -	hypothetical protein M5005_Spy_0393	
M5005_Spy_0394	-	NE	393857	394039	60	+	3572527 -	hypothetical protein M5005_Spy_0394	
M5005_Spy_0395c	-	NE	394230	394409	59	-	3572528 -	transposase	
M5005_Spy_0396c	-	NC	394348	394497	49	-	3572529 COG3328L	transposase	
M5005_Spy_0397c	-	NE	394494	394916	140	-	3572530 COG3328L	transposase	
M5005_Spy_0398	-	NE	395189	395398	69	+	3572531 -	bacteriocin	
M5005_Spy_0399	-	NE	395463	395663	66	+	3572532 -	hypothetical protein M5005_Spy_0399	
M5005_Spy_0400	silD	NE	395957	396151	64	+	3572494 -	hypothetical protein M5005_Spy_0400	
M5005_Spy_0401c	-	NC	396344	396460	38	-	3572495 -	hypothetical protein M5005_Spy_0401	
M5005_Spy_0402	-	NE	396644	397639	331	+	3572496 -	hypothetical protein M5005_Spy_0402	
M5005_Spy_0403	-	NE	397684	398130	148	+	3572497 -	hypothetical protein M5005_Spy_0403	
M5005_Spy_0404c	-	NE	398478	398747	89	-	3572498 -	hypothetical protein M5005_Spy_0404	
M5005_Spy_0405	-	NC	398948	399055	35	+	3572499 -	hypothetical protein M5005_Spy_0405	
M5005_Spy_0406c	-	NE	399269	399511	80	-	3572500 -	hypothetical protein M5005_Spy_0406	
M5005_Spy_0407	mutR	NE	399818	400684	288	+	3572501 -	transcriptional regulator	*
M5005_Spy_0408	fpg	NE	400856	401683	275	+	3572502 COG0266L	formamidopyrimidine-DNA glycosylase	*

M5005_Spy_0409	coaE	**E**	401593	402273	226	+	3572503 COG0237H	dephospho-CoA kinase	*
M5005_Spy_0410	-	**NE**	402463	403965	500	+	3572504 COG0433R	ATPase	*
M5005_Spy_0411	-	**NE**	404087	405280	397	+	3572505 COG2814G	multidrug resistance protein B	
M5005_Spy_0412	rpmG	**NC**	405277	405423	48	+	3572506 COG0267J	50S ribosomal protein L33	
M5005_Spy_0413	secG	**E**	405469	405705	78	+	3572507 COG1314U	preprotein translocase subunit SecG	
M5005_Spy_0414	-	**NE**	405802	408132	776	+	3572508 COG0557K	exoribonuclease II	*
M5005_Spy_0415	smpB	**NC**	408135	408602	155	+	3572509 COG0691O	SsrA-binding protein	*
M5005_Spy_0416	-	**NE**	408617	409327	236	+	3572510 COG3823O	glutaminyl-peptide cyclotransferase	*
M5005_Spy_0417c	pcp	**NE**	409443	410090	215	-	3572511 COG2039O	pyrrolidone-carboxylate peptidase	*
M5005_Spy_0418c	-	**NE**	410140	411066	308	-	3572512 COG3817S	permease	*
M5005_Spy_0419c	-	**NE**	411066	411749	227	-	3572474 COG3819S	permease	*
M5005_Spy_0420c	-	**NE**	411960	412886	308	-	3572475 COG0463M	glucosyltransferase	*
M5005_Spy_0421c	gloA	**NE**	413031	413408	125	-	3572476 COG0346E	lactoylglutathione lyase	*
M5005_Spy_0422c	-	**NE**	413419	414084	221	-	3572477 COG0778C	NAD(P)H-dependent quinone reductase	*
M5005_Spy_0423c	pepQ	**NE**	414133	415218	361	-	3572478 COG0006E	Xaa-Pro dipeptidase	*
M5005_Spy_0424	ccpA	**NC**	415392	416393	333	+	3572479 COG1609K	catabolite control protein A	*
M5005_Spy_0425	-	**C**	416524	417522	332	+	3572480 COG0438M	glycosyltransferase	*
M5005_Spy_0426	-	**E**	417524	418858	444	+	3572481 COG0438M	1,2-diacylglycerol 3-glucosyltransferase	*
M5005_Spy_0427	thrS	**E**	419280	421223	647	+	3572482 COG0441J	threonyl-tRNA synthetase	*
M5005_Spy_0428	drrA/tagH	**NE**	421364	422356	330	+	3572483 COG4586R	daunorubicin resistance ATP-binding protein	*
M5005_Spy_0429	-	**NE**	422358	423176	272	+	3572484 COG4587R	daunorubicin resistance transmembrane protein	*
M5005_Spy_0430	-	**NE**	423178	423963	261	+	3572485 COG3694R	ABC transporter permease	*
M5005_Spy_0431	-	**NC**	424164	424313	49	+	3572486 -	dihydroxyacetone kinase	
M5005_Spy_0432	-	**NE**	424708	425856	382	+	3572487 COG0183I	acetyl-CoA acetyltransferase	*
M5005_Spy_0433	-	**NE**	425813	427060	415	+	3572488 COG0318IQ	long-chain-fatty-acid--CoA ligase	*
M5005_Spy_0434	-	**NE**	427116	428150	344	+	3572489 -	hypothetical protein M5005_Spy_0434	*
M5005_Spy_0435	vicR	**E**	428312	429022	236	+	3572490 COG0745TK	two-component response regulator	*
M5005_Spy_0436	vicK	**NE**	429015	430367	450	+	3572491 COG5002T	two-component sensor histidine kinase	*
M5005_Spy_0437	vicX	**NE**	430371	431180	269	+	3572492 COG1235R	Zn-dependent hydrolase	*
M5005_Spy_0438	rnc	**C**	431624	432316	230	+	3572493 COG0571K	ribonuclease III	*
M5005_Spy_0439	smc	**NE**	432317	435856	1179	+	3572455 COG1196D	chromosome partition protein	*
M5005_Spy_0440c	-	**NE**	436109	436960	283	-	3572456 -	transcriptional regulator	*
M5005_Spy_0441	aroE	**NE**	437234	438106	290	+	3572457 COG0169E	shikimate 5-dehydrogenase	*
M5005_Spy_0442	-	**NE**	438205	438939	244	+	3572458 COG1082G	hypothetical protein M5005_Spy_0442	*
M5005_Spy_0443	-	**NE**	438941	439675	244	+	3572459 COG1478S	hypothetical protein M5005_Spy_0443	*
M5005_Spy_0444	-	**NE**	439668	440654	328	+	3572460 COG0673R	hypothetical protein M5005_Spy_0444	*
M5005_Spy_0445	metK1	**NE**	440664	441863	399	+	3572461 COG1812E	S-adenosylmethionine synthetase	*
M5005_Spy_0446	-	**NE**	441847	442815	322	+	3572462 COG1493T	hypothetical protein M5005_Spy_0446	*
M5005_Spy_0447	-	**NE**	442870	443859	329	+	3572463 COG0463M	cell wall biosynthesis glycosyltransferase	*
M5005_Spy_0448	-	**NC**	444385	444528	47	+	3572464 -	hypothetical protein M5005_Spy_0448	
M5005_Spy_0449	hasB.2	**NE**	444552	445709	385	+	3572465 COG1004M	UDP-glucose 6-dehydrogenase	
M5005_Spy_0450	mefE	**NE**	445794	447002	402	+	3572466 COG2270R	macrolide-efflux protein	
M5005_Spy_0451c	-	**NE**	447105	447314	69	-	3572467 COG3655K	transcriptional regulator	
M5005_Spy_0452c	-	**NE**	447304	448149	281	-	3572468 -	chromosome segregation ATPase	*
M5005_Spy_0453c	-	**NE**	448234	448962	242	-	3572469 -	chromosome segregation ATPase	*
M5005_Spy_0454c	-	**NE**	448953	449177	74	-	3572470 -	hypothetical protein M5005_Spy_0454	
M5005_Spy_0455c	-	**NE**	449177	450271	364	-	3572471 -	hypothetical protein M5005_Spy_0455	*
M5005_Spy_0456	-	**NC**	450317	450619	100	+	3572472 COG3077L	plasmid stabilization system antitoxin protein	*
M5005_Spy_0457	-	**NE**	450619	450924	101	+	3572473 COG3668R	plasmid stabilization system protein	*
M5005_Spy_0458	-	**NE**	450943	451215	90	+	3572435 -	hypothetical protein M5005_Spy_0458	
M5005_Spy_0459	-	**NE**	451320	451772	150	+	3572436 COG4695S	portal protein	
M5005_Spy_0460	-	**NE**	451838	452152	104	+	3572437 -	hypothetical protein M5005_Spy_0460	
M5005_Spy_0461	-	**NE**	452569	452907	112	+	3572438 COG3177S	hypothetical protein M5005_Spy_0461	
M5005_Spy_0462	-	**NE**	452931	453755	274	+	3572439 COG0017J	asparagine synthetase A	
M5005_Spy_0463	-	**NE**	453769	455016	415	+	3572440 COG0439I	hypothetical protein M5005_Spy_0463	
M5005_Spy_0464	mccF	**NE**	455244	455960	238	+	3572441 COG1619V	microcin C7 self-immunity protein	
M5005_Spy_0465	-	**NE**	455973	456173	66	+	3572442 -	hypothetical protein M5005_Spy_0465	
M5005_Spy_0466	-	**NE**	456293	457192	299	+	3572443 COG0124J	hypothetical protein M5005_Spy_0466	
M5005_Spy_0467c	-	**NE**	457645	458358	237	-	3572444 COG2801L	transposase	

M5005_Spy_0468c	-	NE	458406	458681	91	-	3572445 COG2963L	transposase	
M5005_Spy_0469	-	NE	458701	458919	72	+	3572446 -	hypothetical protein M5005_Spy_0469	
M5005_Spy_0470	-	NE	458986	459783	265	+	3572447 COG0561R	HAD superfamily hydrolase	*
M5005_Spy_0471	-	NE	459787	460611	274	+	3572448 COG0561R	HAD superfamily hydrolase	*
M5005_Spy_0472	ftsY	E	460611	462161	516	+	3572449 COG0552U	cell division protein	*
M5005_Spy_0473c	-	NE	462215	463582	455	-	3572450 COG2814G	multidrug resistance protein B	*
M5005_Spy_0474	licT/bglG	NE	463910	464752	280	+	3572451 COG3711K	BigG family transcription antiterminator	*
M5005_Spy_0475	-	NE	464916	466616	566	+	3572452 COG1263G	subunit IIABC	*
M5005_Spy_0476	bglA	NE	466635	468059	474	+	3572453 COG2723G	6-phospho-beta-glucosidase	*
M5005_Spy_0477c	-	NE	468158	468973	271	-	3572454 COG3689S	hypothetical protein M5005_Spy_0477	*
M5005_Spy_0478c	-	NE	468973	469875	300	-	3572416 COG0701R	hypothetical protein M5005_Spy_0478	*
M5005_Spy_0479	-	NE	470009	470206	65	+	3572417 -	hypothetical protein M5005_Spy_0479	
M5005_Spy_0480	-	NE	470304	470432	42	-	3572419 COG2183K	transcription accessory protein	*
M5005_Spy_0481c	-	NC	470412	472544	710	+	3572418 -	hypothetical protein M5005_Spy_0481	
M5005_Spy_0482	-	NE	472486	472968	160	+	3572420 COG3091S	hypothetical protein M5005_Spy_0482	
M5005_Spy_0483	-	NE	473032	473304	90	+	3572421 COG1983KT	stress-responsive transcriptional regulator	
M5005_Spy_0484	ptsK	E	473609	474601	330	+	3572422 COG1493T	HPr kinase/phosphorylase	*
M5005_Spy_0485	lgt	NE	474598	475377	259	+	3572423 COG0682M	prolipoprotein diacylglyceryl transferase	*
M5005_Spy_0486	-	NE	475399	475806	135	+	3572424 COG4768R	hypothetical protein M5005_Spy_0486	*
M5005_Spy_0487	-	NE	475799	476227	142	+	3572425 -	hypothetical protein M5005_Spy_0487	*
M5005_Spy_0488c	-	NE	476386	476670	94	-	3572426 -	hypothetical protein M5005_Spy_0488	
M5005_Spy_0489	-	NE	476869	477237	122	+	3572427 COG0826O	U32 family peptidase	*
M5005_Spy_0491	-	NE	477887	479173	428	+	3572429 COG0826O	U32 family peptidase	*
M5005_Spy_0492c	-	NC	479186	479314	42	-	3572430 -	hypothetical protein M5005_Spy_0492	
M5005_Spy_0493	-	NE	479383	479595	70	+	3572431 COG4443S	hypothetical protein M5005_Spy_0493	*
M5005_Spy_0494c	-	NC	479692	479832	46	-	3572432 -	hypothetical protein M5005_Spy_0494	
M5005_Spy_0495c	lysS	E	479968	481461	497	-	3572433 COG1190J	lysyl-tRNA synthetase	*
M5005_Spy_0496	-	NE	481635	482537	300	+	3572434 COG1011R	HAD superfamily hydrolase	*
M5005_Spy_0497c	-	NE	482645	483268	207	-	3572396 COG0406G	phosphoglycerate mutase	
M5005_Spy_0498c	-	NE	483609	484088	159	-	3572397 COG2606S	transcriptional regulator	*
M5005_Spy_0499c	-	NE	484179	484742	187	-	3572398 COG3859S	thiamine transporter	*
M5005_Spy_0500c	-	C	485011	485859	282	-	3572399 COG3757M	N-acetylmuramoyl-L-alanine amidase	*
M5005_Spy_0501	-	NE	486184	486687	167	+	3572400 -	hypothetical protein M5005_Spy_0501	*
M5005_Spy_0502	-	NE	486671	487057	128	+	3572401 -	hypothetical protein M5005_Spy_0502	*
M5005_Spy_0503c	-	NE	487103	487582	159	-	3572402 COG0386O	glutathione peroxidase	*
M5005_Spy_0504c	pepF	NE	487575	489374	599	-	3572403 COG1164E	oligoendopeptidase F	*
M5005_Spy_0505	ppc	NE	489518	492331	937	+	3572404 COG2352C	phosphoenolpyruvate carboxylase	*
M5005_Spy_0506	ftsW	E	492501	493775	424	+	3572405 COG0772D	cell division protein	*
M5005_Spy_0507c	-	NC	493792	493932	46	-	3572406 -	hypothetical protein M5005_Spy_0507	
M5005_Spy_0508	tuf	E	494129	495325	398	+	3572407 COG0050J	elongation factor Tu	*
M5005_Spy_0509	tpiA	C	495566	496324	252	+	3572408 COG0149G	triosephosphate isomerase	*
M5005_Spy_0510c	murN	E	496423	497658	411	-	3572409 COG2348V	resistance	*
M5005_Spy_0511c	murM	E	497645	498871	408	-	3572410 COG2348V	alanyltransferase/UDP-N-	*
M5005_Spy_0512c	-	NE	498871	499680	269	-	3572411 COG0561R	HAD superfamily hydrolase	*
M5005_Spy_0513c	-	NC	499831	500064	77	-	3572412 -	hypothetical protein M5005_Spy_0513	
M5005_Spy_0514c	-	NE	500136	501437	433	-	3572413 COG1078R	dGTP triphosphohydrolase	*
M5005_Spy_0515	-	NE	501519	501905	128	+	3572414 COG4506S	hypothetical protein M5005_Spy_0515	*
M5005_Spy_0516	pacL	E	502136	504817	893	+	3572415 COG0474P	calcium-transporting ATPase	*
M5005_Spy_0517c	regR	NE	504901	505896	331	-	3572377 COG1609K	LacI family transcriptional regulator	*
M5005_Spy_0518c	-	NE	505960	507867	635	-	3572378 -	oligohyaluronate lyase	*
M5005_Spy_0519c	agaD	NE	507954	508775	273	-	3572379 COG3716G	transporter subunit IID	*
M5005_Spy_0520c	agaW	NE	508762	509544	260	-	3572380 COG3715G	transporter subunit IIC	*
M5005_Spy_0521c	agaV	NE	509563	510051	162	-	3572381 COG3444G	transporter subunit IIB	*
M5005_Spy_0522c	ugl	NE	510087	511286	399	-	3572382 COG4225R	unsaturated glucuronyl hydrolase	
M5005_Spy_0523	-	NE	511353	511883	176	+	3572383 -	hypothetical protein M5005_Spy_0523	*
M5005_Spy_0524	idnO	NE	512059	512853	264	+	3572384 COG1028IQR	gluconate 5-dehydrogenase	*
M5005_Spy_0525	-	NE	512878	513519	213	+	3572385 COG0698G	hypothetical protein M5005_Spy_0525	*
M5005_Spy_0526	kdgK	NE	513548	514549	333	+	3572386 COG0524G	2-dehydro-3-deoxygluconokinase	*
M5005_Spy_0527	kgdA	NE	514554	515189	211	+	3572387 COG0800G	phosphogluconate aldolase	*

M5005_Spy_0528	-	**NE**	515485	516135	216	+	3572388	COG0637R	phosphodismutase	*
M5005_Spy_0529	-	**NE**	516776	517951	391	+	3572389	COG1600C	(Fe-S)-binding protein	
M5005_Spy_0530	prfB	**E**	518105	519118	337	+	3572390	COG1186J	peptide chain release factor 2	*
M5005_Spy_0531	ftsE	**NC**	519137	519829	230	+	3572391	COG2884D	cell division ATP-binding protein	*
M5005_Spy_0532	ftsX	**C**	519822	520751	309	+	3572392	COG2177D	cell division protein	*
M5005_Spy_0533c	-	**NE**	521061	521696	211	-	3572393	COG0491R	hydroxyacylglutathione hydrolase	*
M5005_Spy_0534	-	**NE**	521927	522541	204	+	3572394	COG1028IQR	acetoin reductase	
M5005_Spy_0535	-	**NC**	522545	522691	48	+	3572395	COG1028IQR	acetoin dehydrogenase, partial	
M5005_Spy_0536	dinG	**NE**	522841	525300	819	+	3572358	COG1199KL	polymerase III subunit epsilon	*
M5005_Spy_0537	aspC	**NE**	525635	526828	397	+	3572359	COG0436E	aspartate aminotransferase	*
M5005_Spy_0538	asnC	**E**	526849	528195	448	+	3572360	COG0017J	asparaginyl-tRNA synthetase	*
M5005_Spy_0539	-	**NE**	528609	529499	296	+	3572361	COG1660R	hypothetical protein M5005_Spy_0539	*
M5005_Spy_0540	-	**NE**	529496	530473	325	+	3572362	COG0391S	transporter	*
M5005_Spy_0541	-	**NE**	530470	531381	303	+	3572363	COG1481S	hypothetical protein M5005_Spy_0541	*
M5005_Spy_0542	pepD	**NE**	531514	532911	465	+	3572364	COG4690E	dipeptidase	
M5005_Spy_0543	adcA	**NE**	533063	534610	515	+	3572365	COG3443R	high-affinity zinc uptake system protein znuA	*
M5005_Spy_0544	-	**NE**	534758	535480	240	+	3572366	COG2188K	GntR family transcriptional regulator	*
M5005_Spy_0545	agaS	**NE**	535499	536698	399	+	3572367	COG2222M	galactosamine-6-phosphate deaminase	*
M5005_Spy_0546c	rpmE2	**C**	536795	537055	86	-	3572368	COG0254J	50S ribosomal protein L31	
M5005_Spy_0547c	-	**NC**	537170	538111	313	-	3572369	COG0618R	phosphoesterase, DHH family protein	*
M5005_Spy_0548	flaV	**C**	538505	538954	149	+	3572370	COG0716C	flavodoxin	*
M5005_Spy_0549	-	**NE**	539130	539414	94	+	3572371	COG1605E	hypothetical protein M5005_Spy_0549	
M5005_Spy_0550	-	**NE**	539407	540669	420	+	3572372	COG0038P	chloride channel protein	*
M5005_Spy_0551	rplS	**NC**	540784	541131	115	+	3572373	COG0335J	50S ribosomal protein L19	*
M5005_Spy_0552	-	**NE**	542134	542703	189	+	3572375	COG0546R	DNA gyrase	*
M5005_Spy_0553	gyrB	**E**	542704	544656	650	+	3572376	COG0187L	DNA gyrase subunit B	*
M5005_Spy_0554	ezrA	**E**	545024	546748	574	+	3572339	COG4477D	septation ring formation regulator EzrA	*
M5005_Spy_0555c	-	**NE**	546880	547338	152	-	3572340	COG5506S	hypothetical protein M5005_Spy_0555	*
M5005_Spy_0556	eno	**E**	547565	548872	435	+	3572341	COG0148G	phosphopyruvate hydratase	*
M5005_Spy_0557c	-	**NE**	549398	549988	196	-	3572342	COG3547L	transposase	
M5005_Spy_0558c	-	**NE**	550037	550288	83	-	3572343	-	transposase	
M5005_Spy_0559c	-	**NE**	550650	551306	218	-	3572344	-	transcriptional regulator	
M5005_Spy_0560c	-	**NE**	551303	552190	295	-	3572345	-	transcriptional regulator	
M5005_Spy_0561	epf	**NE**	552662	558841	2059	+	3572346	COG5271R	extracellular matrix binding protein	
M5005_Spy_0562	sagA	**NE**	559706	559867	53	+	3572347	-	streptolysin S	
M5005_Spy_0563	sagB	**NE**	560089	561039	316	+	3572348	COG0778C	streptolysin S biosynthesis protein	*
M5005_Spy_0564	sagC	**NE**	561036	562094	352	+	3572349	-	streptolysin S biosynthesis protein	*
M5005_Spy_0565	sagD	**NE**	562114	563472	452	+	3572350	COG1944S	streptolysin S biosynthesis protein	*
M5005_Spy_0566	sagE	**NE**	563447	564118	223	+	3572351	COG1266R	streptolysin S self-immunity protein	*
M5005_Spy_0567	sagF	**NE**	564115	564798	227	+	3572352	-	streptolysin S biosynthesis protein	*
M5005_Spy_0568	sagG	**NE**	564821	565744	307	+	3572353	COG1131V	streptolysin S export ATP-binding protein	*
M5005_Spy_0569	sagH	**NE**	565753	566880	375	+	3572354	COG1668CP	streptolysin S export transmembrane protein	*
M5005_Spy_0570	sagI	**NE**	566877	567995	372	+	3572355	COG0842V	streptolysin S export transmembrane protein	*
M5005_Spy_0571	-	**NE**	568566	571298	910	+	3572356	COG2374R	hypothetical protein M5005_Spy_0571	*
M5005_Spy_0572	-	**NE**	571576	572076	166	+	3572357	COG4708S	hypothetical protein M5005_Spy_0572	*
M5005_Spy_0573	ligA	**E**	572270	574228	652	+	3572319	COG0272L	NAD-dependent DNA ligase LigA	*
M5005_Spy_0574	-	**E**	574242	575264	340	+	3572320	COG1597IR	lipid kinase	*
M5005_Spy_0575	atpE	**NC**	575657	575854	65	+	3572321	COG0636C	ATP synthase F0F1 subunit C	
M5005_Spy_0576	atpB	**NC**	575889	576605	238	+	3572322	COG0356C	ATP synthase F0F1 subunit A	*
M5005_Spy_0577	atpF	**E**	576623	577117	164	+	3572323	COG0711C	ATP synthase F0F1 subunit B	*
M5005_Spy_0578	atpH	**E**	577117	577653	178	+	3572324	COG0712C	ATP synthase F0F1 subunit delta	*
M5005_Spy_0579	atpA	**E**	577669	579177	502	+	3572325	COG0056C	ATP synthase F0F1 subunit alpha	*
M5005_Spy_0580	atpG	**E**	579193	580068	291	+	3572326	COG0224C	ATP synthase F0F1 subunit gamma	*
M5005_Spy_0581	atpD	**E**	580230	581636	468	+	3572327	COG0055C	ATP synthase F0F1 subunit beta	*
M5005_Spy_0582	atpC	**NC**	581649	582065	138	+	3572328	COG0355C	ATP synthase F0F1 subunit epsilon	*
M5005_Spy_0583	-	**NE**	582331	582588	85	+	3572329	-	hypothetical protein M5005_Spy_0583	
M5005_Spy_0584	murA	**NE**	582815	583924	369	+	3572330	COG0766M	UDP-N-acetylglucosamine 1-carboxyvinyltransferase	*
M5005_Spy_0585	epuA	**NE**	583928	584116	62	+	3572331	-	epuA protein	
M5005_Spy_0586	endA	**NE**	584152	584691	179	+	3572332	-	DNA-entry nuclease	

M5005_Spy_0587	pheS	**E**	584974	586017	347	+	3572333	COG0016J	phenylalanyl-tRNA synthetase subunit alpha	*
M5005_Spy_0588	pheT	**E**	586212	588632	806	+	3572334	COG0072J	phenylalanyl-tRNA synthetase subunit beta	*
M5005_Spy_0589	-	**NE**	588742	589119	125	+	3572335	COG1765O	salt-stress induced protein	*
M5005_Spy_0590	-	**NE**	589112	589498	128	+	3572336	COG3272S	hypothetical protein M5005_Spy_0590	*
M5005_Spy_0591	-	**NE**	589571	590647	358	+	3572337	COG0577V	ABC transporter permease	*
M5005_Spy_0592	-	**NE**	590657	591325	222	+	3572338	COG1136V	ABC transporter ATP-binding protein	*
M5005_Spy_0593c	-	**NE**	591427	592344	305	-	3572300	COG2321R	neutral zinc metallopeptidase	*
M5005_Spy_0594	rexB	**NC**	592495	595710	1071	+	3572301	COG3857L	ATP-dependent nuclease subunit B	*
M5005_Spy_0595	rexA	**C**	595671	599339	1222	+	3572302	COG1074L	ATP-dependent nuclease subunit A	*
M5005_Spy_0596	-	**NE**	599479	600291	270	+	3572303	COG0834ET	arginine-binding protein	*
M5005_Spy_0597	rpsU	**E**	600432	600608	58	+	3572304	-	30S ribosomal protein S21	
M5005_Spy_0598c	mscL	**NE**	600736	601098	120	-	3572305	COG1970M	large-conductance mechanosensitive channel	*
M5005_Spy_0599	dnaG	**E**	601229	603043	604	+	3572306	COG0358L	DNA primase	
M5005_Spy_0600	rpoD	**E**	603052	604161	369	+	3572307	COG0568K	RNA polymerase sigma factor RpoD	*
M5005_Spy_0601	-	**NE**	604397	604735	112	+	3572308	COG2151R	hypothetical protein M5005_Spy_0601	*
M5005_Spy_0602	rmlD	**E**	604873	605727	284	+	3572309	COG1091M	dTDP-4-dehydrorhamnose reductase	
M5005_Spy_0603	rgpAc	**E**	605846	607000	384	+	3572310	COG0438M	alpha-(1,2)-rhamnosyltransferase	*
M5005_Spy_0604	rgpBc	**E**	606990	607922	310	+	3572311	COG0463M	alpha-L-Rha alpha-1,3-L-rhamnosyltransferase	*
M5005_Spy_0605	rgpCc	**E**	607925	608728	267	+	3572312	COG1682GM	polysaccharide export ABC transporter permease	*
M5005_Spy_0606	rgpDc	**E**	608728	609933	401	+	3572313	COG1134GM	polysaccharide export ATP-binding protein	*
M5005_Spy_0607	rgpEc	**E**	609958	610965	335	+	3572314	COG0463M	glycosyltransferase	*
M5005_Spy_0608	rgpFc	**E**	610962	612707	581	+	3572315	COG3754M	alpha-L-Rha alpha-1,3-L-rhamnosyltransferase	*
M5005_Spy_0609	-	**NE**	612704	615178	824	+	3572316	COG1368M	phosphoglycerol transferase	*
M5005_Spy_0610	-	**NE**	615357	616052	231	+	3572317	COG0463M	glycosyltransferase	*
M5005_Spy_0611	-	**NE**	616054	616395	113	+	3572318	-	hypothetical protein M5005_Spy_0611	*
M5005_Spy_0612	amrA	**C**	616388	617674	428	+	3572280	COG2244R	transcriptional activator	*
M5005_Spy_0613	-	**E**	617655	619151	498	+	3572281	COG4713S	hypothetical protein M5005_Spy_0613	*
M5005_Spy_0614	pepT	**NE**	619245	620468	407	+	3572282	COG2195E	peptidase T	*
M5005_Spy_0615	ebsA	**NE**	620509	620997	162	+	3572283	-	pore forming protein	*
M5005_Spy_0616c	-	**NE**	620984	621181	65	-	3572284	COG1141C	ferredoxin	
M5005_Spy_0617	-	**NC**	621230	621706	158	+	3572285	-	hypothetical protein M5005_Spy_0617	*
M5005_Spy_0618	cmk	**C**	621721	622401	226	+	3572286	COG0283F	cytidylate kinase	*
M5005_Spy_0619	infC	**C**	622563	623093	176	+	3572287	COG0290J	translation initiation factor IF-3	*
M5005_Spy_0620	rpmI	**C**	623135	623332	65	+	3572288	COG0291J	50S ribosomal protein L35	
M5005_Spy_0621	rplT	**NC**	623391	623750	119	+	3572289	COG0292J	50S ribosomal protein L20	*
M5005_Spy_0622c	-	**E**	624041	626212	723	-	3572290	COG1368M	phosphoglycerol transferase	*
M5005_Spy_0623	-	**NE**	626359	627522	387	+	3572291	COG1092R	methyltransferase	*
M5005_Spy_0624	aroD	**NE**	627519	628205	228	+	3572292	COG0710E	3-dehydroquinate dehydratase	*
M5005_Spy_0625	aroF	**NE**	628299	629465	388	+	3572293	COG0082E	chorismate synthase	*
M5005_Spy_0626	-	**NE**	629526	629867	113	+	3572294	COG3679S	hypothetical protein M5005_Spy_0626	*
M5005_Spy_0627	gor	**NE**	630088	631440	450	+	3572295	COG1249C	glutathione reductase	*
M5005_Spy_0628c	folC.2	**NE**	631528	632796	422	-	3572296	COG0285H	folylpolyglutamate synthase/dihydrofolate synthase	*
M5005_Spy_0629c	-	**NE**	632826	633266	146	-	3572297	-	hypothetical protein M5005_Spy_0629	*
M5005_Spy_0630	nifS1	**NE**	633501	634643	380	+	3572298	COG1104E	cysteine desulfhydrase	*
M5005_Spy_0631	thiI	**NE**	634655	635869	404	+	3572299	COG0301H	thiamine biosynthesis protein ThiI	*
M5005_Spy_0632	capA	**NE**	635907	637199	430	+	3572261	COG2843M	capsule biosynthesis protein	*
M5005_Spy_0633	rplU	**NC**	637413	637727	104	+	3572262	COG0261J	50S ribosomal protein L21	*
M5005_Spy_0634	-	**NC**	637739	638065	108	+	3572263	COG2868J	hypothetical protein M5005_Spy_0634	*
M5005_Spy_0635	rpmA	**NC**	638093	638386	97	+	3572264	COG0211J	50S ribosomal protein L27	*
M5005_Spy_0636	-	**NE**	638734	639648	304	+	3572265	COG0583K	LysR family transcriptional regulator	*
M5005_Spy_0637	lsp	**NE**	639645	640103	152	+	3572266	COG0597MU	lipoprotein signal peptidase	*
M5005_Spy_0638	-	**NE**	640093	640983	296	+	3572267	COG0564J	ribosomal large subunit pseudouridine synthase D	*
M5005_Spy_0639	pyrR	**NE**	641379	641900	173	+	3572268	COG2065F	phosphoribosyltransferase	*
M5005_Spy_0640	pyrP	**NE**	641916	643175	419	+	3572269	COG2233F	uracil permease	*
M5005_Spy_0641	pyrB	**NE**	643236	644171	311	+	3572270	COG0540F	aspartate carbamoyltransferase	*
M5005_Spy_0642	carA	**NE**	644215	645297	360	+	3572271	COG0505EF	carbamoyl phosphate synthase small subunit	*
M5005_Spy_0643	carB	**NE**	645523	648699	1058	+	3572272	COG0458EF	carbamoyl phosphate synthase large subunit	*
M5005_Spy_0644	-	**NE**	648999	650177	392	+	3572273	COG0845M	periplasmic protein of efflux system	*
M5005_Spy_0645	-	**NE**	650177	650887	236	+	3572274	COG1136V	ABC transporter ATP-binding protein	*

M5005_Spy_0646	-	**NE**	650899	652119	406	+	3572275 COG0577V	ABC transporter permease	*
M5005_Spy_0647	-	**NE**	652373	654106	577	+	3572276 COG4781C	glycerophosphoryl diester phosphodiesterase	*
M5005_Spy_0648	rpsP	**NE**	654233	654505	90	+	3572277 COG0228J	30S ribosomal protein S16	
M5005_Spy_0649	-	**NE**	654515	654754	79	+	3572278 COG1837R	RNA binding protein	
M5005_Spy_0650	-	**NC**	654763	654870	35	+	3572279 -	hypothetical protein M5005_Spy_0650	
M5005_Spy_0651	-	**NE**	655665	658691	1008	+	3572241 -	cell surface protein	*
M5005_Spy_0652	-	**NE**	658711	659112	133	+	3572242 COG3576R	hypothetical protein M5005_Spy_0652	*
M5005_Spy_0653c	czcD	**NE**	659292	660167	291	-	3572243 COG1230P	cobalt-zinc-cadmium resistance protein	*
M5005_Spy_0654	-	**NE**	660303	660824	173	+	3572244 COG1309K	TetR family transcriptional regulator	*
M5005_Spy_0655	rimM	**NE**	661039	661557	172	+	3572245 COG0806J	16S rRNA-processing protein RimM	*
M5005_Spy_0656	trmD	**E**	661547	662278	243	+	3572246 COG0336J	tRNA (guanine-N(1)-)-methyltransferase	*
M5005_Spy_0657	trxB	**NE**	662278	663270	330	+	3572247 COG0492O	thioredoxin reductase	*
M5005_Spy_0658	-	**NE**	663448	664506	352	+	3572248 COG3641R	regulatory protein	*
M5005_Spy_0659	apbA	**NE**	664519	665442	307	+	3572249 COG1893H	2-dehydropantoate 2-reductase	*
M5005_Spy_0660	fruR	**NE**	665698	666411	237	+	3572250 COG1349KG	fructose repressor	*
M5005_Spy_0661	fruB	**NE**	666408	667319	303	+	3572251 COG1105G	1-phosphofructokinase	*
M5005_Spy_0662	fruA	**NE**	667316	669262	648	+	3572252 COG1299G	IIABC	*
M5005_Spy_0663	mur1.1	**NE**	669361	669954	197	+	3572253 COG1705NU	autolysin	*
M5005_Spy_0664	mur1.2	**NE**	670106	670813	235	+	3572254 COG1705NU	autolysin	*
M5005_Spy_0665c	-	**NE**	670870	671088	72	-	3572255 COG2801L	transposase	
M5005_Spy_0666c	-	**NC**	671291	671410	39	-	3572256 -	hypothetical protein M5005_Spy_0666	
M5005_Spy_0667c	-	**NC**	671385	671594	69	-	3572257 -	exotoxin type C	
M5005_Spy_0668c	mac	**NE**	671769	672788	339	-	3572258 -	IgG-degrading protease	
M5005_Spy_0669	-	**NC**	672944	673063	39	+	3572259 -	phage protein	
M5005_Spy_0670	-	**NE**	673287	673463	58	+	3572260 COG0105F	nucleoside diphosphate kinase	
M5005_Spy_0671	-	**NE**	673691	674071	126	+	3572222 COG4835S	transposase	*
M5005_Spy_0672	-	**NE**	674071	674919	282	+	3572223 COG1307S	degV family protein	*
M5005_Spy_0673	papS	**E**	675044	676252	402	+	3572224 COG0617J	tRNA CCA-pyrophosphorylase	*
M5005_Spy_0674	-	**NE**	676249	678126	625	+	3572225 COG0488R	ABC transporter ATP-binding protein	*
M5005_Spy_0675	-	**NC**	678383	678535	50	+	3572226 -	hypothetical protein M5005_Spy_0675	
M5005_Spy_0676	-	**NE**	678544	678762	72	+	3572227 -	hypothetical protein M5005_Spy_0676	
M5005_Spy_0677	fms	**NE**	679018	679428	136	+	3572228 COG0242J	peptide deformylase	*
M5005_Spy_0678c	-	**NE**	679510	681522	670	-	3572229 COG0737F	5\'-nucleotidase	*
M5005_Spy_0679	-	**NE**	681742	682392	216	+	3572230 COG2357S	GTP pyrophosphokinase	*
M5005_Spy_0680	-	**NE**	682395	683063	222	+	3572231 COG0745TK	two-component response regulator	*
M5005_Spy_0681	-	**NE**	683072	684304	410	+	3572232 COG0642T	two-component system histidine kinase	*
M5005_Spy_0682	mvaK1	**E**	684583	685476	297	+	3572233 COG1577I	mevalonate kinase	*
M5005_Spy_0683	mvaD	**E**	685458	686402	314	+	3572234 COG3407I	diphosphomevalonate decarboxylase	*
M5005_Spy_0684	mvaK2	**NC**	686395	687402	335	+	3572235 COG1577I	phosphomevalonate kinase	*
M5005_Spy_0685	-	**E**	687395	688384	329	+	3572236 COG1304C	isopentenyl pyrophosphate isomerase	*
M5005_Spy_0686c	-	**C**	688614	689891	425	-	3572237 COG1257I	3-hydroxy-3-methylglutaryl-CoA reductase	*
M5005_Spy_0687c	mvaS.1	**E**	689878	691053	391	-	3572238 COG3425I	hydroxymethylglutaryl-CoA synthase	*
M5005_Spy_0688	thyA	**NC**	691262	692101	279	+	3572239 COG0207F	thymidylate synthase	*
M5005_Spy_0689	dyr	**E**	692181	692678	165	+	3572240 COG0262H	dihydrofolate reductase	*
M5005_Spy_0690	-	**NE**	692698	692868	56	+	3572202 -	hypothetical protein M5005_Spy_0690	
M5005_Spy_0691	clpX	**NE**	692998	694227	409	+	3572203 COG1219O	ATP-dependent protease ATP-binding subunit ClpX	*
M5005_Spy_0692	engB	**E**	694237	694836	199	+	3572204 COG0218R	ribosome biogenesis GTP-binding protein YsxC	*
M5005_Spy_0693	-	**NE**	694984	695727	247	+	3572205 -	hypothetical protein M5005_Spy_0693	*
M5005_Spy_0694c	clpL	**NE**	695785	697884	699	-	3572206 COG0542O	ATP-dependent protease ATP-binding subunit	*
M5005_Spy_0695	rpiA	**C**	698263	698946	227	+	3572207 COG0120G	ribose-5-phosphate isomerase A	*
M5005_Spy_0696	deoB	**NE**	699023	700234	403	+	3572208 COG1015G	phosphopentomutase	*
M5005_Spy_0697	arsC	**NE**	700253	700693	146	+	3572209 COG1393P	arsenate reductase	*
M5005_Spy_0698	punA	**NC**	700677	701486	269	+	3572210 COG0005F	purine nucleoside phosphorylase	*
M5005_Spy_0699	deoD	**NE**	702149	702862	237	+	3572211 COG0813F	purine nucleoside phosphorylase	*
M5005_Spy_0700	cpsX	**NE**	702855	703643	262	+	3572212 -	LytR family transcriptional regulator	
M5005_Spy_0701c	cpsY	**NE**	703722	704576	284	-	3572213 COG0583K	LysR family transcriptional regulator	*
M5005_Spy_0702	-	**NE**	704849	705385	178	+	3572214 -	hypothetical protein M5005_Spy_0702	*
M5005_Spy_0703	pyrF	**NE**	705662	706354	230	+	3572215 COG0284F	orotidine 5\'-phosphate decarboxylase	*
M5005_Spy_0704	pyrE	**NE**	706412	707041	209	+	3572216 COG0461F	orotate phosphoribosyltransferase	*

M5005_Spy_0705	amiC	**NE**	707238	708692	484	+	3572217 COG0154J	amidase	*
M5005_Spy_0706	-	**NE**	708810	709673	287	+	3572218 COG0834ET	cystine-binding protein	*
M5005_Spy_0707	-	**NE**	709702	710352	216	+	3572219 COG0765E	cystine transporter permease	*
M5005_Spy_0708	ung	**NE**	710485	711138	217	+	3572220 COG0692L	uracil-DNA glycosylase	*
M5005_Spy_0709	pyrC	**NE**	711270	712538	422	+	3572221 COG0044F	dihydroorotase	*
M5005_Spy_0710c	-	**E**	712596	713237	213	-	3572186 COG0344S	glycerol-3-phosphate acyltransferase	*
M5005_Spy_0711	parE	**E**	713372	715321	649	+	3572187 COG0187L	DNA topoisomerase IV subunit B	*
M5005_Spy_0712	parC	**E**	715412	717871	819	+	3572188 COG0188L	DNA topoisomerase IV subunit A	*
M5005_Spy_0713	bcaT	**NE**	717994	719016	340	+	3572189 COG0115EH	branched-chain amino acid aminotransferase	*
M5005_Spy_0714	-	**NC**	719080	719310	76	+	3572190 -	hypothetical protein M5005_Spy_0714	
M5005_Spy_0715	rpsA	**E**	719701	720906	401	+	3572193 COG0539J	30S ribosomal protein S1	*
M5005_Spy_0716c	-	**NE**	721453	721755	100	-	3572195 -	hypothetical protein M5005_Spy_0716	
M5005_Spy_0717	-	**NE**	721954	722940	328	+	3572196 COG1054R	hypothetical protein M5005_Spy_0717	*
M5005_Spy_0718	-	**NE**	723011	723202	63	+	3572197 -	hypothetical protein M5005_Spy_0718	
M5005_Spy_0719	-	**NE**	723251	723670	139	+	3572198 COG0625O	glutathione S-transferase	
M5005_Spy_0720	-	**NE**	723907	724815	302	+	3572199 COG1275P	exfoliative toxin	
M5005_Spy_0721c	-	**NC**	724933	725109	58	-	3572200 -	hypothetical protein M5005_Spy_0721	
M5005_Spy_0722	miaA	**NE**	725243	726142	299	+	3572201 COG0324J	tRNA delta(2)-isopentenylpyrophosphate transferase	*
M5005_Spy_0723	hflX	**NE**	726215	727453	412	+	3572166 COG2262R	GTP-binding protein	*
M5005_Spy_0724	-	**NE**	727446	728081	211	+	3572167 COG4468G	hypothetical protein M5005_Spy_0724	*
M5005_Spy_0725	elaC	**E**	728096	729025	309	+	3572168 COG1234R	ribonuclease Z	*
M5005_Spy_0726	-	**NE**	729025	729789	254	+	3572169 COG0300R	short chain dehydrogenase	*
M5005_Spy_0727	recJ	**NE**	729786	731996	736	+	3572170 COG0608L	single-stranded-DNA-specific exonuclease	*
M5005_Spy_0728	apt	**NE**	732147	732665	172	+	3572171 COG0503F	adenine phosphoribosyltransferase	*
M5005_Spy_0729	dnaD	**NC**	732746	733429	227	+	3572172 COG3935L	DNA replication protein	*
M5005_Spy_0730	nth	**NE**	733426	734082	218	+	3572173 COG0177L	endonuclease III	*
M5005_Spy_0731	-	**E**	734154	734738	194	+	3572174 COG2384R	I/anthranilate synthase component I	*
M5005_Spy_0732	-	**NE**	734829	735617	262	+	3572175 COG0327S	NIF3-related protein	*
M5005_Spy_0733	-	**NE**	735657	736763	368	+	3572176 COG0665E	glycine/D-amino acid oxidase	*
M5005_Spy_0734	cpsFO/rmlA	**E**	736821	737690	289	+	3572177 COG1209M	glucose-1-phosphate thymidylyltransferase	*
M5005_Spy_0735	cpsFP/rmlC	**NC**	737690	738283	197	+	3572178 COG1898M	dTDP-4-dehydrorhamnose 3,5-epimerase	*
M5005_Spy_0736	cpsFQ/rmlB	**E**	738527	739567	346	+	3572179 COG1088M	dTDP-glucose 4,6-dehydratase	*
M5005_Spy_0737	mutX	**NE**	739788	740264	158	+	3572180 COG1051F	7,8-dihydro-8-oxoguanine-triphosphatase	*
M5005_Spy_0738	-	**NE**	740322	741503	393	+	3572181 COG0628R	hypothetical protein M5005_Spy_0738	*
M5005_Spy_0739	-	**NE**	741493	742740	415	+	3572182 COG2956G	hypothetical protein M5005_Spy_0739	*
M5005_Spy_0740c	fbp	**NE**	742799	744085	428	-	3572183 COG1293K	fibronectin-binding protein	*
M5005_Spy_0742	-	**NC**	744497	744622	41	+	3572185 -	hypothetical protein M5005_Spy_0742	
M5005_Spy_0743	-	**NE**	744804	745802	332	+	3572147 COG2984R	ABC transporter substrate-binding protein	*
M5005_Spy_0744	-	**NC**	745792	745971	59	+	3572148 -	hypothetical protein M5005_Spy_0744	
M5005_Spy_0745	-	**NE**	746148	747017	289	+	3572149 COG4120R	ABC transporter permease	*
M5005_Spy_0746	-	**NE**	747014	747772	252	+	3572150 COG1101R	ABC transporter ATP-binding protein	*
M5005_Spy_0747	-	**E**	747976	749637	553	+	3572151 COG0595R	Zn-dependent hydrolase	*
M5005_Spy_0748	estA	**NE**	749770	750555	261	+	3572152 COG0627R	acetyl esterase	*
M5005_Spy_0749	-	**NE**	750585	750911	108	+	3572153 -	hypothetical protein M5005_Spy_0749	*
M5005_Spy_0750	-	**NE**	750957	752864	635	+	3572154 COG0488R	ABC transporter ATP-binding protein	*
M5005_Spy_0751	acoA	**NE**	753149	754117	322	+	3572155 COG1071C	alpha	*
M5005_Spy_0752	acoB	**NE**	754173	755174	333	+	3572156 COG0022C	pyruvate dehydrogenase E1 component subunit beta	*
M5005_Spy_0753	acoC	**NE**	755359	756768	469	+	3572157 COG0508C	subunit E2	*
M5005_Spy_0754c	-	**NE**	756823	757062	79	-	3572158 -	hypothetical protein M5005_Spy_0754	
M5005_Spy_0755	acoL	**NE**	757095	758858	587	+	3572159 COG1249C	dihydrolipoamide dehydrogenase	*
M5005_Spy_0756c	-	**NE**	758874	759086	70	-	3572160 -	hypothetical protein M5005_Spy_0756	
M5005_Spy_0757c	hylA	**NE**	759532	761949	805	-	3572161 -	hyaluronate lyase	
M5005_Spy_0758	lplB	**NE**	762182	763171	329	+	3572162 COG0095H	lipoate-protein ligase A	*
M5005_Spy_0759c	cobQ	**E**	763280	764071	263	-	3572163 COG3442R	hypothetical protein M5005_Spy_0759	*
M5005_Spy_0760c	murC2	**E**	764071	765414	447	-	3572164 COG0769M	diaminopimelate ligase	*
M5005_Spy_0761	-	**E**	765521	766372	283	+	3572165 COG1624S	hypothetical protein M5005_Spy_0761	*
M5005_Spy_0762	-	**NE**	766369	767325	318	+	3572127 COG4856S	hypothetical protein M5005_Spy_0762	*
M5005_Spy_0763	glmM	**E**	767379	768734	451	+	3572128 COG1109G	phosphoglucosamine mutase	*
M5005_Spy_0764	-	**NE**	768868	769509	213	+	3572129 -	hypothetical protein M5005_Spy_0764	*

M5005_Spy_0765	hemN	NE	769506	770702	398	+	3572130 COG0635H	coproporphyrinogen III oxidase	*
M5005_Spy_0766	-	C	770712	771464	250	+	3572131 COG3884I	acyl-ACP thioesterase	*
M5005_Spy_0767	-	NE	771464	772228	254	+	3572132 COG0647G	4-nitrophenylphosphatase	*
M5005_Spy_0768	-	NE	772228	772860	210	+	3572133 COG4478S	hypothetical protein M5005_Spy_0768	
M5005_Spy_0769	cas9	NE	773340	777446	1368	+	3572134 COG3513S	hypothetical protein M5005_Spy_0769	
M5005_Spy_0770	cas1	NE	777446	778315	289	+	3572135 COG1518L	hypothetical protein M5005_Spy_0770	
M5005_Spy_0771	cas2	NE	778312	778653	113	+	3572136 COG3512S	hypothetical protein M5005_Spy_0771	
M5005_Spy_0772	csn2	NE	778643	779305	220	+	3572137 -	hypothetical protein M5005_Spy_0772	
M5005_Spy_0773c	-	NE	779352	779639	95	-	3572138 -	hypothetical protein M5005_Spy_0773	
M5005_Spy_0774	-	NE	779772	779951	59	+	3572139 -	nucleoside diphosphate kinase	
M5005_Spy_0775	-	NC	780015	780161	48	+	3572140 COG0105F	nucleoside diphosphate kinase	
M5005_Spy_0776	lepA	NE	780285	782117	610	+	3572141 COG0481M	GTP-binding protein LepA	
M5005_Spy_0777	sclB	NE	782375	783256	293	+	3572142 -	hypothetical protein M5005_Spy_0777	
M5005_Spy_0778	msrB/crsA	NE	783442	783879	145	+	3572143 COG0229O	methionine sulfoxide reductase B	*
M5005_Spy_0779	-	NE	783994	785013	339	+	3572144 COG2855S	hypothetical protein M5005_Spy_0779	*
M5005_Spy_0780	-	NE	785220	785645	141	+	3572145 COG2893G	subunit IIA	*
M5005_Spy_0781	ptsB	NE	785664	786155	163	+	3572146 COG3444G	subunit IIB	*
M5005_Spy_0782	ptsC	NE	786172	786981	269	+	3572108 COG3715G	subunit IIC	*
M5005_Spy_0783	ptsD	NE	786978	787805	275	+	3572109 COG3716G	subunit IID	*
M5005_Spy_0784	-	NE	787941	789590	549	+	3572110 COG2972T	two-component sensor kinase	*
M5005_Spy_0785	-	NE	789594	790382	262	+	3572111 COG4753T	two-component response regulator	*
M5005_Spy_0786	-	NE	790376	791422	348	+	3572112 COG1840P	iron(III)-binding protein	*
M5005_Spy_0787	-	NE	791519	791866	115	+	3572113 COG0727R	hypothetical protein M5005_Spy_0787	
M5005_Spy_0788	-	NE	792201	792767	188	+	3572114 COG0110R	acetyltransferase	
M5005_Spy_0789	-	NE	792783	793472	229	+	3572115 COG1011R	HAD superfamily hydrolase	
M5005_Spy_0790	gabD	NE	793567	794964	465	+	3572116 COG1012C	succinate-semialdehyde dehydrogenase	*
M5005_Spy_0791	uvrC	NE	795066	796862	598	+	3572117 COG0322L	excinuclease ABC subunit C	*
M5005_Spy_0792	-	NE	797047	797649	200	+	3572118 COG0778C	NAD(P)H-dependent quinone reductase	*
M5005_Spy_0793	-	NE	797774	799183	469	+	3572119 COG0624E	dipeptidase PepV	*
M5005_Spy_0794c	trmE	C	799251	800627	458	-	3572120 COG0486R	tRNA modification GTPase TrmE	*
M5005_Spy_0795	rplJ	NE	800960	801460	166	+	3572121 COG0244J	50S ribosomal protein L10	*
M5005_Spy_0796	rplL	NE	801525	801890	121	+	3572122 COG0222J	50S ribosomal protein L7/L12	*
M5005_Spy_0797	-	NC	802286	802426	46	+	3572123 -	hypothetical protein M5005_Spy_0797	
M5005_Spy_0798	-	NE	802420	802788	122	+	3572124 -	IFN-response binding factor 1	
M5005_Spy_0799	-	NC	802767	802904	45	+	3572125 -	hypothetical protein M5005_Spy_0799	
M5005_Spy_0800	-	NE	802963	803286	107	+	3572126 COG0270L	DNA-cytosine methyltransferase	
M5005_Spy_0801	-	NC	803344	803466	40	+	3572088 -	relaxase	
M5005_Spy_0802	-	NE	803599	803811	70	+	3572089 -	relaxase	
M5005_Spy_0803	srtI	NE	804076	804771	231	+	3572090 -	lantibiotic production protein	
M5005_Spy_0804	srtR	NE	804922	805608	228	+	3572091 COG0745TK	nisin biosynthesis two-component response regulator	
M5005_Spy_0805	srtK	NE	805601	806947	448	+	3572092 COG0642T	nisin biosynthesis sensor protein	
M5005_Spy_0806	srtA	NC	807095	807235	46	+	3572093 -	lantibiotic protein	
M5005_Spy_0807	srtT	NE	807350	807967	205	+	3572094 COG1132V	lantibiotic ABC transporter ATP-binding protein	
M5005_Spy_0808	srtF	NE	808049	808738	229	+	3572095 COG1131V	lantibiotic ABC transporter ATP-binding protein	
M5005_Spy_0809	srtE	NE	808744	809493	249	+	3572096 -	lantibiotic transport permease	
M5005_Spy_0810	srtG	NE	809496	810218	240	+	3572097 COG4200S	lantibiotic transport permease	
M5005_Spy_0811	-	NE	810410	810628	72	+	3572098 COG1396K	Cro/CI family transcriptional regulator	
M5005_Spy_0812c	-	NE	810737	810952	71	-	3572099 -	hypothetical protein M5005_Spy_0812	
M5005_Spy_0817	dacA1	NE	812371	813699	442	+	3572104 COG1686M	D-alanyl-D-alanine carboxypeptidase	*
M5005_Spy_0818c	-	NE	813816	814778	320	-	3572105 COG0726G	polysaccharide deacetylase	*
M5005_Spy_0819	-	NC	814866	815021	51	+	3572106 -	hypothetical protein M5005_Spy_0819	
M5005_Spy_0820	folC.1	E	815110	816387	425	+	3572107 COG0285H	folylpolyglutamate synthase/dihydrofolate synthase	
M5005_Spy_0821	folE	NE	816434	817000	188	+	3572069 COG0302H	GTP cyclohydrolase I	*
M5005_Spy_0822	folP	C	817009	817809	266	+	3572070 COG0294H	dihydropteroate synthase	*
M5005_Spy_0823	folQ	NE	817816	818175	119	+	3572071 COG1539H	dihydroneopterin aldolase	*
M5005_Spy_0824	folK	NC	818172	818672	166	+	3572072 COG0801H	hydroxymethyldihydropteridine pyrophosphokinase	*
M5005_Spy_0825	murB	E	818822	819709	295	+	3572073 COG0812M	UDP-N-acetylenolpyruvoylglucosamine reductase	*
M5005_Spy_0826	potA	NE	819755	820909	384	+	3572074 COG3842E	protein	*
M5005_Spy_0827	potB	NE	820893	821687	264	+	3572075 COG1176E	spermidine/putrescine transporter permease	*

M5005_Spy_0828	potC	**NE**	821684	822460	258	+	3572076 COG1177E	spermidine/putrescine transporter permease	*
M5005_Spy_0829	potD	**NE**	822453	823526	357	+	3572077 COG0687E	spermidine/putrescine-binding protein	*
M5005_Spy_0830c	dpiA	**NE**	823581	824246	221	-	3572078 COG4565KT	transcriptional regulatory protein	*
M5005_Spy_0831c	dpiB	**NE**	824227	825768	513	-	3572079 COG3290T	sensor kinase	*
M5005_Spy_0832	malP	**NE**	825929	827260	443	+	3572080 COG3493C	malate-sodium symport	*
M5005_Spy_0833	-	**NE**	827291	828457	388	+	3572081 COG0281C	NAD-dependent malic enzyme	*
M5005_Spy_0834c	-	**NE**	828540	829631	363	-	3572082 COG1063ER	dehydrogenase	
M5005_Spy_0835	aphA	**NE**	829825	830187	120	+	3572083 COG3700R	class B acid phosphatase	*
M5005_Spy_0837	-	**NE**	830733	832265	510	+	3572085 COG0038P	chloride channel protein	*
M5005_Spy_0838	-	**NE**	832428	833042	204	+	3572086 COG2755E	lipase/acylhydrolase	*
M5005_Spy_0839	-	**NE**	833222	834349	375	+	3572087 COG0628R	hypothetical protein M5005_Spy_0839	*
M5005_Spy_0840	radC	**NE**	834398	835078	226	+	3572049 COG2003L	DNA repair protein RadC	*
M5005_Spy_0841c	-	**NE**	835080	835775	231	-	3572050 COG2071R	glutamine amidotransferase, class I	*
M5005_Spy_0842c	-	**NE**	835785	836429	214	-	3572051 COG2344R	redox-sensing transcriptional repressor Rex	*
M5005_Spy_0843c	-	**NE**	836681	837028	115	-	3572052 -	hypothetical protein M5005_Spy_0843	*
M5005_Spy_0844c	nifS2	**E**	837018	838145	375	-	3572053 COG1104E	cysteine desulfhydrase	*
M5005_Spy_0845c	prs	**NE**	838142	839122	326	-	3572054 COG0462FE	ribose-phosphate pyrophosphokinase	*
M5005_Spy_0846c	-	**NE**	839262	839840	192	-	3572055 COG4116S	adenylate cyclase	*
M5005_Spy_0847	-	**NE**	839928	840599	223	+	3572056 COG2357S	GTP pyrophosphokinase	*
M5005_Spy_0848	ppnK	**E**	840574	841410	278	+	3572057 COG0061G	inorganic polyphosphate/ATP-NAD kinase	*
M5005_Spy_0849	rluD	**NE**	841407	842312	301	+	3572058 COG0564J	ribosomal large subunit pseudouridine synthase D	*
M5005_Spy_0851	pta/eutD	**NE**	842489	843310	273	+	3572060 COG0280C	phosphotransacetylase	*
M5005_Spy_0852	-	**NE**	843437	844054	205	+	3572061 COG4221R	short chain dehydrogenase	
M5005_Spy_0853	-	**NC**	844051	844209	52	+	3572062 COG4221R	short chain dehydrogenase	
M5005_Spy_0854c	-	**NE**	844403	845092	229	-	3572063 -	Na+ driven multidrug efflux pump	*
M5005_Spy_0855	proV	**NE**	845512	846240	242	+	3572064 COG1125E	glycine betaine transport ATP-binding protein	
M5005_Spy_0856	proX	**NE**	846233	847759	508	+	3572065 COG1732M	glycine betaine transporter permease	
M5005_Spy_0857	guaC	**NE**	848037	849020	327	+	3572066 COG0516F	guanosine 5\'-monophosphate oxidoreductase	*
M5005_Spy_0858	xpt	**NE**	849325	849906	193	+	3572067 COG0503F	xanthine phosphoribosyltransferase	*
M5005_Spy_0859	-	**NE**	849906	851189	427	+	3572068 COG2233F	xanthine permease	*
M5005_Spy_0860c	apbE	**NE**	851253	852191	312	-	3572030 COG1477H	thiamine biosynthesis lipoprotein	*
M5005_Spy_0861c	-	**NE**	852244	852429	61	-	3572031 COG1942R	4-oxalocrotonate tautomerase	
M5005_Spy_0862	tdk2	**NE**	852567	853136	189	+	3572032 COG1435F	thymidine kinase	*
M5005_Spy_0863	prfA	**E**	853171	854250	359	+	3572033 COG0216J	peptide chain release factor 1	*
M5005_Spy_0864	hemK	**NE**	854250	855089	279	+	3572034 COG2890J	methyltransferase	*
M5005_Spy_0865	-	**NC**	855073	855663	196	+	3572035 COG0009J	SUA5 protein	*
M5005_Spy_0866	-	**NE**	855681	856133	150	+	3572036 COG0456R	phosphinothricin N-acetyltransferase	*
M5005_Spy_0867	glyA	**NE**	856123	857379	418	+	3572037 COG0112E	serine hydroxymethyltransferase	*
M5005_Spy_0868	-	**NE**	857386	858363	325	+	3572038 -	hypothetical protein M5005_Spy_0868	*
M5005_Spy_0869	-	**NE**	858364	858963	199	+	3572039 COG0741M	hypothetical protein M5005_Spy_0869	*
M5005_Spy_0870	-	**NE**	858973	860697	574	+	3572040 COG1132V	protein/permease	*
M5005_Spy_0871	-	**NE**	860694	862421	575	+	3572041 COG1132V	protein/permease	*
M5005_Spy_0872	nox	**NE**	862962	864032	356	+	3572042 COG0446R	NADH oxidase H2O-forming	*
M5005_Spy_0873c	ldh	**NE**	864191	865174	327	-	3572043 COG0039C	L-lactate dehydrogenase	*
M5005_Spy_0874	gyrA	**E**	865365	867851	828	+	3572044 COG0188L	DNA gyrase subunit A	*
M5005_Spy_0875	-	**E**	867871	868620	249	+	3572045 COG3764M	sortase	*
M5005_Spy_0876	-	**NE**	868700	869116	138	+	3572046 COG0346E	lactoylglutathione lyase	*
M5005_Spy_0877	-	**NC**	869603	869722	39	+	3572047 -	hypothetical protein M5005_Spy_0877	
M5005_Spy_0878	-	**NE**	869730	870098	122	+	3572048 -	hypothetical protein M5005_Spy_0878	*
M5005_Spy_0879c	-	**NE**	870148	871059	303	-	3572010 COG1597IR	hypothetical protein M5005_Spy_0879	
M5005_Spy_0880c	hlyIII	**C**	871176	871826	216	-	3572011 COG1272R	hypothetical protein M5005_Spy_0880	*
M5005_Spy_0881c	-	**NE**	871823	872263	146	-	3572012 -	hypothetical protein M5005_Spy_0881	*
M5005_Spy_0882	rbgA	**E**	872482	873330	282	+	3572013 COG1161R	ribosomal biogenesis GTPase	*
M5005_Spy_0883	rnhB	**NE**	873320	874111	263	+	3572014 COG0164L	ribonuclease HII	*
M5005_Spy_0884	smf	**NE**	874176	875012	278	+	3572015 COG0758LU	hypothetical protein M5005_Spy_0884	*
M5005_Spy_0885	topA	**C**	875119	877248	709	+	3572016 COG0550L	DNA topoisomerase I	*
M5005_Spy_0886c	-	**NE**	877323	877805	160	-	3572017 -	LysR family transcriptional regulator	
M5005_Spy_0887c	-	**NC**	877812	877934	40	-	3572018 -	LysR family transcriptional regulator	
M5005_Spy_0888c	-	**NC**	878080	878205	41	-	3572019 COG0583K	LysR family transcriptional regulator	

M5005_Spy_0889	-	NE	878218	878763	181	+	3572020 COG3641R	regulatory protein (pfoS/R)	
M5005_Spy_0890	ddh	NE	878778	879770	330	+	3572021 COG1052CHR	D-lactate dehydrogenase	
M5005_Spy_0891	satD	E	879875	880540	221	+	3572022 -	hypothetical protein M5005_Spy_0891	
M5005_Spy_0892	satE	NE	880533	881249	238	+	3572023 -	hypothetical protein M5005_Spy_0892	
M5005_Spy_0893	gid	NC	881400	882746	448	+	3572024 COG1206J	tRNA (uracil-5-)-methyltransferase Gid	*
M5005_Spy_0894	oadA2	NE	882899	884305	468	+	3572025 COG5016C	oxaloacetate decarboxylase	
M5005_Spy_0895	-	NE	884348	884662	104	+	3572026 -	hypothetical protein M5005_Spy_0895	
M5005_Spy_0896	-	NE	884662	885012	116	+	3572027 COG4770I	decarboxylase	
M5005_Spy_0897	-	NE	885022	886152	376	+	3572028 COG1883C	oxaloacetate decarboxylase subunit beta	
M5005_Spy_0898c	-	NE	886352	887236	294	-	3572029 COG1767H	A synthase	
M5005_Spy_0899c	citG	NE	887229	887924	231	-	3571991 COG1802K	GntR family transcriptional regulator	
M5005_Spy_0900c	-	NE	888082	889488	468	-	3571992 COG2851C	Mg2+/citrate complex secondary transporter	
M5005_Spy_0901	-	NE	889865	890188	107	+	3571993 -	hypothetical protein M5005_Spy_0901	
M5005_Spy_0902	-	NE	890242	890640	132	+	3571994 COG0511I	protein subunit	
M5005_Spy_0903	oadB	NE	890685	891806	373	+	3571995 COG1883C	oxaloacetate decarboxylase subunit beta	
M5005_Spy_0904	-	NC	891824	891958	44	+	3571996 -	hypothetical protein M5005_Spy_0904	
M5005_Spy_0905	citD	NE	891994	892302	102	+	3571997 COG3052C	citrate lyase subunit gamma	
M5005_Spy_0906	citE	NE	892320	893177	285	+	3571998 COG2301G	citrate lyase subunit beta/citryl-CoA lyase subunit	
M5005_Spy_0907	citF	NC	893180	894712	510	+	3571999 COG3051C	citrate lyase subunit alpha/citrate CoA-transferase	
M5005_Spy_0908	citX	NE	894642	895283	213	+	3572000 COG3697HI	A synthase	
M5005_Spy_0909	oadA1	NE	895298	896692	464	+	3572001 COG5016C	oxaloacetate decarboxylase	
M5005_Spy_0910c	citC	NE	896802	897854	350	-	3572002 COG3053C	(citrate (pro-3S)-lyase) ligase	*
M5005_Spy_0911c	-	NE	897943	898425	160	-	3572003 -	hypothetical protein M5005_Spy_0911	*
M5005_Spy_0912	-	NC	898910	899014	34	+	3572004 -	hypothetical protein M5005_Spy_0912	
M5005_Spy_0913c	xerS	C	899125	900039	304	-	3572005 COG4974L	site-specific tyrosine recombinase XerS	*
M5005_Spy_0914	-	NE	900733	901362	209	+	3572006 COG2932K	phage transcriptional repressor	*
M5005_Spy_0915c	ffh	E	901479	903041	520	-	3572007 COG0541U	signal recognition particle subunit FFH/SRP54	*
M5005_Spy_0916c	ylxM	C	903056	903397	113	-	3572008 COG2739S	DNA-binding protein	*
M5005_Spy_0917c	-	C	903486	904184	232	-	3572009 COG2188K	GntR family transcriptional regulator	*
M5005_Spy_0918c	-	NE	904267	905700	477	-	3571971 -	hypothetical protein M5005_Spy_0918	*
M5005_Spy_0919	guaA	C	905867	907429	520	+	3571972 COG0519F	GMP synthase	*
M5005_Spy_0920c	murM2	NE	907471	908694	407	-	3571973 COG2348V	alanyltransferase	
M5005_Spy_0921c	-	NE	909059	910603	514	-	3571974 COG0488R	ABC transporter ATP-binding protein	*
M5005_Spy_0922c	pdxK	NE	910737	911294	185	-	3571975 COG4720S	hypothetical protein M5005_Spy_0922	*
M5005_Spy_0923c	-	NE	911272	912138	288	-	3571976 COG2240H	pyridoxamine kinase	*
M5005_Spy_0924	-	NE	912228	913496	422	+	3571977 COG1167KE	GntR family transcriptional regulator	*
M5005_Spy_0925c	rnhB	NE	913894	914250	118	-	3571978 COG1328F	anaerobic ribonucleoside-triphosphate reductase	*
M5005_Spy_0926c	-	NC	914572	916149	525	-	3571979 COG1502I	cardiolipin synthetase	*
M5005_Spy_0927c	fhs.1	E	916234	917904	556	-	3571980 COG2759F	formate--tetrahydrofolate ligase	*
M5005_Spy_0928c	lplA	NE	918032	919051	339	-	3571981 COG0095H	lipoate-protein ligase A	*
M5005_Spy_0929c	-	NE	919098	919979	293	-	3571982 COG0846K	SIR2 family protein	*
M5005_Spy_0930c	-	NE	919972	920784	270	-	3571983 COG2110R	hypothetical protein M5005_Spy_0930	*
M5005_Spy_0931c	-	NE	920777	921109	110	-	3571984 COG0509E	glycine cleavage system protein H	*
M5005_Spy_0932c	-	NE	921151	922149	332	-	3571985 COG2141C	luciferase-like monooxygenase	*
M5005_Spy_0933c	-	NE	922146	923345	399	-	3571986 COG1902C	NADH-dependent flavin oxidoreductase	*
M5005_Spy_0934c	-	NE	923342	924178	278	-	3571987 COG0095H	lipoate-protein ligase A	*
M5005_Spy_0935	dpfB	C	924374	925066	230	+	3571988 COG0452H	phosphopantothenate--cysteine ligase	*
M5005_Spy_0936	dfp	E	925059	925604	181	+	3571989 COG0452H	phosphopantothenoylcysteine decarboxylase	*
M5005_Spy_0937	-	NE	925662	926231	189	+	3571990 COG4684S	transporter	*
M5005_Spy_0938	pgmA	E	926407	928125	572	+	3571952 COG1109G	phosphoglucomutase/phosphomannomutase	*
M5005_Spy_0939c	-	NE	928338	929294	318	-	3571953 COG1079R	nucleoside transporter permease	*
M5005_Spy_0940c	-	NE	929296	930360	354	-	3571954 COG4603R	nucleoside transporter permease	*
M5005_Spy_0941c	-	NE	930353	931903	516	-	3571955 COG3845R	nucleoside transport ATP-binding protein	*
M5005_Spy_0942c	-	NE	932024	933076	350	-	3571956 COG1744R	nucleoside-binding protein	*
M5005_Spy_0943c	cdd	NE	933170	933559	129	-	3571957 COG0295F	cytidine deaminase	*
M5005_Spy_0944c	-	NE	934218	934805	195	-	3571958 COG2813J	16S rRNA m(2)G 1207 methyltransferase	*
M5005_Spy_0945	coaA	E	935073	935993	306	+	3571959 COG1072H	pantothenate kinase	*
M5005_Spy_0946	rpsT	NE	936062	936295	77	+	3571960 COG0268J	30S ribosomal protein S20	
M5005_Spy_0947c	ciaH	NE	936420	937730	436	-	3571961 COG2205T	sensor protein	*

M5005_Spy_0948c	ciaR	NE	937723	938397	224	-	3571962 COG0745TK	transcriptional regulatory protein	*
M5005_Spy_0949c	pepN	NE	938743	941280	845	-	3571963 COG0308E	lysyl aminopeptidase/alanine aminopeptidase	*
M5005_Spy_0950c	phoU	NC	941485	942138	217	-	3571964 COG0704P	phosphate transporter protein	*
M5005_Spy_0951c	pstB	NE	942206	942964	252	-	3571965 COG1117P	phosphate transporter ATP-binding protein	*
M5005_Spy_0952c	pstB2	NE	942977	943780	267	-	3571966 COG1117P	phosphate transporter ATP-binding protein	*
M5005_Spy_0953c	pstA	NE	943796	944683	295	-	3571967 COG0581P	phosphate transporter permease	*
M5005_Spy_0954c	pstC	NC	944673	945491	272	-	3571968 COG0573P	phosphate transporter permease	*
M5005_Spy_0955c	pstS	NE	945618	946484	288	-	3571969 COG0226P	phosphate-binding protein	*
M5005_Spy_0956c	-	E	946623	947933	436	-	3571970 COG0144J	16S rRNA m(5)C 967 methyltransferase	*
M5005_Spy_0957c	-	NE	947936	948724	262	-	3571932 COG0483G	myo-inositol-1(or 4)-monophosphatase	*
M5005_Spy_0958c	-	NE	948714	948992	92	-	3571933 COG4476S	hypothetical protein M5005_Spy_0958	
M5005_Spy_0959c	spxA	NE	948994	949398	134	-	3571934 COG1393P	Spx family transcriptional regulator	*
M5005_Spy_0960c	mreA	E	949441	950373	310	-	3571935 COG0196H	adenylyltransferase	*
M5005_Spy_0961c	truB	NE	950402	951286	294	-	3571936 COG0130J	tRNA pseudouridine synthase B	*
M5005_Spy_0962c	-	NE	951402	952742	446	-	3571937 COG4487S	hypothetical protein M5005_Spy_0962	*
M5005_Spy_0963c	-	NE	952839	953795	318	-	3571938 COG4129S	hypothetical protein M5005_Spy_0963	*
M5005_Spy_0964c	-	NE	953806	954402	198	-	3571939 COG0732V	subunit	*
M5005_Spy_0965c	-	NE	954494	955669	391	-	3571940 COG0577V	ABC transporter permease	*
M5005_Spy_0967c	-	NE	957146	957847	233	-	3571942 COG1136V	ABC transporter ATP-binding protein	*
M5005_Spy_0968	-	NE	957965	958507	180	+	3571943 COG1309K	TetR family transcriptional regulator	*
M5005_Spy_0969c	-	NC	958504	958647	47	-	3571944 -	hypothetical protein M5005_Spy_0969	
M5005_Spy_0970c	-	NE	958650	959291	213	-	3571945 COG0490P	component	*
M5005_Spy_0971c	-	NE	959454	959942	162	-	3571946 COG1302S	Gls24 family general stress protein	*
M5005_Spy_0972c	-	NE	959953	960153	66	-	3571947 COG3237S	hypothetical protein M5005_Spy_0972	
M5005_Spy_0973c	-	NE	960194	960733	179	-	3571948 COG1302S	Gls24 family general stress protein	*
M5005_Spy_0974c	-	NC	960746	960934	62	-	3571949 COG5547S	small integral membrane protein	
M5005_Spy_0975c	-	NE	960945	961532	195	-	3571950 -	hypothetical protein M5005_Spy_0975	*
M5005_Spy_0976c	-	NE	961593	961799	68	-	3571951 COG2261S	hypothetical protein M5005_Spy_0976	*
M5005_Spy_0977c	pcrA	C	962205	964523	772	-	3571913 COG0210L	DNA helicase II	*
M5005_Spy_0978	-	NC	964891	965061	56	-	3571915 COG1115E	Na(+)-linked D-alanine glycine permease	*
M5005_Spy_0979c	-	C	965052	966374	440	+	3571914 -	hypothetical protein M5005_Spy_0979	
M5005_Spy_0980	-	NE	966494	967729	411	+	3571916 COG0053P	cobalt-zinc-cadmium resistance protein	*
M5005_Spy_0981c	cfa	NE	968098	968871	257	-	3571917 -	cAMP factor	
M5005_Spy_0982c	-	NE	969241	970077	278	-	3571918 COG0834ET	histidine-binding protein	
M5005_Spy_0983c	-	NC	970093	970722	209	-	3571919 COG1126E	histidine transport ATP-binding protein	
M5005_Spy_0984c	-	NE	970732	971373	213	-	3571920 COG0765E	histidine transporter permease	
M5005_Spy_0985c	-	NE	971479	971814	111	-	3571921 COG2824P	phnA protein	
M5005_Spy_0986c	glmS	E	972010	973824	604	-	3571922 COG0449M	aminotransferase	
M5005_Spy_0987c	sipC	NE	974000	974557	185	-	3571923 COG0681U	signal peptidase I	
M5005_Spy_0988c	pyk	E	974775	976277	500	-	3571924 COG0469G	pyruvate kinase	*
M5005_Spy_0989c	pfkA	E	976340	977353	337	-	3571925 COG0205G	6-phosphofructokinase	*
M5005_Spy_0990c	dnaE	E	977433	980543	1036	-	3571926 COG0587L	DNA polymerase III DnaE	*
M5005_Spy_0991	-	NC	980728	981099	123	+	3571927 COG1725K	GntR family transcriptional regulator	*
M5005_Spy_0992	-	NE	981099	981797	232	+	3571928 COG1131V	ABC transporter ATP-binding protein	*
M5005_Spy_0993	-	NE	981807	982592	261	+	3571929 -	ABC transporter permease	*
M5005_Spy_0994c	-	C	982719	983333	204	-	3571930 COG0398S	membrane-associated alkaline phosphatase	*
M5005_Spy_0995c	-	NE	983924	984112	62	-	3571931 -	phage protein	
M5005_Spy_0996	speA2	NE	984332	985087	251	+	3571893 -	enterotoxin	
M5005_Spy_0997c	-	NE	985209	985868	219	-	3571894 -	phage protein	
M5005_Spy_0998c	-	NE	985868	986089	73	-	3571895 -	phage protein	
M5005_Spy_0999c	-	NE	986099	986872	257	-	3571896 -	phage protein	
M5005_Spy_1000c	-	NE	986883	987485	200	-	3571897 -	phage protein	
M5005_Spy_1001c	-	NE	987497	988261	254	-	3571898 -	phage-associated cell wall hydrolase	
M5005_Spy_1002c	-	NE	988263	988595	110	-	3571899 -	N-acetylmuramoyl-L-alanine amidase	
M5005_Spy_1003c	-	NE	988595	988963	122	-	3571900 -	phage protein	
M5005_Spy_1004c	-	NC	988932	989096	54	-	3571901 -	phage protein	
M5005_Spy_1005c	-	NE	989068	989415	115	-	3571902 -	phage protein	
M5005_Spy_1006c	-	NE	989426	991177	583	-	3571903 COG4675S	phage structural protein	
M5005_Spy_1007c	-	NE	991293	994733	1146	-	3571904 COG1705NU	phage protein	

M5005_Spy_1008c	-	NE	994734	996218	494	-	3571905	COG4722S	hypothetical protein M5005_Spy_1008	
M5005_Spy_1009c	-	NE	996219	998024	601	-	3571906	COG5412S	phage protein	
M5005_Spy_1010c	-	NC	998017	998475	152	-	3571907	-	phage protein	
M5005_Spy_1011c	-	NE	998448	998765	105	-	3571908	-	phage protein	
M5005_Spy_1012c	-	NE	998778	999284	168	-	3571909	COG5437S	antigen A	
M5005_Spy_1013c	-	NE	999296	999706	136	-	3571910	-	antigen B	
M5005_Spy_1014c	-	NE	999708	1000103	131	-	3571911	-	antigen C	
M5005_Spy_1015c	-	NE	1000100	1000411	103	-	3571912	-	phage protein	
M5005_Spy_1016c	-	NE	1000408	1000752	114	-	3571874	-	phage protein	
M5005_Spy_1017c	-	NE	1000766	1001059	97	-	3571875	-	phage protein	
M5005_Spy_1018c	-	NE	1001072	1001962	296	-	3571876	-	phage protein	
M5005_Spy_1019c	-	NE	1001981	1002550	189	-	3571877	-	phage scaffold protein	
M5005_Spy_1020c	-	NE	1002795	1003064	89	-	3571878	-	phage protein	
M5005_Spy_1021c	-	NE	1003071	1003979	302	-	3571879	COG5585T	phage protein	
M5005_Spy_1022c	-	NE	1003948	1005273	441	-	3571880	-	portal protein	
M5005_Spy_1023c	-	NE	1005273	1006547	424	-	3571881	COG1783R	terminase large subunit	
M5005_Spy_1024c	-	NE	1006537	1006917	126	-	3571882	-	phage protein	
M5005_Spy_1025c	-	NE	1007527	1007961	144	-	3571883	-	ArpU family phage encoded transcriptional regulator	
M5005_Spy_1026c	-	NE	1008247	1008513	88	-	3571884	-	phage protein	
M5005_Spy_1027c	-	NE	1008510	1009034	174	-	3571885	-	phage protein	
M5005_Spy_1028c	-	NE	1009037	1009669	210	-	3571886	COG0286V	phage protein	
M5005_Spy_1029c	-	NE	1009671	1009955	94	-	3571887	-	phage protein	
M5005_Spy_1030c	-	NE	1009952	1010146	64	-	3571888	-	phage protein	
M5005_Spy_1031c	-	NE	1010119	1010358	79	-	3571889	-	phage protein	
M5005_Spy_1032c	-	NE	1010355	1010600	81	-	3571890	-	phage protein	
M5005_Spy_1033c	-	NE	1010597	1010953	118	-	3571891	-	phage protein	
M5005_Spy_1034c	-	NE	1010950	1011390	146	-	3571892	COG4570L	phage protein	
M5005_Spy_1035c	-	NE	1011390	1011593	67	-	3571854	-	phage protein	
M5005_Spy_1036c	ssb2	NE	1011599	1012024	141	-	3571855	COG0629L	phage single-strand DNA binding protein	
M5005_Spy_1037c	ssb1	NE	1012017	1012691	224	-	3571856	-	phage single-strand DNA binding protein	
M5005_Spy_1038c	-	NE	1012692	1013174	160	-	3571857	-	phage protein	
M5005_Spy_1039c	-	NE	1013196	1013450	84	-	3571858	-	phage protein	
M5005_Spy_1040c	-	NE	1013431	1013784	117	-	3571859	-	phage protein	
M5005_Spy_1041c	-	NC	1013797	1013934	45	-	3571860	-	phage protein	
M5005_Spy_1042c	-	NC	1013925	1014707	260	-	3571861	COG1484L	phage replication protein	
M5005_Spy_1043c	-	NE	1014694	1015524	276	-	3571862	COG3935L	phage protein	
M5005_Spy_1044	-	NE	1015738	1016199	153	+	3571863	-	phage protein	
M5005_Spy_1045c	-	NC	1016649	1016849	66	-	3571864	COG1396K	transcriptional regulator	
M5005_Spy_1046	-	NE	1016923	1017309	128	+	3571865	-	phage protein	
M5005_Spy_1047c	-	NE	1017298	1017507	69	-	3571866	-	phage protein	
M5005_Spy_1048	-	NE	1017561	1018160	199	+	3571867	-	phage protein	
M5005_Spy_1049c	-	NC	1018190	1018348	52	-	3571868	-	phage protein	
M5005_Spy_1050	-	C	1018705	1019529	274	+	3571869	COG2932K	phage transcriptional repressor	
M5005_Spy_1051	-	NE	1019565	1020458	297	+	3571870	-	phage protein	
M5005_Spy_1052	int.1	NE	1020579	1021667	362	+	3571871	COG4974L	integrase	
M5005_Spy_1053	-	NC	1021799	1021912	37	+	3571872	-	hypothetical protein M5005_Spy_1053	
M5005_Spy_1054	-	NE	1022030	1022650	206	+	3571873	-	hypothetical protein M5005_Spy_1054	
M5005_Spy_1055c	glgP	NE	1022907	1025171	754	-	3571835	COG0058G	glycogen phosphorylase	*
M5005_Spy_1056c	malM	NE	1025206	1026699	497	-	3571836	COG1640G	4-alpha-glucanotransferase	*
M5005_Spy_1057c	malR	NE	1026814	1027833	339	-	3571837	COG1609K	LacI family transcriptional regulator	*
M5005_Spy_1058	malE	NE	1027874	1029325	483	+	3571838	COG2182G	maltose/maltodextrin-binding protein	*
M5005_Spy_1059	malF	NE	1029830	1030960	376	+	3571839	COG1175G	maltose ABC transporter permease	*
M5005_Spy_1060	malG	NE	1030960	1031796	278	+	3571840	COG3833G	maltose ABC transporter permease	*
M5005_Spy_1061	-	NE	1031951	1032751	266	+	3571841	COG1609K	LacI family transcriptional regulator	
M5005_Spy_1062c	malA	NE	1032867	1033682	271	-	3571842	COG5521S	maltodextrose utilization protein	
M5005_Spy_1063c	malD	NE	1033704	1034564	286	-	3571843	COG3833G	maltodextrin transporter permease	
M5005_Spy_1064c	malC	NE	1034561	1035868	435	-	3571844	COG1175G	maltose ABC transporter permease	
M5005_Spy_1065c	amyA	NE	1035943	1038078	711	-	3571845	COG0366G	alpha-amylase	
M5005_Spy_1066c	amyB	NE	1038104	1039807	567	-	3571846	COG0366G	alpha-amylase	

M5005_Spy_1067c	malX	NE	1039973	1041232	419	-	3571847 COG2182G	maltose/maltodextrin-binding protein	
M5005_Spy_1068	-	NE	1041558	1041782	74	+	3571848 -	transposase	
M5005_Spy_1069c	-	NE	1041847	1042830	327	-	3571849 COG0657I	esterase	*
M5005_Spy_1070c	dltD	NE	1042861	1044111	416	-	3571850 COG3966M	hypothetical protein M5005_Spy_1070	*
M5005_Spy_1071c	dltC	NE	1044104	1044343	79	-	3571851 COG0236IQ	D-alanine--poly(phosphoribitol) ligase subunit 2	
M5005_Spy_1072c	dltB	NE	1044361	1045617	418	-	3571852 COG1696M	protein DltB	*
M5005_Spy_1073c	dltA	NE	1045614	1047152	512	-	3571853 COG1020Q	D-alanine--poly(phosphoribitol) ligase subunit 1	*
M5005_Spy_1074c	-	NC	1047164	1047307	47	-	3571815 -	hypothetical protein M5005_Spy_1074	
M5005_Spy_1075c	uvrB	NE	1047571	1049562	663	-	3571816 COG0556L	excinuclease ABC subunit B	*
M5005_Spy_1076	glnH	E	1049755	1051929	724	+	3571817 COG0765E	transporter	*
M5005_Spy_1077	glnQ.2	NC	1051929	1052669	246	+	3571818 COG1126E	glutamine ABC transporter ATP-binding protein	*
M5005_Spy_1078c	-	NC	1052817	1052969	50	-	3571819 -	hypothetical protein M5005_Spy_1078	
M5005_Spy_1079c	-	NE	1052966	1054345	459	-	3571820 COG1455G	IIC	
M5005_Spy_1080c	-	NE	1054523	1054972	149	-	3571821 -	hypothetical protein M5005_Spy_1080	*
M5005_Spy_1081c	-	NE	1054969	1055304	111	-	3571822 COG1447G	IIA	*
M5005_Spy_1082c	-	NE	1055307	1055618	103	-	3571823 COG1440G	IIB	*
M5005_Spy_1083c	-	NC	1055641	1057635	664	-	3571824 COG3711K	component	*
M5005_Spy_1084c	-	NE	1057741	1058835	364	-	3571825 COG3589S	outer surface protein	*
M5005_Spy_1085c	bglA.2	NE	1058844	1060244	466	-	3571826 COG2723G	beta-glucosidase	*
M5005_Spy_1086	-	NE	1060469	1061164	231	+	3571827 COG3201H	nicotinamide mononucleotide transporter	*
M5005_Spy_1087c	-	NC	1061169	1061327	52	-	3571828 -	hypothetical protein M5005_Spy_1087	*
M5005_Spy_1088c	obgE	E	1061396	1062703	435	-	3571829 COG0536R	GTPase ObgE	*
M5005_Spy_1089c	-	NC	1062766	1062894	42	-	3571830 -	hypothetical protein M5005_Spy_1089	
M5005_Spy_1090	-	NE	1063136	1063651	171	+	3571831 COG2963L	transposase	*
M5005_Spy_1091	-	NE	1063824	1064480	218	+	3571832 COG2801L	transposase	
M5005_Spy_1092c	rsuA	NE	1064529	1065263	244	-	3571833 COG1187J	ribosomal small subunit pseudouridine synthase A	*
M5005_Spy_1093	-	NE	1065375	1065740	121	+	3571834 COG5496R	hypothetical protein M5005_Spy_1093	*
M5005_Spy_1094c	-	NE	1065860	1067080	406	-	3571796 COG2814G	major facilitator transporter	*
M5005_Spy_1095	-	NE	1067382	1068938	518	+	3571797 COG4868S	hypothetical protein M5005_Spy_1095	*
M5005_Spy_1096c	-	NE	1069045	1069446	133	-	3571798 COG2050Q	thioesterase	*
M5005_Spy_1097c	-	NE	1069531	1070043	170	-	3571799 COG2820F	phosphorylase Pnp/Udp family protein	
M5005_Spy_1098c	-	NE	1070348	1071703	451	-	3571800 COG2265J	tRNA (uracil-5-)-methyltransferase	*
M5005_Spy_1099c	psr	NE	1071785	1073236	483	-	3571801 COG1316K	LytR family transcriptional regulator	*
M5005_Spy_1100c	aroK	NE	1073444	1073935	163	-	3571802 COG0703E	shikimate kinase	*
M5005_Spy_1101c	aroA1	NE	1073928	1075220	430	-	3571803 COG0128E	3-phosphoshikimate 1-carboxyvinyltransferase	*
M5005_Spy_1102c	-	NE	1075322	1076287	321	-	3571804 COG1295S	ribonuclease BN	*
M5005_Spy_1103c	map	E	1076289	1077149	286	-	3571805 COG0024J	methionine aminopeptidase	*
M5005_Spy_1104c	-	NE	1077165	1078448	427	-	3571806 COG4109K	hypothetical protein M5005_Spy_1104	*
M5005_Spy_1105c	-	NE	1078457	1078999	180	-	3571807 COG1670J	ribosomal-protein-alanine acetyltransferase	*
M5005_Spy_1106c	grab	NE	1079237	1079890	217	-	3571808 COG2723G	protein G-related alpha 2M-binding protein	
M5005_Spy_1107c	murZ	NE	1080248	1081507	419	-	3571809 COG0766M	UDP-N-acetylglucosamine 1-carboxyvinyltransferase	*
M5005_Spy_1108c	metK2	E	1081681	1082877	398	-	3571810 COG0192H	S-adenosylmethionine synthetase	*
M5005_Spy_1109c	inlA	NE	1083414	1085792	792	-	3571811 COG4886S	internalin protein	*
M5005_Spy_1110	birA	E	1085996	1086937	313	+	3571812 COG0340H	biotin--protein ligase	*
M5005_Spy_1111c	-	NE	1086912	1087220	102	-	3571813 -	hypothetical protein M5005_Spy_1111	
M5005_Spy_1112c	dnaX	E	1087210	1088880	556	-	3571814 COG2812L	DNA polymerase III subunits gamma/tau	
M5005_Spy_1113c	-	NE	1088880	1089377	165	-	3571776 COG1956T	GAF domain-containing protein	*
M5005_Spy_1114	-	NE	1089522	1090331	269	+	3571777 COG2339S	hypothetical protein M5005_Spy_1114	*
M5005_Spy_1115c	-	NE	1090384	1090647	87	-	3571778 COG3326S	hypothetical protein M5005_Spy_1115	
M5005_Spy_1116c	udk	NE	1090727	1091353	208	-	3571779 COG0572F	uridine kinase	*
M5005_Spy_1117	deaD2	NE	1091451	1092536	361	+	3571780 COG0513LKJ	ATP-dependent RNA helicase	*
M5005_Spy_1118c	-	NE	1092700	1093920	406	-	3571781 COG0726G	peptidoglycan N-acetylglucosamine deacetylase	*
M5005_Spy_1119c	gapN	E	1094015	1095442	475	-	3571782 COG1012C	dehydrogenase	
M5005_Spy_1120c	pstI	C	1095627	1097360	577	-	3571783 COG1080G	phosphoenolpyruvate-protein phosphotransferase	*
M5005_Spy_1121c	ptsH	C	1097365	1097628	87	-	3571784 COG1925G	phosphocarrier protein HPr	
M5005_Spy_1122	nrdH	NE	1098021	1098239	72	+	3571785 COG0695O	glutaredoxin	
M5005_Spy_1123	nrdE.2	NE	1098259	1100418	719	+	3571786 COG0209F	ribonucleotide-diphosphate reductase subunit alpha	*
M5005_Spy_1124	nrdF	NE	1100751	1101710	319	+	3571787 COG0208F	ribonucleotide-diphosphate reductase subunit beta	*
M5005_Spy_1125	-	NE	1101685	1102998	437	+	3571788 COG0038P	chloride channel protein	*

M5005_Spy_1129c	-	NE	1104384	1105079	231	-	3571792 COG1266R	CAAX amino protease	*
M5005_Spy_1130c	-	NE	1105098	1105859	253	-	3571793 -	hypothetical protein M5005_Spy_1130	*
M5005_Spy_1131c	-	NC	1105856	1106077	73	-	3571794 COG1476K	Cro/CI family transcriptional regulator	
M5005_Spy_1132c	alaS	E	1106432	1109050	872	-	3571795 COG0013J	alanyl-tRNA synthetase	*
M5005_Spy_1133c	prsA	NE	1109437	1110492	351	-	3571757 COG0760O	foldase PrsA	*
M5005_Spy_1134c	-	NE	1110555	1111262	235	-	3571758 COG4122R	O-methyltransferase	*
M5005_Spy_1135c	-	NE	1111328	1112524	398	-	3571759 COG2271G	oxalate/formate antiporter	*
M5005_Spy_1136c	pepB	NE	1112900	1114705	601	-	3571760 COG1164E	oligoendopeptidase F	*
M5005_Spy_1137c	-	NE	1114718	1115680	320	-	3571761 COG4469R	competence protein/transcription factor	*
M5005_Spy_1138c	-	NE	1115975	1116691	238	-	3571762 COG1187J	ribosomal small subunit pseudouridine synthase A	
M5005_Spy_1139c	nagB	NE	1116810	1117514	234	-	3571763 COG0363G	glucosamine-6-phosphate isomerase	*
M5005_Spy_1140	queA	NE	1117716	1118744	342	+	3571764 COG0809J	isomerase	*
M5005_Spy_1141	-	NE	1118751	1119971	406	+	3571765 -	hypothetical protein M5005_Spy_1141	*
M5005_Spy_1142c	-	NE	1120085	1120675	196	-	3571766 COG1686M	hypothetical protein M5005_Spy_1142	*
M5005_Spy_1143c	-	NE	1120672	1120920	82	-	3571767 -	hypothetical protein M5005_Spy_1143	*
M5005_Spy_1144c	-	NE	1120905	1121132	75	-	3571768 -	hypothetical protein M5005_Spy_1144	*
M5005_Spy_1145c	sodA	NE	1121299	1121904	201	-	3571769 COG0605P	superoxide dismutase	*
M5005_Spy_1146c	holA	E	1122001	1123041	346	-	3571770 COG1466L	DNA polymerase III subunit delta	*
M5005_Spy_1147c	comEC	NE	1123112	1125355	747	-	3571771 COG2333R	competence protein ComE	*
M5005_Spy_1148c	comE	NE	1125336	1125998	220	-	3571772 COG1555L	competence protein ComE	*
M5005_Spy_1149c	-	E	1126198	1126938	246	-	3571773 COG0204I	1-acyl-sn-glycerol-3-phosphate acyltransferase	*
M5005_Spy_1150	-	NE	1127056	1127832	258	+	3571774 COG4123R	methyltransferase	*
M5005_Spy_1151	-	NE	1127822	1128100	92	+	3571775 COG2827L	hypothetical protein M5005_Spy_1151	*
M5005_Spy_1152c	kup	NE	1128124	1129107	327	-	3571737 COG3158P	kup system potassium uptake protein, partial	*
M5005_Spy_1154c	deaD	NE	1130251	1131870	539	-	3571739 COG0513LKJ	ATP-dependent RNA helicase	*
M5005_Spy_1155c	prfC	NE	1132177	1133721	514	-	3571740 COG4108J	peptide chain release factor 3	*
M5005_Spy_1156c	-	NE	1133969	1134664	231	-	3571741 COG5522S	hypothetical protein M5005_Spy_1156	*
M5005_Spy_1157c	murF	E	1134744	1136135	463	-	3571742 COG0770M	alanyl-D-alanine ligase	*
M5005_Spy_1158c	ddl	E	1136326	1137372	348	-	3571743 COG1181M	D-alanyl-alanine synthetase A	*
M5005_Spy_1159c	recR	NE	1137473	1138069	198	-	3571744 COG0353L	recombination protein RecR	*
M5005_Spy_1160c	-	NE	1138116	1138307	63	-	3571745 -	penicillin-binding protein	
M5005_Spy_1161c	fdhC	NE	1138868	1139647	259	-	3571746 COG2116P	formate transporter	
M5005_Spy_1162c	-	NE	1139771	1140313	180	-	3571747 -	hypothetical protein M5005_Spy_1162	*
M5005_Spy_1163c	-	NE	1140474	1140995	173	-	3571748 COG1827R	biotin repressor family transcriptional regulator	*
M5005_Spy_1164c	gpmA	NC	1141102	1141797	231	-	3571749 COG0588G	phosphoglyceromutase	*
M5005_Spy_1165	pyrD	NE	1142036	1142971	311	+	3571750 COG0167F	dihydroorotate dehydrogenase 1A	*
M5005_Spy_1166c	-	NC	1143026	1143199	57	-	3571751 -	hypothetical protein M5005_Spy_1166	
M5005_Spy_1167c	-	NE	1143271	1145133	620	-	3571752 COG2217P	ATPase	*
M5005_Spy_1168c	-	NC	1145664	1145846	60	-	3571753 -	phage protein	
M5005_Spy_1169	spd3	NE	1146085	1146885	266	+	3571754 -	streptodornase	
M5005_Spy_1170	-	NE	1147096	1147590	164	+	3571755 -	hypothetical protein M5005_Spy_1170	
M5005_Spy_1171c	-	NE	1147660	1148865	401	-	3571756 COG1705NU	phage-associated cell wall hydrolase	
M5005_Spy_1172c	-	NE	1148981	1149208	75	-	3571718 COG5546S	holin	
M5005_Spy_1173c	-	NE	1149205	1149480	91	-	3571719 -	phage protein	
M5005_Spy_1174c	-	NE	1149490	1150107	205	-	3571720 -	phage protein	
M5005_Spy_1175c	-	NE	1150104	1150541	145	-	3571721 -	phage protein	
M5005_Spy_1176c	-	NE	1150553	1152421	622	-	3571722 COG4926S	phage infection protein	
M5005_Spy_1177c	-	NE	1152418	1153113	231	-	3571723 COG4722S	phage protein	
M5005_Spy_1178c	-	NE	1153110	1155467	785	-	3571724 COG5412S	phage protein	
M5005_Spy_1179c	-	NE	1155467	1155838	123	-	3571725 -	phage protein	
M5005_Spy_1180c	-	NE	1155853	1156116	87	-	3571726 -	phage protein	
M5005_Spy_1181c	-	NE	1156127	1156720	197	-	3571727 -	major tail protein	
M5005_Spy_1182c	-	NE	1156732	1157067	111	-	3571728 -	phage protein	
M5005_Spy_1183c	-	NE	1157068	1157304	78	-	3571729 -	phage protein	
M5005_Spy_1184c	-	NE	1157297	1157635	112	-	3571730 -	phage protein	
M5005_Spy_1185c	-	NE	1157595	1158017	140	-	3571731 -	phage protein	
M5005_Spy_1186c	-	NE	1158027	1158227	66	-	3571732 -	phage protein	
M5005_Spy_1187c	-	NE	1158227	1159138	303	-	3571733 -	phage structural protein	
M5005_Spy_1188c	-	NE	1159163	1159624	153	-	3571734 -	phage protein	

M5005_Spy_1189c	-	NE	1159705	1161120	471	-	3571735	COG4626R	phage terminase	
M5005_Spy_1190c	-	NE	1161230	1161496	88	-	3571736	-	phage protein	
M5005_Spy_1191c	-	NE	1161535	1161750	71	-	3571698	-	phage protein	
M5005_Spy_1192c	-	NE	1161719	1161952	77	-	3571699	-	phage protein	
M5005_Spy_1193c	-	NE	1161949	1163442	497	-	3571700	-	phage protein	
M5005_Spy_1194c	-	NE	1163435	1164703	422	-	3571701	-	phage protein	
M5005_Spy_1195c	-	NE	1164700	1165056	118	-	3571702	-	phage protein	
M5005_Spy_1196c	-	NE	1165205	1165549	114	-	3571703	COG1403V	HNH endonuclease	
M5005_Spy_1197c	-	NE	1165658	1166077	139	-	3571704	-	phage protein	
M5005_Spy_1198c	-	NE	1166345	1166980	211	-	3571705	COG0286V	phage protein	
M5005_Spy_1199c	-	NE	1166982	1167251	89	-	3571706	-	phage protein	
M5005_Spy_1200c	-	NE	1167335	1167847	170	-	3571707	-	phage protein	
M5005_Spy_1201c	-	NE	1167844	1168257	137	-	3571708	-	phage protein	
M5005_Spy_1202c	-	NC	1168363	1168530	55	-	3571709	-	phage protein	
M5005_Spy_1203c	-	NE	1168540	1169337	265	-	3571710	-	phage protein	
M5005_Spy_1204c	-	NE	1169334	1170263	309	-	3571711	COG3723L	recT protein	
M5005_Spy_1205c	-	NE	1170266	1170595	109	-	3571712	-	phage protein	
M5005_Spy_1206c	-	NE	1170651	1170857	68	-	3571713	-	phage protein	
M5005_Spy_1207c	-	NC	1170866	1171006	46	-	3571714	-	phage protein	
M5005_Spy_1208c	-	NE	1171003	1171236	77	-	3571715	-	phage protein	
M5005_Spy_1209c	-	NE	1171217	1171606	129	-	3571716	COG3935L	DNA replication protein	
M5005_Spy_1210c	-	NE	1171751	1171990	79	-	3571717	-	phage replication protein	
M5005_Spy_1211c	-	NC	1172090	1172275	61	-	3571679	-	phage protein	
M5005_Spy_1212c	xis	NE	1172277	1172588	103	-	3571680	-	excisionase	
M5005_Spy_1213c	-	NC	1172666	1172851	61	-	3571681	COG3655K	phage protein	
M5005_Spy_1214	-	NE	1173018	1173257	79	+	3571682	-	phage protein	
M5005_Spy_1215	-	NE	1173399	1174205	268	+	3571683	-	phage protein	
M5005_Spy_1216c	-	NE	1174140	1174406	88	-	3571684	-	phage protein	
M5005_Spy_1217c	-	NE	1174438	1175154	238	-	3571685	COG3645S	phage antirepressor protein	
M5005_Spy_1218c	-	NE	1175166	1175357	63	-	3571686	-	phage protein	
M5005_Spy_1219	-	E	1176511	1176858	115	+	3571687	-	Cro/CI family phage transcriptional regulator	
M5005_Spy_1220	-	NE	1176862	1177242	126	+	3571688	COG2856E	phage protein	
M5005_Spy_1221	-	NE	1177254	1177520	88	+	3571689	-	phage protein	
M5005_Spy_1222	int.2	NE	1177644	1178786	380	+	3571690	COG4974L	integrase	
M5005_Spy_1223c	-	NC	1178876	1179151	91	-	3571691	COG0776L	DNA-binding protein HU	*
M5005_Spy_1224c	-	NE	1179250	1179837	195	-	3571692	COG4698S	hypothetical protein M5005_Spy_1224	*
M5005_Spy_1225c	-	NE	1179815	1180657	280	-	3571693	COG2755E	lipase/acylhydrolase	*
M5005_Spy_1226c	-	NE	1180650	1181501	283	-	3571694	COG1307S	degV family protein	*
M5005_Spy_1227c	-	NE	1181717	1182658	313	-	3571695	-	hypothetical protein M5005_Spy_1227	*
M5005_Spy_1228c	recN	NE	1182830	1184491	553	-	3571696	COG0497L	DNA repair protein	*
M5005_Spy_1229c	argR1	NE	1184513	1184983	156	-	3571697	COG1438K	arginine repressor ArgR	*
M5005_Spy_1230c	-	NE	1184970	1185797	275	-	3571659	COG1189J	hemolysin	*
M5005_Spy_1231c	fps	NE	1185790	1186662	290	-	3571660	COG0142H	dimethylallyltransferase/geranyltranstransferase	*
M5005_Spy_1232c	xseB	NE	1186662	1186877	71	-	3571661	COG1722L	exodeoxyribonuclease VII small subunit	
M5005_Spy_1233c	xseA	NE	1186855	1188195	446	-	3571662	COG1570L	exodeoxyribonuclease VII large subunit	*
M5005_Spy_1234c	folD	C	1188348	1189202	284	-	3571663	COG0190H	dehydrogenase/ 5,10-methylene-tetrahydrofolate	*
M5005_Spy_1235c	-	NE	1189410	1191113	567	-	3571664	COG1109G	phosphoglucomutase	*
M5005_Spy_1236c	phr	NE	1191282	1192691	469	-	3571665	COG0415L	deoxyribodipyrimidine photolyase	*
M5005_Spy_1237c	artP/gtr	NE	1192840	1193574	244	-	3571666	COG1126E	arginine transport ATP-binding protein	*
M5005_Spy_1238c	artQ	NE	1193574	1194260	228	-	3571667	COG0765E	arginine transporter permease	*
M5005_Spy_1239c	-	NC	1194387	1194617	76	-	3571668	COG4703S	hypothetical protein M5005_Spy_1239	*
M5005_Spy_1240	clpE	NE	1194915	1197197	760	+	3571669	COG0542O	ATP-dependent Clp protease ATP-binding subunit	*
M5005_Spy_1241	mutT	NE	1197325	1197780	151	+	3571670	COG0494LR	7,8-dihydro-8-oxoguanine-triphosphatase	*
M5005_Spy_1242	-	NE	1197831	1198133	100	+	3571671	-	hypothetical protein M5005_Spy_1242	*
M5005_Spy_1243c	ileS	E	1198398	1201199	933	-	3571672	COG0060J	isoleucyl-tRNA synthetase	*
M5005_Spy_1244c	divIVAS	E	1201472	1202230	252	-	3571673	COG3599D	cell division initiation protein	*
M5005_Spy_1245c	-	NE	1202240	1203031	263	-	3571674	COG2302S	RNA binding protein	*
M5005_Spy_1246c	-	NC	1203031	1203285	84	-	3571675	COG0762S	hypothetical protein M5005_Spy_1246	
M5005_Spy_1247c	-	NE	1203290	1203958	222	-	3571676	COG1799S	hypothetical protein M5005_Spy_1247	*

M5005_Spy_1248c	-	**NE**	1203958	1204629	223	-	3571677 COG0325R	pyridoxal-5\'-phosphate family protein	*
M5005_Spy_1249c	ftsZ	**E**	1204632	1205951	439	-	3571678 COG0206D	cell division protein FtsZ	*
M5005_Spy_1250c	ftsA	**E**	1205975	1207339	454	-	3571640 COG0849D	cell division protein	*
M5005_Spy_1251c	divIB/ftsQ	**NC**	1207551	1208699	382	-	3571641 COG1589M	cell division protein	*
M5005_Spy_1252c	murG	**E**	1208700	1209803	367	-	3571642 COG0707M	N- acetylglucosaminyltransferase	*
M5005_Spy_1253c	murD	**E**	1209782	1211140	452	-	3571643 COG0771M	synthetase	*
M5005_Spy_1254c	-	**NE**	1211510	1211761	83	-	3571644 -	hypothetical protein M5005_Spy_1254	
M5005_Spy_1255c	typA	**NC**	1211883	1213724	613	-	3571645 COG1217T	GTP-binding protein	*
M5005_Spy_1256c	-	**NE**	1213907	1214296	129	-	3571646 COG0607P	rhodanese-related sulfurtransferase	*
M5005_Spy_1257c	glcK	**NE**	1214306	1215277	323	-	3571647 COG1940KG	glucokinase/xylose repressor	*
M5005_Spy_1258c	-	**NE**	1215282	1215485	67	-	3571648 COG4483S	hypothetical protein M5005_Spy_1258	
M5005_Spy_1259c	dpr	**NE**	1215627	1216154	175	-	3571649 COG0783P	like antioxidant protein	*
M5005_Spy_1260	-	**NE**	1216382	1217008	208	+	3571650 COG2898S	prepilin peptidase	*
M5005_Spy_1261c	-	**NE**	1217090	1218169	359	-	3571651 COG0820R	ribosomal RNA large subunit methyltransferase N	*
M5005_Spy_1262c	-	**NE**	1218173	1218793	206	-	3571652 COG4470S	transcriptional regulator	*
M5005_Spy_1263c	-	**NC**	1219088	1219207	39	-	3571653 -	hypothetical protein M5005_Spy_1263	
M5005_Spy_1264c	-	**NE**	1219241	1219942	233	-	3571654 COG1609K	ribose operon repressor, partial	*
M5005_Spy_1265c	-	**NE**	1220035	1220265	76	-	3571655 COG1609K	ribose operon repressor, partial	*
M5005_Spy_1266c	-	**NE**	1220631	1221668	345	-	3571656 COG3480T	ATP-dependent protease La	
M5005_Spy_1267c	coaD	**E**	1221655	1222146	163	-	3571657 COG0669H	phosphopantetheine adenylyltransferase	*
M5005_Spy_1268c	-	**NE**	1222136	1222675	179	-	3571658 COG0742L	methyltransferase	*
M5005_Spy_1269c	asnA	**NE**	1222798	1223790	330	-	3571620 COG2502E	asparagine synthetase AsnA	*
M5005_Spy_1270c	arcC	**NE**	1224103	1225053	316	-	3571621 COG0549E	carbamate kinase	*
M5005_Spy_1271c	-	**NE**	1225073	1226404	443	-	3571622 COG0624E	hypothetical protein M5005_Spy_1271	*
M5005_Spy_1272c	-	**NE**	1226421	1227914	497	-	3571623 COG1288S	arginine/ornithine antiporter	*
M5005_Spy_1273c	arcB	**NE**	1228084	1229097	337	-	3571624 COG0078E	ornithine carbamoyltransferase	*
M5005_Spy_1274c	-	**NE**	1229137	1229565	142	-	3571625 COG2153R	acetyltransferase	*
M5005_Spy_1275c	arcA	**NE**	1229665	1230900	411	-	3571626 COG2235E	arginine deiminase	*
M5005_Spy_1276c	-	**NE**	1231174	1231854	226	-	3571627 COG0664T	Crp/Fnr family transcriptional regulator	*
M5005_Spy_1277	ahrC.2/argR	**NE**	1231996	1232469	157	+	3571628 COG1438K	arginine repressor ArgR	*
M5005_Spy_1278c	-	**NE**	1232635	1233351	238	-	3571629 COG3382S	hypothetical protein M5005_Spy_1278	*
M5005_Spy_1279c	-	**NE**	1233365	1234444	359	-	3571630 COG2315S	hypothetical protein M5005_Spy_1279	*
M5005_Spy_1280c	yesM	**NC**	1234517	1236250	577	-	3571631 COG2972T	two-component sensor kinase	
M5005_Spy_1281c	yesN	**NE**	1236247	1236987	246	-	3571632 COG4753T	two-component response regulator	*
M5005_Spy_1282c	msrA	**NE**	1237075	1238181	368	-	3571633 COG0225O	bifunctional methionine sulfoxide reductase A/B	*
M5005_Spy_1283c	tlpA	**NE**	1238224	1238847	207	-	3571634 COG1225O	thiol:disulfide interchange protein	*
M5005_Spy_1284c	ccdA	**NE**	1238860	1239570	236	-	3571635 COG0785O	cytochrome C biogenesis protein	*
M5005_Spy_1285c	-	**NE**	1240173	1240466	97	-	3571636 COG1343L	hypothetical protein M5005_Spy_1285	
M5005_Spy_1286c	-	**NE**	1240477	1241502	341	-	3571637 COG1518L	DNA polymerase	
M5005_Spy_1287c	-	**NE**	1241499	1242173	224	-	3571638 COG1468L	hypothetical protein M5005_Spy_1287	
M5005_Spy_1288c	-	**NE**	1242175	1243023	282	-	3571639 COG3649L	hypothetical protein M5005_Spy_1288	
M5005_Spy_1289c	-	**NE**	1243028	1244923	631	-	3571602 -	hypothetical protein M5005_Spy_1289	
M5005_Spy_1290c	-	**NE**	1244923	1245651	242	-	3571603 -	hypothetical protein M5005_Spy_1290	
M5005_Spy_1291c	-	**NE**	1245784	1248186	800	-	3571604 COG1203R	ATP-dependent RNA helicase	
M5005_Spy_1292c	valS	**E**	1248346	1250751	801	-	3571605 COG0525J	valyl-tRNA synthetase	*
M5005_Spy_1293c	-	**NE**	1250995	1251558	187	-	3571606 COG0563F	hypothetical protein M5005_Spy_1293	*
M5005_Spy_1294c	-	**NC**	1251555	1251734	59	-	3571607 COG1670J	ribosomal-protein-serine acetyltransferase	
M5005_Spy_1295c	-	**NE**	1252159	1252554	131	-	3571608 -	hypothetical protein M5005_Spy_1295	*
M5005_Spy_1296c	-	**NE**	1252572	1252826	84	-	3571609 -	hypothetical protein M5005_Spy_1296	*
M5005_Spy_1297	aroA2	**NE**	1253305	1254057	250	+	3571611 COG2876E	3-deoxy-7-phosphoheptulonate synthase	*
M5005_Spy_1298	aroB	**NE**	1254113	1255186	357	+	3571612 COG0337E	3-dehydroquinate synthase	*
M5005_Spy_1299c	-	**NE**	1255455	1255613	52	-	3571613 -	hypothetical protein M5005_Spy_1299	
M5005_Spy_1300c	-	**NE**	1255621	1255926	101	-	3571614 COG1917S	hypothetical protein M5005_Spy_1300	*
M5005_Spy_1301c	-	**NE**	1255928	1256266	112	-	3571615 COG1917S	hypothetical protein M5005_Spy_1301	*
M5005_Spy_1302c	-	**NE**	1256319	1257074	251	-	3571616 COG2226H	SAM-dependent methyltransferase	*
M5005_Spy_1303c	aroE	**NE**	1257309	1258187	292	-	3571617 COG0169E	shikimate 5-dehydrogenase	*
M5005_Spy_1304c	lacZ	**NE**	1258325	1261741	1138	-	3571618 COG3250G	beta-galactosidase	
M5005_Spy_1305c	trxR	**NE**	1261761	1263245	494	-	3571619 COG4753T	two-component response regulator	*
M5005_Spy_1306c	trxS	**NE**	1263245	1264969	574	-	3571583 COG2972T	two-component sensor kinase	*

M5005_Spy_1307c	trxT	**NE**	1264959	1265564	201	-	3571584	COG5578S	hypothetical protein M5005_Spy_1307	*
M5005_Spy_1308c	-	**NE**	1265870	1267315	481	-	3571585	COG1653G	sugar-binding protein	*
M5005_Spy_1309c	-	**NE**	1267396	1268322	308	-	3571586	COG0395G	sugar transporter permease	*
M5005_Spy_1310c	-	**NE**	1268332	1269282	316	-	3571587	COG4209G	sugar transporter permease	*
M5005_Spy_1311	-	**NE**	1269478	1270356	292	+	3571588	COG1940KG	glucokinase	*
M5005_Spy_1312	-	**NC**	1270500	1270622	40	+	3571589	-	hypothetical protein M5005_Spy_1312	
M5005_Spy_1313c	-	**NE**	1270968	1272410	480	-	3571590	COG2723G	beta-glucosidase	*
M5005_Spy_1314c	hyl	**NE**	1272434	1274128	564	-	3571591	-	hyaluronoglucosaminidase	*
M5005_Spy_1315c	-	**NE**	1274179	1275219	346	-	3571592	COG1609K	GntR family transcriptional regulator	*
M5005_Spy_1316	-	**NE**	1275352	1276638	428	+	3571593	COG3538S	hypothetical protein M5005_Spy_1316	*
M5005_Spy_1317	-	**NE**	1276653	1279358	901	+	3571594	COG0383G	alpha-mannosidase	*
M5005_Spy_1318c	rocA	**NE**	1279459	1280814	451	-	3571595	COG2972T	sensory transduction protein kinase	*
M5005_Spy_1319c	-	**NE**	1281479	1282834	451	-	3571596	COG2265J	tRNA (uracil-5-)-methyltransferase	*
M5005_Spy_1320	recX	**NE**	1282949	1283725	258	+	3571597	COG2137R	recombination regulator RecX	
M5005_Spy_1321	-	**C**	1283805	1284338	177	+	3571598	COG3557J	hypothetical protein M5005_Spy_1321	*
M5005_Spy_1322c	-	**NC**	1284437	1284586	49	-	3571599	-	hypothetical protein M5005_Spy_1322	
M5005_Spy_1323	-	**NE**	1284708	1284986	92	+	3571600	-	transposase	
M5005_Spy_1324	-	**NC**	1291239	1291373	44	+	3571555	-	hypothetical protein M5005_Spy_1324	
M5005_Spy_1325c	-	**NE**	1292030	1292578	182	-	3571556	COG1544J	ribosome-associated factor Y	*
M5005_Spy_1326c	comFC	**NE**	1292658	1293323	221	-	3571557	COG1040R	competence protein ComF	*
M5005_Spy_1327c	comFA	**NE**	1293295	1294509	404	-	3571558	COG4098L	competence protein ComF	*
M5005_Spy_1328	-	**NE**	1294676	1295308	210	+	3571559	COG1739S	Xaa-Pro dipeptidase	*
M5005_Spy_1329	cysM	**NE**	1295436	1296377	313	+	3571560	COG0031E	cysteine synthase	*
M5005_Spy_1330c	-	**C**	1296395	1296772	125	-	3571561	COG1098J	hypothetical protein M5005_Spy_1330	*
M5005_Spy_1331c	-	**NE**	1296772	1298172	466	-	3571562	COG0652O	peptidyl-prolyl cis-trans isomerase	
M5005_Spy_1332c	yvqC	**NC**	1298209	1298850	213	-	3571563	COG2197TK	two-component response regulator	*
M5005_Spy_1333c	yvqE	**NE**	1298843	1299847	334	-	3571564	COG4585T	two-component sensor protein	*
M5005_Spy_1334c	yvqF	**NE**	1299844	1300536	230	-	3571565	COG4758S	transporter	*
M5005_Spy_1335c	-	**E**	1300659	1302557	632	-	3571566	COG2815S	serine/threonine protein kinase	*
M5005_Spy_1336c	pppL	**E**	1302554	1303294	246	-	3571567	COG0631T	protein phosphatase 2C	*
M5005_Spy_1337c	sunL	**NE**	1303332	1304654	440	-	3571568	COG0144J	16S rRNA m(5)C 967 methyltransferase	*
M5005_Spy_1338c	fmt	**E**	1304644	1305579	311	-	3571569	COG0223J	methionyl-tRNA formyltransferase	*
M5005_Spy_1339c	priA	**C**	1305641	1308025	794	-	3571570	COG1198L	primosome assembly protein PriA	*
M5005_Spy_1340c	-	**NE**	1308090	1308407	105	-	3571571	COG1758K	DNA-directed RNA polymerase subunit omega	*
M5005_Spy_1341c	gmk	**E**	1308423	1309058	211	-	3571572	COG0194F	guanylate kinase	*
M5005_Spy_1342c	-	**NC**	1309168	1310775	535	-	3571535	COG1418R	hypothetical protein M5005_Spy_1342	*
M5005_Spy_1343c	-	**NE**	1310905	1311801	298	-	3571536	COG0583K	LysR family transcriptional regulator	*
M5005_Spy_1344	atoB	**NE**	1312003	1313190	395	+	3571537	COG0183I	acetyl-CoA acetyltransferase	*
M5005_Spy_1345	atoD.1	**NE**	1313214	1313864	216	+	3571538	COG1788I	acetate CoA-transferase subunit alpha	*
M5005_Spy_1346	atoA	**NE**	1313866	1314525	219	+	3571539	COG2057I	acetate CoA-transferase subunit beta	
M5005_Spy_1347	-	**NE**	1314558	1315337	259	+	3571540	COG1028IQR	3-hydroxybutyrate dehydrogenase	
M5005_Spy_1348	-	**NE**	1315408	1316739	443	+	3571541	COG2610GE	D-beta-hydroxybutyrate permease	*
M5005_Spy_1349	luxS	**NE**	1316723	1317295	190	+	3571542	COG1854T	S-ribosylhomocysteinase	*
M5005_Spy_1350c	-	**NE**	1317440	1318909	489	-	3571543	-	hypothetical protein M5005_Spy_1350	*
M5005_Spy_1351c	-	**NE**	1318923	1320077	384	-	3571544	COG0116L	methyltransferase	*
M5005_Spy_1352c	-	**NC**	1320522	1320848	108	-	3571545	COG3599D	cell division initiation protein	*
M5005_Spy_1353c	-	**NE**	1320970	1321485	171	-	3571546	COG4474S	hypothetical protein M5005_Spy_1353	*
M5005_Spy_1354	recU	**NE**	1321566	1322165	199	+	3571547	COG3331R	Holliday junction-specific endonuclease	*
M5005_Spy_1355	pbp1A	**E**	1322152	1324317	721	+	3571548	COG0744M	multimodular transpeptidase-transglycosylase	*
M5005_Spy_1356c	pepC	**NE**	1324784	1326121	445	-	3571549	COG3579E	aminopeptidase	*
M5005_Spy_1357c	nadE	**E**	1326306	1327130	274	-	3571550	COG0171H	NAD synthetase	
M5005_Spy_1358c	nadE	**E**	1327132	1328586	484	-	3571551	COG1488H	nicotinate phosphoribosyltransferase	
M5005_Spy_1359c	-	**NE**	1328757	1330136	459	-	3571552	COG1113E	amino acid permease	*
M5005_Spy_1360c	-	**C**	1330305	1331222	305	-	3571553	COG0492O	thioredoxin reductase	
M5005_Spy_1361c	aapA	**E**	1331286	1331510	74	-	3571554	-	hypothetical protein M5005_Spy_1361	
M5005_Spy_1362c	-	**NE**	1331614	1332360	248	-	3571516	COG1126E	transporter	*
M5005_Spy_1363c	-	**NE**	1332357	1333160	267	-	3571517	COG0765E	amino acid ABC transporter permease	*
M5005_Spy_1364c	-	**E**	1333355	1334698	447	-	3571518	COG0513LKJ	ATP-dependent RNA helicase	*
M5005_Spy_1365c	mraY	**E**	1334856	1335866	336	-	3571519	COG0472M	transferase	*

M5005_Spy_1366c	ftsI	E	1335868	1338123	751	-	3571520 COG0768M	protein ftsI	*
M5005_Spy_1367c	ftsL	E	1338127	1338450	107	-	3571521 COG4839D	cell division protein	*
M5005_Spy_1368c	mraW	C	1338455	1339468	337	-	3571522 COG0275M	S-adenosyl-methyltransferase MraW	*
M5005_Spy_1369c	-	NC	1339492	1339599	35	-	3571523 -	hypothetical protein M5005_Spy_1369	
M5005_Spy_1370c	proA	NE	1339942	1341192	416	-	3571524 COG0014E	gamma-glutamyl phosphate reductase	
M5005_Spy_1371c	proB	NE	1341185	1342006	273	-	3571525 COG0263E	gamma-glutamyl kinase	
M5005_Spy_1372c	proB	NE	1342071	1343699	542	-	3571526 COG1269C	ABC transporter permease	*
M5005_Spy_1373c	-	NE	1343704	1344438	244	-	3571527 COG1131V	ABC transporter ATP-binding protein	*
M5005_Spy_1374c	-	NE	1344472	1344738	88	-	3571528 -	hypothetical protein M5005_Spy_1374	
M5005_Spy_1375c	tkt	NE	1344931	1346916	661	-	3571529 COG0021G	transketolase	*
M5005_Spy_1376c	tal	NE	1347134	1347778	214	-	3571530 COG0176G	translaldolase	*
M5005_Spy_1377c	-	NE	1347904	1349403	499	-	3571531 COG3711K	trans-acting positive regulator	
M5005_Spy_1378c	npx	NE	1349393	1350739	448	-	3571532 COG0446R	NADH peroxidase	
M5005_Spy_1379c	glpF	NE	1350848	1351549	233	-	3571533 COG0580G	glycerol uptake facilitator protein	*
M5005_Spy_1380c	glpO	NE	1351551	1353389	612	-	3571534 COG0578C	alpha-glycerophosphate oxidase	
M5005_Spy_1381c	glpK	NE	1353405	1354931	508	-	3571496 COG0554C	glycerol kinase	*
M5005_Spy_1382c	-	NE	1355292	1355684	130	-	3571497 COG3212S	hypothetical protein M5005_Spy_1382	*
M5005_Spy_1383c	-	NC	1355811	1356068	85	-	3571498 COG4224S	hypothetical protein M5005_Spy_1383	
M5005_Spy_1384c	glyS	E	1356222	1358261	679	-	3571499 COG0751J	glycyl-tRNA synthetase subunit beta	*
M5005_Spy_1385c	glyQ	E	1358639	1359556	305	-	3571500 COG0752J	glycyl-tRNA synthetase subunit alpha	*
M5005_Spy_1386c	-	NE	1359928	1360461	177	-	3571501 COG1755S	hypothetical protein M5005_Spy_1386	*
M5005_Spy_1387c	-	NE	1360593	1361432	279	-	3571502 COG0656R	aldo/keto reductase	
M5005_Spy_1388c	nagA	NE	1361554	1362702	382	-	3571503 COG1820G	N-acetylglucosamine-6-phosphate deacetylase	*
M5005_Spy_1389c	-	NE	1362820	1364469	549	-	3571504 COG1283P	sodium-dependent phosphate transporter	*
M5005_Spy_1390	-	NE	1364653	1365375	240	+	3571505 COG0711C	hypothetical protein M5005_Spy_1390	
M5005_Spy_1391c	-	NE	1365504	1366346	280	-	3571506 COG1307S	degV family protein	*
M5005_Spy_1392	-	NE	1366639	1367196	185	+	3571507 COG1309K	TetR family transcriptional regulator	*
M5005_Spy_1393c	-	NE	1367232	1368056	274	-	3571508 COG0561R	HAD superfamily hydrolase	*
M5005_Spy_1394c	-	NE	1368058	1368675	205	-	3571509 COG2860S	hypothetical protein M5005_Spy_1394	*
M5005_Spy_1395c	lacD.1	NE	1368872	1369849	325	-	3571510 COG3684G	tagatose 1,6-diphosphate aldolase	*
M5005_Spy_1396c	lacC1	NE	1369999	1370349	116	-	3571511 COG1105G	tagatose-6-phosphate kinase	
M5005_Spy_1397c	lacB.1	NE	1370359	1370874	171	-	3571512 COG0698G	galactose-6-phosphate isomerase subunit LacB	*
M5005_Spy_1398c	lacA.1	NE	1370889	1371314	141	-	3571513 COG0698G	galactose-6-phosphate isomerase subunit LacA	*
M5005_Spy_1399c	-	NE	1371554	1373002	482	-	3571514 COG3775G	IIC	*
M5005_Spy_1400c	-	NE	1373031	1373336	101	-	3571515 COG3414G	IIB	*
M5005_Spy_1401c	-	NE	1373329	1373802	157	-	3571477 COG1762GT	IIA	*
M5005_Spy_1402	lacR.1	NE	1374039	1374809	256	+	3571478 COG1349KG	lactose phosphotransferase system repressor	*
M5005_Spy_1403c	-	NE	1374836	1375006	56	-	3571479 -	copper chaperone	
M5005_Spy_1404c	copZ	NE	1375013	1375216	67	-	3571480 COG2608P	copper chaperone	
M5005_Spy_1405c	copA	NE	1375230	1377461	743	-	3571481 COG2217P	copper-exporting ATPase	*
M5005_Spy_1406c	copY	NE	1377461	1377895	144	-	3571482 COG3682K	copAB ATPase metal-fist type repressor	
M5005_Spy_1407	-	NE	1378067	1379053	328	+	3571483 COG0657I	esterase	
M5005_Spy_1408c	rbfA	C	1379187	1379543	118	-	3571484 COG0858J	ribosome-binding factor A	*
M5005_Spy_1409c	infB	E	1379742	1382603	953	-	3571485 COG0532J	translation initiation factor IF-2	*
M5005_Spy_1410c	-	C	1382623	1382925	100	-	3571486 COG1358J	hypothetical protein M5005_Spy_1410	*
M5005_Spy_1411c	-	C	1382918	1383214	98	-	3571487 COG2740K	hypothetical protein M5005_Spy_1411	*
M5005_Spy_1412c	nusA	E	1383230	1384387	385	-	3571488 COG0195K	transcription elongation factor NusA	*
M5005_Spy_1413c	-	NE	1384562	1385098	178	-	3571489 COG0779S	hypothetical protein M5005_Spy_1413	*
M5005_Spy_1414c	-	NC	1385343	1385522	59	-	3571490 -	phage protein	
M5005_Spy_1415c	sdaD2	NE	1385761	1386933	390	+	3571491 -	phage-encoded streptodornase	
M5005_Spy_1416c	-	NE	1387049	1388245	398	-	3571492 COG1705NU	phage-associated cell wall hydrolase	
M5005_Spy_1417c	-	NE	1388356	1388541	61	-	3571493 -	phage protein	
M5005_Spy_1418c	-	NE	1388538	1388837	99	-	3571494 -	phage protein	
M5005_Spy_1419c	-	NE	1388848	1389468	206	-	3571495 -	phage protein	
M5005_Spy_1420c	-	NC	1389471	1389632	53	-	3571457 -	phage protein	
M5005_Spy_1421c	-	NE	1389641	1391548	635	-	3571458 COG1322S	phage infection protein	
M5005_Spy_1422c	-	NE	1391559	1392194	211	-	3571459 -	phage protein	
M5005_Spy_1423c	-	NE	1392194	1393249	351	-	3571460 -	hyaluronoglucosaminidase	
M5005_Spy_1424c	-	NE	1393246	1395228	660	-	3571461 COG0466O	phage endopeptidase	

M5005_Spy_1425c	-	NE	1395238	1396080	280	-	3571462	-	phage protein	
M5005_Spy_1426c	-	NE	1396092	1400474	1460	-	3571463	COG5412S	phage protein	
M5005_Spy_1427c	-	NE	1400489	1400722	77	-	3571464	-	phage protein	
M5005_Spy_1428c	-	NE	1400797	1401252	151	-	3571465	-	phage protein	
M5005_Spy_1429c	-	NE	1401306	1401905	199	-	3571466	-	phage protein	
M5005_Spy_1430c	-	NE	1401917	1402276	119	-	3571467	-	phage protein	
M5005_Spy_1431c	-	NE	1402280	1402606	108	-	3571468	-	phage protein	
M5005_Spy_1432c	-	NE	1402621	1402899	92	-	3571469	-	phage protein	
M5005_Spy_1433c	-	NE	1402910	1403266	118	-	3571470	-	phage protein	
M5005_Spy_1434c	-	NE	1403278	1404165	295	-	3571471	-	phage protein	
M5005_Spy_1435c	-	NE	1404178	1404747	189	-	3571472	-	phage scaffold protein	
M5005_Spy_1436c	-	NE	1404903	1405169	88	-	3571473	-	phage protein	
M5005_Spy_1437c	-	NE	1405172	1405360	62	-	3571474	-	hypothetical protein M5005_Spy_1437	
M5005_Spy_1438c	-	NE	1405391	1406830	479	-	3571475	COG5585T	phage protein	
M5005_Spy_1439c	-	NE	1406796	1408328	510	-	3571476	-	portal protein	
M5005_Spy_1440c	-	NE	1408344	1409621	425	-	3571438	COG1783R	terminase large subunit	
M5005_Spy_1441c	-	NE	1409611	1410105	164	-	3571439	COG3728L	phage terminase small subunit	
M5005_Spy_1442c	-	NE	1410153	1410569	138	-	3571440	-	phage transcriptional activator	
M5005_Spy_1443c	-	NE	1410566	1410757	63	-	3571441	-	phage protein	
M5005_Spy_1444c	-	NE	1410747	1411598	283	-	3571442	COG0863L	adenine-specific methyltransferase	
M5005_Spy_1445c	-	NE	1411607	1411873	88	-	3571443	-	phage protein	
M5005_Spy_1446c	-	NC	1411870	1412037	55	-	3571444	-	phage protein	
M5005_Spy_1447c	-	NE	1412038	1413360	440	-	3571445	COG0553KL	phage-related DNA helicase	
M5005_Spy_1448c	-	NE	1413357	1413632	91	-	3571446	-	hypothetical protein M5005_Spy_1448	
M5005_Spy_1449c	-	NE	1414019	1416403	794	-	3571447	COG3378R	DNA primase	
M5005_Spy_1450c	-	NE	1416408	1418330	640	-	3571448	COG0749L	phage-encoded DNA polymerase	
M5005_Spy_1451c	-	NE	1418373	1418930	185	-	3571449	-	phage protein	
M5005_Spy_1452c	-	NE	1418941	1419339	132	-	3571450	-	phage protein	
M5005_Spy_1453c	-	NE	1419343	1420497	384	-	3571451	-	phage protein	
M5005_Spy_1454c	-	NE	1420497	1420796	99	-	3571452	-	phage protein	
M5005_Spy_1455c	-	NC	1420884	1421087	67	-	3571453	-	phage protein	
M5005_Spy_1456c	-	NC	1421084	1421236	50	-	3571454	-	phage protein	
M5005_Spy_1457c	-	NE	1421233	1421619	128	-	3571455	-	phage protein	
M5005_Spy_1458c	-	NC	1421616	1421819	67	-	3571456	-	phage protein	
M5005_Spy_1459c	-	NC	1421812	1421982	56	-	3571419	-	phage protein	
M5005_Spy_1460c	-	NC	1421979	1422254	91	-	3571420	-	phage protein	
M5005_Spy_1461c	-	NE	1422316	1422531	71	-	3571421	-	phage protein	
M5005_Spy_1462	-	NE	1422579	1422992	137	+	3571422	-	phage protein	
M5005_Spy_1463c	-	NC	1422973	1423128	51	-	3571423	-	phage protein	
M5005_Spy_1464	-	E	1423403	1423804	133	+	3571424	COG1396K	Cro/CI family phage transcriptional regulator	
M5005_Spy_1465	-	NE	1423818	1424201	127	+	3571425	-	phage protein	
M5005_Spy_1466	-	NE	1424212	1424763	183	+	3571426	-	phage protein	
M5005_Spy_1467	int.3	NE	1424880	1425959	359	+	3571427	COG4974L	integrase	
M5005_Spy_1468c	trmB	NE	1426157	1426792	211	-	3571429	COG0220R	tRNA (guanine-N(7)-)-methyltransferase	*
M5005_Spy_1469c	-	E	1426792	1427583	263	-	3571430	COG0510M	phosphotransferase	*
M5005_Spy_1470c	-	NE	1427647	1428681	344	-	3571431	COG4473U	protein ecsB	*
M5005_Spy_1471c	-	NE	1428684	1429409	241	-	3571432	COG1131V	ABC transporter ATP-binding protein	*
M5005_Spy_1472	hit	NE	1429481	1429900	139	+	3571433	COG0537FGR	bis(5\'-nucleosyl)-tetraphosphatase (asymmetrical)	*
M5005_Spy_1473	-	NE	1429897	1430253	118	+	3571434	-	hypothetical protein M5005_Spy_1473	*
M5005_Spy_1474c	lytR	NE	1430364	1431638	424	-	3571435	COG1316K	LytR family transcriptional regulator	*
M5005_Spy_1475c	-	NE	1431647	1432171	174	-	3571436	COG0456R	acetyltransferase	*
M5005_Spy_1476c	-	NC	1432146	1432607	153	-	3571437	COG0802R	ATP/GTP hydrolase	
M5005_Spy_1477c	-	NE	1432761	1434221	486	-	3571400	COG2252R	guanine-hypoxanthine permease	*
M5005_Spy_1478	-	NE	1434485	1435297	270	+	3571401	COG0561R	HAD superfamily hydrolase	*
M5005_Spy_1479	manL	NE	1435649	1436641	330	+	3571402	COG3444G	IIAB	*
M5005_Spy_1480	manM	NE	1436730	1437539	269	+	3571403	COG3715G	PTS system mannose-specific transporter subunit IIC	*
M5005_Spy_1481	manN	NE	1437556	1438467	303	+	3571404	COG3716G	PTS system mannose-specific transporter subunit IID	*
M5005_Spy_1482	manO	NE	1438581	1438940	119	+	3571405	COG4687S	hypothetical protein M5005_Spy_1482	*
M5005_Spy_1483	serS	E	1439332	1440609	425	+	3571406	COG0172J	seryl-tRNA synthetase	*

M5005_Spy_1484c	accD	E	1440831	1441601	256	-	3571407 COG0825I	acetyl-CoA carboxylase subunit alpha	*
M5005_Spy_1485c	accA	E	1441598	1442464	288	-	3571408 COG0777I	acetyl-CoA carboxylase subunit beta	*
M5005_Spy_1486c	accC	E	1442473	1443837	454	-	3571409 COG0439I	acetyl-CoA carboxylase biotin carboxylase subunit	*
M5005_Spy_1487c	fabZ	C	1443869	1444291	140	-	3571410 COG0764I	(3R)-hydroxymyristoyl-ACP dehydratase	*
M5005_Spy_1488c	accB	E	1444288	1444788	166	-	3571411 COG0511I	protein subunit	*
M5005_Spy_1489c	fabF	E	1444790	1446022	410	-	3571412 COG0304IQ	3-oxoacyl-ACP synthase	*
M5005_Spy_1490c	fabG	E	1446037	1446771	244	-	3571413 COG1028IQR	3-ketoacyl-ACP reductase	*
M5005_Spy_1491c	fabD	E	1446761	1447699	312	-	3571414 COG0331I	ACP S-malonyltransferase	*
M5005_Spy_1492c	fabK	C	1447724	1448695	323	-	3571415 COG2070R	enoyl-ACP reductase	*
M5005_Spy_1493c	acpP	E	1448897	1449121	74	-	3571416 COG0236IQ	acyl carrier protein	
M5005_Spy_1494c	fabH	E	1449182	1450156	324	-	3571417 COG0332I	3-oxoacyl-ACP synthase	*
M5005_Spy_1495c	-	NC	1450157	1450591	144	-	3571418 COG1846K	MarR family transcriptional regulator	*
M5005_Spy_1496c	phaB	E	1450668	1451459	263	-	3571380 COG1024I	enoyl-CoA hydratase	*
M5005_Spy_1497c	dnaJ	E	1451679	1452836	385	-	3571381 COG0484O	molecular chaperone DnaJ	*
M5005_Spy_1498c	dnaK	E	1453117	1454943	608	-	3571382 COG0443O	molecular chaperone DnaK	*
M5005_Spy_1499c	grpE	E	1455124	1455657	177	-	3571383 COG0576O	heat shock protein GrpE	*
M5005_Spy_1500c	hrcA	C	1455699	1456733	344	-	3571384 COG1420K	heat-inducible transcription repressor	*
M5005_Spy_1501c	-	NE	1456868	1457437	189	-	3571385 COG1705NU	N-acetylmuramoyl-L-alanine amidase	*
M5005_Spy_1502c	-	NE	1457434	1458177	247	-	3571386 COG1876M	D-alanyl-D-alanine carboxypeptidase	*
M5005_Spy_1503c	-	NE	1458167	1458874	235	-	3571387 COG0406G	phosphoglycerate mutase	*
M5005_Spy_1504c	-	NE	1459265	1459513	82	-	3571388 COG2261S	hypothetical protein M5005_Spy_1504	
M5005_Spy_1505c	-	NE	1459723	1459938	71	-	3571389 -	hypothetical protein M5005_Spy_1505	
M5005_Spy_1506c	gatB	E	1459935	1461374	479	-	3571390 COG0064J	aspartyl/glutamyl-tRNA amidotransferase subunit B	*
M5005_Spy_1507c	gatA	E	1461374	1462840	488	-	3571391 COG0154J	aspartyl/glutamyl-tRNA amidotransferase subunit A	*
M5005_Spy_1508c	gatC	E	1462840	1463142	100	-	3571392 COG0721J	aspartyl/glutamyl-tRNA amidotransferase subunit C	*
M5005_Spy_1509c	-	NE	1463374	1463583	69	-	3571393 COG0574G	pyruvate, phosphate dikinase, partial	
M5005_Spy_1510c	-	NC	1463555	1463671	38	-	3571394 -	pyruvate, phosphate dikinase	
M5005_Spy_1511c	-	NE	1464245	1464691	148	-	3571395 COG1335Q	pyrazinamidase/nicotinamidase	*
M5005_Spy_1512c	codY	C	1464838	1465620	260	-	3571396 COG4465K	transcriptional repressor CodY	
M5005_Spy_1513c	-	NE	1465838	1467052	404	-	3571397 COG0436E	aminotransferase	*
M5005_Spy_1514	-	NE	1467283	1467735	150	+	3571398 COG0589T	universal stress protein	*
M5005_Spy_1515c	-	NE	1467858	1469135	425	-	3571399 COG4696S	HAD superfamily hydrolase	*
M5005_Spy_1516	asnB	NE	1469318	1470283	321	+	3571361 COG0252EJ	L-asparaginase	*
M5005_Spy_1517c	-	NE	1470632	1471336	234	-	3571362 -	lantibiotic transport permease	*
M5005_Spy_1518c	-	NE	1471349	1472251	300	-	3571363 COG1131V	transporter	*
M5005_Spy_1519c	recG	NE	1472544	1474559	671	-	3571364 COG1200LK	ATP-dependent DNA helicase RecG	*
M5005_Spy_1520	-	NE	1474652	1474852	66	+	3571365 -	hypothetical protein M5005_Spy_1520	
M5005_Spy_1521c	-	NE	1474934	1476388	484	-	3571366 COG1122P	cobalt ABC transporter ATP-binding protein	*
M5005_Spy_1522c	-	NE	1476325	1477005	226	-	3571367 COG0619P	cobalt transporter cbiQ	*
M5005_Spy_1523c	-	NE	1477002	1477595	197	-	3571368 -	permease	*
M5005_Spy_1524c	cycC	NE	1477592	1479262	556	-	3571369 COG1132V	ABC transporter ATP-binding protein	*
M5005_Spy_1525c	cycD	NE	1479255	1481018	587	-	3571370 COG4988CO	ABC transporter ATP-binding protein	*
M5005_Spy_1526c	fhuC	NE	1481015	1481851	278	-	3571371 COG1120PH	ferrichrome ABC transporter ATP-binding protein	*
M5005_Spy_1527c	fhuB2	NE	1481848	1482870	340	-	3571372 COG0609P	ferrichrome transporter permease	*
M5005_Spy_1528c	fhuD2	NE	1482872	1483756	294	-	3571373 COG0614P	ferrichrome-binding protein	*
M5005_Spy_1529c	shp	NE	1483740	1484615	291	-	3571374 -	heme binding protein	*
M5005_Spy_1530c	-	NE	1484812	1488639	1275	-	3571375 COG4886S	Fe3+-siderophore transporter	*
M5005_Spy_1531c	isp2	NE	1489128	1490639	503	-	3571376 COG3942R	hypothetical protein M5005_Spy_1531	*
M5005_Spy_1532c	alr	NC	1490726	1491826	366	-	3571377 COG0787M	alanine racemase	*
M5005_Spy_1533c	acpS	NC	1491823	1492179	118	-	3571378 COG0736I	4\'-phosphopantetheinyl transferase	*
M5005_Spy_1534c	secA	E	1492295	1494814	839	-	3571379 COG0653U	preprotein translocase subunit SecA	*
M5005_Spy_1535c	-	NC	1494892	1494996	34	-	3571341 -	hypothetical protein M5005_Spy_1535	
M5005_Spy_1536c	-	NE	1494980	1495495	171	-	3571342 -	transposase	
M5005_Spy_1537c	-	NE	1495398	1496102	234	-	3571343 COG5433L	transposase, partial	
M5005_Spy_1538c	pmi	NE	1496264	1497217	317	-	3571344 COG1482G	mannose-6-phosphate isomerase	*
M5005_Spy_1539c	scrK	NE	1497312	1498268	318	-	3571345 COG1940KG	fructokinase	*
M5005_Spy_1540c	endoS	NE	1498460	1501447	995	-	3571346 -	endo-beta-N-acetylglucosaminidase F2	
M5005_Spy_1541c	-	NC	1501471	1501635	54	-	3571347 -	hypothetical protein M5005_Spy_1541	
M5005_Spy_1542c	scrA	NE	1501678	1503561	627	-	3571348 COG1263G	IIABC	*

M5005_Spy_1543	scrB	NE	1503803	1505242	479	+	3571349	COG1621G	sucrose-6-phosphate hydrolase	*
M5005_Spy_1544	scrR	NE	1505247	1506212	321	+	3571350	COG1609K	sucrose operon repressor	*
M5005_Spy_1545c	nusB	C	1506353	1506805	150	-	3571351	COG0781K	transcription antitermination protein NusB	*
M5005_Spy_1546c	-	NC	1506798	1507187	129	-	3571352	COG1302S	Gls24 family general stress protein	*
M5005_Spy_1547c	efp	NE	1507233	1507790	185	-	3571353	COG0231J	elongation factor P	*
M5005_Spy_1548c	comEB	NE	1507886	1508347	153	-	3571354	COG2131F	competence protein ComE	*
M5005_Spy_1549c	pepP	NE	1508382	1509455	357	-	3571355	COG0006E	Xaa-Pro dipeptidase	*
M5005_Spy_1550c	uvrA	NE	1509570	1512428	952	-	3571356	COG0178L	excinuclease ABC subunit A	*
M5005_Spy_1551	corA	E	1512601	1513545	314	+	3571357	COG0598P	magnesium and cobalt transporter	*
M5005_Spy_1552	-	NE	1513678	1514334	218	+	3571358	COG4858S	hypothetical protein M5005_Spy_1552	*
M5005_Spy_1553c	rpsR	NC	1514467	1514706	79	-	3571359	COG0238J	30S ribosomal protein S18	
M5005_Spy_1554c	ssb3	NC	1514871	1515362	163	-	3571360	COG0629L	single-stranded DNA-binding protein	*
M5005_Spy_1555c	rpsF	NC	1515384	1515674	96	-	3571322	COG0360J	30S ribosomal protein S6	*
M5005_Spy_1556c	-	NE	1515847	1516140	97	-	3571323	-	hypothetical protein M5005_Spy_1556	*
M5005_Spy_1557	mutY	NE	1516461	1517462	333	+	3571324	COG1194L	A/G-specific adenine glycosylase	*
M5005_Spy_1558	-	NE	1517538	1518125	195	+	3571325	COG1476K	transcriptional regulator	
M5005_Spy_1559c	trx	NE	1518177	1518491	104	-	3571326	COG3118O	thioredoxin	*
M5005_Spy_1560c	-	NE	1518572	1519075	167	-	3571327	COG0671I	phosphatidylglycerophosphatase B	*
M5005_Spy_1561c	mutS2	NE	1519076	1521415	779	-	3571328	COG1193L	DNA mismatch repair protein	*
M5005_Spy_1562c	-	NE	1521564	1522109	181	-	3571329	COG1286R	colicin V production protein	*
M5005_Spy_1563c	-	NE	1522112	1522420	102	-	3571330	-	hypothetical protein M5005_Spy_1563	*
M5005_Spy_1564	-	C	1522577	1523479	300	+	3571331	COG1039L	ribonuclease HIII	*
M5005_Spy_1565	spi	E	1523490	1524083	197	+	3571332	COG0681U	signal peptidase I	*
M5005_Spy_1566	recD	NE	1524141	1526594	817	+	3571333	COG0507L	exodeoxyribonuclease V subunit alpha	*
M5005_Spy_1567	-	NE	1526685	1527167	160	+	3571334	-	hypothetical protein M5005_Spy_1567	*
M5005_Spy_1568c	dinP	NE	1527260	1528354	364	-	3571335	COG0389L	DNA polymerase IV	*
M5005_Spy_1569	pfl	NE	1528563	1530890	775	+	3571336	COG1882C	formate acetyltransferase	*
M5005_Spy_1570c	-	NE	1531068	1532018	316	-	3571337	COG1680V	penicillin-binding protein	*
M5005_Spy_1571c	cppA	NE	1532003	1532755	250	-	3571338	COG2514R	c3-degrading proteinase	*
M5005_Spy_1572	-	NE	1533053	1533949	298	+	3571339	COG0730R	hypothetical protein M5005_Spy_1572	*
M5005_Spy_1573c	glpF.2	C	1534285	1535133	282	-	3571340	COG0580G	aquaporin	*
M5005_Spy_1574c	-	NC	1535303	1535449	48	-	3571302	-	universal stress protein	
M5005_Spy_1575c	norA	NE	1535635	1536831	398	-	3571303	COG2814G	quinolone resistance protein	*
M5005_Spy_1576	srv	NE	1536937	1537656	239	+	3571304	COG0664T	Crp/Fnr family transcriptional regulator	*
M5005_Spy_1577	pepXP	NE	1537678	1539960	760	+	3571305	COG2936R	x-prolyl-dipeptidyl aminopeptidase	*
M5005_Spy_1578c	-	NE	1540040	1540261	73	-	3571306	COG1476K	Cro/CI family transcriptional regulator	
M5005_Spy_1579	-	NE	1540431	1540805	124	+	3571307	COG1476K	transcriptional regulator	*
M5005_Spy_1580	-	NE	1540998	1541297	99	+	3571308	COG0718S	hypothetical protein M5005_Spy_1580	*
M5005_Spy_1581c	-	NE	1541354	1542091	245	-	3571309	COG0789K	MerR family transcriptional regulator	*
M5005_Spy_1582c	dnaQ	NE	1542240	1542827	195	-	3571310	COG0847L	DNA polymerase III subunit epsilon	*
M5005_Spy_1583c	-	NE	1542876	1543406	176	-	3571311	-	hypothetical protein M5005_Spy_1583	*
M5005_Spy_1584	-	NE	1543612	1544781	389	+	3571312	COG2081R	NAD(FAD)-utilizing dehydrogenase	*
M5005_Spy_1585c	deoC	NE	1544876	1545547	223	-	3571313	COG0274F	deoxyribose-phosphate aldolase	*
M5005_Spy_1586c	nupC	NE	1545577	1546779	400	-	3571314	COG1972F	nucleoside permease	*
M5005_Spy_1587c	udp	NE	1546800	1547579	259	-	3571315	COG2820F	uridine phosphorylase	*
M5005_Spy_1588c	-	NC	1547644	1547745	33	-	3571316	-	hypothetical protein M5005_Spy_1588	
M5005_Spy_1589	crgR	NE	1547819	1548562	247	+	3571317	COG2188K	GntR family transcriptional regulator	*
M5005_Spy_1590	rpsN	NE	1548798	1549067	89	+	3571318	COG0199J	30S ribosomal protein S14	*
M5005_Spy_1591c	gcp	E	1549238	1550266	342	-	3571319	COG0533O	DNA-binding/iron metalloprotein/AP endonuclease	*
M5005_Spy_1592c	-	NE	1550256	1550711	151	-	3571320	COG0456R	ribosomal-protein-alanine acetyltransferase	*
M5005_Spy_1593c	-	C	1550683	1551381	232	-	3571321	COG1214O	glycoprotease	*
M5005_Spy_1594	-	E	1551666	1551896	76	+	3571284	COG5503S	hypothetical protein M5005_Spy_1594	
M5005_Spy_1595	-	E	1551898	1553580	560	+	3571285	COG0595R	Zn-dependent hydrolase	*
M5005_Spy_1596c	glnA	NE	1553807	1555153	448	-	3571286	COG0174E	glutamine synthetase	*
M5005_Spy_1597c	glnR	NE	1555191	1555562	123	-	3571287	COG0789K	MerR family transcriptional regulator	*
M5005_Spy_1598c	-	NE	1555629	1556180	183	-	3571288	COG4129S	hypothetical protein M5005_Spy_1598	*
M5005_Spy_1599c	pgk	E	1556443	1557639	398	-	3571289	COG0126G	phosphoglycerate kinase	*
M5005_Spy_1600c	lppC	NE	1557832	1558686	284	-	3571290	COG2503R	acid phosphatase	*
M5005_Spy_1601c	-	NE	1558916	1559806	296	-	3571291	COG0330O	membrane protease	

M5005_Spy_1602c	-	**E**	1560043	1561707	554	-	3571292	COG1461R	kinase	*
M5005_Spy_1603c	asp	**NE**	1561707	1562072	121	-	3571293	COG1302S	alkaline-shock protein	*
M5005_Spy_1604c	-	**NC**	1562097	1562234	45	-	3571294	-	hypothetical protein M5005_Spy_1604	
M5005_Spy_1605c	-	**NE**	1562239	1563372	377	-	3571295	COG5433L	transposase	
M5005_Spy_1606c	rpmB	**NE**	1563550	1563738	62	-	3571296	COG0227J	50S ribosomal protein L28	
M5005_Spy_1607c	fba	**E**	1564120	1565001	293	-	3571297	COG0191G	fructose-bisphosphate aldolase	*
M5005_Spy_1608c	-	**NE**	1565347	1566273	308	-	3571299	COG1073R	alpha/beta hydrolase	*
M5005_Spy_1610c	pyrG	**NE**	1567892	1568044	50	-	3571301	COG0504F	CTP synthase, partial	*
M5005_Spy_1611c	rpoE	**NE**	1568301	1568876	191	-	3571270	COG3343K	DNA-directed RNA polymerase subunit delta	*
M5005_Spy_1612c	tig/ropA	**NE**	1569093	1570376	427	-	3571271	COG0544O	trigger factor	*
M5005_Spy_1613	-	**NE**	1570697	1571542	281	+	3571272	COG0668M	mechanosensitive ion channel MscS	*
M5005_Spy_1614c	-	**NE**	1571607	1572167	186	-	3571273	COG4475S	hypothetical protein M5005_Spy_1614	*
M5005_Spy_1615c	-	**NE**	1572181	1572651	156	-	3571274	COG4720S	hypothetical protein M5005_Spy_1615	*
M5005_Spy_1616c	thiD	**NE**	1572641	1573405	254	-	3571275	COG0351H	phosphomethylpyrimidine kinase	*
M5005_Spy_1617c	truA	**NE**	1573395	1574144	249	-	3571276	COG0101J	tRNA pseudouridine synthase A	*
M5005_Spy_1618c	comX.2	**NE**	1574329	1574880	183	-	3571277	-	competence-specific sigma factor	
M5005_Spy_1619	-	**NC**	1580302	1580436	44	+	3571251	-	hypothetical protein M5005_Spy_1619	
M5005_Spy_1620c	-	**NE**	1580598	1581740	380	-	3571252	COG1929G	glycerate kinase	*
M5005_Spy_1621	hsdR	**NE**	1582022	1585000	992	+	3571253	COG0610V	subunit	*
M5005_Spy_1622	hsdS	**NE**	1585013	1586212	399	+	3571254	COG0732V	subunit	
M5005_Spy_1623	hsdM	**NE**	1586225	1587805	526	+	3571255	COG0286V	subunit	*
M5005_Spy_1624c	-	**NE**	1588015	1588206	63	-	3571256	-	hypothetical protein M5005_Spy_1624	
M5005_Spy_1625c	salR	**NE**	1588358	1588963	201	-	3571257	COG2197TK	transcriptional regulatory protein	*
M5005_Spy_1626c	salK	**NE**	1588944	1590506	520	-	3571258	COG4585T	sensory transduction protein kinase	
M5005_Spy_1627c	salY	**NE**	1590546	1592453	635	-	3571259	COG3127Q	ABC transporter permease	
M5005_Spy_1628c	salX	**NE**	1592455	1593192	245	-	3571260	COG1136V	ABC transporter ATP-binding protein	
M5005_Spy_1629c	salX/salT	**NE**	1593189	1593650	153	-	3571261	COG2274V	lantibiotic transport ATP-binding protein	
M5005_Spy_1630c	salB	**NE**	1593724	1595349	541	-	3571262	COG4403V	serine (threonine) dehydratase	
M5005_Spy_1631c	salA	**NC**	1595432	1595578	48	-	3571263	-	lantibiotic salivaricin A	
M5005_Spy_1632c	lacG	**NE**	1596090	1597496	468	-	3571264	COG2723G	6-phospho-beta-galactosidase	*
M5005_Spy_1633c	lacE	**NE**	1597584	1599281	565	-	3571265	COG1455G	PTS system lactose-specific transporter subunit IIBC	
M5005_Spy_1634c	lacF	**NE**	1599281	1599598	105	-	3571266	COG1447G	PTS system lactose-specific transporter subunit IIA	*
M5005_Spy_1635c	lacD.2	**NE**	1599622	1600605	327	-	3571267	COG3684G	tagatose 1,6-diphosphate aldolase	*
M5005_Spy_1636c	lacC.2	**NE**	1600609	1601538	309	-	3571268	COG1105G	tagatose-6-phosphate kinase	*
M5005_Spy_1637c	lacB.2	**NE**	1601586	1602101	171	-	3571269	COG0698G	galactose-6-phosphate isomerase subunit LacB	*
M5005_Spy_1638c	lacA.2	**NE**	1602136	1602564	142	-	3571231	COG0698G	galactose-6-phosphate isomerase subunit LacA	*
M5005_Spy_1639	lacR.2	**NE**	1603011	1603784	257	+	3571232	COG1349KG	lactose phosphotransferase system repressor	*
M5005_Spy_1640	-	**NE**	1604445	1604732	95	+	3571233	-	DNA-damage-inducible protein J	
M5005_Spy_1641	-	**NE**	1604722	1605057	111	+	3571234	-	hypothetical protein M5005_Spy_1641	*
M5005_Spy_1642	-	**NE**	1605209	1605391	60	+	3571235	-	DNA integration/recombination/invertion protein	
M5005_Spy_1643	-	**NC**	1605380	1605565	61	-	3571237	-	DNA integration/recombination/invertion protein	
M5005_Spy_1644c	-	**NC**	1605479	1605679	66	+	3571236	-	hypothetical protein M5005_Spy_1644	
M5005_Spy_1645	-	**NC**	1606013	1606162	49	+	3571238	-	DNA integration/recombination/invertion protein	
M5005_Spy_1646c	rpsI	**NC**	1606282	1606674	130	-	3571239	COG0103J	30S ribosomal protein S9	*
M5005_Spy_1647c	rplM	**NC**	1606695	1607141	148	-	3571240	COG0102J	50S ribosomal protein L13	*
M5005_Spy_1648c	-	**NC**	1607359	1607565	68	-	3571241	COG1476K	Cro/CI family transcriptional regulator	
M5005_Spy_1649c	-	**NE**	1607562	1608068	168	-	3571242	-	hypothetical protein M5005_Spy_1649	*
M5005_Spy_1650c	-	**NE**	1608204	1609064	286	-	3571243	COG1307S	degV family protein	*
M5005_Spy_1651c	-	**NE**	1609161	1609679	172	-	3571244	COG3688R	hypothetical protein M5005_Spy_1651	*
M5005_Spy_1652c	-	**NE**	1609683	1610429	248	-	3571245	COG0566J	23S rRNA methyltransferase	*
M5005_Spy_1653c	-	**NE**	1610476	1611273	265	-	3571246	-	hypothetical protein M5005_Spy_1653	*
M5005_Spy_1654c	-	**NE**	1611469	1611882	137	-	3571247	COG1939S	hypothetical protein M5005_Spy_1654	*
M5005_Spy_1655c	cysS	**E**	1611875	1613218	447	-	3571248	COG0215J	cysteinyl-tRNA synthetase	*
M5005_Spy_1656c	-	**NE**	1613246	1613476	76	-	3571249	-	hypothetical protein M5005_Spy_1656	
M5005_Spy_1657c	-	**NE**	1613489	1613761	90	-	3571250	-	hypothetical protein M5005_Spy_1657	
M5005_Spy_1658c	cysE	**NC**	1613955	1614536	193	-	3571212	COG1045E	serine acetyltransferase	*
M5005_Spy_1659c	-	**NE**	1614545	1615297	250	-	3571213	-	hypothetical protein M5005_Spy_1659	*
M5005_Spy_1660c	pnp	**NE**	1615290	1617422	710	-	3571214	COG1185J	polynucleotide phosphorylase	*
M5005_Spy_1661c	-	**NE**	1617703	1618431	242	-	3571215	COG0176G	translaldolase	*

M5005_Spy_1662c	ulaA	NE	1618587	1619822	411	-	3571216 COG3037S	IIC	*
M5005_Spy_1663c	-	NE	1619849	1620133	94	-	3571217 COG3414G	PTS system transporter subunit IIB	*
M5005_Spy_1664c	-	NE	1620126	1622186	686	-	3571218 COG3711K	component	*
M5005_Spy_1665c	-	NC	1622411	1622554	47	-	3571219 -	hypothetical protein M5005_Spy_1665	
M5005_Spy_1666c	rpsO	NC	1622538	1622807	89	-	3571220 COG0184J	30S ribosomal protein S15	
M5005_Spy_1667c	-	NC	1622965	1623120	51	-	3571221 -	hypothetical protein M5005_Spy_1667	
M5005_Spy_1668	-	NE	1623246	1623515	89	+	3571222 -	transcriptional regulator	
M5005_Spy_1669	def	E	1623616	1624230	204	+	3571223 COG0242J	peptide deformylase	*
M5005_Spy_1670c	-	NE	1624264	1624806	180	-	3571224 COG0431R	oxidoreductase	
M5005_Spy_1671c	-	NE	1624937	1625365	142	-	3571225 COG1846K	MarR family transcriptional regulator	*
M5005_Spy_1672c	polC	E	1625475	1629872	1465	-	3571226 COG2176L	DNA polymerase III PolC	*
M5005_Spy_1673c	proS	E	1630127	1631983	618	-	3571227 COG0442J	prolyl-tRNA synthetase	*
M5005_Spy_1674c	eep	NE	1632181	1633440	419	-	3571228 COG0750M	pheromone-processing membrane metalloprotease	*
M5005_Spy_1675c	cdsA	E	1633513	1634307	264	-	3571229 COG0575I	phosphatidate cytidylyltransferase	*
M5005_Spy_1676c	uppS	E	1634320	1635069	249	-	3571230 COG0020I	undecaprenyl pyrophosphate synthase	*
M5005_Spy_1677c	yajC	NE	1635288	1635653	121	-	3571192 COG1862U	preprotein translocase subunit YajC	*
M5005_Spy_1678c	-	NE	1635769	1636116	115	-	3571193 COG0526OC	thioredoxin	*
M5005_Spy_1680c	pulA	NE	1638902	1639783	293	-	3571195 -	pullulanase, partial	*
M5005_Spy_1681c	dexB	NE	1639954	1641567	537	-	3571196 COG0366G	glucan 1,6-alpha-glucosidase	*
M5005_Spy_1682c	msmK	NE	1641696	1642829	377	-	3571197 COG3839G	multiple sugar transport ATP-binding protein	*
M5005_Spy_1683c	lrp	NE	1643127	1643975	282	-	3571198 COG2508TQ	hypothetical protein M5005_Spy_1683	*
M5005_Spy_1684	ska	NE	1644335	1645657	440	+	3571199 -	streptokinase	
M5005_Spy_1685c	dtd	NE	1645755	1646198	147	-	3571200 COG1490J	D-tyrosyl-tRNA(Tyr) deacylase	*
M5005_Spy_1686c	relA	E	1646213	1648432	739	-	3571201 COG0317TK	GTP pyrophosphokinase	*
M5005_Spy_1687c	sclA	NE	1648684	1648827	47	-	3571202 -	hypothetical protein M5005_Spy_1687	
M5005_Spy_1690	nrdI2	NE	1650113	1650595	160	+	3571205 COG1780F	flavoprotein NrdI	
M5005_Spy_1691c	-	NE	1650989	1651807	272	-	3571206 COG3568R	exodeoxyribonuclease III	*
M5005_Spy_1693c	-	NE	1653782	1654075	97	-	3571208 COG1263G	IIABC	*
M5005_Spy_1694c	-	NE	1654433	1655182	249	-	3571209 COG1385S	16S ribosomal RNA methyltransferase RsmE	*
M5005_Spy_1695c	prmA	NE	1655182	1656135	317	-	3571210 COG2264J	50S ribosomal protein L11 methyltransferase	*
M5005_Spy_1696c	-	NE	1656206	1656676	156	-	3571211 -	hypothetical protein M5005_Spy_1696	*
M5005_Spy_1697	papB	NE	1656876	1658633	585	+	3571174 COG0147EH	amino-4-deoxychorismate lyase	*
M5005_Spy_1698	trpG	NC	1658666	1659232	188	+	3571175 COG0512EH	anthranilate synthase component II	*
M5005_Spy_1699	-	NE	1659265	1660533	422	+	3571176 COG2256L	recombination factor protein RarA	*
M5005_Spy_1700	pai1	NE	1661030	1661470	146	+	3571178 COG3153R	acetyltransferase	
M5005_Spy_1701	flaR	NE	1661525	1662031	168	+	3571179 COG0563F	topology modulation protein	
M5005_Spy_1702	smeZ	NE	1662280	1662981	233	+	3571180 -	mitogenic exotoxin Z	
M5005_Spy_1703c	-	NE	1663265	1663531	88	-	3571181 -	hypothetical protein M5005_Spy_1703	
M5005_Spy_1704	dppA	NE	1663710	1665338	542	+	3571182 COG0747E	dipeptide-binding protein	*
M5005_Spy_1705	dppB	NE	1665451	1666428	325	+	3571183 COG0601EP	dipeptide transporter permease	*
M5005_Spy_1706	dppC	NE	1666425	1667246	273	+	3571184 COG1173EP	dipeptide transporter permease	*
M5005_Spy_1707	dppD	NE	1667258	1668061	267	+	3571185 COG0444EP	dipeptide transport ATP-binding protein	*
M5005_Spy_1708	dppE	NE	1668045	1668671	208	+	3571186 COG1124EP	dipeptide transport ATP-binding protein	*
M5005_Spy_1709c	-	NE	1668752	1668952	66	-	3571187 -	hypothetical protein M5005_Spy_1709	*
M5005_Spy_1710c	-	NE	1669121	1671598	825	-	3571188 -	histidine triad protein	*
M5005_Spy_1711c	lmb	NE	1671611	1672531	306	-	3571189 COG0803P	laminin binding protein	*
M5005_Spy_1712c	-	NE	1672709	1673842	377	-	3571190 COG5433L	transposase	
M5005_Spy_1713c	-	NC	1673896	1674048	50	-	3571191 -	hypothetical protein M5005_Spy_1713	
M5005_Spy_1714c	-	NE	1674093	1675160	355	-	3571154 COG5373S	cell surface protein	
M5005_Spy_1715c	scpA	NE	1675257	1678751	1164	-	3571155 COG1404O	C5A peptidase	*
M5005_Spy_1716	-	NE	1679185	1680390	401	+	3571156 COG3666L	transposase	
M5005_Spy_1717	-	NC	1680369	1680551	60	+	3571157 -	transposase, partial	
M5005_Spy_1718c	sic1.01	NE	1680969	1681910	313	-	3571158 -	inhibitor of complement protein	
M5005_Spy_1719c	emm1.0	NE	1682099	1683553	484	-	3571159 COG1196D	M protein	
M5005_Spy_1720c	mga	NE	1683738	1685327	529	-	3571160 -	trans-acting positive regulator	
M5005_Spy_1721	-	NC	1685712	1685897	61	+	3571161 -	hypothetical protein M5005_Spy_1721	
M5005_Spy_1722c	-	NE	1686003	1686254	83	-	3571162 -	hypothetical protein M5005_Spy_1722	
M5005_Spy_1723c	isp	NE	1686333	1687934	533	-	3571163 COG3942R	hypothetical protein M5005_Spy_1723	*
M5005_Spy_1724c	ihk	NE	1688036	1689424	462	-	3571164 COG0642T	two component system histidine kinase	*

M5005_Spy_1725c	irr	NE	1689421	1690074	217	-	3571165 COG0745TK	two-component response regulator	*
M5005_Spy_1726c	-	NE	1690168	1691385	405	-	3571166 COG0577V	ABC transporter permease	*
M5005_Spy_1727c	-	NE	1691398	1692072	224	-	3571167 COG1136V	ABC transporter ATP-binding protein	*
M5005_Spy_1728c	-	NE	1692059	1693327	422	-	3571168 COG0845M	periplasmic protein of efflux system	*
M5005_Spy_1729c	-	NE	1693750	1694154	134	-	3571169 -	hypothetical protein M5005_Spy_1729	*
M5005_Spy_1730c	-	NE	1694181	1694477	98	-	3571170 -	hypothetical protein M5005_Spy_1730	*
M5005_Spy_1731	grm	NE	1694721	1694957	78	+	3571171 -	hypothetical protein M5005_Spy_1731	
M5005_Spy_1732c	prsA	C	1695233	1696162	309	-	3571172 COG0760O	foldase PrsA	*
M5005_Spy_1733c	-	NE	1696218	1696535	105	-	3571173 -	hypothetical protein M5005_Spy_1733	
M5005_Spy_1734c	-	NE	1696642	1696947	101	-	3571135 -	hypothetical protein M5005_Spy_1734	
M5005_Spy_1735c	speB	NE	1696949	1698145	398	-	3571136 -	exotoxin B	*
M5005_Spy_1736	-	NC	1698670	1698801	43	+	3571137 -	hypothetical protein M5005_Spy_1736	
M5005_Spy_1737	rgg	NE	1699085	1699927	280	+	3571138 -	transcriptional regulator	*
M5005_Spy_1738c	spd	NE	1700168	1700983	271	-	3571139 -	phage-associated deoxyribonuclease	*
M5005_Spy_1739	-	NC	1701067	1701195	42	+	3571140 -	hypothetical protein M5005_Spy_1739	
M5005_Spy_1740	-	NE	1701347	1701856	169	+	3571141 COG1267I	low temperature requirement C protein	*
M5005_Spy_1741c	gldA	NE	1701938	1703026	362	-	3571142 COG0371C	glycerol dehydrogenase	*
M5005_Spy_1742c	mipB	NE	1703083	1703751	222	-	3571143 COG0176G	fructose-6-phosphate aldolase	*
M5005_Spy_1743c	pflD	NE	1703764	1706136	790	-	3571144 COG1882C	formate acetyltransferase	*
M5005_Spy_1744c	-	NE	1706391	1707695	434	-	3571145 COG1455G	IIC	*
M5005_Spy_1745c	-	NE	1707705	1708013	102	-	3571146 COG1440G	IIB	*
M5005_Spy_1746c	-	NE	1708041	1708361	106	-	3571147 COG1447G	IIA	*
M5005_Spy_1747c	-	NE	1708649	1709629	326	-	3571148 COG2390K	sorbitol operon regulator	*
M5005_Spy_1748c	-	NE	1709645	1710394	249	-	3571149 COG1349KG	DeoR family transcriptional regulator	*
M5005_Spy_1749	-	NE	1710517	1711290	257	+	3571150 COG1180O	pyruvate formate-lyase activating enzyme	*
M5005_Spy_1750	-	NE	1711325	1711525	66	+	3571151 -	hypothetical protein M5005_Spy_1750	
M5005_Spy_1751c	secE	NC	1711527	1711703	58	-	3571152 COG0690U	preprotein translocase subunit SecE	
M5005_Spy_1752c	rpmG	NC	1711717	1711869	50	-	3571153 COG0267J	50S ribosomal protein L33	
M5005_Spy_1753c	pbp2A	NE	1711918	1714254	778	-	3571116 COG0744M	multimodular transpeptidase-transglycosylase	*
M5005_Spy_1754c	-	NE	1714293	1714673	126	-	3571117 COG0251J	translation initiation inhibitor	*
M5005_Spy_1755	-	NE	1715164	1715355	63	+	3571118 -	hypothetical protein M5005_Spy_1755	
M5005_Spy_1756	-	NE	1715249	1716250	333	+	3571119 COG0564J	ribosomal large subunit pseudouridine synthase D	*
M5005_Spy_1757c	-	NE	1716339	1717979	546	-	3571120 COG4640S	hypothetical protein M5005_Spy_1757	*
M5005_Spy_1758	-	NE	1718226	1719722	498	+	3571121 COG4690E	dipeptidase B	*
M5005_Spy_1759c	-	NE	1720207	1720371	54	-	3571122 -	MutR family transcriptional regulator	
M5005_Spy_1760c	-	NE	1720350	1720532	60	-	3571123 -	MutR family transcriptional regulator	
M5005_Spy_1761c	groEL	E	1720925	1722556	543	-	3571124 COG0459O	molecular chaperone GroEL	*
M5005_Spy_1762c	groES	E	1722592	1722882	96	-	3571125 COG0234O	co-chaperonin GroES	*
M5005_Spy_1763c	clpC	NE	1723060	1725504	814	-	3571126 COG0542O	negative regulator of genetic competence	*
M5005_Spy_1764c	ctsR	NE	1725504	1725965	153	-	3571127 COG4463K	transcriptional regulator	*
M5005_Spy_1765c	csp	NE	1726161	1726370	69	-	3571128 COG1278K	cold shock protein	
M5005_Spy_1766c	-	NC	1726507	1726611	34	-	3571129 -	hypothetical protein M5005_Spy_1766	
M5005_Spy_1767c	-	NE	1726595	1727728	377	-	3571130 COG5433L	transposase	
M5005_Spy_1768	ahpC	NE	1728685	1729245	186	+	3571132 COG0450O	peroxiredoxin reductase (NAD(P)H)	*
M5005_Spy_1769	ahpF	NE	1729266	1730798	510	+	3571133 COG3634O	peroxiredoxin reductase (NAD(P)H)	*
M5005_Spy_1770c	hutI	NE	1730856	1732121	421	-	3571134 COG1228Q	imidazolonepropionase	*
M5005_Spy_1771	hutU	NE	1732413	1734443	676	+	3571097 COG2987E	urocanate hydratase	*
M5005_Spy_1772	-	NE	1734532	1735431	299	+	3571098 COG3643E	glutamate formiminotransferase	*
M5005_Spy_1773	-	NE	1735442	1736068	208	+	3571099 COG3404E	formiminotetrahydrofolate cyclodeaminase	*
M5005_Spy_1774	fhs.2	NE	1736086	1737759	557	+	3571100 COG2759F	formate--tetrahydrofolate ligase	*
M5005_Spy_1775	-	NE	1737781	1738377	198	+	3571101 COG3758S	hypothetical protein M5005_Spy_1775	*
M5005_Spy_1776	-	NE	1738597	1739940	447	+	3571102 COG0531E	amino acid permease	*
M5005_Spy_1777	hutH	NE	1739951	1741492	513	+	3571103 COG2986E	histidine ammonia-lyase	*
M5005_Spy_1778	hutG	NE	1741678	1742664	328	+	3571104 COG0010E	formimidoylglutamase	*
M5005_Spy_1779c	-	NE	1742695	1745769	1024	-	3571105 COG3899R	LuxR family transcriptional regulator	*
M5005_Spy_1780	rpsB	E	1746073	1746840	255	+	3571106 COG0052J	30S ribosomal protein S2	*
M5005_Spy_1781	tsf	E	1746974	1748014	346	+	3571107 COG0264J	elongation factor Ts	*
M5005_Spy_1782c	pepO	NE	1748180	1750075	631	-	3571108 COG3590O	neutral endopeptidase	*
M5005_Spy_1783c	dexS	NC	1750283	1751911	542	-	3571109 COG0366G	trehalose-6-phosphate hydrolase	*

M5005_Spy_1784c	-	NE	1751978	1754002	674	-	3571110 COG1263G	PTS system, trehalose-specific IIBC component	*
M5005_Spy_1785	-	NE	1754213	1754926	237	+	3571111 COG2188K	trehalose operon transcriptional repressor	*
M5005_Spy_1786c	-	NE	1755205	1755372	55	-	3571112 COG1733K	MarR family transcriptional regulator, partial	
M5005_Spy_1787	-	NE	1755730	1756587	285	+	3571113 COG2514R	glyoxalase	
M5005_Spy_1788	yaaA	NE	1756629	1757360	243	+	3571114 COG3022S	hypothetical protein M5005_Spy_1788	*
M5005_Spy_1789c	nrdG	NE	1757534	1758148	204	-	3571115 COG0602O	activating protein	*
M5005_Spy_1790c	-	NE	1758148	1758657	169	-	3571077 COG3981R	acetyltransferase	*
M5005_Spy_1791c	-	NC	1758666	1759601	311	-	3571078 COG0673R	virulence factor	*
M5005_Spy_1792c	-	NC	1759630	1759776	48	-	3571079 -	hypothetical protein M5005_Spy_1792	
M5005_Spy_1793c	nrdD	C	1759958	1762156	732	-	3571080 COG1328F	anaerobic ribonucleoside triphosphate reductase	*
M5005_Spy_1794c	-	NE	1762253	1763812	519	-	3571081 COG1807M	hypothetical protein M5005_Spy_1794	*
M5005_Spy_1795c	-	NE	1764225	1764530	101	-	3571082 COG3906S	hypothetical protein M5005_Spy_1795	*
M5005_Spy_1796c	-	E	1764542	1764961	139	-	3571083 COG0816L	Holliday junction resolvase-like protein	*
M5005_Spy_1797c	-	NE	1764958	1765227	89	-	3571084 COG4472S	hypothetical protein M5005_Spy_1797	
M5005_Spy_1798c	spxA	C	1765340	1765738	132	-	3571085 COG1393P	Spx family transcriptional regulator	*
M5005_Spy_1799c	recA	NE	1766029	1767165	378	-	3571086 COG0468L	recombinase A	*
M5005_Spy_1800c	cinA	NE	1767254	1768525	423	-	3571087 COG1058R	competence damage-inducible protein A	*
M5005_Spy_1801c	tag	NC	1768594	1769154	186	-	3571088 COG2818L	DNA-3-methyladenine glycosylase	*
M5005_Spy_1802c	ruvA	NC	1769164	1769760	198	-	3571089 COG0632L	Holliday junction DNA helicase RuvA	*
M5005_Spy_1803c	lmrP	NE	1769762	1770982	406	-	3571090 COG2814G	multidrug resistance protein B	*
M5005_Spy_1804c	mutL	NC	1770993	1772975	660	-	3571091 COG0323L	DNA mismatch repair protein	*
M5005_Spy_1805c	mutS	NC	1773104	1775659	851	-	3571092 COG0249L	DNA mismatch repair protein MutS	*
M5005_Spy_1806c	-	NE	1775646	1775852	68	-	3571093 COG4550S	hypothetical protein M5005_Spy_1806	
M5005_Spy_1807c	argR2	NE	1775995	1776432	145	-	3571094 COG1438K	arginine repressor ArgR	*
M5005_Spy_1808	argS	E	1776723	1778414	563	+	3571095 COG0018J	arginyl-tRNA synthetase	*
M5005_Spy_1809	uviB	NE	1778502	1778810	102	+	3571096 -	bacteriocin	*
M5005_Spy_1810c	-	NE	1778837	1779709	290	-	3571058 COG1284S	hypothetical protein M5005_Spy_1810	*
M5005_Spy_1811c	-	NE	1779752	1780687	311	-	3571059 COG1284S	hypothetical protein M5005_Spy_1811	*
M5005_Spy_1812c	-	NE	1780650	1781591	313	-	3571060 COG1284S	hypothetical protein M5005_Spy_1812	*
M5005_Spy_1813c	aspS	E	1781584	1783332	582	-	3571061 COG0173J	aspartyl-tRNA synthetase	*
M5005_Spy_1814c	hisS	E	1783670	1784950	426	-	3571062 COG0124J	histidyl-tRNA synthetase	*
M5005_Spy_1815	rpmF	NE	1785170	1785352	60	+	3571063 COG0333J	50S ribosomal protein L32	
M5005_Spy_1816	rpmG	NC	1785368	1785517	49	+	3571064 COG0267J	50S ribosomal protein L33	
M5005_Spy_1817	cadD	NE	1785810	1786424	204	+	3571065 COG4300P	cadmium resistance protein	*
M5005_Spy_1818	cadC	NE	1786406	1786774	122	+	3571066 COG0640K	cadmium efflux system accessory protein	*
M5005_Spy_1819	-	NE	1786825	1787748	307	+	3571067 -	hypothetical protein M5005_Spy_1819	
M5005_Spy_1820	-	NE	1787814	1788566	250	+	3571068 COG1674D	DNA translocase FtsK	*
M5005_Spy_1821	-	NE	1788563	1789165	200	+	3571069 -	hypothetical protein M5005_Spy_1821	*
M5005_Spy_1822c	-	NE	1789641	1789925	94	-	3571070 -	transcriptional regulator	
M5005_Spy_1823	-	NE	1790376	1791218	280	+	3571071 COG2035S	hypothetical protein M5005_Spy_1823	*
M5005_Spy_1824c	-	NE	1791265	1791906	213	-	3571072 COG1051F	phosphohydrolase	
M5005_Spy_1825	-	NE	1792113	1792439	108	+	3571073 COG1695K	PadR family transcriptional regulator	*
M5005_Spy_1826	-	NE	1792426	1793013	195	+	3571074 COG4709S	hypothetical protein M5005_Spy_1826	*
M5005_Spy_1827	-	NE	1793010	1794089	359	+	3571075 -	hypothetical protein M5005_Spy_1827	*
M5005_Spy_1828c	-	NE	1794207	1796075	622	-	3571076 COG1511S	phage infection protein	*
M5005_Spy_1830	-	NE	1796614	1797147	177	+	3571039 COG1309K	TetR family transcriptional regulator	*
M5005_Spy_1831c	rpsD	E	1797301	1797912	203	-	3571040 COG0522J	30S ribosomal protein S4	*
M5005_Spy_1832c	-	NC	1798115	1798219	34	-	3571041 -	hypothetical protein M5005_Spy_1832	
M5005_Spy_1833c	-	NE	1798203	1799336	377	-	3571042 COG5433L	transposase	
M5005_Spy_1834c	-	NE	1799968	1800240	90	-	3571043 COG4466S	hypothetical protein M5005_Spy_1834	*
M5005_Spy_1835c	holB/dnaC	E	1800257	1801624	455	-	3571044 COG0305L	replicative DNA helicase	*
M5005_Spy_1836c	rplI	NE	1801654	1802106	150	-	3571045 COG0359J	50S ribosomal protein L9	*
M5005_Spy_1837c	-	NE	1802103	1804079	658	-	3571046 COG3887T	phosphoesterase, DHH family protein	*
M5005_Spy_1838c	gidA	C	1804170	1806068	632	-	3571047 COG0445D	modification protein GidA	*
M5005_Spy_1839c	-	NE	1806192	1806509	105	-	3571048 COG1051F	phosphohydrolase	
M5005_Spy_1840c	mnmA	E	1807286	1808407	373	-	3571049 COG0482J	tRNA-specific 2-thiouridylase MnmA	*
M5005_Spy_1841	sdhB	NE	1808705	1809376	223	+	3571050 COG1760E	L-serine dehydratase	*
M5005_Spy_1842	sdhA	NE	1809388	1810260	290	+	3571051 COG1760E	L-serine dehydratase	*
M5005_Spy_1843c	-	NE	1810673	1811287	204	-	3571052 COG0741M	transglycosylase	*

M5005_Spy_1844c	cbiQ	C	1811659	1812459	266	-	3571053 COG0619P	cobalt transporter	*
M5005_Spy_1845c	cbiO	E	1812452	1813294	280	-	3571054 COG1122P	cobalt ABC transporter ATP-binding protein	*
M5005_Spy_1846c	cbiO	E	1813270	1814160	296	-	3571055 COG1122P	cobalt ABC transporter ATP-binding protein	*
M5005_Spy_1847c	pgsA	NC	1814111	1814653	180	-	3571056 COG0558I	phosphatidyltransferase	*
M5005_Spy_1848c	-	NE	1814667	1815692	341	-	3571057 COG1426S	hypothetical protein M5005_Spy_1848	*
M5005_Spy_1849c	-	NE	1815742	1817031	429	-	3571021 COG0612R	zinc protease	*
M5005_Spy_1850c	-	NE	1817033	1818277	414	-	3571022 COG0612R	zinc protease	*
M5005_Spy_1851	hasA	NE	1818708	1819967	419	+	3571023 COG1215M	hyaluronan synthase	
M5005_Spy_1852	hasB	NE	1820003	1821211	402	+	3571024 COG1004M	UDP-glucose 6-dehydrogenase	
M5005_Spy_1853	hasC	NE	1821393	1822307	304	+	3571025 COG1210M	UTP-glucose-1-phosphate uridylyltransferase	
M5005_Spy_1854	-	NE	1822615	1823028	137	+	3571026 COG2501S	hypothetical protein M5005_Spy_1854	*
M5005_Spy_1855	recF	NE	1823030	1824136	368	+	3571027 COG1195L	recombination protein F	*
M5005_Spy_1856c	-	NE	1824191	1825024	277	-	3571028 COG4975G	glucose uptake protein	*
M5005_Spy_1857c	guaB	NE	1825256	1826737	493	-	3571029 COG0516F	inosine 5\'-monophosphate dehydrogenase	*
M5005_Spy_1858c	trsA	E	1827045	1828067	340	-	3571030 COG0180J	tryptophanyl-tRNA synthetase	*
M5005_Spy_1859c	-	NC	1828159	1828284	41	-	3571031 -	hypothetical protein M5005_Spy_1859	
M5005_Spy_1860	-	NE	1828486	1829358	290	+	3571032 COG1284S	hypothetical protein M5005_Spy_1860	*
M5005_Spy_1861	-	NE	1829437	1831056	539	+	3571033 COG0488R	ABC transporter ATP-binding protein	*
M5005_Spy_1862	-	NE	1831139	1833715	858	+	3571034 COG4485S	ABC transporter permease	*
M5005_Spy_1863c	-	NE	1834881	1835162	93	-	3571037 COG2826L	transposase	
M5005_Spy_1864c	-	NE	1835744	1836223	159	-	3571018 COG1576S	rRNA large subunit methyltransferase	*
M5005_Spy_1865	htrA	C	1836435	1837658	407	+	3571019 COG0265O	protease Do	*
M5005_Spy_1866	parB	NE	1837717	1838523	268	+	3571020 COG1475K	chromosome partitioning protein	

Table S5. Bayesian analysis of Tn-seq datasets for GAS M49 NZ131 grown *in vitro*.

(a) Spy numbers from GAS NZ131 genome.

(b) When available, gene name is provided.

					Bayesian for T_0		Bayesian for T_1		Bayesian for T_2	
Locus Tag(a)	Gene Name(b)	Start	Stop	Length	Z score	Call	Z score	Call	Z score	Call
Spy49_0001	dnaA	232	1587	451	0.9995	E	1	E	0.7995	U
Spy49_0002	dnaN	1742	2878	378	1	E	0.99975	E	1	E
Spy49_0003	-	2953	3150	65	0	NE	0	NE	0	NE
Spy49_0004	-	3480	4595	371	0.00025	NE	0	NE	0	NE
Spy49_0005	pth	4665	5234	189	0.9995	E	1	E	1	E
Spy49_0006	trcF	5321	8740	1139	0.00025	NE	0	NE	0.00025	NE
Spy49_0008	-	8902	9174	90	0.00025	NE	0	NE	0	NE
Spy49_0009	divIC	9161	9532	123	0.21775	U	1	E	0.98775	U
Spy49_0010	-	9667	10953	428	0	NE	0	NE	0	NE
Spy49_0011	mesJ	10950	12236	428	0.996	E	1	E	1	E
Spy49_0012	hpt	12241	12783	180	0.00025	NE	0	NE	0.00025	NE
Spy49_0013	ftsH	12805	14784	659	0.99975	E	1	E	1	E
Spy49_0014	-	15041	16501	486	0.00025	NE	0	NE	0	NE
Spy49_0015	-	31169	32365	398	0.9945	E	1	E	0.99225	E
Spy49_0016	prsA	32618	33580	320	1	E	1	E	1	E
Spy49_0017	recO	33766	34521	251	0.00025	NE	0	NE	0	NE
Spy49_0018	-	34624	35631	335	0.04475	U	1	E	1	E
Spy49_0019	acp	35624	35866	80	0.99875	E	0.08725	U	0.9965	E
Spy49_0020	purC	35987	36721	244	0	NE	0	NE	0.004	NE
Spy49_0021	purL	36798	40571	1257	0.00975	NE	0	NE	0.01125	NE
Spy49_0022	purF	40805	42259	484	0	NE	0	NE	0.00025	NE
Spy49_0023	purM	42287	43309	340	0	NE	0	NE	0.00025	NE
Spy49_0024	purN	43477	44031	184	0	NE	0	NE	0.00075	NE
Spy49_0025	purH	44215	45762	515	0	NE	0	NE	0	NE
Spy49_0026c	-	45820	46944	374	0	NE	0	NE	0.00025	NE
Spy49_0027	purD	47107	48462	451	0	NE	0	NE	0.00025	NE
Spy49_0028	purE	48621	49232	203	0	NE	0	NE	0.002	NE
Spy49_0029	purK	49216	50292	358	0	NE	0	NE	0.00025	NE
Spy49_0030	-	50394	51875	493	0	NE	0	NE	0	NE
Spy49_0031	purB	52048	53340	430	0	NE	0	NE	0.01925	NE
Spy49_0032	-	53471	54391	306	0	NE	0	NE	0	NE
Spy49_0033	ruvB	54617	55615	332	0.01425	NE	0.90475	U	1	E
Spy49_0035	-	55753	56190	145	0	NE	0	NE	0	NE
Spy49_0036	-	56213	56614	133	0.00025	NE	0.99975	E	0.99625	E
Spy49_0037	-	56611	58386	591	0.666	U	1	E	1	E
Spy49_0038	adhE	58695	61337	880	0	NE	0	NE	0	NE
Spy49_0039	adhP	61589	62605	338	0	NE	0	NE	0	NE
Spy49_0040	norM	62993	64282	429	0	NE	0	NE	0	NE
Spy49_0044	rpsJ	64481	64789	102	0.9775	U	0.95575	U	0.004	NE
Spy49_0045	rplC	65037	65663	208	0.998	E	1	E	0.995	E
Spy49_0046	rplD	65687	66310	207	0.82225	U	1	E	0.0035	NE
Spy49_0048	rplW	66310	66540	76	0.95125	U	1	E	0.94925	U
Spy49_0049	rplB	66625	67458	277	1	E	1	E	1	E
Spy49_0050	rpsS	67597	67875	92	0.0005	NE	0.244	U	0.00075	NE
Spy49_0052	rplV	67891	68235	114	0.9925	U	1	E	0.64425	U
Spy49_0053	rpsC	68275	68901	208	0.987	U	1	E	0.9985	E
Spy49_0054	rplP	68905	69318	137	0.93375	U	0.99975	E	0.9955	E
Spy49_0055	rpmC	69328	69534	68	0.8785	U	0.99875	E	0.87975	U
Spy49_0056	rpsQ	69560	69820	86	0.9915	U	1	E	0.98825	U
Spy49_0057	rplN	69845	70213	122	0.99125	U	1	E	0.99	U
Spy49_0058	rplX	70292	70597	101	0.00025	NE	1	E	0.995	E
Spy49_0059	rplE	70621	71163	180	0.9995	E	1	E	0.01425	NE
Spy49_0060	rpsN.1	71179	71364	61	0.92125	U	0.13125	U	0.01	NE
Spy49_0061	rpsH	71515	71913	132	0.99525	E	1	E	0.98825	U
Spy49_0062	rplF	72116	72652	178	0.994	E	1	E	0.9935	E
Spy49_0064	rplR	72757	73113	118	0.987	U	1	E	0.98025	U
Spy49_0065	rpsE	73132	73626	164	0.99425	E	1	E	0.96625	U
Spy49_0066	rpmD	73641	73823	60	0.00025	NE	1	E	0.0005	NE
Spy49_0067	rplO	74038	74478	146	0.96925	U	0.9995	E	0.96575	U
Spy49_0068	secY	74495	75799	434	1	E	1	E	1	E
Spy49_0069	adk	75949	76587	212	0.99775	E	1	E	0.99975	E
Spy49_0070	infA	76705	76923	72	0.98475	U	1	E	0.03775	NE

Spy49_0071	rpmJ	76949	77065	38	-1	S	-1	S	-1	S
Spy49_0072	rpsM	77083	77448	121	0.0015	NE	1	E	0.992	E
Spy49_0073	rpsK	77466	77849	127	0.979	U	0.0005	NE	0.94975	U
Spy49_0074	rpoA	77895	78833	312	1	E	1	E	1	E
Spy49_0075	rplQ	78848	79234	128	0.98375	U	0.00575	NE	0.97975	U
Spy49_0078	-	87367	87615	82	0	NE	0	NE	0.0185	NE
Spy49_0079	-	87670	87882	70	0.00175	NE	0	NE	0	NE
Spy49_0080	adrR	87992	88435	147	0.00025	NE	0	NE	0	NE
Spy49_0081	adrC	88439	89158	239	0.00025	NE	0	NE	0.1085	U
Spy49_0082	adrB	89151	89966	271	0	NE	0	NE	0.62175	U
Spy49_0083c	-	90006	90389	127	0	NE	0	NE	0	NE
Spy49_0084	tyrS	90440	91696	418	0.941	U	1	E	1	E
Spy49_0086	pbp1b	91800	94100	766	0.00025	NE	0	NE	0.00075	NE
Spy49_0087	rpoB	94364	97930	1188	1	E	1	E	1	E
Spy49_0088	rpoC	98048	101662	1204	1	E	1	E	1	E
Spy49_0089	-	101814	102179	121	0	NE	0	NE	0	NE
Spy49_0090	comYA	102272	103210	312	0.00025	NE	0	NE	0.00025	NE
Spy49_0091	comYB	103305	104180	291	0.00025	NE	0	NE	0	NE
Spy49_0092	comYC	104182	104508	108	0	NE	0	NE	0	NE
Spy49_0093	-	104483	104911	142	0.00025	NE	0	NE	0.00025	NE
Spy49_0094	-	104868	105152	94	0	NE	0	NE	0	NE
Spy49_0095	comYD	105145	105579	144	0.01125	NE	0	NE	0.00025	NE
Spy49_0096	-	105599	105889	96	0	NE	0	NE	0	NE
Spy49_0097	-	105987	106940	317	0.00025	NE	0	NE	0	NE
Spy49_0098	ackA	106999	108195	398	0.00025	NE	0	NE	0.587	U
Spy49_0099	-	108381	108623	80	0	NE	0	NE	0.00025	NE
Spy49_0100	proC	108772	109542	256	0	NE	0	NE	0	NE
Spy49_0101	pepA	109590	110657	355	0	NE	0	NE	0.0005	NE
Spy49_0103	-	112439	112732	97	0	NE	0	NE	0	NE
Spy49_0104	trx.1	112729	113046	105	0.00025	NE	0	NE	0	NE
Spy49_0105	-	113064	113708	214	0	NE	0	NE	0	NE
Spy49_0107	ssb	113842	114237	131	0	NE	0	NE	0	NE
Spy49_0108c	-	114497	115138	213	0.00025	NE	0	NE	0	NE
Spy49_0109c	-	115158	116135	325	0	NE	0	NE	0.00025	NE
Spy49_0110c	hslO	116122	116994	290	0.00025	NE	0	NE	0	NE
Spy49_0111	nra	117120	118655	511	0.00025	NE	0	NE	0.00025	NE
Spy49_0112	cbp	119085	121319	744	0	NE	0	NE	0.00025	NE
Spy49_0113	lepA-1	121312	121833	173	0	NE	0	NE	0.00025	NE
Spy49_0114	-	121855	122889	344	0	NE	0	NE	0.00025	NE
Spy49_0116	-	122905	123630	241	0	NE	0	NE	0.00025	NE
Spy49_0117	-	123665	124234	189	0	NE	0	NE	0	NE
Spy49_0118	msmR	124394	125599	401	0	NE	0	NE	0	NE
Spy49_0119	prtF	125984	129466	1160	0	NE	0	NE	0	NE
Spy49_0120c	-	129731	130396	221	0.00025	NE	0	NE	0	NE
Spy49_0121	atoE	130750	132156	468	0.00025	NE	0	NE	0.00025	NE
Spy49_0122c	-	132231	133142	303	0	NE	0	NE	0	NE
Spy49_0123	-	133263	134447	394	0	NE	0	NE	0	NE
Spy49_0124	atoD.2	134459	135118	219	0	NE	0	NE	0	NE
Spy49_0125	-	135121	135768	215	0	NE	0	NE	0	NE
Spy49_0126c	-	135890	136570	226	0	NE	0	NE	0	NE
Spy49_0127	-	136745	137110	121	0	NE	0	NE	0	NE
Spy49_0128	sloR	137147	138166	339	0	NE	0	NE	0.01125	NE
Spy49_0129	-	138620	138940	106	0	NE	0	NE	0	NE
Spy49_0130	ntpI	138930	140930	666	0	NE	0	NE	0.00025	NE
Spy49_0131	ntpK	140932	141411	159	0	NE	0	NE	0.00025	NE
Spy49_0132	ntpE	141479	142063	194	0.00025	NE	0	NE	0	NE
Spy49_0133	ntpC	142079	143077	332	0	NE	0	NE	0	NE
Spy49_0134	ntpG	143074	143394	106	0	NE	0	NE	0	NE
Spy49_0135	ntpA	143595	145370	591	0	NE	0	NE	0	NE
Spy49_0136	ntpB	145371	146786	471	0	NE	0	NE	0.00075	NE
Spy49_0137	ntpD	146831	147457	208	0	NE	0	NE	0	NE
Spy49_0138c	-	147577	148839	420	0	NE	0	NE	0.00025	NE
Spy49_0139c	-	148852	149730	292	0	NE	0	NE	0	NE
Spy49_0141	purA	150168	151460	430	0.00125	NE	0	NE	0	NE
Spy49_0142	-	151787	152830	347	0	NE	0	NE	0	NE
Spy49_0143	nusG	152988	153542	184	0.593	U	0	NE	0.9595	U
Spy49_0144	nga	153917	155272	451	0	NE	0	NE	0.0005	NE
Spy49_0145	-	155277	155762	161	0	NE	0	NE	0.00025	NE

Spy49_0146	slo	155786	157501	571	0	NE	0	NE	0.00025	NE
Spy49_0147	-	157756	158184	142	0.99525	E	0.50925	U	0.00375	NE
Spy49_0148	-	158408	158701	97	0.00025	NE	0.3225	U	0.00025	NE
Spy49_0149c	-	158998	159165	55	0	NE	0	NE	0.00025	NE
Spy49_0150c	-	159350	159676	108	0.99925	E	1	E	0.999	E
Spy49_0151	metB	159929	161299	456	0	NE	0	NE	0	NE
Spy49_0152	leuS	161510	164011	833	1	E	1	E	1	E
Spy49_0153	-	164318	165751	477	0	NE	0	NE	0	NE
Spy49_0154	-	165822	166100	92	0	NE	0	NE	0	NE
Spy49_0155	-	166223	166708	161	0	NE	0	NE	0	NE
Spy49_0156	ulaD	166799	167461	220	0	NE	0	NE	0.086	U
Spy49_0157	-	167466	168329	287	0	NE	0	NE	0	NE
Spy49_0158	araD	168331	169035	234	0	NE	0	NE	0	NE
Spy49_0159	-	169361	171007	548	0	NE	0	NE	0	NE
Spy49_0160	-	171261	172352	363	0	NE	0	NE	0	NE
Spy49_0161	opuAA	172842	174038	398	0	NE	0	NE	0	NE
Spy49_0162	opuABC	174054	175781	575	0	NE	0	NE	0	NE
Spy49_0163	polA	175912	178554	880	0.00025	NE	1	E	1	E
Spy49_0164	-	178732	179187	151	0	NE	0	NE	0	NE
Spy49_0165	-	179239	179706	155	0	NE	0	NE	0	NE
Spy49_0166	-	179862	180161	99	0	NE	0	NE	0	NE
Spy49_0167	-	180383	181717	444	0	NE	0	NE	0	NE
Spy49_0168	-	181710	182090	126	0	NE	0	NE	0	NE
Spy49_0169	-	182234	182443	69	0.0125	NE	0	NE	0	NE
Spy49_0170c	-	182570	182785	71	0	NE	0	NE	0	NE
Spy49_0173c	-	183427	183720	97	0	NE	0	NE	0	NE
Spy49_0176	-	183438	183638	290	0	NE	0	NE	0.00025	NE
Spy49_0178c	-	185332	186204	148	0	NE	0	NE	0	NE
Spy49_0180c	-	186865	187311	287	0	NE	0	NE	0	NE
Spy49_0181	-	187933	188796	380	0	NE	0	NE	0	NE
Spy49_0182	tgt	189015	190157	103	0	NE	0	NE	0	NE
Spy49_0183	-	190374	190685	179	0	NE	0	NE	0	NE
Spy49_0184	bioY	190689	191228	259	0	NE	0	NE	0	NE
Spy49_0185	-	191368	192147	171	0	NE	0	NE	0	NE
Spy49_0186c	-	192147	192662	406	0	NE	0	NE	0.00025	NE
Spy49_0187	-	193276	194496	234	0.00025	NE	0	NE	0	NE
Spy49_0188	speG	194908	195612	449	1	E	1	E	1	E
Spy49_0189c	pgi	196067	197416	465	0	NE	0	NE	0	NE
Spy49_0190	-	197875	199272	223	0.00025	NE	0	NE	0.00025	NE
Spy49_0191c	-	199964	200635	299	0.96075	U	1	E	1	E
Spy49_0192c	hasC.2	200734	201633	338	0.99975	E	1	E	1	E
Spy49_0193	gpsA	201666	202682	68	0.00025	NE	0	NE	0	NE
Spy49_0194	-	203220	203426	568	0	NE	0	NE	0.62625	U
Spy49_0195	-	203419	205125	594	0.00025	NE	0	NE	0.0065	NE
Spy49_0198	-	205128	206912	230	0	NE	0	NE	0	NE
Spy49_0199	-	207105	207797	152	0	NE	0	NE	0	NE
Spy49_0200	dut	207907	208365	464	0.00025	NE	0	NE	0.00025	NE
Spy49_0201	radA	208402	209796	165	0	NE	0.00075	NE	0.674	U
Spy49_0202	-	209985	210482	236	0	NE	0	NE	0	NE
Spy49_0203	-	210613	211323	496	0.99875	E	1	E	1	E
Spy49_0204	gltX	211506	212996	448	0.00025	NE	0	NE	0	NE
Spy49_0205	fasB	213347	214693	427	0.00025	NE	0	NE	0.1585	U
Spy49_0206	-	214690	215973	246	0	NE	0	NE	0.00025	NE
Spy49_0207	fasA	215977	216717	119	0.988	U	0.0005	NE	0.00025	NE
Spy49_0208	rnpA	217257	217616	269	0	NE	0	NE	0	NE
Spy49_0209	-	217600	218409	304	0.00025	NE	0	NE	0.98625	U
Spy49_0210	-	218421	219335	44	-1	S	-1	S	-1	S
Spy49_0211	rpmH	219650	219784	234	0.00025	NE	0	NE	0	NE
Spy49_0212	-	220058	220762	439	0	NE	0	NE	0.00025	NE
Spy49_0213	-	220811	222130	295	0	NE	0	NE	0.00025	NE
Spy49_0214	-	222235	223122	276	0.00025	NE	0	NE	0.03225	NE
Spy49_0215	-	223135	223965	201	0	NE	0	NE	0.00025	NE
Spy49_0216	-	224179	224784	304	0	NE	0	NE	0	NE
Spy49_0217	nanH	224796	225710	312	0	NE	0	NE	0	NE
Spy49_0218c	-	225732	226670	276	0	NE	0	NE	0	NE
Spy49_0219	-	226781	227611	274	0	NE	0	NE	0	NE
Spy49_0220	-	227863	228687	196	0	NE	0	NE	0.00025	NE
Spy49_0221	-	228659	229249	290	0	NE	0	NE	0.00025	NE

Spy49_0223	ksgA	229363	230235	290	0	NE	0	NE	0.00025	NE
Spy49_0224	engC	230662	231534	220	0	NE	0.61175	U	0.97475	U
Spy49_0225	rpe	231544	232206	210	0	NE	0	NE	0.00025	NE
Spy49_0226	-	232199	232831	423	0.00025	NE	0	NE	0.0005	NE
Spy49_0227	-	232833	234104	312	0	NE	0	NE	0	NE
Spy49_0228	cbf	234094	235032	279	0.00025	NE	0	NE	0	NE
Spy49_0229	purR	235298	236137	873	0	NE	0	NE	0	NE
Spy49_0230	prgA	236128	238749	134	0.01	NE	1	E	0.96225	U
Spy49_0231	rpsL	238963	239367	156	0.9985	E	1	E	0.996	E
Spy49_0233	rpsG	239388	239858	692	1	E	1	E	1	E
Spy49_0234	fusA	240225	242303	336	0.99875	E	1	E	1	E
Spy49_0235c	plr	242651	243661	38	-1	S	-1	S	-1	S
Spy49_0236c	-	243887	244003	246	0.0035	NE	0	NE	0	NE
Spy49_0237c	-	244145	244885	498	0.00025	NE	0	NE	0.0005	NE
Spy49_0238	-	244951	246447	604	0	NE	0	NE	0.00025	NE
Spy49_0239	-	246645	248459	279	0.00025	NE	0	NE	0.00025	NE
Spy49_0240	bacA	248637	249476	253	0	NE	0	NE	0.00025	NE
Spy49_0241	mecA	249622	250383	389	0.99975	E	1	E	1	E
Spy49_0242	rgpG	250390	251559	256	0	NE	0	NE	0.97375	U
Spy49_0243	-	251681	252451	420	0.00025	NE	0	NE	0.9965	E
Spy49_0244	-	252546	253808	408	0	NE	0	NE	0.4695	U
Spy49_0245	-	253839	255065	159	0	NE	0	NE	0.2945	U
Spy49_0246	-	255052	255531	472	0.00025	NE	0	NE	0.8825	U
Spy49_0247	-	255524	256942	393	0	NE	0	NE	0	NE
Spy49_0248c	pbp7	257094	258275	395	0	NE	0	NE	0.00025	NE
Spy49_0249	dacA	258489	259676	656	0	NE	0	NE	0	NE
Spy49_0250	oppA	260007	261977	500	0	NE	0	NE	0	NE
Spy49_0251	oppB	262042	263544	308	0.00025	NE	0	NE	0	NE
Spy49_0252	oppC	263544	264470	356	0	NE	0	NE	0	NE
Spy49_0253	oppD	264479	265549	307	0	NE	0	NE	0	NE
Spy49_0254c	oppF	265542	266465	79	0	NE	0	NE	0	NE
Spy49_0256	-	266613	266852	161	0	NE	0	NE	0	NE
Spy49_0257	comX1	272853	273338	194	0.01875	NE	0.9105	U	1	E
Spy49_0258	-	273839	274423	373	0.9945	E	1	E	1	E
Spy49_0259	-	274423	275544	102	0	NE	0	NE	0.00025	NE
Spy49_0260	-	275569	275877	210	0.02375	NE	1	E	0.79075	U
Spy49_0261	nadD	275946	276578	197	0.00075	NE	0	NE	0.00025	NE
Spy49_0262	-	276575	277168	125	0	NE	0	NE	0	NE
Spy49_0263	-	277168	277545	247	0	NE	0	NE	0.00025	NE
Spy49_0264	-	277593	278336	368	0	NE	0	NE	0	NE
Spy49_0265	-	278558	279664	238	0	NE	0	NE	0	NE
Spy49_0266	-	280146	280862	215	0	NE	0	NE	0.98175	U
Spy49_0268	atmA	281260	281907	281	0	NE	0	NE	0	NE
Spy49_0269	atmB	282234	283079	354	0	NE	0	NE	0.00025	NE
Spy49_0270	atmD	283329	284393	230	0	NE	0	NE	0.00025	NE
Spy49_0271c	atmE	284394	285086	444	0	NE	0	NE	0	NE
Spy49_0272	braB	285140	286474	379	0.00025	NE	0	NE	0	NE
Spy49_0273c	-	286820	287959	224	0.89775	U	1	E	0.99975	E
Spy49_0274c	-	288014	288688	463	0.89875	U	1	E	1	E
Spy49_0275	-	288698	290089	237	0.00025	NE	0	NE	0	NE
Spy49_0276	gidB	290158	290871	185	0	NE	0	NE	0.00025	NE
Spy49_0277	lemA	291021	291578	298	0	NE	0	NE	0.00025	NE
Spy49_0278	htpX	291625	292521	177	0.00025	NE	0	NE	0	NE
Spy49_0279	-	292755	293288	228	0.99975	E	1	E	0.98625	U
Spy49_0280	csrR	293555	294241	500	0.0005	NE	1	E	1	E
Spy49_0281	csrS	294247	295749	164	0.69775	U	0	NE	0.996	E
Spy49_0282	nrdR	295964	296458	391	0.99975	E	1	E	1	E
Spy49_0283	dnaB	296442	297617	300	0.99575	E	1	E	0.99975	E
Spy49_0284	dnaI	297618	298520	436	1	E	1	E	1	E
Spy49_0285	engA	298583	299893	1032	0.042	U	0	NE	0.1135	U
Spy49_0286	snf	300100	303198	200	0	NE	0	NE	0	NE
Spy49_0287	-	303442	304044	442	1	E	1	E	1	E
Spy49_0288	murC	304084	305412	160	0	NE	0	NE	0	NE
Spy49_0289	-	305458	305940	522	0.00025	NE	0	NE	0.38975	U
Spy49_0290	-	306058	307626	176	0.00025	NE	0	NE	0.123	U
Spy49_0291	greA	307651	308181	307	1	E	1	E	1	E
Spy49_0292c	oxaA	308800	309723	92	0	NE	0	NE	0	NE
Spy49_0293	-	309805	310083	245	0	NE	0	NE	0	NE

Spy49_0294	-	310344	311081	166	0	NE	0	NE	0	NE
Spy49_0295	-	311120	311620	229	0.422	U	0	NE	0.65875	U
Spy49_0296	-	311635	312324	80	0.98775	U	1	E	0.9805	U
Spy49_0297	-	312502	312744	264	0.99625	E	1	E	1	E
Spy49_0298	glr	312922	313716	328	0.00025	NE	0	NE	0	NE
Spy49_0299	-	313713	314699	173	0.00025	NE	0	NE	0	NE
Spy49_0300	-	314678	315199	153	0.00025	NE	0	NE	0.00025	NE
Spy49_0301	-	315196	315657	248	0.00025	NE	0	NE	0.00025	NE
Spy49_0302	xerD	315654	316400	234	0.00025	NE	0	NE	0.00025	NE
Spy49_0303	scpA	316400	317104	183	0	NE	0	NE	0.0005	NE
Spy49_0304	scpB	317101	317652	240	0	NE	0	NE	0	NE
Spy49_0305	rluB	317773	318495	86	0	NE	0	NE	0.0035	NE
Spy49_0306	-	318495	318755	182	0.00025	NE	0	NE	0.00025	NE
Spy49_0307	-	318933	319481	178	0.00025	NE	0.00025	NE	0.00075	NE
Spy49_0308	-	319792	320328	152	0.00025	NE	0	NE	0.00025	NE
Spy49_0309	-	320553	321011	306	0.00025	NE	0	NE	0.00025	NE
Spy49_0310	-	321303	322223	184	0	NE	0	NE	0.00025	NE
Spy49_0311	-	322262	322816	444	0.00025	NE	0	NE	0.00025	NE
Spy49_0312	hlyX	322950	324284	287	0	NE	0	NE	0	NE
Spy49_0313	pflC	324290	325153	311	1	E	1	E	1	E
Spy49_0314	-	325284	326219	217	0	NE	0	NE	0.00025	NE
Spy49_0315	-	326295	326948	345	0.00025	NE	0	NE	0	NE
Spy49_0316	fhuG	326993	328030	350	0	NE	0	NE	0.00025	NE
Spy49_0317c	fhuB1	327991	329043	310	0	NE	0	NE	0.00025	NE
Spy49_0318	fhuD	329033	329965	260	0	NE	0	NE	0.00025	NE
Spy49_0319c	fhuC1	329991	330773	481	1	E	1	E	1	E
Spy49_0320	murE	331019	332464	544	1	E	1	E	1	E
Spy49_0322	-	332552	334186	209	0	NE	0	NE	0	NE
Spy49_0323	upp	334353	334982	196	0.07225	U	0.99575	E	0.999	E
Spy49_0325	clpP	335206	335796	91	0	NE	0	NE	0	NE
Spy49_0326	-	336288	336563	211	0.5155	U	1	E	0.99775	E
Spy49_0327	tmk	336812	337447	291	0.972	U	1	E	1	E
Spy49_0328	-	337465	338340	53	-1	S	-1	S	-1	S
Spy49_0329	-	338688	338849	107	0.00025	NE	0.0405	NE	0.99325	E
Spy49_0330	-	339004	339327	287	0.13275	U	0.00025	NE	1	E
Spy49_0331	-	339332	340195	130	0	NE	0	NE	0	NE
Spy49_0332c	-	340222	340614	209	0.00025	NE	0	NE	0.00025	NE
Spy49_0333	-	340661	341290	118	0.00025	NE	0	NE	0	NE
Spy49_0334c	-	341597	341953	303	0.00025	NE	0	NE	0.00025	NE
Spy49_0335	exoA	342027	342938	393	0.00025	NE	0	NE	0.00025	NE
Spy49_0336	lctO	343088	344269	1621	0	NE	0	NE	0.005	NE
Spy49_0337	prtS	344611	349476	217	0.00025	NE	0	NE	0	NE
Spy49_0338	-	350303	350956	666	1	E	1	E	1	E
Spy49_0339	metS	351196	353196	337	0.00025	NE	0	NE	0.00025	NE
Spy49_0340	nrdF.2	353694	354707	162	0	NE	0	NE	0	NE
Spy49_0341	nrdI	354711	355199	726	0	NE	0	NE	0	NE
Spy49_0342c	nrdE.2	355166	357346	250	0	NE	0	NE	0.00125	NE
Spy49_0343	-	357508	358260	72	-1	S	-1	S	-1	S
Spy49_0344	-	358946	359164	67	-1	S	-1	S	-1	S
Spy49_0345c	-	359112	359315	65	0.0005	NE	0	NE	0	NE
Spy49_0346c	-	359568	359765	277	0.00025	NE	0	NE	0.0005	NE
Spy49_0347	-	360026	360859	89	0	NE	0	NE	0	NE
Spy49_0348	-	361343	361612	134	0	NE	0	NE	0	NE
Spy49_0349c	-	362242	362646	129	0	NE	0	NE	0	NE
Spy49_0350c	-	363232	363621	230	0	NE	0	NE	0	NE
Spy49_0351c	-	364340	365032	60	0	NE	0	NE	0.00075	NE
Spy49_0352c	-	365190	365372	61	0.03075	NE	0	NE	0.00025	NE
Spy49_0353c	-	365511	365696	122	0	NE	0	NE	0.00025	NE
Spy49_0354c	-	365740	366108	57	-1	S	-1	S	-1	S
Spy49_0355c	-	366083	366256	137	0	NE	0	NE	0	NE
Spy49_0356c	-	366619	367032	168	0	NE	0	NE	0.00025	NE
Spy49_0357c	-	367138	367644	109	0.00025	NE	0	NE	0	NE
Spy49_0358c	-	367681	368010	200	0	NE	0	NE	0.00025	NE
Spy49_0359c	-	368000	368602	88	0	NE	0	NE	0	NE
Spy49_0360c	-	368868	369134	57	0	NE	0	NE	0	NE
Spy49_0361c	-	369315	369488	562	0	NE	0	NE	0	NE
Spy49_0362c	-	369776	371464	285	0.00025	NE	0	NE	0.00025	NE
Spy49_0363c	-	371433	372290	92	0.0345	NE	0	NE	0.00025	NE

Spy49_0364c	-	372434	372712	37	-1	S	-1	S	-1	S
Spy49_0365c	-	372715	372828	64	0	NE	0	NE	0	NE
Spy49_0366c	-	373055	373249	69	0	NE	0	NE	0	NE
Spy49_0367c	-	373246	373455	82	0	NE	0	NE	0	NE
Spy49_0368c	-	373980	374228	61	0	NE	0	NE	0	NE
Spy49_0369	-	374244	374429	222	1	E	1	E	1	E
Spy49_0370	-	374597	375265	381	0	NE	0	NE	0	NE
Spy49_0371	-	375347	376492	78	0	NE	0	NE	0	NE
Spy49_0372	-	377362	377598	232	0	NE	0	NE	0.00025	NE
Spy49_0373	fabG	377591	378289	319	0	NE	0	NE	0	NE
Spy49_0374	-	378365	379324	445	0	NE	0	NE	0	NE
Spy49_0375	glpT	379657	380994	460	0.99925	E	1	E	1	E
Spy49_0376	glmU	381167	382549	184	0	NE	0	NE	0	NE
Spy49_0377	-	382580	383134	83	0	NE	0	NE	0	NE
Spy49_0378	-	383134	383385	231	0.85825	U	1	E	0.9835	U
Spy49_0379	pfs	383405	384100	113	0	NE	0	NE	0	NE
Spy49_0380c	-	384251	384592	215	0	NE	0	NE	0.8575	U
Spy49_0381	-	384689	385336	274	0.03475	NE	0	NE	0	NE
Spy49_0382	mtsA	385590	386414	241	0.01325	NE	0	NE	0	NE
Spy49_0383	mtsB	386478	387203	284	0	NE	0	NE	0	NE
Spy49_0384c	mtsC	387204	388058	268	0.00025	NE	0	NE	0.00025	NE
Spy49_0385	cypB	388206	389012	801	1	E	1	E	1	E
Spy49_0386c	ftsK	389229	391634	116	0.00025	NE	0	NE	0	NE
Spy49_0387	-	391704	392054	141	0.02	NE	0.9995	E	0.982	U
Spy49_0388	rplK	392301	392726	229	1	E	1	E	0.9995	E
Spy49_0390	rplA	392832	393521	242	1	E	1	E	1	E
Spy49_0391	pyrH	393843	394571	185	0.44075	U	1	E	0.99725	E
Spy49_0392	frr	394600	395157	285	0	NE	0	NE	0	NE
Spy49_0394	-	395266	396123	169	0	NE	0	NE	0	NE
Spy49_0395	mrsA	396196	396705	71	0	NE	0	NE	0	NE
Spy49_0396	-	396702	396917	389	0	NE	0	NE	0	NE
Spy49_0398	-	397073	398242	603	0	NE	0	NE	0	NE
Spy49_0399	-	398517	400328	350	0	NE	0	NE	0	NE
Spy49_0400	phoH	400487	401539	191	0	NE	0	NE	0	NE
Spy49_0401	-	401585	402160	165	0.945	U	1	E	0.996	E
Spy49_0402	-	402319	402816	135	0.99725	E	1	E	0.998	E
Spy49_0403	dgk	402797	403204	298	0.914	U	1	E	1	E
Spy49_0404	era	403324	404220	158	0	NE	0	NE	0	NE
Spy49_0405c	-	404241	404717	84	0.00025	NE	0	NE	0.0005	NE
Spy49_0406	-	405023	405277	60	0	NE	0	NE	0	NE
Spy49_0407c	-	405910	406092	49	-1	S	-1	S	-1	S
Spy49_0408c	-	406430	406579	140	0	NE	0	NE	0	NE
Spy49_0409	-	406576	406998	75	0	NE	0	NE	0	NE
Spy49_0410	-	407316	407543	86	0	NE	0	NE	0	NE
Spy49_0411	-	408295	408555	123	0	NE	0	NE	0	NE
Spy49_0412	-	408771	409142	355	0.00025	NE	0	NE	0.0795	U
Spy49_0413	-	409427	410494	132	0	NE	0	NE	0.00025	NE
Spy49_0414c	-	410587	410985	89	0	NE	0	NE	0	NE
Spy49_0415	-	411333	411602	288	0.00025	NE	0	NE	0.00025	NE
Spy49_0416	mutR	412673	413539	275	0	NE	0	NE	0	NE
Spy49_0417	fpg	413711	414538	203	0.9985	E	0.99675	E	0.99975	E
Spy49_0418	coaE	414517	415128	500	0.5455	U	0	NE	0	NE
Spy49_0419	-	415318	416820	397	0	NE	0	NE	0.00025	NE
Spy49_0420	-	416942	418135	48	-1	S	-1	S	-1	S
Spy49_0421	rpmG	418132	418278	78	0.995	E	1	E	0.989	U
Spy49_0422	secG	418324	418560	777	0.00025	NE	0	NE	0.1865	U
Spy49_0423	vacB	418654	420987	155	0.00025	NE	0.00025	NE	0.98825	U
Spy49_0424	smpB	420990	421457	236	0	NE	0	NE	0.00025	NE
Spy49_0425c	-	421472	422182	215	0	NE	0	NE	0	NE
Spy49_0426c	pcp	422300	422947	308	0.00025	NE	0	NE	0	NE
Spy49_0427c	-	422998	423924	227	0.00025	NE	0	NE	0.00025	NE
Spy49_0428c	-	423924	424607	308	0	NE	0	NE	0.00025	NE
Spy49_0429c	-	424818	425744	125	0.00025	NE	0	NE	0	NE
Spy49_0430c	gloA	425889	426266	221	0	NE	0	NE	0	NE
Spy49_0431c	-	426277	426942	361	0	NE	0	NE	0	NE
Spy49_0432	pepQ	426991	428076	333	0.959	U	0.999	E	1	E
Spy49_0433	ccpA	428250	429251	332	0.981	U	1	E	1	E
Spy49_0434	-	429382	430380	444	0.99675	E	1	E	0.99475	E

Spy49_0435	-	430382	431716	647	1	E	1	E	1	E
Spy49_0436	thrS	432138	434081	330	0	NE	0	NE	0.001	NE
Spy49_0437	tagH	434222	435214	272	0.00025	NE	0	NE	0.00025	NE
Spy49_0438	-	435216	436034	261	0	NE	0	NE	0.00025	NE
Spy49_0439	-	436036	436821	49	-1	S	-1	S	-1	S
Spy49_0440	-	437022	437171	382	0	NE	0	NE	0	NE
Spy49_0441	-	437566	438714	415	0.00025	NE	0	NE	0.00025	NE
Spy49_0442	-	438671	439918	344	0	NE	0	NE	0	NE
Spy49_0444	-	439974	441008	236	1	E	1	E	0.9995	E
Spy49_0445	vicR	441170	441880	450	0.00025	NE	0	NE	0	NE
Spy49_0446	vicK	441873	443225	269	0.00025	NE	0	NE	0	NE
Spy49_0447	vicX	443229	444038	230	0.99775	E	0.444	U	0.99725	E
Spy49_0448	rnc	444483	445175	1179	0.00025	NE	0	NE	0.15575	U
Spy49_0449c	smc	445176	448715	283	0.00025	NE	0	NE	0.00025	NE
Spy49_0450	-	448963	449814	290	0	NE	0	NE	0.00025	NE
Spy49_0451	aroE	450088	450960	278	0.00025	NE	0	NE	0.01975	NE
Spy49_0453	-	450957	451793	244	0.00025	NE	0	NE	0.9755	U
Spy49_0454	-	451795	452529	328	0	NE	0	NE	0.00025	NE
Spy49_0455	-	452522	453508	399	0	NE	0	NE	0.00025	NE
Spy49_0456	-	453518	454717	300	0	NE	0	NE	0.00025	NE
Spy49_0457	-	454701	455603	329	0.00025	NE	0	NE	0.00025	NE
Spy49_0458	-	455724	456713	88	0	NE	0	NE	0.00025	NE
Spy49_0459	-	457116	457382	385	0.00025	NE	0	NE	0.00025	NE
Spy49_0460	-	457406	458563	389	0.00025	NE	0	NE	0	NE
Spy49_0461c	-	458648	459817	69	0	NE	0	NE	0	NE
Spy49_0462c	-	459958	460167	552	0	NE	0	NE	0	NE
Spy49_0463c	-	460157	461815	74	0	NE	0	NE	0	NE
Spy49_0464c	-	461806	462030	296	0.00025	NE	0	NE	0	NE
Spy49_0465	-	462034	462924	52	-1	S	-1	S	-1	S
Spy49_0466	-	462958	463116	100	0.0145	NE	0	NE	0.033	NE
Spy49_0467	-	463171	463473	58	0.00025	NE	0	NE	0	NE
Spy49_0468	-	463602	463778	150	0	NE	0	NE	0	NE
Spy49_0469	-	464174	464626	102	0	NE	0	NE	0.00025	NE
Spy49_0470c	-	464692	465000	62	0.0005	NE	0	NE	0	NE
Spy49_0471	-	465238	465426	107	0	NE	0	NE	0	NE
Spy49_0473	-	465417	465740	223	0.00025	NE	0	NE	0	NE
Spy49_0474	-	465785	466456	415	0	NE	0	NE	0	NE
Spy49_0475	-	466617	467864	238	0	NE	0	NE	0	NE
Spy49_0476	-	468092	468808	319	0.00025	NE	0	NE	0	NE
Spy49_0477c	-	468984	469943	267	0	NE	0	NE	0.00025	NE
Spy49_0478c	-	470313	471116	91	0	NE	0	NE	0	NE
Spy49_0479	-	471164	471439	72	0.0305	NE	0	NE	0.00025	NE
Spy49_0480	-	471459	471677	265	0	NE	0	NE	0	NE
Spy49_0481	-	471744	472541	274	0	NE	0	NE	0.00025	NE
Spy49_0482	-	472545	473369	516	1	E	1	E	1	E
Spy49_0483c	ftsY	473369	474919	455	0	NE	0	NE	0	NE
Spy49_0484	-	474973	476340	280	0.00025	NE	0	NE	0.00025	NE
Spy49_0485	bglG	476668	477510	620	0	NE	0	NE	0.00025	NE
Spy49_0487	-	477512	479374	474	0	NE	0	NE	0	NE
Spy49_0488c	bglA	479393	480817	271	0	NE	0	NE	0	NE
Spy49_0489c	-	480916	481731	300	0	NE	0	NE	0	NE
Spy49_0490	-	481731	482633	710	0.00025	NE	0	NE	0.00025	NE
Spy49_0491	-	483170	485302	160	0	NE	0	NE	0	NE
Spy49_0493	-	485244	485726	90	0	NE	0	NE	0	NE
Spy49_0494	-	485790	486062	330	0.00025	NE	1	E	1	E
Spy49_0495	ptsK	486367	487359	259	0.00025	NE	0	NE	0.00025	NE
Spy49_0496	lgt	487356	488135	135	0.00025	NE	0	NE	0	NE
Spy49_0497	-	488157	488564	142	0	NE	0	NE	0	NE
Spy49_0498	-	488557	488985	89	0	NE	0	NE	0	NE
Spy49_0499	-	489044	489313	308	0.00025	NE	0	NE	0	NE
Spy49_0500	-	489631	490557	428	0	NE	0	NE	0	NE
Spy49_0501	-	490659	491945	75	0	NE	0	NE	0	NE
Spy49_0502c	-	492146	492373	46	-1	S	-1	S	-1	S
Spy49_0503c	-	492470	492610	497	1	E	1	E	1	E
Spy49_0504	lysS	492746	494239	300	0.00025	NE	0	NE	0.00025	NE
Spy49_0505c	-	494413	495315	207	0	NE	0	NE	0	NE
Spy49_0506c	-	495423	496046	159	0	NE	0	NE	0	NE
Spy49_0507c	-	496387	496866	187	0	NE	0	NE	0	NE

Spy49_0508c	-	496957	497520	282	0	NE	0	NE	0	NE
Spy49_0509	-	497789	498637	167	0	NE	0	NE	0.00025	NE
Spy49_0510	-	498962	499465	128	0	NE	0	NE	0	NE
Spy49_0511c	-	499449	499835	159	0.00025	NE	0	NE	0.00025	NE
Spy49_0512c	-	499881	500360	599	0	NE	0	NE	0	NE
Spy49_0513	pepF	500353	502152	932	0	NE	0	NE	0	NE
Spy49_0514	ppc	502311	505109	302	1	E	1	E	1	E
Spy49_0515	ftsW	505279	506187	398	1	E	1	E	0.9905	U
Spy49_0516	tufA	506912	508108	252	0.95575	U	1	E	1	E
Spy49_0517c	tpiA	508349	509107	411	1	E	1	E	1	E
Spy49_0518c	murN	509207	510442	408	1	E	1	E	1	E
Spy49_0519c	murM.1	510429	511655	269	0.00025	NE	0	NE	0.00025	NE
Spy49_0520c	-	511655	512464	433	0	NE	0	NE	0	NE
Spy49_0521	-	512920	514221	128	0	NE	0	NE	0	NE
Spy49_0522	-	514303	514689	893	0.9995	E	1	E	1	E
Spy49_0523c	pacL	514920	517601	331	0.00025	NE	0	NE	0	NE
Spy49_0524c	regR	517687	518682	635	0.00025	NE	0	NE	0	NE
Spy49_0525c	-	518746	520653	273	0	NE	0	NE	0.44825	U
Spy49_0526c	agaD	520740	521561	260	0.00025	NE	0	NE	0.00025	NE
Spy49_0527c	agaW	521548	522330	162	0.00025	NE	0	NE	0	NE
Spy49_0528c	agaV	522349	522837	399	0	NE	0	NE	0.00025	NE
Spy49_0529c	ugl	522873	524072	145	0	NE	0	NE	0	NE
Spy49_0530	agaF	524072	524509	264	0	NE	0	NE	0	NE
Spy49_0531	-	524844	525638	213	0	NE	0	NE	0	NE
Spy49_0532	-	525663	526304	333	0	NE	0	NE	0	NE
Spy49_0533	kdgK	526333	527334	211	0	NE	0	NE	0	NE
Spy49_0534	kdgA	527339	527974	216	0.014	NE	0	NE	0.00025	NE
Spy49_0535	-	528272	528922	374	0	NE	0	NE	0	NE
Spy49_0536	-	529618	530742	326	1	E	1	E	0.99175	E
Spy49_0537	prfB	530928	531908	230	0.00075	NE	0.00025	NE	0.27175	U
Spy49_0538	ftsE	531927	532619	309	0.88575	U	1	E	1	E
Spy49_0539c	ftsX	532612	533541	192	0.00025	NE	0	NE	0	NE
Spy49_0540	-	533851	534429	254	0.00025	NE	0	NE	0	NE
Spy49_0541	-	534718	535482	833	0.00025	NE	0	NE	0	NE
Spy49_0543	dinG	535590	538091	397	0	NE	0	NE	0	NE
Spy49_0544	aspC	538426	539619	448	1	E	1	E	1	E
Spy49_0545	asnC	539640	540986	296	1	E	1	E	1	E
Spy49_0546	-	541401	542291	325	0	NE	0	NE	0	NE
Spy49_0547	-	542288	543265	303	0.001	NE	0	NE	0.0035	NE
Spy49_0548	-	543262	544173	465	0	NE	0	NE	0.00025	NE
Spy49_0549	pepD	544306	545703	515	0	NE	0	NE	0.0005	NE
Spy49_0550	adcA	545855	547402	240	0	NE	0	NE	0	NE
Spy49_0551	-	547552	548274	399	0.00025	NE	0	NE	0	NE
Spy49_0552c	agaS	548294	549493	86	0.95875	U	0	NE	0	NE
Spy49_0554c	rpmE2	549590	549850	313	0.00025	NE	0	NE	0.00025	NE
Spy49_0555	-	549965	550906	149	0.00025	NE	0	NE	0	NE
Spy49_0556	flaV	551300	551749	94	0.00025	NE	0	NE	0.0495	U
Spy49_0557	-	551925	552209	420	0.032	NE	0.06425	NE	0.99925	E
Spy49_0558	-	552202	553464	115	0.716	U	1	E	0.0895	U
Spy49_0559	rplS	553579	553926	189	0	NE	0	NE	0	NE
Spy49_0560	-	554947	555516	650	1	E	1	E	1	E
Spy49_0561	gyrB	555517	557469	574	1	E	1	E	1	E
Spy49_0562c	ezrA	557837	559561	152	0	NE	0	NE	0	NE
Spy49_0563	-	559693	560151	435	0.99875	E	1	E	1	E
Spy49_0564c	eno	560384	561691	191	0.00025	NE	0	NE	0	NE
Spy49_0565c	-	562280	562855	53	-1	S	-1	S	-1	S
Spy49_0566c	-	562941	563102	513	0	NE	0	NE	0	NE
Spy49_0567	-	563464	565005	1130	0	NE	0	NE	0.2585	U
Spy49_0568	-	565552	568944	53	0.0315	NE	0	NE	0	NE
Spy49_0569	sagA	569809	569970	316	0.00025	NE	0	NE	0	NE
Spy49_0570	sagB	570192	571142	352	0.00025	NE	0	NE	0	NE
Spy49_0572	sagC	571139	572197	452	0	NE	0	NE	0.00025	NE
Spy49_0573	sagD	572217	573575	223	0	NE	0	NE	0	NE
Spy49_0574	sagE	573550	574221	227	0.00025	NE	0	NE	0.00025	NE
Spy49_0575	sagF	574218	574901	307	0.9135	U	0	NE	0.99975	E
Spy49_0576	sagG	574924	575847	375	0	NE	0.99625	E	0.73725	U
Spy49_0577	sagH	575856	576983	372	0.00025	NE	0.00025	NE	0.99375	E
Spy49_0578	sagI	576980	578098	910	0.0005	NE	0	NE	0	NE

Spy49_0579	-	578669	581401	166	0	NE	0	NE	0	NE
Spy49_0580	-	581679	582179	652	0.99175	U	1	E	1	E
Spy49_0581	ligA	582373	584331	340	1	E	1	E	0.99975	E
Spy49_0582	-	584345	585367	65	0.931	U	1	E	0.93375	U
Spy49_0583	-	585762	585959	238	1	E	1	E	1	E
Spy49_0584	-	585994	586710	164	0.99825	E	0.969	U	0.994	E
Spy49_0585	-	586728	587222	178	1	E	1	E	1	E
Spy49_0586	-	587222	587758	502	1	E	1	E	1	E
Spy49_0587	-	587774	589282	291	1	E	1	E	1	E
Spy49_0588	-	589298	590173	468	1	E	1	E	1	E
Spy49_0589	-	590335	591741	138	0.97825	U	0	NE	0.9285	U
Spy49_0590	atpC	591754	592170	85	0.00025	NE	0	NE	0	NE
Spy49_0591	-	592436	592693	423	0.00025	NE	0	NE	0.00025	NE
Spy49_0592	murA	592758	594029	62	0.001	NE	0	NE	0	NE
	-	594033	594221		0	NE	0	NE	0	NE
	pheS	595077	596120		0	NE	0	NE	0.00025	NE
Spy49_0596	pheT	596330	598735	347	1	E	1	E	1	E
Spy49_0597	-	598845	599222	801	1	E	1	E	1	E
Spy49_0598	-	599215	599601	125	0	NE	0	NE	0	NE
Spy49_0599	-	599674	600750	128	0	NE	0	NE	0.00025	NE
Spy49_0600	-	600760	601428	358	0.00025	NE	0	NE	0	NE
Spy49_0601	-	601532	602449	222	0	NE	0	NE	0.0005	NE
Spy49_0602c	rexB	602600	605815	305	0	NE	0	NE	0	NE
Spy49_0603	rexA	605776	609444	1071	0.006	NE	0	NE	1	E
Spy49_0604	-	609584	610204	1222	0.93275	U	1	E	1	E
Spy49_0605	rpsU	610543	610719	206	0	NE	0	NE	0	NE
Spy49_0606	mscL	610847	611209	58	0.955	U	0.9985	E	0.26675	U
Spy49_0607c	dnaG	611339	613153	120	0	NE	0	NE	0	NE
Spy49_0608	rpoD	613162	614271	604	0.999	E	1	E	1	E
Spy49_0609	-	614507	614845	369	1	E	1	E	1	E
Spy49_0610	rmlD	614983	615837	112	0.00025	NE	0	NE	0	NE
Spy49_0611	rgpAc	615956	617110	284	0.99925	E	1	E	1	E
Spy49_0612	rgpBc	617100	618032	384	0.2065	U	1	E	1	E
Spy49_0613	rgpCc	618035	618838	310	0.0595	U	1	E	1	E
Spy49_0614	rgpDc	618838	620058	267	1	E	1	E	1	E
Spy49_0615	rgpEc	620069	621076	406	1	E	1	E	1	E
Spy49_0617	rgpFc	621073	622818	335	0.99975	E	1	E	1	E
Spy49_0618	-	622815	625289	581	0.99875	E	1	E	1	E
Spy49_0619	-	625468	626163	824	0.00025	NE	0	NE	0	NE
Spy49_0620	-	626165	626506	231	0	NE	0	NE	0	NE
Spy49_0621	-	626499	627785	113	0.00025	NE	0	NE	0	NE
Spy49_0622	-	627766	629262	428	0.00025	NE	0.99575	E	1	E
Spy49_0623	pepT	629356	630579	498	0.00375	NE	1	E	1	E
Spy49_0624	-	630620	631108	407	0.00025	NE	0	NE	0.00025	NE
Spy49_0625	-	631095	631292	162	0	NE	0	NE	0	NE
Spy49_0626c	-	631341	631817	65	0	NE	0	NE	0	NE
Spy49_0627	cmk	631832	632512	158	0	NE	0	NE	0.00025	NE
Spy49_0628	infC	632674	633204	226	0.00025	NE	0	NE	0.16625	U
Spy49_0629	rpmI	633246	633443	176	0.9975	E	1	E	0.99225	E
Spy49_0630	rplT	633502	633861	65	0	NE	0	NE	0	NE
Spy49_0631	-	634152	636362	119	0.02	NE	0.9965	E	0.00075	NE
Spy49_0632c	-	636470	637633	736	1	E	1	E	1	E
Spy49_0633	aroD	637630	638316	387	0.00025	NE	0	NE	0.00025	NE
Spy49_0634	aroF	638410	639576	228	0.00025	NE	0	NE	0	NE
Spy49_0635	-	639637	639978	388	0	NE	0	NE	0	NE
Spy49_0636	gor	640200	641552	113	0	NE	0	NE	0	NE
Spy49_0637	folC.2	641640	642908	450	0	NE	0	NE	0.00025	NE
Spy49_0638c	-	642938	643378	422	0	NE	0	NE	0	NE
Spy49_0639c	nifS	643613	644755	146	0	NE	0	NE	0	NE
Spy49_0640	thiI	644767	645981	380	0	NE	0	NE	0.00025	NE
Spy49_0641	-	646019	647287	404	0	NE	0	NE	0	NE
Spy49_0642	rplU	647526	647840	422	0.00025	NE	0	NE	0.0005	NE
Spy49_0643	-	647852	648178	104	0.9925	U	0.00075	NE	0.9885	U
Spy49_0644	rpmA	648206	648499	108	0.00275	NE	1	E	0.8775	U
Spy49_0645	-	648847	649761	97	0.057	U	0	NE	0.923	U
Spy49_0646	lsp	649758	650216	304	0.00025	NE	0	NE	0.00025	NE
Spy49_0647	-	650206	651096	152	0	NE	0	NE	0.00025	NE
Spy49_0648	pyrR	651492	652013	296	0	NE	0	NE	0	NE

Spy49_0650	pyrP	652029	653288	173	0	NE	0	NE	0	NE
Spy49_0651	pyrB	653349	654284	419	0	NE	0	NE	0	NE
Spy49_0652	carA	654328	655410	311	0	NE	0	NE	0	NE
Spy49_0653	carB	655656	658832	360	0	NE	0	NE	0.52125	U
Spy49_0654	-	659041	660312	1058	0	NE	0	NE	0.00025	NE
Spy49_0655	-	660312	661022	423	0	NE	0	NE	0.00025	NE
Spy49_0656	-	661034	662254	236	0	NE	0	NE	0.00025	NE
Spy49_0657	-	662508	664241	406	0.00025	NE	0	NE	0.00025	NE
Spy49_0658	rpsP	664368	664640	577	0.39425	U	0	NE	0.997	E
Spy49_0659	-	664650	664889	90	0.999	E	1	E	0.99825	E
Spy49_0660	-	665821	668847	79	0.9955	E	0	NE	0.9675	U
Spy49_0661	-	668867	669268	1008	0.00025	NE	0	NE	0.00025	NE
Spy49_0662	czcD	669448	670323	133	0.00025	NE	0	NE	0.00025	NE
Spy49_0663c	-	670459	670980	291	0	NE	0	NE	0	NE
Spy49_0664	rimM	671195	671713	173	0	NE	0	NE	0.00025	NE
Spy49_0665	trmD	671700	672434	172	0.2	U	0.00025	NE	0.00025	NE
Spy49_0667	-	672434	673426	244	0.998	E	1	E	1	E
Spy49_0668	-	673603	674661	330	0.00025	NE	0	NE	0	NE
Spy49_0669	apbA	674674	675597	352	0.00025	NE	0	NE	0	NE
Spy49_0671	fruR	675853	676566	307	0	NE	0	NE	0	NE
Spy49_0672	fruK	676563	677474	237	0	NE	0	NE	0	NE
Spy49_0673	fruA	677471	679417	303	0	NE	0	NE	0	NE
Spy49_0674	-	679516	680109	648	0	NE	0	NE	0.00025	NE
Spy49_0675	-	680261	680968	197	0.00025	NE	0	NE	0.00025	NE
Spy49_0676	-	681025	681243	235	0	NE	0	NE	0	NE
Spy49_0677c	mac	681925	682944	72	0	NE	0	NE	0.00025	NE
	-	683850	684230		0	NE	0	NE	0.00075	NE
Spy49_0679	-	684230	685078	339	0	NE	0	NE	0.00025	NE
Spy49_0680	papS	685252	686412	126	0	NE	0	NE	0.0005	NE
Spy49_0681	-	686409	688286	282	0.00025	NE	0	NE	0	NE
Spy49_0682	-	689180	689590	386	1	E	1	E	1	E
Spy49_0683	-	689672	691684	625	0	NE	0	NE	0.00025	NE
Spy49_0684	-	691904	692554	136	0	NE	0	NE	0	NE
Spy49_0686c	-	692557	693225	670	0	NE	0	NE	0.0005	NE
Spy49_0687	-	693234	694466	216	0.00025	NE	0	NE	0.00025	NE
Spy49_0688	mvaK1	694760	695638	222	0.00025	NE	0	NE	0.00025	NE
Spy49_0689	mvaD	695620	696564	410	0	NE	0	NE	0	NE
Spy49_0690	mvaK2	696557	697570	292	0.0005	NE	1	E	1	E
Spy49_0691	-	697557	698546	314	0.977	U	1	E	1	E
Spy49_0692	mvaS1	698776	700053	337	1	E	1	E	1	E
Spy49_0693	mvaS2	700040	701215	329	0.6545	U	1	E	1	E
Spy49_0694c	thyA	701424	702263	425	0.9995	E	1	E	1	E
Spy49_0695c	dyr	702343	702840	391	1	E	1	E	1	E
Spy49_0696	-	702860	703060	279	0.00025	NE	0	NE	0.99975	E
Spy49_0697	clpX	703161	704390	165	0.00025	NE	1	E	1	E
Spy49_0698	engB	704400	704999	66	0	NE	0	NE	0.00525	NE
Spy49_0700	-	705147	705890	409	0.306	U	0	NE	0.80075	U
Spy49_0701	clpL	705948	708047	199	0.0005	NE	1	E	0.9985	E
Spy49_0702	rpiA	708425	709108	247	0	NE	0	NE	0	NE
Spy49_0703c	deoB	709185	710396	699	0.00025	NE	0	NE	0.00025	NE
Spy49_0704	arsC	710415	710855	227	0.00025	NE	0	NE	0.98625	U
Spy49_0705	punA	710839	711648	403	0	NE	0	NE	0	NE
Spy49_0706	deoD	712176	712946	146	0	NE	0	NE	0	NE
Spy49_0707	-	712939	713727	269	0	NE	0	NE	0	NE
Spy49_0708	cpsY	713807	714712	256	0	NE	0	NE	0	NE
Spy49_0709	-	714934	715470	262	0	NE	0	NE	0	NE
Spy49_0710c	pyrF	715747	716439	301	0	NE	0	NE	0.00025	NE
Spy49_0711	pyrE	716497	717126	178	0	NE	0	NE	0	NE
Spy49_0712	amiC	717323	718777	230	0	NE	0	NE	0	NE
Spy49_0713	-	718895	719758	209	0	NE	0	NE	0.00025	NE
Spy49_0714	-	719787	720437	484	0	NE	0	NE	0.002	NE
Spy49_0715	ung	720570	721223	287	0.00025	NE	0	NE	0	NE
Spy49_0716	pyrC	721355	722623	216	0.00025	NE	0	NE	0	NE
Spy49_0717	-	722681	723322	217	0	NE	0	NE	0	NE
Spy49_0718	parE	723457	725406	422	0	NE	0	NE	0.00025	NE
Spy49_0719c	parC	725497	727956	213	0.9945	E	1	E	1	E
Spy49_0720	bcaT	728079	729101	649	0.99975	E	1	E	1	E
Spy49_0721	-	729165	729395	819	1	E	1	E	1	E

Spy49_0722	rpsA	729786	730991	340	0	NE	0	NE	0	NE
Spy49_0723	-	731493	731795	76	0.96775	U	0	NE	0.058	U
Spy49_0724	-	731996	732982	401	0.971	U	1	E	1	E
Spy49_0725c	-	733293	733700	100	0	NE	0	NE	0	NE
Spy49_0726	-	733950	734858	328	0.00025	NE	0	NE	0	NE
Spy49_0727	-	734976	735152	135	0	NE	0	NE	0.00025	NE
Spy49_0728	miaA	735286	736185	302	0	NE	0	NE	0	NE
Spy49_0729c	hflX	736258	737496	58	0	NE	0	NE	0	NE
Spy49_0730	-	737489	738124	299	0.00025	NE	0	NE	0.0065	NE
Spy49_0731	-	738139	739068	412	0	NE	0	NE	0	NE
Spy49_0732	-	739228	739833	211	0	NE	0	NE	0.00025	NE
Spy49_0733	recJ	739830	742040	309	1	E	1	E	1	E
Spy49_0734	apt	742190	742708	201	0	NE	0	NE	0	NE
Spy49_0735	dnaD	742789	743472	736	0.00025	NE	0	NE	0	NE
Spy49_0736	nth	743469	744125	172	0	NE	0	NE	0.00025	NE
Spy49_0737	-	744197	744883	227	1	E	1	E	1	E
Spy49_0738	-	744873	745661	218	0	NE	0	NE	0	NE
Spy49_0739	-	745701	746825	228	0.996	E	1	E	0.997	E
Spy49_0740	rmlA	746864	747733	262	0	NE	0	NE	0.01375	NE
Spy49_0741	rmlC	747733	748326	374	0.00025	NE	0	NE	0.00025	NE
Spy49_0742	rmlB	748570	749610	289	1	E	1	E	1	E
Spy49_0744	-	749693	750832	197	0.99125	U	1	E	0.97625	U
Spy49_0745	-	750960	751226	346	0.99925	E	1	E	0.99975	E
Spy49_0746c	-	751238	751624	379	0.00025	NE	0	NE	0.00025	NE
Spy49_0747c	-	751627	751977	88	0	NE	0	NE	0.00075	NE
Spy49_0748c	-	752273	752443	128	0	NE	0	NE	0	NE
Spy49_0749c	-	752469	753257	116	0.0185	NE	0.0155	NE	0.99875	E
Spy49_0750	-	753308	753499	56	-1	S	-1	S	-1	S
Spy49_0751c	-	753578	753889	262	0.00025	NE	0	NE	0.935	U
Spy49_0752	-	753891	754061	63	0.00225	NE	0.895	U	0.36025	U
Spy49_0753	-	754054	754257	103	0.00825	NE	0.005	NE	0.42875	U
Spy49_0754	-	754254	754640	56	0.541	U	0	NE	0.93575	U
Spy49_0755	-	754786	754989	67	0.02575	NE	0.00025	NE	0.90725	U
Spy49_0756	-	755077	755376	128	0	NE	0	NE	0.00025	NE
Spy49_0757	-	755376	756533	67	0.9885	U	0	NE	0.00325	NE
Spy49_0759	-	756548	757105	99	0.00025	NE	0.0005	NE	0.9705	U
Spy49_0760	-	757148	759070	385	0	NE	0.00025	NE	0.00025	NE
Spy49_0761	-	759075	761459	185	0.00025	NE	0	NE	0.00025	NE
Spy49_0762	-	761827	762120	640	0	NE	0	NE	0.73875	U
Spy49_0763	-	762117	763439	794	0	NE	0	NE	0.988	U
Spy49_0764	-	763603	763875	97	0.402	U	0.9875	U	0.3905	U
Spy49_0765	-	764008	764424	440	0	NE	0	NE	0.9845	U
Spy49_0766	-	764496	764966	90	0	NE	0	NE	0	NE
Spy49_0767	-	764956	766233	138	0	NE	0	NE	0.00025	NE
Spy49_0768	-	766249	767781	156	0	NE	0	NE	0.00025	NE
Spy49_0769	-	767747	769189	425	0	NE	0	NE	0	NE
Spy49_0770	-	769410	769802	510	0	NE	0	NE	0.00025	NE
Spy49_0771	-	769845	770414	480	0	NE	0	NE	0	NE
Spy49_0772	-	770427	771314	130	0.00025	NE	0	NE	0.00025	NE
Spy49_0773	-	771326	771682	189	0.00025	NE	0	NE	0.127	U
Spy49_0774	-	771693	771971	295	0	NE	0	NE	0.00025	NE
Spy49_0775	-	771968	772312	118	0	NE	0	NE	0	NE
Spy49_0776	-	772316	772675	92	0	NE	0	NE	0.00025	NE
Spy49_0777	-	772687	773286	114	0	NE	0	NE	0.00025	NE
Spy49_0778	-	773393	773794	119	0	NE	0	NE	0.9005	U
Spy49_0779	-	773869	774102	199	0	NE	0	NE	1	E
Spy49_0780	-	774117	778499	133	0.0195	NE	0	NE	0.19775	U
Spy49_0781	-	778511	779353	77	0	NE	0	NE	0.00025	NE
Spy49_0782	-	779363	781342	1460	0.001	NE	0	NE	0.049	U
Spy49_0783	-	781339	783582	280	0	NE	0	NE	0.0005	NE
Spy49_0784	-	783594	783755	659	0.00025	NE	0	NE	0.26	U
Spy49_0785	-	783758	784369	747	0.00025	NE	0	NE	0.86775	U
Spy49_0786	-	784379	784834	53	-1	S	-1	S	-1	S
Spy49_0788	-	784952	785704	203	0	NE	0	NE	0	NE
Spy49_0789	-	785870	786157	151	0	NE	0	NE	0	NE
Spy49_0790	speH	786363	787073	250	0	NE	0	NE	0	NE
Spy49_0791	mutX	787677	788153	95	0	NE	0	NE	0	NE
Spy49_0792	-	788211	789392	236	0	NE	0	NE	0	NE

Spy49_0793	-	789382	790629	158	0	NE	0	NE	0	NE
Spy49_0794	-	790688	792340	393	0.00025	NE	0	NE	0.00025	NE
Spy49_0795	-	792694	793692	415	0	NE	0	NE	0	NE
Spy49_0796c	-	793643	793861	550	0	NE	0	NE	0.00025	NE
Spy49_0797	-	794037	794906	332	0.00025	NE	0	NE	0.00025	NE
Spy49_0798	-	794903	795661	72	-1	S	-1	S	-1	S
Spy49_0799	-	795865	797526	289	0	NE	0	NE	0	NE
Spy49_0800	estA	797659	798444	252	0	NE	0	NE	0	NE
Spy49_0801	-	798474	798800	553	0.99975	E	1	E	1	E
Spy49_0803	-	798846	800753	261	0	NE	0	NE	0.00025	NE
Spy49_0804	acoA	801026	802006	108	0	NE	0	NE	0.00025	NE
Spy49_0805	acoB	802062	803063	635	0.0165	NE	0	NE	0	NE
Spy49_0807	acoC	803248	804657	326	0.00025	NE	0	NE	0.00025	NE
Spy49_0808	acoL	804984	806747	333	0	NE	0	NE	0	NE
Spy49_0809	hylA	807422	809839	469	0	NE	0	NE	0	NE
Spy49_0810	-	810072	811061	587	0	NE	0	NE	0.00025	NE
Spy49_0811c	cobQ	811170	811961	805	0	NE	0	NE	0	NE
Spy49_0812	murC2	811961	813304	329	0	NE	0	NE	0.00025	NE
Spy49_0813c	-	813406	814257	263	0.9995	E	1	E	1	E
Spy49_0814c	-	814254	815210	447	0.99975	E	1	E	1	E
Spy49_0815	glmM	815264	816616	283	0.8475	U	1	E	1	E
Spy49_0816	-	816739	817380	318	0	NE	0	NE	0.00025	NE
Spy49_0817	hemN	817377	818573	450	1	E	1	E	1	E
Spy49_0818	-	818583	819335	213	0.00025	NE	0	NE	0.00025	NE
Spy49_0819	-	819335	820099	398	0	NE	0	NE	0.00025	NE
Spy49_0820	-	820096	820731	250	0.00025	NE	1	E	1	E
Spy49_0821	-	821210	825316	254	0	NE	0	NE	0.00025	NE
Spy49_0822	-	825316	826185	211	0.00025	NE	0	NE	0.00025	NE
Spy49_0823	-	826182	826523	1368	0.00025	NE	0	NE	0	NE
Spy49_0825	-	826513	827175	289	0.00025	NE	0	NE	0.00025	NE
Spy49_0826	-	827952	828122	113	0	NE	0	NE	0	NE
Spy49_0827	lepA-2	828223	830055	220	0	NE	0	NE	0.00025	NE
Spy49_0828	sclB	830338	831435	56	0	NE	0	NE	0	NE
Spy49_0829	csrA	831620	832057	610	0.00025	NE	0	NE	0	NE
Spy49_0830	-	832187	833206	365	0.05675	U	0	NE	0	NE
Spy49_0831	-	833413	833838	145	0	NE	0	NE	0.00025	NE
Spy49_0832	-	833865	834356	339	0	NE	0	NE	0.00025	NE
Spy49_0833	-	834449	835183	141	0	NE	0	NE	0	NE
Spy49_0834	-	835180	836019	163	0	NE	0	NE	0.81775	U
Spy49_0835	-	836144	837793	244	0.00025	NE	0	NE	0.00025	NE
Spy49_0836	-	837797	838585	279	0	NE	0	NE	0	NE
Spy49_0837	-	838579	839625	549	0	NE	0	NE	0.00025	NE
Spy49_0839	-	839741	841138	262	0.00025	NE	0	NE	0.00025	NE
Spy49_0840	uvrC	841239	843035	348	0	NE	0	NE	0	NE
Spy49_0841	-	843221	843823	465	0	NE	0	NE	0	NE
Spy49_0842	-	843948	845357	598	0.00025	NE	0	NE	0	NE
Spy49_0843	trmE	845425	846801	200	0	NE	0	NE	0	NE
Spy49_0844	rplJ	847136	847636	469	0	NE	0	NE	0	NE
Spy49_0845c	-	847701	847799	458	0.00025	NE	1	E	0.99575	E
Spy49_0846	rplL	847874	848065	166	0.00025	NE	0	NE	0.00025	NE
Spy49_0847	dacA1	848292	849605	32	-1	S	-1	S	-1	S
Spy49_0848	-	849658	850620	63	0.9105	U	0.0035	NE	0.7455	U
Spy49_0849	folC1	850952	852229	437	0.00025	NE	0	NE	0.00025	NE
Spy49_0850c	folE	852264	852848	320	0	NE	0	NE	0	NE
Spy49_0851	folP	852857	853657	425	0.00025	NE	1	E	1	E
Spy49_0852	folQ	853664	854023	194	0	NE	0	NE	0	NE
Spy49_0853	folK	854020	854520	266	0.00025	NE	0	NE	0.43975	U
Spy49_0854	murB	854670	855557	119	0.00025	NE	0	NE	0.00025	NE
Spy49_0855	potA	855603	856757	166	0	NE	0	NE	0.0005	NE
Spy49_0856	potB	856741	857535	295	1	E	1	E	0.99975	E
Spy49_0857	potC	857532	858308	384	0.00025	NE	0	NE	0	NE
Spy49_0858	potD	858301	859374	264	0	NE	0	NE	0	NE
Spy49_0859	-	859429	860094	258	0	NE	0	NE	0	NE
Spy49_0860	-	860075	861616	357	0	NE	0	NE	0	NE
Spy49_0861c	malP	861777	863108	221	0.00025	NE	0	NE	0	NE
Spy49_0862c	-	863140	864306	513	0	NE	0	NE	0.00025	NE
Spy49_0863	-	864388	865437	443	0.00025	NE	0	NE	0.00025	NE
Spy49_0864	aphA	865673	866404	388	0.00025	NE	0	NE	0	NE

Spy49_0865c	-	866582	868114	349	0	NE	0	NE	0	NE
Spy49_0866	-	868277	868891	243	0	NE	0	NE	0	NE
Spy49_0867	-	869071	870180	510	0	NE	0	NE	0.00025	NE
Spy49_0868	radC	870247	870927	204	0.00025	NE	0	NE	0.00025	NE
Spy49_0869	-	870929	871624	369	0	NE	0	NE	0	NE
Spy49_0870	-	871634	872278	226	0	NE	0	NE	0.00025	NE
Spy49_0871c	-	872530	872877	231	0	NE	0	NE	0.00025	NE
Spy49_0872c	-	872867	873994	214	0	NE	0	NE	0	NE
Spy49_0873c	prs	873991	874971	115	0.00025	NE	0	NE	0	NE
Spy49_0874c	-	875111	875689	375	1	E	1	E	1	E
Spy49_0875c	-	875777	876448	326	0.00025	NE	0	NE	0.00025	NE
Spy49_0876c	ppnK	876423	877259	192	0	NE	0	NE	0.912	U
Spy49_0877	rluD	877256	878161	223	0.00025	NE	0	NE	0.00025	NE
Spy49_0878	eutD	878165	879160	278	1	E	1	E	1	E
Spy49_0879	-	879286	880041	301	0	NE	0	NE	0	NE
Spy49_0880	-	880235	880924	331	0	NE	0	NE	0	NE
Spy49_0881	proV	881350	882078	251	0	NE	0	NE	0.0005	NE
Spy49_0882c	proX	882071	883597	229	0	NE	0	NE	0	NE
Spy49_0883	-	883875	884858	242	0.00025	NE	0	NE	0.00025	NE
Spy49_0884	-	884895	885152	508	0	NE	0	NE	0.00025	NE
Spy49_0885	xpt	885163	885744	327	0	NE	0	NE	0	NE
Spy49_0886c	-	885744	887027	85	0	NE	0	NE	0	NE
Spy49_0887	-	887091	888029	193	0.00025	NE	0	NE	0	NE
Spy49_0888	-	888082	888267	427	0	NE	0	NE	0	NE
Spy49_0891c	tdk	888405	888974	312	0	NE	0	NE	0	NE
Spy49_0892c	prfA	889009	890088	61	0	NE	0	NE	0	NE
Spy49_0893	hemK	890088	890927	189	0	NE	0	NE	0	NE
Spy49_0894	-	890911	891501	359	1	E	1	E	1	E
Spy49_0895	-	891519	891971	279	0.00025	NE	0	NE	0.06075	U
Spy49_0896	glyA	891961	893217	196	0.8395	U	1	E	1	E
Spy49_0897	-	893224	894201	150	0.00025	NE	0	NE	0	NE
Spy49_0898	-	894202	894801	418	0	NE	0	NE	0	NE
Spy49_0899	-	894811	896535	325	0	NE	0	NE	0.0595	U
Spy49_0900	-	896532	898247	199	0.00025	NE	0	NE	0.00025	NE
Spy49_0901	nox1	898500	899870	574	0	NE	0	NE	0.064	U
Spy49_0902	ldh	900029	901012	571	0.00025	NE	0	NE	0.00025	NE
Spy49_0903	gyrA	901203	903689	456	0	NE	0	NE	0	NE
Spy49_0904c	-	903709	904458	327	0.00025	NE	0.00425	NE	1	E
Spy49_0905	-	904538	904954	828	1	E	1	E	1	E
Spy49_0906	-	905603	906007	249	0.00025	NE	0.80075	U	0.999	E
Spy49_0907	-	906057	906968	138	0.00025	NE	0	NE	0.99625	E
Spy49_0908	hlyIII	907220	907735	134	0	NE	0	NE	0	NE
Spy49_0909c	-	907732	908172	303	0.00025	NE	0	NE	0.00025	NE
Spy49_0910c	rbgA	908391	909239	171	0.99975	E	1	E	1	E
Spy49_0911c	rnhB	909229	910020	146	0.00875	NE	0	NE	0	NE
Spy49_0912	smf	910085	910921	282	0.916	U	1	E	1	E
Spy49_0913	topA	911028	913157	263	0	NE	0	NE	0	NE
Spy49_0914	-	913233	913715	278	0	NE	0	NE	0	NE
Spy49_0915	-	913722	913844	709	1	E	1	E	1	E
Spy49_0916c	-	914130	914696	160	0	NE	0	NE	0	NE
Spy49_0917c	-	914711	915703	40	-1	S	-1	S	-1	S
Spy49_0918	-	915808	916473	188	0	NE	0	NE	0	NE
Spy49_0919	-	916466	917182	330	0.00025	NE	0	NE	0.00025	NE
Spy49_0920	gid	917333	918679	221	0.998	E	1	E	1	E
Spy49_0921	-	918856	920238	238	0	NE	0	NE	0	NE
Spy49_0922	-	920281	920595	448	0	NE	0	NE	0.9835	U
Spy49_0923	-	920595	920945	460	0.00025	NE	0	NE	0.00025	NE
Spy49_0924	-	920955	922085	104	0	NE	0	NE	0.00125	NE
Spy49_0925	citG	922286	923170	116	0.00025	NE	0	NE	0	NE
Spy49_0926	-	923163	923858	376	0	NE	0	NE	0.7035	U
Spy49_0927c	-	924016	925422	294	0	NE	0	NE	0	NE
Spy49_0928c	-	925799	926122	231	0.00025	NE	0	NE	0	NE
Spy49_0929c	-	926611	927732	468	0	NE	0	NE	0	NE
Spy49_0930	citD	927920	928228	107	0	NE	0	NE	0	NE
Spy49_0933	citE	928216	929103	373	0.00175	NE	0	NE	0	NE
Spy49_0934	citF	929106	930638	102	0.00025	NE	0	NE	0	NE
Spy49_0936	citX	930535	931209	295	0.115	U	0	NE	0	NE
Spy49_0937	oadA	931224	932618	510	0	NE	0	NE	0	NE

Spy49_0938	citC	932729	933781	224	0.00025	NE	0	NE	0	NE
Spy49_0939	-	933968	934351	464	0.00025	NE	0	NE	0	NE
Spy49_0940c	xerS	935045	936115	350	0	NE	0	NE	0	NE
Spy49_0942c	-	936585	937283	127	0	NE	0	NE	0	NE
Spy49_0943c	ffh	937400	938962	356	0.0035	NE	0.00025	NE	0.9995	E
Spy49_0944	-	938977	939318	232	0.00025	NE	0	NE	0	NE
Spy49_0945c	-	939407	940105	520	1	E	1	E	1	E
Spy49_0946c	-	940188	941477	113	0.99725	E	0.02475	NE	0.00025	NE
Spy49_0947c	guaA	941789	943351	232	0.00025	NE	0	NE	0.00025	NE
Spy49_0948c	murM2	943393	944604	429	0	NE	0	NE	0	NE
Spy49_0949	-	944959	946524	520	0.2225	U	0	NE	0.99675	E
Spy49_0951c	-	946637	947194	403	0	NE	0	NE	0.00025	NE
Spy49_0952c	pdxK	947172	948038	521	0	NE	0	NE	0	NE
Spy49_0953c	-	948128	949396	185	0	NE	0	NE	0	NE
Spy49_0954c	-	949867	950223	288	0	NE	0	NE	0	NE
Spy49_0955	-	950545	952122	422	0	NE	0	NE	0.00025	NE
Spy49_0957c	fhs1	952207	953877	118	0	NE	0	NE	0	NE
Spy49_0958c	lplA	954005	955024	525	0.00025	NE	1	E	0.998	E
Spy49_0959c	-	955071	955952	556	0.97375	U	0.00975	NE	1	E
Spy49_0960c	-	955945	956757	339	0	NE	0	NE	0.00025	NE
Spy49_0961c	-	956750	957082	293	0.00025	NE	0	NE	0.00025	NE
Spy49_0962c	-	957124	958122	270	0.00025	NE	0	NE	0	NE
Spy49_0963c	-	958119	959318	110	0	NE	0	NE	0	NE
Spy49_0964c	-	959315	960151	332	0.00025	NE	0	NE	0	NE
Spy49_0965c	dpfB	960347	961039	399	0.00025	NE	0	NE	0	NE
Spy49_0966c	dfp	961032	961577	278	0	NE	0	NE	0	NE
Spy49_0967	-	961636	962205	230	0.00025	NE	1	E	0.99975	E
Spy49_0968	pgmA	962381	964099	181	0.99975	E	1	E	1	E
Spy49_0969	-	964312	965268	189	0.00025	NE	0	NE	1	E
Spy49_0970	-	965270	966334	572	0.998	E	1	E	1	E
Spy49_0971c	-	966327	967859	318	0	NE	0	NE	0	NE
Spy49_0972c	-	967998	969050	354	0	NE	0	NE	0	NE
Spy49_0973c	cdd	969144	969533	510	0	NE	0	NE	0	NE
Spy49_0974c	-	970186	970779	350	0.03775	NE	0	NE	0	NE
Spy49_0975c	coaA	971047	971967	129	0	NE	0	NE	0	NE
Spy49_0976c	rpsT	972036	972269	197	0	NE	0	NE	0	NE
Spy49_0977	ciaH	972394	973704	306	0.8445	U	1	E	1	E
Spy49_0978	ciaR	973697	974371	77	0.00275	NE	0	NE	0.9535	U
Spy49_0979c	pepN	974718	977255	436	0.00025	NE	0.00075	NE	0.9995	E
Spy49_0980c	phoU	977460	978113	224	0.00025	NE	1	E	1	E
Spy49_0981c	pstB	978182	978940	845	0.00025	NE	0	NE	0.00025	NE
Spy49_0982c	pstB2	978953	979756	217	0.00025	NE	0	NE	0.0005	NE
Spy49_0983c	pstA	979772	980659	252	0.99475	E	0	NE	0.00025	NE
Spy49_0984c	pstC	980649	981584	267	0.00025	NE	0	NE	0.99875	E
Spy49_0985c	pstS	981595	982461	295	0.00025	NE	0	NE	0.88725	U
Spy49_0986	-	982600	983910	311	0.99925	E	0.00025	NE	0.98675	U
Spy49_0987c	-	983913	984701	288	0.36625	U	0	NE	0.63625	U
Spy49_0988c	-	984691	984969	436	0.99975	E	1	E	1	E
Spy49_0989c	spxA	984971	985375	262	0	NE	0.00025	NE	0.00025	NE
Spy49_0990c	-	985418	986350	92	0.00025	NE	0	NE	0	NE
Spy49_0991c	truB	986340	987263	134	0	NE	0	NE	0.00025	NE
Spy49_0992c	-	987379	988719	310	0.992	U	1	E	1	E
Spy49_0993c	-	988816	989772	307	0	NE	0	NE	0	NE
Spy49_0994c	-	989783	990379	446	0.00025	NE	0	NE	0.00025	NE
Spy49_0995c	-	990471	993107	318	0	NE	0	NE	0.00025	NE
Spy49_0996c	-	993122	993823	198	0	NE	0	NE	0	NE
Spy49_0997c	-	993941	994483	878	0	NE	0	NE	0.00025	NE
Spy49_0998c	-	994638	995267	233	0	NE	0	NE	0.00025	NE
Spy49_0999	-	995430	995918	180	0	NE	0	NE	0.00025	NE
Spy49_1000c	-	995929	996129	209	0.00025	NE	0	NE	0.00025	NE
Spy49_1001c	-	996170	996709	162	0	NE	0	NE	0	NE
Spy49_1002c	-	996722	996910	66	0	NE	0	NE	0	NE
Spy49_1003c	-	996921	997508	179	0.00025	NE	0	NE	0	NE
Spy49_1004c	-	997569	997817	62	0.16625	U	0.0005	NE	0.008	NE
Spy49_1005c	pcrA	998180	1000498	195	0.00025	NE	0	NE	0	NE
Spy49_1006c	-	1001027	1002349	82	0	NE	0	NE	0	NE
Spy49_1007c	-	1002469	1003704	772	0.00025	NE	1	E	1	E
Spy49_1008	cfa	1004074	1004847	440	0	NE	0	NE	0.24275	U

Spy49_1009	-	1005217	1006053	411	0.00025	NE	0	NE	0	NE
Spy49_1010c	-	1006069	1006698	257	0.00025	NE	0	NE	0.00025	NE
Spy49_1011c	-	1006708	1007349	278	0.00025	NE	0	NE	0.00025	NE
Spy49_1012c	-	1007456	1007791	209	0	NE	0	NE	0	NE
Spy49_1013c	glmS	1007987	1009801	213	0.00025	NE	0	NE	0	NE
Spy49_1014c	sipC	1009977	1010534	111	0	NE	0	NE	0	NE
Spy49_1015c	pyk	1010752	1012254	604	1	E	1	E	1	E
Spy49_1016c	pfk	1012317	1013330	185	0.00025	NE	0	NE	0.0015	NE
Spy49_1017c	dnaE	1013410	1016520	500	1	E	1	E	1	E
Spy49_1018c	-	1016705	1017076	337	0.99575	E	1	E	1	E
Spy49_1019c	-	1017076	1017774	1036	1	E	1	E	1	E
Spy49_1020	-	1017784	1018569	123	0.00025	NE	0	NE	0.00025	NE
Spy49_1021	-	1018706	1019311	232	0.00025	NE	0.0345	NE	0.99975	E
Spy49_1022	-	1020050	1020670	261	0.08325	U	0.00025	NE	1	E
Spy49_1023c	glgP	1020927	1023191	201	0.00025	NE	1	E	1	E
Spy49_1024	malQ	1023226	1024719	206	0.00025	NE	0	NE	0	NE
Spy49_1025c	malR	1024834	1025853	754	0.00025	NE	0	NE	0.00025	NE
Spy49_1026c	malE	1026097	1027344	497	0.00025	NE	0	NE	0.0005	NE
Spy49_1027c	malF	1027618	1028979	339	0.00025	NE	0.00075	NE	0.00375	NE
Spy49_1028	malG	1028979	1029815	415	0	NE	0	NE	0	NE
Spy49_1029	-	1029996	1030982	453	0.00025	NE	0	NE	0.00025	NE
Spy49_1030	-	1031207	1031431	278	0	NE	0	NE	0.00025	NE
Spy49_1819c	-	1031496	1032479	328	0	NE	0	NE	0	NE
Spy49_1033c	dltD	1032510	1033760	327	0	NE	0	NE	0	NE
Spy49_1034c	dltC	1033753	1033992	416	0.00025	NE	0	NE	0.00025	NE
Spy49_1035c	dltB	1034010	1035266	79	0.00025	NE	0	NE	0	NE
Spy49_1036c	dltA	1035263	1036801	418	0.00025	NE	0	NE	0.13625	U
Spy49_1037c	-	1036813	1036956	512	0.00025	NE	0	NE	0.888	U
Spy49_1038c	uvrB	1037219	1039210	47	-1	S	-1	S	-1	S
Spy49_1039c	glnP	1039403	1041577	663	0.00025	NE	0	NE	0.00025	NE
Spy49_1040	glnQ	1041577	1042317	724	0.00725	NE	0	NE	1	E
Spy49_1041	-	1042465	1042617	246	0.00025	NE	0	NE	0.99975	E
Spy49_1042c	-	1042614	1043993	50	-1	S	-1	S	-1	S
Spy49_1043c	-	1044171	1044620	459	0	NE	0	NE	0.003	NE
Spy49_1044c	-	1044617	1044952	149	0	NE	0	NE	0.00025	NE
Spy49_1045c	-	1044955	1045266	111	0.00025	NE	0	NE	0.00025	NE
Spy49_1046c	-	1045289	1047283	103	0	NE	0	NE	0	NE
Spy49_1047c	-	1047389	1048483	664	0	NE	0	NE	0.775	U
Spy49_1048c	-	1048492	1049892	364	0	NE	0	NE	0.00025	NE
Spy49_1049c	-	1050117	1050812	466	0.00025	NE	0	NE	0.00025	NE
Spy49_1050	-	1050817	1050975	231	0.00025	NE	0	NE	0.00025	NE
Spy49_1051c	obgE	1051044	1052357	52	-1	S	-1	S	-1	S
Spy49_1053c	-	1052786	1053181	437	0.81525	U	1	E	1	E
Spy49_1054	-	1053321	1054199	131	0	NE	0	NE	0	NE
Spy49_1055	-	1054597	1055097	292	0.00025	NE	0	NE	0.00025	NE
Spy49_1056	-	1055100	1055906	166	0	NE	0	NE	0	NE
Spy49_1057	-	1057303	1058613	268	0.00025	NE	0	NE	0.00025	NE
Spy49_1059	-	1058770	1058985	436	0	NE	0	NE	0	NE
Spy49_1060	-	1059129	1059314	71	0	NE	0	NE	0.18975	U
Spy49_1062	rsuA	1059757	1060488	61	0	NE	0	NE	0.00025	NE
Spy49_1064c	-	1060603	1060968	243	0	NE	0	NE	0.32225	U
Spy49_1065	-	1061088	1062308	121	0	NE	0	NE	0	NE
Spy49_1066c	-	1062720	1064165	406	0.0005	NE	0	NE	0.00025	NE
Spy49_1067	-	1064270	1064671	481	0.00025	NE	0	NE	0.00025	NE
Spy49_1070c	-	1064835	1065269	133	0	NE	0	NE	0	NE
Spy49_1071c	-	1065688	1067046	144	0	NE	0	NE	0	NE
Spy49_1072c	psr	1067128	1068579	452	0	NE	0	NE	0.00025	NE
Spy49_1073c	aroK	1068787	1069278	483	0	NE	0	NE	0	NE
Spy49_1074c	aroA1	1069271	1070554	163	0	NE	0	NE	0.00025	NE
Spy49_1075c	-	1070665	1071630	427	0	NE	0	NE	0.00025	NE
Spy49_1076c	map	1071632	1072492	321	0.01175	NE	0	NE	0	NE
Spy49_1077c	-	1072508	1073791	286	0.042	U	1	E	0.993	E
Spy49_1078c	-	1073800	1074342	427	0	NE	0	NE	0.51825	U
Spy49_1079c	grab	1074609	1075148	180	0	NE	0	NE	0	NE
Spy49_1080c	murZ	1075506	1076765	179	0	NE	0	NE	0	NE
Spy49_1082c	metK	1076939	1078135	419	0	NE	0	NE	0	NE
Spy49_1083c	inlA	1078672	1081050	398	1	E	1	E	1	E
Spy49_1084c	birA	1081425	1082195	792	0.0275	NE	0	NE	0.00025	NE

Spy49_1085	-	1082170	1082373	256	0.00025	NE	1	E	1	E
Spy49_1086c	dnaX	1082469	1084139	67	0	NE	0	NE	0.00025	NE
Spy49_1087c	-	1084139	1084636	556	1	E	1	E	1	E
Spy49_1088c	-	1084842	1085591	165	0	NE	0	NE	0	NE
Spy49_1089	udk	1085989	1086615	249	0	NE	0	NE	0	NE
Spy49_1090c	deaD2	1086713	1087798	208	0	NE	0	NE	0	NE
Spy49_1091	-	1087909	1089183	361	0	NE	0	NE	0	NE
Spy49_1092c	gapN	1089304	1090707	424	0	NE	0	NE	0	NE
Spy49_1093c	ptsI	1090892	1092625	467	1	E	1	E	1	E
Spy49_1094c	ptsH	1092630	1092893	577	0.63275	U	0.00725	NE	1	E
Spy49_1095c	nrdH	1093253	1093504	87	0.959	U	0.11725	U	0.94925	U
Spy49_1096	nrdE1	1093524	1095683	83	0	NE	0	NE	0	NE
Spy49_1097	nrdF1	1095664	1095981	719	0.0005	NE	0	NE	0.003	NE
Spy49_1098	nrdF	1096016	1096975	105	0	NE	0	NE	0	NE
Spy49_1099	-	1096950	1098263	319	0	NE	0	NE	0.00075	NE
Spy49_1100	-	1098401	1098694	437	0.00025	NE	0	NE	0.00025	NE
Spy49_1101	-	1099127	1099297	97	0	NE	0	NE	0.0715	U
Spy49_1103	-	1099337	1099558	56	-1	S	-1	S	-1	S
Spy49_1104	-	1099651	1100346	73	0	NE	0	NE	0	NE
Spy49_1105c	-	1100365	1101126	231	0	NE	0	NE	0	NE
Spy49_1106c	-	1101123	1101353	253	0	NE	0	NE	0	NE
Spy49_1107c	alaS	1101699	1104317	76	0.0235	NE	0	NE	0	NE
Spy49_1108c	prsA	1104704	1105759	872	1	E	1	E	1	E
Spy49_1109c	-	1105822	1106529	351	0	NE	0	NE	0	NE
Spy49_1110c	-	1106595	1107791	235	0.00025	NE	0	NE	0	NE
Spy49_1111c	pepB	1108168	1109973	398	0.00025	NE	0	NE	0.00025	NE
Spy49_1112c	-	1109986	1110948	601	0.00025	NE	0	NE	0.00025	NE
Spy49_1113c	-	1111245	1111961	320	0	NE	0	NE	0.00025	NE
Spy49_1114c	nagB	1112080	1112784	238	0	NE	0	NE	0	NE
Spy49_1115c	queA	1112986	1114014	234	0	NE	0	NE	0	NE
Spy49_1116	-	1114021	1115241	342	0	NE	0	NE	0.00025	NE
Spy49_1117	-	1115355	1115945	406	0	NE	0	NE	0.00025	NE
Spy49_1119c	-	1115942	1116190	196	0	NE	0	NE	0.00025	NE
Spy49_1120c	-	1116175	1116402	82	0	NE	0	NE	0	NE
Spy49_1121c	sodM	1116524	1117168	75	0.57725	U	0	NE	0.96925	U
Spy49_1122c	holA	1117265	1118305	214	0	NE	0	NE	0	NE
Spy49_1123c	comEC	1118376	1120619	346	1	E	1	E	1	E
Spy49_1124c	comE	1120600	1121262	747	0	NE	0	NE	0.00025	NE
Spy49_1126c	-	1121462	1122202	220	0	NE	0	NE	0.008	NE
Spy49_1127c	-	1122242	1123096	246	1	E	1	E	1	E
Spy49_1128	-	1123086	1123364	284	0.00025	NE	0	NE	0	NE
Spy49_1129	kup	1123388	1125388	92	0	NE	0	NE	0.00025	NE
Spy49_1130c	deaD1	1125516	1127135	666	0.00025	NE	0	NE	0	NE
Spy49_1132c	prfC	1127442	1128986	539	0	NE	0	NE	0	NE
Spy49_1133c	-	1129234	1129929	514	0	NE	0	NE	0.00025	NE
Spy49_1135c	murF	1130009	1131400	231	0	NE	0	NE	0.00025	NE
Spy49_1136c	ddl	1131591	1132637	463	1	E	1	E	1	E
Spy49_1137c	recR	1132738	1133334	348	1	E	1	E	1	E
Spy49_1138c	-	1133381	1133572	198	0.00725	NE	0	NE	0.99875	E
Spy49_1139c	fdhC	1134132	1134947	63	0	NE	0	NE	0.02225	NE
Spy49_1140c	-	1135034	1135576	271	0	NE	0	NE	0.00025	NE
Spy49_1141c	-	1135737	1136258	180	0.00025	NE	0	NE	0.00025	NE
Spy49_1142c	gpmA	1136365	1137060	173	0.00025	NE	0	NE	0.00025	NE
Spy49_1143c	pyrD	1137357	1138238	231	0.996	E	1	E	0.9925	E
Spy49_1144	-	1138293	1138466	293	0	NE	0	NE	0	NE
Spy49_1145c	cadA	1138538	1140400	57	-1	S	-1	S	-1	S
Spy49_1146c	-	1140732	1141007	620	0.00025	NE	0	NE	0.062	U
Spy49_1147c	-	1141106	1141693	91	0.00025	NE	1	E	0.84025	U
Spy49_1148c	-	1141671	1142513	195	0.00025	NE	0	NE	0.00025	NE
Spy49_1149c	-	1142506	1143357	280	0	NE	0	NE	0.0005	NE
Spy49_1150c	-	1143573	1144529	283	0	NE	0.00025	NE	0.9995	E
Spy49_1151c	recN	1144701	1146362	318	0	NE	0	NE	0	NE
Spy49_1152c	-	1146383	1146853	553	0.00025	NE	0	NE	0	NE
Spy49_1153c	-	1146840	1147667	156	0	NE	0	NE	0	NE
Spy49_1154c	fps	1147867	1148532	275	0	NE	0	NE	0	NE
Spy49_1155c	xseB	1148532	1148747	221	0	NE	0	NE	0	NE
Spy49_1156c	xseA	1148725	1150065	71	0	NE	0	NE	0	NE
Spy49_1157c	folD	1150218	1151072	446	0	NE	0	NE	0	NE

Spy49_1158c	-	1151280	1152974	284	0.00025	NE	0	NE	0.98475	U
Spy49_1159c	phr	1153152	1154561	564	0.00025	NE	0	NE	0	NE
Spy49_1160c	gtr	1154710	1155444	469	0.00025	NE	0	NE	0.00025	NE
Spy49_1161c	-	1155444	1156130	244	0	NE	0	NE	0.00025	NE
Spy49_1162c	-	1156257	1156487	228	0.00025	NE	0	NE	0.00025	NE
Spy49_1163c	clpE	1156785	1159067	76	0.8645	U	0.006	NE	0.878	U
Spy49_1164	mutT	1159195	1159650	760	0.00025	NE	0	NE	0.00075	NE
Spy49_1165	-	1159701	1160003	151	0	NE	0	NE	0	NE
Spy49_1166	ileS	1160268	1163024	100	0.00025	NE	0	NE	0.00025	NE
Spy49_1167c	divIVA	1163342	1164100	918	1	E	1	E	1	E
Spy49_1168c	-	1164110	1164901	252	0.00025	NE	0	NE	0.204	U
Spy49_1169c	-	1164901	1165155	263	0.00025	NE	0	NE	0	NE
Spy49_1170c	-	1165160	1165828	84	0	NE	0	NE	0.0005	NE
Spy49_1171c	-	1165828	1166499	222	0.91975	U	0.00025	NE	1	E
Spy49_1172c	ftsZ	1166502	1167821	223	0	NE	0	NE	0	NE
Spy49_1173c	ftsA	1167845	1169209	439	1	E	1	E	1	E
Spy49_1174c	ftsQ	1169420	1170568	454	1	E	1	E	1	E
Spy49_1175c	murG	1170569	1171651	382	0.00025	NE	1	E	0.99975	E
Spy49_1176c	murD	1171651	1173009	360	1	E	1	E	1	E
Spy49_1177c	-	1173365	1173616	452	1	E	1	E	1	E
Spy49_1178c	-	1173738	1175579	83	0	NE	0	NE	0	NE
Spy49_1179c	-	1175762	1176151	613	0	NE	0	NE	0	NE
Spy49_1180c	-	1176161	1177132	129	0	NE	0	NE	0.00025	NE
Spy49_1181c	-	1177137	1177340	323	0	NE	0	NE	0.00025	NE
Spy49_1182c	-	1177482	1178009	67	0	NE	0	NE	0	NE
Spy49_1183c	-	1178237	1178863	175	0.00025	NE	0	NE	0	NE
Spy49_1184	-	1178945	1180024	208	0	NE	0	NE	0	NE
Spy49_1185c	-	1180028	1180648	359	0.00025	NE	0	NE	0	NE
Spy49_1186c	-	1181096	1182073	206	0	NE	0	NE	0	NE
Spy49_1187c	-	1182487	1183524	325	0.00025	NE	0	NE	0.00025	NE
Spy49_1188c	coaD	1183511	1184002	345	0	NE	0	NE	0	NE
Spy49_1189c	-	1183992	1184531	163	1	E	1	E	1	E
Spy49_1190c	asnA	1184654	1185646	179	0	NE	0	NE	0	NE
Spy49_1191c	arcC	1185959	1186909	330	0	NE	0	NE	0.00025	NE
Spy49_1192c	-	1186929	1188260	316	0	NE	0	NE	0.1035	U
Spy49_1193c	-	1188277	1189578	443	0	NE	0	NE	0	NE
Spy49_1194c	arcB	1189941	1190954	433	0	NE	0	NE	0	NE
Spy49_1195c	-	1190979	1191419	337	0.163	U	0	NE	0	NE
Spy49_1196c	arcA	1191519	1192754	146	0	NE	0	NE	0	NE
Spy49_1197c	-	1193028	1193708	411	0	NE	0	NE	0	NE
Spy49_1198c	argR	1193850	1194323	226	0.00025	NE	0	NE	0	NE
Spy49_1199	-	1194490	1195206	157	0	NE	0	NE	0	NE
Spy49_1200c	-	1195220	1196299	238	0	NE	0	NE	0.00025	NE
Spy49_1201c	yesM	1196372	1198105	359	0	NE	0	NE	0.00025	NE
Spy49_1202c	yesN	1198102	1198842	577	0	NE	0	NE	0.00025	NE
Spy49_1203c	msrA1	1198930	1200036	246	0	NE	0	NE	0.00025	NE
Spy49_1204c	-	1200079	1200702	368	0.00025	NE	0	NE	0.0005	NE
Spy49_1205c	ccdA	1200715	1201404	207	0.00025	NE	0	NE	0.00025	NE
Spy49_1206c	-	1202097	1202390	229	0.00025	NE	0	NE	0	NE
Spy49_1207c	-	1202401	1203426	97	0	NE	0	NE	0	NE
Spy49_1208c	-	1203423	1204097	341	0	NE	0	NE	0.00025	NE
Spy49_1209c	-	1204099	1204947	224	0	NE	0	NE	0.00025	NE
Spy49_1210c	-	1204952	1206847	282	0	NE	0	NE	0	NE
Spy49_1211c	-	1206847	1207575	631	0.12025	U	0	NE	0.721	U
Spy49_1212c	-	1207708	1210110	242	0.00025	NE	0	NE	0.00025	NE
Spy49_1213c	valS	1210270	1212918	800	0.00025	NE	0	NE	0.00025	NE
Spy49_1215c	-	1212920	1213483	882	1	E	1	E	1	E
Spy49_1216c	-	1213480	1213659	187	0	NE	0	NE	0	NE
Spy49_1217c	-	1214086	1214481	59	-1	S	-1	S	-1	S
Spy49_1218c	-	1214499	1214753	131	0.9435	U	0	NE	0	NE
Spy49_1219c	aroA2	1215231	1215983	84	0.00025	NE	0.00325	NE	0.36475	U
Spy49_1220	aroB	1216039	1217112	250	0	NE	0	NE	0	NE
Spy49_1221	-	1217548	1217853	357	0	NE	0	NE	0.00025	NE
Spy49_1222c	-	1217855	1218193	101	0	NE	0	NE	0	NE
Spy49_1223c	-	1218246	1219001	112	0	NE	0	NE	0	NE
Spy49_1224c	aroE1	1219236	1220114	251	0	NE	0	NE	0.645	U
Spy49_1225c	-	1220252	1223668	292	0	NE	0	NE	0.00025	NE
Spy49_1226c	-	1223688	1225172	1138	0	NE	0	NE	0.03425	NE

Spy49_1227c	-	1225172	1226896	494	0	NE	0	NE	0.00025	NE
Spy49_1228c	-	1226886	1227491	574	0	NE	0	NE	0	NE
Spy49_1229c	-	1227797	1229242	201	0	NE	0	NE	0.002	NE
Spy49_1230c	-	1229323	1230249	481	0	NE	0	NE	0.02875	NE
Spy49_1231c	-	1230259	1231209	308	0.536	U	0	NE	0.00025	NE
Spy49_1233c	-	1231405	1232100	316	0	NE	0	NE	0	NE
Spy49_1234	-	1232893	1234335	231	0.00025	NE	0	NE	0	NE
Spy49_1235c	hyl	1234359	1236053	480	0	NE	0	NE	0	NE
Spy49_1236c	-	1236104	1237144	564	0	NE	0	NE	0.03875	U
Spy49_1237c	-	1237277	1238563	346	0.00025	NE	0	NE	0.00025	NE
Spy49_1238	-	1238578	1241283	428	0	NE	0	NE	0.15425	U
Spy49_1239	-	1241384	1242739	901	0	NE	0	NE	0.91675	U
Spy49_1240c	-	1243405	1244760	451	0	NE	0	NE	0	NE
Spy49_1241c	recX	1244875	1245651	451	0.00025	NE	0	NE	0.7135	U
Spy49_1242	-	1245731	1246264	258	0	NE	0	NE	0.00025	NE
Spy49_1243	-	1246634	1246912	177	0.00025	NE	0	NE	0.06475	U
Spy49_1244	-	1246927	1247034	92	0	NE	0	NE	0	NE
Spy49_1245	-	1254000	1254548	35	-1	S	-1	S	-1	S
Spy49_1247c	comF	1254628	1255293	182	0	NE	0	NE	0.00025	NE
Spy49_1248c	-	1255265	1256590	221	0	NE	0	NE	0	NE
Spy49_1249c	-	1256646	1257278	441	0	NE	0	NE	0	NE
Spy49_1250	cysM	1257406	1258347	210	0.00025	NE	0	NE	0	NE
Spy49_1251	-	1258365	1258742	313	0	NE	0	NE	0	NE
Spy49_1252c	-	1258742	1260142	125	0	NE	0	NE	0.993	E
Spy49_1253c	-	1260179	1260820	466	0	NE	0	NE	0.00225	NE
Spy49_1254c	-	1260813	1261817	213	0.00025	NE	1	E	0.99675	E
Spy49_1255c	-	1261814	1262506	334	0	NE	0	NE	0.00025	NE
Spy49_1256c	-	1262629	1264527	230	0	NE	0	NE	0	NE
Spy49_1257c	pppL	1264524	1265264	632	0.5185	U	1	E	1	E
Spy49_1259c	sunL	1265302	1266624	246	0.9555	U	1	E	1	E
Spy49_1260c	fmt	1266614	1267549	440	0	NE	0	NE	0	NE
Spy49_1261c	priA	1267611	1269995	311	0.99975	E	1	E	1	E
Spy49_1262c	-	1270060	1270377	794	0.00025	NE	0.00025	NE	1	E
Spy49_1263c	gmk	1270393	1271028	105	0.0435	U	0.00275	NE	0.979	U
Spy49_1265c	-	1271138	1272745	211	1	E	1	E	0.998	E
Spy49_1266c	-	1272875	1273771	535	0	NE	0	NE	0	NE
Spy49_1267c	atoB	1273972	1275159	298	0	NE	0	NE	0	NE
Spy49_1268	atoD1	1275183	1275833	395	0	NE	0	NE	0.22225	U
Spy49_1269	atoA	1275835	1276494	216	0	NE	0	NE	0.00025	NE
Spy49_1270	-	1276527	1277306	219	0	NE	0	NE	0	NE
Spy49_1271	-	1277377	1278708	259	0	NE	0	NE	0.00025	NE
Spy49_1272	luxS	1278782	1279264	443	0	NE	0	NE	0.00025	NE
Spy49_1273	-	1279411	1280880	160	0	NE	0	NE	0	NE
Spy49_1274c	-	1280894	1282048	489	0	NE	0	NE	0.00025	NE
Spy49_1275c	-	1282493	1282819	384	0	NE	0	NE	0	NE
Spy49_1276c	-	1282947	1283456	108	0.99925	E	1	E	0.95425	U
Spy49_1277c	recU	1283537	1284145	169	0	NE	0	NE	0.00025	NE
Spy49_1278	pbp1A	1284132	1286297	202	0.9875	U	0.2455	U	0.986	U
Spy49_1279	pepC	1286764	1288101	721	0.95475	U	1	E	1	E
Spy49_1280c	nadE	1288286	1289134	445	0.00025	NE	0	NE	0	NE
Spy49_1281c	-	1289112	1290566	282	1	E	1	E	0.998	E
Spy49_1282c	aapA	1290737	1292116	484	0.9995	E	1	E	1	E
Spy49_1283c	nox2	1292285	1293202	459	0.00025	NE	0	NE	0	NE
Spy49_1284c	-	1293266	1293490	305	0	NE	0	NE	0	NE
Spy49_1285c	-	1293594	1294337	74	0	NE	1	E	0.00125	NE
Spy49_1286c	-	1294337	1295140	247	0.00025	NE	0	NE	0.00025	NE
Spy49_1287c	-	1295335	1296678	267	0.00025	NE	0	NE	0.00025	NE
Spy49_1289c	mraY	1296836	1297846	447	0.9465	U	1	E	1	E
Spy49_1290c	ftsI	1297848	1300103	336	0.98825	U	1	E	1	E
Spy49_1291c	ftsL	1300107	1300430	751	0.99975	E	1	E	1	E
Spy49_1292c	mraW	1300435	1301385	107	0.58975	U	1	E	0.93775	U
Spy49_1293c	-	1301736	1301942	316	0.00025	NE	0	NE	0.00025	NE
Spy49_1294c	proA	1301917	1303167	68	0	NE	0	NE	0	NE
Spy49_1295c	proB	1303160	1303981	416	0	NE	0	NE	0	NE
Spy49_1296c	-	1304045	1305673	273	0	NE	0	NE	0.00025	NE
Spy49_1297c	-	1305678	1306412	542	0	NE	0	NE	0.00025	NE
Spy49_1298c	-	1306446	1306712	244	0	NE	0	NE	0	NE
Spy49_1299c	tkt	1306904	1308889	88	0	NE	0	NE	0.00025	NE

Spy49_1300c	tal	1309107	1309751	661	0.32125	U	0.00725	NE	1	E
Spy49_1301c	-	1309877	1311376	214	0.00025	NE	0	NE	0	NE
Spy49_1303c	npx	1311366	1312712	499	0	NE	0	NE	0	NE
Spy49_1304c	glpF1	1312821	1313522	448	0	NE	0	NE	0.00025	NE
Spy49_1305c	glpO	1313524	1315362	233	0	NE	0	NE	0.0045	NE
Spy49_1306c	glpK	1315378	1316904	612	0	NE	0	NE	0.00025	NE
Spy49_1307c	-	1317264	1317656	508	0.00125	NE	0	NE	0.00025	NE
Spy49_1308c	-	1317784	1318041	130	0.00025	NE	0	NE	0	NE
Spy49_1309c	glyS	1318195	1320234	85	0	NE	0	NE	0	NE
Spy49_1310c	glyQ	1320606	1321523	679	1	E	1	E	1	E
Spy49_1311c	-	1321896	1322402	305	1	E	1	E	1	E
Spy49_1312c	-	1322561	1323400	168	0.00025	NE	0	NE	0	NE
Spy49_1313c	nagA	1323522	1324670	279	0	NE	0	NE	0.00025	NE
Spy49_1314c	-	1324787	1326418	382	0	NE	0	NE	0.00025	NE
Spy49_1316c	-	1327469	1328311	543	0.00025	NE	0	NE	0.03675	NE
Spy49_1318c	-	1328604	1329161	280	0	NE	0	NE	0	NE
Spy49_1319	-	1329198	1330022	185	0.00025	NE	0	NE	0.00025	NE
Spy49_1320c	-	1330024	1330641	274	0	NE	0	NE	0	NE
Spy49_1321c	lacD1	1330838	1331815	205	0	NE	0	NE	0.00025	NE
Spy49_1322c	lacC1	1331897	1332160	325	0	NE	0	NE	0	NE
Spy49_1323c	-	1331965	1332315	87	0	NE	0	NE	0	NE
Spy49_1324c	lacB	1332325	1332840	116	-1	S	-1	S	-1	S
Spy49_1325c	lacA	1332855	1333280	171	0	NE	0	NE	0	NE
Spy49_1326c	-	1333538	1334989	141	0	NE	0	NE	0.00025	NE
Spy49_1327c	-	1335015	1335320	483	0	NE	0	NE	0.00025	NE
Spy49_1328c	-	1335313	1335786	101	0	NE	0	NE	0	NE
Spy49_1329c	lacR1	1336023	1336793	157	0.00025	NE	0	NE	0	NE
Spy49_1330	copZ	1336997	1337200	256	0	NE	0	NE	0	NE
Spy49_1331c	copA	1337214	1339445	67	0	NE	0	NE	0	NE
Spy49_1332c	copY	1339445	1339882	743	0	NE	0	NE	0	NE
Spy49_1334c	-	1340045	1341046	145	0	NE	0	NE	0	NE
Spy49_1335	rbfA	1341098	1341448	333	0	NE	0	NE	0	NE
Spy49_1336c	infB	1341654	1344515	116	0.9855	U	1	E	0.98375	U
Spy49_1337c	-	1344535	1344837	953	0.99975	E	1	E	1	E
Spy49_1338c	-	1344830	1345126	100	0.00025	NE	0	NE	0.00025	NE
Spy49_1339c	nusA	1345142	1346299	98	0.9875	U	1	E	0.9865	U
Spy49_1340c	-	1346474	1347010	385	1	E	1	E	1	E
Spy49_1341c	trmB	1347269	1347904	178	0.00025	NE	0	NE	0.00025	NE
Spy49_1342c	-	1347904	1348695	211	0	NE	0	NE	0	NE
Spy49_1343c	-	1348759	1349793	263	0.38775	U	1	E	1	E
Spy49_1344c	-	1349796	1350521	344	0	NE	0	NE	0.00025	NE
Spy49_1345c	hit	1350593	1351012	241	0	NE	0	NE	0.0005	NE
Spy49_1346	-	1351009	1351365	139	0	NE	0	NE	0	NE
Spy49_1347	-	1351476	1352759	118	0	NE	0	NE	0	NE
Spy49_1348c	-	1352768	1353292	427	0	NE	0	NE	0	NE
Spy49_1349c	-	1353267	1353728	174	0	NE	0	NE	0	NE
Spy49_1350c	-	1353882	1355342	153	0.00025	NE	1	E	0.04125	U
Spy49_1351c	-	1355607	1356002	486	0.00025	NE	0	NE	0	NE
Spy49_1352	-	1356019	1356420	131	0	NE	0	NE	0	NE
Spy49_1353	manL	1356772	1357764	133	0.00025	NE	0	NE	0	NE
Spy49_1354	manM	1357853	1358662	330	0	NE	0	NE	0.00025	NE
Spy49_1355	manN	1358679	1359590	269	0	NE	0	NE	0	NE
Spy49_1356	-	1359704	1360063	303	0.00025	NE	0	NE	0	NE
Spy49_1357	serS	1360454	1361731	119	0.00025	NE	0	NE	0	NE
Spy49_1358	accA	1361964	1362734	425	0.99975	E	1	E	1	E
Spy49_1359c	accD	1362731	1363597	256	0.66025	U	1	E	0.97275	U
Spy49_1360c	accC	1363606	1364970	288	1	E	1	E	1	E
Spy49_1361c	fabZ	1365002	1365424	454	1	E	1	E	1	E
Spy49_1362c	accB	1365421	1365921	140	0.98325	U	0.9985	E	0.97425	U
Spy49_1363c	fabF	1365923	1367155	166	0.6345	U	0.99975	E	0.9875	U
Spy49_1364c	fabG	1367170	1367904	410	1	E	1	E	1	E
Spy49_1365c	fabD	1367894	1368832	244	0.95475	U	1	E	1	E
Spy49_1366c	fabK	1368857	1369828	312	1	E	1	E	0.9965	E
Spy49_1367c	acpP	1370029	1370253	323	0.952	U	1	E	1	E
Spy49_1368c	fabH	1370314	1371288	74	0.724	U	1	E	0.96375	U
Spy49_1369c	-	1371289	1371723	324	0.73025	U	1	E	0.996	E
Spy49_1370c	phaB	1371797	1372588	144	0	NE	0	NE	0	NE
Spy49_1371c	dnaJ	1372809	1373945	263	0.99975	E	1	E	1	E

Spy49_1372c	-	1374008	1374217	378	0.99475	E	1	E	1	E
Spy49_1373	dnaK	1374226	1376052	69	0.60925	U	0	NE	0.00075	NE
Spy49_1374c	grpE	1376233	1376805	608	1	E	1	E	1	E
Spy49_1375c	hrcA	1376808	1377851	190	0.92525	U	1	E	0.99075	E
Spy49_1376c	-	1377978	1378547	347	0.00025	NE	0	NE	0.00025	NE
Spy49_1377c	-	1378544	1379287	189	0	NE	0	NE	0	NE
Spy49_1378c	-	1379277	1379984	247	0	NE	0	NE	0	NE
Spy49_1379c	-	1380376	1380615	235	0	NE	0	NE	0	NE
Spy49_1380c	-	1380834	1381049	79	0	NE	0	NE	0	NE
Spy49_1381c	gatB	1381046	1382485	71	0	NE	0	NE	0	NE
Spy49_1382c	gatA	1382485	1383951	479	1	E	1	E	1	E
Spy49_1383c	gatC	1383951	1384253	488	1	E	1	E	1	E
Spy49_1384c	-	1384485	1384733	100	0.96525	U	1	E	0.955	U
Spy49_1385c	-	1385248	1385802	82	0	NE	0	NE	0	NE
Spy49_1386c	codY	1385949	1386731	184	0.00025	NE	0	NE	0	NE
Spy49_1387c	-	1386949	1388130	260	1	E	1	E	1	E
Spy49_1388c	-	1388395	1388847	393	0	NE	0	NE	0	NE
Spy49_1389	-	1388970	1390358	150	0	NE	0	NE	0	NE
Spy49_1390c	asnB	1390430	1391395	462	0	NE	0	NE	0.00025	NE
Spy49_1391	-	1391744	1392448	321	0	NE	0	NE	0.00025	NE
Spy49_1392c	-	1392461	1393363	234	0	NE	0	NE	0	NE
Spy49_1393c	recG	1393655	1395670	300	0.00025	NE	0	NE	0.00025	NE
Spy49_1394c	-	1396045	1397499	671	0	NE	0	NE	0.9995	E
Spy49_1395c	-	1397436	1398116	484	0	NE	0	NE	0	NE
Spy49_1396c	-	1398113	1398706	226	0.00025	NE	0	NE	0.00025	NE
Spy49_1397c	cycC	1398703	1400373	197	0	NE	0	NE	0	NE
Spy49_1398c	cycD	1400366	1402129	556	0.00025	NE	0	NE	0	NE
Spy49_1400c	fhuC2	1402126	1402962	587	0.00025	NE	0	NE	0	NE
Spy49_1401c	fhuB2	1402959	1403981	278	0	NE	0	NE	0	NE
Spy49_1402c	fhuD2	1403983	1405728	340	0	NE	0	NE	0.00125	NE
Spy49_1403c	-	1405925	1409752	581	0	NE	0	NE	0.00025	NE
Spy49_1405c	isp2	1410242	1411753	1275	0.00025	NE	0	NE	0.00025	NE
Spy49_1407c	alr	1411840	1412940	503	0.00025	NE	0	NE	0	NE
Spy49_1408c	acpS	1412937	1413293	366	1	E	1	E	1	E
Spy49_1409c	secA	1413409	1415928	118	0.9995	E	1	E	0.9975	E
Spy49_1410c	pmi	1417389	1418342	839	1	E	1	E	1	E
Spy49_1412c	scrK	1418389	1419393	317	0	NE	0	NE	0	NE
Spy49_1413c	endoS	1419562	1422093	334	0	NE	0	NE	0	NE
Spy49_1414c	scrA	1422324	1424207	843	0	NE	0	NE	0	NE
Spy49_1415c	scrB	1424449	1425888	627	0	NE	0	NE	0	NE
Spy49_1416	scrR	1425893	1426858	479	0	NE	0	NE	0.12925	U
Spy49_1417	nusB	1426999	1427451	321	0.00025	NE	0	NE	0.00025	NE
Spy49_1418c	-	1427444	1427833	150	0	NE	0	NE	0	NE
Spy49_1419c	efp	1427879	1428436	129	0.00025	NE	0	NE	0.0005	NE
Spy49_1420c	comEB	1428532	1428993	185	0	NE	0	NE	0	NE
Spy49_1421c	pepP	1429028	1430101	153	0	NE	0	NE	0.00025	NE
Spy49_1422c	uvrA	1430216	1433074	357	0	NE	0	NE	0	NE
Spy49_1423c	-	1433247	1434191	952	0	NE	0	NE	0	NE
Spy49_1424	-	1434323	1434979	314	0.212	U	0.00025	NE	0.99825	E
Spy49_1425	rpsR	1435112	1435351	218	0	NE	0	NE	0	NE
Spy49_1426c	ssb2	1435516	1436007	79	0.87025	U	0.9515	U	0.02275	NE
Spy49_1427c	rpsF	1436029	1436319	163	0.00175	NE	0.9875	U	0.999	E
Spy49_1428c	-	1436492	1436785	96	0.97675	U	0	NE	0.971	U
Spy49_1429c	mutY	1436953	1438107	97	0	NE	0	NE	0.00025	NE
Spy49_1430	-	1438284	1438871	384	0	NE	0	NE	0	NE
Spy49_1431	trx2	1438923	1439237	195	0.00025	NE	0	NE	0.00025	NE
Spy49_1432c	-	1439318	1439713	104	0	NE	0	NE	0.00025	NE
Spy49_1433c	mutS2	1439822	1442161	131	0	NE	0	NE	0	NE
Spy49_1434c	-	1442310	1442855	779	0.00025	NE	0	NE	0	NE
Spy49_1435c	-	1442858	1443166	181	0.00025	NE	0	NE	0	NE
Spy49_1436c	-	1443323	1444225	102	0	NE	0	NE	0	NE
Spy49_1437	spi	1444236	1444829	300	0.9885	U	1	E	1	E
Spy49_1438	recD	1444887	1447340	197	0.009	NE	1	E	0.99975	E
Spy49_1439	-	1447431	1447913	817	0	NE	0	NE	0.0005	NE
Spy49_1440	dinP	1448006	1449100	160	0	NE	0	NE	0.00025	NE
Spy49_1441c	pfl	1449309	1451636	364	0.06525	U	0	NE	0.0685	U
Spy49_1442	-	1451724	1452113	775	0.00025	NE	0	NE	0.06875	U
Spy49_1443c	cppA	1452098	1452850	129	0	NE	0	NE	0	NE

Spy49_1444c	-	1453112	1454044	250	0	NE	0	NE	0	NE
Spy49_1445	glpF2	1454380	1455327	310	0	NE	0	NE	0	NE
Spy49_1447c	norA	1455730	1456926	315	0	NE	0	NE	0.963	U
Spy49_1449c	-	1457032	1457751	398	0.00025	NE	0	NE	0	NE
Spy49_1450	pepXP	1457773	1460055	239	0.00025	NE	0	NE	0	NE
Spy49_1451	-	1460135	1460356	760	0.00025	NE	0	NE	0.00025	NE
Spy49_1452c	-	1460526	1460900	73	0	NE	0	NE	0	NE
Spy49_1453	-	1461245	1461424	124	0	NE	0	NE	0	NE
Spy49_1454c	spd3	1461662	1462462	59	0.00925	NE	0	NE	0	NE
Spy49_1455	-	1462842	1463168	266	0	NE	0	NE	0	NE
Spy49_1456	-	1463218	1464084	108	0	NE	0	NE	0	NE
Spy49_1457c	-	1464072	1464596	288	0	NE	0	NE	0	NE
Spy49_1458c	-	1464736	1465944	174	0	NE	0	NE	0	NE
Spy49_1459c	-	1466060	1466287	402	0	NE	0	NE	0	NE
Spy49_1460c	-	1466284	1466559	75	0	NE	0	NE	0	NE
Spy49_1461c	-	1466569	1467186	91	0	NE	0	NE	0	NE
Spy49_1462c	-	1467189	1467350	205	0	NE	0	NE	0	NE
Spy49_1463c	-	1467364	1469274	53	-1	S	-1	S	-1	S
Spy49_1464c	-	1469290	1470294	636	0.00025	NE	0	NE	0.00025	NE
Spy49_1465c	-	1470291	1472342	334	0.00025	NE	0	NE	0.8635	U
Spy49_1466c	-	1472339	1473118	683	0.00075	NE	0	NE	0.90375	U
Spy49_1467c	-	1473151	1476786	259	0.00025	NE	0	NE	0.06925	U
Spy49_1468c	-	1476801	1477130	1211	0	NE	0	NE	0.00225	NE
Spy49_1470c	-	1477172	1477531	109	0	NE	0	NE	0.0005	NE
Spy49_1471c	-	1477584	1478237	119	0.00025	NE	0	NE	0.00025	NE
Spy49_1472c	-	1478247	1478636	217	0	NE	0	NE	0.00025	NE
Spy49_1474c	-	1478633	1478989	129	0	NE	0	NE	0.00025	NE
Spy49_1475c	-	1478979	1479287	118	0	NE	0	NE	0.00025	NE
Spy49_1476c	-	1479284	1479637	102	0	NE	0	NE	0	NE
Spy49_1477c	-	1479651	1479893	117	0.00025	NE	0	NE	0	NE
Spy49_1478c	-	1479903	1480985	80	0	NE	0	NE	0.007	NE
Spy49_1479c	-	1480988	1481368	360	0	NE	0	NE	0	NE
Spy49_1480c	-	1481378	1481911	126	0.00025	NE	0	NE	0.00025	NE
Spy49_1481c	-	1482055	1482321	177	0	NE	0	NE	0	NE
Spy49_1482c	-	1482324	1482638	88	0	NE	0	NE	0	NE
Spy49_1483c	-	1482708	1482893	104	0	NE	0	NE	0	NE
Spy49_1484c	-	1482897	1484459	61	0	NE	0	NE	0	NE
Spy49_1485c	-	1484440	1485942	520	0	NE	0	NE	0	NE
Spy49_1486c	-	1485954	1487243	500	0	NE	0	NE	0	NE
Spy49_1487c	-	1487221	1487703	429	0	NE	0	NE	0	NE
Spy49_1488c	-	1488535	1488975	160	0.00025	NE	0	NE	0	NE
Spy49_1489c	-	1489415	1489936	146	0	NE	0	NE	0	NE
Spy49_1491c	-	1489933	1490226	173	0	NE	0	NE	0.00025	NE
Spy49_1492c	-	1490223	1490408	97	0	NE	0	NE	0.0005	NE
Spy49_1493c	-	1490506	1490991	61	0.03475	NE	0	NE	0	NE
Spy49_1494c	-	1491276	1491680	161	0	NE	0	NE	0.952	U
Spy49_1496c	-	1491664	1491954	134	0	NE	0	NE	0	NE
Spy49_1497c	-	1492193	1492549	96	0.52275	U	0	NE	0.0015	NE
Spy49_1498c	-	1492625	1492987	118	0	NE	0	NE	0.00975	NE
Spy49_1499c	-	1493196	1493621	120	0	NE	0	NE	0.00025	NE
Spy49_1500c	-	1493614	1494288	141	0	NE	0	NE	0.09725	U
Spy49_1501c	-	1494289	1494435	224	0	NE	0	NE	0.00025	NE
Spy49_1502c	-	1494793	1495047	48	-1	S	-1	S	-1	S
Spy49_1504c	-	1495034	1495381	84	0	NE	0	NE	0.00025	NE
Spy49_1505c	-	1495522	1497093	115	0	NE	0	NE	0.10075	U
Spy49_1506c	-	1497209	1497652	523	0.00025	NE	0	NE	0.02375	NE
Spy49_1507c	-	1498078	1498350	147	0	NE	0	NE	0.00025	NE
Spy49_1509c	-	1498523	1498702	90	0.00025	NE	0	NE	0.0005	NE
Spy49_1510c	-	1498833	1498967	59	0.9765	U	1	E	0.97825	U
Spy49_1511c	-	1499249	1499608	44	-1	S	-1	S	-1	S
Spy49_1512c	-	1499682	1499939	119	0.00025	NE	0	NE	0	NE
Spy49_1513c	-	1500142	1500483	85	0	NE	0	NE	0	NE
Spy49_1514c	-	1500755	1500904	113	0.00025	NE	0	NE	0.00025	NE
Spy49_1516c	-	1500936	1501664	49	-1	S	-1	S	-1	S
Spy49_1517c	-	1501697	1501885	242	0	NE	0	NE	0.00025	NE
Spy49_1518c	-	1502073	1502255	62	0.00025	NE	0	NE	0.00225	NE
Spy49_1520c	-	1502532	1503293	60	-1	S	-1	S	-1	S
Spy49_1521c	-	1503460	1503732	253	0.00025	NE	0	NE	0	NE

Spy49_1522	-	1504006	1504473	90	0.99225	U	0	NE	0.99525	E
Spy49_1525	-	1504589	1505368	155	0.00025	NE	0	NE	0	NE
Spy49_1526	-	1505502	1505660	259	0.00025	NE	0	NE	0.00025	NE
Spy49_1528c	-	1506024	1506782	52	-1	S	-1	S	-1	S
Spy49_1529	-	1506855	1507277	252	0.00025	NE	1	E	0.93025	U
Spy49_1530	-	1507398	1508534	140	0	NE	0	NE	0	NE
Spy49_1531	-	1508573	1508893	378	0	NE	0	NE	0	NE
Spy49_1532	-	1508950	1509687	106	0	NE	0	NE	0	NE
Spy49_1533c	dnaQ	1509840	1510427	245	0	NE	0	NE	0.00025	NE
Spy49_1534c	-	1510476	1511057	195	0.00025	NE	0	NE	0.98775	U
Spy49_1535c	-	1511183	1511698	193	0.0005	NE	0	NE	0.836	U
Spy49_1536c	-	1511601	1512305	171	0	NE	0	NE	0	NE
Spy49_1537c	-	1512526	1513695	234	0	NE	0	NE	0.00025	NE
Spy49_1538	deoC	1513790	1514461	389	0.00025	NE	0	NE	0.00025	NE
Spy49_1540c	nupC	1514491	1515693	223	0	NE	0	NE	0	NE
Spy49_1541c	udp	1515714	1516493	400	0	NE	0	NE	0.00025	NE
Spy49_1542c	-	1516733	1517491	259	0.00025	NE	0	NE	0	NE
Spy49_1543	rpsN	1517700	1517969	252	0	NE	0	NE	0	NE
Spy49_1544	gcp	1518141	1519169	89	0	NE	0	NE	0	NE
Spy49_1545c	-	1519159	1519614	342	0.4205	U	1	E	0.98275	U
Spy49_1546c	-	1519586	1520284	151	0	NE	0	NE	0	NE
Spy49_1547c	-	1520568	1520798	232	0.29175	U	1	E	1	E
Spy49_1548	-	1520800	1522482	76	0	NE	1	E	0.00025	NE
Spy49_1549	glnA	1522710	1524056	560	1	E	1	E	1	E
Spy49_1550c	glnR	1524094	1524465	448	0.0005	NE	0	NE	0.00325	NE
Spy49_1551c	-	1524532	1525083	123	0	NE	0	NE	0	NE
Spy49_1552c	pgk	1525345	1526541	183	0	NE	0	NE	0.00025	NE
Spy49_1553c	lppC	1526726	1527580	398	0.9995	E	1	E	0.705	U
Spy49_1554c	-	1527820	1528701	284	0	NE	0	NE	0	NE
Spy49_1555c	-	1528938	1530602	293	0	NE	0	NE	0	NE
Spy49_1556c	asp	1530602	1530967	554	0.84	U	1	E	1	E
Spy49_1557c	rpmB	1531119	1531307	121	0.00025	NE	0.00075	NE	0.9865	U
Spy49_1558c	fba	1531689	1532570	62	0	NE	0	NE	0	NE
Spy49_1559c	-	1532916	1533842	293	1	E	1	E	1	E
Spy49_1560c	pyrG	1534010	1535614	308	0.00025	NE	0	NE	0	NE
Spy49_1561c	rpoE	1535871	1536479	534	0.00025	NE	0	NE	0.00025	NE
Spy49_1562c	tig	1536663	1537946	202	0	NE	0	NE	0	NE
Spy49_1563c	-	1538267	1539112	427	0.03825	NE	0	NE	0	NE
Spy49_1564	-	1539177	1539737	281	0.00025	NE	0	NE	0.00125	NE
Spy49_1565c	-	1539751	1540221	186	0	NE	0	NE	0	NE
Spy49_1566c	thiD	1540211	1540975	156	0	NE	0	NE	0	NE
Spy49_1567c	truA	1540965	1541714	254	0	NE	0	NE	0.00025	NE
Spy49_1568c	comX2	1542131	1542388	249	0	NE	0	NE	0	NE
Spy49_1569c	-	1548174	1549316	85	0	NE	0	NE	0	NE
Spy49_1571c	hsdR	1549597	1552575	380	0	NE	0	NE	0	NE
Spy49_1572	hsdS	1552588	1553730	992	0	NE	0	NE	0	NE
Spy49_1573	hsdM	1553743	1555323	380	0	NE	0	NE	0.00025	NE
Spy49_1574	-	1555533	1555733	526	0.00025	NE	0	NE	0	NE
Spy49_1575c	salR	1555876	1556481	66	0	NE	0	NE	0	NE
Spy49_1576c	salK	1556462	1557883	201	0	NE	0	NE	0	NE
Spy49_1577c	salY	1558064	1559971	473	0.00025	NE	0	NE	0	NE
Spy49_1578c	salX	1559973	1560710	635	0	NE	0	NE	0	NE
Spy49_1579c	salT	1560707	1561168	245	0	NE	0	NE	0.00025	NE
Spy49_1580c	salB	1561242	1562867	153	0	NE	0	NE	0	NE
Spy49_1582c	salA	1562951	1563097	541	0	NE	0	NE	0.00025	NE
Spy49_1583c	lacG	1563609	1565042	48	-1	S	-1	S	-1	S
Spy49_1584c	lacE	1565103	1566800	477	0	NE	0	NE	0	NE
Spy49_1585c	lacF	1566800	1567117	565	0	NE	0	NE	0	NE
Spy49_1586c	lacD2	1567141	1568124	105	0	NE	0	NE	0	NE
Spy49_1587c	lacC2	1568128	1569057	327	0	NE	0	NE	0	NE
Spy49_1588c	lacB2	1569103	1569618	309	0	NE	0	NE	0	NE
Spy49_1589c	lacA2	1569653	1570081	171	0	NE	0	NE	0.00075	NE
Spy49_1590c	lacR2	1570527	1571300	142	0	NE	0	NE	0	NE
Spy49_1591	-	1572028	1572249	257	0	NE	0	NE	0	NE
Spy49_1592	-	1572239	1572574	73	0	NE	0	NE	0	NE
Spy49_1593	-	1573340	1573660	111	0	NE	0	NE	0	NE
Spy49_1594	rpsI	1573828	1574220	106	0	NE	0	NE	0	NE
Spy49_1595c	rplM	1574241	1574687	130	0.063	U	0.99725	E	0.99175	E

Spy49_1596c	-	1574905	1575111	148	0.89875	U	0.99975	E	0.913	U
Spy49_1597c	-	1575108	1575806	68	0.01	NE	0.00025	NE	0.05825	U
Spy49_1598c	-	1575942	1576817	232	0	NE	0	NE	0.00025	NE
Spy49_1599c	-	1576898	1577380	291	0	NE	0	NE	0.3945	U
Spy49_1600c	-	1577419	1578165	160	0	NE	0	NE	0	NE
Spy49_1601c	-	1578212	1579009	248	0	NE	0	NE	0	NE
Spy49_1602c	-	1579205	1579618	265	0	NE	0	NE	0	NE
Spy49_1603c	cysS	1579611	1580954	137	0	NE	0	NE	0	NE
Spy49_1604c	-	1580982	1581212	447	1	E	1	E	1	E
Spy49_1605c	cysE	1581691	1582272	76	0	NE	0	NE	0	NE
Spy49_1606c	-	1582281	1583033	193	0.46375	U	0	NE	0	NE
Spy49_1607c	pnp	1583026	1585158	250	0.00025	NE	0	NE	0	NE
Spy49_1608c	-	1585438	1586166	710	0	NE	0	NE	0.02175	NE
Spy49_1609c	ulaA	1586179	1587558	242	0	NE	0	NE	0	NE
Spy49_1610c	-	1587585	1587869	459	0	NE	0	NE	0.00025	NE
Spy49_1611c	-	1587862	1589922	94	0	NE	0	NE	0.00025	NE
Spy49_1612c	rpsO	1590275	1590544	686	0	NE	0	NE	0	NE
Spy49_1613c	-	1590720	1590920	89	0	NE	0	NE	0.96975	U
Spy49_1614c	def	1591319	1591933	66	0	NE	0	NE	0	NE
Spy49_1615	-	1591967	1592509	204	0	NE	1	E	0.985	U
Spy49_1616c	-	1592640	1593068	180	0	NE	0	NE	0	NE
Spy49_1617c	polC	1593178	1597575	142	0	NE	0	NE	0	NE
Spy49_1618c	proS	1597830	1599686	1465	0.99975	E	1	E	1	E
Spy49_1619c	eep	1599884	1601143	618	0.9995	E	1	E	1	E
Spy49_1620c	cdsA	1601216	1602010	419	0	NE	0	NE	0.00025	NE
Spy49_1621c	uppS	1602023	1602772	264	0.84425	U	1	E	1	E
Spy49_1622c	yajC	1602991	1603356	249	0.95275	U	1	E	1	E
Spy49_1624c	-	1603472	1603819	121	0	NE	0	NE	0	NE
Spy49_1625c	pulA	1603961	1607365	115	0	NE	0	NE	0	NE
Spy49_1626c	dexB	1607656	1609269	1134	0	NE	0	NE	0	NE
Spy49_1627c	msmK	1609398	1610531	537	0	NE	0	NE	0	NE
Spy49_1628c	lrp	1610829	1611722	377	0.00025	NE	0	NE	0	NE
Spy49_1629c	ska	1612017	1613339	297	0	NE	0	NE	0	NE
Spy49_1630	dtd	1613437	1613880	440	0	NE	0	NE	0	NE
Spy49_1631c	relA	1613895	1616114	147	0	NE	0	NE	0	NE
Spy49_1632c	sclA	1616358	1617626	739	0.18525	U	0	NE	1	E
Spy49_1633c	nrdI2	1618008	1618490	422	0.00025	NE	0	NE	0.00025	NE
Spy49_1634	-	1618883	1619701	160	0	NE	0	NE	0	NE
Spy49_1635c	-	1619784	1621970	272	0	NE	0	NE	0	NE
Spy49_1636c	-	1622326	1623075	728	0.00025	NE	0	NE	0.0165	NE
Spy49_1637c	prmA	1623075	1624028	249	0	NE	0	NE	0.004	NE
Spy49_1638c	nudC	1624045	1625127	317	0.00025	NE	0	NE	0.00025	NE
Spy49_1639c	nudB	1625304	1625660	360	0.00025	NE	0	NE	0.00025	NE
Spy49_1640c	nudA	1625688	1626005	118	0	NE	0	NE	0	NE
Spy49_1641c	-	1626019	1626489	105	0	NE	0	NE	0	NE
Spy49_1642c	-	1626554	1626673	156	0	NE	0	NE	0	NE
Spy49_1643c	-	1626954	1627532	39	-1	S	-1	S	-1	S
Spy49_1644c	-	1627693	1627983	192	0.00025	NE	0	NE	0	NE
Spy49_1646c	-	1628038	1628844	96	0	NE	0	NE	0	NE
Spy49_1647c	-	1629499	1629813	268	0	NE	0	NE	0	NE
Spy49_1649c	-	1630202	1630438	104	0	NE	0	NE	0	NE
Spy49_1651	-	1630510	1630704	78	0	NE	0	NE	0	NE
Spy49_1652	papB	1630830	1632587	64	0.5285	U	0	NE	0	NE
Spy49_1653	trpG	1632620	1633186	585	0	NE	0	NE	0	NE
Spy49_1654	-	1633219	1634487	188	0	NE	0	NE	0	NE
Spy49_1655	pai1	1634985	1635425	422	0	NE	0	NE	0	NE
Spy49_1656	-	1635460	1635966	146	0	NE	0	NE	0.00025	NE
Spy49_1657	-	1636090	1636425	168	0	NE	0	NE	0	NE
Spy49_1658c	-	1636435	1637079	111	0.00025	NE	0	NE	0.00025	NE
Spy49_1659c	dppA	1638159	1639649	214	0	NE	0	NE	0	NE
Spy49_1660	dppB	1639762	1640739	496	0	NE	0	NE	0	NE
Spy49_1661	dppC	1640736	1641557	325	0	NE	0	NE	0.0005	NE
Spy49_1662	dppD	1641569	1642372	273	0	NE	0	NE	0.00025	NE
Spy49_1663	dppF	1642356	1642982	267	0	NE	0	NE	0	NE
Spy49_1664	-	1643064	1643264	208	0	NE	0	NE	0	NE
Spy49_1665c	-	1643433	1645904	66	0	NE	0	NE	0	NE
Spy49_1666c	lmb	1645917	1646837	823	0	NE	0	NE	0.00025	NE
Spy49_1667c	-	1647073	1648410	306	0	NE	0	NE	0.00025	NE

Spy49_1668c	scpA	1648661	1652050	445	0	NE	0	NE	0.00025	NE
Spy49_1669c	ennX	1652385	1653491	1129	0	NE	0	NE	0.00025	NE
Spy49_1670c	emm49	1653701	1654858	368	0	NE	0	NE	0	NE
Spy49_1671c	-	1655083	1656330	385	0.00025	NE	0	NE	0.00025	NE
Spy49_1672c	mga	1656520	1658121	415	0	NE	0	NE	0	NE
Spy49_1673c	-	1658797	1659048	533	0	NE	0	NE	0	NE
Spy49_1674c	isp1	1659126	1660754	83	0	NE	0	NE	0	NE
Spy49_1675c	-	1660856	1662244	542	0	NE	0	NE	0	NE
Spy49_1676c	-	1662241	1662894	462	0	NE	0	NE	0.00025	NE
Spy49_1677c	-	1662988	1664205	217	0	NE	0	NE	0	NE
Spy49_1678c	-	1664218	1664892	405	0	NE	0	NE	0	NE
Spy49_1679c	-	1664879	1666147	224	0.00025	NE	0	NE	0	NE
Spy49_1680c	-	1666571	1666975	422	0	NE	0	NE	0	NE
Spy49_1681c	-	1667002	1667298	134	0.00025	NE	0	NE	0.00025	NE
Spy49_1682c	sfbX49	1667440	1669395	98	0	NE	0	NE	0	NE
Spy49_1683c	sof	1669575	1672655	651	0	NE	0	NE	0.00025	NE
Spy49_1684c	-	1673078	1673314	1026	0.00025	NE	0	NE	0.00175	NE
Spy49_1686	ropA	1673590	1674540	78	0	NE	0	NE	0.0005	NE
Spy49_1687c	-	1674572	1674889	316	0	NE	0	NE	0.4775	U
Spy49_1688c	-	1674997	1675302	105	0	NE	0	NE	0	NE
Spy49_1689c	speB	1675304	1676500	101	0	NE	0	NE	0.996	E
Spy49_1690c	ropB	1677441	1678283	398	0	NE	0	NE	0	NE
Spy49_1691	mf1	1678512	1679342	280	0	NE	0	NE	0	NE
Spy49_1692c	-	1679426	1679554	276	0	NE	0	NE	0.00025	NE
Spy49_1693	-	1679707	1680216	42	-1	S	-1	S	-1	S
Spy49_1694	gldA	1680285	1681373	169	0	NE	0	NE	0	NE
Spy49_1695c	mipB	1681430	1682098	362	0	NE	0	NE	0	NE
Spy49_1696c	pflD	1682111	1684528	222	0	NE	0	NE	0	NE
Spy49_1697c	-	1684739	1686043	805	0	NE	0	NE	0	NE
Spy49_1698c	-	1686053	1686361	434	0	NE	0	NE	0.00025	NE
Spy49_1699c	-	1686389	1686709	102	0	NE	0	NE	0.00025	NE
Spy49_1700c	-	1686996	1687976	106	0	NE	0	NE	0	NE
Spy49_1701c	-	1687992	1688741	326	0	NE	0	NE	0.00025	NE
Spy49_1702c	-	1688864	1689637	249	0	NE	0	NE	0	NE
Spy49_1703	secE	1689873	1690049	257	0	NE	0	NE	0.00025	NE
Spy49_1704c	rpmG	1690063	1690215	58	0.97525	U	1	E	0.97625	U
Spy49_1705c	pbp2A	1690264	1692600	50	-1	S	-1	S	-1	S
Spy49_1706c	-	1692639	1693019	778	0.00025	NE	0	NE	0.04025	U
Spy49_1707c	-	1693510	1693701	126	0	NE	0	NE	0	NE
Spy49_1708	-	1693595	1694596	63	0	NE	0	NE	0	NE
Spy49_1709	-	1694685	1696409	333	0	NE	0	NE	0	NE
Spy49_1710c	-	1696572	1698071	574	0	NE	0	NE	0	NE
Spy49_1713	-	1698783	1699130	499	0	NE	0	NE	0	NE
Spy49_1714c	groEL	1699290	1700921	115	0	NE	0	NE	0	NE
Spy49_1715c	groES	1700957	1701247	543	0.9935	U	1	E	1	E
Spy49_1716c	clpC	1701425	1703869	96	0.98425	U	1	E	0.98	U
Spy49_1717c	ctsR	1703869	1704330	814	0.00025	NE	0	NE	0.00025	NE
Spy49_1718c	csp	1704526	1704735	153	0.00025	NE	0	NE	0	NE
Spy49_1719c	-	1705476	1706609	69	0.0135	NE	0	NE	0	NE
Spy49_1720c	ahpC	1707061	1707621	377	0	NE	0	NE	0	NE
Spy49_1721	nox3	1707642	1709174	186	0	NE	0	NE	0	NE
Spy49_1722	hutI	1709232	1710497	510	0	NE	0	NE	0	NE
Spy49_1723c	hutU	1710789	1712819	421	0	NE	0	NE	0	NE
Spy49_1724	-	1712907	1713806	676	0	NE	0	NE	0	NE
Spy49_1725	-	1713817	1714443	299	0	NE	0	NE	0.00025	NE
Spy49_1726	fhs2	1714461	1716134	208	0	NE	0	NE	0	NE
Spy49_1727	-	1716155	1716751	557	0	NE	0	NE	0.61225	U
Spy49_1728	-	1716971	1718314	198	0	NE	0	NE	0	NE
Spy49_1729	hutH	1718326	1719867	447	0	NE	0	NE	0.00025	NE
Spy49_1730	hutG	1720054	1721040	513	0	NE	0	NE	0	NE
Spy49_1731	-	1721071	1724145	328	0	NE	0	NE	0	NE
Spy49_1732c	rpsB	1724449	1725216	1024	0.00075	NE	0	NE	0	NE
Spy49_1733	tsf	1725350	1726390	255	1	E	1	E	0.99925	E
Spy49_1734	pepO	1726556	1728451	346	0.65075	U	1	E	0.997	E
Spy49_1735c	dexS	1728659	1730287	631	0	NE	0	NE	0.1435	U
Spy49_1736c	-	1730354	1732378	542	1	E	1	E	1	E
Spy49_1737c	-	1732589	1733302	674	0	NE	0	NE	0.5975	U
Spy49_1738	-	1733581	1733820	237	0.00025	NE	0	NE	0.0005	NE

Spy49_1739c	-	1734105	1734962	79	0	NE	0	NE	0	NE
Spy49_1740	-	1735004	1735735	285	0.00025	NE	0	NE	0.00025	NE
Spy49_1742	nrdG	1735909	1736523	243	0.00025	NE	0	NE	0	NE
Spy49_1743c	-	1736523	1737032	204	0.00025	NE	0	NE	0.91675	U
Spy49_1744c	-	1737041	1737976	169	0.00025	NE	0	NE	0.00025	NE
Spy49_1745c	-	1738005	1738151	311	0.00025	NE	1	E	1	E
Spy49_1746c	nrdD	1738333	1740531	48	-1	S	-1	S	-1	S
Spy49_1747c	-	1740628	1742187	732	0.00025	NE	0	NE	0.629	U
Spy49_1748c	-	1742600	1742905	519	0.00025	NE	0	NE	0	NE
Spy49_1749c	-	1742917	1743336	101	0	NE	0	NE	0.00025	NE
Spy49_1750c	-	1743333	1743602	139	0.00025	NE	0.19025	U	0.98825	U
Spy49_1751c	spxA	1743716	1744114	89	0.00025	NE	0	NE	0.00025	NE
Spy49_1752c	recA	1744405	1745541	132	0.0005	NE	0.00075	NE	0.0005	NE
Spy49_1753c	cinA	1745629	1746900	378	0	NE	0	NE	0.984	U
Spy49_1754c	tag	1746969	1747529	423	0	NE	0	NE	0.00025	NE
Spy49_1755c	ruvA	1747539	1748135	186	0	NE	0	NE	0	NE
Spy49_1756c	lmrP	1748137	1749357	198	0.00375	NE	0.92175	U	0.99825	E
Spy49_1757c	mutL	1749368	1751350	406	0	NE	0	NE	0.99275	E
Spy49_1758c	mutS	1751480	1754035	660	0.32375	U	0	NE	1	E
Spy49_1759c	-	1754371	1754706	851	0.92175	U	0	NE	0.2075	U
Spy49_1761c	argS	1755099	1756790	111	0	NE	0	NE	0	NE
Spy49_1762	-	1756878	1757186	563	1	E	1	E	1	E
Spy49_1763	-	1757213	1758085	102	0	NE	0	NE	0	NE
Spy49_1764c	-	1758128	1759063	290	0	NE	0	NE	0	NE
Spy49_1765c	-	1759026	1759967	311	0.0005	NE	0	NE	0	NE
Spy49_1767c	aspS	1759960	1761708	313	0.00025	NE	0	NE	0.00025	NE
Spy49_1768c	hisS	1762046	1763326	582	1	E	1	E	1	E
Spy49_1769c	rpmF	1763546	1763728	426	1	E	1	E	1	E
Spy49_1770	rpmG	1763744	1763893	60	0	NE	0	NE	0	NE
Spy49_1771	-	1763933	1764157	49	-1	S	-1	S	-1	S
Spy49_1772c	cadD	1764186	1764800	74	0.00025	NE	0	NE	0	NE
Spy49_1773	cadX	1764812	1765150	204	0.00025	NE	0	NE	0	NE
Spy49_1774	-	1765194	1766117	112	0	NE	0	NE	0	NE
Spy49_1775	-	1766183	1766935	307	0.00025	NE	0	NE	0.00025	NE
Spy49_1777	-	1766932	1767534	250	0	NE	0	NE	0	NE
Spy49_1778	-	1768745	1769587	200	0.00025	NE	0	NE	0.00075	NE
Spy49_1779	-	1769591	1770274	280	0.00025	NE	0	NE	0.0075	NE
Spy49_1780c	-	1770481	1770807	227	0.00475	NE	0	NE	0.00025	NE
Spy49_1781	-	1770794	1771381	108	0.00025	NE	0	NE	0	NE
Spy49_1782	-	1771378	1772451	195	0	NE	0	NE	0.00025	NE
Spy49_1783	-	1772567	1774861	357	0.00025	NE	0	NE	0.00025	NE
Spy49_1784c	-	1774975	1775511	764	0.00025	NE	0	NE	0.36525	U
Spy49_1785	rpsD	1775661	1776272	178	0	NE	0	NE	0	NE
Spy49_1786c	-	1777001	1777273	203	0.99975	E	1	E	0.99925	E
Spy49_1787c	dnaC	1777290	1778657	90	0.0035	NE	0.00025	NE	0.00125	NE
Spy49_1788c	rplI	1778687	1779139	455	1	E	1	E	1	E
Spy49_1789c	-	1779136	1781112	150	0.00025	NE	0	NE	0.04825	U
Spy49_1790c	gidA	1781202	1783100	658	0.98	U	1	E	1	E
Spy49_1791c	-	1783224	1783541	632	0.00275	NE	1	E	1	E
Spy49_1792c	mnmA	1784316	1785437	105	0.00025	NE	0	NE	0	NE
Spy49_1793c	sdhB	1785735	1786406	373	0.9795	U	1	E	1	E
Spy49_1794	sdhA	1786418	1787290	223	0.00025	NE	0	NE	0	NE
Spy49_1795	-	1787704	1788318	290	0	NE	0	NE	0.00675	NE
Spy49_1796c	-	1788690	1789490	204	0	NE	0	NE	0	NE
Spy49_1797c	cbiO	1789483	1790325	266	0.9985	E	1	E	1	E
Spy49_1798c	cbiO	1790301	1791191	280	0.9845	U	1	E	1	E
Spy49_1799c	pgsA	1791142	1791684	296	0.99975	E	0.99375	E	0.99975	E
Spy49_1800c	-	1791698	1792723	180	0.99975	E	1	E	1	E
Spy49_1801c	-	1792773	1794062	341	0	NE	0	NE	0	NE
Spy49_1802c	-	1794064	1795308	429	0	NE	0	NE	0	NE
Spy49_1803c	hasA	1795670	1797007	414	0	NE	0	NE	0	NE
Spy49_1804	hasB	1797043	1798251	445	0.00025	NE	0	NE	0	NE
Spy49_1806	hasC	1798433	1799347	402	0.00025	NE	0	NE	0	NE
Spy49_1808	-	1799655	1800068	304	0.0005	NE	0	NE	0.00125	NE
Spy49_1809	recF	1800070	1801176	137	0	NE	0	NE	0	NE
Spy49_1810	-	1801232	1802095	368	0.0005	NE	0	NE	0.67575	U
Spy49_1811c	guaB	1802298	1803779	287	0	NE	0	NE	0	NE
Spy49_1812c	trsA	1804087	1805109	493	0.00025	NE	0	NE	0	NE

Spy49_1813c	-	1805528	1806400	340	1	E	1	E	1	E
Spy49_1814	-	1806479	1808086	290	0	NE	0	NE	0	NE
Spy49_1815	-	1808184	1810760	535	0	NE	0	NE	0	NE
Spy49_1817	-	1813005	1813484	858	0.00225	NE	0.00025	NE	0.017	NE
Spy49_1819c	degP	1813696	1814919		0	NE	0	NE	0.00025	NE
Spy49_1820c	parB	1814978	1815784	159	0	NE	0	NE	0	NE
Spy49_1821				407	0.9795	U	1	E	0.99975	E
Spy49_1822				268	0.00025	NE	0	NE	0.00025	NE

Table S6. Integrated gene essentiality determination for GAS M49 NZ131 for all time points.

(a) Spy numbers from GAS NZ131 genome.
(b) When available, gene name is provided.
(c) Call integrating data from Bayesian analyses on 3 time points.
(d) Hyperlink to the NCBI Gene website (http://www.ncbi.nlm.nih.gov/gene).

Locus Tag(a)	Gene Name(b)	Call(c)	Start	Stop	Length	Strand	Gene ID(d)	COG Designation	Protein Function
Spy49_0001	dnaA	NC	232	1587	451	+	6986144	COG0593L	chromosomal replication initiation protein
Spy49_0002	dnaN	E	1742	2878	378	+	6984517	COG0592L	DNA polymerase III subunit beta
Spy49_0003	-	NE	2953	3150	65	+	6984518	COG4481S	hypothetical protein Spy49_0003
Spy49_0004	-	NE	3480	4595	371	+	6984519	COG0012J	GTP-dependent nucleic acid-binding protein EngD
Spy49_0005	pth	E	4665	5234	189	+	6984520	COG0193J	peptidyl-tRNA hydrolase
Spy49_0006	trcF	NE	5321	8740	1139	+	6984521	COG1197LK	transcription-repair coupling factor
Spy49_0008	-	NE	8902	9174	90	+	6984522	COG1188J	hypothetical protein Spy49_0008
Spy49_0009	divIC	NC	9161	9532	123	+	6984523	COG2919D	cell division protein
Spy49_0010	-	NE	9667	10953	428	+	6984524	COG2367V	hypothetical protein Spy49_0010
Spy49_0011	mesJ	E	10950	12236	428	+	6984525	COG0037D	tRNA(Ile)-lysidine synthetase
Spy49_0012	hpt	NE	12241	12783	180	+	6984526	COG0634F	Hypoxanthine-guanine phosphoribosyltransferase
Spy49_0013	ftsH	E	12805	14784	659	+	6984527	COG0465O	cell division protein FtsH
Spy49_0014	-	NE	15041	16501	486	+	6984528	COG0531E	amino acid permease
Spy49_0015	-	E	31169	32365	398	+	6984563	COG3883S	secreted protein strept secreted protein
Spy49_0016	prsA	E	32618	33580	320	+	6984564	COG0462FE	ribose-phosphate pyrophosphokinase
Spy49_0017	recO	NE	33766	34521	251	+	6984565	COG1381L	DNA repair protein RecO
Spy49_0018	-	E	34624	35631	335	+	6984566	COG0416I	glycerol-3-phosphate acyltransferase PlsX
Spy49_0019	acp	NC	35624	35866	80	+	6984567	COG0236IQ	acyl carrier protein
Spy49_0020	purC	NE	35987	36721	244	+	6984568	COG0152F	phosphoribosylaminoimidazole-succinocarboxamide synthase
Spy49_0021	purL	NE	36798	40571	1257	+	6984569	COG0046F	phosphoribosylformylglycinamidine synthase, synthetase subunit/phosphoribosylformylglycinamidine syn...
Spy49_0022	purF	NE	40805	42259	484	+	6984570	COG0034F	amidophosphoribosyltransferase
Spy49_0023	purM	NE	42287	43309	340	+	6984571	COG0150F	phosphoribosylaminoimidazole synthetase
Spy49_0024	purN	NE	43477	44031	184	+	6984572	COG0299F	phosphoribosylglycinamide formyltransferase
Spy49_0025	purH	NE	44215	45762	515	+	6984573	COG0138F	bifunctional phosphoribosylaminoimidazolecarboxamide formyltransferase/IMP cyclohydrolase
Spy49_0026c	-	NE	45820	46944	374	-	6984574	COG3942R	Autolysin
Spy49_0027	purD	NE	47107	48462	451	+	6984575	COG0151F	phosphoribosylamine--glycine ligase
Spy49_0028	purE	NE	48621	49232	203	+	6984576	COG0041F	phosphoribosylaminoimidazole carboxylase catalytic subunit
Spy49_0029	purK	NE	49216	50292	358	+	6984577	COG0026F	phosphoribosylaminoimidazole carboxylase ATPase subunit
Spy49_0030	-	NE	50394	51875	493	+	6984578	-	hypothetical protein Spy49_0030
Spy49_0031	purB	NE	52048	53340	430	+	6984579	COG0015F	adenylosuccinate lyase
Spy49_0032	-	NE	53471	54391	306	+	6984580	-	Cro/CI family transcriptional regulator
Spy49_0033	ruvB	C	54617	55615	332	+	6984581	COG2255L	Holliday junction DNA helicase RuvB
Spy49_0035	-	NE	55753	56190	145	+	6984582	COG0394T	protein tyrosine phosphatase
Spy49_0036	-	E	56213	56614	133	+	6984663	COG4642S	hypothetical protein Spy49_0036
Spy49_0037	-	E	56611	58386	591	+	6984664	COG1835I	acyltransferase
Spy49_0038	adhE	NE	58695	61337	880	+	6984665	COG1454C	bifunctional acetaldehyde-CoA/alcohol dehydrogenase
Spy49_0039	adhP	NE	61589	62605	338	+	6984666	COG1064R	alcohol dehydrogenase
Spy49_0040	norM	NE	62993	64282	429	+	6984667	COG0534V	Na+ driven multidrug efflux pump
Spy49_0044	rpsJ	NC	64481	64789	102	+	6984668	COG0051J	30S ribosomal protein S10
Spy49_0045	rplC	E	65037	65663	208	+	6984669	COG0087J	50S ribosomal protein L3
Spy49_0046	rplD	NC	65687	66310	207	+	6984670	COG0088J	50S ribosomal protein L4
Spy49_0048	rplW	NC	66310	66540	76	+	6984671	COG0089J	50S ribosomal protein L23p
Spy49_0049	rplB	E	66625	67458	277	+	6984672	COG0090J	50S ribosomal protein L2
Spy49_0050	rpsS	NC	67597	67875	92	+	6984673	COG0185J	30S ribosomal protein S19
Spy49_0052	rplV	NC	67891	68235	114	+	6984674	COG0091J	50S ribosomal protein L22
Spy49_0053	rpsC	E	68275	68901	208	+	6984675	COG0092J	30S ribosomal protein S3
Spy49_0054	rplP	E	68905	69318	137	+	6984676	COG0197J	50S ribosomal protein L16
Spy49_0055	rpmC	NC	69328	69534	68	+	6984677	COG0255J	50S ribosomal protein L29
Spy49_0056	rpsQ	NC	69560	69820	86	+	6984678	COG0186J	30S ribosomal protein S17
Spy49_0057	rplN	NC	69845	70213	122	+	6984679	COG0093J	50S ribosomal protein L14
Spy49_0058	rplX	E	70292	70597	101	+	6984680	COG0198J	50S ribosomal protein L24
Spy49_0059	rplE	NC	70621	71163	180	+	6984681	COG0094J	50S ribosomal protein L5
Spy49_0060	rpsN.1	NC	71179	71364	61	+	6984682	COG0199J	30S ribosomal protein S14p
Spy49_0061	rpsH	NC	71515	71913	132	+	6984683	COG0096J	30S ribosomal protein S8
Spy49_0062	rplF	E	72116	72652	178	+	6984684	COG0097J	50S ribosomal protein L6
Spy49_0064	rplR	NC	72757	73113	118	+	6984685	COG0256J	50S ribosomal protein L18
Spy49_0065	rpsE	NC	73132	73626	164	+	6984686	COG0098J	30S ribosomal protein S5
Spy49_0066	rpmD	NC	73641	73823	60	+	6984687	COG1841J	50S ribosomal protein L30
Spy49_0067	rplO	NC	74038	74478	146	+	6984688	COG0200J	50S ribosomal protein L15
Spy49_0068	secY	E	74495	75799	434	+	6984689	COG0201U	preprotein translocase subunit SecY
Spy49_0069	adk	E	75949	76587	212	+	6984690	COG0563F	adenylate kinase
Spy49_0070	infA	NC	76705	76923	72	+	6984691	COG0361J	translation initiation factor IF-1
Spy49_0071	rpmJ	NC	76949	77065	38	+	6984692	COG0257J	50S ribosomal protein L36
Spy49_0072	rpsM	E	77083	77448	121	+	6984693	COG0099J	30S ribosomal protein S13
Spy49_0073	rpsK	NC	77466	77849	127	+	6984694	COG0100J	30S ribosomal protein S11
Spy49_0074	rpoA	E	77895	78833	312	+	6984695	COG0202K	DNA-directed RNA polymerase subunit alpha
Spy49_0075	rplQ	NC	78848	79234	128	+	6984696	COG0203J	50S ribosomal protein L17
Spy49_0078	-	NE	87367	87615	82	+	6984713	COG1947I	4-diphosphocytidyl-2-C-methyl-D-erythritol kinase
Spy49_0079	-	NE	87670	87882	70	+	6984714	COG1947I	4-diphosphocytidyl-2-C-methyl-D-erythritol kinase
Spy49_0080	adrR	NE	87992	88435	147	+	6984715	COG1846K	repressor protein
Spy49_0081	adrC	NC	88439	89158	239	+	6984716	COG1121P	ABC transporter
Spy49_0082	adrB	NC	89151	89966	271	+	6984717	COG1108P	ABC transporter
Spy49_0083c	-	NE	90006	90389	127	-	6984718	COG0537FGR	histidine triad (HIT) protein
Spy49_0084	tyrS	E	90440	91696	418	-	6984719	COG0162J	tyrosyl-tRNA synthetase
Spy49_0086	pbp1b	NE	91800	94100	766	+	6984720	COG0744M	multimodular transpeptidase-transglycosylase
Spy49_0087	rpoB	E	94364	97930	1188	+	6984721	COG0085K	DNA-directed RNA polymerase subunit beta
Spy49_0088	rpoC	E	98048	101662	1204	+	6984722	COG0086K	DNA-directed RNA polymerase subunit beta\'
Spy49_0089	-	NE	101814	102179	121	+	6984723	COG4699S	DNA binding protein
Spy49_0090	comYA	NE	102272	103210	312	+	6984724	COG2804NU	ABC transporter
Spy49_0091	comYB	NE	103305	104180	291	+	6984725	COG1459NU	competence protein, ABC transporter subunit
Spy49_0092	comYC	NE	104182	104508	108	+	6984726	COG4537U	competence protein
Spy49_0093	-	NE	104483	104911	142	+	6984727	COG2165NU	Competence protein
Spy49_0094	-	NE	104868	105152	94	+	6984728	-	hypothetical protein Spy49_0094
Spy49_0095	comYD	NE	105145	105579	144	+	6984729	COG4940U	competence protein
Spy49_0096	-	NE	105599	105889	96	+	6984730	-	hypothetical protein Spy49_0096
Spy49_0097	-	NE	105987	106940	317	+	6984731	COG0827L	adenine-specific methyltransferase
Spy49_0098	ackA	NC	106999	108195	398	+	6984732	COG0282C	acetate kinase
Spy49_0099	-	NE	108381	108623	80	+	6984733	-	hypothetical protein Spy49_0099
Spy49_0100	proC	NE	108772	109542	256	-	6984734	COG0345E	pyrroline-5-carboxylate reductase
Spy49_0101	pepA	NE	109590	110657	355	-	6984735	COG1363G	glutamyl-aminopeptidase
Spy49_0103	-	NE	112439	112732	97	+	6984736	-	hypothetical protein Spy49_0103
Spy49_0104	trx.1	NE	112729	113046	105	+	6984737	COG0526OC	thioredoxin
Spy49_0105	-	NE	113064	113708	214	+	6984738	COG0073R	tRNA binding domain-containing protein
Spy49_0107	ssb	NE	113842	114237	131	+	6984739	COG0629L	single-stranded DNA-binding protein
Spy49_0108c	-	NE	114497	115138	213	-	6984740	COG1428F	deoxyadenosine kinase/deoxyguanosine kinase
Spy49_0109c	-	NE	115158	116135	325	-	6984741	COG0042J	transcriptional regulator
Spy49_0110c	hslO	NE	116122	116994	290	-	6984742	COG1281O	Hsp33-like chaperonin
Spy49_0111	nra	NE	117120	118655	511	-	6984823	-	Regulatory protein
Spy49_0112	cbp	NE	119085	121319	744	-	6984824	-	collagen binding protein
Spy49_0113	lepA-1	NE	121312	121833	173	+	6984825	COG4959OU	signal peptidase I
Spy49_0114	-	NE	121855	122889	344	+	6984826	-	hypothetical protein Spy49_0114

Spy49_0116	-	NE	122905	123630	241	+	6984827	COG4509S	NPQTN specific sortase B
Spy49_0117	-	NE	123665	124234	189	+	6984828	-	hypothetical protein Spy49_0117
Spy49_0118	msmR	NE	124394	125599	401	-	6984829	COG2207K	AraC family transcriptional regulator
Spy49_0119	prtF	NE	125984	129466	1160	+	6984830	COG4932M	fibronectin binding protein
Spy49_0120c	-	NE	129731	130396	221	-	6984831	-	hypothetical protein Spy49_0120c
Spy49_0121	atoE	NE	130750	132156	468	+	6984832	COG2031I	short-chain fatty acids transporter
Spy49_0122c	-	NE	132231	133142	303	-	6984833	COG0583K	transcriptional regulator
Spy49_0123	-	NE	133263	134447	394	+	6984834	COG0183I	acetyl-CoA acetyltransferase
Spy49_0124	atoD.2	NE	134459	135118	219	+	6984835	COG1788I	acetate CoA-transferase subunit alpha
Spy49_0125	-	NE	135121	135768	215	+	6984836	COG2057I	Butyrate-acetoacetate CoA-transferase subunit beta
Spy49_0126c	-	NE	135890	136570	226	-	6984837	COG2964S	transcriptional regulator
Spy49_0127	-	NE	136745	137110	121	+	6984838	COG0251J	translation initiation inhibitor
Spy49_0128	sloR	NE	137147	138166	339	+	6984839	COG1299G	regulatory protein
Spy49_0129	-	NE	138620	138940	106	+	6984840	-	hypothetical protein Spy49_0129
Spy49_0130	ntpI	NE	138930	140930	666	+	6984841	COG1269C	V-type ATP synthase subunit I
Spy49_0131	ntpK	NE	140932	141411	159	+	6984842	COG0636C	V-type ATP synthase subunit K
Spy49_0132	ntpE	NE	141479	142063	194	+	6984843	-	V-type Na+-ATPase subunit E
Spy49_0133	ntpC	NE	142079	143077	332	+	6984844	COG1527C	V-type sodium ATP synthase subunit C
Spy49_0134	ntpG	NE	143074	143394	106	+	6984845	COG1436C	V-type ATP synthase subunit F
Spy49_0135	ntpA	NE	143595	145370	591	+	6984846	COG1155C	V-type ATP synthase subunit A
Spy49_0136	ntpB	NE	145371	146786	471	+	6984847	COG1156C	V-type ATP synthase subunit B
Spy49_0137	ntpD	NE	146831	147457	208	+	6984848	COG1394C	V-type ATP synthase subunit D
Spy49_0138c	-	NE	147577	148839	420	-	6984849	COG3853P	toxic anion resistance protein
Spy49_0139c	-	NE	148852	149730	292	-	6984850	-	hypothetical protein Spy49_0139c
Spy49_0141	purA	NE	150168	151460	430	+	6984851	COG0104F	adenylosuccinate synthetase
Spy49_0142	-	NE	151787	152830	347	+	6984852	COG1744R	ABC transporter
Spy49_0143	nusG	NC	152988	153542	184	+	6984853	COG0250K	transcription antitermination protein NusG
Spy49_0144	nga	NE	153917	155272	451	+	6984854	-	Nicotine adenine dinucleotide glycohydrolase (NADGH)
Spy49_0145	-	NE	155277	155762	161	+	6984855	-	hypothetical protein Spy49_0145
Spy49_0146	slo	NE	155786	157501	571	+	6984856	-	Thiol-activated cytolysin
Spy49_0147	-	NC	157756	158184	142	+	6984857	-	hypothetical protein Spy49_0147
Spy49_0148	-	NC	158408	158701	97	+	6984858	-	hypothetical protein Spy49_0148
Spy49_0149c	-	NE	158998	159165	55	-	6984859	-	hypothetical protein Spy49_0149c
Spy49_0150c	-	E	159350	159676	108	-	6984860	-	hypothetical protein Spy49_0150c
Spy49_0151	metB	NE	159929	161299	456	+	6984861	COG0626E	cystathionine beta-lyase
Spy49_0152	leuS	E	161510	164011	833	+	6984862	COG0495J	leucyl-tRNA synthetase
Spy49_0153	-	NE	164318	165751	477	+	6984863	COG3037S	PTS system ascorbate-specific transporter subunit IIC
Spy49_0154	-	NE	165822	166100	92	+	6984864	COG3414G	PTS system IIB component
Spy49_0155	-	NE	166223	166708	161	+	6984865	COG1762GT	PTS system IIA component
Spy49_0156	ulaD	NC	166799	167461	220	+	6984866	COG0269G	3-keto-L-gulonate-6-phosphate decarboxylase
Spy49_0157	-	NE	167466	168329	287	+	6984867	COG3623G	L-xylulose 5-phosphate 3-epimerase
Spy49_0158	araD	NE	168331	169035	234	+	6984868	COG0235G	L-ribulose-5-phosphate 4-epimerase
Spy49_0159	-	NE	169361	171007	548	+	6984869	COG3711K	transcriptional regulator
Spy49_0160	-	NE	171261	172352	363	+	6984870	COG2220R	L-ascorbate 6-phosphate lactonase
Spy49_0161	opuAA	NE	172842	174038	398	+	6984871	COG4175E	L-proline glycine betaine ABC transport ATP- binding protein proV
Spy49_0162	opuABC	NE	174054	175781	575	+	6984872	COG4176E	glycine/betaine ABC transporter permease
Spy49_0163	polA	E	175912	178554	880	+	6984873	COG0749L	DNA polymerase I
Spy49_0164	-	NE	178732	179187	151	+	6984874	COG1832R	CoA-binding domain-containing protein
Spy49_0165	-	NE	179239	179706	155	+	6984875	COG0735P	ferric transport regulator protein
Spy49_0166	-	NE	179862	180161	99	+	6984876	-	M protein trans-acting positive regulator-like protein
Spy49_0167	-	NE	180383	181717	444	+	6984877	COG3969R	hypothetical protein Spy49_0167
Spy49_0168	-	NE	181710	182090	126	+	6984878	COG1475K	hypothetical protein Spy49_0168
Spy49_0169	-	NE	182234	182443	69	+	6984879	-	hypothetical protein Spy49_0169
Spy49_0170c	-	NE	182570	182785	71	-	6984880	COG2801L	transposase
Spy49_0173c	-	NE	183427	183720	97	-	6984881	COG2963L	hypothetical protein Spy49_0173c
Spy49_0176	-	NE	185332	186204	290	+	6984883	COG0157H	nicotinate-nucleotide pyrophosphorylase
Spy49_0178c	-	NE	186865	187311	148	-	6984884	COG2801L	transposase, IS861
Spy49_0180c	-	NE	187933	188796	287	-	6984885	COG5523S	hypothetical protein Spy49_0180c
Spy49_0181	tgt	NE	189015	190157	380	+	6984886	COG0343J	queuine tRNA-ribosyltransferase
Spy49_0182	-	NE	190374	190685	103	+	6984887	COG4357S	hypothetical protein Spy49_0182
Spy49_0183	bioY	NE	190689	191228	179	+	6984888	COG1268R	Biotin transporter bioY
Spy49_0184	-	NE	191368	192147	259	+	6984889	COG1234R	hypothetical protein Spy49_0184
Spy49_0185	-	NE	192147	192662	171	+	6984890	COG0590FJ	cytosine deaminase
Spy49_0186c	-	NE	193276	194496	406	-	6984891	COG5279D	hypothetical protein Spy49_0186c
Spy49_0187	speG	NE	194908	195612	234	+	6984892	-	Exotoxin type G
Spy49_0188	pgi	E	196067	197416	449	+	6984893	COG0166G	glucose-6-phosphate isomerase
Spy49_0189c	-	NE	197875	199272	465	-	6984894	-	RofA family transcriptional regulator
Spy49_0190	-	NE	199964	200635	223	+	6984895	COG0705R	hypothetical protein Spy49_0190
Spy49_0191c	hasC.2	E	200734	201633	299	-	6984896	COG1210M	UTP--glucose-1-phosphate uridylyltransferase
Spy49_0192c	gpsA	E	201666	202682	338	-	6984897	COG0240C	NAD(P)H-dependent glycerol-3-phosphate dehydrogenase
Spy49_0193	-	NE	203220	203426	68	+	6984898	-	MarR family transcriptional regulator, partial
Spy49_0194	-	NC	203419	205125	568	+	6984899	COG1132V	ABC transporter ATP-binding protein
Spy49_0195	-	NE	205128	206912	594	+	6984900	COG1132V	ABC transporter ATP-binding protein/permease
Spy49_0198	-	NE	207105	207797	230	+	6984901	COG1636S	hypothetical protein Spy49_0198
Spy49_0199	dut	NE	207907	208365	152	+	6984902	COG0756F	deoxyuridine 5\'-triphosphate nucleotidohydrolase
Spy49_0200	radA	NE	208402	209796	464	+	6984983	COG1066O	DNA repair protein RadA
Spy49_0201	-	NC	209985	210482	165	+	6984984	COG0288P	carbonic anhydrase
Spy49_0202	-	NE	210613	211323	236	+	6984985	COG2013S	hypothetical protein Spy49_0202
Spy49_0203	gltX	E	211506	212996	496	+	6984986	COG0008J	glutamyl-tRNA synthetase
Spy49_0204	fasB	NE	213347	214693	448	+	6984987	COG2972T	histidine kinase
Spy49_0205	-	NC	214690	215973	427	+	6984988	COG2972T	histidine kinase
Spy49_0206	fasA	NE	215977	216717	246	+	6984989	COG3279KT	Response regulator FasA
Spy49_0207	rnpA	NC	217257	217616	119	+	6984990	COG0594J	ribonuclease P
Spy49_0208	-	NE	217600	218409	269	+	6984991	COG0706U	hypothetical protein Spy49_0208
Spy49_0209	-	NC	218421	219335	304	+	6984992	COG1847R	R3H domain-containing protein
Spy49_0210	rpmH	NC	219650	219784	44	+	6984993	-	50S ribosomal protein L34
Spy49_0211	-	NE	220058	220762	234	+	6984994	COG3010G	N-acetylmannosamine-6-phosphate 2-epimerase
Spy49_0212	-	NE	220811	222130	439	+	6984995	COG1653G	sugar ABC transporter substrate-binding protein
Spy49_0213	-	NE	222235	223122	295	+	6984996	COG1175G	sugar ABC transporter
Spy49_0214	-	NE	223135	223965	276	+	6984997	COG0395G	sugar ABC transporter permease
Spy49_0215	-	NE	224179	224784	201	+	6984998	COG5578S	hypothetical protein Spy49_0215
Spy49_0216	nanH	NE	224796	225710	304	+	6984999	COG0329EM	acylneuraminate lyase
Spy49_0217	-	NE	225732	226670	312	+	6985000	COG1940KG	glucose kinase
Spy49_0218c	-	NE	226781	227611	276	-	6985001	COG1737K	phosphosugar-binding transcriptional regulator
Spy49_0219	-	NE	227863	228687	274	+	6985002	COG0084L	Sec-independent protein TatD
Spy49_0220	-	NE	228659	229249	196	+	6985003	COG1658L	ribonuclease M5
Spy49_0221	ksgA	NE	229363	230235	290	+	6985004	COG0030J	dimethyladenosine transferase
Spy49_0223	engC	NE	230662	231534	290	+	6985005	COG1162R	ribosome-associated GTPase
Spy49_0224	rpe	NC	231544	232206	220	+	6985006	COG0036G	ribulose-phosphate 3-epimerase
Spy49_0225	-	NE	232199	232831	210	+	6985007	COG1564H	thiamin pyrophosphokinase
Spy49_0226	-	NE	232833	234104	423	+	6985008	COG1322S	hypothetical protein Spy49_0226
Spy49_0227	cbf	NE	234094	235032	312	+	6985009	COG3481R	CMP-binding factor
Spy49_0228	purR	NE	235298	236137	279	+	6985010	COG0503F	pur operon repressor
Spy49_0229	prgA	NE	236128	238749	873	+	6985011	-	surface exclusion protein
Spy49_0230	rpsL	NC	238963	239367	134	+	6985012	-	30S ribosomal protein S12, partial
Spy49_0231	rpsG	E	239388	239858	156	+	6985013	COG0049J	30S ribosomal protein S7
Spy49_0233	fusA	E	240225	242303	692	+	6985014	COG0480J	elongation factor G

Spy49_0234	plr	E	242651	243661	336	+	6985015	COG0057G	NAD-dependent glyceraldehyde-3-phosphate dehydrogenase
Spy49_0235c	-	NC	243887	244003	38	-	6985016	-	hypothetical protein Spy49_0235c
Spy49_0236c	-	NE	244145	244885	246	-	6985017	COG1126E	amino acid ABC transporter ATP-binding protein
Spy49_0237c	-	NE	244951	246447	498	-	6985018	COG0765E	glutamine-binding periplasmic protein
Spy49_0238	-	NE	246645	248459	604	+	6985019	COG4907S	hypothetical protein Spy49_0238
Spy49_0239	bacA	NE	248637	249476	279	+	6985020	COG1968V	undecaprenyl pyrophosphate phosphatase
Spy49_0240	mecA	NE	249622	250383	253	+	6985021	COG4862OTN	adaptor protein
Spy49_0241	rgpG	E	250390	251559	389	+	6985022	COG0472M	undecaprenyl-phosphate N-acetylglucosaminyl 1- phosphate transferase
Spy49_0242	-	NC	251681	252451	256	+	6985023	COG0396O	iron-sulfur cluster assembly ATPase SufC
Spy49_0243	-	C	252546	253808	420	+	6985024	COG0719O	iron-sulfur cluster assembly protein SufD
Spy49_0244	-	NC	253839	255065	408	+	6985025	COG0520E	cysteine desulfurase
Spy49_0245	-	NC	255052	255531	159	+	6985026	COG0822C	iron-sulfur cluster assembly scaffold protein IscU
Spy49_0246	-	NC	255524	256942	472	+	6985027	COG0719O	iron-sulfur cluster assembly protein SufB
Spy49_0247	pbp7	NE	257094	258275	393	-	6985028	COG1686M	D-alanyl-D-alanine carboxypeptidase
Spy49_0248c	dacA	NE	258489	259676	395	-	6985029	COG1686M	Penicillin-binding protein 7
Spy49_0249	oppA	NE	260007	261977	656	+	6985030	COG4166E	peptide ABC transporter substrate-binding protein
Spy49_0250	oppB	NE	262042	263544	500	+	6985031	COG0601EP	peptide ABC transporter permease
Spy49_0251	oppC	NE	263544	264470	308	+	6985032	COG1173EP	peptide ABC transporter permease
Spy49_0252	oppD	NE	264479	265549	356	+	6985033	COG0444EP	peptide transport ATP-binding protein oppD
Spy49_0253	oppF	NE	265542	266465	307	+	6985034	COG1123R	peptide transport ATP-binding protein oppF
Spy49_0254c	-	NE	266613	266852	79	-	6985035	-	transposase
Spy49_0256	comX1	NE	272853	273338	161	+	6985042	-	ComX1 Alternate Sigma Factor
Spy49_0257	-	C	273839	274423	194	+	6985043	COG2179R	hypothetical protein Spy49_0257
Spy49_0258	-	E	274423	275544	373	+	6985044	COG1161R	GTP-binding protein YqeH
Spy49_0259	-	NE	275569	275877	102	+	6985045	COG1534J	hypothetical protein Spy49_0259
Spy49_0260	nadD	NC	275946	276578	210	+	6985046	COG1057H	nicotinic acid mononucleotide adenylyltransferase
Spy49_0261	-	NE	276575	277168	197	+	6985047	COG1713H	hypothetical protein Spy49_0261
Spy49_0262	-	NE	277168	277545	125	+	6985048	COG0799S	hypothetical protein Spy49_0262
Spy49_0263	-	NE	277593	278336	247	+	6985049	COG2226H	ubiquinone/menaquinone biosynthesis methyltransferase UbiE/COQ5
Spy49_0264	-	NE	278558	279664	368	+	6985050	COG1323R	hypothetical protein Spy49_0264
Spy49_0265	-	NE	280146	280862	238	+	6985051	COG0217S	hypothetical protein Spy49_0265
Spy49_0266	atmA	NC	281260	281907	215	+	6985052	COG0834ET	ABC transporter substrate-binding protein
Spy49_0268	atmB	NE	282234	283079	281	+	6985053	COG1464P	methionine ABC transporter substrate-binding protein
Spy49_0269	atmD	NE	283329	284393	354	+	6985054	COG1135P	ABC transporter
Spy49_0270	atmE	NE	284394	285086	230	+	6985055	COG2011P	methionine ABC transporter permease
Spy49_0271c	braB	NE	285140	286474	444	-	6985056	COG1114E	branched-chain amino acid ABC transporter
Spy49_0272	-	NE	286820	287959	379	+	6985057	COG3633E	serine/threonine transporter SstT
Spy49_0273c	-	E	288014	288688	224	-	6985058	COG0569P	potassium uptake protein, Trk family
Spy49_0274c	-	E	288698	290089	463	-	6985059	COG0168P	V-type Na+-ATPase subunit J
Spy49_0275	gidB	NE	290158	290871	237	-	6985060	COG0357M	16S rRNA methyltransferase GidB
Spy49_0276	lemA	NE	291021	291578	185	+	6985061	COG1704S	cytoplasmic membrane protein
Spy49_0277	htpX	NE	291625	292521	298	+	6985062	COG0501O	heat shock protein HtpX
Spy49_0278	-	NE	292755	293288	177	+	6985143	COG1399R	hypothetical protein Spy49_0278
Spy49_0279	csrR	NC	293555	294241	228	+	6985144	COG0745TK	Response regulator CsrR
Spy49_0280	csrS	E	294247	295749	500	+	6985145	COG0642T	transmembrane histidine kinase CsrS
Spy49_0281	nrdR	C	295964	296458	164	+	6985146	COG1327K	NrdR family transcriptional regulator
Spy49_0282	dnaB	E	296442	297617	391	+	6985147	COG3611L	chromosome replication initiation protein/membrane attachment protein
Spy49_0283	dnaI	E	297618	298520	300	+	6985148	COG1484L	primosomal protein DnaI
Spy49_0284	engA	E	298583	299893	436	+	6985149	COG1160R	GTP-binding protein EngA
Spy49_0285	snf	NC	300100	303198	1032	+	6985150	COG0553KL	SNF helicase
Spy49_0286	-	NE	303442	304044	200	+	6985151	-	hypothetical protein Spy49_0286
Spy49_0287	murC	E	304084	305412	442	+	6985152	COG0773M	UDP-N-acetylmuramate--L-alanine ligase
Spy49_0288	-	NE	305458	305940	160	+	6985153	COG0456R	arylalkylamine n-acetyltransferase
Spy49_0289	-	NC	306058	307626	522	+	6985154	COG1559R	aminodeoxychorismate lyase
Spy49_0290	greA	NC	307651	308181	176	+	6985155	COG0782K	transcription elongation factor GreA
Spy49_0291	oxaA	E	308800	309723	307	-	6985156	COG0706U	OxaA-like protein
Spy49_0292c	-	NE	309805	310083	92	-	6985157	COG1254C	acylphosphatase
Spy49_0293	-	NE	310344	311081	245	+	6985158	COG0566J	rRNA methylase
Spy49_0294	-	NE	311120	311620	166	+	6985159	COG1418R	hypothetical protein Spy49_0294
Spy49_0295	-	NC	311635	312324	229	+	6985160	COG0670R	hypothetical protein Spy49_0295
Spy49_0296	-	NC	312502	312744	80	+	6985161	COG3763S	hypothetical protein Spy49_0296
Spy49_0297	glr	E	312922	313716	264	+	6985162	COG0796M	glutamate racemase
Spy49_0298	-	NE	313713	314699	328	+	6985163	COG0127F	deoxyribonucleotide triphosphate pyrophosphatase/unknown domain fusion protein
Spy49_0299	-	NE	314678	315199	173	+	6985164	COG0622R	hypothetical protein Spy49_0299
Spy49_0300	-	NE	315196	315657	153	+	6985165	COG4109K	CBS domain-containing protein
Spy49_0301	xerD	NE	315654	316400	248	+	6985166	COG4974L	site-specific tyrosine recombinase XerD-like protein
Spy49_0302	scpA	NE	316400	317104	234	+	6985167	COG1354S	segregation and condensation protein A
Spy49_0303	scpB	NE	317101	317652	183	+	6985168	COG1386K	segregation and condensation protein B
Spy49_0304	rluB	NE	317773	318495	240	+	6985169	COG1187J	ribosomal large subunit pseudouridine synthase
Spy49_0305	-	NE	318495	318755	86	+	6985170	COG0759S	hypothetical protein Spy49_0305
Spy49_0306	-	NE	318933	319481	182	+	6985171	COG0219J	rRNA methylase
Spy49_0307	-	NE	319792	320328	178	+	6985172	COG3601S	riboflavin transporter YpaA
Spy49_0308	-	NE	320553	321011	152	+	6985173	COG0671I	phosphatase, partial
Spy49_0309	-	NE	321303	322223	306	+	6985174	COG1242R	hypothetical protein Spy49_0309
Spy49_0310	-	NE	322262	322816	184	+	6985175	COG2265J	hypothetical protein Spy49_0310
Spy49_0311	hlyX	NE	322950	324284	444	+	6985176	COG1253R	hemolysin
Spy49_0312	pflC	NE	324290	325153	287	+	6985177	COG1180O	pyruvate formate-lyase activating enzyme
Spy49_0313	-	E	325284	326219	311	+	6985178	COG1227C	manganese-dependent inorganic pyrophosphatase
Spy49_0314	-	NE	326295	326948	217	+	6985179	-	hypothetical protein Spy49_0314
Spy49_0315	fhuG	NE	326993	328030	345	-	6985180	COG0609P	ferrichrome ABC transporter permease
Spy49_0316	fhuB1	NE	327991	329043	350	-	6985181	COG0609P	ferrichrome ABC transporter permease
Spy49_0317c	fhuD	NE	329033	329965	310	-	6985182	COG0614P	ferrichrome ABC transporter substrate-binding protein
Spy49_0318	fhuC1	NE	329991	330773	260	-	6985183	COG1120PH	ferrichrome transport ATP-binding protein fhuC
Spy49_0319c	murE	E	331019	332464	481	-	6985184	COG0769M	UDP-N-acetylmuramoylalanyl-D-glutamate--L-lysine ligase
Spy49_0320	-	E	332552	334186	544	+	6985185	COG2244R	polysaccharide biosynthesis protein
Spy49_0322	upp	NE	334353	334982	209	+	6985186	COG0035F	uracil phosphoribosyltransferase
Spy49_0323	clpP	E	335206	335796	196	+	6985187	COG0740OU	ATP-dependent Clp protease proteolytic subunit
Spy49_0325	-	NE	336288	336563	91	+	6985188	COG4471S	hypothetical protein Spy49_0325
Spy49_0326	tmk	E	336812	337447	211	+	6985189	COG0125F	thymidylate kinase
Spy49_0327	-	E	337465	338340	291	+	6985190	COG0470L	DNA polymerase III subunit delta\'
Spy49_0328	-	NC	338688	338849	53	+	6985191	COG1774S	signal peptidase-like protein
Spy49_0329	-	C	339004	339327	107	+	6985192	COG4467S	DNA replication intiation control protein YabA
Spy49_0330	-	C	339332	340195	287	+	6985193	COG0313R	tetrapyrrole methylase
Spy49_0331	-	NE	340222	340614	130	+	6985194	-	hypothetical protein Spy49_0331
Spy49_0332c	-	NE	340661	341290	209	-	6985195	COG3142P	copper homeostasis protein
Spy49_0333	-	NE	341597	341953	118	+	6985196	COG1393P	arsenate reductase
Spy49_0334c	exoA	NE	342027	342938	303	-	6985197	COG0708L	3-exo-deoxyribonuclease
Spy49_0335	lctO	NE	343088	344269	393	+	6985198	COG1304C	lactate oxidase
Spy49_0336	prtS	NE	344611	349476	1621	+	6985199	COG1404O	cell envelope proteinase
Spy49_0337	-	NE	350303	350956	217	+	6985200	COG3619S	hypothetical protein Spy49_0337
Spy49_0338	metS	E	351196	353196	666	+	6985201	COG0143J	methionyl-tRNA synthetase
Spy49_0339	nrdF.2	NE	353694	354707	337	+	6985202	COG0208F	ribonucleotide-diphosphate reductase subunit beta
Spy49_0340	nrdI	NE	354711	355199	162	+	6985203	COG1780F	ribonucleotide reductase stimulatory protein
Spy49_0341	nrdE.2	NE	355166	357346	726	+	6985204	COG0209F	ribonucleotide-diphosphate reductase subunit alpha
Spy49_0342c	-	NE	357508	358260	250	-	6985205	COG5585T	hypothetical protein Spy49_0342c
Spy49_0343	-	NC	358946	359164	72	+	6985206	-	hypothetical protein Spy49_0343
Spy49_0344	-	NC	359112	359315	67	+	6985207	-	hypothetical protein Spy49_0344

Spy49_0345c	-	NE	359568	359765	65	-	6985208	COG3464L	transposase
Spy49_0346c	-	NE	360026	360859	277	-	6985209	COG3464L	transposase, ISSmu2
Spy49_0347	-	NE	361343	361612	89	+	6985210	-	hypothetical protein Spy49_0347
Spy49_0348	-	NE	362242	362646	134	+	6985211	-	hypothetical protein Spy49_0348
Spy49_0349c	-	NE	363232	363621	129	-	6985212	-	hypothetical protein Spy49_0349c
Spy49_0350c	-	NE	364340	365032	230	-	6985213	-	hypothetical protein Spy49_0350c
Spy49_0351c	-	NE	365190	365372	60	-	6985214	-	hypothetical protein Spy49_0351c
Spy49_0352c	-	NE	365511	365696	61	-	6985215	-	hypothetical protein Spy49_0352c
Spy49_0353c	-	NE	365740	366108	122	-	6985216	-	hypothetical protein Spy49_0353c
Spy49_0354c	-	NC	366083	366256	57	-	6985217	-	hypothetical protein Spy49_0354c
Spy49_0355c	-	NE	366619	367032	137	-	6985218	-	hypothetical protein Spy49_0355c
Spy49_0356c	-	NE	367138	367644	168	-	6985219	-	hypothetical protein Spy49_0356c
Spy49_0357c	-	NE	367681	368010	109	-	6985220	-	hypothetical protein Spy49_0357c
Spy49_0358c	-	NE	368000	368602	200	-	6985221	-	Prophage ps3 protein 07
Spy49_0359c	-	NE	368868	369134	88	-	6985222	-	hypothetical protein Spy49_0359c
Spy49_0360c	-	NE	369315	369488	57	-	6985303	-	phage protein
Spy49_0361c	-	NE	369776	371464	562	-	6985304	COG3378R	DNA primase-phage associated
Spy49_0362c	-	NE	371433	372290	285	-	6985305	-	replication protein
Spy49_0363c	-	NE	372434	372712	92	-	6985306	-	hypothetical protein Spy49_0363c
Spy49_0364c	-	NC	372715	372828	37	-	6985307	-	hypothetical protein Spy49_0364c, partial
Spy49_0365c	-	NE	373055	373249	64	-	6985308	-	hypothetical protein Spy49_0365c
Spy49_0366c	-	NE	373246	373455	69	-	6985309	-	hypothetical protein Spy49_0366c
Spy49_0367c	-	NE	373980	374228	82	-	6985310	-	hypothetical protein Spy49_0367c
Spy49_0368c	-	NE	374244	374429	61	-	6985311	COG1426S	hypothetical protein Spy49_0368c
Spy49_0369	-	E	374597	375265	222	+	6985312	COG1396K	phage-associated protein
Spy49_0370	-	NE	375347	376492	381	+	6985313	COG4974L	integrase
Spy49_0371	-	NE	377362	377598	78	+	6985314	-	hypothetical protein Spy49_0371
Spy49_0372	fabG	NE	377591	378289	232	+	6985315	COG1028IQR	3-ketoacyl-ACP reductase
Spy49_0373	-	NE	378365	379324	319	+	6985316	COG0673R	hypothetical protein Spy49_0373
Spy49_0374	glpT	NE	379657	380994	445	+	6985317	COG2271G	glycerol-3-phosphate transporter
Spy49_0375	glmU	E	381167	382549	460	+	6985318	COG1207M	bifunctional N-acetylglucosamine-1-phosphate uridyltransferase/glucosamine-1-phosphate acetyltransfe...
Spy49_0376	-	NE	382580	383134	184	+	6985319	COG0494LR	ADP-ribose pyrophosphatase
Spy49_0377	-	NE	383134	383385	83	+	6985320	-	hypothetical protein Spy49_0377
Spy49_0378	pfs	NC	383405	384100	231	+	6985321	COG0775F	5\'-methylthioadenosine/S-adenosylhomocysteine nucleosidase
Spy49_0379	-	NE	384251	384592	113	+	6985322	-	hypothetical protein Spy49_0379
Spy49_0380c	-	NC	384689	385336	215	-	6985323	COG1321K	metal-dependent transcriptional regulator
Spy49_0381	mtsA	NE	385590	386414	274	+	6985324	COG0803P	Metal ABC transporter substrate-binding lipoprotein
Spy49_0382	mtsB	NE	386478	387203	241	+	6985325	COG1121P	ATP-binding protein MtsB
Spy49_0383	mtsC	NE	387204	388058	284	+	6985326	COG1108P	Integral membrane protein MtsC, ABC transporter
Spy49_0384c	cypB	NE	388206	389012	268	-	6985327	COG0652O	cyclophilin-type protein
Spy49_0385	ftsK	E	389229	391634	801	+	6985328	COG1674D	cell division protein FtsK
Spy49_0386c	-	NE	391704	392054	116	-	6985329	-	hypothetical protein Spy49_0386c
Spy49_0387	rplK	NC	392301	392726	141	+	6985330	COG0080J	50S ribosomal protein L11
Spy49_0388	rplA	E	392832	393521	229	+	6985331	COG0081J	50S ribosomal protein L1
Spy49_0390	pyrH	E	393843	394571	242	+	6985332	COG0528F	uridylate kinase
Spy49_0391	frr	E	394600	395157	185	+	6985333	COG0233J	ribosome recycling factor
Spy49_0392	-	NE	395266	396123	285	+	6985334	COG2996S	S1 RNA binding domain
Spy49_0394	mrsA	NE	396196	396705	169	+	6985335	COG0225O	methionine sulfoxide reductase A
Spy49_0395	-	NE	396702	396917	71	+	6985336	COG4479S	hypothetical protein Spy49_0395
Spy49_0396	-	NE	397073	398242	389	+	6985337	-	42 kDa protein
Spy49_0398	-	NE	398517	400328	603	+	6985338	COG4716S	myosin-cross-reactive antigen
Spy49_0399	phoH	NE	400487	401539	350	+	6985339	COG1702T	phosphate starvation-induced protein
Spy49_0400	-	NE	401585	402160	191	+	6985340	COG1573L	hypothetical protein Spy49_0400
Spy49_0401	-	E	402319	402816	165	+	6985341	COG0319R	metalloprotease
Spy49_0402	dgk	E	402797	403204	135	+	6985342	COG0818M	Diacylglycerol kinase
Spy49_0403	era	E	403324	404220	298	+	6985343	COG1159R	GTP-binding protein Era
Spy49_0404	-	NE	404241	404717	158	+	6985344	COG1051F	hypothetical protein Spy49_0404
Spy49_0405c	-	NE	405023	405277	84	-	6985345	-	hypothetical protein Spy49_0405c
Spy49_0406	-	NE	405910	406092	60	+	6985346	-	hypothetical protein Spy49_0406
Spy49_0407c	-	NC	406430	406579	49	-	6985347	COG3328L	transposase
Spy49_0408c	-	NE	406576	406998	140	-	6985348	COG3328L	transposase for insertion sequence element IS905
Spy49_0409	-	NE	407316	407543	75	+	6985349	-	bacteriocin
Spy49_0410	-	NE	408295	408555	86	+	6985350	-	hypothetical protein Spy49_0410
Spy49_0411	-	NE	408771	409142	123	+	6985351	-	hypothetical protein Spy49_0411
Spy49_0412	-	NC	409427	410494	355	+	6985352	-	hypothetical protein Spy49_0412
Spy49_0413	-	NE	410587	410985	132	+	6985353	-	hypothetical protein Spy49_0413
Spy49_0414c	-	NE	411333	411602	89	-	6985354	-	hypothetical protein Spy49_0414c
Spy49_0415	mutR	NE	412673	413539	288	+	6985355	-	positive transcriptional regulator
Spy49_0416	fpg	NE	413711	414538	275	+	6985356	COG0266L	formamidopyrimidine-DNA glycosylase
Spy49_0417	coaE	E	414517	415128	203	+	6985357	COG0237H	dephospho-CoA kinase
Spy49_0418	-	NC	415318	416820	500	+	6985358	COG0433R	hypothetical protein Spy49_0418
Spy49_0419	-	NE	416942	418135	397	+	6985359	COG2814G	multidrug resistance efflux pump
Spy49_0420	rpmG	NC	418132	418278	48	+	6985360	COG0267J	50S ribosomal protein L33
Spy49_0421	secG	NC	418324	418560	78	+	6985361	-	preprotein translocase subunit SecG
Spy49_0422	vacB	NC	418654	420987	777	+	6985362	COG0557K	exoribonuclease R
Spy49_0423	smpB	NC	420990	421457	155	+	6985363	COG0691O	SsrA-binding protein
Spy49_0424	-	NE	421472	422182	236	+	6985364	COG3823O	glutamine cyclotransferase
Spy49_0425c	pcp	NE	422300	422947	215	-	6985365	COG2039O	pyrrolidone-carboxylate peptidase
Spy49_0426c	-	NE	422998	423924	308	-	6985366	COG3817S	hypothetical protein Spy49_0426c
Spy49_0427c	-	NE	423924	424607	227	-	6985367	COG3819S	hypothetical protein Spy49_0427c
Spy49_0428c	-	NE	424818	425744	308	-	6985368	COG0463M	sugar transferase
Spy49_0429c	gloA	NE	425889	426266	125	-	6985369	COG0346E	lactoylglutathione lyase
Spy49_0430c	-	NE	426277	426942	221	-	6985370	COG0778C	NAD(P)H-flavin oxidoreductase
Spy49_0431c	pepQ	NE	426991	428076	361	-	6985371	COG0006E	Xaa-Pro dipeptidase
Spy49_0432	ccpA	E	428250	429251	333	+	6985372	COG1609K	Catabolite control protein A
Spy49_0433	-	E	429382	430380	332	+	6985373	COG0438M	sugar transferase
Spy49_0434	-	E	430382	431716	444	+	6985374	COG0438M	glucosyl transferase
Spy49_0435	thrS	E	432138	434081	647	+	6985375	COG0441J	threonyl-tRNA synthetase
Spy49_0436	tagH	NE	434222	435214	330	+	6985376	COG4586R	Teichoic acid export ATP-binding protein tagH
Spy49_0437	-	NE	435216	436034	272	+	6985377	COG4587R	hypothetical protein Spy49_0437
Spy49_0438	-	NE	436036	436821	261	+	6985378	COG3694R	hypothetical protein Spy49_0438
Spy49_0439	-	NC	437022	437171	49	+	6985379	-	dihydroxyacetone kinase
Spy49_0440	-	NE	437566	438714	382	+	6985380	COG0183I	acetyl-CoA acetyltransferase
Spy49_0441	-	NE	438671	439918	415	+	6985381	COG0318IQ	Long-chain-fatty-acid--CoA ligase
Spy49_0442	-	NE	439974	441008	344	+	6985382	-	hypothetical protein Spy49_0442
Spy49_0444	vicR	E	441170	441880	236	+	6985463	COG0745TK	two-component response regulator
Spy49_0445	vicK	NE	441873	443225	450	+	6985464	COG5002T	two-component sensor histidine kinase
Spy49_0446	vicX	NE	443229	444038	269	+	6985465	COG1235R	Zn-dependent hydrolase
Spy49_0447	rnc	NC	444483	445175	230	+	6985466	COG0571K	ribonuclease III
Spy49_0448	smc	NC	445176	448715	1179	+	6985467	COG1196D	chromosome segregation SMC
Spy49_0449c	-	NE	448963	449814	283	-	6985468	-	positive regulator
Spy49_0450	aroE	NE	450088	450960	290	+	6985469	COG0169E	shikimate 5-dehydrogenase
Spy49_0451	-	NE	450957	451793	278	+	6985470	COG1082G	hypothetical protein Spy49_0451
Spy49_0453	-	NC	451795	452529	244	+	6985471	COG1478S	hypothetical protein Spy49_0453
Spy49_0454	-	NE	452522	453508	328	+	6985472	COG0673R	hypothetical protein Spy49_0454
Spy49_0455	-	NE	453518	454717	399	+	6985473	COG1812E	S-adenosylmethionine synthetase
Spy49_0456	-	NE	454701	455603	300	+	6985474	COG1493T	hypothetical protein Spy49_0456

Spy49_0457	-	NE	455724	456713	329	+	6985475	COG0463M	hypothetical protein Spy49_0457
Spy49_0458	-	NE	457116	457382	88	+	6985476	-	hypothetical protein Spy49_0458
Spy49_0459	-	NE	457406	458563	385	+	6985477	COG1004M	UDP-glucose dehydrogenase
Spy49_0460	-	NE	458648	459817	389	+	6985478	-	efflux protein
Spy49_0461c	-	NE	459958	460167	69	-	6985479	COG3655K	hypothetical protein Spy49_0461c
Spy49_0462c	-	NE	460157	461815	552	-	6985480	-	hypothetical protein Spy49_0462c
Spy49_0463c	-	NE	461806	462030	74	-	6985481	-	hypothetical protein Spy49_0463c
Spy49_0464c	-	NE	462034	462924	296	-	6985482	-	hypothetical protein Spy49_0464c
Spy49_0465	-	NC	462958	463116	52	+	6985483	-	hypothetical protein Spy49_0465
Spy49_0466	-	NE	463171	463473	100	+	6985484	COG3077L	hypothetical protein Spy49_0466
Spy49_0467	-	NE	463602	463778	58	+	6985485	-	hypothetical protein Spy49_0467, partial
Spy49_0468	-	NE	464174	464626	150	+	6985486	COG4695S	Portal protein
Spy49_0469	-	NE	464692	465000	102	+	6985487	-	hypothetical protein Spy49_0469
Spy49_0470c	-	NE	465238	465426	62	-	6985488	-	hypothetical protein Spy49_0470c
Spy49_0471	-	NE	465417	465740	107	+	6985489	COG3177S	hypothetical protein Spy49_0471
Spy49_0473	-	NE	465785	466456	223	+	6985490	COG0017J	asparagine synthetase A
Spy49_0474	-	NE	466617	467864	415	+	6985491	COG0439I	hypothetical protein Spy49_0474
Spy49_0475	-	NE	468092	468808	238	+	6985492	COG1619V	Microcin C7 self-immunity protein mccF
Spy49_0476	-	NE	468984	469943	319	+	6985493	COG0124J	hypothetical protein Spy49_0476
Spy49_0477c	-	NE	470313	471116	267	-	6985494	COG2801L	transposase
Spy49_0478c	-	NE	471164	471439	91	-	6985495	COG2963L	transposase OrfA, IS3 family
Spy49_0479	-	NE	471459	471677	72	+	6985496	-	hypothetical protein Spy49_0479
Spy49_0480	-	NE	471744	472541	265	+	6985497	COG0561R	hypothetical protein Spy49_0480
Spy49_0481	-	NE	472545	473369	274	+	6985498	COG0561R	hypothetical protein Spy49_0481
Spy49_0482	ftsY	E	473369	474919	516	+	6985499	COG0552U	signal recognition particle receptor protein FtsY subunit alpha
Spy49_0483c	-	NE	474973	476340	455	-	6985500	COG2814G	drug resistance protein
Spy49_0484	bglG	NE	476668	477510	280	+	6985501	COG3711K	transcription antiterminator
Spy49_0485	-	NE	477512	479374	620	+	6985502	COG1263G	beta-glucoside permease IIABC component
Spy49_0487	bglA	NE	479393	480817	474	+	6985503	COG2723G	beta-glucosidase
Spy49_0488c	-	NE	480916	481731	271	-	6985504	COG3689S	hypothetical protein Spy49_0488c
Spy49_0489c	-	NE	481731	482633	300	-	6985505	COG0701R	hypothetical protein Spy49_0489c
Spy49_0490	-	NE	483170	485302	710	+	6985506	COG2183K	hypothetical protein Spy49_0490
Spy49_0491	-	NE	485244	485726	160	+	6985507	COG3091S	hypothetical protein Spy49_0491
Spy49_0493	-	NE	485790	486062	90	+	6985508	COG1983KT	hypothetical protein Spy49_0493
Spy49_0494	ptsK	E	486367	487359	330	+	6985509	COG1493T	HPr kinase/phosphorylase
Spy49_0495	lgt	NE	487356	488135	259	+	6985510	COG0682M	prolipoprotein diacylglyceryl transferase
Spy49_0496	-	NE	488157	488564	135	+	6985511	COG4768R	hypothetical protein Spy49_0496
Spy49_0497	-	NE	488557	488985	142	+	6985512	-	hypothetical protein Spy49_0497
Spy49_0498	-	NE	489044	489313	89	+	6985513	-	hypothetical protein Spy49_0498
Spy49_0499	-	NE	489631	490557	308	+	6985514	COG0826O	protease
Spy49_0500	-	NE	490659	491945	428	+	6985515	COG0826O	protease
Spy49_0501	-	NE	492146	492373	75	+	6985516	COG4443S	hypothetical protein Spy49_0501
Spy49_0502c	-	NC	492470	492610	46	-	6985517	-	hypothetical protein Spy49_0502c
Spy49_0503c	lysS	E	492746	494239	497	-	6985518	COG1190J	lysyl-tRNA synthetase
Spy49_0504	-	NE	494413	495315	300	+	6985519	COG1011R	hypothetical protein Spy49_0504
Spy49_0505c	-	NE	495423	496046	207	-	6985520	COG0406G	phosphoglycerate mutase
Spy49_0506c	-	NE	496387	496866	159	-	6985521	COG2606S	transcription regulator
Spy49_0507c	-	NE	496957	497520	187	-	6985522	COG3859S	thiamin transporter
Spy49_0508c	-	NE	497789	498637	282	-	6985523	COG3757M	endolysin, phage associated
Spy49_0509	-	NE	498962	499465	167	+	6985524	-	ABC transporter permease
Spy49_0510	-	NE	499449	499835	128	+	6985525	-	hypothetical protein Spy49_0510
Spy49_0511c	-	NE	499881	500360	159	-	6985526	COG0386O	glutathione peroxidase
Spy49_0512c	pepF	NE	500353	502152	599	-	6985527	COG1164E	oligoendopeptidase F
Spy49_0513	ppc	NE	502311	505109	932	+	6985528	COG2352C	phosphoenolpyruvate carboxylase
Spy49_0514	ftsW	E	505279	506187	302	+	6985529	COG0772D	cell division protein FtsW
Spy49_0515	tufA	NC	506912	508108	398	+	6985530	COG0050J	elongation factor Tu
Spy49_0516	tpiA	E	508349	509107	252	+	6985531	COG0149G	triosephosphate isomerase
Spy49_0517c	murN	E	509207	510442	411	-	6985532	COG2348V	peptidoglycan branched peptide synthesis protein, alanine adding enzyme
Spy49_0518c	murM.1	E	510429	511655	408	-	6985533	COG2348V	peptidoglycan branched peptide synthesis protein, serine/alanine adding enzyme
Spy49_0519c	-	NE	511655	512464	269	-	6985534	COG0561R	hypothetical protein Spy49_0519c
Spy49_0520c	-	NE	512920	514221	433	-	6985535	COG1078R	HD domain-containing protein
Spy49_0521	-	NE	514303	514689	128	+	6985536	COG4506S	hypothetical protein Spy49_0521
Spy49_0522	pacL	E	514920	517601	893	+	6985537	COG0474P	calcium-transporting ATPase
Spy49_0523c	regR	NE	517687	518682	331	-	6985538	COG1609K	transcriptional regulator
Spy49_0524c	-	NE	518746	520653	635	-	6985539	-	hypothetical protein Spy49_0524c
Spy49_0525c	agaD	NC	520740	521561	273	-	6985540	COG3716G	PTS dependent galactosamine IID component
Spy49_0526c	agaW	NE	521548	522330	260	-	6985541	COG3715G	PTS dependent N-acetyl-galactosamine- IIC component
Spy49_0527c	agaV	NE	522349	522837	162	-	6985542	COG3444G	PTS system IIB component
Spy49_0528c	ugl	NE	522873	524072	399	-	6985543	COG4225R	glucuronyl hydrolase
Spy49_0529c	agaF	NE	524072	524509	145	-	6985544	COG2893G	PTS dependent N-acetyl-galactosamine- and galactosamine IIA component
Spy49_0530	-	NE	524844	525638	264	+	6985545	COG1028IQR	gluconate 5-dehydrogenase
Spy49_0531	-	NE	525663	526304	213	+	6985546	COG0698G	hypothetical protein Spy49_0531
Spy49_0532	kdgK	NE	526333	527334	333	+	6985547	COG0524G	2-dehydro-3-deoxygluconokinase
Spy49_0533	kdgA	NE	527339	527974	211	+	6985548	COG0800G	keto-hydroxyglutarate-aldolase/keto-deoxy-phosphogluconate aldolase
Spy49_0534	-	NE	528272	528922	216	+	6985549	COG0637R	hypothetical protein Spy49_0534
Spy49_0535	-	NE	529618	530742	374	+	6985550	COG1600C	hypothetical protein Spy49_0535
Spy49_0536	prfB	E	530928	531908	326	+	6985551	COG1186J	peptide chain release factor 2
Spy49_0537	ftsE	NC	531927	532619	230	+	6985552	COG2884D	cell division transporter ATP-binding protein ftsE
Spy49_0538	ftsX	E	532612	533541	309	+	6985553	COG2177D	cell division protein FtsX
Spy49_0539c	-	NE	533851	534429	192	-	6985554	COG0491R	oxyacylglutathione hydrolase
Spy49_0540	-	NE	534718	535482	254	+	6985555	COG1028IQR	acetoin reductase
Spy49_0541	dinG	NE	535590	538091	833	+	6985556	COG1199KL	bifunctional ATP-dependent DNA helicase/DNA polymerase III subunit epsilon
Spy49_0543	aspC	NE	538426	539619	397	+	6985557	COG0436E	aspartate aminotransferase
Spy49_0544	asnC	E	539640	540986	448	+	6985558	COG0017J	asparaginyl-tRNA synthetase
Spy49_0545	-	E	541401	542291	296	+	6985559	COG1660R	hypothetical protein Spy49_0545
Spy49_0546	-	NE	542288	543265	325	+	6985560	COG0391S	hypothetical protein Spy49_0546
Spy49_0547	-	NE	543262	544173	303	+	6985561	COG1481S	hypothetical protein Spy49_0547
Spy49_0548	pepD	NE	544306	545703	465	+	6985562	COG4690E	dipeptidase A
Spy49_0549	adcA	NE	545855	547402	515	+	6985563	COG3443R	zinc-binding protein adcA
Spy49_0550	-	NE	547552	548274	240	+	6985564	COG2188K	GntR family transcriptional regulator
Spy49_0551	agaS	NE	548294	549493	399	+	6985565	COG2222M	tagatose-6-phosphate aldose/ketose isomerase
Spy49_0552c	rpmE2	NC	549590	549850	86	-	6985566	COG0254J	50S ribosomal protein L31 type B
Spy49_0554c	-	NE	549965	550906	313	-	6985567	COG0618R	hypothetical protein Spy49_0554c
Spy49_0555	flaV	NE	551300	551749	149	+	6985568	COG0716C	flavodoxin
Spy49_0556	-	NC	551925	552209	94	+	6985569	COG1605E	hypothetical protein Spy49_0556
Spy49_0557	-	C	552202	553464	420	+	6985570	COG0038P	transmembrane protein
Spy49_0558	rplS	NC	553579	553926	115	+	6985571	COG0335J	50S ribosomal protein L19
Spy49_0559	-	NE	554947	555516	189	+	6985573	COG0546R	phosphoglycolate phosphatase
Spy49_0560	gyrB	E	555517	557469	650	+	6985574	COG0187L	DNA gyrase subunit B
Spy49_0561	ezrA	E	557837	559561	574	+	6985575	COG4477D	septation ring formation regulator EzrA
Spy49_0562c	-	NE	559693	560151	152	-	6985576	COG5506S	hypothetical protein Spy49_0562c
Spy49_0563	eno	E	560384	561691	435	+	6985577	COG0148G	phosphopyruvate hydratase
Spy49_0564c	-	NE	562280	562855	191	-	6985578	COG3547L	transposase
Spy49_0565c	-	NC	562941	563102	53	-	6985579	-	transposase
Spy49_0566c	-	NE	563464	565005	513	-	6985580	-	RofA family transcriptional regulator
Spy49_0567	-	NC	565552	568944	1130	+	6985581	-	extracellular matrix binding protein
Spy49_0568	sagA	NE	569809	569970	53	+	6985582	-	streptolysin S associated protein SagA

Spy49_0569	sagB	NE	570192	571142	316	+	6985583	-	streptolysin S biosynthesis protein B SagB
Spy49_0570	sagC	NE	571139	572197	352	+	6985584	-	streptolysin S biosynthesis protein C SagC
Spy49_0572	sagD	NE	572217	573575	452	+	6985585	COG1944S	streptolysin S biosynthesis protein D SagD
Spy49_0573	sagE	NE	573550	574221	223	+	6985586	-	streptolysin S self-immunity protein SagE
Spy49_0574	sagF	NE	574218	574901	227	+	6985587	-	streptolysin S biosynthesis protein SagF
Spy49_0575	sagG	C	574924	575847	307	+	6985588	COG1131V	streptolysin S export ATP-binding protein SagG
Spy49_0576	sagH	NC	575856	576983	375	+	6985589	COG0842V	streptolysin S export transmembrane protein SagH
Spy49_0577	sagI	C	576980	578098	372	+	6985590	COG0842V	streptolysin S export transmembrane protein SagI
Spy49_0578	-	NE	578669	581401	910	+	6985591	COG2374R	endonuclease/exonuclease/phosphatase
Spy49_0579	-	NE	581679	582179	166	+	6985592	COG4708S	hypothetical protein Spy49_0579
Spy49_0580	ligA	E	582373	584331	652	+	6985593	COG0272L	NAD-dependent DNA ligase LigA
Spy49_0581	-	E	584345	585367	340	+	6985594	COG1597IR	lipid kinase
Spy49_0582	-	NC	585762	585959	65	+	6985595	COG0636C	F0F1 ATP synthase subunit C
Spy49_0583	-	E	585994	586710	238	+	6985596	COG0356C	F0F1 ATP synthase subunit A
Spy49_0584	-	NC	586728	587222	164	+	6985597	COG0711C	F0F1 ATP synthase subunit B
Spy49_0585	-	E	587222	587758	178	+	6985598	COG0712C	F0F1 ATP synthase subunit delta
Spy49_0586	-	E	587774	589282	502	+	6985599	COG0056C	F0F1 ATP synthase subunit alpha
Spy49_0587	-	E	589298	590173	291	+	6985600	COG0224C	F0F1 ATP synthase subunit gamma
Spy49_0588	-	E	590335	591741	468	+	6985601	COG0055C	F0F1 ATP synthase subunit beta
Spy49_0589	atpC	NC	591754	592170	138	+	6985602	COG0355C	F0F1 ATP synthase subunit epsilon
Spy49_0590	-	NE	592436	592693	85	+	6985603	-	hypothetical protein Spy49_0590
Spy49_0591	murA	NE	592758	594029	423	+	6985604	COG0766M	UDP-N-acetylglucosamine 1-carboxyvinyltransferase
Spy49_0592	-	NE	594033	594221	62	+	6985605	-	hypothetical protein Spy49_0592
Spy49_0596	pheS	E	595077	596120	347	+	6985608	COG0016J	phenylalanyl-tRNA synthetase subunit alpha
Spy49_0597	pheT	E	596330	598735	801	+	6985609	COG0072J	phenylalanyl-tRNA synthetase subunit beta
Spy49_0598	-	NE	598845	599222	125	+	6985610	-	hypothetical protein Spy49_0598
Spy49_0599	-	NE	599215	599601	128	+	6985611	COG3272S	hypothetical protein Spy49_0599
Spy49_0600	-	NE	599674	600750	358	+	6985612	-	hypothetical protein Spy49_0600
Spy49_0601	-	NE	600760	601428	222	+	6985613	COG1136V	ABC transporter ATP-binding protein
Spy49_0602c	-	NE	601532	602449	305	-	6985614	COG2321R	hypothetical protein Spy49_0602c
Spy49_0603	rexB	C	602600	605815	1071	+	6985615	COG3857L	ATP-dependent exonuclease subunit B
Spy49_0604	rexA	E	605776	609444	1222	+	6985616	COG1074L	ATP-dependent exonuclease subunit A
Spy49_0605	-	NE	609584	610204	206	+	6985617	COG0834ET	ABC transporter
Spy49_0606	rpsU	NC	610543	610719	58	+	6985618	COG0828J	30S ribosomal protein S21p
Spy49_0607c	mscL	NE	610847	611209	120	-	6985619	COG1970M	large conductance mechanosensitive channel
Spy49_0608	dnaG	E	611339	613153	604	+	6985620	COG0358L	DNA primase
Spy49_0609	rpoD	E	613162	614271	369	+	6985621	COG0568K	RNA polymerase sigma factor RpoD
Spy49_0610	-	NE	614507	614845	112	+	6985622	COG2151R	probably aromatic ring hydroxylating enzyme
Spy49_0611	rmlD	E	614983	615837	284	+	6985623	COG1091M	dTDP-4-dehydrorhamnose reductase
Spy49_0612	rgpAc	E	615956	617110	384	+	6985624	COG0438M	rhamnosyltransferase
Spy49_0613	rgpBc	E	617100	618032	310	+	6985625	COG0463M	Rhamnosyltransferase
Spy49_0614	rgpCc	E	618035	618838	267	+	6985626	COG1682GM	Teichoic acid translocation permease tagG
Spy49_0615	rgpDc	E	618838	620058	406	+	6985627	COG1134GM	Teichoic acid export ATP-binding protein tagH
Spy49_0617	rgpEc	E	620069	621076	335	+	6985628	COG0463M	glycosyltransferase
Spy49_0618	rgpFc	E	621073	622818	581	+	6985629	COG3754M	RgpFc protein
Spy49_0619	-	NE	622815	625289	824	+	6985630	COG1368M	hypothetical protein Spy49_0619
Spy49_0620	-	NE	625468	626163	231	+	6985631	COG0463M	glycosyl transferase
Spy49_0621	-	NE	626165	626506	113	+	6985632	-	hypothetical protein Spy49_0621
Spy49_0622	-	E	626499	627785	428	+	6985633	COG2244R	Heteropolysaccharide repeat unit export protein
Spy49_0623	-	E	627766	629262	498	+	6985634	COG4713S	hypothetical protein Spy49_0623
Spy49_0624	pepT	NE	629356	630579	407	+	6985635	COG2195E	peptidase T
Spy49_0625	-	NE	630620	631108	162	+	6985636	-	pore-forming peptide
Spy49_0626c	-	NE	631095	631292	65	-	6985637	COG1141C	ferredoxin
Spy49_0627	-	NE	631341	631817	158	+	6985638	-	hypothetical protein Spy49_0627
Spy49_0628	cmk	NC	631832	632512	226	+	6985639	COG0283F	cytidylate kinase
Spy49_0629	infC	E	632674	633204	176	+	6985640	COG0290J	translation initiation factor IF-3
Spy49_0630	rpmI	NE	633246	633443	65	+	6985641	COG0291J	50S ribosomal protein L35
Spy49_0631	rplT	NC	633502	633861	119	+	6985642	COG0292J	50S ribosomal protein L20
Spy49_0632c	-	E	634152	636362	736	-	6985643	COG1368M	phosphoglycerol transferase
Spy49_0633	-	NE	636470	637633	387	+	6985644	COG1092R	hypothetical protein Spy49_0633
Spy49_0634	aroD	NE	637630	638316	228	+	6985645	COG0710E	3-dehydroquinate dehydratase
Spy49_0635	aroF	NE	638410	639576	388	+	6985646	COG0082E	chorismate synthase
Spy49_0636	-	NE	639637	639978	113	+	6985647	COG3679S	hypothetical protein Spy49_0636
Spy49_0637	gor	NE	640200	641552	450	+	6985648	COG1249C	glutathione reductase
Spy49_0638c	folC.2	NE	641640	642908	422	-	6985649	COG0285H	dihydrofolate synthase / Folylpolyglutamate synthase
Spy49_0639c	-	NE	642938	643378	146	-	6985650	-	hypothetical protein Spy49_0639c
Spy49_0640	nifS	NE	643613	644755	380	+	6985651	COG1104E	cysteine desulfurase
Spy49_0641	thiI	NE	644767	645981	404	+	6985652	COG0301H	thiamine biosynthesis protein ThiI
Spy49_0642	-	NE	646019	647287	422	+	6985653	COG2843M	hypothetical protein Spy49_0642
Spy49_0643	rplU	NC	647526	647840	104	+	6985654	COG0261J	50S ribosomal protein L21
Spy49_0644	-	NC	647852	648178	108	+	6985655	COG2868J	hypothetical protein Spy49_0644
Spy49_0645	rpmA	NC	648206	648499	97	+	6985656	COG0211J	50S ribosomal protein L27
Spy49_0646	-	NE	648847	649761	304	+	6985657	COG0583K	capsular polysaccharide biosynthesis regulatory protein
Spy49_0647	lsp	NE	649758	650216	152	+	6985658	COG0597MU	lipoprotein signal peptidase
Spy49_0648	-	NE	650206	651096	296	+	6985659	COG0564J	ribosomal large subunit pseudouridine synthase
Spy49_0650	pyrR	NE	651492	652013	173	+	6985660	COG2065F	bifunctional pyrimidine regulatory protein PyrR uracil phosphoribosyltransferase
Spy49_0651	pyrP	NE	652029	653288	419	+	6985661	COG2233F	uracil permease
Spy49_0652	pyrB	NE	653349	654284	311	+	6985662	COG0540F	aspartate carbamoyltransferase catalytic subunit
Spy49_0653	carA	NC	654328	655410	360	+	6985663	COG0505EF	carbamoyl phosphate synthase small subunit
Spy49_0654	carB	NE	655656	658832	1058	+	6985664	COG0458EF	carbamoyl phosphate synthase large subunit
Spy49_0655	-	NE	659041	660312	423	+	6985665	COG0845M	hypothetical protein Spy49_0655
Spy49_0656	-	NE	660312	661022	236	+	6985666	COG1136V	ABC transporter
Spy49_0657	-	NE	661034	662254	406	+	6985667	COG0577V	Permease
Spy49_0658	-	C	662508	664241	577	+	6985668	COG4781C	glycerophosphoryl diester phosphodiesterase
Spy49_0659	rpsP	E	664368	664640	90	+	6985669	COG0228J	30S ribosomal protein S16
Spy49_0660	-	NC	664650	664889	79	+	6985670	COG1837R	KH domain-containing protein
Spy49_0661	-	NE	665821	668847	1008	+	6985671	-	hypothetical protein Spy49_0661
Spy49_0662	-	NE	668867	669268	133	+	6985672	COG3576R	hypothetical protein Spy49_0662
Spy49_0663c	czcD	NE	669448	670323	291	-	6985673	COG1230P	cation-efflux system membrane protein
Spy49_0664	-	NE	670459	670980	173	+	6985674	-	hypothetical protein Spy49_0664
Spy49_0665	rimM	NC	671195	671713	172	+	6985675	COG0806J	16S rRNA-processing protein RimM
Spy49_0667	trmD	E	671700	672434	244	+	6985676	COG0336J	tRNA (guanine-N(1)-)-methyltransferase
Spy49_0668	-	NE	672434	673426	330	+	6985677	COG0492O	thioredoxin reductase
Spy49_0669	-	NE	673603	674661	352	+	6985678	COG3641R	regulatory protein
Spy49_0671	apbA	NE	674674	675597	307	+	6985679	COG1893H	2-dehydropantoate 2-reductase
Spy49_0672	fruR	NE	675853	676566	237	+	6985680	COG1349KG	transcriptional repressor
Spy49_0673	fruK	NE	676563	677474	303	+	6985681	COG1105G	tagatose-6-phosphate kinase
Spy49_0674	fruA	NE	677471	679417	648	+	6985682	COG1299G	PTS system fructose-specific IIABC component
Spy49_0675	-	NE	679516	680109	197	+	6985683	COG1705NU	peptidoglycan hydrolase
Spy49_0676	-	NE	680261	680968	235	+	6985684	COG1705NU	peptidoglycan hydrolase
Spy49_0677c	-	NE	681025	681243	72	-	6985685	-	transposase, ISSmu1
Spy49_0679	mac	NE	681925	682944	339	-	6985687	-	Immunoglobulin G-endopeptidase (IdeS) / Mac
Spy49_0680	-	NE	683850	684230	126	+	6985688	COG4835S	hypothetical protein Spy49_0680
Spy49_0681	-	NE	684230	685078	282	+	6985689	COG1307S	DegV family protein
Spy49_0682	papS	E	685252	686412	386	+	6985690	COG0617J	tRNA CCA-pyrophosphorylase
Spy49_0683	-	NE	686409	688286	625	+	6985691	COG0488R	ABC transporter
Spy49_0684	-	NE	689180	689590	136	+	6985692	COG0242J	peptide deformylase

Spy49_0686c	-	NE	689672	691684	670	-	6985693	COG0737F	5-nucleotidase
Spy49_0687	-	NE	691904	692554	216	+	6985694	COG2357S	GTP pyrophosphokinase
Spy49_0688	-	NE	692557	693225	222	+	6985695	COG0745TK	DNA-binding response regulator
Spy49_0689	-	NE	693234	694466	410	+	6985696	COG0642T	two-component sensor histidine kinase
Spy49_0690	mvaK1	E	694760	695638	292	+	6985697	COG1577I	mevalonate kinase
Spy49_0691	mvaD	E	695620	696564	314	+	6985698	COG3407I	Diphosphomevalonate decarboxylase
Spy49_0692	mvaK2	E	696557	697570	337	+	6985699	COG1577I	phosphomevalonate kinase
Spy49_0693	-	E	697557	698546	329	+	6985700	COG1304C	isopentenyl pyrophosphate isomerase
Spy49_0694c	mvaS1	E	698776	700053	425	-	6985701	COG1257I	hydroxymethylglutaryl-CoA reductase
Spy49_0695c	mvaS2	E	700040	701215	391	-	6985702	COG3425I	hydroxymethylglutaryl-CoA synthase
Spy49_0696	thyA	C	701424	702263	279	+	6985703	COG0207F	thymidylate synthase
Spy49_0697	dyr	E	702343	702840	165	+	6985704	COG0262H	dihydrofolate reductase
Spy49_0698	-	NE	702860	703060	66	+	6985705	-	hypothetical protein Spy49_0698
Spy49_0700	clpX	NC	703161	704390	409	+	6985706	COG1219O	ATP-dependent protease ATP-binding protein ClpX
Spy49_0701	engB	E	704400	704999	199	+	6985707	COG0218R	ribosome biogenesis GTP-binding protein YsxC
Spy49_0702	-	NE	705147	705890	247	+	6985708	-	hypothetical protein Spy49_0702
Spy49_0703c	clpL	NE	705948	708047	699	-	6985709	COG0542O	ATP-dependent Clp proteinase
Spy49_0704	rpiA	NC	708425	709108	227	+	6985710	COG0120G	ribose-5-phosphate isomerase A
Spy49_0705	deoB	NE	709185	710396	403	+	6985711	COG1015G	phosphopentomutase
Spy49_0706	arsC	NE	710415	710855	146	+	6985712	COG1393P	arsenate reductase
Spy49_0707	punA	NE	710839	711648	269	+	6985713	COG0005F	purine nucleoside phosphorylase
Spy49_0708	deoD	NE	712176	712946	256	+	6985714	COG0813F	purine nucleoside phosphorylase
Spy49_0709	-	NE	712939	713727	262	+	6985715	-	histidine protein kinase
Spy49_0710c	cpsY	NE	713807	714712	301	-	6985716	COG0583K	transcriptional regulator
Spy49_0711	-	NE	714934	715470	178	+	6985717	-	hypothetical protein Spy49_0711
Spy49_0712	pyrF	NE	715747	716439	230	+	6985718	COG0284F	orotidine 5\'-phosphate decarboxylase
Spy49_0713	pyrE	NE	716497	717126	209	+	6985719	COG0461F	orotate phosphoribosyltransferase
Spy49_0714	amiC	NE	717323	718777	484	+	6985720	COG0154J	amidase
Spy49_0715	-	NE	718895	719758	287	+	6985721	COG0834ET	ABC transporter
Spy49_0716	-	NE	719787	720437	216	+	6985722	COG0765E	amino-acid ABC transporter permease yckA
Spy49_0717	ung	NE	720570	721223	217	+	6985723	COG0692L	uracil-DNA glycosylase
Spy49_0718	pyrC	NE	721355	722623	422	+	6985724	COG0044F	dihydroorotase
Spy49_0719c	-	E	722681	723322	213	-	6985725	COG0344S	glycerol-3-phosphate acyltransferase PlsY
Spy49_0720	parE	E	723457	725406	649	+	6985726	COG0187L	DNA topoisomerase IV subunit B
Spy49_0721	parC	E	725497	727956	819	+	6985727	COG0188L	DNA topoisomerase IV subunit A
Spy49_0722	bcaT	NE	728079	729101	340	+	6985728	COG0115EH	branched-chain amino acid aminotransferase
Spy49_0723	-	NC	729165	729395	76	+	6985729	-	hypothetical protein Spy49_0723
Spy49_0724	rpsA	E	729786	730991	401	+	6985732	COG0539J	30S ribosomal protein S1
Spy49_0725c	-	NE	731493	731795	100	-	6985734	-	hypothetical protein Spy49_0725c
Spy49_0726	-	NE	731996	732982	328	+	6985735	COG1054R	hypothetical protein Spy49_0726
Spy49_0727	-	NE	733293	733700	135	+	6985736	-	hypothetical protein Spy49_0727
Spy49_0728	-	NE	733950	734858	302	+	6985737	COG1275P	exfoliative toxin
Spy49_0729c	-	NE	734976	735152	58	-	6985738	-	hypothetical protein Spy49_0729c
Spy49_0730	miaA	NE	735286	736185	299	+	6985739	COG0324J	tRNA delta(2)-isopentenylpyrophosphate transferase
Spy49_0731	hflX	NE	736258	737496	412	+	6985740	COG2262R	GTP-binding protein HflX
Spy49_0732	-	NE	737489	738124	211	+	6985741	COG4468G	hypothetical protein Spy49_0732
Spy49_0733	-	E	738139	739068	309	+	6985742	COG1234R	ribonuclease Z
Spy49_0734	-	NE	739228	739833	201	+	6985743	COG0300R	oxidoreductase
Spy49_0735	recJ	NE	739830	742040	736	+	6985744	COG0608L	Single-stranded-DNA-specific exonuclease recJ
Spy49_0736	apt	NE	742190	742708	172	+	6985745	COG0503F	adenine phosphoribosyltransferase
Spy49_0737	dnaD	E	742789	743472	227	+	6985746	COG3935L	DNA replication protein DnaD
Spy49_0738	nth	NE	743469	744125	218	+	6985747	COG0177L	endonuclease III
Spy49_0739	-	E	744197	744883	228	+	6985748	COG2384R	hypothetical protein Spy49_0739
Spy49_0740	-	NE	744873	745661	262	+	6985749	COG0327S	hypothetical protein Spy49_0740
Spy49_0741	-	NE	745701	746825	374	+	6985750	COG0665E	oxidoreductase
Spy49_0742	rmlA	E	746864	747733	289	+	6985751	COG1209M	glucose-1-phosphate thymidylyltransferase
Spy49_0744	rmlC	NC	747733	748326	197	+	6985752	COG1898M	dTDP-4-dehydrorhamnose 3,5-epimerase
Spy49_0745	rmlB	E	748570	749610	346	+	6985753	COG1088M	dTDP-glucose 4,6-dehydratase
Spy49_0746c	-	NE	749693	750832	379	-	6985754	COG4974L	Prophage ps2 integrase
Spy49_0747c	-	NE	750960	751226	88	-	6985755	-	hypothetical protein Spy49_0747c
Spy49_0748c	-	NE	751238	751624	128	-	6985756	-	phage-associated protein
Spy49_0749c	-	C	751627	751977	116	-	6985757	COG1396K	repressor-phage associated
Spy49_0750	-	NC	752273	752443	56	+	6985758	-	hypothetical protein Spy49_0750
Spy49_0751c	-	NC	752469	753257	262	-	6985759	-	hypothetical protein Spy49_0751c
Spy49_0752	-	NC	753308	753499	63	+	6985760	-	antirepressor protein
Spy49_0753	-	NC	753578	753889	103	+	6985761	-	excisionase
Spy49_0754	-	NC	753891	754061	56	+	6985762	-	putative phage associated protein
Spy49_0755	-	NC	754054	754257	67	+	6985763	-	hypothetical protein Spy49_0755
Spy49_0756	-	NE	754254	754640	128	+	6985764	-	hypothetical protein Spy49_0756
Spy49_0757	-	NC	754786	754989	67	+	6985765	-	putative phage associated protein
Spy49_0759	-	NC	755077	755376	99	+	6985766	-	putative phage associated protein
Spy49_0760	-	NE	755376	756533	385	+	6985767	-	phage-associated protein
Spy49_0761	-	NE	756548	757105	185	+	6985768	-	hypothetical protein Spy49_0761
Spy49_0762	-	NC	757148	759070	640	+	6985769	COG0749L	DNA polymerase
Spy49_0763	-	NC	759075	761459	794	+	6985770	COG3378R	DNA primase/helicase-phage associated
Spy49_0764	-	NC	761827	762120	97	+	6985771	-	hypothetical protein Spy49_0764
Spy49_0765	-	NC	762117	763439	440	+	6985772	COG0553KL	helicase-phage associated
Spy49_0766	-	NE	763603	763875	90	+	6985773	-	hypothetical protein Spy49_0766
Spy49_0767	-	NE	764008	764424	138	+	6985774	-	transcriptional activator-phage associated
Spy49_0768	-	NE	764496	764966	156	+	6985775	COG3728L	terminase small subunit
Spy49_0769	-	NE	764956	766233	425	+	6985776	COG1783R	terminase large subunit
Spy49_0770	-	NE	766249	767781	510	+	6985777	-	phage-associated protein
Spy49_0771	-	NE	767747	769189	480	+	6985778	COG5585T	phage-associated protein
Spy49_0772	-	NE	769410	769802	130	+	6985779	-	putative phage associated protein
Spy49_0773	-	NC	769845	770414	189	+	6985780	-	phage protein
Spy49_0774	-	NE	770427	771314	295	+	6985781	-	major head protein (phage associated)
Spy49_0775	-	NE	771326	771682	118	+	6985782	-	phage protein
Spy49_0776	-	NE	771693	771971	92	+	6985783	-	putative phage associated protein
Spy49_0777	-	NE	771968	772312	114	+	6985784	-	hypothetical protein Spy49_0777
Spy49_0778	-	NC	772316	772675	119	+	6985785	-	putative phage associated protein
Spy49_0779	-	C	772687	773286	199	+	6985786	-	phage protein
Spy49_0780	-	NC	773393	773794	133	+	6985787	-	putative phage associated protein
Spy49_0781	-	NE	773869	774102	77	+	6985788	-	putative phage associated protein
Spy49_0782	-	NC	774117	778499	1460	+	6985789	-	hypothetical protein Spy49_0782
Spy49_0783	-	NE	778511	779353	280	+	6985790	-	putative phage associated protein
Spy49_0784	-	NC	779363	781342	659	+	6985791	-	phage protein
Spy49_0785	-	NC	781339	783582	747	+	6985792	-	phage hyaluronidase
Spy49_0786	-	NC	783594	783755	53	+	6985793	-	hypothetical protein Spy49_0786
Spy49_0788	-	NE	783758	784369	203	+	6985794	-	hypothetical protein Spy49_0788
Spy49_0789	-	NE	784379	784834	151	+	6985795	COG4824R	holin protein-phage associated
Spy49_0790	-	NE	784952	785704	250	+	6985796	-	phage-associated cell wall hydrolase
Spy49_0791	-	NE	785870	786157	95	+	6985797	-	hypothetical protein Spy49_0791
Spy49_0792	speH	NE	786363	787073	236	+	6985798	-	streptococcal pyrogenic exotoxin H (SpeH)
Spy49_0793	mutX	NE	787677	788153	158	+	6985799	COG1051F	7,8-dihydro-8-oxoguanine-triphosphatase
Spy49_0794	-	NE	788211	789392	393	+	6985800	COG0628R	hypothetical protein Spy49_0794
Spy49_0795	-	NE	789382	790629	415	+	6985801	COG3063NU	hypothetical protein Spy49_0795
Spy49_0796c	-	NE	790688	792340	550	-	6985802	COG1293K	fibronectin-binding protein-like protein A

Spy49_0797	-	NE	792694	793692	332	+	6985803	COG2984R	ABC transporter substrate-binding protein
Spy49_0798	-	NC	793643	793861	72	+	6985804	-	hypothetical protein Spy49_0798
Spy49_0799	-	NE	794037	794906	289	+	6985805	COG4120R	ABC transport protein
Spy49_0800	-	NE	794903	795661	252	+	6985806	COG1101R	ABC transporter
Spy49_0801	-	E	795865	797526	553	+	6985807	COG0595R	hypothetical protein Spy49_0801
Spy49_0803	estA	NE	797659	798444	261	+	6985808	COG0627R	Tributyrin esterase
Spy49_0804	-	NE	798474	798800	108	+	6985809	-	hypothetical protein Spy49_0804
Spy49_0805	-	NE	798846	800753	635	+	6985810	COG0488R	ABC transporter
Spy49_0807	acoA	NE	801026	802006	326	+	6985811	COG1071C	pyruvate dehydrogenase E1 component subunit alpha
Spy49_0808	acoB	NE	802062	803063	333	+	6985812	COG0022C	pyruvate dehydrogenase E1 component subunit beta
Spy49_0809	acoC	NE	803248	804657	469	+	6985813	COG0508C	branched-chain alpha-keto acid dehydrogenase subunit E2
Spy49_0810	acoL	NE	804984	806747	587	+	6985814	COG1249C	dihydrolipoamide dehydrogenase
Spy49_0811c	hylA	NE	807422	809839	805	-	6985815	-	Extracellular hyaluronate lyase
Spy49_0812	-	NE	810072	811061	329	+	6985816	COG0095H	Lipoate-protein ligase A
Spy49_0813c	cobQ	E	811170	811961	263	-	6985817	COG3442R	Cobyric acid synthase
Spy49_0814c	murC2	E	811961	813304	447	-	6985818	COG0769M	UDP-N-acetylmuramyl tripeptide synthetase
Spy49_0815	-	E	813406	814257	283	+	6985819	COG1624S	hypothetical protein Spy49_0815
Spy49_0816	-	NE	814254	815210	318	+	6985820	COG4856S	hypothetical protein Spy49_0816
Spy49_0817	glmM	E	815264	816616	450	+	6985821	COG1109G	phosphoglucosamine mutase
Spy49_0818	-	NE	816739	817380	213	+	6985822	-	hypothetical protein Spy49_0818
Spy49_0819	hemN	NE	817377	818573	398	+	6985823	COG0635H	coproporphyrinogen III oxidase
Spy49_0820	-	E	818583	819335	250	+	6985824	COG3884I	acyl-ACP thioesterase
Spy49_0821	-	NE	819335	820099	254	+	6985825	COG0647G	phosphatase NagD
Spy49_0822	-	NE	820096	820731	211	+	6985826	COG4478S	hypothetical protein Spy49_0822
Spy49_0823	-	NE	821210	825316	1368	+	6985827	COG3513S	hypothetical protein Spy49_0823
Spy49_0825	-	NE	825316	826185	289	+	6985828	COG1518L	Lipoyl-binding domain-containing protein
Spy49_0826	-	NE	826182	826523	113	+	6985829	COG3512S	hypothetical protein Spy49_0826
Spy49_0827	-	NE	826513	827175	220	+	6985830	-	hypothetical protein Spy49_0827
Spy49_0828	-	NE	827952	828122	56	+	6985831	COG0105F	nucleoside diphosphate kinase
Spy49_0829	lepA-2	NE	828223	830055	610	+	6985832	COG0481M	GTP-binding protein LepA
Spy49_0830	sclB	NC	830338	831435	365	+	6985833	-	SclB protein
Spy49_0831	csrA	NE	831620	832057	145	+	6985834	COG0229O	methionine sulfoxide reductase B
Spy49_0832	-	NE	832187	833206	339	+	6985835	COG2855S	hypothetical protein Spy49_0832
Spy49_0833	-	NE	833413	833838	141	+	6985836	COG2893G	PTS system IIA component
Spy49_0834	-	NC	833865	834356	163	+	6985837	COG3444G	PTS system IIB component
Spy49_0835	-	NE	834449	835183	244	+	6985838	COG3715G	phosphotransferase system (PTS), enzyme II component C
Spy49_0836	-	NE	835180	836019	279	+	6985839	COG3716G	phospotransferase system (PTS), enzyme II component D
Spy49_0837	-	NE	836144	837793	549	+	6985840	COG2972T	two-component sensor histidine kinase
Spy49_0839	-	NE	837797	838585	262	+	6985841	COG4753T	two-component response regulator
Spy49_0840	-	NE	838579	839625	348	+	6985842	COG1840P	iron ABC transporter iron-binding protein
Spy49_0841	-	NE	839741	841138	465	+	6985843	COG1012C	succinate-semialdehyde dehydrogenase NADP+
Spy49_0842	uvrC	NE	841239	843035	598	+	6985844	COG0322L	excinuclease ABC subunit C
Spy49_0843	-	NE	843221	843823	200	+	6985845	COG0778C	NADH dehydrogenase
Spy49_0844	-	NE	843948	845357	469	+	6985846	COG0624E	dipeptidase PepV
Spy49_0845c	trmE	E	845425	846801	458	-	6985847	COG0486R	tRNA modification GTPase TrmE
Spy49_0846	rplJ	NE	847136	847636	166	+	6985848	COG0244J	50S ribosomal protein L10
Spy49_0847	-	NC	847701	847799	32	+	6985849	-	hypothetical protein Spy49_0847
Spy49_0848	rplL	NC	847874	848065	63	+	6985850	COG0222J	50S ribosomal protein L7/L12, partial
Spy49_0849	dacA1	NE	848292	849605	437	+	6985851	COG1686M	Penicillin-binding protein 7
Spy49_0850c	-	NE	849658	850620	320	-	6985852	COG0726G	polysaccharide deacetylase
Spy49_0851	folC1	E	850952	852229	425	+	6985853	COG0285H	dihydrofolate synthase
Spy49_0852	folE	NE	852264	852848	194	+	6985854	COG0302H	GTP cyclohydrolase I
Spy49_0853	folP	NC	852857	853657	266	+	6985855	COG0294H	dihydropteroate synthase
Spy49_0854	folQ	NE	853664	854023	119	+	6985856	COG1539H	dihydroneopterin aldolase
Spy49_0855	folK	NE	854020	854520	166	+	6985857	COG0801H	2-amino-4-hydroxy-6- hydroxymethyldihydropteridine pyrophosphokinase
Spy49_0856	murB	E	854670	855557	295	+	6985858	COG0812M	UDP-N-acetylenolpyruvoylglucosamine reductase
Spy49_0857	potA	NE	855603	856757	384	+	6985859	COG3842E	Spermidine Putrescine transport ATP-binding protein potA
Spy49_0858	potB	NE	856741	857535	264	+	6985860	COG1176E	Spermidine Putrescine ABC transporter permease potB
Spy49_0859	potC	NE	857532	858308	258	+	6985861	COG1177E	Spermidine Putrescine ABC transporter permease potC
Spy49_0860	potD	NE	858301	859374	357	+	6985862	COG0687E	spermidine/putrescine ABC transporter substrate-binding protein
Spy49_0861c	-	NE	859429	860094	221	-	6985863	COG4565KT	two-component response regulator
Spy49_0862c	-	NE	860075	861616	513	-	6985864	COG3290T	two-component sensor histidine kinase
Spy49_0863	malP	NE	861777	863108	443	+	6985865	COG3493C	L-malate permease
Spy49_0864	-	NE	863140	864306	388	+	6985866	COG0281C	NAD-dependent malic enzyme
Spy49_0865c	-	NE	864388	865437	349	-	6985867	COG1063ER	zinc-containing alcohol dehydrogenase
Spy49_0866	aphA	NE	865673	866404	243	+	6985868	COG3700R	acid phosphatase/phosphotransferase
Spy49_0867	-	NE	866582	868114	510	+	6985869	COG0038P	voltage-gated chloride channel protein
Spy49_0868	-	NE	868277	868891	204	+	6985870	COG2755E	hypothetical protein Spy49_0868
Spy49_0869	-	NE	869071	870180	369	+	6985871	COG0628R	hypothetical protein Spy49_0869
Spy49_0870	radC	NE	870247	870927	226	+	6985872	COG2003L	DNA repair protein RadC
Spy49_0871c	-	NE	870929	871624	231	-	6985873	COG2071R	glutamine amidotransferase, class I
Spy49_0872c	-	NE	871634	872278	214	-	6985874	COG2344R	redox-sensing transcriptional repressor Rex
Spy49_0873c	-	NE	872530	872877	115	-	6985875	-	hypothetical protein Spy49_0873c
Spy49_0874c	-	E	872867	873994	375	-	6985876	COG1104E	cysteine desulfurase
Spy49_0875c	prs	NE	873991	874971	326	-	6985877	COG0462FE	ribose-phosphate pyrophosphokinase
Spy49_0876c	-	NE	875111	875689	192	-	6985878	COG4116S	hypothetical protein Spy49_0876c
Spy49_0877	-	NE	875777	876448	223	+	6985879	COG2357S	GTP pyrophosphokinase
Spy49_0878	ppnK	E	876423	877259	278	+	6985880	COG0061G	inorganic polyphosphate/ATP-NAD kinase
Spy49_0879	rluD	NE	877256	878161	301	+	6985881	COG0564J	RluD subfamily ribosomal large subunit pseudouridine synthase
Spy49_0880	eutD	NE	878165	879160	331	+	6985882	COG0280C	phosphotransacetylase
Spy49_0881	-	NE	879286	880041	251	+	6985883	COG4221R	short chain dehydrogenase/reductase
Spy49_0882c	-	NE	880235	880924	229	-	6985884	-	hypothetical protein Spy49_0882c
Spy49_0883	proV	NE	881350	882078	242	+	6985885	COG1125E	L-proline glycine betaine ABC transport ATP- binding protein proV
Spy49_0884	proX	NE	882071	883597	508	+	6985886	COG1732M	L-proline glycine betaine binding ABC transporter protein proX / Osmotic adaptation
Spy49_0885	-	NE	883875	884858	327	+	6985887	COG0516F	guanosine 5\'-monophosphate oxidoreductase
Spy49_0886c	-	NE	884895	885152	85	-	6985888	-	hypothetical protein Spy49_0886c
Spy49_0887	xpt	NE	885163	885744	193	+	6985889	COG0503F	xanthine phosphoribosyltransferase
Spy49_0888	-	NE	885744	887027	427	+	6985890	COG2233F	xanthine permease
Spy49_0891c	-	NE	887091	888029	312	-	6985891	COG1477H	thiamine biosynthesis lipoprotein
Spy49_0892c	-	NE	888082	888267	61	-	6985892	COG1942R	4-oxalocrotonate tautomerase
Spy49_0893	tdk	NE	888405	888974	189	+	6985893	COG1435F	thymidine kinase
Spy49_0894	prfA	E	889009	890088	359	+	6985894	COG0216J	peptide chain release factor 1
Spy49_0895	hemK	NC	890088	890927	279	+	6985895	COG2890J	protoporphyrinogen oxidase
Spy49_0896	-	E	890911	891501	196	+	6985896	COG0009J	Sua5/YciO/YrdC family protein
Spy49_0897	-	NE	891519	891971	150	+	6985897	COG0456R	hypothetical protein Spy49_0897
Spy49_0898	glyA	NE	891961	893217	418	+	6985898	COG0112E	serine hydroxymethyltransferase
Spy49_0899	-	NC	893224	894201	325	+	6985899	-	hypothetical protein Spy49_0899
Spy49_0900	-	NE	894202	894801	199	+	6985900	-	Pneumococcal vaccine antigen A-like protein
Spy49_0901	-	NC	894811	896535	574	+	6985901	COG1132V	ABC transporter ATP-binding protein/permease
Spy49_0902	-	NE	896532	898247	571	+	6985902	COG1132V	ABC transporter
Spy49_0903	nox1	NE	898500	899870	456	+	6985903	COG0446R	NADH oxidase
Spy49_0904c	ldh	C	900029	901012	327	-	6985904	COG0039C	L-lactate dehydrogenase
Spy49_0905	gyrA	E	901203	903689	828	+	6985905	COG0188L	DNA gyrase subunit A
Spy49_0906	-	C	903709	904458	249	+	6985906	COG3764M	Sortase
Spy49_0907	-	C	904538	904954	138	+	6985907	COG0346E	lactoylglutathione lyase
Spy49_0908	-	NE	905603	906007	134	+	6985908	-	hypothetical protein Spy49_0908
Spy49_0909c	-	NE	906057	906968	303	-	6985909	COG1597IR	hypothetical protein Spy49_0909c

Spy49_0910c	hlyIII	E	907220	907735	171	-	6985910	COG1272R	hemolysin III, partial
Spy49_0911c	-	NE	907732	908172	146	-	6985911	-	hypothetical protein Spy49_0911c
Spy49_0912	rbgA	E	908391	909239	282	+	6985912	COG1161R	ribosomal biogenesis GTPase
Spy49_0913	rnhB	NE	909229	910020	263	+	6985913	COG0164L	ribonuclease HII
Spy49_0914	smf	NE	910085	910921	278	+	6985914	COG0758LU	DNA processing protein
Spy49_0915	topA	E	911028	913157	709	+	6985915	COG0550L	DNA topoisomerase I
Spy49_0916c	-	NE	913233	913715	160	-	6985916	-	LysR family transcriptional regulator
Spy49_0917c	-	NC	913722	913844	40	-	6985917	-	LysR family transcriptional regulator
Spy49_0918	-	NE	914130	914696	188	+	6985918	COG3641R	Regulatory protein
Spy49_0919	-	NE	914711	915703	330	+	6985919	COG1052CHR	D-lactate dehydrogenase
Spy49_0920	-	E	915808	916473	221	+	6985920	-	acid tolerance SatD-like protein
Spy49_0921	-	NE	916466	917182	238	+	6985921	-	hypothetical protein Spy49_0921
Spy49_0922	gid	NC	917333	918679	448	+	6985922	COG1206J	tRNA (uracil-5-)-methyltransferase Gid
Spy49_0923	-	NE	918856	920238	460	+	6985923	COG5016C	oxaloacetate decarboxylase
Spy49_0924	-	NE	920281	920595	104	+	6985924	-	hypothetical protein Spy49_0924
Spy49_0925	-	NE	920595	920945	116	+	6985925	COG4770I	Biotin carboxyl carrier protein of oxaloacetate decarboxylase
Spy49_0926	-	NC	920955	922085	376	+	6985926	COG1883C	Oxaloacetate decarboxylase subunit beta
Spy49_0927c	citG	NE	922286	923170	294	-	6985927	COG1767H	2-(5-triphosphoribosyl)-3- dephosphocoenzyme-A synthase
Spy49_0928c	-	NE	923163	923858	231	-	6985928	COG1802K	GntR family transcriptional regulator
Spy49_0929c	-	NE	924016	925422	468	-	6985929	COG2851C	citrate transporter
Spy49_0930	-	NE	925799	926122	107	+	6985930	-	hypothetical protein Spy49_0930
Spy49_0933	-	NE	926611	927732	373	+	6985931	COG1883C	Oxaloacetate decarboxylase subunit beta
Spy49_0934	citD	NE	927920	928228	102	+	6985932	COG3052C	citrate lyase subunit gamma
Spy49_0936	citE	NC	928216	929103	295	+	6985933	COG2301G	citrate lyase subunit beta
Spy49_0937	citF	NE	929106	930638	510	+	6985934	COG3051C	citrate lyase subunit alpha
Spy49_0938	citX	NE	930535	931209	224	+	6985935	COG3697HI	2\'-(5\'\'-triphosphoribosyl)-3\'-dephospho-CoA:apo-citrate lyase
Spy49_0939	oadA	NE	931224	932618	464	+	6985936	COG5016C	oxaloacetate decarboxylase
Spy49_0940c	citC	NE	932729	933781	350	-	6985937	COG3053C	citrate (pro-3S)-lyase ligase
Spy49_0942c	-	NE	933968	934351	127	-	6985938	-	hypothetical protein Spy49_0942c
Spy49_0943c	xerS	C	935045	936115	356	-	6985939	COG4974L	site-specific tyrosine recombinase XerS
Spy49_0944	-	NE	936585	937283	232	+	6985940	COG2932K	repressor protein
Spy49_0945c	ffh	E	937400	938962	520	-	6985941	COG0541U	signal recognition particle subunit Ffh SRP54
Spy49_0946c	-	NC	938977	939318	113	-	6985942	COG2739S	DNA-binding protein
Spy49_0947c	-	NE	939407	940105	232	-	6985943	COG2188K	GntR family transcriptional regulator
Spy49_0948c	-	NE	940188	941477	429	-	6985944	-	hypothetical protein Spy49_0948c
Spy49_0949	guaA	C	941789	943351	520	+	6985945	COG0519F	GMP synthase
Spy49_0951c	murM2	NE	943393	944604	403	-	6985946	COG2348V	antimicrobial resistance factor
Spy49_0952c	-	NE	944959	946524	521	-	6985947	COG0488R	ABC transporter ATP-binding protein
Spy49_0953c	-	NE	946637	947194	185	-	6985948	COG4720S	hypothetical protein Spy49_0953c
Spy49_0954c	pdxK	NE	947172	948038	288	-	6985949	COG2240H	pyridoxamine kinase
Spy49_0955	-	NE	948128	949396	422	+	6985950	COG1167KE	transcriptional regulator/aminotransferase
Spy49_0957c	-	NE	949867	950223	118	-	6985951	COG1328F	anaerobic ribonucleotide reductase
Spy49_0958c	-	E	950545	952122	525	-	6985952	COG1502I	Cardiolipin synthetase
Spy49_0959c	fhs1	C	952207	953877	556	-	6985953	COG2759F	formate--tetrahydrofolate ligase
Spy49_0960c	lplA	NE	954005	955024	339	-	6985954	COG0095H	Lipoate-protein ligase A
Spy49_0961c	-	NE	955071	955952	293	-	6985955	COG0846K	hypothetical protein Spy49_0961c
Spy49_0962c	-	NE	955945	956757	270	-	6985956	COG2110R	hypothetical protein Spy49_0962c
Spy49_0963c	-	NE	956750	957082	110	-	6985957	COG0509E	glycine cleavage system H protein
Spy49_0964c	-	NE	957124	958122	332	-	6985958	COG2141C	hypothetical protein Spy49_0964c
Spy49_0965c	-	NE	958119	959318	399	-	6985959	COG1902C	trimethylamine dehydrogenase
Spy49_0966c	-	NE	959315	960151	278	-	6985960	COG0095H	Lipoate-protein ligase A
Spy49_0967	dpfB	E	960347	961039	230	+	6985961	COG0452H	phosphopantothenate--cysteine ligase
Spy49_0968	dfp	E	961032	961577	181	+	6985962	COG0452H	phosphopantothenoylcysteine decarboxylase
Spy49_0969	-	C	961636	962205	189	+	6985963	COG4684S	hypothetical protein Spy49_0969
Spy49_0970	pgmA	E	962381	964099	572	+	6985964	COG1109G	phosphoglucomutase
Spy49_0971c	-	NE	964312	965268	318	-	6985965	COG1079R	sugar ABC transporter
Spy49_0972c	-	NE	965270	966334	354	-	6985966	COG4603R	sugar ABC transporter
Spy49_0973c	-	NE	966327	967859	510	-	6985967	COG3845R	sugar ABC transporter
Spy49_0974c	-	NE	967998	969050	350	-	6985968	COG1744R	lipoprotein
Spy49_0975c	cdd	NE	969144	969533	129	-	6985969	COG0295F	cytidine deaminase
Spy49_0976c	-	NE	970186	970779	197	-	6985970	COG2813J	hypothetical protein Spy49_0976c
Spy49_0977	coaA	E	971047	971967	306	+	6985971	COG1072H	pantothenate kinase
Spy49_0978	rpsT	NC	972036	972269	77	+	6985972	COG0268J	30S ribosomal protein S20
Spy49_0979c	ciaH	C	972394	973704	436	-	6985973	COG2205T	histidine kinase
Spy49_0980c	ciaR	E	973697	974371	224	-	6985974	COG0745TK	CiaR family transcriptional regulator
Spy49_0981c	pepN	NE	974718	977255	845	-	6985975	COG0308E	lysyl-aminopeptidase
Spy49_0982c	phoU	NE	977460	978113	217	-	6985976	COG0704P	phosphate uptake regulatory protein
Spy49_0983c	pstB	NC	978182	978940	252	-	6985977	COG1117P	phosphate transporter ATP-binding protein
Spy49_0984c	pstB2	C	978953	979756	267	-	6985978	COG1117P	phosphate transporter ATP-binding protein
Spy49_0985c	pstA	NC	979772	980659	295	-	6985979	COG0581P	phosphate ABC transporter permease
Spy49_0986	pstC	NC	980649	981584	311	-	6985980	COG0573P	phosphate ABC transporter permease
Spy49_0987c	pstS	NC	981595	982461	288	-	6985981	COG0226P	phosphate ABC transporter periplasmic phosphate-binding protein pstS
Spy49_0988c	-	E	982600	983910	436	-	6985982	COG0144J	nucleolar protein
Spy49_0989c	-	NE	983913	984701	262	-	6985983	COG0483G	Myo-inositol-1(or 4)-monophosphatase
Spy49_0990c	-	NE	984691	984969	92	-	6985984	COG4476S	hypothetical protein Spy49_0990c
Spy49_0991c	spxA	NE	984971	985375	134	-	6985985	COG1393P	transcriptional regulator Spx
Spy49_0992c	-	E	985418	986350	310	-	6985986	COG0196H	bifunctional riboflavin kinase/FMN adenylyltransferase
Spy49_0993c	truB	NE	986340	987263	307	-	6985987	COG0130J	tRNA pseudouridine synthase B
Spy49_0994c	-	NE	987379	988719	446	-	6985988	COG4487S	hypothetical protein Spy49_0994c
Spy49_0995c	-	NE	988816	989772	318	-	6985989	COG4129S	hypothetical protein Spy49_0995c
Spy49_0996c	-	NE	989783	990379	198	-	6985990	COG0732V	hypothetical protein Spy49_0996c
Spy49_0997c	-	NE	990471	993107	878	-	6985991	COG0577V	ABC transporter permease
Spy49_0998c	-	NE	993122	993823	233	-	6985992	COG1136V	ABC transporter
Spy49_0999	-	NE	993941	994483	180	+	6985993	COG1309K	TetR family transcriptional regulator
Spy49_1000c	-	NE	994638	995267	209	-	6985994	COG0490P	transcriptional regulator
Spy49_1001c	-	NE	995430	995918	162	-	6985995	COG1302S	hypothetical protein Spy49_1001c
Spy49_1002c	-	NE	995929	996129	66	-	6985996	COG3237S	hypothetical protein Spy49_1002c
Spy49_1003c	-	NE	996170	996709	179	-	6985997	COG1302S	hypothetical protein Spy49_1003c
Spy49_1004c	-	NC	996722	996910	62	-	6985998	COG5547S	hypothetical protein Spy49_1004c
Spy49_1005c	-	NE	996921	997508	195	-	6985999	-	hypothetical protein Spy49_1005c
Spy49_1006c	-	NE	997569	997817	82	-	6986000	COG2261S	hypothetical protein Spy49_1006c
Spy49_1007c	pcrA	E	998180	1000498	772	-	6986001	COG0210L	ATP-dependent DNA helicase
Spy49_1008	-	NC	1001027	1002349	440	+	6986002	COG1115E	amino acid symporter
Spy49_1009	-	NE	1002469	1003704	411	+	6986003	COG0053P	cation efflux system protein
Spy49_1010c	cfa	NE	1004074	1004847	257	-	6986004	-	CAMP factor
Spy49_1011c	-	NE	1005217	1006053	278	-	6986005	COG0834ET	amino acid ABC transporter substrate-binding protein
Spy49_1012c	-	NE	1006069	1006698	209	-	6986006	COG1126E	amino acid ABC transporter ATP-binding protein
Spy49_1013c	-	NE	1006708	1007349	213	-	6986007	COG0765E	amino acid ABC transporter permease
Spy49_1014c	-	NE	1007456	1007791	111	-	6986008	COG2824P	PhnA protein
Spy49_1015c	glmS	E	1007987	1009801	604	-	6986009	COG0449M	glucosamine--fructose-6-phosphate aminotransferase
Spy49_1016c	sipC	NE	1009977	1010534	185	-	6986010	COG0681U	signal peptidase I
Spy49_1017c	pyk	E	1010752	1012254	500	-	6986011	COG0469G	pyruvate kinase
Spy49_1018c	pfk	E	1012317	1013330	337	-	6986012	COG0205G	6-phosphofructokinase
Spy49_1019c	dnaE	E	1013410	1016520	1036	-	6986013	COG0587L	DNA polymerase III DnaE
Spy49_1020	-	NE	1016705	1017076	123	+	6986014	COG1725K	GntR family transcriptional regulator
Spy49_1021	-	C	1017076	1017774	232	+	6986015	COG1131V	ABC transporter
Spy49_1022	-	C	1017784	1018569	261	+	6986016	-	hypothetical protein Spy49_1022

Spy49_1023c	-	E	1018706	1019311	201	-	6986017	COG0398S	hypothetical protein Spy49_1023c
Spy49_1024	-	NE	1020050	1020670	206	+	6986019	-	hypothetical protein Spy49_1024
Spy49_1025c	glgP	NE	1020927	1023191	754	-	6986020	COG0058G	glycogen phosphorylase
Spy49_1026c	malQ	NE	1023226	1024719	497	-	6986021	COG1640G	4-alpha-glucanotransferase
Spy49_1027c	malR	NE	1024834	1025853	339	-	6986022	COG1609K	maltose operon transcriptional repressor
Spy49_1028	malE	NE	1026097	1027344	415	+	6986023	COG2182G	maltose maltodextrin ABC transporter substrate binding periplasmic protein malE
Spy49_1029	malF	NE	1027618	1028979	453	+	6986024	COG1175G	maltose ABC transporter permease
Spy49_1030	malG	NE	1028979	1029815	278	+	6986025	COG3833G	maltose AVC transporter permease
Spy49_1032	-	NE	1031207	1031431	74	+	6986027	-	transposase for insertion-like sequence element IS1161
Spy49_1033c	-	NE	1031496	1032479	327	-	6986028	COG0657I	esterase
Spy49_1034c	dltD	NE	1032510	1033760	416	-	6986029	COG3966M	Protein dltD
Spy49_1035c	dltC	NE	1033753	1033992	79	-	6986030	COG0236IQ	D-alanine--poly(phosphoribitol) ligase subunit 2
Spy49_1036c	dltB	NC	1034010	1035266	418	-	6986031	COG1696M	hypothetical protein Spy49_1036c
Spy49_1037c	dltA	NC	1035263	1036801	512	-	6986032	COG1020Q	D-alanine--poly(phosphoribitol) ligase subunit 1
Spy49_1038c	-	NC	1036813	1036956	47	-	6986033	-	hypothetical protein Spy49_1038c
Spy49_1039c	uvrB	NE	1037219	1039210	663	-	6986034	COG0556L	excinuclease ABC subunit B
Spy49_1040	glnP	C	1039403	1041577	724	+	6986035	COG0765E	glutamine ABC transporter glutamine-binding protein/permease
Spy49_1041	glnQ	C	1041577	1042317	246	+	6986036	COG1126E	glutamine transport ATP-binding protein glnQ
Spy49_1042c	-	NC	1042465	1042617	50	-	6986037	-	hypothetical protein Spy49_1042c
Spy49_1043c	-	NE	1042614	1043993	459	-	6986038	COG1455G	PTS system IIC component
Spy49_1044c	-	NE	1044171	1044620	149	-	6986039	-	hypothetical protein Spy49_1044c
Spy49_1045c	-	NE	1044617	1044952	111	-	6986040	COG1447G	PTS enzyme III
Spy49_1046c	-	NE	1044955	1045266	103	-	6986041	COG1440G	PTS system enzyme II
Spy49_1047c	-	NC	1045289	1047283	664	-	6986042	COG3711K	transcriptional antiterminator
Spy49_1048c	-	NE	1047389	1048483	364	-	6986043	COG3589S	hypothetical protein Spy49_1048c
Spy49_1049c	-	NE	1048492	1049892	466	-	6986044	COG2723G	beta-glucosidase
Spy49_1050	-	NE	1050117	1050812	231	+	6986045	COG3201H	ribosyl nicotinamide transporter
Spy49_1051c	-	NC	1050817	1050975	52	-	6986046	-	hypothetical protein Spy49_1051c
Spy49_1053c	obgE	E	1051044	1052357	437	-	6986047	COG0536R	GTPase ObgE
Spy49_1054	-	NE	1052786	1053181	131	+	6986048	COG2963L	hypothetical protein Spy49_1054
Spy49_1055	-	NE	1053321	1054199	292	+	6986049	COG2801L	transposase, IS861
Spy49_1056	-	NE	1054597	1055097	166	+	6986050	COG2963L	hypothetical protein Spy49_1056
Spy49_1057	-	NE	1055100	1055906	268	+	6986051	COG2801L	transposase, IS861
Spy49_1059	-	NE	1057303	1058613	436	+	6986052	COG0471P	integral membrane protein
Spy49_1060	-	NC	1058770	1058985	71	+	6986053	-	hypothetical protein Spy49_1060
Spy49_1062	-	NE	1059129	1059314	61	+	6986054	-	Insertion element iso-IS904 coding for a transposase
Spy49_1064c	rsuA	NC	1059757	1060488	243	-	6986055	COG1187J	16S pseudouridylate synthase
Spy49_1065	-	NE	1060603	1060968	121	+	6986056	COG5496R	hypothetical protein Spy49_1065
Spy49_1066c	-	NE	1061088	1062308	406	-	6986057	COG2814G	hypothetical protein Spy49_1066c
Spy49_1067	-	NE	1062720	1064165	481	+	6986058	COG4868S	hypothetical protein Spy49_1067
Spy49_1070c	-	NE	1064270	1064671	133	-	6986059	COG2050Q	phenylacetic acid degradation protein PaaI
Spy49_1071c	-	NE	1064835	1065269	144	-	6986060	COG2820F	Uridine phosphorylase
Spy49_1072c	-	NE	1065688	1067046	452	-	6986061	COG2265J	RNA methyltransferase
Spy49_1073c	psr	NE	1067128	1068579	483	-	6986062	COG1316K	PBP 5 synthesis repressor
Spy49_1074c	aroK	NE	1068787	1069278	163	-	6986063	COG0703E	shikimate kinase
Spy49_1075c	aroA1	NE	1069271	1070554	427	-	6986064	COG0128E	3-phosphoshikimate 1-carboxyvinyltransferase
Spy49_1076c	-	NE	1070665	1071630	321	-	6986065	COG1295S	ribonuclease BN
Spy49_1077c	map	E	1071632	1072492	286	-	6986066	COG0024J	methionine aminopeptidase
Spy49_1078c	-	NC	1072508	1073791	427	-	6986067	COG4109K	hypothetical protein Spy49_1078c
Spy49_1079c	-	NE	1073800	1074342	180	-	6986068	COG1670J	acetyl transferase
Spy49_1080c	grab	NE	1074609	1075148	179	-	6986069	-	Protein G-related alpha 2 macroglobulin-binding protein (GRAB)
Spy49_1082c	murZ	NE	1075506	1076765	419	-	6986070	COG0766M	UDP-N-acetylglucosamine 1-carboxyvinyltransferase
Spy49_1083c	metK	E	1076939	1078135	398	-	6986071	COG0192H	S-adenosylmethionine synthetase
Spy49_1084c	inlA	NE	1078672	1081050	792	-	6986072	COG4886S	internalin
Spy49_1085	birA	E	1081425	1082195	256	+	6986073	COG0340H	Biotin-protein ligase / Biotin operon repressor
Spy49_1086c	-	NE	1082170	1082373	67	-	6986074	-	hypothetical protein Spy49_1086c
Spy49_1087c	dnaX	E	1082469	1084139	556	-	6986075	COG2812L	DNA polymerase III subunits gamma/tau
Spy49_1088c	-	NE	1084139	1084636	165	-	6986076	COG1956T	hypothetical protein Spy49_1088c
Spy49_1089	-	NE	1084842	1085591	249	+	6986077	COG2339S	hypothetical protein Spy49_1089
Spy49_1090c	udk	NE	1085989	1086615	208	-	6986078	COG0572F	uridine kinase
Spy49_1091	deaD2	NE	1086713	1087798	361	+	6986079	COG0513LKJ	RNA helicase
Spy49_1092c	-	NE	1087909	1089183	424	-	6986080	COG0726G	polysaccharide deacetylase
Spy49_1093c	gapN	E	1089304	1090707	467	-	6986081	COG1012C	glyceraldehyde-3-phosphate dehydrogenase (NADP)
Spy49_1094c	ptsI	C	1090892	1092625	577	-	6986082	COG1080G	phosphoenolpyruvate-protein phosphotransferase of PTS system
Spy49_1095c	ptsH	NC	1092630	1092893	87	-	6986083	COG1925G	phosphocarrier protein HPr
Spy49_1096	nrdH	NE	1093253	1093504	83	+	6986084	COG0695O	glutaredoxin
Spy49_1097	nrdE1	NE	1093524	1095683	719	+	6986085	COG0209F	ribonucleotide-diphosphate reductase subunit alpha
Spy49_1098	nrdF1	NE	1095664	1095981	105	+	6986086	-	hypothetical protein Spy49_1098
Spy49_1099	nrdF	NE	1096016	1096975	319	+	6986087	COG0208F	ribonucleotide-diphosphate reductase subunit beta
Spy49_1100	-	NE	1096950	1098263	437	+	6986088	COG0038P	chloride channel protein
Spy49_1101	-	NC	1098401	1098694	97	+	6986089	COG2963L	hypothetical protein Spy49_1101
Spy49_1103	-	NC	1099127	1099297	56	+	6986090	-	transposase
Spy49_1104	-	NE	1099337	1099558	73	+	6986091	COG2801L	transposase
Spy49_1105c	-	NE	1099651	1100346	231	-	6986092	COG1266R	hypothetical protein Spy49_1105c
Spy49_1106c	-	NE	1100365	1101126	253	-	6986093	-	hypothetical protein Spy49_1106c
Spy49_1107c	-	NE	1101123	1101353	76	-	6986094	COG1476K	Cro/CI family transcriptional regulator
Spy49_1108c	alaS	E	1101699	1104317	872	-	6986095	COG0013J	alanyl-tRNA synthetase
Spy49_1109c	prsA	NE	1104704	1105759	351	-	6986096	COG0760O	foldase PrsA
Spy49_1110c	-	NE	1105822	1106529	235	-	6986097	COG4122R	methyltransferase
Spy49_1111c	-	NE	1106595	1107791	398	-	6986098	COG2814G	oxalate:formate antiporter
Spy49_1112c	pepB	NE	1108168	1109973	601	-	6986099	COG1164E	oligopeptidase PepB
Spy49_1113c	-	NE	1109986	1110948	320	-	6986100	COG4469R	transcription factor
Spy49_1114c	-	NE	1111245	1111961	238	-	6986101	COG1187J	16S pseudouridylate synthetase
Spy49_1115c	nagB	NE	1112080	1112784	234	-	6986102	COG0363G	glucosamine-6-phosphate deaminase
Spy49_1116	queA	NE	1112986	1114014	342	+	6986103	COG0809J	S-adenosylmethionine:tRNA ribosyltransferase-isomerase
Spy49_1117	-	NE	1114021	1115241	406	+	6986104	-	hypothetical protein Spy49_1117
Spy49_1119c	-	NE	1115355	1115945	196	-	6984357	COG1686M	hypothetical protein Spy49_1119c
Spy49_1120c	-	NE	1115942	1116190	82	-	6984358	-	hypothetical protein Spy49_1120c
Spy49_1121c	-	NC	1116175	1116402	75	-	6984359	-	hypothetical protein Spy49_1121c
Spy49_1122c	sodM	NE	1116524	1117168	214	-	6984360	COG0605P	superoxide dismutase
Spy49_1123c	holA	E	1117265	1118305	346	-	6984361	COG1466L	DNA polymerase III subunit delta
Spy49_1124c	comEC	NE	1118376	1120619	747	-	6984362	COG2333R	DNA internalization-related competence protein ComEC/Rec2
Spy49_1126c	comE	NE	1120600	1121262	220	-	6984363	COG1555L	competence protein
Spy49_1127c	-	E	1121462	1122202	246	-	6984364	COG0204I	1-acyl-sn-glycerol-3-phosphate acyltransferase
Spy49_1128	-	NE	1122242	1123096	284	+	6984365	COG4123R	hypothetical protein Spy49_1128
Spy49_1129	-	NE	1123086	1123364	92	+	6984366	COG2827L	hypothetical protein Spy49_1129
Spy49_1130c	kup	NE	1123388	1125388	666	-	6984367	COG3158P	potassium uptake protein
Spy49_1132c	deaD1	NE	1125516	1127135	539	-	6984368	COG0513LKJ	ATP-dependent RNA helicase
Spy49_1133c	prfC	NE	1127442	1128986	514	-	6984369	COG4108J	peptide chain release factor 3
Spy49_1135c	-	NE	1129234	1129929	231	-	6984370	COG5522S	hypothetical protein Spy49_1135c
Spy49_1136c	murF	E	1130009	1131400	463	-	6984371	COG0770M	UDP-N-acetylmuramoyl-tripeptide-D-alanyl-D-alanine ligase
Spy49_1137c	ddl	E	1131591	1132637	348	-	6984372	COG1181M	D-alanyl-alanine synthetase A
Spy49_1138c	recR	C	1132738	1133334	198	-	6984373	COG0353L	recombination protein RecR
Spy49_1139c	-	NE	1133381	1133572	63	-	6984374	-	hypothetical protein Spy49_1139c
Spy49_1140c	fdhC	NE	1134132	1134947	271	-	6984375	COG2116P	formate/nitrite transporter family member
Spy49_1141c	-	NE	1135034	1135576	180	-	6984376	-	hypothetical protein Spy49_1141c
Spy49_1142c	-	NE	1135737	1136258	173	-	6984377	COG1827R	transcriptional regulator, biotin repressor family

Spy49_1143c	gpmA	E	1136365	1137060	231	-	6984378	COG0588G	phosphoglyceromutase
Spy49_1144	pyrD	NE	1137357	1138238	293	+	6984379	COG0167F	dihydroorotate dehydrogenase 1A
Spy49_1145c	-	NC	1138293	1138466	57	-	6984380	-	hypothetical protein Spy49_1145c
Spy49_1146c	cadA	NC	1138538	1140400	620	-	6984381	COG2217P	heavy metal-transporting ATPase
Spy49_1147c	-	NC	1140732	1141007	91	-	6984382	COG0776L	DNA-binding protein HU
Spy49_1148c	-	NE	1141106	1141693	195	-	6984383	COG4698S	hypothetical protein Spy49_1148c
Spy49_1149c	-	NE	1141671	1142513	280	-	6984384	COG2755E	Lipase/acylhydrolase
Spy49_1150c	-	C	1142506	1143357	283	-	6984385	COG1307S	DegV family protein
Spy49_1151c	-	NE	1143573	1144529	318	-	6984386	-	hypothetical protein Spy49_1151c
Spy49_1152c	recN	NE	1144701	1146362	553	-	6984387	COG0497L	DNA repair and genetic recombination protein
Spy49_1153c	-	NE	1146383	1146853	156	-	6984388	COG1438K	repressor protein
Spy49_1154c	-	NE	1146840	1147667	275	-	6984389	COG1189J	hemolysin
Spy49_1155c	fps	NE	1147867	1148532	221	-	6984390	COG0142H	Octaprenyl-diphosphate synthase / Dimethylallyltransferase / Geranyltranstransferase / Geranylgerany...
Spy49_1156c	xseB	NE	1148532	1148747	71	-	6984391	COG1722L	exodeoxyribonuclease VII small subunit
Spy49_1157c	xseA	NE	1148725	1150065	446	-	6984392	COG1570L	exodeoxyribonuclease VII large subunit
Spy49_1158c	folD	NC	1150218	1151072	284	-	6984393	COG0190H	bifunctional 5,10-methylene-tetrahydrofolate dehydrogenase/ 5,10-methylene-tetrahydrofolate cyclohyd...
Spy49_1159c	-	NE	1151280	1152974	564	-	6984394	COG1109G	phosphoglucomutase/phosphomannomutase
Spy49_1160c	phr	NE	1153152	1154561	469	-	6984395	COG0415L	deoxyribodipyrimidine photolyase
Spy49_1161c	gtr	NE	1154710	1155444	244	-	6984396	COG1126E	amino acid ABC transporter
Spy49_1162c	-	NE	1155444	1156130	228	-	6984397	COG0765E	amino acid ABC transporter
Spy49_1163c	-	NC	1156257	1156487	76	-	6984398	COG4703S	hypothetical protein Spy49_1163c
Spy49_1164	clpE	NE	1156785	1159067	760	+	6984399	COG0542O	ATP-dependent protease
Spy49_1165	mutT	NE	1159195	1159650	151	+	6984400	COG0494LR	MutT/nudix family protein
Spy49_1166	-	NE	1159701	1160003	100	+	6984401	-	hypothetical protein Spy49_1166
Spy49_1167c	ileS	E	1160268	1163024	918	-	6984402	COG0060J	isoleucyl-tRNA synthetase
Spy49_1168c	divIVA	NC	1163342	1164100	252	-	6984403	COG3599D	cell-division initiation protein
Spy49_1169c	-	NE	1164110	1164901	263	-	6984404	COG2302S	hypothetical protein Spy49_1169c
Spy49_1170c	-	NE	1164901	1165155	84	-	6984405	COG0762S	YGGT family protein
Spy49_1171c	-	C	1165160	1165828	222	-	6984406	COG1799S	hypothetical protein Spy49_1171c
Spy49_1172c	-	NE	1165828	1166499	223	-	6984407	COG0325R	hypothetical protein Spy49_1172c
Spy49_1173c	ftsZ	E	1166502	1167821	439	-	6984408	COG0206D	cell division protein FtsZ
Spy49_1174c	ftsA	E	1167845	1169209	454	-	6984409	COG0849D	cell division protein FtsA
Spy49_1175c	ftsQ	E	1169420	1170568	382	-	6984410	COG1589M	cell division protein FtsQ
Spy49_1176c	murG	E	1170569	1171651	360	-	6984411	COG0707M	undecaprenyldiphospho-muramoylpentapeptide beta-N- acetylglucosaminyltransferase
Spy49_1177c	murD	E	1171651	1173009	452	-	6984412	COG0771M	UDP-N-acetylmuramoyl-L-alanyl-D-glutamate synthetase
Spy49_1178c	-	NE	1173365	1173616	83	-	6984413	-	hypothetical protein Spy49_1178c
Spy49_1179c	-	NE	1173738	1175579	613	-	6984414	COG1217T	GTP-binding protein TypA/BipA
Spy49_1180c	-	NE	1175762	1176151	129	-	6984415	COG0607P	hypothetical protein Spy49_1180c
Spy49_1181c	-	NE	1176161	1177132	323	-	6984416	COG1940KG	glucokinase
Spy49_1182c	-	NE	1177137	1177340	67	-	6984417	COG4483S	hypothetical protein Spy49_1182c
Spy49_1183c	-	NE	1177482	1178009	175	-	6984418	COG0783P	peroxide resistance protein
Spy49_1184	-	NE	1178237	1178863	208	+	6984419	-	Type IV prepilin peptidase-like protein
Spy49_1185c	-	NE	1178945	1180024	359	-	6984420	COG0820R	ribosomal RNA large subunit methyltransferase N
Spy49_1186c	-	NE	1180028	1180648	206	-	6984421	COG4470S	hypothetical protein Spy49_1186c
Spy49_1187c	-	NE	1181096	1182073	325	-	6984422	COG1609K	ribose operon repressor
Spy49_1188c	-	NE	1182487	1183524	345	-	6984423	COG3480T	ATP-dependent protease La
Spy49_1189c	coaD	E	1183511	1184002	163	-	6984424	COG0669H	phosphopantetheine adenylyltransferase
Spy49_1190c	-	NE	1183992	1184531	179	-	6984425	COG0742L	hypothetical protein Spy49_1190c
Spy49_1191c	asnA	NE	1184654	1185646	330	-	6984426	COG2502E	asparagine synthetase AsnA
Spy49_1192c	arcC	NC	1185959	1186909	316	-	6984427	COG0549E	carbamate kinase
Spy49_1193c	-	NE	1186929	1188260	443	-	6984428	COG0624E	hypothetical protein Spy49_1193c
Spy49_1194c	-	NE	1188277	1189578	433	-	6984429	COG1288S	hypothetical protein Spy49_1194c
Spy49_1195c	arcB	NC	1189941	1190954	337	-	6984430	COG0078E	ornithine carbamoyltransferase
Spy49_1196c	-	NE	1190979	1191419	146	-	6984431	COG2153R	hypothetical protein Spy49_1196c
Spy49_1197c	arcA	NE	1191519	1192754	411	-	6984432	COG2235E	arginine deiminase
Spy49_1198c	-	NE	1193028	1193708	226	-	6984433	COG0664T	Crp/Fnr family transcriptional regulator
Spy49_1199	argR	NE	1193850	1194323	157	+	6984434	COG1438K	arginine repressor
Spy49_1200c	-	NE	1194490	1195206	238	-	6984435	COG3382S	hypothetical protein Spy49_1200c
Spy49_1201c	-	NE	1195220	1196299	359	-	6984436	COG2315S	hypothetical protein Spy49_1201c
Spy49_1202c	yesM	NE	1196372	1198105	577	-	6984437	COG2972T	two-component sensor histidine kinase
Spy49_1203c	yesN	NE	1198102	1198842	246	-	6984438	COG4753T	two-component response regulator
Spy49_1204c	msrA1	NE	1198930	1200036	368	-	6984439	COG0225O	peptide methionine sulfoxide reductase msrA/msrB
Spy49_1205c	-	NE	1200079	1200702	207	-	6984440	COG1225O	hypothetical protein Spy49_1205c
Spy49_1206c	ccdA	NE	1200715	1201404	229	-	6984441	COG0785O	cytochrome C biogenesis protein
Spy49_1207c	-	NE	1202097	1202390	97	-	6984442	COG1343L	hypothetical protein Spy49_1207c
Spy49_1208c	-	NE	1202401	1203426	341	-	6984443	COG1518L	hypothetical protein Spy49_1208c
Spy49_1209c	-	NE	1203423	1204097	224	-	6984444	COG1468L	hypothetical protein Spy49_1209c
Spy49_1210c	-	NE	1204099	1204947	282	-	6984445	COG3649L	hypothetical protein Spy49_1210c
Spy49_1211c	-	NC	1204952	1206847	631	-	6984446	-	hypothetical protein Spy49_1211c
Spy49_1212c	-	NE	1206847	1207575	242	-	6984447	-	hypothetical protein Spy49_1212c
Spy49_1213c	-	NE	1207708	1210110	800	-	6984448	COG1203R	ATP-dependent RNA helicase SrmB
Spy49_1215c	valS	E	1210270	1212918	882	-	6984449	COG0525J	valyl-tRNA synthetase
Spy49_1216c	-	NE	1212920	1213483	187	-	6984450	-	hypothetical protein Spy49_1216c
Spy49_1217c	-	NC	1213480	1213659	59	-	6984451	COG1670J	hypothetical protein Spy49_1217c
Spy49_1218c	-	NC	1214086	1214481	131	-	6984452	-	hypothetical protein Spy49_1218c
Spy49_1219c	-	NC	1214499	1214753	84	-	6984453	-	hypothetical protein Spy49_1219c
Spy49_1220	aroA2	NE	1215231	1215983	250	+	6984455	COG2876E	3-deoxy-7-phosphoheptulonate synthase
Spy49_1221	aroB	NE	1216039	1217112	357	+	6984456	COG0337E	3-dehydroquinate synthase
Spy49_1222c	-	NE	1217548	1217853	101	-	6984457	COG1917S	acetate kinase
Spy49_1223c	-	NE	1217855	1218193	112	-	6984458	COG1917S	hypothetical protein Spy49_1223c
Spy49_1224c	-	NE	1218246	1219001	251	-	6984459	COG2226H	ubiquinone/menaquinone biosynthesis methyltransferase UbiE/COQ5
Spy49_1225c	aroE1	NE	1219236	1220114	292	-	6984460	COG0169E	Shikimate 5-dehydrogenase
Spy49_1226c	-	NE	1220252	1223668	1138	-	6984461	COG3250G	beta-galactosidase
Spy49_1227c	-	NE	1223688	1225172	494	-	6984462	COG4753T	two-component sensor response regulator
Spy49_1228c	-	NE	1225172	1226896	574	-	6984463	COG2972T	two-component sensor histidine kinase
Spy49_1229c	-	NE	1226886	1227491	201	-	6984464	COG5578S	hypothetical protein Spy49_1229c
Spy49_1230c	-	NE	1227797	1229242	481	-	6984465	COG1653G	ABC transporter substrate-binding protein
Spy49_1231c	-	NC	1229323	1230249	308	-	6984466	COG0395G	ABC transporter permease
Spy49_1233c	-	NE	1230259	1231209	316	-	6984467	COG4209G	sugar-binding transport protein
Spy49_1234	-	NE	1231405	1232100	231	+	6984468	COG1940KG	transcriptional regulator
Spy49_1235c	-	NE	1232893	1234335	480	-	6984469	COG2723G	beta-glucosidase
Spy49_1236c	hyl	NC	1234359	1236053	564	-	6984470	-	hyaluronidase
Spy49_1237c	-	NE	1236104	1237144	346	-	6984471	COG1609K	transcription regulator
Spy49_1238	-	NC	1237277	1238563	428	+	6984472	COG3538S	hypothetical protein Spy49_1238
Spy49_1239	-	NC	1238578	1241283	901	+	6984473	COG0383G	Alpha-mannosidase
Spy49_1240c	-	NE	1241384	1242739	451	-	6984474	COG2972T	histidine kinase
Spy49_1241c	-	NC	1243405	1244760	451	-	6984475	COG2265J	TrmA family RNA methyltransferase
Spy49_1242	recX	NE	1244875	1245651	258	+	6984476	COG2137R	recombination regulator RecX
Spy49_1243	-	NC	1245731	1246264	177	+	6984477	COG3557J	hypothetical protein Spy49_1243
Spy49_1244	-	NE	1246634	1246912	92	+	6984478	-	hypothetical protein Spy49_1244
Spy49_1245	-	C	1246927	1247034	35	+	6984479	-	hypothetical protein Spy49_1245
Spy49_1247c	-	NE	1254000	1254548	182	-	6984490	COG1544J	ribosomal subunit Interface protein
Spy49_1248c	comF	NE	1254628	1255293	221	-	6984491	COG1040R	late competence protein
Spy49_1249c	-	NE	1255265	1256590	441	-	6984492	COG4098L	late competence protein required for DNA uptake
Spy49_1250	-	NE	1256646	1257278	210	+	6984493	COG1739S	hypothetical protein Spy49_1250
Spy49_1251	cysM	NE	1257406	1258347	313	+	6984494	COG0031E	cysteine synthase A
Spy49_1252c	-	C	1258365	1258742	125	-	6984495	COG1098J	hypothetical protein Spy49_1252c

Spy49_1253c	-	NE	1258742	1260142	466	-	6984496	COG0652O	Hydrolase, haloacid dehalogenase family/peptidyl-prolyl cis-trans isomerase, cyclophilin type
Spy49_1254c	-	E	1260179	1260820	213	-	6984497	COG2197TK	two-component response regulator
Spy49_1255c	-	NE	1260813	1261817	334	-	6984498	COG4585T	two-component sensor histidine kinase
Spy49_1256c	-	NE	1261814	1262506	230	-	6984499	COG4758S	hypothetical protein Spy49_1256c
Spy49_1257c	-	E	1262629	1264527	632	-	6984500	COG2815S	Serine/threonine protein kinase
Spy49_1259c	pppL	E	1264524	1265264	246	-	6984501	COG0631T	phosphoprotein phosphatase
Spy49_1260c	sunL	NE	1265302	1266624	440	-	6984502	COG0144J	RNA-binding Sun protein
Spy49_1261c	fmt	E	1266614	1267549	311	-	6984503	COG0223J	methionyl-tRNA formyltransferase
Spy49_1262c	priA	C	1267611	1269995	794	-	6984504	COG1198L	primosome assembly protein PriA
Spy49_1263c	-	NC	1270060	1270377	105	-	6984505	COG1758K	DNA-directed RNA polymerase subunit omega
Spy49_1265c	gmk	E	1270393	1271028	211	-	6984506	COG0194F	guanylate kinase
Spy49_1266c	-	NE	1271138	1272745	535	-	6984507	COG1418R	hypothetical protein Spy49_1266c
Spy49_1267c	-	NE	1272875	1273771	298	-	6984508	COG0583K	transcriptional regulator
Spy49_1268	atoB	NC	1273972	1275159	395	+	6984509	COG0183I	acetyl-CoA acetyltransferase
Spy49_1269	atoD1	NE	1275183	1275833	216	+	6984510	COG1788I	acetyl-CoA:acetoacetyl-CoA transferase A subunit
Spy49_1270	atoA	NE	1275835	1276494	219	+	6984511	COG2057I	acetyl-CoA:acetoacetyl-CoA transferase b subunit
Spy49_1271	-	NE	1276527	1277306	259	+	6984512	COG1028IQR	3-hydroxybutyrate dehydrogenase
Spy49_1272	-	NE	1277377	1278708	443	+	6984513	COG2610GE	permease
Spy49_1273	luxS	NE	1278782	1279264	160	+	6984514	COG1854T	S-ribosylhomocysteinase
Spy49_1274c	-	NE	1279411	1280880	489	-	6984515	-	hypothetical protein Spy49_1274c
Spy49_1275c	-	NE	1280894	1282048	384	-	6984516	COG0116L	hypothetical protein Spy49_1275c
Spy49_1276c	-	NC	1282493	1282819	108	-	6984583	COG3599D	hypothetical protein Spy49_1276c
Spy49_1277c	-	NE	1282947	1283456	169	-	6984584	COG4474S	hypothetical protein Spy49_1277c
Spy49_1278	recU	NC	1283537	1284145	202	+	6984585	COG3331R	Holliday junction-specific endonuclease
Spy49_1279	pbp1A	E	1284132	1286297	721	+	6984586	COG0744M	multimodular transpeptidase-transglycosylase
Spy49_1280c	pepC	NE	1286764	1288101	445	-	6984587	COG3579E	aminopeptidase C
Spy49_1281c	nadE	E	1288286	1289134	282	-	6984588	COG0171H	NAD synthetase
Spy49_1282c	-	E	1289112	1290566	484	-	6984589	COG1488H	nicotinate phosphoribosyltransferase
Spy49_1283c	aapA	NE	1290737	1292116	459	-	6984590	COG1113E	amino acid permease
Spy49_1284c	nox2	NE	1292285	1293202	305	-	6984591	COG0492O	thioredoxin reductase
Spy49_1285c	-	NC	1293266	1293490	74	-	6984592	-	hypothetical protein Spy49_1285c
Spy49_1286c	-	NE	1293594	1294337	247	-	6984593	COG1126E	amino acid ABC transporter ATP-binding protein
Spy49_1287c	-	NE	1294337	1295140	267	-	6984594	COG0765E	amino acid ABC transporter
Spy49_1289c	-	E	1295335	1296678	447	-	6984595	COG0513LKJ	ATP-dependent RNA helicase
Spy49_1290c	mraY	E	1296836	1297846	336	-	6984596	COG0472M	phospho-N-acetylmuramoyl-pentapeptide-transferase
Spy49_1291c	ftsI	E	1297848	1300103	751	-	6984597	COG0768M	cell division protein FtsI
Spy49_1292c	ftsL	NC	1300107	1300430	107	-	6984598	COG4839D	cell division protein FtsL
Spy49_1293c	mraW	NE	1300435	1301385	316	-	6984599	COG0275M	S-adenosyl-methyltransferase MraW
Spy49_1294c	-	NE	1301736	1301942	68	-	6984600	-	hypothetical protein Spy49_1294c
Spy49_1295c	proA	NE	1301917	1303167	416	-	6984601	COG0014E	gamma-glutamyl phosphate reductase
Spy49_1296c	proB	NE	1303160	1303981	273	-	6984602	COG0263E	gamma-glutamyl kinase
Spy49_1297c	-	NE	1304045	1305673	542	-	6984603	-	hypothetical protein Spy49_1297c
Spy49_1298c	-	NE	1305678	1306412	244	-	6984604	COG1131V	ABC transporter
Spy49_1299c	-	NE	1306446	1306712	88	-	6984605	-	hypothetical protein Spy49_1299c
Spy49_1300c	tkt	C	1306904	1308889	661	-	6984606	COG0021G	transketolase
Spy49_1301c	tal	NE	1309107	1309751	214	-	6984607	COG0176G	translaldolase
Spy49_1303c	-	NE	1309877	1311376	499	-	6984608	-	transcriptional regulatory protein
Spy49_1304c	npx	NE	1311366	1312712	448	-	6984609	COG0446R	NADH peroxidase
Spy49_1305c	glpF1	NE	1312821	1313522	233	-	6984610	COG0580G	glycerol uptake facilitator protein
Spy49_1306c	glpO	NE	1313524	1315362	612	-	6984611	COG0578C	aerobic glycerol-3-phosphate dehydrogenase
Spy49_1307c	glpK	NE	1315378	1316904	508	-	6984612	COG0554C	glycerol kinase
Spy49_1308c	-	NE	1317264	1317656	130	-	6984613	COG3212S	hypothetical protein Spy49_1308c
Spy49_1309c	-	NE	1317784	1318041	85	-	6984614	COG4224S	hypothetical protein Spy49_1309c
Spy49_1310c	glyS	E	1318195	1320234	679	-	6984615	COG0751J	glycyl-tRNA synthetase subunit beta
Spy49_1311c	glyQ	E	1320606	1321523	305	-	6984616	COG0752J	glycyl-tRNA synthetase subunit alpha
Spy49_1312c	-	NE	1321896	1322402	168	-	6984617	COG1755S	hypothetical protein Spy49_1312c
Spy49_1313c	-	NE	1322561	1323400	279	-	6984618	COG0656R	2,5-diketo-D-gluconic acid reductase
Spy49_1314c	nagA	NE	1323522	1324670	382	-	6984619	COG1820G	N-acetylglucosamine-6-phosphate deacetylase
Spy49_1316c	-	NE	1324787	1326418	543	-	6984620	COG1283P	Sodium-dependent phosphate transporter
Spy49_1318c	-	NE	1327469	1328311	280	-	6984621	COG1307S	hypothetical protein Spy49_1318c
Spy49_1319	-	NE	1328604	1329161	185	+	6984622	COG1309K	TetR family transcriptional regulator
Spy49_1320c	-	NE	1329198	1330022	274	-	6984623	COG0561R	hypothetical protein Spy49_1320c
Spy49_1321c	-	NE	1330024	1330641	205	-	6984624	COG2860S	hypothetical protein Spy49_1321c
Spy49_1322c	lacD1	NE	1330838	1331815	325	-	6984625	COG3684G	tagatose 1,6-diphosphate aldolase
Spy49_1323c	lacC1	NE	1331897	1332160	87	-	6984626	COG1105G	Tagatose-6-phosphate kinase / 1- phosphofructokinase
Spy49_1324c	-	NC	1331965	1332315	116	-	6984627	COG1105G	Tagatose-6-phosphate kinase / 1- phosphofructokinase
Spy49_1325c	lacB	NE	1332325	1332840	171	-	6984628	COG0698G	galactose-6-phosphate isomerase subunit LacB
Spy49_1326c	lacA	NE	1332855	1333280	141	-	6984629	COG0698G	galactose-6-phosphate isomerase subunit LacA
Spy49_1327c	-	NE	1333538	1334989	483	-	6984630	COG3775G	PTS system enzyme IIC component
Spy49_1328c	-	NE	1335015	1335320	101	-	6984631	COG3414G	PTS system IIB component
Spy49_1329c	-	NE	1335313	1335786	157	-	6984632	COG1762GT	PTS system enzyme IIA component
Spy49_1330	lacR1	NE	1336023	1336793	256	+	6984633	COG1349KG	lactose phosphotransferase system repressor
Spy49_1331c	copZ	NE	1336997	1337200	67	-	6984634	COG2608P	copper chaperone-copper transport operon
Spy49_1332c	copA	NE	1337214	1339445	743	-	6984635	COG2217P	copper-transporting ATPase
Spy49_1334c	copY	NE	1339445	1339882	145	-	6984636	COG3682K	Negative transcriptional regulator, CopY
Spy49_1335	-	NE	1340045	1341046	333	+	6984637	COG0657I	esterase
Spy49_1336c	rbfA	NC	1341098	1341448	116	-	6984638	COG0858J	ribosome-binding factor A
Spy49_1337c	infB	E	1341654	1344515	953	-	6984639	COG0532J	translation initiation factor IF-2
Spy49_1338c	-	NE	1344535	1344837	100	-	6984640	COG1358J	hypothetical protein Spy49_1338c
Spy49_1339c	-	NC	1344830	1345126	98	-	6984641	COG2740K	Predicted nucleic-acid-binding protein
Spy49_1340c	nusA	E	1345142	1346299	385	-	6984642	COG0195K	transcription elongation factor NusA
Spy49_1341c	-	NE	1346474	1347010	178	-	6984643	COG0779S	hypothetical protein Spy49_1341c
Spy49_1342c	trmB	NE	1347269	1347904	211	-	6984645	COG0220R	tRNA (guanine-N(7)-)-methyltransferase
Spy49_1343c	-	E	1347904	1348695	263	-	6984646	COG0510M	hypothetical protein Spy49_1343c
Spy49_1344c	-	NE	1348759	1349793	344	-	6984647	COG4473U	ABC transporter
Spy49_1345c	-	NE	1349796	1350521	241	-	6984648	COG1131V	ABC transporter ATP-binding protein
Spy49_1346	hit	NE	1350593	1351012	139	+	6984649	COG0537FGR	cell-cycle regulation histidine triad (HIT) protein
Spy49_1347	-	NE	1351009	1351365	118	+	6984650	-	hypothetical protein Spy49_1347
Spy49_1348c	-	NE	1351476	1352759	427	-	6984651	COG1316K	transcription regulator
Spy49_1349c	-	NE	1352768	1353292	174	-	6984652	COG0456R	histone acetyltransferase
Spy49_1350c	-	NC	1353267	1353728	153	-	6984653	COG0802R	hypothetical protein Spy49_1350c
Spy49_1351c	-	NE	1353882	1355342	486	-	6984654	COG2252R	hypothetical protein Spy49_1351c
Spy49_1352	-	NE	1355607	1356002	131	+	6984655	COG0561R	hypothetical protein Spy49_1352
Spy49_1353	-	NE	1356019	1356420	133	+	6984656	COG0561R	hypothetical protein Spy49_1353, partial
Spy49_1354	manL	NE	1356772	1357764	330	+	6984657	COG3444G	mannose-specific phosphotransferase system component IIAB
Spy49_1355	manM	NE	1357853	1358662	269	+	6984658	COG3715G	PTS system mannose-specific transporter subunit IIC
Spy49_1356	manN	NE	1358679	1359590	303	+	6984659	COG3716G	mannose-specific phosphotransferase system component IID
Spy49_1357	-	NE	1359704	1360063	119	+	6984660	COG4687S	hypothetical protein Spy49_1357
Spy49_1358	serS	E	1360454	1361731	425	+	6984661	COG0172J	seryl-tRNA synthetase
Spy49_1359c	accA	NC	1361964	1362734	256	-	6984662	COG0825I	acetyl-CoA carboxylase subunit alpha
Spy49_1360c	accD	E	1362731	1363597	288	-	6984743	COG0777I	acetyl-CoA carboxylase subunit beta
Spy49_1361c	accC	E	1363606	1364970	454	-	6984744	COG0439I	acetyl-CoA carboxylase biotin carboxylase subunit
Spy49_1362c	fabZ	NC	1365002	1365424	140	-	6984745	COG0764I	(3R)-hydroxymyristoyl-ACP dehydratase
Spy49_1363c	accB	NC	1365421	1365921	166	-	6984746	COG0511I	acetyl-CoA carboxylase biotin carboxyl carrier protein subunit
Spy49_1364c	fabF	E	1365923	1367155	410	-	6984747	COG0304IQ	3-oxoacyl-ACP synthase II
Spy49_1365c	fabG	E	1367170	1367904	244	-	6984748	COG1028IQR	3-ketoacyl-ACP reductase
Spy49_1366c	fabD	E	1367894	1368832	312	-	6984749	COG0331I	Malonyl CoA-ACP transacylase

Spy49_1367c	fabK	E	1368857	1369828	323	-	6984750	COG2070R	Enoyl-ACP reductase (FMN)
Spy49_1368c	acpP	NC	1370029	1370253	74	-	6984751	COG0236IQ	acyl carrier protein
Spy49_1369c	fabH	E	1370314	1371288	324	-	6984752	COG0332I	3-oxoacyl-ACP synthase III
Spy49_1370c	-	NE	1371289	1371723	144	-	6984753	COG1846K	transcriptional regulator
Spy49_1371c	phaB	E	1371797	1372588	263	-	6984754	COG1024I	enoyl-CoA hydratase
Spy49_1372c	dnaJ	E	1372809	1373945	378	-	6984755	COG0484O	molecular chaperone DnaJ
Spy49_1373	-	NC	1374008	1374217	69	+	6984756	-	hypothetical protein Spy49_1373
Spy49_1374c	dnaK	E	1374226	1376052	608	-	6984757	COG0443O	molecular chaperone DnaK
Spy49_1375c	grpE	E	1376233	1376805	190	-	6984758	COG0576O	heat shock protein GrpE
Spy49_1376c	hrcA	NE	1376808	1377851	347	-	6984759	COG1420K	heat-inducible transcription repressor
Spy49_1377c	-	NE	1377978	1378547	189	-	6984760	COG1705NU	N-acetyl-muramidase
Spy49_1378c	-	NE	1378544	1379287	247	-	6984761	COG1876M	D-alanyl-D-alanine carboxypeptidase
Spy49_1379c	-	NE	1379277	1379984	235	-	6984762	COG0406G	phosphoglycerate mutase family
Spy49_1380c	-	NE	1380376	1380615	79	-	6984763	COG2261S	hypothetical protein Spy49_1380c
Spy49_1381c	-	NE	1380834	1381049	71	-	6984764	-	hypothetical protein Spy49_1381c
Spy49_1382c	gatB	E	1381046	1382485	479	-	6984765	COG0064J	aspartyl/glutamyl-tRNA amidotransferase subunit B
Spy49_1383c	gatA	E	1382485	1383951	488	-	6984766	COG0154J	aspartyl/glutamyl-tRNA amidotransferase subunit A
Spy49_1384c	gatC	NC	1383951	1384253	100	-	6984767	COG0721J	aspartyl/glutamyl-tRNA amidotransferase subunit C
Spy49_1385c	-	NE	1384485	1384733	82	-	6984768	COG0574G	pyruvate,phosphate dikinase
Spy49_1386c	-	NE	1385248	1385802	184	-	6984769	COG1335Q	Nicotinamidase
Spy49_1387c	codY	E	1385949	1386731	260	-	6984770	COG4465K	transcriptional repressor CodY
Spy49_1388c	-	NE	1386949	1388130	393	-	6984771	COG0436E	aminotransferase AlaT
Spy49_1389	-	NE	1388395	1388847	150	+	6984772	COG0589T	hypothetical protein Spy49_1389
Spy49_1390c	-	NE	1388970	1390358	462	-	6984773	COG4696S	hypothetical protein Spy49_1390c
Spy49_1391	asnB	NE	1390430	1391395	321	+	6984774	COG0252EJ	L-asparaginase
Spy49_1392c	-	NE	1391744	1392448	234	-	6984775	-	hypothetical protein Spy49_1392c
Spy49_1393c	-	NE	1392461	1393363	300	-	6984776	COG1131V	ABC transporter
Spy49_1394c	recG	C	1393655	1395670	671	-	6984777	COG1200LK	ATP-dependent DNA helicase RecG
Spy49_1395c	-	NE	1396045	1397499	484	-	6984778	COG1122P	cobalt ABC transporter ATPase
Spy49_1396c	-	NE	1397436	1398116	226	-	6984779	COG0619P	cobalt ABC transporter permease
Spy49_1397c	-	NE	1398113	1398706	197	-	6984780	-	ABC transporter permease
Spy49_1398c	cycC	NE	1398703	1400373	556	-	6984781	COG1132V	ABC transporter
Spy49_1400c	cycD	NE	1400366	1402129	587	-	6984782	COG4988CO	ABC transporter
Spy49_1401c	fhuC2	NE	1402126	1402962	278	-	6984783	COG1120PH	ferrichrome transport ATP-binding protein fhuC
Spy49_1402c	fhuB2	NE	1402959	1403981	340	-	6984784	COG0609P	ferrichrome ABC transporter permease
Spy49_1403c	fhuD2	NE	1403983	1405728	581	-	6984785	COG0614P	ferrichrome ABC transporter substrate-binding protein
Spy49_1405c	-	NE	1405925	1409752	1275	-	6984786	COG4886S	Fe3+-siderophore transport protein
Spy49_1407c	isp2	NE	1410242	1411753	503	-	6984787	COG3942R	Immunogenic secreted protein
Spy49_1408c	alr	E	1411840	1412940	366	-	6984788	COG0787M	alanine racemase
Spy49_1409c	acpS	E	1412937	1413293	118	-	6984789	COG0736I	4\'-phosphopantetheinyl transferase
Spy49_1410c	secA	E	1413409	1415928	839	-	6984790	COG0653U	preprotein translocase subunit SecA
Spy49_1412c	pmi	NE	1417389	1418342	317	-	6984791	COG1482G	mannose-6-phosphate isomerase
Spy49_1413c	scrK	NE	1418389	1419393	334	-	6984792	COG1940KG	Fructokinase
Spy49_1414c	endoS	NE	1419562	1422093	843	-	6984793	COG3469G	endo-beta-N-acetylglucosaminidase
Spy49_1415c	scrA	NE	1422324	1424207	627	-	6984794	COG1263G	sucrose-specific PTS permease, enzyme II
Spy49_1416	scrB	NC	1424449	1425888	479	+	6984795	COG1621G	Sucrose-6-phosphate hydrolase
Spy49_1417	scrR	NE	1425893	1426858	321	+	6984796	COG1609K	sucrose operon repressor
Spy49_1418c	nusB	NE	1426999	1427451	150	-	6984797	COG0781K	transcription antitermination protein NusB
Spy49_1419c	-	NE	1427444	1427833	129	-	6984798	COG1302S	hypothetical protein Spy49_1419c
Spy49_1420c	efp	NE	1427879	1428436	185	-	6984799	COG0231J	elongation factor P
Spy49_1421c	comEB	NE	1428532	1428993	153	-	6984800	COG2131F	late competence protein required for DNA binding
Spy49_1422c	pepP	NE	1429028	1430101	357	-	6984801	COG0006E	aminopeptidase YpdF
Spy49_1423c	uvrA	NE	1430216	1433074	952	-	6984802	COG0178L	excinuclease ABC subunit A
Spy49_1424	-	C	1433247	1434191	314	+	6984803	COG0598P	divalent cation transport protein, magnesium transporter CorA family
Spy49_1425	-	NE	1434323	1434979	218	+	6984804	COG4858S	hypothetical protein Spy49_1425
Spy49_1426c	rpsR	NC	1435112	1435351	79	-	6984805	COG0238J	30S ribosomal protein S18
Spy49_1427c	ssb2	C	1435516	1436007	163	-	6984806	COG0629L	single-stranded DNA-binding protein
Spy49_1428c	rpsF	NC	1436029	1436319	96	-	6984807	COG0360J	30S ribosomal protein S6
Spy49_1429c	-	NE	1436492	1436785	97	-	6984808	-	hypothetical protein Spy49_1429c
Spy49_1430	mutY	NE	1436953	1438107	384	+	6984809	COG1194L	A/G-specific adenine glycosylase
Spy49_1431	-	NE	1438284	1438871	195	+	6984810	COG1476K	Cro/CI family transcriptional regulator
Spy49_1432c	trx2	NE	1438923	1439237	104	-	6984811	COG3118O	thioredoxin reductase
Spy49_1433c	-	NE	1439318	1439713	131	-	6984812	-	PAP2 family protein, partial
Spy49_1434c	mutS2	NE	1439822	1442161	779	-	6984813	COG1193L	DNA mismatch repair protein
Spy49_1435c	-	NE	1442310	1442855	181	-	6984814	COG1286R	hypothetical protein Spy49_1435c
Spy49_1436c	-	NE	1442858	1443166	102	-	6984815	-	hypothetical protein Spy49_1436c
Spy49_1437	-	E	1443323	1444225	300	+	6984816	COG1039L	ribonuclease HIII
Spy49_1438	spi	E	1444236	1444829	197	+	6984817	COG0681U	signal peptidase I
Spy49_1439	recD	NE	1444887	1447340	817	+	6984818	COG0507L	Exodeoxyribonuclease V subunit alpha
Spy49_1440	-	NE	1447431	1447913	160	+	6984819	-	hypothetical protein Spy49_1440
Spy49_1441c	dinP	NC	1448006	1449100	364	-	6984820	COG0389L	DNA polymerase IV
Spy49_1442	pfl	NC	1449309	1451636	775	+	6984821	COG1882C	pyruvate formate-lyase
Spy49_1443c	-	NE	1451724	1452113	129	-	6984822	COG1680V	esterase
Spy49_1444c	cppA	NE	1452098	1452850	250	-	6984903	-	C3-degrading proteinase
Spy49_1445	-	NE	1453112	1454044	310	+	6984904	-	hypothetical protein Spy49_1445
Spy49_1447c	glpF2	NC	1454380	1455327	315	-	6984905	COG0580G	glycerol uptake facilitator protein
Spy49_1449c	norA	NE	1455730	1456926	398	-	6984906	COG2814G	antibiotic resistance protein NorA
Spy49_1450	-	NE	1457032	1457751	239	+	6984907	COG0664T	Crp/Fnr family transcriptional regulator
Spy49_1451	pepXP	NE	1457773	1460055	760	+	6984908	COG2936R	x-prolyl-dipeptidyl aminopeptidase
Spy49_1452c	-	NE	1460135	1460356	73	-	6984909	-	hypothetical protein Spy49_1452c
Spy49_1453	-	NE	1460526	1460900	124	+	6984910	COG1476K	repressor-phage associated
Spy49_1454c	-	NE	1461245	1461424	59	-	6984911	-	Paratox
Spy49_1455	spd3	NE	1461662	1462462	266	+	6984912	-	streptodornase
Spy49_1456	-	NE	1462842	1463168	108	+	6984913	-	hypothetical protein Spy49_1456
Spy49_1457c	-	NE	1463218	1464084	288	-	6984914	-	putative phage associated protein
Spy49_1458c	-	NE	1464072	1464596	174	-	6984915	COG3600S	phage-associated protein
Spy49_1459c	-	NE	1464736	1465944	402	-	6984916	COG1705NU	phage-associated cell wall hydrolase
Spy49_1460c	-	NE	1466060	1466287	75	-	6984917	COG5546S	Holin
Spy49_1461c	-	NE	1466284	1466559	91	-	6984918	-	hypothetical protein Spy49_1461c
Spy49_1462c	-	NE	1466569	1467186	205	-	6984919	-	hypothetical protein Spy49_1462c
Spy49_1463c	-	NC	1467189	1467350	53	-	6984920	-	hypothetical protein Spy49_1463c
Spy49_1464c	-	NE	1467364	1469274	636	-	6984921	-	hypothetical protein Spy49_1464c
Spy49_1465c	-	NC	1469290	1470294	334	-	6984922	-	Hyaluronidase-phage associated
Spy49_1466c	-	NC	1470291	1472342	683	-	6984923	-	hypothetical protein Spy49_1466c
Spy49_1467c	-	NC	1472339	1473118	259	-	6984924	COG4722S	phage associated hypothetical protein
Spy49_1468c	-	NE	1473151	1476786	1211	-	6984925	COG5412S	minor tail protein
Spy49_1470c	-	NE	1476801	1477130	109	-	6984926	-	hypothetical protein Spy49_1470c
Spy49_1471c	-	NE	1477172	1477531	119	-	6984927	-	hypothetical protein Spy49_1471c
Spy49_1472c	-	NE	1477584	1478237	217	-	6984928	-	Major tail protein
Spy49_1474c	-	NE	1478247	1478636	129	-	6984929	-	hypothetical protein Spy49_1474c
Spy49_1475c	-	NE	1478633	1478989	118	-	6984930	-	hypothetical protein Spy49_1475c
Spy49_1476c	-	NE	1478979	1479287	102	-	6984931	-	hypothetical protein-phage 370.2
Spy49_1477c	-	NE	1479284	1479637	117	-	6984932	-	hypothetical protein Spy49_1477c
Spy49_1478c	-	NE	1479651	1479893	80	-	6984933	-	hypothetical protein Spy49_1478c
Spy49_1479c	-	NE	1479903	1480985	360	-	6984934	-	phage protein
Spy49_1480c	-	NE	1480988	1481368	126	-	6984935	-	structural protein-phage associated
Spy49_1481c	-	NE	1481378	1481911	177	-	6984936	-	hypothetical protein Spy49_1481c

Spy49_1482c	-	NE	1482055	1482321	88	-	6984937	-	putative phage associated protein
Spy49_1483c	-	NE	1482324	1482638	104	-	6984938	-	hypothetical protein Spy49_1483c
Spy49_1484c	-	NE	1482708	1482893	61	-	6984939	-	hypothetical protein Spy49_1484c
Spy49_1485c	-	NE	1482897	1484459	520	-	6984940	COG5585T	hypothetical protein Spy49_1485c
Spy49_1486c	-	NE	1484440	1485942	500	-	6984941	-	hypothetical protein Spy49_1486c
Spy49_1487c	-	NE	1485954	1487243	429	-	6984942	COG1783R	Large terminase
Spy49_1488c	-	NE	1487221	1487703	160	-	6984943	COG3728L	Small terminase
Spy49_1489c	-	NE	1488535	1488975	146	-	6984944	-	hypothetical protein Spy49_1489c
Spy49_1491c	-	NE	1489415	1489936	173	-	6984945	-	phage protein
Spy49_1492c	-	NE	1489933	1490226	97	-	6984946	-	hypothetical protein Spy49_1492c
Spy49_1493c	-	NE	1490223	1490408	61	-	6984947	-	putative phage associated protein
Spy49_1494c	-	NC	1490506	1490991	161	-	6984948	COG2226H	DNA N-4 cytosine methyltransferase M.NgoMXV
Spy49_1496c	-	NE	1491276	1491680	134	-	6984949	-	phage protein
Spy49_1497c	-	NC	1491664	1491954	96	-	6984950	-	hypothetical protein Spy49_1497c
Spy49_1498c	-	NE	1492193	1492549	118	-	6984951	-	putative phage associated protein
Spy49_1499c	-	NE	1492625	1492987	120	-	6984952	COG4570L	hypothetical protein Spy49_1499c
Spy49_1500c	-	NC	1493196	1493621	141	-	6984953	COG0629L	Single-strand binding protein 3
Spy49_1501c	-	NE	1493614	1494288	224	-	6984954	-	Recombination protein
Spy49_1502c	-	NC	1494289	1494435	48	-	6984955	-	hypothetical protein Spy49_1502c
Spy49_1504c	-	NE	1494793	1495047	84	-	6984956	-	hypothetical protein Spy49_1504c
Spy49_1505c	-	NC	1495034	1495381	115	-	6984957	-	hypothetical protein Spy49_1505c
Spy49_1506c	-	NE	1495522	1497093	523	-	6984958	COG1484L	DNA replication protein DnaC
Spy49_1507c	-	NE	1497209	1497652	147	-	6984959	-	hypothetical protein Spy49_1507c
Spy49_1509c	-	NE	1498078	1498350	90	-	6984960	-	hypothetical protein Spy49_1509c
Spy49_1510c	-	NC	1498523	1498702	59	-	6984961	-	hypothetical protein Spy49_1510c
Spy49_1511c	-	NC	1498833	1498967	44	-	6984962	-	putative phage associated protein
Spy49_1512c	-	NE	1499249	1499608	119	-	6984963	-	hypothetical protein Spy49_1512c
Spy49_1513c	-	NE	1499682	1499939	85	-	6984964	-	hypothetical protein Spy49_1513c
Spy49_1514c	-	NE	1500142	1500483	113	-	6984965	-	hypothetical protein Spy49_1514c
Spy49_1516c	-	NC	1500755	1500904	49	-	6984966	-	putative phage associated protein
Spy49_1517c	-	NE	1500936	1501664	242	-	6984967	COG3645S	P1-type antirepressor-phage associated
Spy49_1518c	-	NE	1501697	1501885	62	-	6984968	-	cro protein
Spy49_1520c	-	NC	1502073	1502255	60	-	6984969	-	hypothetical protein Spy49_1520c
Spy49_1521c	-	NE	1502532	1503293	253	-	6984970	COG3617K	Antirepressor
Spy49_1522	-	C	1503460	1503732	90	+	6984971	-	hypothetical protein Spy49_1522
Spy49_1525	-	NE	1504006	1504473	155	+	6984972	-	hypothetical protein Spy49_1525
Spy49_1526	-	NE	1504589	1505368	259	+	6984973	-	hypothetical protein Spy49_1526
Spy49_1528c	-	NC	1505502	1505660	52	-	6984974	-	hypothetical protein Spy49_1528c
Spy49_1529	-	NC	1506024	1506782	252	+	6984975	COG2932K	Repressor protein
Spy49_1530	-	NE	1506855	1507277	140	+	6984976	-	Lj965 prophage superinfection immunity protein
Spy49_1531	-	NE	1507398	1508534	378	+	6984977	COG4974L	Prophage NZ131.3 integrase
Spy49_1532	-	NE	1508573	1508893	106	+	6984978	COG0718S	hypothetical protein Spy49_1532
Spy49_1533c	-	NE	1508950	1509687	245	-	6984979	COG0789K	MerR family transcriptional regulator
Spy49_1534c	dnaQ	NE	1509840	1510427	195	-	6984980	COG0847L	DNA polymerase III subunit epsilon
Spy49_1535c	-	NC	1510476	1511057	193	-	6984981	-	hypothetical protein Spy49_1535c
Spy49_1536c	-	NE	1511183	1511698	171	-	6984982	-	transposase-IS1548
Spy49_1537c	-	NE	1511601	1512305	234	-	6985063	COG5433L	transposase-IS1548
Spy49_1538	-	NE	1512526	1513695	389	+	6985064	COG2081R	hypothetical protein Spy49_1538
Spy49_1540c	deoC	NE	1513790	1514461	223	-	6985065	COG0274F	deoxyribose-phosphate aldolase
Spy49_1541c	nupC	NE	1514491	1515693	400	-	6985066	COG1972F	nucleoside permease nupC
Spy49_1542c	udp	NE	1515714	1516493	259	-	6985067	COG2820F	Uridine phosphorylase
Spy49_1543	-	NE	1516733	1517491	252	+	6985068	COG2188K	GntR family transcriptional regulator
Spy49_1544	rpsN	NE	1517700	1517969	89	+	6985069	COG0199J	30S ribosomal protein S14
Spy49_1545c	gcp	NC	1518141	1519169	342	-	6985070	COG0533O	DNA-binding/iron metalloprotein/AP endonuclease
Spy49_1546c	-	NE	1519159	1519614	151	-	6985071	COG0456R	ribosomal-protein-S18p-alanine acetyltransferase
Spy49_1547c	-	E	1519586	1520284	232	-	6985072	COG1214O	molecular chaperone
Spy49_1548	-	NC	1520568	1520798	76	+	6985073	COG5503S	hypothetical protein Spy49_1548
Spy49_1549	-	E	1520800	1522482	560	+	6985074	COG0595R	hypothetical protein Spy49_1549
Spy49_1550c	glnA	NE	1522710	1524056	448	-	6985075	COG0174E	glutamine synthetase
Spy49_1551c	glnR	NE	1524094	1524465	123	-	6985076	COG0789K	transcriptional regulator
Spy49_1552c	-	NE	1524532	1525083	183	-	6985077	COG4129S	hypothetical protein Spy49_1552c
Spy49_1553c	pgk	NC	1525345	1526541	398	-	6985078	COG0126G	phosphoglycerate kinase
Spy49_1554c	lppC	NE	1526726	1527580	284	-	6985079	COG2503R	acid phosphatase
Spy49_1555c	-	NE	1527820	1528701	293	-	6985080	COG0330O	hypersensitive- induced response protein-like protein
Spy49_1556c	-	E	1528938	1530602	554	-	6985081	COG1461R	DAK2 domain-containing protein
Spy49_1557c	asp	NC	1530602	1530967	121	-	6985082	COG1302S	alkaline-shock protein
Spy49_1558c	rpmB	NE	1531119	1531307	62	-	6985083	COG0227J	50S ribosomal protein L28
Spy49_1559c	fba	E	1531689	1532570	293	-	6985084	COG0191G	fructose-bisphosphate aldolase
Spy49_1560c	-	NE	1532916	1533842	308	-	6985086	COG1073R	hypothetical protein Spy49_1560c
Spy49_1561c	pyrG	NE	1534010	1535614	534	-	6985087	COG0504F	CTP synthetase
Spy49_1562c	rpoE	NE	1535871	1536479	202	-	6985088	COG3343K	DNA-directed RNA polymerase subunit delta
Spy49_1563c	tig	NE	1536663	1537946	427	-	6985089	COG0544O	trigger factor
Spy49_1564	-	NE	1538267	1539112	281	+	6985090	COG0668M	mechanosensitive ion channel
Spy49_1565c	-	NE	1539177	1539737	186	-	6985091	COG4475S	hypothetical protein Spy49_1565c
Spy49_1566c	-	NE	1539751	1540221	156	-	6985092	COG4720S	hypothetical protein Spy49_1566c
Spy49_1567c	thiD	NE	1540211	1540975	254	-	6985093	COG0351H	phosphomethylpyrimidine kinase
Spy49_1568c	truA	NE	1540965	1541714	249	-	6985094	COG0101J	tRNA pseudouridine synthase A
Spy49_1569c	comX2	NE	1542131	1542388	85	-	6985095	-	ComX2 alternate sigma factor
Spy49_1571c	-	NE	1548174	1549316	380	-	6985102	COG1929G	glycerate kinase
Spy49_1572	hsdR	NE	1549597	1552575	992	+	6985103	COG0610V	type I site-specific deoxyribonuclease
Spy49_1573	hsdS	NE	1552588	1553730	380	+	6985104	COG0732V	specificity determinant HsdS
Spy49_1574	hsdM	NE	1553743	1555323	526	+	6985105	COG0286V	type I site-specific deoxyribonuclease
Spy49_1575c	-	NE	1555533	1555733	66	-	6985106	-	hypothetical protein Spy49_1575c
Spy49_1576c	salR	NE	1555876	1556481	201	-	6985107	COG2197TK	response regulator of salavaricin regulon
Spy49_1577c	salK	NE	1556462	1557883	473	-	6985108	COG4585T	SalK
Spy49_1578c	salY	NE	1558064	1559971	635	-	6985109	-	ABC transporter
Spy49_1579c	salX	NE	1559973	1560710	245	-	6985110	COG1136V	salivaricin A ABC transporter
Spy49_1580c	salT	NE	1560707	1561168	153	-	6985111	COG2274V	salivaricin A modification enzyme
Spy49_1582c	salB	NE	1561242	1562867	541	-	6985112	COG4403V	salivaricin A modification enzyme, amino acid dehydration
Spy49_1583c	salA	NC	1562951	1563097	48	-	6985113	-	Lantibiotic salivaricin A
Spy49_1584c	lacG	NE	1563609	1565042	477	-	6985114	COG2723G	6-phospho-beta-galactosidase
Spy49_1585c	lacE	NE	1565103	1566800	565	-	6985115	COG1455G	PTS system lactose-specific transporter subunit IIBC
Spy49_1586c	lacF	NE	1566800	1567117	105	-	6985116	COG1447G	PTS system lactose-specific transporter subunit IIA
Spy49_1587c	lacD2	NE	1567141	1568124	327	-	6985117	COG3684G	tagatose 1,6-diphosphate aldolase
Spy49_1588c	lacC2	NE	1568128	1569057	309	-	6985118	COG1105G	tagatose-6-phosphate kinase
Spy49_1589c	lacB2	NE	1569103	1569618	171	-	6985119	COG0698G	galactose-6-phosphate isomerase subunit LacB
Spy49_1590c	lacA2	NE	1569653	1570081	142	-	6985120	COG0698G	galactose-6-phosphate isomerase subunit LacA
Spy49_1591	lacR2	NE	1570527	1571300	257	+	6985121	COG1349KG	lactose phosphotransferase system repressor
Spy49_1592	-	NE	1572028	1572249	73	+	6985122	-	DNA-damage-inducible protein J
Spy49_1593	-	NE	1572239	1572574	111	+	6985123	-	hypothetical protein Spy49_1593
Spy49_1594	-	NE	1573340	1573660	106	+	6985124	COG4974L	degenerate integrase
Spy49_1595c	rpsI	E	1573828	1574220	130	-	6985125	COG0103J	30S ribosomal protein S9
Spy49_1596c	rplM	NC	1574241	1574687	148	-	6985126	COG0102J	50S ribosomal protein L13
Spy49_1597c	-	NC	1574905	1575111	68	-	6985127	COG1476K	transcription regulator
Spy49_1598c	-	NE	1575108	1575806	232	-	6985128	-	hypothetical protein Spy49_1598c
Spy49_1599c	-	NC	1575942	1576817	291	-	6985129	COG1307S	hypothetical protein Spy49_1599c
Spy49_1600c	-	NE	1576898	1577380	160	-	6985130	COG3688R	hypothetical protein Spy49_1600c

Spy49_1601c	-	NE	1577419	1578165	248	-	6985131	COG0566J	23S rRNA methyltransferase
Spy49_1602c	-	NE	1578212	1579009	265	-	6985132	-	hypothetical protein Spy49_1602c
Spy49_1603c	-	NE	1579205	1579618	137	-	6985133	COG1939S	hypothetical protein Spy49_1603c
Spy49_1604c	cysS	E	1579611	1580954	447	-	6985134	COG0215J	cysteinyl-tRNA synthetase
Spy49_1605c	-	NE	1580982	1581212	76	-	6985135	-	hypothetical protein Spy49_1605c
Spy49_1606c	cysE	NC	1581691	1582272	193	-	6985136	COG1045E	Serine acetyltransferase cysE
Spy49_1607c	-	NE	1582281	1583033	250	-	6985137	-	hypothetical protein Spy49_1607c
Spy49_1608c	pnp	NE	1583026	1585158	710	-	6985138	COG1185J	polynucleotide phosphorylase/polyadenylase
Spy49_1609c	-	NE	1585438	1586166	242	-	6985139	COG0176G	translaldolase
Spy49_1610c	ulaA	NE	1586179	1587558	459	-	6985140	COG3037S	PTS system ascorbate-specific transporter subunit IIC
Spy49_1611c	-	NE	1587585	1587869	94	-	6985141	COG3414G	PTS system IIB component
Spy49_1612c	-	NE	1587862	1589922	686	-	6985142	COG3711K	transcriptional regulator
Spy49_1613c	rpsO	NE	1590275	1590544	89	-	6985223	COG0184J	30S ribosomal protein S15
Spy49_1614c	-	NE	1590720	1590920	66	-	6985224	-	hypothetical protein Spy49_1614c
Spy49_1615	def	C	1591319	1591933	204	+	6985225	COG0242J	peptide deformylase
Spy49_1616c	-	NE	1591967	1592509	180	-	6985226	COG0431R	hypothetical protein Spy49_1616c
Spy49_1617c	-	NE	1592640	1593068	142	-	6985227	COG1846K	MarR family transcriptional regulator
Spy49_1618c	polC	E	1593178	1597575	1465	-	6985228	COG2176L	DNA polymerase III PolC
Spy49_1619c	proS	E	1597830	1599686	618	-	6985229	COG0442J	prolyl-tRNA synthetase
Spy49_1620c	eep	NE	1599884	1601143	419	-	6985230	COG0750M	hypothetical protein Spy49_1620c
Spy49_1621c	cdsA	E	1601216	1602010	264	-	6985231	COG0575I	phosphatidate cytidylyltransferase
Spy49_1622c	uppS	E	1602023	1602772	249	-	6985232	COG0020I	undecaprenyl pyrophosphate synthase
Spy49_1624c	yajC	NE	1602991	1603356	121	-	6985233	COG1862U	preprotein translocase subunit YajC
Spy49_1625c	-	NE	1603472	1603819	115	-	6985234	COG0526OC	bacterocin transport accessory protein, Bta
Spy49_1626c	pulA	NE	1603961	1607365	1134	-	6985235	COG1523G	pullulanase
Spy49_1627c	dexB	NE	1607656	1609269	537	-	6985236	COG0366G	glucan 1,6-alpha-glucosidase
Spy49_1628c	msmK	NE	1609398	1610531	377	-	6985237	COG3839G	sugar ABC transporter ATP-binding protein
Spy49_1629c	lrp	NE	1610829	1611722	297	-	6985238	COG2508TQ	hypothetical protein Spy49_1629c
Spy49_1630	ska	NE	1612017	1613339	440	+	6985239	-	streptokinase
Spy49_1631c	dtd	NE	1613437	1613880	147	-	6985240	COG1490J	D-tyrosyl-tRNA(Tyr) deacylase
Spy49_1632c	relA	C	1613895	1616114	739	-	6985241	COG0317TK	GTP pyrophosphokinase / Guanosine- 3,5-bis(Diphosphate) 3-pyrophosphohydrolase
Spy49_1633c	sclA	NE	1616358	1617626	422	-	6985242	-	Collagen-like surface protein
Spy49_1634	nrdI2	NE	1618008	1618490	160	+	6985243	COG1780F	flavoprotein NrdI
Spy49_1635c	-	NE	1618883	1619701	272	-	6985244	COG3568R	hypothetical protein Spy49_1635c
Spy49_1636c	-	NE	1619784	1621970	728	-	6985245	COG1263G	PTS system glucose-specific IIBC component
Spy49_1637c	-	NE	1622326	1623075	249	-	6985246	COG1385S	16S ribosomal RNA methyltransferase RsmE
Spy49_1638c	prmA	NE	1623075	1624028	317	-	6985247	COG2264J	ribosomal protein L11 methyltransferase
Spy49_1639c	nudC	NE	1624045	1625127	360	-	6985248	-	hypothetical protein Spy49_1639c
Spy49_1640c	nudB	NE	1625304	1625660	118	-	6985249	COG1051F	MutT/nudix family protein
Spy49_1641c	nudA	NE	1625688	1626005	105	-	6985250	COG4043S	hypothetical protein Spy49_1641c
Spy49_1642c	-	NE	1626019	1626489	156	-	6985251	-	hypothetical protein Spy49_1642c
Spy49_1643c	-	NC	1626554	1626673	39	-	6985252	-	hypothetical protein Spy49_1643c
Spy49_1644c	-	NE	1626954	1627532	192	-	6985253	COG2801L	transposase
Spy49_1646c	-	NE	1627693	1627983	96	-	6985254	-	transposase
Spy49_1647c	-	NE	1628038	1628844	268	-	6985255	COG2801L	transposase, IS861
Spy49_1649c	-	NE	1629499	1629813	104	-	6985256	-	hypothetical protein Spy49_1649c
Spy49_1651	-	NE	1630202	1630438	78	+	6985257	-	hypothetical protein Spy49_1651
Spy49_1652	-	NC	1630510	1630704	64	+	6985258	-	hypothetical protein Spy49_1652
Spy49_1653	papB	NE	1630830	1632587	585	+	6985259	COG0147EH	Para-aminobenzoate synthase, aminase component / aminodeoxychorismate lyase
Spy49_1654	trpG	NE	1632620	1633186	188	+	6985260	COG0512EH	anthranilate synthase component II
Spy49_1655	-	NE	1633219	1634487	422	+	6985261	COG2256L	recombination factor protein RarA
Spy49_1656	pai1	NE	1634985	1635425	146	+	6985263	COG3153R	Pai1 protein
Spy49_1657	-	NE	1635460	1635966	168	+	6985264	COG0563F	topology modulation protein
Spy49_1658c	-	NE	1636090	1636425	111	-	6985265	-	hypothetical protein Spy49_1658c
Spy49_1659c	-	NE	1636435	1637079	214	-	6985266	-	hypothetical protein Spy49_1659c
Spy49_1660	dppA	NE	1638159	1639649	496	+	6985267	COG0747E	peptide ABC transporter substrate-binding protein
Spy49_1661	dppB	NE	1639762	1640739	325	+	6985268	COG0601EP	peptide ABC transporter permease
Spy49_1662	dppC	NE	1640736	1641557	273	+	6985269	COG1173EP	peptide ABC transporter permease
Spy49_1663	dppD	NE	1641569	1642372	267	+	6985270	COG0444EP	peptide ABC transporter ATP-binding protein
Spy49_1664	dppF	NE	1642356	1642982	208	+	6985271	COG1124EP	peptide ABC transporter ATP-binding protein dppF
Spy49_1665c	-	NE	1643064	1643264	66	-	6985272	-	hypothetical protein Spy49_1665c
Spy49_1666c	-	NE	1643433	1645904	823	-	6985273	-	hypothetical protein Spy49_1666c
Spy49_1667c	lmb	NE	1645917	1646837	306	-	6985274	COG0803P	laminin-binding surface protein
Spy49_1668c	-	NE	1647073	1648410	445	-	6985275	-	cell surface/fibronectin-binding protein
Spy49_1669c	scpA	NE	1648661	1652050	1129	-	6985276	COG1404O	C5a peptidase
Spy49_1670c	ennX	NE	1652385	1653491	368	-	6985277	COG1196D	EnnX protein
Spy49_1671c	emm49	NE	1653701	1654858	385	-	6985278	-	Antiphagocytic M protein
Spy49_1672c	-	NE	1655083	1656330	415	-	6985279	COG1196D	Fc-gamma receptor
Spy49_1673c	mga	NE	1656520	1658121	533	-	6985280	-	M protein trans-acting positive regulator (Mga)
Spy49_1674c	-	NE	1658797	1659048	83	-	6985281	-	hypothetical protein Spy49_1674c
Spy49_1675c	isp1	NE	1659126	1660754	542	-	6985282	COG3942R	Immunogenic secreted protein
Spy49_1676c	-	NE	1660856	1662244	462	-	6985283	COG0642T	two-component system histidine kinase
Spy49_1677c	-	NE	1662241	1662894	217	-	6985284	COG0745TK	two-component system response regulator
Spy49_1678c	-	NE	1662988	1664205	405	-	6985285	COG0577V	ABC transporter
Spy49_1679c	-	NE	1664218	1664892	224	-	6985286	COG1136V	ABC transporter ATP-binding protein
Spy49_1680c	-	NE	1664879	1666147	422	-	6985287	COG0845M	ATP-binding cassette transporter protein
Spy49_1681c	-	NE	1666571	1666975	134	-	6985288	-	hypothetical protein Spy49_1681c
Spy49_1682c	-	NE	1667002	1667298	98	-	6985289	-	hypothetical protein Spy49_1682c
Spy49_1683c	sfbX49	NE	1667440	1669395	651	-	6985290	-	fibronectin-binding protein
Spy49_1684c	sof	NE	1669575	1672655	1026	-	6985291	-	Serum opacity factor
Spy49_1686	-	NE	1673078	1673314	78	+	6985292	-	hypothetical protein Spy49_1686
Spy49_1687c	ropA	NE	1673590	1674540	316	-	6985293	-	foldase PrsA
Spy49_1688c	-	NE	1674572	1674889	105	-	6985294	-	hypothetical protein Spy49_1688c
Spy49_1689c	-	C	1674997	1675302	101	-	6985295	-	Spi SpeB protease inhibitor
Spy49_1690c	speB	NE	1675304	1676500	398	-	6985296	-	strepotococcal cysteine protease (streptopain) / streptococcal pyrogenic exotoxin B (SpeB)
Spy49_1691	ropB	NE	1677441	1678283	280	+	6985297	-	RopB
Spy49_1692c	mf1	NE	1678512	1679342	276	-	6985298	-	streptodornase B / Mitogenic factor 1
Spy49_1693	-	NC	1679426	1679554	42	+	6985299	-	hypothetical protein Spy49_1693
Spy49_1694	-	NE	1679707	1680216	169	+	6985300	COG1267I	low temperature requirement C protein
Spy49_1695c	gldA	NE	1680285	1681373	362	-	6985301	COG0371C	glycerol dehydrogenase
Spy49_1696c	mipB	NE	1681430	1682098	222	-	6985302	COG0176G	fructose-6-phosphate aldolase
Spy49_1697c	pflD	NE	1682111	1684528	805	-	6985383	COG1882C	pyruvate formate lyase 3
Spy49_1698c	-	NE	1684739	1686043	434	-	6985384	COG1455G	PTS system cellobiose-specific IIC component
Spy49_1699c	-	NE	1686053	1686361	102	-	6985385	COG1440G	PTS system cellobiose-specific IIB component
Spy49_1700c	-	NE	1686389	1686709	106	-	6985386	COG1447G	PTS system enzyme III
Spy49_1701c	-	NE	1686996	1687976	326	-	6985387	COG2390K	transcriptional regulator
Spy49_1702c	-	NE	1687992	1688741	249	-	6985388	COG1349KG	DeoR family transcriptional regulator
Spy49_1703	-	NE	1688864	1689637	257	+	6985389	COG1180O	pyruvate formate-lyase activating enzyme
Spy49_1704c	secE	NC	1689873	1690049	58	-	6985390	COG0690U	preprotein translocase subunit SecE
Spy49_1705c	rpmG	NC	1690063	1690215	50	-	6985391	COG0267J	50S ribosomal protein L33
Spy49_1706c	pbp2A	NC	1690264	1692600	778	-	6985392	COG0744M	multimodular transpeptidase-transglycosylase
Spy49_1707c	-	NE	1692639	1693019	126	-	6985393	COG0251J	translation initiation inhibitor
Spy49_1708	-	NE	1693510	1693701	63	+	6985394	-	hypothetical protein Spy49_1708
Spy49_1709	-	NE	1693595	1694596	333	+	6985395	COG0564J	ribosomal large subunit pseudouridine synthase
Spy49_1710c	-	NE	1694685	1696409	574	-	6985396	COG4640S	hypothetical protein Spy49_1710c
Spy49_1713	-	NE	1696572	1698071	499	+	6985397	COG4690E	dipeptidase
Spy49_1714c	-	NE	1698783	1699130	115	-	6985398	COG1395K	transcriptional regulator

Spy49_1715c	groEL	E	1699290	1700921	543	-	6985399	COG0459O	molecular chaperone GroEL
Spy49_1716c	groES	NC	1700957	1701247	96	-	6985400	COG0234O	molecular chaperone GroES
Spy49_1717c	clpC	NE	1701425	1703869	814	-	6985401	COG0542O	endopeptidase Clp ATP-binding protein C
Spy49_1718c	ctsR	NE	1703869	1704330	153	-	6985402	COG4463K	transcriptional regulator
Spy49_1719c	csp	NE	1704526	1704735	69	-	6985403	COG1278K	Major cold-shock protein
Spy49_1720c	-	NE	1705476	1706609	377	-	6985405	COG5433L	transposase
Spy49_1721	ahpC	NE	1707061	1707621	186	+	6985406	COG0450O	alkyl hydroperoxide reductase C
Spy49_1722	nox3	NE	1707642	1709174	510	+	6985407	COG3634O	alkyl hydroperoxide reductase F
Spy49_1723c	hutI	NE	1709232	1710497	421	-	6985408	COG1228Q	imidazolonepropionase
Spy49_1724	hutU	NE	1710789	1712819	676	+	6985409	COG2987E	urocanate hydratase
Spy49_1725	-	NE	1712907	1713806	299	+	6985410	COG3643E	glutamate formiminotransferase
Spy49_1726	-	NE	1713817	1714443	208	+	6985411	COG3404E	formiminotetrahydrofolate cyclodeaminase
Spy49_1727	fhs2	NE	1714461	1716134	557	+	6985412	COG2759F	formate--tetrahydrofolate ligase
Spy49_1728	-	NE	1716155	1716751	198	+	6985413	COG3758S	hypothetical protein Spy49_1728
Spy49_1729	-	NE	1716971	1718314	447	+	6985414	COG0531E	cationic amino acid transporter protein
Spy49_1730	hutH	NE	1718326	1719867	513	+	6985415	COG2986E	histidine ammonia-lyase
Spy49_1731	hutG	NE	1720054	1721040	328	+	6985416	COG0010E	formimidoylglutamase
Spy49_1732c	-	NE	1721071	1724145	1024	-	6985417	COG3899R	regulatory protein
Spy49_1733	rpsB	E	1724449	1725216	255	+	6985418	COG0052J	30S ribosomal protein S2
Spy49_1734	tsf	E	1725350	1726390	346	+	6985419	COG0264J	elongation factor Ts
Spy49_1735c	pepO	NC	1726556	1728451	631	-	6985420	COG3590O	endopeptidase O
Spy49_1736c	dexS	E	1728659	1730287	542	-	6985421	COG0366G	trehalose-6-phosphate hydrolase
Spy49_1737c	-	NC	1730354	1732378	674	-	6985422	COG1263G	PTS system enzyme II
Spy49_1738	-	NE	1732589	1733302	237	+	6985423	COG2188K	trehalose operon transcriptional repressor
Spy49_1739c	-	NE	1733581	1733820	79	-	6985424	COG1733K	hypothetical protein Spy49_1739c
Spy49_1740	-	NE	1734105	1734962	285	+	6985425	COG2514R	glyoxalase
Spy49_1742	-	NE	1735004	1735735	243	+	6985426	COG3022S	hypothetical protein Spy49_1742
Spy49_1743c	nrdG	NC	1735909	1736523	204	-	6985427	COG0602O	ribonucleotide reductase of class III (anaerobic), activating protein
Spy49_1744c	-	NE	1736523	1737032	169	-	6985428	COG3981R	hypothetical protein Spy49_1744c
Spy49_1745c	-	E	1737041	1737976	311	-	6985429	COG0673R	oxidoreductase
Spy49_1746c	-	NC	1738005	1738151	48	-	6985430	-	hypothetical protein Spy49_1746c
Spy49_1747c	nrdD	NC	1738333	1740531	732	-	6985431	COG1328F	anaerobic ribonucleoside triphosphate reductase
Spy49_1748c	-	NE	1740628	1742187	519	-	6985432	-	hypothetical protein Spy49_1748c
Spy49_1749c	-	NE	1742600	1742905	101	-	6985433	COG3906S	hypothetical protein Spy49_1749c
Spy49_1750c	-	NC	1742917	1743336	139	-	6985434	COG0816L	Holliday junction resolvase-like protein
Spy49_1751c	-	NE	1743333	1743602	89	-	6985435	COG4472S	hypothetical protein Spy49_1751c
Spy49_1752c	spxA	NE	1743716	1744114	132	-	6985436	COG1393P	transcriptional regulator Spx
Spy49_1753c	recA	NC	1744405	1745541	378	-	6985437	COG0468L	recombinase A
Spy49_1754c	cinA	NE	1745629	1746900	423	-	6985438	COG1058R	competence damage-inducible protein A
Spy49_1755c	tag	NE	1746969	1747529	186	-	6985439	COG2818L	DNA-3-methyladenine glycosylase
Spy49_1756c	ruvA	C	1747539	1748135	198	-	6985440	COG0632L	Holliday junction DNA helicase RuvA
Spy49_1757c	lmrP	C	1748137	1749357	406	-	6985441	-	integral membrane protein
Spy49_1758c	mutL	C	1749368	1751350	660	-	6985442	COG0323L	DNA mismatch repair protein
Spy49_1759c	mutS	NC	1751480	1754035	851	-	6985443	COG0249L	DNA mismatch repair protein MutS
Spy49_1761c	-	NE	1754371	1754706	111	-	6985444	COG1438K	arginine repressor
Spy49_1762	argS	E	1755099	1756790	563	+	6985445	COG0018J	arginyl-tRNA synthetase
Spy49_1763	-	NE	1756878	1757186	102	+	6985446	-	hypothetical protein Spy49_1763
Spy49_1764c	-	NE	1757213	1758085	290	-	6985447	COG1284S	hypothetical protein Spy49_1764c
Spy49_1765c	-	NE	1758128	1759063	311	-	6985448	COG1284S	hypothetical protein Spy49_1765c
Spy49_1767c	-	NE	1759026	1759967	313	-	6985449	COG1284S	hypothetical protein Spy49_1767c
Spy49_1768c	aspS	E	1759960	1761708	582	-	6985450	COG0173J	aspartyl-tRNA synthetase
Spy49_1769c	hisS	E	1762046	1763326	426	-	6985451	COG0124J	histidyl-tRNA synthetase
Spy49_1770	rpmF	NE	1763546	1763728	60	+	6985452	COG0333J	50S ribosomal protein L32
Spy49_1771	rpmG	NC	1763744	1763893	49	+	6985453	COG0267J	50S ribosomal protein L33
Spy49_1772c	-	NE	1763933	1764157	74	-	6985454	-	hypothetical protein Spy49_1772c
Spy49_1773	cadD	NE	1764186	1764800	204	+	6985455	COG4300P	Cadmium resistance protein
Spy49_1774	cadX	NE	1764812	1765150	112	+	6985456	COG0640K	cadmium efflux system accessory
Spy49_1775	-	NE	1765194	1766117	307	+	6985457	-	hypothetical protein Spy49_1775
Spy49_1777	-	NE	1766183	1766935	250	+	6985458	COG1674D	hypothetical protein Spy49_1777
Spy49_1778	-	NE	1766932	1767534	200	+	6985459	-	hypothetical protein Spy49_1778
Spy49_1779	-	NE	1768745	1769587	280	+	6985460	COG2035S	hypothetical protein Spy49_1779
Spy49_1780c	-	NE	1769591	1770274	227	-	6985461	COG1051F	hypothetical protein Spy49_1780c
Spy49_1781	-	NE	1770481	1770807	108	+	6985462	COG1695K	hypothetical protein Spy49_1781
Spy49_1782	-	NE	1770794	1771381	195	+	6986105	COG4709S	hypothetical protein Spy49_1782
Spy49_1783	-	NE	1771378	1772451	357	+	6986106	COG0572F	hypothetical protein Spy49_1783
Spy49_1784c	-	NC	1772567	1774861	764	-	6986107	COG1511S	phage infection protein
Spy49_1785	-	NE	1774975	1775511	178	+	6986108	COG1309K	transcriptional regulator
Spy49_1786c	rpsD	E	1775661	1776272	203	-	6986109	COG0522J	30S ribosomal protein S4
Spy49_1787c	-	NE	1777001	1777273	90	-	6986110	COG4466S	hypothetical protein Spy49_1787c
Spy49_1788c	dnaC	E	1777290	1778657	455	-	6986111	COG0305L	replicative DNA helicase
Spy49_1789c	rplI	NC	1778687	1779139	150	-	6986112	COG0359J	50S ribosomal protein L9
Spy49_1790c	-	E	1779136	1781112	658	-	6986113	COG3887T	DHH family protein
Spy49_1791c	gidA	E	1781202	1783100	632	-	6986114	COG0445D	tRNA uridine 5-carboxymethylaminomethyl modification protein GidA
Spy49_1792c	-	NE	1783224	1783541	105	-	6986115	COG1051F	hypothetical protein Spy49_1792c
Spy49_1793c	mnmA	E	1784316	1785437	373	-	6986116	COG0482J	tRNA-specific 2-thiouridylase MnmA
Spy49_1794	sdhB	NE	1785735	1786406	223	+	6986117	COG1760E	L-serine dehydratase subunit beta
Spy49_1795	sdhA	NE	1786418	1787290	290	+	6986118	COG1760E	L-serine dehydratase subunit alpha
Spy49_1796c	-	NE	1787704	1788318	204	-	6986119	-	hypothetical protein Spy49_1796c
Spy49_1797c	-	E	1788690	1789490	266	-	6986120	COG0619P	cobalt ABC transporter permease
Spy49_1798c	cbiO	E	1789483	1790325	280	-	6986121	COG1122P	cobalt transporter ATP-binding protein
Spy49_1799c	cbiO	E	1790301	1791191	296	-	6986122	COG1122P	cobalt transporter ATP-binding protein
Spy49_1800c	pgsA	E	1791142	1791684	180	-	6986123	COG0558I	CDP-diacylglycerol--glycerol-3-phosphate 3-phosphatidyltransferase
Spy49_1801c	-	NE	1791698	1792723	341	-	6986124	COG1426S	hypothetical protein Spy49_1801c
Spy49_1802c	-	NE	1792773	1794062	429	-	6986125	COG0612R	hypothetical protein Spy49_1802c
Spy49_1803c	-	NE	1794064	1795308	414	-	6986126	COG0612R	hypothetical protein Spy49_1803c
Spy49_1804	hasA	NE	1795670	1797007	445	+	6986127	COG1215M	hyaluronan synthase
Spy49_1806	hasB	NE	1797043	1798251	402	+	6986128	COG1004M	UDP-glucose 6-dehydrogenase
Spy49_1808	hasC	NE	1798433	1799347	304	+	6986129	COG1210M	UDP-glucose pyrophosphorylase
Spy49_1809	-	NE	1799655	1800068	137	+	6986130	COG2501S	hypothetical protein Spy49_1809
Spy49_1810	recF	NE	1800070	1801176	368	+	6986131	COG1195L	recombination protein F
Spy49_1811c	-	NE	1801232	1802095	287	-	6986132	COG4975G	hypothetical protein Spy49_1811c
Spy49_1812c	guaB	NE	1802298	1803779	493	-	6986133	COG0516F	inosine 5\'-monophosphate dehydrogenase
Spy49_1813c	trsA	E	1804087	1805109	340	-	6986134	COG0180J	tryptophanyl-tRNA synthetase II
Spy49_1814	-	NE	1805528	1806400	290	+	6986135	COG1284S	hypothetical protein Spy49_1814
Spy49_1815	-	NE	1806479	1808086	535	+	6986136	COG0488R	ABC transporter ATP-binding protein
Spy49_1817	-	NE	1808184	1810760	858	+	6986137	COG4485S	ABC transporter permease
Spy49_1820c	-	NE	1813005	1813484	159	-	6986141	COG1576S	rRNA large subunit methyltransferase
Spy49_1821	degP	E	1813696	1814919	407	+	6986142	COG0265O	endopeptidase degP
Spy49_1822	parB	NE	1814978	1815784	268	+	6986143	COG1475K	Chromosome partitioning protein parB

Table S7. Conserved GAS essential genes identified in both M1T1 5448 and M49 NZ131 compared to the predicted GAS core genome.

(a) Spy numbers from MGAS5005 genome.
(b) Hyperlink to the NCBI Gene website (http://www.ncbi.nlm.nih.gov/gene).
(c) Call integrating data from Bayesian analyses on 3 time points.
(d) Spy numbers from GAS NZ131 genome.
(e) When available, gene name is provided.
(f)Asteriks refer to genes found in the GAS core genome.
(g) *S. sanguinis* ortholog found essential.
(h) *S. peumoniae* ortholog found essential.

Essential in both GAS strains
Essential or Critical in one GAS strain, but not the other
Critical for both GAS strains or Critical for one and Essential for the other

GAS M1T1 5448			GAS M49 NZ131			Gene	GAS Core	Found essential in other Strep. species		
Locus Tag(a)	Gene ID(b)	Call(c)	Locus Tag(d)	Gene ID (b)	Call(c)	Name(e)	Genome(f)	*S. sanguinis*(g)	*S. pneumoniae*(h)	Protein Function
M5005_Spy_0001	3571011	E	Spy49_0001	6986144	NC	dnaA	*	SSA_0001	SP_0001	chromosome replication initiator DnaA
M5005_Spy_0002	3571012	E	Spy49_0002	6984517	E	**dnaN**	*	SSA_0002	SP_0002	DNA polymerase III subunit beta
M5005_Spy_0003	3571013	NE	Spy49_0003	6984518	NE	-				hypothetical protein M5005_Spy_0003
M5005_Spy_0004	3571014	NE	Spy49_0004	6984519	NE	ychF	*			GTP-dependent nucleic acid-binding protein EngD
M5005_Spy_0005	3571015	NC	Spy49_0005	6984520	E	pth	*	SSA_0006		peptidyl-tRNA hydrolase
M5005_Spy_0006	3571016	NE	Spy49_0006	6984521	NE	trcF	*			transcription-repair coupling factor
M5005_Spy_0007	3572946	NE	Spy49_0008	6984522	NE	-	*			heat shock protein 15
M5005_Spy_0008	3572947	NE	Spy49_0009	6984523	NC	divIC	*			cell division protein
M5005_Spy_0009	3572948	NC				-				hypothetical protein M5005_Spy_0009
M5005_Spy_0010	3572949	NE	Spy49_0010	6984524	NE	-	*			beta-lactamase
M5005_Spy_0011	3572950	E	Spy49_0011	6984525	E	**tilS/mesJ**	*	SSA_0013	SP_0011	tRNA(Ile)-lysidine synthetase
M5005_Spy_0012	3572951	NC	Spy49_0012	6984526	NE	hpt				hypoxanthine-guanine phosphoribosyltransferase
M5005_Spy_0013	3572952	E	Spy49_0013	6984527	E	**ftsH**	*		SP_0013	cell division protein
M5005_Spy_0014	3572953	NE	Spy49_0014	6984528	NE	-	*			amino acid permease
M5005_Spy_0015c	3572954	NC				-				hypothetical protein M5005_Spy_0015
M5005_Spy_0017	3572901	E	Spy49_0015	6984563	E	**sibA**	*		SP_2216	secreted protein
M5005_Spy_0018	3572902	E	Spy49_0016	6984564	E	**prsA.2**	*			ribose-phosphate pyrophosphokinase
M5005_Spy_0019	3572903	NE	Spy49_0017	6984565	NE	recO	*			DNA repair protein RecO
M5005_Spy_0020	3572904	E	Spy49_0018	6984566	E	**plsX**	*		SP_0027	glycerol-3-phosphate acyltransferase
M5005_Spy_0021	3572905	NC	Spy49_0019	6984567	NC	acpP.2				acyl carrier protein
M5005_Spy_0022	3572906	NC	Spy49_0020	6984568	NE	purC				phosphoribosylaminoimidazole-succinocarboxamide synthase
M5005_Spy_0023	3572907	NE	Spy49_0021	6984569	NE	purL	*			phosphoribosylformylglycinamidine synthase
M5005_Spy_0024	3572908	NE	Spy49_0022	6984570	NE	purF	*			amidophosphoribosyltransferase
M5005_Spy_0025	3572909	NE	Spy49_0023	6984571	NE	purM	*			phosphoribosylaminoimidazole synthetase
M5005_Spy_0026	3572910	NE	Spy49_0024	6984572	NE	purN	*			phosphoribosylglycinamide formyltransferase
M5005_Spy_0027	3572911	NE	Spy49_0025	6984573	NE	purH				bifunctional phosphoribosylaminoimidazolecarboxamide formyltransferase/IMP cyclohydrolase
M5005_Spy_0028c	3572912	NE	Spy49_0026c	6984574	NE	-	*			autolysin
M5005_Spy_0029	3572913	NE	Spy49_0027	6984575	NE	purD	*			phosphoribosylamine--glycine ligase
M5005_Spy_0030	3572914	NE	Spy49_0028	6984576	NE	purE	*			phosphoribosylaminoimidazole carboxylase catalytic subunit
M5005_Spy_0031	3572915	NE	Spy49_0029	6984577	NE	purK	*			phosphoribosylaminoimidazole carboxylase ATPase subunit
M5005_Spy_0032	3572916	NE	Spy49_0030	6984578	NE	-				hypothetical protein M5005_Spy_0032
M5005_Spy_0033	3572917	NE	Spy49_0031	6984579	NE	purB	*			adenylosuccinate lyase
M5005_Spy_0034	3572880	NE	Spy49_0032	6984580	NE	-				transcriptional regulator
M5005_Spy_0035	3572881	NE	Spy49_0033	6984581	C	ruvB	*		SP_0259	Holliday junction DNA helicase RuvB
M5005_Spy_0036	3572882	NE	Spy49_0035	6984582	NE	-	*			protein tyrosine phosphatase
M5005_Spy_0037	3572883	NC	Spy49_0036	6984663	E	-	*			hypothetical protein M5005_Spy_0037
M5005_Spy_0038	3572884	E	Spy49_0037	6984664	E	**-**	*			acyltransferase
M5005_Spy_0039	3572885	NE	Spy49_0038	6984665	NE	adh2/adhE				bifunctional acetaldehyde-CoA/alcohol dehydrogenase
M5005_Spy_0040	3572886	NE	Spy49_0039	6984666	NE	adhA/adhP	*			alcohol dehydrogenase
M5005_Spy_0041	3572887	NE	Spy49_0040	6984667	NE	norM	*			Na+ driven multidrug efflux pump
M5005_Spy_0043	3572889	E	Spy49_0044	6984668	NC	rpsJ	*	SSA_0106	SP_0208	30S ribosomal protein S10
M5005_Spy_0044	3572890	E	Spy49_0045	6984669	E	**rplC**	*	SSA_0107	SP_0209	50S ribosomal protein L3
M5005_Spy_0045	3572891	E	Spy49_0046	6984670	NC	rplD	*	SSA_0108	SP_0210	50S ribosomal protein L4
M5005_Spy_0046	3572892	NE	Spy49_0048	6984671	NC	rplW	*		SP_0211	50S ribosomal protein L23
M5005_Spy_0047	3572893	E	Spy49_0049	6984672	E	**rplB**	*	SSA_0110	SP_0212	50S ribosomal protein L2
M5005_Spy_0048	3572894	C	Spy49_0050	6984673	NC	rpsS	*	SSA_0111	SP_0213	30S ribosomal protein S19
M5005_Spy_0049	3572895	E	Spy49_0052	6984674	NC	rplV	*	SSA_0112	SP_0214	50S ribosomal protein L22
M5005_Spy_0050	3572896	E	Spy49_0053	6984675	E	**rpsC**	*	SSA_0113	SP_0215	30S ribosomal protein S3
M5005_Spy_0051	3572897	E	Spy49_0054	6984676	E	**rplP**	*	SSA_0114	SP_0216	50S ribosomal protein L16
M5005_Spy_0052	3572898	NE	Spy49_0055	6984677	NC	rpmC				50S ribosomal protein L29
M5005_Spy_0053	3572899	NC	Spy49_0056	6984678	NC	rpsQ				30S ribosomal protein S17
M5005_Spy_0054	3572861	C	Spy49_0057	6984679	NC	rplN	*	SSA_0117	SP_0219	50S ribosomal protein L14
M5005_Spy_0055	3572862	NC	Spy49_0058	6984680	E	rplX	*		SP_0220	50S ribosomal protein L24
M5005_Spy_0056	3572863	NC	Spy49_0059	6984681	NC	rplE	*			50S ribosomal protein L5
M5005_Spy_0057	3572864	NC	Spy49_0060	6984682	NC	rpsN				30S ribosomal protein S14
M5005_Spy_0058	3572865	NC	Spy49_0061	6984683	NC	rpsH	*			30S ribosomal protein S8
M5005_Spy_0059	3572866	E	Spy49_0062	6984684	E	**rplF**	*	SSA_0122	SP_0225	50S ribosomal protein L6
M5005_Spy_0060	3572867	NC	Spy49_0064	6984685	NC	rplR	*			50S ribosomal protein L18
M5005_Spy_0061	3572868	E	Spy49_0065	6984686	NC	rpsE	*	SSA_0124	SP_0227	30S ribosomal protein S5
M5005_Spy_0062	3572869	E	Spy49_0066	6984687	NC	rpmD			SP_0228	50S ribosomal protein L30
M5005_Spy_0063	3572870	NE	Spy49_0067	6984688	NC	rplO	*			50S ribosomal protein L15
M5005_Spy_0064	3572871	E	Spy49_0068	6984689	E	**secY**	*	SSA_0127		preprotein translocase subunit SecY
M5005_Spy_0065	3572872	NC	Spy49_0069	6984690	E	adk	*	SSA_0128	SP_0231	adenylate kinase
M5005_Spy_0066	3572873	E	Spy49_0070	6984691	NC	infA		SSA_0129	SP_0232	translation initiation factor IF-1
M5005_Spy_0067	3572874	NC	Spy49_0071	6984692	NC	rpmJ				50S ribosomal protein L36
M5005_Spy_0068	3572875	E	Spy49_0072	6984693	E	rpsM			SP_0234	30S ribosomal protein S13
M5005_Spy_0069	3572876	E	Spy49_0073	6984694	NC	rpsK	*	SSA_0131	SP_0235	30S ribosomal protein S11
M5005_Spy_0070	3572877	E	Spy49_0074	6984695	E	**rpoA**	*	SSA_0132	SP_0236	DNA-directed RNA polymerase subunit alpha
M5005_Spy_0071	3572878	E	Spy49_0075	6984696	NC	rplQ	*	SSA_0133	SP_0237	50S ribosomal protein L17
M5005_Spy_0072c	3572879	NC				-				hypothetical protein M5005_Spy_0072
M5005_Spy_0073	3572845	NC				-				hypothetical protein M5005_Spy_0073
M5005_Spy_0074	3572846	NC				-				4-diphosphocytidyl-2-C-methyl-D-erythritol kinase
M5005_Spy_0075	3572847	NC	Spy49_0078	6984713	NE	-				4-diphosphocytidyl-2-C-methyl-D-erythritol kinase, partial
M5005_Spy_0076	3572848	NC	Spy49_0079	6984714	NE	-				4-diphosphocytidyl-2-C-methyl-D-erythritol kinase
M5005_Spy_0077	3572849	NE	Spy49_0080	6984715	NE	adcR	*			MarR family transcriptional regulator
M5005_Spy_0078	3572850	NE	Spy49_0081	6984716	NC	adcC	*			high-affinity zinc uptake system ATP-binding protein
M5005_Spy_0079	3572819	NE	Spy49_0082	6984717	NC	adcB	*			high-affinity zinc uptake system membrane protein
M5005_Spy_0080c	3572820	NE	Spy49_0083c	6984718	NE	-				bis(5\'-nucleosyl)-tetraphosphatase (asymmetrical)
M5005_Spy_0081c	3572821	E	Spy49_0084	6984719	E	**tyrS**	*	SSA_0174	SP_2100	tyrosyl-tRNA synthetase
M5005_Spy_0082	3572822	NE	Spy49_0086	6984720	NE	pbp1b	*			multimodular transpeptidase-transglycosylase PBP 1B
M5005_Spy_0083	3572823	E	Spy49_0087	6984721	E	**rpoB**	*	SSA_0176	SP_1961	DNA-directed RNA polymerase subunit beta
M5005_Spy_0084	3572824	E	Spy49_0088	6984722	E	**rpoC**	*	SSA_0177	SP_1960	DNA-directed RNA polymerase subunit beta\'
M5005_Spy_0085	3572825	NE	Spy49_0089	6984723	NE	-	*			DNA binding protein
M5005_Spy_0086	3572826	NE	Spy49_0090	6984724	NE	comYA	*			competence protein ComG
M5005_Spy_0087	3572827	NE	Spy49_0091	6984725	NE	comYB	*			competence protein ComG
M5005_Spy_0088	3572828	NE	Spy49_0092	6984726	NE	comYC	*			competence protein ComG
M5005_Spy_0089	3572829	NE	Spy49_0093	6984727	NE	-	*			competence protein ComG
M5005_Spy_0090	3572830	NE	Spy49_0094	6984728	NE	-				hypothetical protein M5005_Spy_0090
M5005_Spy_0091	3572831	NE	Spy49_0095	6984729	NE	comYD	*			competence protein ComG
M5005_Spy_0092	3572832	NE	Spy49_0096	6984730	NE	-	*			competence protein ComG

M5005_Spy_0093	3572833	NE	Spy49_0097	6984731	NE	-	*			adenine-specific methyltransferase
M5005_Spy_0094	3572834	NE	Spy49_0098	6984732	NC	ackA	*			acetate kinase
M5005_Spy_0095	3572835	NE	Spy49_0099	6984733	NE	-				hypothetical protein M5005_Spy_0095
M5005_Spy_0096c	3572836	NE	Spy49_0100	6984734	NE	proC	*			pyrroline-5-carboxylate reductase
M5005_Spy_0097c	3572837	NE	Spy49_0101	6984735	NE	pepA	*			glutamyl aminopeptidase
M5005_Spy_0098	3572838	NC	Spy49_0103	6984736	NE	-				hypothetical protein M5005_Spy_0098
M5005_Spy_0099	3572800	NE				-	*			hypothetical protein M5005_Spy_0099
M5005_Spy_0100	3572801	NE	Spy49_0104	6984737	NE	trx.1				thioredoxin
M5005_Spy_0101	3572802	NE	Spy49_0105	6984738	NE	-				tRNA-binding domain-containing protein
M5005_Spy_0102	3572803	NE	Spy49_0107	6984739	NE	ssb				single-stranded DNA-binding protein
M5005_Spy_0103c	3572804	NE	Spy49_0108c	6984740	NE	-	*			deoxyadenosine kinase/deoxyguanosine kinase
M5005_Spy_0104c	3572805	NE	Spy49_0109c	6984741	NE	-	*			tRNA-dihydrouridine synthase
M5005_Spy_0105c	3572806	NE	Spy49_0110c	6984742	NE	hslO	*			heat shock protein 33
M5005_Spy_0106c	3572807	NE	Spy49_0111	6984823	NE	rofA/nra				transcriptional regulator
M5005_Spy_0107	3572808	NE	Spy49_0112	6984824	NE	cbp				fibronectin-binding protein
M5005_Spy_0108	3572809	NE	Spy49_0113	6984825	NE	lepA-1				signal peptidase I
M5005_Spy_0109	3572810	NE	Spy49_0114	6984826	NE	prtF				fibronectin-binding protein
M5005_Spy_0110	3572811	NE	Spy49_0116	6984827	NE	eftLSL.B				hypothetical protein M5005_Spy_0110
M5005_Spy_0111	3572812	NE	Spy49_0117	6984828	NE	-				hypothetical protein M5005_Spy_0111
M5005_Spy_0112c	3572813	NE				-				transposase
M5005_Spy_0113c	3572814	NE				-				transposase
M5005_Spy_0114	3572815	NE				-				sortase
			Spy49_0118	6984829	NE	msmR				
			Spy49_0119	6984830	NE	prtF				
			Spy49_0120c	6984831	NE					
M5005_Spy_0115c	3572816	NE				-	*			hypothetical protein M5005_Spy_0115
M5005_Spy_0116	3572817	NE	Spy49_0121	6984832	NE	atoE	*			short-chain fatty acids transporter
M5005_Spy_0117c	3572818	NE				-				LysR family transcriptional regulator
M5005_Spy_0118c	3572780	NE	Spy49_0122c	6984833	NE	-				LysR family transcriptional regulator
M5005_Spy_0119	3572781	NE	Spy49_0123	6984834	NE	-				acetyl-CoA acetyltransferase
M5005_Spy_0120	3572782	NE	Spy49_0124	6984835	NE	atoD.2				acetate CoA-transferase subunit alpha
M5005_Spy_0121	3572783	NE	Spy49_0125	6984836	NE	-	*			acetyl-CoA:acetoacetyl-CoA transferase subunit beta
M5005_Spy_0122c	3572784	NE	Spy49_0126c	6984837	NE	-	*			DNA-binding protein
M5005_Spy_0123	3572785	NE	Spy49_0127	6984838	NE	-	*			translation initiation inhibitor
M5005_Spy_0124	3572786	NE	Spy49_0128	6984839	NE	sloR	*			transcriptional regulator
M5005_Spy_0125	3572787	NE	Spy49_0129	6984840	NE	-	*			hypothetical protein M5005_Spy_0125
M5005_Spy_0126	3572788	NE	Spy49_0130	6984841	NE	ntpI	*			V-type ATP synthase subunit I
M5005_Spy_0127	3572789	NE	Spy49_0131	6984842	NE	ntpK	*			V-type ATP synthase subunit K
M5005_Spy_0128	3572790	NE	Spy49_0132	6984843	NE	ntpE	*			V-type sodium ATP synthase subunit E
M5005_Spy_0129	3572791	NE	Spy49_0133	6984844	NE	ntpC	*			V-type ATP synthase subunit C
M5005_Spy_0130	3572792	NE	Spy49_0134	6984845	NE	ntpF				V-type ATP synthase subunit F
M5005_Spy_0131	3572793	NE	Spy49_0135	6984846	NE	ntpA	*			V-type ATP synthase subunit A
M5005_Spy_0132	3572794	NE	Spy49_0136	6984847	NE	ntpB	*			V-type ATP synthase subunit B
M5005_Spy_0133	3572795	NE	Spy49_0137	6984848	NE	ntpD	*			V-type ATP synthase subunit D
M5005_Spy_0134c	3572796	NE	Spy49_0138c	6984849	NE	-	*			tellurite resistance protein
M5005_Spy_0135c	3572797	NE	Spy49_0139c	6984850	NE	-	*			hypothetical protein M5005_Spy_0135
M5005_Spy_0136	3572798	NE	Spy49_0141	6984851	NE	purA	*			adenylosuccinate synthetase
M5005_Spy_0137	3572799	NE	Spy49_0142	6984852	NE	-	*			nucleoside-binding protein
M5005_Spy_0138	3572761	C	Spy49_0143	6984853	NC	nusG	*		SP_2007	transcription antitermination protein NusG
M5005_Spy_0139	3572762	NE	Spy49_0144	6984854	NE	nga	*			NAD glycohydrolase
M5005_Spy_0140	3572763	NE	Spy49_0145	6984855	NE	-				hypothetical protein M5005_Spy_0140
M5005_Spy_0141	3572764	NE	Spy49_0146	6984856	NE	slo	*			streptolysin O
M5005_Spy_0142	3572765	NE	Spy49_0147	6984857	NC	-				hypothetical protein M5005_Spy_0142
M5005_Spy_0143c	3572766	NE	Spy49_0148	6984858	NC	-				hypothetical protein M5005_Spy_0143
M5005_Spy_0144c	3572767	NC	Spy49_0149c	6984859	NE	-				hypothetical protein M5005_Spy_0144
M5005_Spy_0145c	3572768	NE	Spy49_0150c	6984860	E	-				hypothetical protein M5005_Spy_0145
M5005_Spy_0146	3572769	NE	Spy49_0151	6984861	NE	metB				cystathionine beta-lyase
M5005_Spy_0147	3572770	E	Spy49_0152	6984862	E	**leuS**	*	SSA_0289	SP_0254	leucyl-tRNA synthetase
M5005_Spy_0148	3572771	NC	Spy49_0153	6984863	NE	ulaA	*			PTS system ascorbate-specific transporter subunit IIC
M5005_Spy_0149	3572772	NE	Spy49_0154	6984864	NE	-	*			PTS system 3-keto-L-gulonate specific transporter subunit IIB
M5005_Spy_0150	3572773	NE	Spy49_0155	6984865	NE	-	*			PTS system 3-keto-L-gulonate specific transporter subunit IIA
M5005_Spy_0151	3572774	NE	Spy49_0156	6984866	NC	ulaD	*			3-keto-L-gulonate-6-phosphate decarboxylase
M5005_Spy_0152	3572775	NE	Spy49_0157	6984867	NE	-	*			L-xylulose 5-phosphate 3-epimerase
M5005_Spy_0153	3572776	NE	Spy49_0158	6984868	NE	araD	*			L-ribulose-5-phosphate 4-epimerase
M5005_Spy_0154	3572777	NC				-				hypothetical protein M5005_Spy_0154
M5005_Spy_0155	3572778	NE	Spy49_0159	6984869	NE	-	*			BigG family transcription antiterminator
M5005_Spy_0156	3572779	NE	Spy49_0160	6984870	NE	-	*			L-ascorbate 6-phosphate lactonase
M5005_Spy_0157	3572741	NE	Spy49_0161	6984871	NE	opuAA	*			glycine betaine transport ATP-binding protein
M5005_Spy_0158	3572742	NE	Spy49_0162	6984872	NE	opuABC	*			glycine betaine-binding protein/glycine betaine transporter permease
M5005_Spy_0159	3572743	E	Spy49_0163	6984873	E	**polA**	*	SSA_0100	SP_0032	DNA polymerase I
M5005_Spy_0160	3572744	NE	Spy49_0164	6984874	NE	-	*			CoA binding protein
M5005_Spy_0161	3572745	NE	Spy49_0165	6984875	NE	perR	*			ferric uptake regulation protein
M5005_Spy_0162	3572746	NE	Spy49_0166	6984876	NE	vlg				trans-acting positive regulator Mry
M5005_Spy_0163	3572747	NE	Spy49_0167	6984877	NE	-				3\'-phosphoadenosine 5\'-phosphosulfate sulfotransferase
M5005_Spy_0164	3572748	NE	Spy49_0168	6984878	NE	-				parB-like nuclease
M5005_Spy_0165c	3572749	NE	Spy49_0169	6984879	NE	-				transposase
M5005_Spy_0166c	3572750	NE	Spy49_0170c	6984880	NE	-				transposase
M5005_Spy_0167c	3572751	NE				-				transposase
M5005_Spy_0168c	3572752	NE	Spy49_0173c	6984881	NE	-				transposase
M5005_Spy_0169c	3572753	NE				-	*			malonate permease
M5005_Spy_0170	3572754	NE	Spy49_0176	6984883	NE	nadC				nicotinate-nucleotide pyrophosphorylase
M5005_Spy_0171c	3572755	NE	Spy49_0178c	6984884	NE	-				transposase
M5005_Spy_0172c	3572756	NE				-				transposase
M5005_Spy_0173c	3572757	NE	Spy49_0180c	6984885	NE	-	*			hypothetical protein M5005_Spy_0173
M5005_Spy_0174	3572758	NC				-				hypothetical protein M5005_Spy_0174
M5005_Spy_0175	3572759	NE	Spy49_0181	6984886	NE	tgt	*			queuine tRNA-ribosyltransferase
M5005_Spy_0176	3572760	NE	Spy49_0182	6984887	NE	-	*			hypothetical protein M5005_Spy_0176
M5005_Spy_0177	3572722	NE	Spy49_0183	6984888	NE	bioY	*			bioY protein
M5005_Spy_0178	3572723	NE	Spy49_0184	6984889	NE	-	*			metal-dependent hydrolase
M5005_Spy_0179	3572724	NE	Spy49_0185	6984890	NE	-				tRNA-specific adenosine deaminase
M5005_Spy_0180c	3572725	NE	Spy49_0186c	6984891	NE	-	*			S-layer protein
M5005_Spy_0181	3572726	NC				-				hypothetical protein M5005_Spy_0181
M5005_Spy_0182	3572727	NE	Spy49_0187	6984892	NE	speG				exotoxin type G
M5005_Spy_0183	3572728	NC				-				hypothetical protein M5005_Spy_0183
M5005_Spy_0184	3572729	NC				-				hypothetical protein M5005_Spy_0184
M5005_Spy_0185	3572730	E	Spy49_0188	6984893	E	**pgi**	*	SSA_2183	SP_2070	glucose-6-phosphate isomerase
M5005_Spy_0186c	3572731	NE	Spy49_0189c	6984894	NE	ralp4	*			transcriptional regulator
M5005_Spy_0187	3572732	NE				-				transposase
M5005_Spy_0188	3572733	NE				-				transposase
M5005_Spy_0189	3572734	NC				-				hypothetical protein M5005_Spy_0189
M5005_Spy_0190	3572735	NC				-				hypothetical protein M5005_Spy_0190
M5005_Spy_0191	3572736	NE	Spy49_0190	6984895	NE	-	*			rhomboid family integral membrane protein

M5005_Spy_0192c	3572737	NC	Spy49_0191c	6984896	E	hasC.2	*	SSA_2169	SP_2092	UTP-glucose-1-phosphate uridylyltransferase
M5005_Spy_0194c	3572739	E	Spy49_0192c	6984897	E	**gpsA**	*		SP_2091	glycerol-3-phosphate dehydrogenase, partial
M5005_Spy_0195	3572740	NE	Spy49_0193	6984898	NE	-	*			MarR family transcriptional regulator
M5005_Spy_0196	3572702	NE	Spy49_0194	6984899	NC	-	*			multidrug resistance ABC transporter ATP-binding protein/permease
M5005_Spy_0197	3572703	NE	Spy49_0195	6984900	NE	-	*			multidrug resistance ABC transporter ATP-binding protein/permease
M5005_Spy_0198	3572704	NE	Spy49_0198	6984901	NE	-	*			hypothetical protein M5005_Spy_0198
M5005_Spy_0199	3572705	NE	Spy49_0199	6984902	NE	dut	*			deoxyuridine 5\'-triphosphate nucleotidohydrolase
M5005_Spy_0200	3572706	NE	Spy49_0200	6984983	NE	radA	*			DNA repair protein RadA
M5005_Spy_0201	3572707	C	Spy49_0201	6984984	NC	-	*			carbonic anhydrase
M5005_Spy_0202	3572708	NE	Spy49_0202	6984985	NE	-	*			hypothetical protein M5005_Spy_0202
M5005_Spy_0203	3572709	E	Spy49_0203	6984986	E	**gltX**	*	SSA_2144	SP_2069	glutamyl-tRNA synthetase
M5005_Spy_0204	3572710	NE	Spy49_0204	6984987	NE	fasB	*			sensory transduction protein kinase
M5005_Spy_0205	3572711	NE	Spy49_0205	6984988	NC	fasC				sensory transduction protein kinase
M5005_Spy_0206	3572712	NE	Spy49_0206	6984989	NE	fasA	*			response regulator
M5005_Spy_0207	3572713	C	Spy49_0207	6984990	NC	rnpA	*	SSA_2140	SP_2042	ribonuclease P
M5005_Spy_0208	3572714	NE	Spy49_0208	6984991	NE	-	*			hypothetical protein M5005_Spy_0208
M5005_Spy_0209	3572715	NE	Spy49_0209	6984992	NC	-	*			jag protein
M5005_Spy_0210	3572716	NC				-				hypothetical protein M5005_Spy_0210
M5005_Spy_0211	3572717	NC	Spy49_0210	6984993	NC	rpmH				50S ribosomal protein L34
M5005_Spy_0212	3572718	NE	Spy49_0211	6984994	NE	-	*			N-acetylmannosamine-6-phosphate 2-epimerase
M5005_Spy_0213	3572719	NE	Spy49_0212	6984995	NE	-	*			N-acetylneuraminate-binding protein
M5005_Spy_0214	3572720	NE	Spy49_0213	6984996	NE	-	*			N-acetylneuraminate transporter permease
M5005_Spy_0215	3572721	NE	Spy49_0214	6984997	NE	-	*			N-acetylneuraminate transporter permease
M5005_Spy_0216	3572683	NE	Spy49_0215	6984998	NE	-	*			hypothetical protein M5005_Spy_0216
M5005_Spy_0217	3572684	NE	Spy49_0216	6984999	NE	nanH	*			N-acetylneuraminate lyase
M5005_Spy_0218	3572685	NE	Spy49_0217	6985000	NE	-	*			N-acetylmannosamine kinase
M5005_Spy_0219c	3572686	NE	Spy49_0218c	6985001	NE	-	*			RpiR family transcriptional regulator
M5005_Spy_0220	3572687	NE	Spy49_0219	6985002	NE	tatD	*			sec-independent protein translocase
M5005_Spy_0221	3572688	NE	Spy49_0220	6985003	NE	-	*			ribonuclease M5
M5005_Spy_0222	3572689	NE	Spy49_0221	6985004	NE	ksgA	*			dimethyladenosine transferase
M5005_Spy_0223	3572690	E	Spy49_0223	6985005	NE	engC	*		SP_1984	ribosome-associated GTPase
M5005_Spy_0224	3572691	C	Spy49_0224	6985006	NC	rpe	*			ribulose-phosphate 3-epimerase
M5005_Spy_0225	3572692	NE	Spy49_0225	6985007	NE	-	*			thiamin pyrophosphokinase
M5005_Spy_0226	3572693	NE	Spy49_0226	6985008	NE	-	*			rmuC family protein
M5005_Spy_0227	3572694	NE	Spy49_0227	6985009	NE	cbf	*			CMP-binding factor
M5005_Spy_0228	3572695	NE	Spy49_0228	6985010	NE	purR	*			pur operon repressor
M5005_Spy_0229	3572696	NE	Spy49_0229	6985011	NE	prgA	*			surface exclusion protein
M5005_Spy_0230	3572697	E	Spy49_0230	6985012	NC	rpsL	*	SSA_2111	SP_0271	30S ribosomal protein S12
M5005_Spy_0231	3572698	E	Spy49_0231	6985013	E	**rpsG**	*	SSA_2110	SP_0272	30S ribosomal protein S7
M5005_Spy_0232	3572699	E	Spy49_0233	6985014	E	**fus**	*	SSA_2109	SP_0273	elongation factor G
M5005_Spy_0233	3572700	E	Spy49_0234	6985015	E	**plr**	*	SSA_2108	SP_2012	glyceraldehyde-3-phosphate dehydrogenase
M5005_Spy_0234c	3572701	NC	Spy49_0235c	6985016	NC	-				hypothetical protein M5005_Spy_0234
M5005_Spy_0235c	3572663	E	Spy49_0236c	6985017	NE	-	*		SP_1460	amino acid transport ATP-binding protein
M5005_Spy_0236c	3572664	E	Spy49_0237c	6985018	NE	-	*		SP_1461	amino acid ABC transporter permease
M5005_Spy_0237	3572665	NE	Spy49_0238	6985019	NE	-	*			hypothetical protein M5005_Spy_0237
M5005_Spy_0238	3572666	NE	Spy49_0239	6985020	NE	uppP/bacA	*			undecaprenyl pyrophosphate phosphatase
M5005_Spy_0239	3572667	NE	Spy49_0240	6985021	NE	mecA	*			adaptor protein
M5005_Spy_0240	3572668	E	Spy49_0241	6985022	E	**rgpG**	*	SSA_1870	SP_0337	undecaprenyl-phosphate alpha-N-acetylglucosaminephosphotransferase
M5005_Spy_0241	3572669	NC				rgpG				hypothetical protein M5005_Spy_0241
M5005_Spy_0242	3572670	C	Spy49_0242	6985023	NC	-	*		SP_1541	ABC transporter ATP-binding protein
M5005_Spy_0243	3572671	C	Spy49_0243	6985024	C	-	*		SP_0868	ABC transporter
M5005_Spy_0244	3572672	C	Spy49_0244	6985025	NC	nifS3	*		SP_0869	cysteine desulfhydrase
M5005_Spy_0245	3572673	C	Spy49_0245	6985026	NC	nifU	*		SP_0870	iscU protein
M5005_Spy_0246	3572674	C	Spy49_0246	6985027	NC	-	*		SP_0871	ABC transporter
M5005_Spy_0247c	3572675	NE	Spy49_0247	6985028	NE	pbp7				D-alanyl-D-alanine carboxypeptidase
M5005_Spy_0248c	3572676	NE	Spy49_0248c	6985029	NE	dacA2	*			D-alanyl-D-alanine carboxypeptidase
M5005_Spy_0249	3572677	NE	Spy49_0249	6985030	NE	oppA				oligopeptide-binding protein
M5005_Spy_0250	3572678	NE	Spy49_0250	6985031	NE	oppB	*			oligopeptide transporter permease
M5005_Spy_0251	3572679	NE	Spy49_0251	6985032	NE	oppC	*			oligopeptide transporter permease
M5005_Spy_0252	3572680	NE	Spy49_0252	6985033	NE	oppD	*			oligopeptide transport ATP-binding protein
M5005_Spy_0253	3572681	NE	Spy49_0253	6985034	NE	oppF	*			oligopeptide transport ATP-binding protein
M5005_Spy_0254c	3572682	NC	Spy49_0254c	6985035	NE	-				transposase
M5005_Spy_0255c	3572650	NC	Spy49_0256	6985042	NE	-				hypothetical protein M5005_Spy_0255
M5005_Spy_0256	3572657	NE				comX1.1				competence-specific sigma factor
M5005_Spy_0257	3572658	NE				-				transposase, partial
M5005_Spy_0258	3572659	NE				-				transposase
M5005_Spy_0259	3572660	NC				-				hypothetical protein M5005_Spy_0259
M5005_Spy_0260	3572661	E	Spy49_0257	6985043	C	-	*	SSA_0575	SP_1750	lipase
M5005_Spy_0261	3572662	C	Spy49_0258	6985044	E	-	*	SSA_1189	SP_1749	GTP-binding protein YqeH
M5005_Spy_0262	3572630	NE	Spy49_0259	6985045	NE	-				RNA-binding protein
M5005_Spy_0263	3572631	E	Spy49_0260	6985046	NC	nadD	*			nicotinic acid mononucleotide adenylyltransferase
M5005_Spy_0264	3572632	NE	Spy49_0261	6985047	NE	-	*			HAD superfamily hydrolase
M5005_Spy_0265	3572633	NE	Spy49_0262	6985048	NE	-	*			iojap superfamily protein
M5005_Spy_0266	3572634	NE	Spy49_0263	6985049	NE	-	*			methyltransferase
M5005_Spy_0267	3572635	NE	Spy49_0264	6985050	NE	-	*			hypothetical protein M5005_Spy_0267
M5005_Spy_0268	3572636	NC				-				hypothetical protein M5005_Spy_0268
M5005_Spy_0269	3572637	NE	Spy49_0265	6985051	NE	-	*			hypothetical protein M5005_Spy_0269
M5005_Spy_0270	3572638	NE	Spy49_0266	6985052	NC	atmA	*			ABC transporter substrate-binding protein
M5005_Spy_0271	3572639	NE	Spy49_0268	6985053	NE	atmB	*			ABC transporter substrate-binding protein
M5005_Spy_0272	3572640	NE	Spy49_0269	6985054	NE	atmD	*			ABC transporter ATP-binding protein
M5005_Spy_0273	3572641	NE	Spy49_0270	6985055	NE	atmE	*			ABC transporter permease
M5005_Spy_0274c	3572642	NE	Spy49_0271c	6985056	NE	braB				branched-chain amino acid transporter carrier protein
M5005_Spy_0275	3572643	NE	Spy49_0272	6985057	NE	-	*			serine/threonine transporter SstT
M5005_Spy_0276c	3572644	C	Spy49_0273c	6985058	E	-	*			potassium uptake protein
M5005_Spy_0277c	3572645	NE	Spy49_0274c	6985059	E	-	*			potassium uptake protein
M5005_Spy_0278c	3572646	NE	Spy49_0275	6985060	NE	gidB	*			16S rRNA methyltransferase GidB
M5005_Spy_0279	3572647	NE	Spy49_0276	6985061	NE	lemA	*			hypothetical protein M5005_Spy_0279
M5005_Spy_0280	3572648	NE	Spy49_0277	6985062	NE	htpX	*			heat shock protein HtpX
M5005_Spy_0281	3572649	NE	Spy49_0278	6985143	NE	-	*			hypothetical protein M5005_Spy_0281
M5005_Spy_0282	3572611	NE	Spy49_0279	6985144	NC	covR				response regulator
M5005_Spy_0283	3572612	NE	Spy49_0280	6985145	E	covS	*			transmembrane histidine kinase
M5005_Spy_0284	3572613	NE	Spy49_0281	6985146	C	nrdR	*		SP_1713	NrdR family transcriptional regulator
M5005_Spy_0285	3572614	E	Spy49_0282	6985147	E	**dnaB**	*	SSA_1806	SP_1712	replicative DNA helicase
M5005_Spy_0286	3572615	E	Spy49_0283	6985148	E	**dnaI**	*	SSA_1805	SP_1711	primosomal protein DnaI
M5005_Spy_0287	3572616	E	Spy49_0284	6985149	E	**engA**	*	SSA_1803	SP_1709	GTP-binding protein EngA
M5005_Spy_0288	3572617	NE	Spy49_0285	6985150	NC	snf	*			SWF/SNF family helicase
M5005_Spy_0289	3572618	NE	Spy49_0286	6985151	NE	-	*			hypothetical protein M5005_Spy_0289
M5005_Spy_0290	3572619	E	Spy49_0287	6985152	E	**murC**	*	SSA_1800	SP_1521	UDP-N-acetylmuramate--L-alanine ligase
M5005_Spy_0291	3572620	NE	Spy49_0288	6985153	NE	-	*			acetyltransferase
M5005_Spy_0292	3572621	C	Spy49_0289	6985154	NC	-	*		SP_1518	aminodeoxychorismate lyase
M5005_Spy_0293	3572622	NE	Spy49_0290	6985155	NC	greA	*			transcription elongation factor GreA
M5005_Spy_0294c	3572623	NC				-				transposase

M5005_Spy_0295c	3572624	E	Spy49_0291	6985156	E	**oxaA**	*		SP_1975	OxaA-like protein precursor
M5005_Spy_0296c	3572625	NE	Spy49_0292c	6985157	NE	-	*			acylphosphatase
M5005_Spy_0297c	3572626	NE				-				transposase
M5005_Spy_0298c	3572627	NE				-				transposase
M5005_Spy_0299	3572628	NE	Spy49_0293	6985158	NE	-	*			23S rRNA methyltransferase
M5005_Spy_0300	3572629	NE	Spy49_0294	6985159	NE	-	*			HAD superfamily hydrolase
M5005_Spy_0301	3572591	NE	Spy49_0295	6985160	NC	-	*			hypothetical protein M5005_Spy_0301
M5005_Spy_0302	3572592	C	Spy49_0296	6985161	NC	-				hypothetical protein M5005_Spy_0302
M5005_Spy_0303	3572593	E	Spy49_0297	6985162	E	**glr**	*	SSA_1784	SP_1881	glutamate racemase
M5005_Spy_0304	3572594	NE	Spy49_0298	6985163	NE	-	*			deoxyribonucleotide triphosphate pyrophosphatase/unknown domain fusion protein
M5005_Spy_0305	3572595	NE	Spy49_0299	6985164	NE	-	*			phosphoesterase
M5005_Spy_0306	3572596	NE	Spy49_0300	6985165	NE	-	*			hypothetical protein M5005_Spy_0306
M5005_Spy_0307	3572597	NE	Spy49_0301	6985166	NE	xerD	*			site-specific tyrosine recombinase XerD
M5005_Spy_0308	3572598	NE	Spy49_0302	6985167	NE	scpA	*			segregation and condensation protein A
M5005_Spy_0309	3572599	NE	Spy49_0303	6985168	NE	scpB	*			segregation and condensation protein B
M5005_Spy_0310	3572600	NE	Spy49_0304	6985169	NE	rluB	*			ribosomal large subunit pseudouridine synthase B
M5005_Spy_0311	3572601	NE	Spy49_0305	6985170	NE	-				hypothetical protein M5005_Spy_0311
M5005_Spy_0312	3572602	NE	Spy49_0306	6985171	NE	-	*			23S rRNA methyltransferase
M5005_Spy_0313	3572603	C	Spy49_0307	6985172	NE	-	*			riboflavin transporter
M5005_Spy_0314	3572604	NE	Spy49_0308	6985173	NE	-	*			phosphatidylglycerophosphatase B
M5005_Spy_0315	3572605	NE	Spy49_0309	6985174	NE	-	*			Fe-S oxidoreductase
M5005_Spy_0316	3572606	NE	Spy49_0310	6985175	NE	-	*			SAM-dependent methyltransferase
M5005_Spy_0317	3572607	NE	Spy49_0311	6985176	NE	hlyX	*			hemolysin
M5005_Spy_0318	3572608	NE	Spy49_0312	6985177	NE	pflC	*			pyruvate formate-lyase activating enzyme
M5005_Spy_0319	3572609	NC	Spy49_0313	6985178	E	ppaC	*	SSA_1748	SP_1534	manganese-dependent inorganic pyrophosphatase
M5005_Spy_0320	3572610	NE	Spy49_0314	6985179	NE	-	*			hypothetical protein M5005_Spy_0320
M5005_Spy_0321c	3572572	NE	Spy49_0315	6985180	NE	fhuG	*			ferrichrome transporter permease
M5005_Spy_0322c	3572573	NE	Spy49_0316	6985181	NE	fhuB	*			ferrichrome transporter permease
M5005_Spy_0323c	3572574	NE	Spy49_0317c	6985182	NE	fhuD	*			ferrichrome-binding protein
M5005_Spy_0324c	3572575	NE	Spy49_0318	6985183	NE	fhuA	*			ferrichrome ABC transporter ATP-binding protein
M5005_Spy_0325c	3572576	E	Spy49_0319c	6985184	E	**murE**	*	SSA_1739	SP_1530	UDP-N-acetylmuramoylalanyl-D-glutamate--L-lysine ligase
M5005_Spy_0326	3572577	E	Spy49_0320	6985185	E	**-**	*	SSA_1738	SP_1529	export protein for polysaccharides and teichoic acids
M5005_Spy_0327	3572578	NE	Spy49_0322	6985186	NE	upp	*			uracil phosphoribosyltransferase
M5005_Spy_0328	3572579	C	Spy49_0323	6985187	E	clpP	*			ATP-dependent Clp protease proteolytic subunit
M5005_Spy_0329	3572580	NE	Spy49_0325	6985188	NE	-				hypothetical protein M5005_Spy_0329
M5005_Spy_0330	3572581	E	Spy49_0326	6985189	E	**tmk**	*	SSA_1722	SP_0935	thymidylate kinase
M5005_Spy_0331	3572582	E	Spy49_0327	6985190	E	**dnaX**	*	SSA_1721		DNA polymerase III subunit delta\'
M5005_Spy_0332	3572583	NE				-				tpl protein
M5005_Spy_0333	3572584	NC	Spy49_0328	6985191	NC	-				signal peptidase-like protein
M5005_Spy_0334	3572585	NC	Spy49_0329	6985192	C	-	*		SP_0937	DNA replication intiation control protein YabA
M5005_Spy_0335	3572586	NE	Spy49_0330	6985193	C	-	*			corrin/porphyrin methyltransferase
M5005_Spy_0336	3572587	NE	Spy49_0331	6985194	NE	-	*			hypothetical protein M5005_Spy_0336
M5005_Spy_0337c	3572588	NE	Spy49_0332c	6985195	NE	cutC	*			copper homeostasis protein
M5005_Spy_0338	3572589	NE	Spy49_0333	6985196	NE	-	*			arsenate reductase
M5005_Spy_0339c	3572590	NC	Spy49_0334c	6985197	NE	exoA	*			exodeoxyribonuclease III
M5005_Spy_0340	3572552	NE	Spy49_0335	6985198	NE	lctO	*			L-lactate oxidase
M5005_Spy_0341	3572553	NE	Spy49_0336	6985199	NE	prtS	*			lactocepin
M5005_Spy_0343	3572554	NE				-				hypothetical protein M5005_Spy_0343
M5005_Spy_0344	3572555	NE	Spy49_0337	6985200	NE	-	*			permease
M5005_Spy_0345	3572556	E	Spy49_0338	6985201	E	**metG**	*	SSA_1703	SP_0788	methionyl-tRNA synthetase
M5005_Spy_0346	3572557	NC				-				hypothetical protein M5005_Spy_0346
M5005_Spy_0347	3572558	NE	Spy49_0339	6985202	NE	nrdF	*			ribonucleotide-diphosphate reductase subunit beta
M5005_Spy_0348	3572559	NE	Spy49_0340	6985203	NE	nrdI	*			ribonucleotide reductase stimulatory protein
M5005_Spy_0349	3572560	NE	Spy49_0341	6985204	NE	nrdE.1	*			ribonucleotide-diphosphate reductase subunit alpha
M5005_Spy_0350	3572561	NC				-				hypothetical protein M5005_Spy_0350
M5005_Spy_0351c	3572562	NE	Spy49_0342c	6985205	NE	spyA	*			C3 family ADP-ribosyltransferase
M5005_Spy_0352	3572563	NE	Spy49_0343	6985206	NC	-				hypothetical protein M5005_Spy_0352
M5005_Spy_0353	3572564	NE	Spy49_0344	6985207	NC	-				hypothetical protein M5005_Spy_0353
M5005_Spy_0354c	3572565	NE	Spy49_0345c	6985208	NE	-				hypothetical protein M5005_Spy_0354
M5005_Spy_0355	3572566	NE	Spy49_0346c	6985209	NE	-				hypothetical protein M5005_Spy_0355
M5005_Spy_0356c	3572567	NE	Spy49_0347	6985210	NE	speJ				exotoxin type J
M5005_Spy_0357c	3572568	NE	Spy49_0348	6985211	NE	-				hypothetical protein M5005_Spy_0357
M5005_Spy_0358	3572569	NE	Spy49_0349c	6985212	NE	-				hypothetical protein M5005_Spy_0358
			Spy49_0350c	6985213	NE					
			Spy49_0351c	6985214	NE					
			Spy49_0352c	6985215	NE					
			Spy49_0353c	6985216	NE					
			Spy49_0354c	6985217	NC					
			Spy49_0355c	6985218	NE					
			Spy49_0356c	6985219	NE					
			Spy49_0357c	6985220	NE					
			Spy49_0358c	6985221	NE					
			Spy49_0359c	6985222	NE					
			Spy49_0360c	6985303	NE					
			Spy49_0361c	6985304	NE					
			Spy49_0362c	6985305	NE					
			Spy49_0363c	6985306	NE					
			Spy49_0364c	6985307	NC					
			Spy49_0365c	6985308	NE					
			Spy49_0366c	6985309	NE					
			Spy49_0367c	6985310	NE					
			Spy49_0368c	6985311	NE					
			Spy49_0369	6985312	E					
			Spy49_0370	6985313	NE					
			Spy49_0371	6985314	NE					
M5005_Spy_0359	3572570	NE	Spy49_0372	6985315	NE	fabG	*			3-ketoacyl-ACP reductase
M5005_Spy_0360	3572571	NE	Spy49_0373	6985316	NE	-	*			NAD-dependent oxidoreductase
M5005_Spy_0361	3572533	NE	Spy49_0374	6985317	NE	glpT	*			phosphoglycerate transporter protein
M5005_Spy_0362	3572534	E	Spy49_0375	6985318	E	**glmU**	*	SSA_1642	SP_0988	bifunctional N-acetylglucosamine-1-phosphate uridyltransferase/glucosamine-1-phosphate acetyltransfe...
M5005_Spy_0363	3572535	NE	Spy49_0376	6985319	NE	-	*			phosphohydrolase
M5005_Spy_0364	3572536	NC	Spy49_0377	6985320	NE	-				hypothetical protein M5005_Spy_0364
M5005_Spy_0365	3572537	E	Spy49_0378	6985321	NC	pfs	*		SP_0991	5\'-methylthioadenosine/S-adenosylhomocysteine nucleosidase
M5005_Spy_0366	3572538	NE	Spy49_0379	6985322	NE	-				hypothetical protein M5005_Spy_0366
M5005_Spy_0367c	3572539	NC	Spy49_0380c	6985323	NC	mtsR	*			iron-dependent repressor
M5005_Spy_0368	3572540	NE	Spy49_0381	6985324	NE	mtsA	*			manganese-binding protein
M5005_Spy_0369	3572541	NE	Spy49_0382	6985325	NE	mtsB	*			manganese transporter ATP-binding protein
M5005_Spy_0370	3572542	NE	Spy49_0383	6985326	NE	mtsC	*			manganese transporter membrane protein
M5005_Spy_0371c	3572543	NE	Spy49_0384c	6985327	NE	cypB	*			peptidyl-prolyl cis-trans isomerase
M5005_Spy_0372	3572544	E	Spy49_0385	6985328	E	**ftsK**	*		SP_0878	cell division protein
M5005_Spy_0373c	3572545	NE	Spy49_0386c	6985329	NE	-				hypothetical protein M5005_Spy_0373
M5005_Spy_0374	3572546	NC	Spy49_0387	6985330	NC	rplK	*			50S ribosomal protein L11

M5005_Spy_0375	3572547	E	Spy49_0388	6985331	E	**rplA**	*	SSA_1622		50S ribosomal protein L1
M5005_Spy_0376	3572548	NE				-				transposase
M5005_Spy_0377	3572549	E	Spy49_0390	6985332	E	**pyrH**	*	SSA_1620	SP_0944	uridylate kinase
M5005_Spy_0380	3572513	E	Spy49_0391	6985333	E	**frr**	*	SSA_1619	SP_0945	ribosome recycling factor
M5005_Spy_0381	3572514	NE	Spy49_0392	6985334	NE	-	*			S1 RNA-binding domain-containing protein
M5005_Spy_0382	3572515	NE	Spy49_0394	6985335	NE	msrA.2	*			methionine sulfoxide reductase A
M5005_Spy_0383	3572516	NE	Spy49_0395	6985336	NE	-				hypothetical protein M5005_Spy_0383
M5005_Spy_0384	3572517	NE	Spy49_0396	6985337	NE	-	*			surface antigen
M5005_Spy_0385	3572518	NE	Spy49_0398	6985338	NE	-				hypothetical protein M5005_Spy_0385
M5005_Spy_0386	3572519	NE	Spy49_0399	6985339	NE	phoH	*			phoH protein
M5005_Spy_0387	3572520	NE	Spy49_0400	6985340	NE	-	*			uracil DNA glycosylase
M5005_Spy_0388	3572521	E	Spy49_0401	6985341	E	**-**	*		SP_0967	metalloprotease
M5005_Spy_0389	3572522	E	Spy49_0402	6985342	E	**dgk**	*		SP_0968	diacylglycerol kinase
M5005_Spy_0390	3572523	C	Spy49_0403	6985343	E	era	*			GTP-binding protein Era
M5005_Spy_0391	3572524	NE	Spy49_0404	6985344	NE	-	*			phosphohydrolase
M5005_Spy_0392c	3572525	NE	Spy49_0405c	6985345	NE	-				hypothetical protein M5005_Spy_0392
M5005_Spy_0393	3572526	NE	Spy49_0406	6985346	NE	-				hypothetical protein M5005_Spy_0393
M5005_Spy_0394	3572527	NE				-				hypothetical protein M5005_Spy_0394
M5005_Spy_0395c	3572528	NE				-				transposase
M5005_Spy_0396c	3572529	NC	Spy49_0407c	6985347	NC	-				transposase
M5005_Spy_0397c	3572530	NE	Spy49_0408c	6985348	NE	-				transposase
M5005_Spy_0398	3572531	NE	Spy49_0409	6985349	NE	-				bacteriocin
M5005_Spy_0399	3572532	NE	Spy49_0410	6985350	NE	-				hypothetical protein M5005_Spy_0399
M5005_Spy_0400	3572494	NE	Spy49_0411	6985351	NE	silD				hypothetical protein M5005_Spy_0400
M5005_Spy_0401c	3572495	NC	Spy49_0412	6985352	NC	-				hypothetical protein M5005_Spy_0401
M5005_Spy_0402	3572496	NE	Spy49_0413	6985353	NE	-				hypothetical protein M5005_Spy_0402
M5005_Spy_0403	3572497	NE	Spy49_0414c	6985354	NE	-				hypothetical protein M5005_Spy_0403
M5005_Spy_0404c	3572498	NE				-				hypothetical protein M5005_Spy_0404
M5005_Spy_0405	3572499	NC				-				hypothetical protein M5005_Spy_0405
M5005_Spy_0406c	3572500	NE				-				hypothetical protein M5005_Spy_0406
M5005_Spy_0407	3572501	NE	Spy49_0415	6985355	NE	mutR	*			transcriptional regulator
M5005_Spy_0408	3572502	NE	Spy49_0416	6985356	NE	fpg	*			formamidopyrimidine-DNA glycosylase
M5005_Spy_0409	3572503	E	Spy49_0417	6985357	E	**coaE**	*	SSA_1606	SP_0971	dephospho-CoA kinase
M5005_Spy_0410	3572504	NE	Spy49_0418	6985358	NC	-	*			ATPase
M5005_Spy_0411	3572505	NE	Spy49_0419	6985359	NE					multidrug resistance protein B
M5005_Spy_0412	3572506	NC	Spy49_0420	6985360	NC	rpmG				50S ribosomal protein L33
M5005_Spy_0413	3572507	E	Spy49_0421	6985361	NC	secG		SSA_1604		preprotein translocase subunit SecG
M5005_Spy_0414	3572508	NE	Spy49_0422	6985362	NC	vacB	*			exoribonuclease II
M5005_Spy_0415	3572509	NC	Spy49_0423	6985363	NC	smpB	*			SsrA-binding protein
M5005_Spy_0416	3572510	NE	Spy49_0424	6985364	NE	-	*			glutaminyl-peptide cyclotransferase
M5005_Spy_0417c	3572511	NE	Spy49_0425c	6985365	NE	pcp	*			pyrrolidone-carboxylate peptidase
M5005_Spy_0418c	3572512	NE	Spy49_0426c	6985366	NE	-	*			permease
M5005_Spy_0419c	3572474	NE	Spy49_0427c	6985367	NE	-	*			permease
M5005_Spy_0420c	3572475	NE	Spy49_0428c	6985368	NE	-	*			glucosyltransferase
M5005_Spy_0421c	3572476	NE	Spy49_0429c	6985369	NE	gloA	*			lactoylglutathione lyase
M5005_Spy_0422c	3572477	NE	Spy49_0430c	6985370	NE	-	*			NAD(P)H-dependent quinone reductase
M5005_Spy_0423c	3572478	NE	Spy49_0431c	6985371	NE	pepQ	*			Xaa-Pro dipeptidase
M5005_Spy_0424	3572479	NC	Spy49_0432	6985372	E	ccpA	*			catabolite control protein A
M5005_Spy_0425	3572480	C	Spy49_0433	6985373	E	-	*		SP_1075	glycosyltransferase
M5005_Spy_0426	3572481	E	Spy49_0434	6985374	E	**-**	*			1,2-diacylglycerol 3-glucosyltransferase
M5005_Spy_0427	3572482	E	Spy49_0435	6985375	E	**thrS**	*	SSA_1571	SP_1631	threonyl-tRNA synthetase
M5005_Spy_0428	3572483	NE	Spy49_0436	6985376	NE	drrA/tagH	*			daunorubicin resistance ATP-binding protein
M5005_Spy_0429	3572484	NE	Spy49_0437	6985377	NE	-	*			daunorubicin resistance transmembrane protein
M5005_Spy_0430	3572485	NE	Spy49_0438	6985378	NE	-	*			ABC transporter permease
M5005_Spy_0431	3572486	NC	Spy49_0439	6985379	NC	-				dihydroxyacetone kinase
M5005_Spy_0432	3572487	NE	Spy49_0440	6985380	NE	-	*			acetyl-CoA acetyltransferase
M5005_Spy_0433	3572488	NE	Spy49_0441	6985381	NE	-	*			long-chain-fatty-acid--CoA ligase
M5005_Spy_0434	3572489	NE	Spy49_0442	6985382	NE	-	*			hypothetical protein M5005_Spy_0434
M5005_Spy_0435	3572490	E	Spy49_0444	6985463	E	**vicR**	*	SSA_1565	SP_1227	two-component response regulator
M5005_Spy_0436	3572491	NE	Spy49_0445	6985464	NE	vicK	*			two-component sensor histidine kinase
M5005_Spy_0437	3572492	NE	Spy49_0446	6985465	NE	vicX	*			Zn-dependent hydrolase
M5005_Spy_0438	3572493	C	Spy49_0447	6985466	NC	rnc	*		SP_1248	ribonuclease III
M5005_Spy_0439	3572455	NE	Spy49_0448	6985467	NC	smc	*			chromosome partition protein
M5005_Spy_0440c	3572456	NE	Spy49_0449c	6985468	NE	-	*			transcriptional regulator
M5005_Spy_0441	3572457	NE	Spy49_0450	6985469	NE	aroE	*			shikimate 5-dehydrogenase
M5005_Spy_0442	3572458	NE	Spy49_0451	6985470	NE	-	*			hypothetical protein M5005_Spy_0442
M5005_Spy_0443	3572459	NE	Spy49_0453	6985471	NC	-	*			hypothetical protein M5005_Spy_0443
M5005_Spy_0444	3572460	NE	Spy49_0454	6985472	NE	-	*			hypothetical protein M5005_Spy_0444
M5005_Spy_0445	3572461	NE	Spy49_0455	6985473	NE	metK1	*			S-adenosylmethionine synthetase
M5005_Spy_0446	3572462	NE	Spy49_0456	6985474	NE	-	*			hypothetical protein M5005_Spy_0446
M5005_Spy_0447	3572463	NE	Spy49_0457	6985475	NE	-	*			cell wall biosynthesis glycosyltransferase
M5005_Spy_0448	3572464	NC	Spy49_0458	6985476	NE	-				hypothetical protein M5005_Spy_0448
M5005_Spy_0449	3572465	NE	Spy49_0459	6985477	NE	hasB.2				UDP-glucose 6-dehydrogenase
M5005_Spy_0450	3572466	NE	Spy49_0460	6985478	NE	mefE				macrolide-efflux protein
M5005_Spy_0451c	3572467	NE	Spy49_0461c	6985479	NE	-				transcriptional regulator
M5005_Spy_0452c	3572468	NE	Spy49_0462c	6985480	NE	-	*			chromosome segregation ATPase
M5005_Spy_0453c	3572469	NE	Spy49_0463c	6985481	NE	-	*			chromosome segregation ATPase
M5005_Spy_0454c	3572470	NE	Spy49_0464c	6985482	NE	-				hypothetical protein M5005_Spy_0454
M5005_Spy_0455c	3572471	NE	Spy49_0465	6985483	NC	-	*			hypothetical protein M5005_Spy_0455
M5005_Spy_0456	3572472	NC	Spy49_0466	6985484	NE	-	*			plasmid stabilization system antitoxin protein
M5005_Spy_0457	3572473	NE				-	*			plasmid stabilization system protein
M5005_Spy_0458	3572435	NE	Spy49_0467	6985485	NE	-				hypothetical protein M5005_Spy_0458
M5005_Spy_0459	3572436	NE	Spy49_0468	6985486	NE	-				portal protein
M5005_Spy_0460	3572437	NE	Spy49_0469	6985487	NE	-				hypothetical protein M5005_Spy_0460
			Spy49_0470c	6985488	NE					
M5005_Spy_0461	3572438	NE	Spy49_0471	6985489	NE	-				hypothetical protein M5005_Spy_0461
M5005_Spy_0462	3572439	NE	Spy49_0473	6985490	NE	-				asparagine synthetase A
M5005_Spy_0463	3572440	NE	Spy49_0474	6985491	NE	-				hypothetical protein M5005_Spy_0463
M5005_Spy_0464	3572441	NE	Spy49_0475	6985492	NE	mccF				microcin C7 self-immunity protein
M5005_Spy_0465	3572442	NE				-				hypothetical protein M5005_Spy_0465
M5005_Spy_0466	3572443	NE	Spy49_0476	6985493	NE	-				hypothetical protein M5005_Spy_0466
M5005_Spy_0467c	3572444	NE	Spy49_0477c	6985494	NE	-				transposase
M5005_Spy_0468c	3572445	NE	Spy49_0478c	6985495	NE	-				transposase
M5005_Spy_0469	3572446	NE	Spy49_0479	6985496	NE	-				hypothetical protein M5005_Spy_0469
M5005_Spy_0470	3572447	NE	Spy49_0480	6985497	NE	-	*			HAD superfamily hydrolase
M5005_Spy_0471	3572448	NE	Spy49_0481	6985498	NE	-	*			HAD superfamily hydrolase
M5005_Spy_0472	3572449	E	Spy49_0482	6985499	E	**ftsY**	*	SSA_1557	SP_1244	cell division protein
M5005_Spy_0473c	3572450	NE	Spy49_0483c	6985500	NE	-	*			multidrug resistance protein B
M5005_Spy_0474	3572451	NE	Spy49_0484	6985501	NE	licT/bglG	*			BigG family transcription antiterminator
M5005_Spy_0475	3572452	NE	Spy49_0485	6985502	NE	-	*			PTS system beta-glucoside-specific transporter subunit IIABC
M5005_Spy_0476	3572453	NE	Spy49_0487	6985503	NE	bglA	*			6-phospho-beta-glucosidase
M5005_Spy_0477c	3572454	NE	Spy49_0488c	6985504	NE	-	*			hypothetical protein M5005_Spy_0477

M5005_Spy_0478c	3572416	NE	Spy49_0489c	6985505	NE	-	*			hypothetical protein M5005_Spy_0478
M5005_Spy_0479	3572417	NE				-				hypothetical protein M5005_Spy_0479
M5005_Spy_0480	3572419	NE	Spy49_0490	6985506	NE	-	*			transcription accessory protein
M5005_Spy_0481c	3572418	NC				-				hypothetical protein M5005_Spy_0481
M5005_Spy_0482	3572420	NE	Spy49_0491	6985507	NE	-				hypothetical protein M5005_Spy_0482
M5005_Spy_0483	3572421	NE	Spy49_0493	6985508	NE	-				stress-responsive transcriptional regulator
M5005_Spy_0484	3572422	E	Spy49_0494	6985509	E	**ptsK**	*		SP_1413	HPr kinase/phosphorylase
M5005_Spy_0485	3572423	NE	Spy49_0495	6985510	NE	lgt	*			prolipoprotein diacylglyceryl transferase
M5005_Spy_0486	3572424	NE	Spy49_0496	6985511	NE	-	*			hypothetical protein M5005_Spy_0486
M5005_Spy_0487	3572425	NE	Spy49_0497	6985512	NE	-	*			hypothetical protein M5005_Spy_0487
M5005_Spy_0488c	3572426	NE	Spy49_0498	6985513	NE	-				hypothetical protein M5005_Spy_0488
M5005_Spy_0489	3572427	NE	Spy49_0499	6985514	NE	-	*			U32 family peptidase
M5005_Spy_0491	3572429	NE	Spy49_0500	6985515	NE	-	*			U32 family peptidase
M5005_Spy_0492c	3572430	NC				-				hypothetical protein M5005_Spy_0492
M5005_Spy_0493	3572431	NE	Spy49_0501	6985516	NE	-	*			hypothetical protein M5005_Spy_0493
M5005_Spy_0494c	3572432	NC	Spy49_0502c	6985517	NC	-				hypothetical protein M5005_Spy_0494
M5005_Spy_0495c	3572433	E	Spy49_0503c	6985518	E	**lysS**	*	SSA_1529	SP_0713	lysyl-tRNA synthetase
M5005_Spy_0496	3572434	NE	Spy49_0504	6985519	NE	-	*			HAD superfamily hydrolase
M5005_Spy_0497c	3572396	NE	Spy49_0505c	6985520	NE	-				phosphoglycerate mutase
M5005_Spy_0498c	3572397	NE	Spy49_0506c	6985521	NE	-	*			transcriptional regulator
M5005_Spy_0499c	3572398	NE	Spy49_0507c	6985522	NE	-	*			thiamine transporter
M5005_Spy_0500c	3572399	C	Spy49_0508c	6985523	NE	-	*			N-acetylmuramoyl-L-alanine amidase
M5005_Spy_0501	3572400	NE	Spy49_0509	6985524	NE	-	*			hypothetical protein M5005_Spy_0501
M5005_Spy_0502	3572401	NE	Spy49_0510	6985525	NE	-	*			hypothetical protein M5005_Spy_0502
M5005_Spy_0503c	3572402	NE	Spy49_0511c	6985526	NE	-	*			glutathione peroxidase
M5005_Spy_0504c	3572403	NE	Spy49_0512c	6985527	NE	pepF	*			oligoendopeptidase F
M5005_Spy_0505	3572404	NE	Spy49_0513	6985528	NE	ppc	*			phosphoenolpyruvate carboxylase
M5005_Spy_0506	3572405	E	Spy49_0514	6985529	E	**ftsW**	*	SSA_1522	SP_0803	cell division protein
M5005_Spy_0507c	3572406	NC				-				hypothetical protein M5005_Spy_0507
M5005_Spy_0508	3572407	E	Spy49_0515	6985530	NC	tuf	*	SSA_1520	SP_1489	elongation factor Tu
M5005_Spy_0509	3572408	C	Spy49_0516	6985531	E	tpiA	*	SSA_0859	SP_1574	triosephosphate isomerase
M5005_Spy_0510c	3572409	E	Spy49_0517c	6985532	E	**murN**	*		SP_0616	factor essential for expression of methicillin resistance
M5005_Spy_0511c	3572410	E	Spy49_0518c	6985533	E	**murM**	*	SSA_0862		UDP-N-acetylmuramoylpentapeptide-lysine N(6)-alanyltransferase/UDP-N-acetylmuramoylpentapeptide-lysi...
M5005_Spy_0512c	3572411	NE	Spy49_0519c	6985534	NE	-	*			HAD superfamily hydrolase
M5005_Spy_0513c	3572412	NC				-				hypothetical protein M5005_Spy_0513
M5005_Spy_0514c	3572413	NE	Spy49_0520c	6985535	NE	-	*			dGTP triphosphohydrolase
M5005_Spy_0515	3572414	NE	Spy49_0521	6985536	NE	-	*			hypothetical protein M5005_Spy_0515
M5005_Spy_0516	3572415	E	Spy49_0522	6985537	E	**pacL**	*		SP_1551	calcium-transporting ATPase
M5005_Spy_0517c	3572377	NE	Spy49_0523c	6985538	NE	regR	*			LacI family transcriptional regulator
M5005_Spy_0518c	3572378	NE	Spy49_0524c	6985539	NE	-	*			oligohyaluronate lyase
M5005_Spy_0519c	3572379	NE	Spy49_0525c	6985540	NC	agaD	*			PTS system N-acetylgalactosamine-specific transporter subunit IID
M5005_Spy_0520c	3572380	NE	Spy49_0526c	6985541	NE	agaW	*			PTS system N-acetylgalactosamine-specific transporter subunit IIC
M5005_Spy_0521c	3572381	NE	Spy49_0527c	6985542	NE	agaV	*			PTS system N-acetylgalactosamine-specific transporter subunit IIB
M5005_Spy_0522c	3572382	NE	Spy49_0528c	6985543	NE	ugl				unsaturated glucuronyl hydrolase
M5005_Spy_0523	3572383	NE	Spy49_0529c	6985544	NE	agaF	*			hypothetical protein M5005_Spy_0523
M5005_Spy_0524	3572384	NE	Spy49_0530	6985545	NE	idnO	*			gluconate 5-dehydrogenase
M5005_Spy_0525	3572385	NE	Spy49_0531	6985546	NE	-	*			hypothetical protein M5005_Spy_0525
M5005_Spy_0526	3572386	NE	Spy49_0532	6985547	NE	kdgK	*			2-dehydro-3-deoxygluconokinase
M5005_Spy_0527	3572387	NE	Spy49_0533	6985548	NE	kgdA	*			keto-hydroxyglutarate-aldolase/keto-deoxy-phosphogluconate aldolase
M5005_Spy_0528	3572388	NE	Spy49_0534	6985549	NE	-	*			beta-phosphoglucomutase/glucose-1-phosphate phosphodismutase
M5005_Spy_0529	3572389	NE	Spy49_0535	6985550	NE	-				(Fe-S)-binding protein
M5005_Spy_0530	3572390	E	Spy49_0536	6985551	E	**prfB**	*	SSA_0869	SP_0755	peptide chain release factor 2
M5005_Spy_0531	3572391	NC	Spy49_0537	6985552	NC	ftsE	*			cell division ATP-binding protein
M5005_Spy_0532	3572392	C	Spy49_0538	6985553	E	ftsX	*	SSA_0871	SP_0757	cell division protein
M5005_Spy_0533c	3572393	NE	Spy49_0539c	6985554	NE	-	*			hydroxyacylglutathione hydrolase
M5005_Spy_0534	3572394	NE	Spy49_0540	6985555	NE	-				acetoin reductase
M5005_Spy_0535	3572395	NC				-				acetoin dehydrogenase, partial
M5005_Spy_0536	3572358	NE	Spy49_0541	6985556	NE	dinG	*			bifunctional ATP-dependent DNA helicase/DNA polymerase III subunit epsilon
M5005_Spy_0537	3572359	NE	Spy49_0543	6985557	NE	aspC	*			aspartate aminotransferase
M5005_Spy_0538	3572360	E	Spy49_0544	6985558	E	**asnC**	*		SP_1542	asparaginyl-tRNA synthetase
M5005_Spy_0539	3572361	NE	Spy49_0545	6985559	E	-	*			hypothetical protein M5005_Spy_0539
M5005_Spy_0540	3572362	NE	Spy49_0546	6985560	NE	-	*			transporter
M5005_Spy_0541	3572363	NE	Spy49_0547	6985561	NE	-	*			hypothetical protein M5005_Spy_0541
M5005_Spy_0542	3572364	NE	Spy49_0548	6985562	NE	pepD				dipeptidase
M5005_Spy_0543	3572365	NE	Spy49_0549	6985563	NE	adcA	*			high-affinity zinc uptake system protein znuA
M5005_Spy_0544	3572366	NE	Spy49_0550	6985564	NE	-	*			GntR family transcriptional regulator
M5005_Spy_0545	3572367	NE	Spy49_0551	6985565	NE	agaS	*			galactosamine-6-phosphate deaminase
M5005_Spy_0546c	3572368	C	Spy49_0552c	6985566	NC	rpmE2			SP_1299	50S ribosomal protein L31
M5005_Spy_0547c	3572369	NC	Spy49_0554c	6985567	NE	-	*			phosphoesterase, DHH family protein
M5005_Spy_0548	3572370	C	Spy49_0555	6985568	NE	flaV	*		SP_1297	flavodoxin
M5005_Spy_0549	3572371	NE	Spy49_0556	6985569	NC	-				hypothetical protein M5005_Spy_0549
M5005_Spy_0550	3572372	NE	Spy49_0557	6985570	C	-	*			chloride channel protein
M5005_Spy_0551	3572373	NC	Spy49_0558	6985571	NC	rplS	*			50S ribosomal protein L19
M5005_Spy_0552	3572375	NE	Spy49_0559	6985573	NE	-	*			DNA gyrase
M5005_Spy_0553	3572376	E	Spy49_0560	6985574	E	**gyrB**	*	SSA_0878	SP_0806	DNA gyrase subunit B
M5005_Spy_0554	3572339	E	Spy49_0561	6985575	E	**ezrA**	*		SP_0807	septation ring formation regulator EzrA
M5005_Spy_0555c	3572340	NE	Spy49_0562c	6985576	NE	-	*			hypothetical protein M5005_Spy_0555
M5005_Spy_0556	3572341	E	Spy49_0563	6985577	E	**eno**	*	SSA_0886	SP_1128	phosphopyruvate hydratase
M5005_Spy_0557c	3572342	NE	Spy49_0564c	6985578	NE	-				transposase
M5005_Spy_0558c	3572343	NE	Spy49_0565c	6985579	NC	-				transposase
M5005_Spy_0559c	3572344	NE	Spy49_0566c	6985580	NE	-				transcriptional regulator
M5005_Spy_0560c	3572345	NE				-				transcriptional regulator
M5005_Spy_0561	3572346	NE	Spy49_0567	6985581	NC	epf				extracellular matrix binding protein
M5005_Spy_0562	3572347	NE	Spy49_0568	6985582	NE	sagA				streptolysin S
M5005_Spy_0563	3572348	NE	Spy49_0569	6985583	NE	sagB	*			streptolysin S biosynthesis protein
M5005_Spy_0564	3572349	NE	Spy49_0570	6985584	NE	sagC	*			streptolysin S biosynthesis protein
M5005_Spy_0565	3572350	NE	Spy49_0572	6985585	NE	sagD	*			streptolysin S biosynthesis protein
M5005_Spy_0566	3572351	NE	Spy49_0573	6985586	NE	sagE	*			streptolysin S self-immunity protein
M5005_Spy_0567	3572352	NE	Spy49_0574	6985587	NE	sagF	*			streptolysin S biosynthesis protein
M5005_Spy_0568	3572353	NE	Spy49_0575	6985588	C	sagG	*			streptolysin S export ATP-binding protein
M5005_Spy_0569	3572354	NE	Spy49_0576	6985589	NC	sagH	*			streptolysin S export transmembrane protein
M5005_Spy_0570	3572355	NE	Spy49_0577	6985590	C	sagI	*			streptolysin S export transmembrane protein
M5005_Spy_0571	3572356	NE	Spy49_0578	6985591	NE	-	*			hypothetical protein M5005_Spy_0571
M5005_Spy_0572	3572357	NE	Spy49_0579	6985592	NE	-	*			hypothetical protein M5005_Spy_0572
M5005_Spy_0573	3572319	E	Spy49_0580	6985593	E	**ligA**	*	SSA_1484		NAD-dependent DNA ligase LigA
M5005_Spy_0574	3572320	E	Spy49_0581	6985594	E	**-**	*			lipid kinase
M5005_Spy_0575	3572321	NC	Spy49_0582	6985595	NC	atpE				ATP synthase F0F1 subunit C
M5005_Spy_0576	3572322	NC	Spy49_0583	6985596	E	atpB	*	SSA_0783	SP_1513	ATP synthase F0F1 subunit A
M5005_Spy_0577	3572323	E	Spy49_0584	6985597	NC	atpF	*	SSA_0784	SP_1512	ATP synthase F0F1 subunit B
M5005_Spy_0578	3572324	E	Spy49_0585	6985598	E	**atpH**	*	SSA_0785	SP_1511	ATP synthase F0F1 subunit delta
M5005_Spy_0579	3572325	E	Spy49_0586	6985599	E	**atpA**	*	SSA_0786	SP_1510	ATP synthase F0F1 subunit alpha

M5005_Spy_0580	3572326	E	Spy49_0587	6985600	E	**atpG**	*	SSA_0787	SP_1509	ATP synthase F0F1 subunit gamma
M5005_Spy_0581	3572327	E	Spy49_0588	6985601	E	**atpD**	*	SSA_0788	SP_1508	ATP synthase F0F1 subunit beta
M5005_Spy_0582	3572328	NC	Spy49_0589	6985602	NC	atpC	*			ATP synthase F0F1 subunit epsilon
M5005_Spy_0583	3572329	NE	Spy49_0590	6985603	NE	-				hypothetical protein M5005_Spy_0583
M5005_Spy_0584	3572330	NE	Spy49_0591	6985604	NE	murA	*			UDP-N-acetylglucosamine 1-carboxyvinyltransferase
M5005_Spy_0585	3572331	NE				epuA				epuA protein
M5005_Spy_0586	3572332	NE				endA				DNA-entry nuclease
			Spy49_0592	6985605	NE					
M5005_Spy_0587	3572333	E	Spy49_0596	6985608	E	**pheS**	*	SSA_0912	SP_0579	phenylalanyl-tRNA synthetase subunit alpha
M5005_Spy_0588	3572334	E	Spy49_0597	6985609	E	**pheT**	*	SSA_0914	SP_0581	phenylalanyl-tRNA synthetase subunit beta
M5005_Spy_0589	3572335	NE				-	*			salt-stress induced protein
			Spy49_0598	6985610	NE					
M5005_Spy_0590	3572336	NE	Spy49_0599	6985611	NE	-	*			hypothetical protein M5005_Spy_0590
M5005_Spy_0591	3572337	NE	Spy49_0600	6985612	NE	-	*			ABC transporter permease
M5005_Spy_0592	3572338	NE	Spy49_0601	6985613	NE	-	*			ABC transporter ATP-binding protein
M5005_Spy_0593c	3572300	NE	Spy49_0602c	6985614	NE	-	*			neutral zinc metallopeptidase
M5005_Spy_0594	3572301	NC	Spy49_0603	6985615	C	rexB	*	SSA_1452	SP_1151	ATP-dependent nuclease subunit B
M5005_Spy_0595	3572302	C	Spy49_0604	6985616	E	rexA	*		SP_1152	ATP-dependent nuclease subunit A
M5005_Spy_0596	3572303	NE	Spy49_0605	6985617	NE	-	*			arginine-binding protein
M5005_Spy_0597	3572304	E	Spy49_0606	6985618	NC	rpsU				30S ribosomal protein S21
M5005_Spy_0598c	3572305	NE	Spy49_0607c	6985619	NE	mscL	*			large-conductance mechanosensitive channel
M5005_Spy_0599	3572306	E	Spy49_0608	6985620	E	**dnaG**		SSA_0824	SP_1072	DNA primase
M5005_Spy_0600	3572307	E	Spy49_0609	6985621	E	**rpoD**	*	SSA_0825	SP_1073	RNA polymerase sigma factor RpoD
M5005_Spy_0601	3572308	NE	Spy49_0610	6985622	NE	-	*			hypothetical protein M5005_Spy_0601
M5005_Spy_0602	3572309	E	Spy49_0611	6985623	E	rmlD				dTDP-4-dehydrorhamnose reductase
M5005_Spy_0603	3572310	E	Spy49_0612	6985624	E	**rgpAc**	*		SP_1366	alpha-(1,2)-rhamnosyltransferase
M5005_Spy_0604	3572311	E	Spy49_0613	6985625	E	**rgpBc**	*		SP_1365	alpha-L-Rha alpha-1,3-L-rhamnosyltransferase
M5005_Spy_0605	3572312	E	Spy49_0614	6985626	E	**rgpCc**	*			polysaccharide export ABC transporter permease
M5005_Spy_0606	3572313	E	Spy49_0615	6985627	E	**rgpDc**	*			polysaccharide export ATP-binding protein
M5005_Spy_0607	3572314	E	Spy49_0617	6985628	E	**rgpEc**	*			glycosyltransferase
M5005_Spy_0608	3572315	E	Spy49_0618	6985629	E	**rgpFc**	*			alpha-L-Rha alpha-1,3-L-rhamnosyltransferase
M5005_Spy_0609	3572316	NE	Spy49_0619	6985630	NE	-	*			phosphoglycerol transferase
M5005_Spy_0610	3572317	NE	Spy49_0620	6985631	NE	-	*			glycosyltransferase
M5005_Spy_0611	3572318	NE	Spy49_0621	6985632	NE	-	*			hypothetical protein M5005_Spy_0611
M5005_Spy_0612	3572280	C	Spy49_0622	6985633	E	amrA	*	SSA_1738		transcriptional activator
M5005_Spy_0613	3572281	E	Spy49_0623	6985634	E	**-**	*			hypothetical protein M5005_Spy_0613
M5005_Spy_0614	3572282	NE	Spy49_0624	6985635	NE	pepT	*			peptidase T
M5005_Spy_0615	3572283	NE	Spy49_0625	6985636	NE	ebsA	*			pore forming protein
M5005_Spy_0616c	3572284	NE	Spy49_0626c	6985637	NE	-				ferredoxin
M5005_Spy_0617	3572285	NC	Spy49_0627	6985638	NE	-	*			hypothetical protein M5005_Spy_0617
M5005_Spy_0618	3572286	C	Spy49_0628	6985639	NC	cmk	*		SP_1603	cytidylate kinase
M5005_Spy_0619	3572287	C	Spy49_0629	6985640	E	infC	*	SSA_1500	SP_0959	translation initiation factor IF-3
M5005_Spy_0620	3572288	C	Spy49_0630	6985641	NE	rpmI			SP_0960	50S ribosomal protein L35
M5005_Spy_0621	3572289	NC	Spy49_0631	6985642	NC	rplT	*			50S ribosomal protein L20
M5005_Spy_0622c	3572290	E	Spy49_0632c	6985643	E	**-**	*			phosphoglycerol transferase
M5005_Spy_0623	3572291	NE	Spy49_0633	6985644	NE	-	*			methyltransferase
M5005_Spy_0624	3572292	NE	Spy49_0634	6985645	NE	aroD	*			3-dehydroquinate dehydratase
M5005_Spy_0625	3572293	NE	Spy49_0635	6985646	NE	aroF	*			chorismate synthase
M5005_Spy_0626	3572294	NE	Spy49_0636	6985647	NE	-	*			hypothetical protein M5005_Spy_0626
M5005_Spy_0627	3572295	NE	Spy49_0637	6985648	NE	gor	*			glutathione reductase
M5005_Spy_0628c	3572296	NE	Spy49_0638c	6985649	NE	folC.2	*			folylpolyglutamate synthase/dihydrofolate synthase
M5005_Spy_0629c	3572297	NE	Spy49_0639c	6985650	NE	-	*			hypothetical protein M5005_Spy_0629
M5005_Spy_0630	3572298	NE	Spy49_0640	6985651	NE	nifS1	*			cysteine desulfhydrase
M5005_Spy_0631	3572299	NE	Spy49_0641	6985652	NE	thiI	*			thiamine biosynthesis protein ThiI
M5005_Spy_0632	3572261	NE	Spy49_0642	6985653	NE	capA	*			capsule biosynthesis protein
M5005_Spy_0633	3572262	NC	Spy49_0643	6985654	NC	rplU	*			50S ribosomal protein L21
M5005_Spy_0634	3572263	NC	Spy49_0644	6985655	NC	-	*			hypothetical protein M5005_Spy_0634
M5005_Spy_0635	3572264	NC	Spy49_0645	6985656	NC	rpmA	*			50S ribosomal protein L27
M5005_Spy_0636	3572265	NE	Spy49_0646	6985657	NE	-	*			LysR family transcriptional regulator
M5005_Spy_0637	3572266	NE	Spy49_0647	6985658	NE	lsp	*			lipoprotein signal peptidase
M5005_Spy_0638	3572267	NE	Spy49_0648	6985659	NE	-	*			ribosomal large subunit pseudouridine synthase D
M5005_Spy_0639	3572268	NE	Spy49_0650	6985660	NE	pyrR	*			bifunctional pyrimidine regulatory protein PyrR/uracil phosphoribosyltransferase
M5005_Spy_0640	3572269	NE	Spy49_0651	6985661	NE	pyrP	*			uracil permease
M5005_Spy_0641	3572270	NE	Spy49_0652	6985662	NE	pyrB	*			aspartate carbamoyltransferase
M5005_Spy_0642	3572271	NE	Spy49_0653	6985663	NC	carA	*			carbamoyl phosphate synthase small subunit
M5005_Spy_0643	3572272	NE	Spy49_0654	6985664	NE	carB	*			carbamoyl phosphate synthase large subunit
M5005_Spy_0644	3572273	NE	Spy49_0655	6985665	NE	-	*			periplasmic protein of efflux system
M5005_Spy_0645	3572274	NE	Spy49_0656	6985666	NE	-	*			ABC transporter ATP-binding protein
M5005_Spy_0646	3572275	NE	Spy49_0657	6985667	NE	-	*			ABC transporter permease
M5005_Spy_0647	3572276	NE	Spy49_0658	6985668	C	-	*		SP_0994	glycerophosphoryl diester phosphodiesterase
M5005_Spy_0648	3572277	NE	Spy49_0659	6985669	E	rpsP		SSA_1310	SP_0775	30S ribosomal protein S16
M5005_Spy_0649	3572278	NE	Spy49_0660	6985670	NC	-				RNA binding protein
M5005_Spy_0650	3572279	NC	Spy49_0661	6985671	NE	-				hypothetical protein M5005_Spy_0650
M5005_Spy_0651	3572241	NE				-	*			cell surface protein
M5005_Spy_0652	3572242	NE	Spy49_0662	6985672	NE	-	*			hypothetical protein M5005_Spy_0652
M5005_Spy_0653c	3572243	NE	Spy49_0663c	6985673	NE	czcD	*			cobalt-zinc-cadmium resistance protein
M5005_Spy_0654	3572244	NE	Spy49_0664	6985674	NE	-	*			TetR family transcriptional regulator
M5005_Spy_0655	3572245	NE	Spy49_0665	6985675	NC	rimM	*			16S rRNA-processing protein RimM
M5005_Spy_0656	3572246	E	Spy49_0667	6985676	E	**trmD**	*	SSA_1302	SP_0779	tRNA (guanine-N(1)-)-methyltransferase
M5005_Spy_0657	3572247	NE	Spy49_0668	6985677	NE	trxB	*			thioredoxin reductase
M5005_Spy_0658	3572248	NE	Spy49_0669	6985678	NE	-	*			regulatory protein
M5005_Spy_0659	3572249	NE	Spy49_0671	6985679	NE	apbA	*			2-dehydropantoate 2-reductase
M5005_Spy_0660	3572250	NE	Spy49_0672	6985680	NE	fruR	*			fructose repressor
M5005_Spy_0661	3572251	NE	Spy49_0673	6985681	NE	fruB	*			1-phosphofructokinase
M5005_Spy_0662	3572252	NE	Spy49_0674	6985682	NE	fruA	*			PTS system fructose-specific transporter subunit IIABC
M5005_Spy_0663	3572253	NE	Spy49_0675	6985683	NE	mur1.1	*			autolysin
M5005_Spy_0664	3572254	NE	Spy49_0676	6985684	NE	mur1.2	*			autolysin
M5005_Spy_0665c	3572255	NE	Spy49_0677c	6985685	NE	-				transposase
M5005_Spy_0666c	3572256	NC	Spy49_0679	6985687	NE	-				hypothetical protein M5005_Spy_0666
M5005_Spy_0667c	3572257	NC				-				exotoxin type C
M5005_Spy_0668c	3572258	NE				mac				IgG-degrading protease
M5005_Spy_0669	3572259	NC				-				phage protein
M5005_Spy_0670	3572260	NE				-				nucleoside diphosphate kinase
M5005_Spy_0671	3572222	NE	Spy49_0680	6985688	NE	-	*			transposase
M5005_Spy_0672	3572223	NE	Spy49_0681	6985689	NE	-	*			degV family protein
M5005_Spy_0673	3572224	E	Spy49_0682	6985690	E	**papS**	*	SSA_1086	SP_1554	tRNA CCA-pyrophosphorylase
M5005_Spy_0674	3572225	NE	Spy49_0683	6985691	NE	-	*			ABC transporter ATP-binding protein
M5005_Spy_0675	3572226	NC				-				hypothetical protein M5005_Spy_0675
M5005_Spy_0676	3572227	NE				-				hypothetical protein M5005_Spy_0676
M5005_Spy_0677	3572228	NE	Spy49_0684	6985692	NE	fms	*			peptide deformylase
M5005_Spy_0678c	3572229	NE	Spy49_0686c	6985693	NE	-	*			5\'-nucleotidase

M5005_Spy_0679	3572230	NE	Spy49_0687	6985694	NE	-	*			GTP pyrophosphokinase
M5005_Spy_0680	3572231	NE	Spy49_0688	6985695	NE	-	*			two-component response regulator
M5005_Spy_0681	3572232	NE	Spy49_0689	6985696	NE	-	*			two-component system histidine kinase
M5005_Spy_0682	3572233	E	Spy49_0690	6985697	E	**mvaK1**	*	SSA_0333	SP_0381	mevalonate kinase
M5005_Spy_0683	3572234	E	Spy49_0691	6985698	E	**mvaD**	*	SSA_0334	SP_0382	diphosphomevalonate decarboxylase
M5005_Spy_0684	3572235	NC	Spy49_0692	6985699	E	mvaK2	*	SSA_0335	SP_0383	phosphomevalonate kinase
M5005_Spy_0685	3572236	E	Spy49_0693	6985700	E	**-**	*	SSA_0336	SP_0384	isopentenyl pyrophosphate isomerase
M5005_Spy_0686c	3572237	C	Spy49_0694c	6985701	E	mvaS1	*	SSA_0337	SP_1726	3-hydroxy-3-methylglutaryl-CoA reductase
M5005_Spy_0687c	3572238	E	Spy49_0695c	6985702	E	**mvaS2**	*	SSA_0338	SP_1727	hydroxymethylglutaryl-CoA synthase
M5005_Spy_0688	3572239	NC	Spy49_0696	6985703	C	thyA	*	SSA_1091	SP_0669	thymidylate synthase
M5005_Spy_0689	3572240	E	Spy49_0697	6985704	E	**dyr**	*	SSA_1092	SP_1571	dihydrofolate reductase
M5005_Spy_0690	3572202	NE	Spy49_0698	6985705	NE	-				hypothetical protein M5005_Spy_0690
M5005_Spy_0691	3572203	NE	Spy49_0700	6985706	NC	clpX	*			ATP-dependent protease ATP-binding subunit ClpX
M5005_Spy_0692	3572204	E	Spy49_0701	6985707	E	**engB**	*	SSA_1094	SP_1568	ribosome biogenesis GTP-binding protein YsxC
M5005_Spy_0693	3572205	NE	Spy49_0702	6985708	NE	-	*			hypothetical protein M5005_Spy_0693
M5005_Spy_0694c	3572206	NE	Spy49_0703c	6985709	NE	clpL	*			ATP-dependent protease ATP-binding subunit
M5005_Spy_0695	3572207	C	Spy49_0704	6985710	NC	rpiA	*		SP_0828	ribose-5-phosphate isomerase A
M5005_Spy_0696	3572208	NE	Spy49_0705	6985711	NE	deoB	*			phosphopentomutase
M5005_Spy_0697	3572209	NE	Spy49_0706	6985712	NE	arsC	*			arsenate reductase
M5005_Spy_0698	3572210	NC	Spy49_0707	6985713	NE	punA	*			purine nucleoside phosphorylase
M5005_Spy_0699	3572211	NE	Spy49_0708	6985714	NE	deoD	*			purine nucleoside phosphorylase
M5005_Spy_0700	3572212	NE	Spy49_0709	6985715	NE	cpsX				LytR family transcriptional regulator
M5005_Spy_0701c	3572213	NE	Spy49_0710c	6985716	NE	cpsY	*			LysR family transcriptional regulator
M5005_Spy_0702	3572214	NE	Spy49_0711	6985717	NE	-	*			hypothetical protein M5005_Spy_0702
M5005_Spy_0703	3572215	NE	Spy49_0712	6985718	NE	pyrF	*			orotidine 5\'-phosphate decarboxylase
M5005_Spy_0704	3572216	NE	Spy49_0713	6985719	NE	pyrE	*			orotate phosphoribosyltransferase
M5005_Spy_0705	3572217	NE	Spy49_0714	6985720	NE	amiC	*			amidase
M5005_Spy_0706	3572218	NE	Spy49_0715	6985721	NE	-	*			cystine-binding protein
M5005_Spy_0707	3572219	NE	Spy49_0716	6985722	NE	-	*			cystine transporter permease
M5005_Spy_0708	3572220	NE	Spy49_0717	6985723	NE	ung	*			uracil-DNA glycosylase
M5005_Spy_0709	3572221	NE	Spy49_0718	6985724	NE	pyrC	*			dihydroorotase
M5005_Spy_0710c	3572186	E	Spy49_0719c	6985725	E	**-**	*	SSA_1233	SP_0851	glycerol-3-phosphate acyltransferase
M5005_Spy_0711	3572187	E	Spy49_0720	6985726	E	**parE**	*	SSA_1232	SP_0852	DNA topoisomerase IV subunit B
M5005_Spy_0712	3572188	E	Spy49_0721	6985727	E	**parC**	*	SSA_1226	SP_0855	DNA topoisomerase IV subunit A
M5005_Spy_0713	3572189	NE	Spy49_0722	6985728	NE	bcaT	*			branched-chain amino acid aminotransferase
M5005_Spy_0714	3572190	NC	Spy49_0723	6985729	NC	-				hypothetical protein M5005_Spy_0714
M5005_Spy_0715	3572193	E	Spy49_0724	6985732	E	**rpsA**	*	SSA_1223	SP_0862	30S ribosomal protein S1
M5005_Spy_0716c	3572195	NE	Spy49_0725c	6985734	NE	-				hypothetical protein M5005_Spy_0716
M5005_Spy_0717	3572196	NE	Spy49_0726	6985735	NE	-	*			hypothetical protein M5005_Spy_0717
M5005_Spy_0718	3572197	NE	Spy49_0727	6985736	NE	-				hypothetical protein M5005_Spy_0718
M5005_Spy_0719	3572198	NE				-				glutathione S-transferase
M5005_Spy_0720	3572199	NE	Spy49_0728	6985737	NE	-				exfoliative toxin
M5005_Spy_0721c	3572200	NC	Spy49_0729c	6985738	NE	-				hypothetical protein M5005_Spy_0721
M5005_Spy_0722	3572201	NE	Spy49_0730	6985739	NE	miaA	*			tRNA delta(2)-isopentenylpyrophosphate transferase
M5005_Spy_0723	3572166	NE	Spy49_0731	6985740	NE	hflX	*			GTP-binding protein
M5005_Spy_0724	3572167	NE	Spy49_0732	6985741	NE	-	*			hypothetical protein M5005_Spy_0724
M5005_Spy_0725	3572168	E	Spy49_0733	6985742	E	**elaC**	*	SSA_1430	SP_0674	ribonuclease Z
M5005_Spy_0726	3572169	NE	Spy49_0734	6985743	NE	-	*			short chain dehydrogenase
M5005_Spy_0727	3572170	NE	Spy49_0735	6985744	NE	recJ	*			single-stranded-DNA-specific exonuclease
M5005_Spy_0728	3572171	NE	Spy49_0736	6985745	NE	apt	*			adenine phosphoribosyltransferase
M5005_Spy_0729	3572172	NC	Spy49_0737	6985746	E	dnaD	*	SSA_1419	SP_1575	DNA replication protein
M5005_Spy_0730	3572173	NE	Spy49_0738	6985747	NE	nth	*			endonuclease III
M5005_Spy_0731	3572174	E	Spy49_0739	6985748	E	**-**	*		SP_1610	para-aminobenzoate synthase component I/anthranilate synthase component I
M5005_Spy_0732	3572175	NE	Spy49_0740	6985749	NE	-	*			NIF3-related protein
M5005_Spy_0733	3572176	NE	Spy49_0741	6985750	NE	-	*			glycine/D-amino acid oxidase
M5005_Spy_0734	3572177	E	Spy49_0742	6985751	E	**cpsFO/rmlA**	*			glucose-1-phosphate thymidylyltransferase
M5005_Spy_0735	3572178	NC	Spy49_0744	6985752	NC	cpsFP/rmlC	*			dTDP-4-dehydrorhamnose 3,5-epimerase
M5005_Spy_0736	3572179	E	Spy49_0745	6985753	E	**cpsFQ/rmlB**	*			dTDP-glucose 4,6-dehydratase
			Spy49_0746c	6985754	NE					
			Spy49_0747c	6985755	NE					
			Spy49_0748c	6985756	NE					
			Spy49_0749c	6985757	C					
			Spy49_0750	6985758	NC					
			Spy49_0751c	6985759	NC					
			Spy49_0752	6985760	NC					
			Spy49_0753	6985761	NC					
			Spy49_0754	6985762	NC					
			Spy49_0755	6985763	NC					
			Spy49_0756	6985764	NE					
			Spy49_0757	6985765	NC					
			Spy49_0759	6985766	NC					
			Spy49_0760	6985767	NE					
			Spy49_0761	6985768	NE					
			Spy49_0762	6985769	NC					
			Spy49_0763	6985770	NC					
			Spy49_0764	6985771	NC					
			Spy49_0765	6985772	NC					
			Spy49_0766	6985773	NE					
			Spy49_0767	6985774	NE					
			Spy49_0768	6985775	NE					
			Spy49_0769	6985776	NE					
			Spy49_0770	6985777	NE					
			Spy49_0771	6985778	NE					
			Spy49_0772	6985779	NE					
			Spy49_0773	6985780	NC					
			Spy49_0774	6985781	NE					
			Spy49_0775	6985782	NE					
			Spy49_0776	6985783	NE					
			Spy49_0777	6985784	NE					
			Spy49_0778	6985785	NC					
			Spy49_0779	6985786	C					
			Spy49_0780	6985787	NC					
			Spy49_0781	6985788	NE					
			Spy49_0782	6985789	NC					
			Spy49_0783	6985790	NE					
			Spy49_0784	6985791	NC					
			Spy49_0785	6985792	NC					
			Spy49_0786	6985793	NC					
			Spy49_0788	6985794	NE					
			Spy49_0789	6985795	NE					
			Spy49_0790	6985796	NE					

			Spy49_0791	6985797	NE					
			Spy49_0792	6985798	NE	speH				
M5005_Spy_0737	3572180	NE	Spy49_0793	6985799	NE	mutX	*			7,8-dihydro-8-oxoguanine-triphosphatase
M5005_Spy_0738	3572181	NE	Spy49_0794	6985800	NE	-	*			hypothetical protein M5005_Spy_0738
M5005_Spy_0739	3572182	NE	Spy49_0795	6985801	NE	-	*			hypothetical protein M5005_Spy_0739
M5005_Spy_0740c	3572183	NE	Spy49_0796c	6985802	NE	fbp	*			fibronectin-binding protein
M5005_Spy_0742	3572185	NC				-				hypothetical protein M5005_Spy_0742
M5005_Spy_0743	3572147	NE	Spy49_0797	6985803	NE	-	*			ABC transporter substrate-binding protein
M5005_Spy_0744	3572148	NC	Spy49_0798	6985804	NC	-				hypothetical protein M5005_Spy_0744
M5005_Spy_0745	3572149	NE	Spy49_0799	6985805	NE	-	*			ABC transporter permease
M5005_Spy_0746	3572150	NE	Spy49_0800	6985806	NE	-	*			ABC transporter ATP-binding protein
M5005_Spy_0747	3572151	E	Spy49_0801	6985807	E	**-**	*	SSA_0312	SP_0121	Zn-dependent hydrolase
M5005_Spy_0748	3572152	NE	Spy49_0803	6985808	NE	estA	*			acetyl esterase
M5005_Spy_0749	3572153	NE	Spy49_0804	6985809	NE	-	*			hypothetical protein M5005_Spy_0749
M5005_Spy_0750	3572154	NE	Spy49_0805	6985810	NE	-	*			ABC transporter ATP-binding protein
M5005_Spy_0751	3572155	NE	Spy49_0807	6985811	NE	acoA	*			pyruvate dehydrogenase E1 component subunit alpha
M5005_Spy_0752	3572156	NE	Spy49_0808	6985812	NE	acoB	*			pyruvate dehydrogenase E1 component subunit beta
M5005_Spy_0753	3572157	NE	Spy49_0809	6985813	NE	acoC	*			branched-chain alpha-keto acid dehydrogenase subunit E2
M5005_Spy_0754c	3572158	NE				-				hypothetical protein M5005_Spy_0754
M5005_Spy_0755	3572159	NE	Spy49_0810	6985814	NE	acoL	*			dihydrolipoamide dehydrogenase
M5005_Spy_0756c	3572160	NE				-				hypothetical protein M5005_Spy_0756
M5005_Spy_0757c	3572161	NE	Spy49_0811c	6985815	NE	hylA				hyaluronate lyase
M5005_Spy_0758	3572162	NE	Spy49_0812	6985816	NE	lplB	*			lipoate-protein ligase A
M5005_Spy_0759c	3572163	E	Spy49_0813c	6985817	E	**cobQ**	*	SSA_0800	SP_1590	hypothetical protein M5005_Spy_0759
M5005_Spy_0760c	3572164	E	Spy49_0814c	6985818	E	**murC2**	*	SSA_0801	SP_1589	UDP-N-acetylmuramoylalanyl-D-glutamate--2,6-diaminopimelate ligase
M5005_Spy_0761	3572165	E	Spy49_0815	6985819	E	**-**	*		SP_1561	hypothetical protein M5005_Spy_0761
M5005_Spy_0762	3572127	NE	Spy49_0816	6985820	NE	-	*			hypothetical protein M5005_Spy_0762
M5005_Spy_0763	3572128	E	Spy49_0817	6985821	E	**glmM**	*	SSA_0804	SP_1559	phosphoglucosamine mutase
M5005_Spy_0764	3572129	NE	Spy49_0818	6985822	NE	-	*			hypothetical protein M5005_Spy_0764
M5005_Spy_0765	3572130	NE	Spy49_0819	6985823	NE	hemN	*			coproporphyrinogen III oxidase
M5005_Spy_0766	3572131	C	Spy49_0820	6985824	E	-	*			acyl-ACP thioesterase
M5005_Spy_0767	3572132	NE	Spy49_0821	6985825	NE	-	*			4-nitrophenylphosphatase
M5005_Spy_0768	3572133	NE	Spy49_0822	6985826	NE	-				hypothetical protein M5005_Spy_0768
M5005_Spy_0769	3572134	NE	Spy49_0823	6985827	NE	cas9				hypothetical protein M5005_Spy_0769
M5005_Spy_0770	3572135	NE	Spy49_0825	6985828	NE	cas1				hypothetical protein M5005_Spy_0770
M5005_Spy_0771	3572136	NE	Spy49_0826	6985829	NE	cas2				hypothetical protein M5005_Spy_0771
M5005_Spy_0772	3572137	NE	Spy49_0827	6985830	NE	csn2				hypothetical protein M5005_Spy_0772
M5005_Spy_0773c	3572138	NE				-				hypothetical protein M5005_Spy_0773
M5005_Spy_0774	3572139	NE				-				nucleoside diphosphate kinase
M5005_Spy_0775	3572140	NC	Spy49_0828	6985831	NE	-				nucleoside diphosphate kinase
M5005_Spy_0776	3572141	NE	Spy49_0829	6985832	NE	lepA				GTP-binding protein LepA
M5005_Spy_0777	3572142	NE	Spy49_0830	6985833	NC	sclB				hypothetical protein M5005_Spy_0777
M5005_Spy_0778	3572143	NE	Spy49_0831	6985834	NE	msrB/crsA	*			methionine sulfoxide reductase B
M5005_Spy_0779	3572144	NE	Spy49_0832	6985835	NE	-	*			hypothetical protein M5005_Spy_0779
M5005_Spy_0780	3572145	NE	Spy49_0833	6985836	NE	-	*			PTS system mannose/fructose family transporter subunit IIA
M5005_Spy_0781	3572146	NE	Spy49_0834	6985837	NC	ptsB	*			PTS system mannose/fructose family transporter subunit IIB
M5005_Spy_0782	3572108	NE	Spy49_0835	6985838	NE	ptsC	*			PTS system mannose/fructose family transporter subunit IIC
M5005_Spy_0783	3572109	NE	Spy49_0836	6985839	NE	ptsD	*			PTS system mannose/fructose family transporter subunit IID
M5005_Spy_0784	3572110	NE	Spy49_0837	6985840	NE	-	*			two-component sensor kinase
M5005_Spy_0785	3572111	NE	Spy49_0839	6985841	NE	-	*			two-component response regulator
M5005_Spy_0786	3572112	NE	Spy49_0840	6985842	NE	-	*			iron(III)-binding protein
M5005_Spy_0787	3572113	NE				-				hypothetical protein M5005_Spy_0787
M5005_Spy_0788	3572114	NE				-				acetyltransferase
M5005_Spy_0789	3572115	NE				-				HAD superfamily hydrolase
M5005_Spy_0790	3572116	NE	Spy49_0841	6985843	NE	gabD	*			succinate-semialdehyde dehydrogenase
M5005_Spy_0791	3572117	NE	Spy49_0842	6985844	NE	uvrC	*			excinuclease ABC subunit C
M5005_Spy_0792	3572118	NE	Spy49_0843	6985845	NE	-	*			NAD(P)H-dependent quinone reductase
M5005_Spy_0793	3572119	NE	Spy49_0844	6985846	NE	-	*			dipeptidase PepV
M5005_Spy_0794c	3572120	C	Spy49_0845c	6985847	E	trmE	*		SP_1016	tRNA modification GTPase TrmE
M5005_Spy_0795	3572121	NE	Spy49_0846	6985848	NE	rplJ	*			50S ribosomal protein L10
			Spy49_0847	6985849	NC					
M5005_Spy_0796	3572122	NE	Spy49_0848	6985850	NC	rplL	*			50S ribosomal protein L7/L12
M5005_Spy_0797	3572123	NC				-				hypothetical protein M5005_Spy_0797
M5005_Spy_0798	3572124	NE				-				IFN-response binding factor 1
M5005_Spy_0799	3572125	NC				-				hypothetical protein M5005_Spy_0799
M5005_Spy_0800	3572126	NE				-				DNA-cytosine methyltransferase
M5005_Spy_0801	3572088	NC				-				relaxase
M5005_Spy_0802	3572089	NE				-				relaxase
M5005_Spy_0803	3572090	NE				srtI				lantibiotic production protein
M5005_Spy_0804	3572091	NE				srtR				nisin biosynthesis two-component response regulator
M5005_Spy_0805	3572092	NE				srtK				nisin biosynthesis sensor protein
M5005_Spy_0806	3572093	NC				srtA				lantibiotic protein
M5005_Spy_0807	3572094	NE				srtT				lantibiotic ABC transporter ATP-binding protein
M5005_Spy_0808	3572095	NE				srtF				lantibiotic ABC transporter ATP-binding protein
M5005_Spy_0809	3572096	NE				srtE				lantibiotic transport permease
M5005_Spy_0810	3572097	NE				srtG				lantibiotic transport permease
M5005_Spy_0811	3572098	NE				-				Cro/CI family transcriptional regulator
M5005_Spy_0812c	3572099	NE				-				hypothetical protein M5005_Spy_0812
M5005_Spy_0817	3572104	NE	Spy49_0849	6985851	NE	dacA1	*			D-alanyl-D-alanine carboxypeptidase
M5005_Spy_0818c	3572105	NE	Spy49_0850c	6985852	NE	-	*			polysaccharide deacetylase
M5005_Spy_0819	3572106	NC				-				hypothetical protein M5005_Spy_0819
M5005_Spy_0820	3572107	E	Spy49_0851	6985853	E	folC.1		SSA_0198	SP_0290	folylpolyglutamate synthase/dihydrofolate synthase
M5005_Spy_0821	3572069	NE	Spy49_0852	6985854	NE	folE	*			GTP cyclohydrolase I
M5005_Spy_0822	3572070	C	Spy49_0853	6985855	NC	folP	*	SSA_0197		dihydropteroate synthase
M5005_Spy_0823	3572071	NE	Spy49_0854	6985856	NE	folQ	*			dihydroneopterin aldolase
M5005_Spy_0824	3572072	NC	Spy49_0855	6985857	NE	folK	*			2-amino-4-hydroxy-6-hydroxymethyldihydropteridine pyrophosphokinase
M5005_Spy_0825	3572073	E	Spy49_0856	6985858	E	**murB**	*	SSA_1047		UDP-N-acetylenolpyruvoylglucosamine reductase
M5005_Spy_0826	3572074	NE	Spy49_0857	6985859	NE	potA	*			spermidine/putrescine transporter ATP-binding protein
M5005_Spy_0827	3572075	NE	Spy49_0858	6985860	NE	potB	*			spermidine/putrescine transporter permease
M5005_Spy_0828	3572076	NE	Spy49_0859	6985861	NE	potC	*			spermidine/putrescine transporter permease
M5005_Spy_0829	3572077	NE	Spy49_0860	6985862	NE	potD	*			spermidine/putrescine-binding protein
M5005_Spy_0830c	3572078	NE	Spy49_0861c	6985863	NE	dpiA	*			transcriptional regulatory protein
M5005_Spy_0831c	3572079	NE	Spy49_0862c	6985864	NE	dpiB	*			sensor kinase
M5005_Spy_0832	3572080	NE	Spy49_0863	6985865	NE	malP	*			malate-sodium symport
M5005_Spy_0833	3572081	NE	Spy49_0864	6985866	NE	-	*			NAD-dependent malic enzyme
M5005_Spy_0834c	3572082	NE	Spy49_0865c	6985867	NE	-				Zn-dependent alcohol dehydrogenase and related dehydrogenase
M5005_Spy_0835	3572083	NE	Spy49_0866	6985868	NE	aphA	*			class B acid phosphatase
M5005_Spy_0837	3572085	NE	Spy49_0867	6985869	NE	-	*			chloride channel protein
M5005_Spy_0838	3572086	NE	Spy49_0868	6985870	NE	-	*			lipase/acylhydrolase
M5005_Spy_0839	3572087	NE	Spy49_0869	6985871	NE	-	*			hypothetical protein M5005_Spy_0839
M5005_Spy_0840	3572049	NE	Spy49_0870	6985872	NE	radC	*			DNA repair protein RadC
M5005_Spy_0841c	3572050	NE	Spy49_0871c	6985873	NE	-	*			glutamine amidotransferase, class I

M5005_Spy_0842c	3572051	NE	Spy49_0872c	6985874	NE	-	*			redox-sensing transcriptional repressor Rex
M5005_Spy_0843c	3572052	NE	Spy49_0873c	6985875	NE	-	*			hypothetical protein M5005_Spy_0843
M5005_Spy_0844c	3572053	E	Spy49_0874c	6985876	E	**nifS2**	*	SSA_1213	SP_1094	cysteine desulfhydrase
M5005_Spy_0845c	3572054	NE	Spy49_0875c	6985877	NE	prs	*			ribose-phosphate pyrophosphokinase
M5005_Spy_0846c	3572055	NE	Spy49_0876c	6985878	NC	-	*			adenylate cyclase
M5005_Spy_0847	3572056	NE	Spy49_0877	6985879	NE	-	*			GTP pyrophosphokinase
M5005_Spy_0848	3572057	E	Spy49_0878	6985880	E	**ppnK**	*	SSA_1209	SP_1098	inorganic polyphosphate/ATP-NAD kinase
M5005_Spy_0849	3572058	NE	Spy49_0879	6985881	NE	rluD	*			ribosomal large subunit pseudouridine synthase D
M5005_Spy_0851	3572060	NE	Spy49_0880	6985882	NE	pta/eutD	*			phosphotransacetylase
M5005_Spy_0852	3572061	NE	Spy49_0881	6985883	NE	-				short chain dehydrogenase
M5005_Spy_0853	3572062	NC				-				short chain dehydrogenase
M5005_Spy_0854c	3572063	NE	Spy49_0882c	6985884	NE	-	*			Na+ driven multidrug efflux pump
M5005_Spy_0855	3572064	NE	Spy49_0883	6985885	NE	proV				glycine betaine transport ATP-binding protein
M5005_Spy_0856	3572065	NE	Spy49_0884	6985886	NE	proX				glycine betaine transporter permease
M5005_Spy_0857	3572066	NE	Spy49_0885	6985887	NE	guaC	*			guanosine 5\'-monophosphate oxidoreductase
			Spy49_0886c	6985888	NE					
M5005_Spy_0858	3572067	NE	Spy49_0887	6985889	NE	xpt	*			xanthine phosphoribosyltransferase
M5005_Spy_0859	3572068	NE	Spy49_0888	6985890	NE	-	*			xanthine permease
M5005_Spy_0860c	3572030	NE	Spy49_0891c	6985891	NE	apbE	*			thiamine biosynthesis lipoprotein
M5005_Spy_0861c	3572031	NE	Spy49_0892c	6985892	NE	-				4-oxalocrotonate tautomerase
M5005_Spy_0862	3572032	NE	Spy49_0893	6985893	NE	tdk2	*			thymidine kinase
M5005_Spy_0863	3572033	E	Spy49_0894	6985894	E	**prfA**	*	SSA_1152	SP_1020	peptide chain release factor 1
M5005_Spy_0864	3572034	NE	Spy49_0895	6985895	NC	hemK	*			peptide release factor-glutamine N5-methyltransferase
M5005_Spy_0865	3572035	NC	Spy49_0896	6985896	E	-	*		SP_1022	SUA5 protein
M5005_Spy_0866	3572036	NE	Spy49_0897	6985897	NE	-	*			phosphinothricin N-acetyltransferase
M5005_Spy_0867	3572037	NE	Spy49_0898	6985898	NE	glyA	*			serine hydroxymethyltransferase
M5005_Spy_0868	3572038	NE	Spy49_0899	6985899	NC	-	*			hypothetical protein M5005_Spy_0868
M5005_Spy_0869	3572039	NE				-	*			hypothetical protein M5005_Spy_0869
			Spy49_0900	6985900	NE					
M5005_Spy_0870	3572040	NE	Spy49_0901	6985901	NC	-	*			multidrug resistance ABC transporter ATP-binding protein/permease
M5005_Spy_0871	3572041	NE	Spy49_0902	6985902	NE	-	*			multidrug resistance ABC transporter ATP-binding protein/permease
M5005_Spy_0872	3572042	NE	Spy49_0903	6985903	NE	nox	*			NADH oxidase H2O-forming
M5005_Spy_0873c	3572043	NE	Spy49_0904c	6985904	C	ldh	*		SP_1220	L-lactate dehydrogenase
M5005_Spy_0874	3572044	E	Spy49_0905	6985905	E	**gyrA**	*	SSA_1220	SP_1219	DNA gyrase subunit A
M5005_Spy_0875	3572045	E	Spy49_0906	6985906	C	-	*			sortase
M5005_Spy_0876	3572046	NE	Spy49_0907	6985907	C	-	*			lactoylglutathione lyase
M5005_Spy_0877	3572047	NC	Spy49_0908	6985908	NE	-				hypothetical protein M5005_Spy_0877
M5005_Spy_0878	3572048	NE				-	*			hypothetical protein M5005_Spy_0878
M5005_Spy_0879c	3572010	NE	Spy49_0909c	6985909	NE	-				hypothetical protein M5005_Spy_0879
M5005_Spy_0880c	3572011	C	Spy49_0910c	6985910	E	hlyIII	*			hypothetical protein M5005_Spy_0880
M5005_Spy_0881c	3572012	NE	Spy49_0911c	6985911	NE	-	*			hypothetical protein M5005_Spy_0881
M5005_Spy_0882	3572013	E	Spy49_0912	6985912	E	**rbgA**	*	SSA_1189	SP_1155	ribosomal biogenesis GTPase
M5005_Spy_0883	3572014	NE	Spy49_0913	6985913	NE	rnhB	*			ribonuclease HII
M5005_Spy_0884	3572015	NE	Spy49_0914	6985914	NE	smf	*			hypothetical protein M5005_Spy_0884
M5005_Spy_0885	3572016	C	Spy49_0915	6985915	E	topA	*	SSA_1184	SP_1263	DNA topoisomerase I
M5005_Spy_0886c	3572017	NE	Spy49_0916c	6985916	NE	-				LysR family transcriptional regulator
M5005_Spy_0887c	3572018	NC	Spy49_0917c	6985917	NC	-				LysR family transcriptional regulator
M5005_Spy_0888c	3572019	NC				-				LysR family transcriptional regulator
M5005_Spy_0889	3572020	NE	Spy49_0918	6985918	NE	-				regulatory protein (pfoS/R)
M5005_Spy_0890	3572021	NE	Spy49_0919	6985919	NE	ddh				D-lactate dehydrogenase
M5005_Spy_0891	3572022	E	Spy49_0920	6985920	E	satD				hypothetical protein M5005_Spy_0891
M5005_Spy_0892	3572023	NE	Spy49_0921	6985921	NE	satE				hypothetical protein M5005_Spy_0892
M5005_Spy_0893	3572024	NC	Spy49_0922	6985922	NC	gid	*			tRNA (uracil-5-)-methyltransferase Gid
M5005_Spy_0894	3572025	NE	Spy49_0923	6985923	NE	oadA2				oxaloacetate decarboxylase
M5005_Spy_0895	3572026	NE	Spy49_0924	6985924	NE	-				hypothetical protein M5005_Spy_0895
M5005_Spy_0896	3572027	NE	Spy49_0925	6985925	NE	-				biotin carboxyl carrier protein of oxaloacetate decarboxylase
M5005_Spy_0897	3572028	NE	Spy49_0926	6985926	NC	-				oxaloacetate decarboxylase subunit beta
M5005_Spy_0898c	3572029	NE	Spy49_0927c	6985927	NE	-				2-(5\'\'-triphosphoribosyl)-3\'-dephosphocoenzyme-A synthase
M5005_Spy_0899c	3571991	NE	Spy49_0928c	6985928	NE	citG				GntR family transcriptional regulator
M5005_Spy_0900c	3571992	NE	Spy49_0929c	6985929	NE	-				Mg2+/citrate complex secondary transporter
M5005_Spy_0901	3571993	NE	Spy49_0930	6985930	NE	-				hypothetical protein M5005_Spy_0901
M5005_Spy_0902	3571994	NE				-				acetyl-CoA carboxylase biotin carboxyl carrier protein subunit
M5005_Spy_0903	3571995	NE	Spy49_0933	6985931	NE	oadB				oxaloacetate decarboxylase subunit beta
M5005_Spy_0904	3571996	NC				-				hypothetical protein M5005_Spy_0904
M5005_Spy_0905	3571997	NE	Spy49_0934	6985932	NE	citD				citrate lyase subunit gamma
M5005_Spy_0906	3571998	NE	Spy49_0936	6985933	NC	citE				citrate lyase subunit beta/citryl-CoA lyase subunit
M5005_Spy_0907	3571999	NC	Spy49_0937	6985934	NE	citF				citrate lyase subunit alpha/citrate CoA-transferase
M5005_Spy_0908	3572000	NE	Spy49_0938	6985935	NE	citX				2-(5\'\'-triphosphoribosyl)-3\'-dephosphocoenzyme-A synthase
M5005_Spy_0909	3572001	NE	Spy49_0939	6985936	NE	oadA1				oxaloacetate decarboxylase
M5005_Spy_0910c	3572002	NE	Spy49_0940c	6985937	NE	citC	*			(citrate (pro-3S)-lyase) ligase
M5005_Spy_0911c	3572003	NE	Spy49_0942c	6985938	NE	-	*			hypothetical protein M5005_Spy_0911
M5005_Spy_0912	3572004	NC				-				hypothetical protein M5005_Spy_0912
M5005_Spy_0913c	3572005	C	Spy49_0943c	6985939	C	xerS	*		SP_1159	site-specific tyrosine recombinase XerS
M5005_Spy_0914	3572006	NE	Spy49_0944	6985940	NE	-	*			phage transcriptional repressor
M5005_Spy_0915c	3572007	E	Spy49_0945c	6985941	E	**ffh**	*	SSA_1167	SP_1287	signal recognition particle subunit FFH/SRP54
M5005_Spy_0916c	3572008	C	Spy49_0946c	6985942	NC	ylxM	*			DNA-binding protein
M5005_Spy_0917c	3572009	C	Spy49_0947c	6985943	NE	-	*			GntR family transcriptional regulator
M5005_Spy_0918c	3571971	NE	Spy49_0948c	6985944	NE	-	*			hypothetical protein M5005_Spy_0918
M5005_Spy_0919	3571972	C	Spy49_0949	6985945	C	guaA	*		SP_1445	GMP synthase
M5005_Spy_0920c	3571973	NE	Spy49_0951c	6985946	NE	murM2				UDP-N-acetylmuramoylpentapeptide-lysine N(6)-alanyltransferase
M5005_Spy_0921c	3571974	NE	Spy49_0952c	6985947	NE	-	*			ABC transporter ATP-binding protein
M5005_Spy_0922c	3571975	NE	Spy49_0953c	6985948	NE	pdxK	*			hypothetical protein M5005_Spy_0922
M5005_Spy_0923c	3571976	NE	Spy49_0954c	6985949	NE	-	*			pyridoxamine kinase
M5005_Spy_0924	3571977	NE	Spy49_0955	6985950	NE	-	*			GntR family transcriptional regulator
M5005_Spy_0925c	3571978	NE	Spy49_0957c	6985951	NE	rnhB	*			anaerobic ribonucleoside-triphosphate reductase
M5005_Spy_0926c	3571979	NC	Spy49_0958c	6985952	E	-	*			cardiolipin synthetase
M5005_Spy_0927c	3571980	E	Spy49_0959c	6985953	C	fhs.1	*			formate--tetrahydrofolate ligase
M5005_Spy_0928c	3571981	NE	Spy49_0960c	6985954	NE	lplA	*			lipoate-protein ligase A
M5005_Spy_0929c	3571982	NE	Spy49_0961c	6985955	NE	-	*			SIR2 family protein
M5005_Spy_0930c	3571983	NE	Spy49_0962c	6985956	NE	-	*			hypothetical protein M5005_Spy_0930
M5005_Spy_0931c	3571984	NE	Spy49_0963c	6985957	NE	-	*			glycine cleavage system protein H
M5005_Spy_0932c	3571985	NE	Spy49_0964c	6985958	NE	-	*			luciferase-like monooxygenase
M5005_Spy_0933c	3571986	NE	Spy49_0965c	6985959	NE	-	*			NADH-dependent flavin oxidoreductase
M5005_Spy_0934c	3571987	NE	Spy49_0966c	6985960	NE	-	*			lipoate-protein ligase A
M5005_Spy_0935	3571988	C	Spy49_0967	6985961	E	dpfB	*		SP_1230	phosphopantothenate--cysteine ligase
M5005_Spy_0936	3571989	E	Spy49_0968	6985962	E	**dfp**	*		SP_1231	phosphopantothenoylcysteine decarboxylase
M5005_Spy_0937	3571990	NE	Spy49_0969	6985963	C	-	*			transporter
M5005_Spy_0938	3571952	E	Spy49_0970	6985964	E	**pgmA**	*		SP_1498	phosphoglucomutase/phosphomannomutase
M5005_Spy_0939c	3571953	NE	Spy49_0971c	6985965	NE	-	*			nucleoside transporter permease
M5005_Spy_0940c	3571954	NE	Spy49_0972c	6985966	NE	-	*			nucleoside transporter permease
M5005_Spy_0941c	3571955	NE	Spy49_0973c	6985967	NE	-	*			nucleoside transport ATP-binding protein
M5005_Spy_0942c	3571956	NE	Spy49_0974c	6985968	NE	-	*			nucleoside-binding protein

M5005_Spy_0943c	3571957	NE	Spy49_0975c	6985969	NE	cdd	*			cytidine deaminase
M5005_Spy_0944c	3571958	NE	Spy49_0976c	6985970	NE	-	*			16S rRNA m(2)G 1207 methyltransferase
M5005_Spy_0945	3571959	E	Spy49_0977	6985971	E	**coaA**	*		SP_0839	pantothenate kinase
M5005_Spy_0946	3571960	NE	Spy49_0978	6985972	NC	rpsT				30S ribosomal protein S20
M5005_Spy_0947c	3571961	NE	Spy49_0979c	6985973	C	ciaH	*			sensor protein
M5005_Spy_0948c	3571962	NE	Spy49_0980c	6985974	E	ciaR	*			transcriptional regulatory protein
M5005_Spy_0949c	3571963	NE	Spy49_0981c	6985975	NE	pepN	*			lysyl aminopeptidase/alanine aminopeptidase
M5005_Spy_0950c	3571964	NC	Spy49_0982c	6985976	NE	phoU	*			phosphate transporter protein
M5005_Spy_0951c	3571965	NE	Spy49_0983c	6985977	NC	pstB	*			phosphate transporter ATP-binding protein
M5005_Spy_0952c	3571966	NE	Spy49_0984c	6985978	C	pstB2	*	SSA_0944		phosphate transporter ATP-binding protein
M5005_Spy_0953c	3571967	NE	Spy49_0985c	6985979	NC	pstA	*			phosphate transporter permease
M5005_Spy_0954c	3571968	NE	Spy49_0986	6985980	NC	pstC	*			phosphate transporter permease
M5005_Spy_0955c	3571969	NE	Spy49_0987c	6985981	NC	pstS	*			phosphate-binding protein
M5005_Spy_0956c	3571970	E	Spy49_0988c	6985982	E	**-**	*			16S rRNA m(5)C 967 methyltransferase
M5005_Spy_0957c	3571932	NE	Spy49_0989c	6985983	NE	-	*			myo-inositol-1(or 4)-monophosphatase
M5005_Spy_0958c	3571933	NE	Spy49_0990c	6985984	NE	-				hypothetical protein M5005_Spy_0958
M5005_Spy_0959c	3571934	NE	Spy49_0991c	6985985	NE	spxA	*			Spx family transcriptional regulator
M5005_Spy_0960c	3571935	E	Spy49_0992c	6985986	E	**mreA**	*	SSA_0936	SP_1110	bifunctional riboflavin kinase/FMN adenylyltransferase
M5005_Spy_0961c	3571936	NE	Spy49_0993c	6985987	NE	truB	*			tRNA pseudouridine synthase B
M5005_Spy_0962c	3571937	NE	Spy49_0994c	6985988	NE	-	*			hypothetical protein M5005_Spy_0962
M5005_Spy_0963c	3571938	NE	Spy49_0995c	6985989	NE	-	*			hypothetical protein M5005_Spy_0963
M5005_Spy_0964c	3571939	NE	Spy49_0996c	6985990	NE	-	*			type I restriction-modification system specificity subunit
M5005_Spy_0965c	3571940	NE	Spy49_0997c	6985991	NE	-	*			ABC transporter permease
M5005_Spy_0967c	3571942	NE	Spy49_0998c	6985992	NE	-	*			ABC transporter ATP-binding protein
M5005_Spy_0968	3571943	NE	Spy49_0999	6985993	NE	-	*			TetR family transcriptional regulator
M5005_Spy_0969c	3571944	NC				-				hypothetical protein M5005_Spy_0969
M5005_Spy_0970c	3571945	NE	Spy49_1000c	6985994	NE	-	*			NAD-dependent K+ or Na+ uptake system component
M5005_Spy_0971c	3571946	NE	Spy49_1001c	6985995	NE	-	*			Gls24 family general stress protein
M5005_Spy_0972c	3571947	NE	Spy49_1002c	6985996	NE	-				hypothetical protein M5005_Spy_0972
M5005_Spy_0973c	3571948	NE	Spy49_1003c	6985997	NE	-	*			Gls24 family general stress protein
M5005_Spy_0974c	3571949	NC	Spy49_1004c	6985998	NC	-				small integral membrane protein
M5005_Spy_0975c	3571950	NE	Spy49_1005c	6985999	NE	-	*			hypothetical protein M5005_Spy_0975
M5005_Spy_0976c	3571951	NE	Spy49_1006c	6986000	NE	-	*			hypothetical protein M5005_Spy_0976
M5005_Spy_0977c	3571913	C	Spy49_1007c	6986001	E	pcrA	*		SP_1087	DNA helicase II
M5005_Spy_0978	3571915	NC	Spy49_1008	6986002	NC	-	*			Na(+)-linked D-alanine glycine permease
M5005_Spy_0979c	3571914	C				-				hypothetical protein M5005_Spy_0979
M5005_Spy_0980	3571916	NE	Spy49_1009	6986003	NE	-	*			cobalt-zinc-cadmium resistance protein
M5005_Spy_0981c	3571917	NE	Spy49_1010c	6986004	NE	cfa				cAMP factor
M5005_Spy_0982c	3571918	NE	Spy49_1011c	6986005	NE	-				histidine-binding protein
M5005_Spy_0983c	3571919	NC	Spy49_1012c	6986006	NE	-				histidine transport ATP-binding protein
M5005_Spy_0984c	3571920	NE	Spy49_1013c	6986007	NE	-				histidine transporter permease
M5005_Spy_0985c	3571921	NE	Spy49_1014c	6986008	NE	-				phnA protein
M5005_Spy_0986c	3571922	E	Spy49_1015c	6986009	E	glmS		SSA_2107	SP_1087	glucosamine--fructose-6-phosphate aminotransferase
M5005_Spy_0987c	3571923	NE	Spy49_1016c	6986010	NE	sipC				signal peptidase I
M5005_Spy_0988c	3571924	E	Spy49_1017c	6986011	E	**pyk**	*	SSA_0848	SP_0897	pyruvate kinase
M5005_Spy_0989c	3571925	E	Spy49_1018c	6986012	E	**pfkA**	*	SSA_0847	SP_0896	6-phosphofructokinase
M5005_Spy_0990c	3571926	E	Spy49_1019c	6986013	E	**dnaE**	*	SSA_0846	SP_0895	DNA polymerase III DnaE
M5005_Spy_0991	3571927	NC	Spy49_1020	6986014	NE	-	*			GntR family transcriptional regulator
M5005_Spy_0992	3571928	NE	Spy49_1021	6986015	C	-	*			ABC transporter ATP-binding protein
M5005_Spy_0993	3571929	NE	Spy49_1022	6986016	C	-	*			ABC transporter permease
M5005_Spy_0994c	3571930	C	Spy49_1023c	6986017	E	-	*			membrane-associated alkaline phosphatase
M5005_Spy_0995c	3571931	NE				-				phage protein
M5005_Spy_0996	3571893	NE				speA2				enterotoxin
M5005_Spy_0997c	3571894	NE				-				phage protein
M5005_Spy_0998c	3571895	NE				-				phage protein
M5005_Spy_0999c	3571896	NE				-				phage protein
M5005_Spy_1000c	3571897	NE				-				phage protein
M5005_Spy_1001c	3571898	NE				-				phage-associated cell wall hydrolase
M5005_Spy_1002c	3571899	NE				-				N-acetylmuramoyl-L-alanine amidase
M5005_Spy_1003c	3571900	NE				-				phage protein
M5005_Spy_1004c	3571901	NC				-				phage protein
M5005_Spy_1005c	3571902	NE				-				phage protein
M5005_Spy_1006c	3571903	NE				-				phage structural protein
M5005_Spy_1007c	3571904	NE				-				phage protein
M5005_Spy_1008c	3571905	NE				-				hypothetical protein M5005_Spy_1008
M5005_Spy_1009c	3571906	NE				-				phage protein
M5005_Spy_1010c	3571907	NC				-				phage protein
M5005_Spy_1011c	3571908	NE				-				phage protein
M5005_Spy_1012c	3571909	NE				-				antigen A
M5005_Spy_1013c	3571910	NE				-				antigen B
M5005_Spy_1014c	3571911	NE				-				antigen C
M5005_Spy_1015c	3571912	NE				-				phage protein
M5005_Spy_1016c	3571874	NE				-				phage protein
M5005_Spy_1017c	3571875	NE				-				phage protein
M5005_Spy_1018c	3571876	NE				-				phage protein
M5005_Spy_1019c	3571877	NE				-				phage scaffold protein
M5005_Spy_1020c	3571878	NE				-				phage protein
M5005_Spy_1021c	3571879	NE				-				phage protein
M5005_Spy_1022c	3571880	NE				-				portal protein
M5005_Spy_1023c	3571881	NE				-				terminase large subunit
M5005_Spy_1024c	3571882	NE				-				phage protein
M5005_Spy_1025c	3571883	NE				-				ArpU family phage encoded transcriptional regulator
M5005_Spy_1026c	3571884	NE				-				phage protein
M5005_Spy_1027c	3571885	NE				-				phage protein
M5005_Spy_1028c	3571886	NE				-				phage protein
M5005_Spy_1029c	3571887	NE				-				phage protein
M5005_Spy_1030c	3571888	NE				-				phage protein
M5005_Spy_1031c	3571889	NE				-				phage protein
M5005_Spy_1032c	3571890	NE				-				phage protein
M5005_Spy_1033c	3571891	NE				-				phage protein
M5005_Spy_1034c	3571892	NE				-				phage protein
M5005_Spy_1035c	3571854	NE				-				phage protein
M5005_Spy_1036c	3571855	NE				ssb2				phage single-strand DNA binding protein
M5005_Spy_1037c	3571856	NE				ssb1				phage single-strand DNA binding protein
M5005_Spy_1038c	3571857	NE				-				phage protein
M5005_Spy_1039c	3571858	NE				-				phage protein
M5005_Spy_1040c	3571859	NE				-				phage protein
M5005_Spy_1041c	3571860	NC				-				phage protein
M5005_Spy_1042c	3571861	NC				-				phage replication protein
M5005_Spy_1043c	3571862	NE				-				phage protein
M5005_Spy_1044	3571863	NE				-				phage protein
M5005_Spy_1045c	3571864	NC				-				transcriptional regulator

M5005_Spy_1046	3571865	NE				-				phage protein
M5005_Spy_1047c	3571866	NE				-				phage protein
M5005_Spy_1048	3571867	NE				-				phage protein
M5005_Spy_1049c	3571868	NC				-				phage protein
M5005_Spy_1050	3571869	C				-				phage transcriptional repressor
M5005_Spy_1051	3571870	NE				-				phage protein
M5005_Spy_1052	3571871	NE				int.1				integrase
M5005_Spy_1053	3571872	NC				-				hypothetical protein M5005_Spy_1053
M5005_Spy_1054	3571873	NE	Spy49_1024	6986019	NE	-				hypothetical protein M5005_Spy_1054
M5005_Spy_1055c	3571835	NE	Spy49_1025c	6986020	NE	glgP	*			glycogen phosphorylase
M5005_Spy_1056c	3571836	NE	Spy49_1026c	6986021	NE	malM	*			4-alpha-glucanotransferase
M5005_Spy_1057c	3571837	NE	Spy49_1027c	6986022	NE	malR	*			LacI family transcriptional regulator
M5005_Spy_1058	3571838	NE	Spy49_1028	6986023	NE	malE	*			maltose/maltodextrin-binding protein
M5005_Spy_1059	3571839	NE	Spy49_1029	6986024	NE	malF	*			maltose ABC transporter permease
M5005_Spy_1060	3571840	NE	Spy49_1030	6986025	NE	malG	*			maltose ABC transporter permease
M5005_Spy_1061	3571841	NE				-				LacI family transcriptional regulator
M5005_Spy_1062c	3571842	NE				malA				maltodextrose utilization protein
M5005_Spy_1063c	3571843	NE				malD				maltodextrin transporter permease
M5005_Spy_1064c	3571844	NE				malC				maltose ABC transporter permease
M5005_Spy_1065c	3571845	NE				amyA				alpha-amylase
M5005_Spy_1066c	3571846	NE				amyB				neopullulanase/cyclomaltodextrinase/maltogenic alpha-amylase
M5005_Spy_1067c	3571847	NE				malX				maltose/maltodextrin-binding protein
M5005_Spy_1068	3571848	NE	Spy49_1032	6986027	NE	-				transposase
M5005_Spy_1069c	3571849	NE	Spy49_1033c	6986028	NE	-	*			esterase
M5005_Spy_1070c	3571850	NE	Spy49_1034c	6986029	NE	dltD	*			hypothetical protein M5005_Spy_1070
M5005_Spy_1071c	3571851	NE	Spy49_1035c	6986030	NE	dltC				D-alanine--poly(phosphoribitol) ligase subunit 2
M5005_Spy_1072c	3571852	NE	Spy49_1036c	6986031	NC	dltB	*			protein DltB
M5005_Spy_1073c	3571853	NE	Spy49_1037c	6986032	NC	dltA	*			D-alanine--poly(phosphoribitol) ligase subunit 1
M5005_Spy_1074c	3571815	NC	Spy49_1038c	6986033	NC	-				hypothetical protein M5005_Spy_1074
M5005_Spy_1075c	3571816	NE	Spy49_1039c	6986034	NE	uvrB	*			excinuclease ABC subunit B
M5005_Spy_1076	3571817	E	Spy49_1040	6986035	C	glnH	*		SP_1461	transporter
M5005_Spy_1077	3571818	NC	Spy49_1041	6986036	C	glnQ.2	*		SP_1460	glutamine ABC transporter ATP-binding protein
M5005_Spy_1078c	3571819	NC	Spy49_1042c	6986037	NC	-				hypothetical protein M5005_Spy_1078
M5005_Spy_1079c	3571820	NE	Spy49_1043c	6986038	NE	-				PTS system cellobiose-specific transporter subunit IIC
M5005_Spy_1080c	3571821	NE	Spy49_1044c	6986039	NE	-	*			hypothetical protein M5005_Spy_1080
M5005_Spy_1081c	3571822	NE	Spy49_1045c	6986040	NE	-	*			PTS system cellobiose-specific transporter subunit IIA
M5005_Spy_1082c	3571823	NE	Spy49_1046c	6986041	NE	-	*			PTS system cellobiose-specific transporter subunit IIB
M5005_Spy_1083c	3571824	NC	Spy49_1047c	6986042	NC	-	*			PTS system, mannitol (cryptic)-specific IIA component
M5005_Spy_1084c	3571825	NE	Spy49_1048c	6986043	NE	-	*			outer surface protein
M5005_Spy_1085c	3571826	NE	Spy49_1049c	6986044	NE	bglA.2	*			beta-glucosidase
M5005_Spy_1086	3571827	NE	Spy49_1050	6986045	NE	-	*			nicotinamide mononucleotide transporter
M5005_Spy_1087c	3571828	NC	Spy49_1051c	6986046	NC	-	*			hypothetical protein M5005_Spy_1087
M5005_Spy_1088c	3571829	E	Spy49_1053c	6986047	E	**obgE**	*	SSA_0807	SP_1079	GTPase ObgE
M5005_Spy_1089c	3571830	NC				-				hypothetical protein M5005_Spy_1089
M5005_Spy_1090	3571831	NE	Spy49_1054	6986048	NE	-	*			transposase
M5005_Spy_1091	3571832	NE	Spy49_1055	6986049	NE	-				transposase
			Spy49_1056	6986050	NE					
			Spy49_1057	6986051	NE					
			Spy49_1059	6986052	NE					
			Spy49_1060	6986053	NC					
			Spy49_1062	6986054	NE					
M5005_Spy_1092c	3571833	NE	Spy49_1064c	6986055	NC	rsuA	*			ribosomal small subunit pseudouridine synthase A
M5005_Spy_1093	3571834	NE	Spy49_1065	6986056	NE	-	*			hypothetical protein M5005_Spy_1093
M5005_Spy_1094c	3571796	NE	Spy49_1066c	6986057	NE	-	*			major facilitator transporter
M5005_Spy_1095	3571797	NE	Spy49_1067	6986058	NE	-	*			hypothetical protein M5005_Spy_1095
M5005_Spy_1096c	3571798	NE	Spy49_1070c	6986059	NE	-	*			thioesterase
M5005_Spy_1097c	3571799	NE	Spy49_1071c	6986060	NE	-				phosphorylase Pnp/Udp family protein
M5005_Spy_1098c	3571800	NE	Spy49_1072c	6986061	NE	-	*			tRNA (uracil-5-)-methyltransferase
M5005_Spy_1099c	3571801	NE	Spy49_1073c	6986062	NE	psr	*			LytR family transcriptional regulator
M5005_Spy_1100c	3571802	NE	Spy49_1074c	6986063	NE	aroK	*			shikimate kinase
M5005_Spy_1101c	3571803	NE	Spy49_1075c	6986064	NE	aroA1	*			3-phosphoshikimate 1-carboxyvinyltransferase
M5005_Spy_1102c	3571804	NE	Spy49_1076c	6986065	NE	-	*			ribonuclease BN
M5005_Spy_1103c	3571805	E	Spy49_1077c	6986066	E	**map**	*	SSA_1491	SP_1084	methionine aminopeptidase
M5005_Spy_1104c	3571806	NE	Spy49_1078c	6986067	NC	-	*			hypothetical protein M5005_Spy_1104
M5005_Spy_1105c	3571807	NE	Spy49_1079c	6986068	NE	-	*			ribosomal-protein-alanine acetyltransferase
M5005_Spy_1106c	3571808	NE	Spy49_1080c	6986069	NE	grab				protein G-related alpha 2M-binding protein
M5005_Spy_1107c	3571809	NE	Spy49_1082c	6986070	NE	murZ	*			UDP-N-acetylglucosamine 1-carboxyvinyltransferase
M5005_Spy_1108c	3571810	E	Spy49_1083c	6986071	E	**metK2**	*	SSA_1495	SP_0762	S-adenosylmethionine synthetase
M5005_Spy_1109c	3571811	NE	Spy49_1084c	6986072	NE	inlA	*			internalin protein
M5005_Spy_1110	3571812	E	Spy49_1085	6986073	E	**birA**	*	SSA_0999		biotin--protein ligase
M5005_Spy_1111c	3571813	NE	Spy49_1086c	6986074	NE	-				hypothetical protein M5005_Spy_1111
M5005_Spy_1112c	3571814	E	Spy49_1087c	6986075	E	dnaX		SSA_0997	SP_0865	DNA polymerase III subunits gamma/tau
M5005_Spy_1113c	3571776	NE	Spy49_1088c	6986076	NE	-	*			GAF domain-containing protein
M5005_Spy_1114	3571777	NE	Spy49_1089	6986077	NE	-	*			hypothetical protein M5005_Spy_1114
M5005_Spy_1115c	3571778	NE				-				hypothetical protein M5005_Spy_1115
M5005_Spy_1116c	3571779	NE	Spy49_1090c	6986078	NE	udk	*			uridine kinase
M5005_Spy_1117	3571780	NE	Spy49_1091	6986079	NE	deaD2	*			ATP-dependent RNA helicase
M5005_Spy_1118c	3571781	NE	Spy49_1092c	6986080	NE	-	*			peptidoglycan N-acetylglucosamine deacetylase
M5005_Spy_1119c	3571782	E	Spy49_1093c	6986081	E	gapN				NADP-dependent glyceraldehyde-3-phosphate dehydrogenase
M5005_Spy_1120c	3571783	C	Spy49_1094c	6986082	C	pstI	*		SP_1176	phosphoenolpyruvate-protein phosphotransferase
M5005_Spy_1121c	3571784	C	Spy49_1095c	6986083	NC	ptsH			SP_1177	phosphocarrier protein HPr
M5005_Spy_1122	3571785	NE	Spy49_1096	6986084	NE	nrdH				glutaredoxin
M5005_Spy_1123	3571786	NE	Spy49_1097	6986085	NE	nrdE.2	*			ribonucleotide-diphosphate reductase subunit alpha
			Spy49_1098	6986086	NE					
M5005_Spy_1124	3571787	NE	Spy49_1099	6986087	NE	nrdF	*			ribonucleotide-diphosphate reductase subunit beta
M5005_Spy_1125	3571788	NE	Spy49_1100	6986088	NE	-	*			chloride channel protein
			Spy49_1101	6986089	NC					
			Spy49_1103	6986090	NC					
			Spy49_1104	6986091	NE					
M5005_Spy_1129c	3571792	NE	Spy49_1105c	6986092	NE	-	*			CAAX amino protease
M5005_Spy_1130c	3571793	NE	Spy49_1106c	6986093	NE	-	*			hypothetical protein M5005_Spy_1130
M5005_Spy_1131c	3571794	NC	Spy49_1107c	6986094	NE	-				Cro/CI family transcriptional regulator
M5005_Spy_1132c	3571795	E	Spy49_1108c	6986095	E	**alaS**	*	SSA_0756	SP_1383	alanyl-tRNA synthetase
M5005_Spy_1133c	3571757	NE	Spy49_1109c	6986096	NE	prsA	*			foldase PrsA
M5005_Spy_1134c	3571758	NE	Spy49_1110c	6986097	NE	-	*			O-methyltransferase
M5005_Spy_1135c	3571759	NE	Spy49_1111c	6986098	NE	-	*			oxalate/formate antiporter
M5005_Spy_1136c	3571760	NE	Spy49_1112c	6986099	NE	pepB	*			oligoendopeptidase F
M5005_Spy_1137c	3571761	NE	Spy49_1113c	6986100	NE	-	*			competence protein/transcription factor
M5005_Spy_1138c	3571762	NE	Spy49_1114c	6986101	NE	-				ribosomal small subunit pseudouridine synthase A
M5005_Spy_1139c	3571763	NE	Spy49_1115c	6986102	NE	nagB	*			glucosamine-6-phosphate isomerase
M5005_Spy_1140	3571764	NE	Spy49_1116	6986103	NE	queA	*			S-adenosylmethionine--tRNA ribosyltransferase-isomerase

M5005_Spy_1141	3571765	NE	Spy49_1117	6986104	NE	-	*			hypothetical protein M5005_Spy_1141
M5005_Spy_1142c	3571766	NE	Spy49_1119c	6984357	NE	-	*			hypothetical protein M5005_Spy_1142
M5005_Spy_1143c	3571767	NE	Spy49_1120c	6984358	NE	-	*			hypothetical protein M5005_Spy_1143
M5005_Spy_1144c	3571768	NE	Spy49_1121c	6984359	NC	-	*			hypothetical protein M5005_Spy_1144
M5005_Spy_1145c	3571769	NE	Spy49_1122c	6984360	NE	sodA	*			superoxide dismutase
M5005_Spy_1146c	3571770	E	Spy49_1123c	6984361	E	**holA**	*	SSA_0720	SP_0765	DNA polymerase III subunit delta
M5005_Spy_1147c	3571771	NE	Spy49_1124c	6984362	NE	comEC	*			competence protein ComE
M5005_Spy_1148c	3571772	NE	Spy49_1126c	6984363	NE	comE	*			competence protein ComE
M5005_Spy_1149c	3571773	E	Spy49_1127c	6984364	E	**-**	*	SSA_0713		1-acyl-sn-glycerol-3-phosphate acyltransferase
M5005_Spy_1150	3571774	NE	Spy49_1128	6984365	NE	-	*			methyltransferase
M5005_Spy_1151	3571775	NE	Spy49_1129	6984366	NE	-	*			hypothetical protein M5005_Spy_1151
M5005_Spy_1152c	3571737	NE	Spy49_1130c	6984367	NE	kup	*			kup system potassium uptake protein, partial
M5005_Spy_1154c	3571739	NE	Spy49_1132c	6984368	NE	deaD	*			ATP-dependent RNA helicase
M5005_Spy_1155c	3571740	NE	Spy49_1133c	6984369	NE	prfC	*			peptide chain release factor 3
M5005_Spy_1156c	3571741	NE	Spy49_1135c	6984370	NE	-	*			hypothetical protein M5005_Spy_1156
M5005_Spy_1157c	3571742	E	Spy49_1136c	6984371	E	**murF**	*	SSA_0692	SP_1670	UDP-N-acetylmuramoylalanine-D-glutamyl-lysine--D-alanyl-D-alanine ligase
M5005_Spy_1158c	3571743	E	Spy49_1137c	6984372	E	**ddl**	*	SSA_0691	SP_1671	D-alanyl-alanine synthetase A
M5005_Spy_1159c	3571744	NE	Spy49_1138c	6984373	C	recR	*		SP_1672	recombination protein RecR
M5005_Spy_1160c	3571745	NE	Spy49_1139c	6984374	NE	-				penicillin-binding protein
M5005_Spy_1161c	3571746	NE	Spy49_1140c	6984375	NE	fdhC				formate transporter
M5005_Spy_1162c	3571747	NE	Spy49_1141c	6984376	NE	-	*			hypothetical protein M5005_Spy_1162
M5005_Spy_1163c	3571748	NE	Spy49_1142c	6984377	NE	-	*			biotin repressor family transcriptional regulator
M5005_Spy_1164c	3571749	NC	Spy49_1143c	6984378	E	gpmA	*	SSA_0688	SP_1655	phosphoglyceromutase
M5005_Spy_1165	3571750	NE	Spy49_1144	6984379	NE	pyrD	*			dihydroorotate dehydrogenase 1A
M5005_Spy_1166c	3571751	NC	Spy49_1145c	6984380	NC	-				hypothetical protein M5005_Spy_1166
M5005_Spy_1167c	3571752	NE	Spy49_1146c	6984381	NC	-	*			lead, cadmium, zinc and mercury transporting ATPase
M5005_Spy_1168c	3571753	NC				-				phage protein
M5005_Spy_1169	3571754	NE				spd3				streptodornase
M5005_Spy_1170	3571755	NE				-				hypothetical protein M5005_Spy_1170
M5005_Spy_1171c	3571756	NE				-				phage-associated cell wall hydrolase
M5005_Spy_1172c	3571718	NE				-				holin
M5005_Spy_1173c	3571719	NE				-				phage protein
M5005_Spy_1174c	3571720	NE				-				phage protein
M5005_Spy_1175c	3571721	NE				-				phage protein
M5005_Spy_1176c	3571722	NE				-				phage infection protein
M5005_Spy_1177c	3571723	NE				-				phage protein
M5005_Spy_1178c	3571724	NE				-				phage protein
M5005_Spy_1179c	3571725	NE				-				phage protein
M5005_Spy_1180c	3571726	NE				-				phage protein
M5005_Spy_1181c	3571727	NE				-				major tail protein
M5005_Spy_1182c	3571728	NE				-				phage protein
M5005_Spy_1183c	3571729	NE				-				phage protein
M5005_Spy_1184c	3571730	NE				-				phage protein
M5005_Spy_1185c	3571731	NE				-				phage protein
M5005_Spy_1186c	3571732	NE				-				phage protein
M5005_Spy_1187c	3571733	NE				-				phage structural protein
M5005_Spy_1188c	3571734	NE				-				phage protein
M5005_Spy_1189c	3571735	NE				-				phage terminase
M5005_Spy_1190c	3571736	NE				-				phage protein
M5005_Spy_1191c	3571698	NE				-				phage protein
M5005_Spy_1192c	3571699	NE				-				phage protein
M5005_Spy_1193c	3571700	NE				-				phage protein
M5005_Spy_1194c	3571701	NE				-				phage protein
M5005_Spy_1195c	3571702	NE				-				phage protein
M5005_Spy_1196c	3571703	NE				-				HNH endonuclease
M5005_Spy_1197c	3571704	NE				-				phage protein
M5005_Spy_1198c	3571705	NE				-				phage protein
M5005_Spy_1199c	3571706	NE				-				phage protein
M5005_Spy_1200c	3571707	NE				-				phage protein
M5005_Spy_1201c	3571708	NE				-				phage protein
M5005_Spy_1202c	3571709	NC				-				phage protein
M5005_Spy_1203c	3571710	NE				-				phage protein
M5005_Spy_1204c	3571711	NE				-				recT protein
M5005_Spy_1205c	3571712	NE				-				phage protein
M5005_Spy_1206c	3571713	NE				-				phage protein
M5005_Spy_1207c	3571714	NC				-				phage protein
M5005_Spy_1208c	3571715	NE				-				phage protein
M5005_Spy_1209c	3571716	NE				-				DNA replication protein
M5005_Spy_1210c	3571717	NE				-				phage replication protein
M5005_Spy_1211c	3571679	NC				-				phage protein
M5005_Spy_1212c	3571680	NE				xis				excisionase
M5005_Spy_1213c	3571681	NC				-				phage protein
M5005_Spy_1214	3571682	NE				-				phage protein
M5005_Spy_1215	3571683	NE				-				phage protein
M5005_Spy_1216c	3571684	NE				-				phage protein
M5005_Spy_1217c	3571685	NE				-				phage antirepressor protein
M5005_Spy_1218c	3571686	NE				-				phage protein
M5005_Spy_1219	3571687	E				-				Cro/CI family phage transcriptional regulator
M5005_Spy_1220	3571688	NE				-				phage protein
M5005_Spy_1221	3571689	NE				-				phage protein
M5005_Spy_1222	3571690	NE				int.2				integrase
M5005_Spy_1223c	3571691	NC	Spy49_1147c	6984382	NC	-	*			DNA-binding protein HU
M5005_Spy_1224c	3571692	NE	Spy49_1148c	6984383	NE	-	*			hypothetical protein M5005_Spy_1224
M5005_Spy_1225c	3571693	NE	Spy49_1149c	6984384	NE	-	*			lipase/acylhydrolase
M5005_Spy_1226c	3571694	NE	Spy49_1150c	6984385	C	-	*			degV family protein
M5005_Spy_1227c	3571695	NE	Spy49_1151c	6984386	NE	-	*			hypothetical protein M5005_Spy_1227
M5005_Spy_1228c	3571696	NE	Spy49_1152c	6984387	NE	recN	*			DNA repair protein
M5005_Spy_1229c	3571697	NE	Spy49_1153c	6984388	NE	argR1	*			arginine repressor ArgR
M5005_Spy_1230c	3571659	NE	Spy49_1154c	6984389	NE	-	*			hemolysin
M5005_Spy_1231c	3571660	NE	Spy49_1155c	6984390	NE	fps	*			dimethylallyltransferase/geranyltranstransferase
M5005_Spy_1232c	3571661	NE	Spy49_1156c	6984391	NE	xseB				exodeoxyribonuclease VII small subunit
M5005_Spy_1233c	3571662	NE	Spy49_1157c	6984392	NE	xseA	*			exodeoxyribonuclease VII large subunit
M5005_Spy_1234c	3571663	C	Spy49_1158c	6984393	NC	folD	*		SP_0825	bifunctional 5,10-methylene-tetrahydrofolate dehydrogenase/ 5,10-methylene-tetrahydrofolate cyclohyd...
M5005_Spy_1235c	3571664	NE	Spy49_1159c	6984394	NE	-	*			phosphoglucomutase
M5005_Spy_1236c	3571665	NE	Spy49_1160c	6984395	NE	phr	*			deoxyribodipyrimidine photolyase
M5005_Spy_1237c	3571666	NE	Spy49_1161c	6984396	NE	artP/gtr	*			arginine transport ATP-binding protein
M5005_Spy_1238c	3571667	NE	Spy49_1162c	6984397	NE	artQ	*			arginine transporter permease
M5005_Spy_1239c	3571668	NC	Spy49_1163c	6984398	NC	-	*			hypothetical protein M5005_Spy_1239
M5005_Spy_1240	3571669	NE	Spy49_1164	6984399	NE	clpE	*			ATP-dependent Clp protease ATP-binding subunit
M5005_Spy_1241	3571670	NE	Spy49_1165	6984400	NE	mutT	*			7,8-dihydro-8-oxoguanine-triphosphatase
M5005_Spy_1242	3571671	NE	Spy49_1166	6984401	NE	-	*			hypothetical protein M5005_Spy_1242

M5005_Spy_1243c	3571672	E	Spy49_1167c	6984402	E	**ileS**	*	SSA_0661	SP_1659	isoleucyl-tRNA synthetase
M5005_Spy_1244c	3571673	E	Spy49_1168c	6984403	NC	divIVAS	*			cell division initiation protein
M5005_Spy_1245c	3571674	NE	Spy49_1169c	6984404	NE	-	*			RNA binding protein
M5005_Spy_1246c	3571675	NC	Spy49_1170c	6984405	NE	-				hypothetical protein M5005_Spy_1246
M5005_Spy_1247c	3571676	NE	Spy49_1171c	6984406	C	-	*			hypothetical protein M5005_Spy_1247
M5005_Spy_1248c	3571677	NE	Spy49_1172c	6984407	NE	-	*			pyridoxal-5\'-phosphate family protein
M5005_Spy_1249c	3571678	E	Spy49_1173c	6984408	E	**ftsZ**	*	SSA_0656	SP_1666	cell division protein FtsZ
M5005_Spy_1250c	3571640	E	Spy49_1174c	6984409	E	**ftsA**	*		SP_1667	cell division protein
M5005_Spy_1251c	3571641	NC	Spy49_1175c	6984410	E	divIB/ftsQ	*		SP_0690	cell division protein
M5005_Spy_1252c	3571642	E	Spy49_1176c	6984411	E	**murG**	*	SSA_0653	SP_0689	undecaprenyldiphospho-muramoylpentapeptide beta-N-acetylglucosaminyltransferase
M5005_Spy_1253c	3571643	E	Spy49_1177c	6984412	E	**murD**	*	SSA_0652	SP_0688	UDP-N-acetylmuramoyl-L-alanyl-D-glutamate synthetase
M5005_Spy_1254c	3571644	NE	Spy49_1178c	6984413	NE	-				hypothetical protein M5005_Spy_1254
M5005_Spy_1255c	3571645	NC	Spy49_1179c	6984414	NE	typA	*			GTP-binding protein
M5005_Spy_1256c	3571646	NE	Spy49_1180c	6984415	NE	-	*			rhodanese-related sulfurtransferase
M5005_Spy_1257c	3571647	NE	Spy49_1181c	6984416	NE	glcK	*			glucokinase/xylose repressor
M5005_Spy_1258c	3571648	NE	Spy49_1182c	6984417	NE	-				hypothetical protein M5005_Spy_1258
M5005_Spy_1259c	3571649	NE	Spy49_1183c	6984418	NE	dpr	*			non-specific DNA-binding protein/iron-binding ferritin-like antioxidant protein
M5005_Spy_1260	3571650	NE	Spy49_1184	6984419	NE	-	*			prepilin peptidase
M5005_Spy_1261c	3571651	NE	Spy49_1185c	6984420	NE	-	*			ribosomal RNA large subunit methyltransferase N
M5005_Spy_1262c	3571652	NE	Spy49_1186c	6984421	NE	-	*			transcriptional regulator
M5005_Spy_1263c	3571653	NC				-				hypothetical protein M5005_Spy_1263
M5005_Spy_1264c	3571654	NE	Spy49_1187c	6984422	NE	-	*			ribose operon repressor, partial
M5005_Spy_1265c	3571655	NE				-	*			ribose operon repressor, partial
M5005_Spy_1266c	3571656	NE	Spy49_1188c	6984423	NE	-				ATP-dependent protease La
M5005_Spy_1267c	3571657	E	Spy49_1189c	6984424	E	**coaD**	*	SSA_0625	SP_1968	phosphopantetheine adenylyltransferase
M5005_Spy_1268c	3571658	NE	Spy49_1190c	6984425	NE	-	*			methyltransferase
M5005_Spy_1269c	3571620	NE	Spy49_1191c	6984426	NE	asnA	*			asparagine synthetase AsnA
M5005_Spy_1270c	3571621	NE	Spy49_1192c	6984427	NC	arcC	*			carbamate kinase
M5005_Spy_1271c	3571622	NE	Spy49_1193c	6984428	NE	-	*			hypothetical protein M5005_Spy_1271
M5005_Spy_1272c	3571623	NE	Spy49_1194c	6984429	NE	-	*			arginine/ornithine antiporter
M5005_Spy_1273c	3571624	NE	Spy49_1195c	6984430	NC	arcB	*			ornithine carbamoyltransferase
M5005_Spy_1274c	3571625	NE	Spy49_1196c	6984431	NE	-	*			acetyltransferase
M5005_Spy_1275c	3571626	NE	Spy49_1197c	6984432	NE	arcA	*			arginine deiminase
M5005_Spy_1276c	3571627	NE	Spy49_1198c	6984433	NE	-	*			Crp/Fnr family transcriptional regulator
M5005_Spy_1277	3571628	NE	Spy49_1199	6984434	NE	ahrC.2/argR	*			arginine repressor ArgR
M5005_Spy_1278c	3571629	NE	Spy49_1200c	6984435	NE	-	*			hypothetical protein M5005_Spy_1278
M5005_Spy_1279c	3571630	NE	Spy49_1201c	6984436	NE	-	*			hypothetical protein M5005_Spy_1279
M5005_Spy_1280c	3571631	NC	Spy49_1202c	6984437	NE	yesM				two-component sensor kinase
M5005_Spy_1281c	3571632	NE	Spy49_1203c	6984438	NE	yesN	*			two-component response regulator
M5005_Spy_1282c	3571633	NE	Spy49_1204c	6984439	NE	msrA	*			bifunctional methionine sulfoxide reductase A/B
M5005_Spy_1283c	3571634	NE	Spy49_1205c	6984440	NE	tlpA	*			thiol:disulfide interchange protein
M5005_Spy_1284c	3571635	NE	Spy49_1206c	6984441	NE	ccdA	*			cytochrome C biogenesis protein
M5005_Spy_1285c	3571636	NE	Spy49_1207c	6984442	NE	-				hypothetical protein M5005_Spy_1285
M5005_Spy_1286c	3571637	NE	Spy49_1208c	6984443	NE	-				DNA polymerase
M5005_Spy_1287c	3571638	NE	Spy49_1209c	6984444	NE	-				hypothetical protein M5005_Spy_1287
M5005_Spy_1288c	3571639	NE	Spy49_1210c	6984445	NE	-				hypothetical protein M5005_Spy_1288
M5005_Spy_1289c	3571602	NE	Spy49_1211c	6984446	NC	-				hypothetical protein M5005_Spy_1289
M5005_Spy_1290c	3571603	NE	Spy49_1212c	6984447	NE	-				hypothetical protein M5005_Spy_1290
M5005_Spy_1291c	3571604	NE	Spy49_1213c	6984448	NE	-				ATP-dependent RNA helicase
M5005_Spy_1292c	3571605	E	Spy49_1215c	6984449	E	**valS**	*	SSA_1819	SP_0568	valyl-tRNA synthetase
M5005_Spy_1293c	3571606	NE	Spy49_1216c	6984450	NE	-	*			hypothetical protein M5005_Spy_1293
M5005_Spy_1294c	3571607	NC	Spy49_1217c	6984451	NE	-				ribosomal-protein-serine acetyltransferase
M5005_Spy_1295c	3571608	NE	Spy49_1218c	6984452	NC	-	*			hypothetical protein M5005_Spy_1295
M5005_Spy_1296c	3571609	NE	Spy49_1219c	6984453	NC	-	*			hypothetical protein M5005_Spy_1296
M5005_Spy_1297	3571611	NE	Spy49_1220	6984455	NE	aroA2	*			3-deoxy-7-phosphoheptulonate synthase
M5005_Spy_1298	3571612	NE	Spy49_1221	6984456	NE	aroB	*			3-dehydroquinate synthase
M5005_Spy_1299c	3571613	NE				-				hypothetical protein M5005_Spy_1299
M5005_Spy_1300c	3571614	NE	Spy49_1222c	6984457	NE	-	*			hypothetical protein M5005_Spy_1300
M5005_Spy_1301c	3571615	NE	Spy49_1223c	6984458	NE	-	*			hypothetical protein M5005_Spy_1301
M5005_Spy_1302c	3571616	NE	Spy49_1224c	6984459	NC	-	*			SAM-dependent methyltransferase
M5005_Spy_1303c	3571617	NE	Spy49_1225c	6984460	NE	aroE	*			shikimate 5-dehydrogenase
M5005_Spy_1304c	3571618	NE	Spy49_1226c	6984461	NE	lacZ				beta-galactosidase
M5005_Spy_1305c	3571619	NE	Spy49_1227c	6984462	NE	trxR	*			two-component response regulator
M5005_Spy_1306c	3571583	NE	Spy49_1228c	6984463	NE	trxS	*			two-component sensor kinase
M5005_Spy_1307c	3571584	NE	Spy49_1229c	6984464	NE	trxT	*			hypothetical protein M5005_Spy_1307
M5005_Spy_1308c	3571585	NE	Spy49_1230c	6984465	NE	-	*			sugar-binding protein
M5005_Spy_1309c	3571586	NE	Spy49_1231c	6984466	NC	-	*			sugar transporter permease
M5005_Spy_1310c	3571587	NE	Spy49_1233c	6984467	NE	-	*			sugar transporter permease
M5005_Spy_1311	3571588	NE	Spy49_1234	6984468	NE	-	*			glucokinase
M5005_Spy_1312	3571589	NC				-				hypothetical protein M5005_Spy_1312
M5005_Spy_1313c	3571590	NE	Spy49_1235c	6984469	NE	-	*			beta-glucosidase
M5005_Spy_1314c	3571591	NE	Spy49_1236c	6984470	NC	hyl	*			hyaluronoglucosaminidase
M5005_Spy_1315c	3571592	NE	Spy49_1237c	6984471	NE	-	*			GntR family transcriptional regulator
M5005_Spy_1316	3571593	NE	Spy49_1238	6984472	NC	-	*			hypothetical protein M5005_Spy_1316
M5005_Spy_1317	3571594	NE	Spy49_1239	6984473	NC	-	*			alpha-mannosidase
M5005_Spy_1318c	3571595	NE	Spy49_1240c	6984474	NE	rocA	*			sensory transduction protein kinase
M5005_Spy_1319c	3571596	NE	Spy49_1241c	6984475	NC	-	*			tRNA (uracil-5-)-methyltransferase
M5005_Spy_1320	3571597	NE	Spy49_1242	6984476	NE	recX				recombination regulator RecX
M5005_Spy_1321	3571598	C	Spy49_1243	6984477	NC	-	*		SP_1903	hypothetical protein M5005_Spy_1321
M5005_Spy_1322c	3571599	NC	Spy49_1244	6984478	NE	-				hypothetical protein M5005_Spy_1322
M5005_Spy_1323	3571600	NE	Spy49_1245	6984479	C	-				transposase
M5005_Spy_1324	3571555	NC				-				hypothetical protein M5005_Spy_1324
M5005_Spy_1325c	3571556	NE	Spy49_1247c	6984490	NE	-	*			ribosome-associated factor Y
M5005_Spy_1326c	3571557	NE	Spy49_1248c	6984491	NE	comFC	*			competence protein ComF
M5005_Spy_1327c	3571558	NE	Spy49_1249c	6984492	NE	comFA	*			competence protein ComF
M5005_Spy_1328	3571559	NE	Spy49_1250	6984493	NE	-	*			Xaa-Pro dipeptidase
M5005_Spy_1329	3571560	NE	Spy49_1251	6984494	NE	cysM	*			cysteine synthase
M5005_Spy_1330c	3571561	C	Spy49_1252c	6984495	C	-	*			hypothetical protein M5005_Spy_1330
M5005_Spy_1331c	3571562	NE	Spy49_1253c	6984496	NE	-				peptidyl-prolyl cis-trans isomerase
M5005_Spy_1332c	3571563	NC	Spy49_1254c	6984497	E	yvqC	*			two-component response regulator
M5005_Spy_1333c	3571564	NE	Spy49_1255c	6984498	NE	yvqE	*			two-component sensor protein
M5005_Spy_1334c	3571565	NE	Spy49_1256c	6984499	NE	yvqF	*			transporter
M5005_Spy_1335c	3571566	E	Spy49_1257c	6984500	E	**-**	*		SP_1732	serine/threonine protein kinase
M5005_Spy_1336c	3571567	E	Spy49_1259c	6984501	E	**pppL**	*		SP_1733	protein phosphatase 2C
M5005_Spy_1337c	3571568	NE	Spy49_1260c	6984502	NE	sunL	*			16S rRNA m(5)C 967 methyltransferase
M5005_Spy_1338c	3571569	E	Spy49_1261c	6984503	E	**fmt**	*	SSA_1848	SP_1735	methionyl-tRNA formyltransferase
M5005_Spy_1339c	3571570	C	Spy49_1262c	6984504	C	priA	*		SP_1736	primosome assembly protein PriA
M5005_Spy_1340c	3571571	NE	Spy49_1263c	6984505	NC	-	*			DNA-directed RNA polymerase subunit omega
M5005_Spy_1341c	3571572	E	Spy49_1265c	6984506	E	**gmk**	*	SSA_1851	SP_1738	guanylate kinase
M5005_Spy_1342c	3571535	NC	Spy49_1266c	6984507	NE	-	*			hypothetical protein M5005_Spy_1342
M5005_Spy_1343c	3571536	NE	Spy49_1267c	6984508	NE	-	*			LysR family transcriptional regulator

M5005_Spy_1344	3571537	NE	Spy49_1268	6984509	NC	atoB	*			acetyl-CoA acetyltransferase
M5005_Spy_1345	3571538	NE	Spy49_1269	6984510	NE	atoD.1	*			acetate CoA-transferase subunit alpha
M5005_Spy_1346	3571539	NE	Spy49_1270	6984511	NE	atoA				acetate CoA-transferase subunit beta
M5005_Spy_1347	3571540	NE	Spy49_1271	6984512	NE	-				3-hydroxybutyrate dehydrogenase
M5005_Spy_1348	3571541	NE	Spy49_1272	6984513	NE	-	*			D-beta-hydroxybutyrate permease
M5005_Spy_1349	3571542	NE	Spy49_1273	6984514	NE	luxS	*			S-ribosylhomocysteinase
M5005_Spy_1350c	3571543	NE	Spy49_1274c	6984515	NE	-	*			hypothetical protein M5005_Spy_1350
M5005_Spy_1351c	3571544	NE	Spy49_1275c	6984516	NE	-	*			methyltransferase
M5005_Spy_1352c	3571545	NC	Spy49_1276c	6984583	NC	-	*			cell division initiation protein
M5005_Spy_1353c	3571546	NE	Spy49_1277c	6984584	NE	-	*			hypothetical protein M5005_Spy_1353
M5005_Spy_1354	3571547	NE	Spy49_1278	6984585	NC	recU	*			Holliday junction-specific endonuclease
M5005_Spy_1355	3571548	E	Spy49_1279	6984586	E	**pbp1A**	*			multimodular transpeptidase-transglycosylase
M5005_Spy_1356c	3571549	NE	Spy49_1280c	6984587	NE	pepC	*			aminopeptidase
M5005_Spy_1357c	3571550	E	Spy49_1281c	6984588	E	nadE		SSA_1863	SP_1420	NAD synthetase
M5005_Spy_1358c	3571551	E	Spy49_1282c	6984589	E	nadE		SSA_1864		nicotinate phosphoribosyltransferase
M5005_Spy_1359c	3571552	NE	Spy49_1283c	6984590	NE	-	*			amino acid permease
M5005_Spy_1360c	3571553	C	Spy49_1284c	6984591	NE	-		SSA_1865		thioredoxin reductase
M5005_Spy_1361c	3571554	E	Spy49_1285c	6984592	NC	aapA				hypothetical protein M5005_Spy_1361
M5005_Spy_1362c	3571516	NE	Spy49_1286c	6984593	NE	-	*			transporter
M5005_Spy_1363c	3571517	NE	Spy49_1287c	6984594	NE	-	*			amino acid ABC transporter permease
M5005_Spy_1364c	3571518	E	Spy49_1289c	6984595	E	**-**	*			ATP-dependent RNA helicase
M5005_Spy_1365c	3571519	E	Spy49_1290c	6984596	E	**mraY**	*	SSA_1870	SP_0337	phospho-N-acetylmuramoyl-pentapeptide-transferase
M5005_Spy_1366c	3571520	E	Spy49_1291c	6984597	E	**ftsI**	*	SSA_1871	SP_0336	division specific D,D-transpeptidase/cell division protein ftsI
M5005_Spy_1367c	3571521	E	Spy49_1292c	6984598	NC	ftsL	*	SSA_1872	SP_0335	cell division protein
M5005_Spy_1368c	3571522	C	Spy49_1293c	6984599	NE	mraW	*		SP_0334	S-adenosyl-methyltransferase MraW
M5005_Spy_1369c	3571523	NC	Spy49_1294c	6984600	NE	-				hypothetical protein M5005_Spy_1369
M5005_Spy_1370c	3571524	NE	Spy49_1295c	6984601	NE	proA				gamma-glutamyl phosphate reductase
M5005_Spy_1371c	3571525	NE	Spy49_1296c	6984602	NE	proB				gamma-glutamyl kinase
M5005_Spy_1372c	3571526	NE	Spy49_1297c	6984603	NE	proB	*			ABC transporter permease
M5005_Spy_1373c	3571527	NE	Spy49_1298c	6984604	NE	-	*			ABC transporter ATP-binding protein
M5005_Spy_1374c	3571528	NE	Spy49_1299c	6984605	NE	-				hypothetical protein M5005_Spy_1374
M5005_Spy_1375c	3571529	NE	Spy49_1300c	6984606	C	tkt	*			transketolase
M5005_Spy_1376c	3571530	NE	Spy49_1301c	6984607	NE	tal	*			translaldolase
M5005_Spy_1377c	3571531	NE	Spy49_1303c	6984608	NE	-				trans-acting positive regulator
M5005_Spy_1378c	3571532	NE	Spy49_1304c	6984609	NE	npx				NADH peroxidase
M5005_Spy_1379c	3571533	NE	Spy49_1305c	6984610	NE	glpF	*			glycerol uptake facilitator protein
M5005_Spy_1380c	3571534	NE	Spy49_1306c	6984611	NE	glpO				alpha-glycerophosphate oxidase
M5005_Spy_1381c	3571496	NE	Spy49_1307c	6984612	NE	glpK	*			glycerol kinase
M5005_Spy_1382c	3571497	NE	Spy49_1308c	6984613	NE	-	*			hypothetical protein M5005_Spy_1382
M5005_Spy_1383c	3571498	NC	Spy49_1309c	6984614	NE	-				hypothetical protein M5005_Spy_1383
M5005_Spy_1384c	3571499	E	Spy49_1310c	6984615	E	**glyS**	*	SSA_1879	SP_1474	glycyl-tRNA synthetase subunit beta
M5005_Spy_1385c	3571500	E	Spy49_1311c	6984616	E	**glyQ**	*	SSA_1880	SP_1475	glycyl-tRNA synthetase subunit alpha
M5005_Spy_1386c	3571501	NE	Spy49_1312c	6984617	NE	-	*			hypothetical protein M5005_Spy_1386
M5005_Spy_1387c	3571502	NE	Spy49_1313c	6984618	NE	-				aldo/keto reductase
M5005_Spy_1388c	3571503	NE	Spy49_1314c	6984619	NE	nagA	*			N-acetylglucosamine-6-phosphate deacetylase
M5005_Spy_1389c	3571504	NE	Spy49_1316c	6984620	NE	-	*			sodium-dependent phosphate transporter
M5005_Spy_1390	3571505	NE				-				hypothetical protein M5005_Spy_1390
M5005_Spy_1391c	3571506	NE	Spy49_1318c	6984621	NE	-	*			degV family protein
M5005_Spy_1392	3571507	NE	Spy49_1319	6984622	NE	-	*			TetR family transcriptional regulator
M5005_Spy_1393c	3571508	NE	Spy49_1320c	6984623	NE	-	*			HAD superfamily hydrolase
M5005_Spy_1394c	3571509	NE	Spy49_1321c	6984624	NE	-	*			hypothetical protein M5005_Spy_1394
M5005_Spy_1395c	3571510	NE	Spy49_1322c	6984625	NE	lacD.1	*			tagatose 1,6-diphosphate aldolase
M5005_Spy_1396c	3571511	NE	Spy49_1323c	6984626	NE	lacC1				tagatose-6-phosphate kinase
			Spy49_1324c	6984627	NC					
M5005_Spy_1397c	3571512	NE	Spy49_1325c	6984628	NE	lacB.1	*			galactose-6-phosphate isomerase subunit LacB
M5005_Spy_1398c	3571513	NE	Spy49_1326c	6984629	NE	lacA.1	*			galactose-6-phosphate isomerase subunit LacA
M5005_Spy_1399c	3571514	NE	Spy49_1327c	6984630	NE	-	*			PTS system galactose-specific transporter subunit IIC
M5005_Spy_1400c	3571515	NE	Spy49_1328c	6984631	NE	-	*			PTS system galactose-specific transporter subunit IIB
M5005_Spy_1401c	3571477	NE	Spy49_1329c	6984632	NE	-	*			PTS system galactose-specific transporter subunit IIA
M5005_Spy_1402	3571478	NE	Spy49_1330	6984633	NE	lacR.1	*			lactose phosphotransferase system repressor
M5005_Spy_1403c	3571479	NE				-				copper chaperone
M5005_Spy_1404c	3571480	NE	Spy49_1331c	6984634	NE	copZ				copper chaperone
M5005_Spy_1405c	3571481	NE	Spy49_1332c	6984635	NE	copA	*			copper-exporting ATPase
M5005_Spy_1406c	3571482	NE	Spy49_1334c	6984636	NE	copY				copAB ATPase metal-fist type repressor
M5005_Spy_1407	3571483	NE	Spy49_1335	6984637	NE	-				esterase
M5005_Spy_1408c	3571484	C	Spy49_1336c	6984638	NC	rbfA	*		SP_0557	ribosome-binding factor A
M5005_Spy_1409c	3571485	E	Spy49_1337c	6984639	E	**infB**	*		SP_0556	translation initiation factor IF-2
M5005_Spy_1410c	3571486	C	Spy49_1338c	6984640	NE	-	*		SP_0555	hypothetical protein M5005_Spy_1410
M5005_Spy_1411c	3571487	C	Spy49_1339c	6984641	NC	-	*		SP_0554	hypothetical protein M5005_Spy_1411
M5005_Spy_1412c	3571488	E	Spy49_1340c	6984642	E	**nusA**	*		SP_0553	transcription elongation factor NusA
M5005_Spy_1413c	3571489	NE	Spy49_1341c	6984643	NE	-	*			hypothetical protein M5005_Spy_1413
M5005_Spy_1414c	3571490	NC				-				phage protein
M5005_Spy_1415c	3571491	NE				sdaD2				phage-encoded streptodornase
M5005_Spy_1416c	3571492	NE				-				phage-associated cell wall hydrolase
M5005_Spy_1417c	3571493	NE				-				phage protein
M5005_Spy_1418c	3571494	NE				-				phage protein
M5005_Spy_1419c	3571495	NE				-				phage protein
M5005_Spy_1420c	3571457	NC				-				phage protein
M5005_Spy_1421c	3571458	NE				-				phage infection protein
M5005_Spy_1422c	3571459	NE				-				phage protein
M5005_Spy_1423c	3571460	NE				-				hyaluronoglucosaminidase
M5005_Spy_1424c	3571461	NE				-				phage endopeptidase
M5005_Spy_1425c	3571462	NE				-				phage protein
M5005_Spy_1426c	3571463	NE				-				phage protein
M5005_Spy_1427c	3571464	NE				-				phage protein
M5005_Spy_1428c	3571465	NE				-				phage protein
M5005_Spy_1429c	3571466	NE				-				phage protein
M5005_Spy_1430c	3571467	NE				-				phage protein
M5005_Spy_1431c	3571468	NE				-				phage protein
M5005_Spy_1432c	3571469	NE				-				phage protein
M5005_Spy_1433c	3571470	NE				-				phage protein
M5005_Spy_1434c	3571471	NE				-				phage protein
M5005_Spy_1435c	3571472	NE				-				phage scaffold protein
M5005_Spy_1436c	3571473	NE				-				phage protein
M5005_Spy_1437c	3571474	NE				-				hypothetical protein M5005_Spy_1437
M5005_Spy_1438c	3571475	NE				-				phage protein
M5005_Spy_1439c	3571476	NE				-				portal protein
M5005_Spy_1440c	3571438	NE				-				terminase large subunit
M5005_Spy_1441c	3571439	NE				-				phage terminase small subunit
M5005_Spy_1442c	3571440	NE				-				phage transcriptional activator
M5005_Spy_1443c	3571441	NE				-				phage protein
M5005_Spy_1444c	3571442	NE				-				adenine-specific methyltransferase

M5005_Spy_1445c	3571443	NE				-				phage protein
M5005_Spy_1446c	3571444	NC				-				phage protein
M5005_Spy_1447c	3571445	NE				-				phage-related DNA helicase
M5005_Spy_1448c	3571446	NE				-				hypothetical protein M5005_Spy_1448
M5005_Spy_1449c	3571447	NE				-				DNA primase
M5005_Spy_1450c	3571448	NE				-				phage-encoded DNA polymerase
M5005_Spy_1451c	3571449	NE				-				phage protein
M5005_Spy_1452c	3571450	NE				-				phage protein
M5005_Spy_1453c	3571451	NE				-				phage protein
M5005_Spy_1454c	3571452	NE				-				phage protein
M5005_Spy_1455c	3571453	NC				-				phage protein
M5005_Spy_1456c	3571454	NC				-				phage protein
M5005_Spy_1457c	3571455	NE				-				phage protein
M5005_Spy_1458c	3571456	NC				-				phage protein
M5005_Spy_1459c	3571419	NC				-				phage protein
M5005_Spy_1460c	3571420	NC				-				phage protein
M5005_Spy_1461c	3571421	NE				-				phage protein
M5005_Spy_1462	3571422	NE				-				phage protein
M5005_Spy_1463c	3571423	NC				-				phage protein
M5005_Spy_1464	3571424	E				-				Cro/CI family phage transcriptional regulator
M5005_Spy_1465	3571425	NE				-				phage protein
M5005_Spy_1466	3571426	NE				-				phage protein
M5005_Spy_1467	3571427	NE				int.3				integrase
M5005_Spy_1468c	3571429	NE	Spy49_1342c	6984645	NE	trmB	*			tRNA (guanine-N(7)-)-methyltransferase
M5005_Spy_1469c	3571430	E	Spy49_1343c	6984646	E	**-**	*	SSA_1903	SP_0549	phosphotransferase
M5005_Spy_1470c	3571431	NE	Spy49_1344c	6984647	NE	-	*			protein ecsB
M5005_Spy_1471c	3571432	NE	Spy49_1345c	6984648	NE	-	*			ABC transporter ATP-binding protein
M5005_Spy_1472	3571433	NE	Spy49_1346	6984649	NE	hit	*			bis(5\'-nucleosyl)-tetraphosphatase (asymmetrical)
M5005_Spy_1473	3571434	NE	Spy49_1347	6984650	NE	-	*			hypothetical protein M5005_Spy_1473
M5005_Spy_1474c	3571435	NE	Spy49_1348c	6984651	NE	lytR	*			LytR family transcriptional regulator
M5005_Spy_1475c	3571436	NE	Spy49_1349c	6984652	NE	-	*			acetyltransferase
M5005_Spy_1476c	3571437	NC	Spy49_1350c	6984653	NC	-				ATP/GTP hydrolase
M5005_Spy_1477c	3571400	NE	Spy49_1351c	6984654	NE	-	*			guanine-hypoxanthine permease
M5005_Spy_1478	3571401	NE	Spy49_1352	6984655	NE	-	*			HAD superfamily hydrolase
M5005_Spy_1479	3571402	NE	Spy49_1353	6984656	NE	manL	*			PTS system mannose-specific transporter subunit IIAB
M5005_Spy_1480	3571403	NE	Spy49_1354	6984657	NE	manM	*			PTS system mannose-specific transporter subunit IIC
M5005_Spy_1481	3571404	NE	Spy49_1355	6984658	NE	manN	*			PTS system mannose-specific transporter subunit IID
			Spy49_1356	6984659	NE					
M5005_Spy_1482	3571405	NE	Spy49_1357	6984660	NE	manO	*			hypothetical protein M5005_Spy_1482
M5005_Spy_1483	3571406	E	Spy49_1358	6984661	E	**serS**	*	SSA_1925	SP_0411	seryl-tRNA synthetase
M5005_Spy_1484c	3571407	E	Spy49_1359c	6984662	NC	accD	*	SSA_1930	SP_0427	acetyl-CoA carboxylase subunit alpha
M5005_Spy_1485c	3571408	E	Spy49_1360c	6984743	E	**accA**	*	SSA_1931	SP_0426	acetyl-CoA carboxylase subunit beta
M5005_Spy_1486c	3571409	E	Spy49_1361c	6984744	E	**accC**	*	SSA_1932		acetyl-CoA carboxylase biotin carboxylase subunit
M5005_Spy_1487c	3571410	C	Spy49_1362c	6984745	NC	fabZ	*	SSA_1933	SP_0424	(3R)-hydroxymyristoyl-ACP dehydratase
M5005_Spy_1488c	3571411	E	Spy49_1363c	6984746	NC	accB	*	SSA_1934	SP_0423	acetyl-CoA carboxylase biotin carboxyl carrier protein subunit
M5005_Spy_1489c	3571412	E	Spy49_1364c	6984747	E	**fabF**	*	SSA_1935	SP_0422	3-oxoacyl-ACP synthase
M5005_Spy_1490c	3571413	E	Spy49_1365c	6984748	E	**fabG**	*	SSA_1936	SP_0421	3-ketoacyl-ACP reductase
M5005_Spy_1491c	3571414	E	Spy49_1366c	6984749	E	**fabD**	*	SSA_1937	SP_0420	ACP S-malonyltransferase
M5005_Spy_1492c	3571415	C	Spy49_1367c	6984750	E	fabK	*	SSA_1938	SP_0419	enoyl-ACP reductase
M5005_Spy_1493c	3571416	E	Spy49_1368c	6984751	NC	acpP		SSA_1940	SP_0418	acyl carrier protein
M5005_Spy_1494c	3571417	E	Spy49_1369c	6984752	E	**fabH**	*	SSA_1940	SP_0417	3-oxoacyl-ACP synthase
M5005_Spy_1495c	3571418	NC	Spy49_1370c	6984753	NE	-	*			MarR family transcriptional regulator
M5005_Spy_1496c	3571380	E	Spy49_1371c	6984754	E	**phaB**	*		SP_0415	enoyl-CoA hydratase
M5005_Spy_1497c	3571381	E	Spy49_1372c	6984755	E	**dnaJ**	*	SSA_2005		molecular chaperone DnaJ
			Spy49_1373	6984756	NC					
M5005_Spy_1498c	3571382	E	Spy49_1374c	6984757	E	**dnaK**	*	SSA_2007	SP_0517	molecular chaperone DnaK
M5005_Spy_1499c	3571383	E	Spy49_1375c	6984758	E	**grpE**	*			heat shock protein GrpE
M5005_Spy_1500c	3571384	C	Spy49_1376c	6984759	NE	hrcA	*			heat-inducible transcription repressor
M5005_Spy_1501c	3571385	NE	Spy49_1377c	6984760	NE	-	*			N-acetylmuramoyl-L-alanine amidase
M5005_Spy_1502c	3571386	NE	Spy49_1378c	6984761	NE	-	*			D-alanyl-D-alanine carboxypeptidase
M5005_Spy_1503c	3571387	NE	Spy49_1379c	6984762	NE	-	*			phosphoglycerate mutase
M5005_Spy_1504c	3571388	NE	Spy49_1380c	6984763	NE	-				hypothetical protein M5005_Spy_1504
M5005_Spy_1505c	3571389	NE	Spy49_1381c	6984764	NE	-				hypothetical protein M5005_Spy_1505
M5005_Spy_1506c	3571390	E	Spy49_1382c	6984765	E	**gatB**	*	SSA_0571	SP_0436	aspartyl/glutamyl-tRNA amidotransferase subunit B
M5005_Spy_1507c	3571391	E	Spy49_1383c	6984766	E	**gatA**	*	SSA_0570	SP_0437	aspartyl/glutamyl-tRNA amidotransferase subunit A
M5005_Spy_1508c	3571392	E	Spy49_1384c	6984767	NC	gatC	*	SSA_0569	SP_0438	aspartyl/glutamyl-tRNA amidotransferase subunit C
M5005_Spy_1509c	3571393	NE	Spy49_1385c	6984768	NE	-				pyruvate, phosphate dikinase, partial
M5005_Spy_1510c	3571394	NC				-				pyruvate, phosphate dikinase
M5005_Spy_1511c	3571395	NE	Spy49_1386c	6984769	NE	-	*			pyrazinamidase/nicotinamidase
M5005_Spy_1512c	3571396	C	Spy49_1387c	6984770	E	codY			SP_1584	transcriptional repressor CodY
M5005_Spy_1513c	3571397	NE	Spy49_1388c	6984771	NE	-	*			aminotransferase
M5005_Spy_1514	3571398	NE	Spy49_1389	6984772	NE	-	*			universal stress protein
M5005_Spy_1515c	3571399	NE	Spy49_1390c	6984773	NE	-	*			HAD superfamily hydrolase
M5005_Spy_1516	3571361	NE	Spy49_1391	6984774	NE	asnB	*			L-asparaginase
M5005_Spy_1517c	3571362	NE	Spy49_1392c	6984775	NE	-	*			lantibiotic transport permease
M5005_Spy_1518c	3571363	NE	Spy49_1393c	6984776	NE	-	*			transporter
M5005_Spy_1519c	3571364	NE	Spy49_1394c	6984777	C	recG	*			ATP-dependent DNA helicase RecG
M5005_Spy_1520	3571365	NE				-				hypothetical protein M5005_Spy_1520
M5005_Spy_1521c	3571366	NE	Spy49_1395c	6984778	NE	-	*			cobalt ABC transporter ATP-binding protein
M5005_Spy_1522c	3571367	NE	Spy49_1396c	6984779	NE	-	*			cobalt transporter cbiQ
M5005_Spy_1523c	3571368	NE	Spy49_1397c	6984780	NE	-	*			permease
M5005_Spy_1524c	3571369	NE	Spy49_1398c	6984781	NE	cycC	*			ABC transporter ATP-binding protein
M5005_Spy_1525c	3571370	NE	Spy49_1400c	6984782	NE	cycD	*			ABC transporter ATP-binding protein
M5005_Spy_1526c	3571371	NE	Spy49_1401c	6984783	NE	fhuC	*			ferrichrome ABC transporter ATP-binding protein
M5005_Spy_1527c	3571372	NE	Spy49_1402c	6984784	NE	fhuB2	*			ferrichrome transporter permease
M5005_Spy_1528c	3571373	NE	Spy49_1403c	6984785	NE	fhuD2	*			ferrichrome-binding protein
M5005_Spy_1529c	3571374	NE				shp	*			heme binding protein
M5005_Spy_1530c	3571375	NE	Spy49_1405c	6984786	NE	-	*			Fe3+-siderophore transporter
M5005_Spy_1531c	3571376	NE	Spy49_1407c	6984787	NE	isp2	*			hypothetical protein M5005_Spy_1531
M5005_Spy_1532c	3571377	NC	Spy49_1408c	6984788	E	alr	*	SSA_0548	SP_1698	alanine racemase
M5005_Spy_1533c	3571378	NC	Spy49_1409c	6984789	E	acpS	*	SSA_0547	SP_1699	4\'-phosphopantetheinyl transferase
M5005_Spy_1534c	3571379	E	Spy49_1410c	6984790	E	**secA**	*	SSA_0543	SP_1702	preprotein translocase subunit SecA
M5005_Spy_1535c	3571341	NC				-				hypothetical protein M5005_Spy_1535
M5005_Spy_1536c	3571342	NE				-				transposase
M5005_Spy_1537c	3571343	NE				-				transposase, partial
M5005_Spy_1538c	3571344	NE	Spy49_1412c	6984791	NE	pmi	*			mannose-6-phosphate isomerase
M5005_Spy_1539c	3571345	NE	Spy49_1413c	6984792	NE	scrK	*			fructokinase
M5005_Spy_1540c	3571346	NE	Spy49_1414c	6984793	NE	endoS				endo-beta-N-acetylglucosaminidase F2
M5005_Spy_1541c	3571347	NC				-				hypothetical protein M5005_Spy_1541
M5005_Spy_1542c	3571348	NE	Spy49_1415c	6984794	NE	scrA	*			PTS system sucrose-specific transporter subunit IIABC
M5005_Spy_1543	3571349	NE	Spy49_1416	6984795	NC	scrB	*			sucrose-6-phosphate hydrolase
M5005_Spy_1544	3571350	NE	Spy49_1417	6984796	NE	scrR	*			sucrose operon repressor

M5005_Spy_1545c	3571351	C	Spy49_1418c	6984797	NE	nusB	*		SP_0433	transcription antitermination protein NusB
M5005_Spy_1546c	3571352	NC	Spy49_1419c	6984798	NE	-	*			Gls24 family general stress protein
M5005_Spy_1547c	3571353	NE	Spy49_1420c	6984799	NE	efp	*			elongation factor P
M5005_Spy_1548c	3571354	NE	Spy49_1421c	6984800	NE	comEB	*			competence protein ComE
M5005_Spy_1549c	3571355	NE	Spy49_1422c	6984801	NE	pepP	*			Xaa-Pro dipeptidase
M5005_Spy_1550c	3571356	NE	Spy49_1423c	6984802	NE	uvrA	*			excinuclease ABC subunit A
M5005_Spy_1551	3571357	E	Spy49_1424	6984803	C	corA	*		SP_0185	magnesium and cobalt transporter
M5005_Spy_1552	3571358	NE	Spy49_1425	6984804	NE	-	*			hypothetical protein M5005_Spy_1552
M5005_Spy_1553c	3571359	NC	Spy49_1426c	6984805	NC	rpsR				30S ribosomal protein S18
M5005_Spy_1554c	3571360	NC	Spy49_1427c	6984806	C	ssb3	*	SSA_0438	SP_1540	single-stranded DNA-binding protein
M5005_Spy_1555c	3571322	NC	Spy49_1428c	6984807	NC	rpsF	*			30S ribosomal protein S6
M5005_Spy_1556c	3571323	NE	Spy49_1429c	6984808	NE	-	*			hypothetical protein M5005_Spy_1556
M5005_Spy_1557	3571324	NE	Spy49_1430	6984809	NE	mutY	*			A/G-specific adenine glycosylase
M5005_Spy_1558	3571325	NE	Spy49_1431	6984810	NE	-				transcriptional regulator
M5005_Spy_1559c	3571326	NE	Spy49_1432c	6984811	NE	trx	*			thioredoxin
M5005_Spy_1560c	3571327	NE	Spy49_1433c	6984812	NE	-	*			phosphatidylglycerophosphatase B
M5005_Spy_1561c	3571328	NE	Spy49_1434c	6984813	NE	mutS2	*			DNA mismatch repair protein
M5005_Spy_1562c	3571329	NE	Spy49_1435c	6984814	NE	-	*			colicin V production protein
M5005_Spy_1563c	3571330	NE	Spy49_1436c	6984815	NE	-	*			hypothetical protein M5005_Spy_1563
M5005_Spy_1564	3571331	C	Spy49_1437	6984816	E	-	*			ribonuclease HIII
M5005_Spy_1565	3571332	E	Spy49_1438	6984817	E	**spi**	*		SP_0402	signal peptidase I
M5005_Spy_1566	3571333	NE	Spy49_1439	6984818	NE	recD	*			exodeoxyribonuclease V subunit alpha
M5005_Spy_1567	3571334	NE	Spy49_1440	6984819	NE	-	*			hypothetical protein M5005_Spy_1567
M5005_Spy_1568c	3571335	NE	Spy49_1441c	6984820	NC	dinP	*			DNA polymerase IV
M5005_Spy_1569	3571336	NE	Spy49_1442	6984821	NC	pfl	*			formate acetyltransferase
M5005_Spy_1570c	3571337	NE	Spy49_1443c	6984822	NE	-	*			penicillin-binding protein
M5005_Spy_1571c	3571338	NE	Spy49_1444c	6984903	NE	cppA	*			c3-degrading proteinase
M5005_Spy_1572	3571339	NE	Spy49_1445	6984904	NE	-	*			hypothetical protein M5005_Spy_1572
M5005_Spy_1573c	3571340	C	Spy49_1447c	6984905	NC	glpF.2	*		SP_1491	aquaporin
M5005_Spy_1574c	3571302	NC				-				universal stress protein
M5005_Spy_1575c	3571303	NE	Spy49_1449c	6984906	NE	norA	*			quinolone resistance protein
M5005_Spy_1576	3571304	NE	Spy49_1450	6984907	NE	srv	*			Crp/Fnr family transcriptional regulator
M5005_Spy_1577	3571305	NE	Spy49_1451	6984908	NE	pepXP	*			x-prolyl-dipeptidyl aminopeptidase
M5005_Spy_1578c	3571306	NE	Spy49_1452c	6984909	NE	-				Cro/CI family transcriptional regulator
M5005_Spy_1579	3571307	NE	Spy49_1453	6984910	NE	-	*			transcriptional regulator
			Spy49_1454c	6984911	NE					
			Spy49_1455	6984912	NE					
			Spy49_1456	6984913	NE					
			Spy49_1457c	6984914	NE					
			Spy49_1458c	6984915	NE					
			Spy49_1459c	6984916	NE					
			Spy49_1460c	6984917	NE					
			Spy49_1461c	6984918	NE					
			Spy49_1462c	6984919	NE					
			Spy49_1463c	6984920	NC					
			Spy49_1464c	6984921	NE					
			Spy49_1465c	6984922	NC					
			Spy49_1466c	6984923	NC					
			Spy49_1467c	6984924	NC					
			Spy49_1468c	6984925	NE					
			Spy49_1470c	6984926	NE					
			Spy49_1471c	6984927	NE					
			Spy49_1472c	6984928	NE					
			Spy49_1474c	6984929	NE					
			Spy49_1475c	6984930	NE					
			Spy49_1476c	6984931	NE					
			Spy49_1477c	6984932	NE					
			Spy49_1478c	6984933	NE					
			Spy49_1479c	6984934	NE					
			Spy49_1480c	6984935	NE					
			Spy49_1481c	6984936	NE					
			Spy49_1482c	6984937	NE					
			Spy49_1483c	6984938	NE					
			Spy49_1484c	6984939	NE					
			Spy49_1485c	6984940	NE					
			Spy49_1486c	6984941	NE					
			Spy49_1487c	6984942	NE					
			Spy49_1488c	6984943	NE					
			Spy49_1489c	6984944	NE					
			Spy49_1491c	6984945	NE					
			Spy49_1492c	6984946	NE					
			Spy49_1493c	6984947	NE					
			Spy49_1494c	6984948	NC					
			Spy49_1496c	6984949	NE					
			Spy49_1497c	6984950	NC					
			Spy49_1498c	6984951	NE					
			Spy49_1499c	6984952	NE					
			Spy49_1500c	6984953	NC					
			Spy49_1501c	6984954	NE					
			Spy49_1502c	6984955	NC					
			Spy49_1504c	6984956	NE					
			Spy49_1505c	6984957	NC					
			Spy49_1506c	6984958	NE					
			Spy49_1507c	6984959	NE					
			Spy49_1509c	6984960	NE					
			Spy49_1510c	6984961	NC					
			Spy49_1511c	6984962	NC					
			Spy49_1512c	6984963	NE					
			Spy49_1513c	6984964	NE					
			Spy49_1514c	6984965	NE					
			Spy49_1516c	6984966	NC					
			Spy49_1517c	6984967	NE					
			Spy49_1518c	6984968	NE					
			Spy49_1520c	6984969	NC					
			Spy49_1521c	6984970	NE					
			Spy49_1522	6984971	C					
			Spy49_1525	6984972	NE					
			Spy49_1526	6984973	NE					
			Spy49_1528c	6984974	NC					
			Spy49_1529	6984975	NC					
			Spy49_1530	6984976	NE					
			Spy49_1531	6984977	NE					

M5005_Spy_1580	3571308	NE	Spy49_1532	6984978	NE	-	*			hypothetical protein M5005_Spy_1580
M5005_Spy_1581c	3571309	NE	Spy49_1533c	6984979	NE	-	*			MerR family transcriptional regulator
M5005_Spy_1582c	3571310	NE	Spy49_1534c	6984980	NC	dnaQ	*			DNA polymerase III subunit epsilon
M5005_Spy_1583c	3571311	NE	Spy49_1535c	6984981	NC	-	*			hypothetical protein M5005_Spy_1583
			Spy49_1536c	6984982	NE					
			Spy49_1537c	6985063	NE					
M5005_Spy_1584	3571312	NE	Spy49_1538	6985064	NE	-	*			NAD(FAD)-utilizing dehydrogenase
M5005_Spy_1585c	3571313	NE	Spy49_1540c	6985065	NE	deoC	*			deoxyribose-phosphate aldolase
M5005_Spy_1586c	3571314	NE	Spy49_1541c	6985066	NE	nupC	*			nucleoside permease
M5005_Spy_1587c	3571315	NE	Spy49_1542c	6985067	NE	udp	*			uridine phosphorylase
M5005_Spy_1588c	3571316	NC				-				hypothetical protein M5005_Spy_1588
M5005_Spy_1589	3571317	NE	Spy49_1543	6985068	NE	crgR	*			GntR family transcriptional regulator
M5005_Spy_1590	3571318	NE	Spy49_1544	6985069	NE	rpsN	*			30S ribosomal protein S14
M5005_Spy_1591c	3571319	E	Spy49_1545c	6985070	NC	gcp	*		SP_0129	DNA-binding/iron metalloprotein/AP endonuclease
M5005_Spy_1592c	3571320	NE	Spy49_1546c	6985071	NE	-	*			ribosomal-protein-alanine acetyltransferase
M5005_Spy_1593c	3571321	C	Spy49_1547c	6985072	E	-	*			glycoprotease
M5005_Spy_1594	3571284	E	Spy49_1548	6985073	NC	-			SP_0122	hypothetical protein M5005_Spy_1594
M5005_Spy_1595	3571285	E	Spy49_1549	6985074	E	**-**	*	SSA_0312	SP_0121	Zn-dependent hydrolase
M5005_Spy_1596c	3571286	NE	Spy49_1550c	6985075	NE	glnA	*			glutamine synthetase
M5005_Spy_1597c	3571287	NE	Spy49_1551c	6985076	NE	glnR	*			MerR family transcriptional regulator
M5005_Spy_1598c	3571288	NE	Spy49_1552c	6985077	NE	-	*			hypothetical protein M5005_Spy_1598
M5005_Spy_1599c	3571289	E	Spy49_1553c	6985078	NC	pgk	*	SSA_0302	SP_0499	phosphoglycerate kinase
M5005_Spy_1600c	3571290	NE	Spy49_1554c	6985079	NE	lppC	*			acid phosphatase
M5005_Spy_1601c	3571291	NE	Spy49_1555c	6985080	NE	-				membrane protease
M5005_Spy_1602c	3571292	E	Spy49_1556c	6985081	E	**-**	*		SP_0443	kinase
M5005_Spy_1603c	3571293	NE	Spy49_1557c	6985082	NC	asp	*			alkaline-shock protein
M5005_Spy_1604c	3571294	NC				-				hypothetical protein M5005_Spy_1604
M5005_Spy_1605c	3571295	NE				-				transposase
M5005_Spy_1606c	3571296	NE	Spy49_1558c	6985083	NE	rpmB				50S ribosomal protein L28
M5005_Spy_1607c	3571297	E	Spy49_1559c	6985084	E	**fba**	*	SSA_1992	SP_0605	fructose-bisphosphate aldolase
M5005_Spy_1608c	3571299	NE	Spy49_1560c	6985086	NE	-	*			alpha/beta hydrolase
M5005_Spy_1610c	3571301	NE	Spy49_1561c	6985087	NE	pyrG	*			CTP synthase, partial
M5005_Spy_1611c	3571270	NE	Spy49_1562c	6985088	NE	rpoE	*			DNA-directed RNA polymerase subunit delta
M5005_Spy_1612c	3571271	NE	Spy49_1563c	6985089	NE	tig/ropA	*			trigger factor
M5005_Spy_1613	3571272	NE	Spy49_1564	6985090	NE	-	*			mechanosensitive ion channel MscS
M5005_Spy_1614c	3571273	NE	Spy49_1565c	6985091	NE	-	*			hypothetical protein M5005_Spy_1614
M5005_Spy_1615c	3571274	NE	Spy49_1566c	6985092	NE	-	*			hypothetical protein M5005_Spy_1615
M5005_Spy_1616c	3571275	NE	Spy49_1567c	6985093	NE	thiD	*			phosphomethylpyrimidine kinase
M5005_Spy_1617c	3571276	NE	Spy49_1568c	6985094	NE	truA	*			tRNA pseudouridine synthase A
M5005_Spy_1618c	3571277	NE	Spy49_1569c	6985095	NE	comX.2				competence-specific sigma factor
M5005_Spy_1619	3571251	NC				-				hypothetical protein M5005_Spy_1619
M5005_Spy_1620c	3571252	NE	Spy49_1571c	6985102	NE	-	*			glycerate kinase
M5005_Spy_1621	3571253	NE	Spy49_1572	6985103	NE	hsdR	*			type I restriction-modification system restriction subunit
M5005_Spy_1622	3571254	NE	Spy49_1573	6985104	NE	hsdS				type I restriction-modification system specificity subunit
M5005_Spy_1623	3571255	NE	Spy49_1574	6985105	NE	hsdM	*			type I restriction-modification system methylation subunit
M5005_Spy_1624c	3571256	NE	Spy49_1575c	6985106	NE	-				hypothetical protein M5005_Spy_1624
M5005_Spy_1625c	3571257	NE	Spy49_1576c	6985107	NE	salR	*			transcriptional regulatory protein
M5005_Spy_1626c	3571258	NE	Spy49_1577c	6985108	NE	salK				sensory transduction protein kinase
M5005_Spy_1627c	3571259	NE	Spy49_1578c	6985109	NE	salY				ABC transporter permease
M5005_Spy_1628c	3571260	NE	Spy49_1579c	6985110	NE	salX				ABC transporter ATP-binding protein
M5005_Spy_1629c	3571261	NE	Spy49_1580c	6985111	NE	salX/salT				lantibiotic transport ATP-binding protein
M5005_Spy_1630c	3571262	NE	Spy49_1582c	6985112	NE	salB				serine (threonine) dehydratase
M5005_Spy_1631c	3571263	NC	Spy49_1583c	6985113	NC	salA				lantibiotic salivaricin A
M5005_Spy_1632c	3571264	NE	Spy49_1584c	6985114	NE	lacG	*			6-phospho-beta-galactosidase
M5005_Spy_1633c	3571265	NE	Spy49_1585c	6985115	NE	lacE				PTS system lactose-specific transporter subunit IIBC
M5005_Spy_1634c	3571266	NE	Spy49_1586c	6985116	NE	lacF	*			PTS system lactose-specific transporter subunit IIA
M5005_Spy_1635c	3571267	NE	Spy49_1587c	6985117	NE	lacD.2	*			tagatose 1,6-diphosphate aldolase
M5005_Spy_1636c	3571268	NE	Spy49_1588c	6985118	NE	lacC.2	*			tagatose-6-phosphate kinase
M5005_Spy_1637c	3571269	NE	Spy49_1589c	6985119	NE	lacB.2	*			galactose-6-phosphate isomerase subunit LacB
M5005_Spy_1638c	3571231	NE	Spy49_1590c	6985120	NE	lacA.2	*			galactose-6-phosphate isomerase subunit LacA
M5005_Spy_1639	3571232	NE	Spy49_1591	6985121	NE	lacR.2	*			lactose phosphotransferase system repressor
M5005_Spy_1640	3571233	NE	Spy49_1592	6985122	NE	-				DNA-damage-inducible protein J
M5005_Spy_1641	3571234	NE	Spy49_1593	6985123	NE	-	*			hypothetical protein M5005_Spy_1641
M5005_Spy_1642	3571235	NE	Spy49_1594	6985124	NE	-				DNA integration/recombination/invertion protein
M5005_Spy_1643	3571237	NC				-				DNA integration/recombination/invertion protein
M5005_Spy_1644c	3571236	NC				-				hypothetical protein M5005_Spy_1644
M5005_Spy_1645	3571238	NC				-				DNA integration/recombination/invertion protein
M5005_Spy_1646c	3571239	NC	Spy49_1595c	6985125	E	rpsI	*		SP_0295	30S ribosomal protein S9
M5005_Spy_1647c	3571240	NC	Spy49_1596c	6985126	NC	rplM	*			50S ribosomal protein L13
M5005_Spy_1648c	3571241	NC	Spy49_1597c	6985127	NC	-				Cro/CI family transcriptional regulator
M5005_Spy_1649c	3571242	NE	Spy49_1598c	6985128	NE	-	*			hypothetical protein M5005_Spy_1649
M5005_Spy_1650c	3571243	NE	Spy49_1599c	6985129	NC	-	*			degV family protein
M5005_Spy_1651c	3571244	NE	Spy49_1600c	6985130	NE	-	*			hypothetical protein M5005_Spy_1651
M5005_Spy_1652c	3571245	NE	Spy49_1601c	6985131	NE	-	*			23S rRNA methyltransferase
M5005_Spy_1653c	3571246	NE	Spy49_1602c	6985132	NE	-	*			hypothetical protein M5005_Spy_1653
M5005_Spy_1654c	3571247	NE	Spy49_1603c	6985133	NE	-	*			hypothetical protein M5005_Spy_1654
M5005_Spy_1655c	3571248	E	Spy49_1604c	6985134	E	**cysS**	*	SSA_2044	SP_0591	cysteinyl-tRNA synthetase
M5005_Spy_1656c	3571249	NE	Spy49_1605c	6985135	NE	-				hypothetical protein M5005_Spy_1656
M5005_Spy_1657c	3571250	NE				-				hypothetical protein M5005_Spy_1657
M5005_Spy_1658c	3571212	NC	Spy49_1606c	6985136	NC	cysE	*			serine acetyltransferase
M5005_Spy_1659c	3571213	NE	Spy49_1607c	6985137	NE	-	*			hypothetical protein M5005_Spy_1659
M5005_Spy_1660c	3571214	NE	Spy49_1608c	6985138	NE	pnp	*			polynucleotide phosphorylase
M5005_Spy_1661c	3571215	NE	Spy49_1609c	6985139	NE	-	*			translaldolase
M5005_Spy_1662c	3571216	NE	Spy49_1610c	6985140	NE	ulaA	*			PTS system ascorbate-specific transporter subunit IIC
M5005_Spy_1663c	3571217	NE	Spy49_1611c	6985141	NE	-	*			PTS system transporter subunit IIB
M5005_Spy_1664c	3571218	NE	Spy49_1612c	6985142	NE	-	*			PTS system, mannitol (cryptic)-specific IIA component
M5005_Spy_1665c	3571219	NC				-				hypothetical protein M5005_Spy_1665
M5005_Spy_1666c	3571220	NC	Spy49_1613c	6985223	NE	rpsO				30S ribosomal protein S15
M5005_Spy_1667c	3571221	NC	Spy49_1614c	6985224	NE	-				hypothetical protein M5005_Spy_1667
M5005_Spy_1668	3571222	NE				-				transcriptional regulator
M5005_Spy_1669	3571223	E	Spy49_1615	6985225	C	def	*	SSA_2061	SP_1456	peptide deformylase
M5005_Spy_1670c	3571224	NE	Spy49_1616c	6985226	NE	-				oxidoreductase
M5005_Spy_1671c	3571225	NE	Spy49_1617c	6985227	NE	-	*			MarR family transcriptional regulator
M5005_Spy_1672c	3571226	E	Spy49_1618c	6985228	E	**polC**	*	SSA_2066	SP_0274	DNA polymerase III PolC
M5005_Spy_1673c	3571227	E	Spy49_1619c	6985229	E	**proS**	*	SSA_2069	SP_0264	prolyl-tRNA synthetase
M5005_Spy_1674c	3571228	NE	Spy49_1620c	6985230	NE	eep	*			pheromone-processing membrane metalloprotease
M5005_Spy_1675c	3571229	E	Spy49_1621c	6985231	E	**cdsA**	*	SSA_2072	SP_0262	phosphatidate cytidylyltransferase
M5005_Spy_1676c	3571230	E	Spy49_1622c	6985232	E	**uppS**	*	SSA_2073	SP_0261	undecaprenyl pyrophosphate synthase
M5005_Spy_1677c	3571192	NE	Spy49_1624c	6985233	NE	yajC	*			preprotein translocase subunit YajC
M5005_Spy_1678c	3571193	NE	Spy49_1625c	6985234	NE	-	*			thioredoxin
M5005_Spy_1680c	3571195	NE	Spy49_1626c	6985235	NE	pulA	*			pullulanase, partial
M5005_Spy_1681c	3571196	NE	Spy49_1627c	6985236	NE	dexB	*			glucan 1,6-alpha-glucosidase

M5005_Spy_1682c	3571197	NE	Spy49_1628c	6985237	NE	msmK	*			multiple sugar transport ATP-binding protein
M5005_Spy_1683c	3571198	NE	Spy49_1629c	6985238	NE	lrp	*			hypothetical protein M5005_Spy_1683
M5005_Spy_1684	3571199	NE	Spy49_1630	6985239	NE	ska				streptokinase
M5005_Spy_1685c	3571200	NE	Spy49_1631c	6985240	NE	dtd	*			D-tyrosyl-tRNA(Tyr) deacylase
M5005_Spy_1686c	3571201	E	Spy49_1632c	6985241	C	relA	*			GTP pyrophosphokinase
M5005_Spy_1687c	3571202	NE	Spy49_1633c	6985242	NE	sclA				hypothetical protein M5005_Spy_1687
M5005_Spy_1690	3571205	NE	Spy49_1634	6985243	NE	nrdI2				flavoprotein NrdI
M5005_Spy_1691c	3571206	NE	Spy49_1635c	6985244	NE	-	*			exodeoxyribonuclease III
M5005_Spy_1693c	3571208	NE	Spy49_1636c	6985245	NE	-	*			PTS system glucose-specific transporter subunit IIABC
M5005_Spy_1694c	3571209	NE	Spy49_1637c	6985246	NE	-	*			16S ribosomal RNA methyltransferase RsmE
M5005_Spy_1695c	3571210	NE	Spy49_1638c	6985247	NE	prmA	*			50S ribosomal protein L11 methyltransferase
M5005_Spy_1696c	3571211	NE	Spy49_1639c	6985248	NE	-	*			hypothetical protein M5005_Spy_1696
			Spy49_1640c	6985249	NE					
			Spy49_1641c	6985250	NE					
			Spy49_1642c	6985251	NE					
			Spy49_1643c	6985252	NC					
			Spy49_1644c	6985253	NE					
			Spy49_1646c	6985254	NE					
			Spy49_1647c	6985255	NE					
			Spy49_1649c	6985256	NE					
			Spy49_1651	6985257	NE					
			Spy49_1652	6985258	NC					
M5005_Spy_1697	3571174	NE	Spy49_1653	6985259	NE	papB	*			para-aminobenzoate synthetase component I/4-amino-4-deoxychorismate lyase
M5005_Spy_1698	3571175	NC	Spy49_1654	6985260	NE	trpG	*			anthranilate synthase component II
M5005_Spy_1699	3571176	NE	Spy49_1655	6985261	NE	-	*			recombination factor protein RarA
M5005_Spy_1700	3571178	NE	Spy49_1656	6985263	NE	pai1				acetyltransferase
M5005_Spy_1701	3571179	NE	Spy49_1657	6985264	NE	flaR				topology modulation protein
M5005_Spy_1702	3571180	NE	Spy49_1658c	6985265	NE	smeZ				mitogenic exotoxin Z
M5005_Spy_1703c	3571181	NE	Spy49_1659c	6985266	NE	-				hypothetical protein M5005_Spy_1703
M5005_Spy_1704	3571182	NE	Spy49_1660	6985267	NE	dppA	*			dipeptide-binding protein
M5005_Spy_1705	3571183	NE	Spy49_1661	6985268	NE	dppB	*			dipeptide transporter permease
M5005_Spy_1706	3571184	NE	Spy49_1662	6985269	NE	dppC	*			dipeptide transporter permease
M5005_Spy_1707	3571185	NE	Spy49_1663	6985270	NE	dppD	*			dipeptide transport ATP-binding protein
M5005_Spy_1708	3571186	NE	Spy49_1664	6985271	NE	dppE	*			dipeptide transport ATP-binding protein
M5005_Spy_1709c	3571187	NE	Spy49_1665c	6985272	NE	-	*			hypothetical protein M5005_Spy_1709
M5005_Spy_1710c	3571188	NE	Spy49_1666c	6985273	NE	-	*			histidine triad protein
M5005_Spy_1711c	3571189	NE	Spy49_1667c	6985274	NE	lmb	*			laminin binding protein
M5005_Spy_1712c	3571190	NE				-				transposase
M5005_Spy_1713c	3571191	NC				-				hypothetical protein M5005_Spy_1713
M5005_Spy_1714c	3571154	NE	Spy49_1668c	6985275	NE	-				cell surface protein
M5005_Spy_1715c	3571155	NE	Spy49_1669c	6985276	NE	scpA	*			C5A peptidase
M5005_Spy_1716	3571156	NE	Spy49_1670c	6985277	NE	-				transposase
M5005_Spy_1717	3571157	NC				-				transposase, partial
M5005_Spy_1718c	3571158	NE	Spy49_1671c	6985278	NE	sic1.01				inhibitor of complement protein
M5005_Spy_1719c	3571159	NE	Spy49_1672c	6985279	NE	emm1.0				M protein
M5005_Spy_1720c	3571160	NE	Spy49_1673c	6985280	NE	mga				trans-acting positive regulator
M5005_Spy_1721	3571161	NC	Spy49_1674c	6985281	NE	-				hypothetical protein M5005_Spy_1721
M5005_Spy_1722c	3571162	NE				-				hypothetical protein M5005_Spy_1722
M5005_Spy_1723c	3571163	NE	Spy49_1675c	6985282	NE	isp	*			hypothetical protein M5005_Spy_1723
M5005_Spy_1724c	3571164	NE	Spy49_1676c	6985283	NE	ihk	*			two component system histidine kinase
M5005_Spy_1725c	3571165	NE	Spy49_1677c	6985284	NE	irr	*			two-component response regulator
M5005_Spy_1726c	3571166	NE	Spy49_1678c	6985285	NE	-	*			ABC transporter permease
M5005_Spy_1727c	3571167	NE	Spy49_1679c	6985286	NE	-	*			ABC transporter ATP-binding protein
M5005_Spy_1728c	3571168	NE	Spy49_1680c	6985287	NE	-	*			periplasmic protein of efflux system
M5005_Spy_1729c	3571169	NE	Spy49_1681c	6985288	NE	-	*			hypothetical protein M5005_Spy_1729
M5005_Spy_1730c	3571170	NE	Spy49_1682c	6985289	NE	-	*			hypothetical protein M5005_Spy_1730
M5005_Spy_1731	3571171	NE	Spy49_1683c	6985290	NE	grm				hypothetical protein M5005_Spy_1731
			Spy49_1684c	6985291	NE					
			Spy49_1686	6985292	NE					
M5005_Spy_1732c	3571172	C	Spy49_1687c	6985293	NE	prsA	*			foldase PrsA
M5005_Spy_1733c	3571173	NE	Spy49_1688c	6985294	NE	-				hypothetical protein M5005_Spy_1733
M5005_Spy_1734c	3571135	NE	Spy49_1689c	6985295	C	-				hypothetical protein M5005_Spy_1734
M5005_Spy_1735c	3571136	NE	Spy49_1690c	6985296	NE	speB	*			exotoxin B
M5005_Spy_1736	3571137	NC				-				hypothetical protein M5005_Spy_1736
M5005_Spy_1737	3571138	NE	Spy49_1691	6985297	NE	rgg	*			transcriptional regulator
M5005_Spy_1738c	3571139	NE	Spy49_1692c	6985298	NE	spd	*			phage-associated deoxyribonuclease
M5005_Spy_1739	3571140	NC	Spy49_1693	6985299	NC	-				hypothetical protein M5005_Spy_1739
M5005_Spy_1740	3571141	NE	Spy49_1694	6985300	NE	-	*			low temperature requirement C protein
M5005_Spy_1741c	3571142	NE	Spy49_1695c	6985301	NE	gldA	*			glycerol dehydrogenase
M5005_Spy_1742c	3571143	NE	Spy49_1696c	6985302	NE	mipB	*			fructose-6-phosphate aldolase
M5005_Spy_1743c	3571144	NE	Spy49_1697c	6985383	NE	pflD	*			formate acetyltransferase
M5005_Spy_1744c	3571145	NE	Spy49_1698c	6985384	NE	-	*			PTS system cellobiose-specific transporter subunit IIC
M5005_Spy_1745c	3571146	NE	Spy49_1699c	6985385	NE	-	*			PTS system cellobiose-specific transporter subunit IIB
M5005_Spy_1746c	3571147	NE	Spy49_1700c	6985386	NE	-	*			PTS system cellobiose-specific transporter subunit IIA
M5005_Spy_1747c	3571148	NE	Spy49_1701c	6985387	NE	-	*			sorbitol operon regulator
M5005_Spy_1748c	3571149	NE	Spy49_1702c	6985388	NE	-	*			DeoR family transcriptional regulator
M5005_Spy_1749	3571150	NE	Spy49_1703	6985389	NE	-	*			pyruvate formate-lyase activating enzyme
M5005_Spy_1750	3571151	NE				-				hypothetical protein M5005_Spy_1750
M5005_Spy_1751c	3571152	NC	Spy49_1704c	6985390	NC	secE				preprotein translocase subunit SecE
M5005_Spy_1752c	3571153	NC	Spy49_1705c	6985391	NC	rpmG				50S ribosomal protein L33
M5005_Spy_1753c	3571116	NE	Spy49_1706c	6985392	NC	pbp2A	*			multimodular transpeptidase-transglycosylase
M5005_Spy_1754c	3571117	NE	Spy49_1707c	6985393	NE	-	*			translation initiation inhibitor
M5005_Spy_1755	3571118	NE	Spy49_1708	6985394	NE	-				hypothetical protein M5005_Spy_1755
M5005_Spy_1756	3571119	NE	Spy49_1709	6985395	NE	-	*			ribosomal large subunit pseudouridine synthase D
M5005_Spy_1757c	3571120	NE	Spy49_1710c	6985396	NE	-	*			hypothetical protein M5005_Spy_1757
M5005_Spy_1758	3571121	NE	Spy49_1713	6985397	NE	-	*			dipeptidase B
M5005_Spy_1759c	3571122	NE	Spy49_1714c	6985398	NE	-				MutR family transcriptional regulator
M5005_Spy_1760c	3571123	NE				-				MutR family transcriptional regulator
M5005_Spy_1761c	3571124	E	Spy49_1715c	6985399	E	**groEL**	*	SSA_0226	SP_1906	molecular chaperone GroEL
M5005_Spy_1762c	3571125	E	Spy49_1716c	6985400	NC	groES	*	SSA_0225		co-chaperonin GroES
M5005_Spy_1763c	3571126	NE	Spy49_1717c	6985401	NE	clpC	*			negative regulator of genetic competence
M5005_Spy_1764c	3571127	NE	Spy49_1718c	6985402	NE	ctsR	*			transcriptional regulator
M5005_Spy_1765c	3571128	NE	Spy49_1719c	6985403	NE	csp				cold shock protein
M5005_Spy_1766c	3571129	NC				-				hypothetical protein M5005_Spy_1766
M5005_Spy_1767c	3571130	NE	Spy49_1720c	6985405	NE	-				transposase
M5005_Spy_1768	3571132	NE	Spy49_1721	6985406	NE	ahpC	*			peroxiredoxin reductase (NAD(P)H)
M5005_Spy_1769	3571133	NE	Spy49_1722	6985407	NE	ahpF	*			peroxiredoxin reductase (NAD(P)H)
M5005_Spy_1770c	3571134	NE	Spy49_1723c	6985408	NE	hutI	*			imidazolonepropionase
M5005_Spy_1771	3571097	NE	Spy49_1724	6985409	NE	hutU	*			urocanate hydratase
M5005_Spy_1772	3571098	NE	Spy49_1725	6985410	NE	-	*			glutamate formiminotransferase
M5005_Spy_1773	3571099	NE	Spy49_1726	6985411	NE	-	*			formiminotetrahydrofolate cyclodeaminase

M5005_Spy_1774	3571100	NE	Spy49_1727	6985412	NE	fhs.2	*			formate--tetrahydrofolate ligase
M5005_Spy_1775	3571101	NE	Spy49_1728	6985413	NE	-	*			hypothetical protein M5005_Spy_1775
M5005_Spy_1776	3571102	NE	Spy49_1729	6985414	NE	-	*			amino acid permease
M5005_Spy_1777	3571103	NE	Spy49_1730	6985415	NE	hutH	*			histidine ammonia-lyase
M5005_Spy_1778	3571104	NE	Spy49_1731	6985416	NE	hutG	*			formimidoylglutamase
M5005_Spy_1779c	3571105	NE	Spy49_1732c	6985417	NE	-	*			LuxR family transcriptional regulator
M5005_Spy_1780	3571106	E	Spy49_1733	6985418	E	**rpsB**	*	SSA_2203	SP_2215	30S ribosomal protein S2
M5005_Spy_1781	3571107	E	Spy49_1734	6985419	E	**tsf**	*	SSA_2202	SP_2214	elongation factor Ts
M5005_Spy_1782c	3571108	NE	Spy49_1735c	6985420	NC	pepO	*			neutral endopeptidase
M5005_Spy_1783c	3571109	NC	Spy49_1736c	6985421	E	dexS	*		SP_1883	trehalose-6-phosphate hydrolase
M5005_Spy_1784c	3571110	NE	Spy49_1737c	6985422	NC	-	*			PTS system, trehalose-specific IIBC component
M5005_Spy_1785	3571111	NE	Spy49_1738	6985423	NE	-	*			trehalose operon transcriptional repressor
M5005_Spy_1786c	3571112	NE	Spy49_1739c	6985424	NE	-				MarR family transcriptional regulator, partial
M5005_Spy_1787	3571113	NE	Spy49_1740	6985425	NE	-				glyoxalase
M5005_Spy_1788	3571114	NE	Spy49_1742	6985426	NE	yaaA	*			hypothetical protein M5005_Spy_1788
M5005_Spy_1789c	3571115	NE	Spy49_1743c	6985427	NC	nrdG	*			anaerobic ribonucleoside-triphosphate reductase activating protein
M5005_Spy_1790c	3571077	NE	Spy49_1744c	6985428	NE	-	*			acetyltransferase
M5005_Spy_1791c	3571078	NC	Spy49_1745c	6985429	E	-	*			virulence factor
M5005_Spy_1792c	3571079	NC	Spy49_1746c	6985430	NC	-				hypothetical protein M5005_Spy_1792
M5005_Spy_1793c	3571080	C	Spy49_1747c	6985431	NC	nrdD	*			anaerobic ribonucleoside triphosphate reductase
M5005_Spy_1794c	3571081	NE	Spy49_1748c	6985432	NE	-	*			hypothetical protein M5005_Spy_1794
M5005_Spy_1795c	3571082	NE	Spy49_1749c	6985433	NE	-	*			hypothetical protein M5005_Spy_1795
M5005_Spy_1796c	3571083	E	Spy49_1750c	6985434	NC	-	*	SSA_2240		Holliday junction resolvase-like protein
M5005_Spy_1797c	3571084	NE	Spy49_1751c	6985435	NE	-				hypothetical protein M5005_Spy_1797
M5005_Spy_1798c	3571085	C	Spy49_1752c	6985436	NE	spxA	*		SP_1405	Spx family transcriptional regulator
M5005_Spy_1799c	3571086	NE	Spy49_1753c	6985437	NC	recA	*			recombinase A
M5005_Spy_1800c	3571087	NE	Spy49_1754c	6985438	NE	cinA	*			competence damage-inducible protein A
M5005_Spy_1801c	3571088	NC	Spy49_1755c	6985439	NE	tag	*			DNA-3-methyladenine glycosylase
M5005_Spy_1802c	3571089	NC	Spy49_1756c	6985440	C	ruvA	*		SP_0179	Holliday junction DNA helicase RuvA
M5005_Spy_1803c	3571090	NE	Spy49_1757c	6985441	C	lmrP	*			multidrug resistance protein B
M5005_Spy_1804c	3571091	NC	Spy49_1758c	6985442	C	mutL	*			DNA mismatch repair protein
M5005_Spy_1805c	3571092	NC	Spy49_1759c	6985443	NC	mutS	*			DNA mismatch repair protein MutS
M5005_Spy_1806c	3571093	NE				-				hypothetical protein M5005_Spy_1806
M5005_Spy_1807c	3571094	NE	Spy49_1761c	6985444	NE	argR2	*			arginine repressor ArgR
M5005_Spy_1808	3571095	E	Spy49_1762	6985445	E	**argS**	*	SSA_2262	SP_2078	arginyl-tRNA synthetase
M5005_Spy_1809	3571096	NE	Spy49_1763	6985446	NE	uviB	*			bacteriocin
M5005_Spy_1810c	3571058	NE	Spy49_1764c	6985447	NE	-	*			hypothetical protein M5005_Spy_1810
M5005_Spy_1811c	3571059	NE	Spy49_1765c	6985448	NE	-	*			hypothetical protein M5005_Spy_1811
M5005_Spy_1812c	3571060	NE	Spy49_1767c	6985449	NE	-	*			hypothetical protein M5005_Spy_1812
M5005_Spy_1813c	3571061	E	Spy49_1768c	6985450	E	**aspS**	*		SP_2114	aspartyl-tRNA synthetase
M5005_Spy_1814c	3571062	E	Spy49_1769c	6985451	E	**hisS**	*	SSA_2284	SP_2121	histidyl-tRNA synthetase
M5005_Spy_1815	3571063	NE	Spy49_1770	6985452	NE	rpmF				50S ribosomal protein L32
M5005_Spy_1816	3571064	NC	Spy49_1771	6985453	NC	rpmG				50S ribosomal protein L33
			Spy49_1772c	6985454	NE					
M5005_Spy_1817	3571065	NE	Spy49_1773	6985455	NE	cadD	*			cadmium resistance protein
M5005_Spy_1818	3571066	NE	Spy49_1774	6985456	NE	cadC	*			cadmium efflux system accessory protein
M5005_Spy_1819	3571067	NE	Spy49_1775	6985457	NE	-				hypothetical protein M5005_Spy_1819
M5005_Spy_1820	3571068	NE	Spy49_1777	6985458	NE	-	*			DNA translocase FtsK
M5005_Spy_1821	3571069	NE	Spy49_1778	6985459	NE	-	*			hypothetical protein M5005_Spy_1821
M5005_Spy_1822c	3571070	NE				-				transcriptional regulator
M5005_Spy_1823	3571071	NE	Spy49_1779	6985460	NE	-	*			hypothetical protein M5005_Spy_1823
M5005_Spy_1824c	3571072	NE	Spy49_1780c	6985461	NE	-				phosphohydrolase
M5005_Spy_1825	3571073	NE	Spy49_1781	6985462	NE	-	*			PadR family transcriptional regulator
M5005_Spy_1826	3571074	NE	Spy49_1782	6986105	NE	-	*			hypothetical protein M5005_Spy_1826
M5005_Spy_1827	3571075	NE	Spy49_1783	6986106	NE	-	*			hypothetical protein M5005_Spy_1827
M5005_Spy_1828c	3571076	NE	Spy49_1784c	6986107	NC	-	*			phage infection protein
M5005_Spy_1830	3571039	NE	Spy49_1785	6986108	NE	-	*			TetR family transcriptional regulator
M5005_Spy_1831c	3571040	E	Spy49_1786c	6986109	E	**rpsD**	*	SSA_2350	SP_0085	30S ribosomal protein S4
M5005_Spy_1832c	3571041	NC				-				hypothetical protein M5005_Spy_1832
M5005_Spy_1833c	3571042	NE				-				transposase
M5005_Spy_1834c	3571043	NE	Spy49_1787c	6986110	NE	-	*			hypothetical protein M5005_Spy_1834
M5005_Spy_1835c	3571044	E	Spy49_1788c	6986111	E	**holB/dnaC**	*	SSA_2356	SP_2203	replicative DNA helicase
M5005_Spy_1836c	3571045	NE	Spy49_1789c	6986112	NC	rplI	*			50S ribosomal protein L9
M5005_Spy_1837c	3571046	NE	Spy49_1790c	6986113	E	-	*			phosphoesterase, DHH family protein
M5005_Spy_1838c	3571047	C	Spy49_1791c	6986114	E	gidA	*		SP_0120	tRNA uridine 5-carboxymethylaminomethyl modification protein GidA
M5005_Spy_1839c	3571048	NE	Spy49_1792c	6986115	NE	-				phosphohydrolase
M5005_Spy_1840c	3571049	E	Spy49_1793c	6986116	E	**mnmA**	*	SSA_2360	SP_0118	tRNA-specific 2-thiouridylase MnmA
M5005_Spy_1841	3571050	NE	Spy49_1794	6986117	NE	sdhB	*			L-serine dehydratase
M5005_Spy_1842	3571051	NE	Spy49_1795	6986118	NE	sdhA	*			L-serine dehydratase
M5005_Spy_1843c	3571052	NE	Spy49_1796c	6986119	NE	-	*			transglycosylase
M5005_Spy_1844c	3571053	C	Spy49_1797c	6986120	E	cbiQ	*			cobalt transporter
M5005_Spy_1845c	3571054	E	Spy49_1798c	6986121	E	**cbiO**	*			cobalt ABC transporter ATP-binding protein
M5005_Spy_1846c	3571055	E	Spy49_1799c	6986122	E	**cbiO**	*			cobalt ABC transporter ATP-binding protein
M5005_Spy_1847c	3571056	NC	Spy49_1800c	6986123	E	pgsA	*	SSA_2368		CDP-diacylglycerol--glycerol-3-phosphate 3-phosphatidyltransferase
M5005_Spy_1848c	3571057	NE	Spy49_1801c	6986124	NE	-	*			hypothetical protein M5005_Spy_1848
M5005_Spy_1849c	3571021	NE	Spy49_1802c	6986125	NE	-	*			zinc protease
M5005_Spy_1850c	3571022	NE	Spy49_1803c	6986126	NE	-	*			zinc protease
M5005_Spy_1851	3571023	NE	Spy49_1804	6986127	NE	hasA				hyaluronan synthase
M5005_Spy_1852	3571024	NE	Spy49_1806	6986128	NE	hasB				UDP-glucose 6-dehydrogenase
M5005_Spy_1853	3571025	NE	Spy49_1808	6986129	NE	hasC				UTP-glucose-1-phosphate uridylyltransferase
M5005_Spy_1854	3571026	NE	Spy49_1809	6986130	NE	-	*			hypothetical protein M5005_Spy_1854
M5005_Spy_1855	3571027	NE	Spy49_1810	6986131	NE	recF	*			recombination protein F
M5005_Spy_1856c	3571028	NE	Spy49_1811c	6986132	NE	-	*			glucose uptake protein
M5005_Spy_1857c	3571029	NE	Spy49_1812c	6986133	NE	guaB	*			inosine 5\'-monophosphate dehydrogenase
M5005_Spy_1858c	3571030	E	Spy49_1813c	6986134	E	**trsA**	*	SSA_2375	SP_2229	tryptophanyl-tRNA synthetase
M5005_Spy_1859c	3571031	NC				-				hypothetical protein M5005_Spy_1859
M5005_Spy_1860	3571032	NE	Spy49_1814	6986135	NE	-	*			hypothetical protein M5005_Spy_1860
M5005_Spy_1861	3571033	NE	Spy49_1815	6986136	NE	-	*			ABC transporter ATP-binding protein
M5005_Spy_1862	3571034	NE	Spy49_1817	6986137	NE	-	*			ABC transporter permease
M5005_Spy_1863c	3571037	NE				-				transposase
M5005_Spy_1864c	3571018	NE	Spy49_1820c	6986141	NE	-	*			rRNA large subunit methyltransferase
M5005_Spy_1865	3571019	C	Spy49_1821	6986142	E	htrA	*			protease Do
M5005_Spy_1866	3571020	NE	Spy49_1822	6986143	NE	parB				chromosome partitioning protein

Table S8. Bacterial strains and plasmids.

Strains and plasmids	Description	Reference our source
***E. coli* strains**		
DH5α	*hsdR17 recA1 gyrA endA1 relA1*	Hanahan and Meselson, 1983
C43[DE3]	F^{-} *ompT gal dcm hsdS*$_B$(r_B^- m_B^-)(DE3)	Miroux and Walker, 1996
***S. pyogenes* strains**		
5448	M1T1	Chatellier *et al.*, 2000
NZ131	M49	Simon and Ferretti, 1991
5448.*vicR*$_{ind}$	5448, theophylline-inducible expression of *vicR*; Sp^R	This study
5448.*murE*$_{ind}$	5448, theophylline-inducible expression of *murE* Sp^R	This study
NZ131.*vicR*$_{ind}$	NZ131, theophylline-inducible expression of *vicR*; Sp^R	This study
NZ131.*murE*$_{ind}$	NZ131, theophylline-inducible expression of *murE*; Sp^R	This study
**Plasmids**		
pOSKAR	*Oskar*-delivery plasmid; Sp^R, Km^R	Le Breton et al., 2013
pKRMIT	*Krmit*-delivery plasmid; Sp^R, Km^R	This study
pCRS	Temperature-sensitive conditional vector; Sp^R	Le Breton et al. 2013
pSinS	Suicide plasmid for stable insertional inactivation; Sp^R	This study
pCRK	Temperature-sensitive conditional vector; Km^R	Le Breton et al. 2013
pHlpK	Temperature-sensitive conditional helper vector; Km^R	This study
pEU7742-E	Plasmid containing the fusion of the P*sag* promoter with the synthetic ribosiwtch E	Topp *et al.*, 2010
pSinMurE	Suicid plasmid for mutagenesis and conditional expression control of *murE*	This study
pSinVicR	Suicid plasmid for mutagenesis and conditional expression control of *vicR*	This study

Table S9. Primers used in this study.

Name and main purpose	Sequence (5'- -3')	Restriction site(s)/Barcode	Target	Role
**Construction of the *Krmit* transposon**				
oKrmit1	cccCTGCAGTAACAGGTTGG**A**TGATAAGTCCCCGGTCTG	*Pst*I/*Mme*I	pOSKAR	*Mme*I modification of *Oskar* to create *Krmit*
**Production of *Krmit* insertion tags**				
Adapterbarcode501A	TTCCCTACACGACGCTCTTCCGATCTTATAGCCTNN	Adapter501		*Mme*I Tnseq adapter
Adapterbarcode501B	AGGCTATAAGATCGGAAGAGCGTCGTGTAGGGAAAGAG	Adapter501		*Mme*I Tnseq adapter
Adapterbarcode502A	TTCCCTACACGACGCTCTTCCGATCTATAGAGGCNN	Adapter502		*Mme*I Tnseq adapter
Adapterbarcode502B	GCCTCTATAGATCGGAAGAGCGTCGTGTAGGGAAAGAG	Adapter502		*Mme*I Tnseq adapter
Adapterbarcode503A	TTCCCTACACGACGCTCTTCCGATCTCCTATCCTNN	Adapter503		*Mme*I Tnseq adapter
Adapterbarcode503B	AGGATAGGAGATCGGAAGAGCGTCGTGTAGGGAAAGAG	Adapter503		*Mme*I Tnseq adapter
Adapterbarcode504A	TTCCCTACACGACGCTCTTCCGATCTGGCTCTGANN	Adapter504		*Mme*I Tnseq adapter
Adapterbarcode504B	TCAGAGCCAGATCGGAAGAGCGTCGTGTAGGGAAAGAG	Adapter504		*Mme*I Tnseq adapter
Adapterbarcode505A	TTCCCTACACGACGCTCTTCCGATCTAGGCGAAGNN	Adapter505		*Mme*I Tnseq adapter
Adapterbarcode505B	CTTCGCCTAGATCGGAAGAGCGTCGTGTAGGGAAAGAG	Adapter505		*Mme*I Tnseq adapter
Adapterbarcode506A	TTCCCTACACGACGCTCTTCCGATCTTAATCTTANN	Adapter506		*Mme*I Tnseq adapter
Adapterbarcode506B	TAAGATTAAGATCGGAAGAGCGTCGTGTAGGGAAAGAG	Adapter506		*Mme*I Tnseq adapter
Adapterbarcode507A	TTCCCTACACGACGCTCTTCCGATCTCAGGACGTNN	Adapter507		*Mme*I Tnseq adapter
Adapterbarcode507B	ACGTCCTGAGATCGGAAGAGCGTCGTGTAGGGAAAGAG	Adapter507		*Mme*I Tnseq adapter
Adapterbarcode508A	TTCCCTACACGACGCTCTTCCGATCTGTACTGACNN	Adapter508		*Mme*I Tnseq adapter
Adapterbarcode508B	GTCAGTACAGATCGGAAGAGCGTCGTGTAGGGAAAGAG	Adapter508		*Mme*I Tnseq adapter
oKrmit-Tnseq2	CAAGCAGAAGACGGCATACGAAGCGCCTACGAGGAATTTGTATCG		*Krmit*	PCR of *Krmit* insertion tags
oAdapterPCR	AATGATACGGCGACCACCGAGATCACACTCTTTCCCTACACGACGCTCTTCC		Tnseq adapter	PCR of *Krmit* insertion tags
**Creation of the pSinS/pHlpK system**				
RepAminus1	cccAGATCTAATCATTAGGAATTGAGTCAGG	*Bgl*II	pCRS	Deletion of the *repA*ts gene to create pSinS
RepAminus2	cccAGATCTGGTTCTTGATGCTGAAACGG	*Bgl*II	pCRS	Deletion of the *repA*ts gene to create pSinS
KmR1NotI	cccGCGGCCGCGATAAACCCAGCGAACCATTTGAGGTG	*Not*I	pCRK	Deletion of the ColE1 *oriR* to create pHlpK
OTS2	cccGCGGCCGCATTAATCGCAACATCAAACCAAA		pCRK	Deletion of the ColE1 *oriR* to create pHlpK
**Theophilline-inducible *murE* and *vicR* mutants**				
Psag-RBSWE1	cccGGATCCACGGTAAGACAGAAATCGGTC	*BamH*I	pEU7742-E	Amplification of P*sag*-riboswitchE
Psag-RBSWE2	CTTGTTGTTACCTCCTTAGCAGGG		pEU7742-E	Amplification of P*sag*-riboswitchE
oRBSWE-V1	ACCAGCATCGTCTTGATGCC			
oMurE-1	CCCTGCTAAGGAGGTAACAACAAGATGATAACCATTGAACAATTATTAG		*murE* gene	Amplification of *murE* fragment for PCR-SOEing with P*sag*-riboswitchE
oMurE-2	cccGGATCCATTTAGGAAAACCCCAACATCG	*BamH*I	*murE* gene	Amplification of *murE* fragment for PCR-SOEing with P*sag*-riboswitchE
oMurE-3	ATATCAGCTAGGCTAGCACC		*murE* gene	Verification of correct PCR-SOEing
oVicR-1	CCCTGCTAAGGAGGTAACAACAAGATGAAAAAAATACTTATTGTGGATG		*vicR* gene	Amplification of *vicR* fragment for PCR-SOEing with P*sag*-riboswitchE
oVicR-2	cccGGATCCCGCCAAAATAATCATATCCCC	*BamH*I	*vicR* gene	Amplification of *vicR* fragment for PCR-SOEing with P*sag*-riboswitchE
oVicR-3	CATAAGATTTCATGTAGTACCC		*vicR* gene	Verification of correct PCR-SOEing

Table S10. Summary of the 20 publicly available GAS genomes.

GAS strain	Serotype	Size	Link to genome sequence
emm **Pattern A-C (Throat tropism)**			
**MGAS5005**	**M1T1**	**1,838,554**	**http://www.ncbi.nlm.nih.gov/nuccore/CP000017.2**
SF370	M1	1,852,441	http://www.ncbi.nlm.nih.gov/nuccore/NC_002737.1
A20	M1	1,837,281	http://www.ncbi.nlm.nih.gov/nuccore/CP003901.1
M1-476	M1	1,831,128	http://www.ncbi.nlm.nih.gov/nuccore/AP012491.2
MGAS315	M3	1,900,521	http://www.ncbi.nlm.nih.gov/nuccore/NC_004070.1
SSI-1	M3	1,894,275	http://www.ncbi.nlm.nih.gov/nuccore/NC_004606.1
Manfredo	M5	1,841,271	http://www.ncbi.nlm.nih.gov/nuccore/AM295007.1
MGAS10394	M6	1,899,877	http://www.ncbi.nlm.nih.gov/nuccore/CP000003.1
MGAS8232	M18	1,895,017	http://www.ncbi.nlm.nih.gov/nuccore/AE009949.1
MGAS9429	M12	1,836,467	http://www.ncbi.nlm.nih.gov/nuccore/CP000259.1
MGAS2096	M12	1,860,355	http://www.ncbi.nlm.nih.gov/nuccore/94991497
HKU16	M12	1,908,100	http://www.ncbi.nlm.nih.gov/nuccore/AFRY01000001.1
HSC5	M14	1,818,351	http://www.ncbi.nlm.nih.gov/nuccore/NC_021807.1
emm **pattern D (skin tropism)**			
Alab49	M53	1,827,308	http://www.ncbi.nlm.nih.gov/nuccore/CP003068.1
emm **pattern E (generalists)**			
MGAS10270	M2	1,928,252	http://www.ncbi.nlm.nih.gov/nuccore/NC_008022.1
MGAS10750	M4	1,937,111	http://www.ncbi.nlm.nih.gov/nuccore/CP000262.1
MGAS6180	M28	1,897,573	http://www.ncbi.nlm.nih.gov/nuccore/CP000056.1
**NZ131**	**M49**	**1,815,785**	**http://www.ncbi.nlm.nih.gov/nuccore/CP000829.1**
MGAS1882	M59	1,781,029	http://www.ncbi.nlm.nih.gov/nuccore/NC_017053.1
MGAS15252	M59	1,750,832	http://www.ncbi.nlm.nih.gov/nuccore/CP003116.1
